# Comparative analysis of liver transcriptome reveals adaptive responses to hypoxia environmental condition in Tibetan chicken

**DOI:** 10.5713/ab.23.0126

**Published:** 2023-08-23

**Authors:** Yongqing Cao, Tao Zeng, Wei Han, Xueying Ma, Tiantian Gu, Li Chen, Yong Tian, Wenwu Xu, Jianmei Yin, Guohui Li, Lizhi Lu, Shuangbao Gun

**Affiliations:** 1 College of Animal Science and Technology, Gansu Agricultural University, Lanzhou 730070, China; 2 State Key Laboratory for Managing Biotic and Chemical Threats to the Quality and Safety of Agro-products, Institute of Animal Husbandry and Veterinary Science, Zhejiang Academy of Agricultural Science, Hangzhou 310021, China; 3 Jiangsu Institute of Poultry Science, Yangzhou 225125, China; 4 Technology Innovation Co., Ltd., Jiangsu Institute of Poultry Science, Yangzhou 211412, China; 5 Institute of Animal Husbandry and Veterinary Medicine, Tibet Academy Agricultural and Animal Husbandry Sciences, Lhasa 850004, China; 6 China-Ukraine Joint Research Center for Protection, Exploitation and Utilization of Poultry Germplasm Resources, Hangzhou 310021, China

**Keywords:** Hypoxia Adaptation, Lipid Metabolism, Liver, Tibetan Chicken, Transcriptome

## Abstract

**Objective:**

Tibetan chickens, which have unique adaptations to extreme high-altitude environments, exhibit phenotypic and physiological characteristics that are distinct from those of lowland chickens. However, the mechanisms underlying hypoxic adaptation in the liver of chickens remain unknown.

**Methods:**

RNA-sequencing (RNA-Seq) technology was used to assess the differentially expressed genes (DEGs) involved in hypoxia adaptation in highland chickens (native Tibetan chicken [HT]) and lowland chickens (Langshan chicken [LS], Beijing You chicken [BJ], Qingyuan Partridge chicken [QY], and Chahua chicken [CH]).

**Results:**

A total of 352 co-DEGs were specifically screened between HT and four native lowland chicken breeds. Gene ontology and Kyoto encyclopedia of genes and genomes enrichment analyses indicated that these co-DEGs were widely involved in lipid metabolism processes, such as the peroxisome proliferator-activated receptors (PPAR) signaling pathway, fatty acid degradation, fatty acid metabolism and fatty acid biosynthesis. To further determine the relationship from the 352 co-DEGs, protein-protein interaction network was carried out and identified eight genes (*ACSL1*, *CPT1A*, *ACOX1*, *PPARC1A*, *SCD*, *ACSBG2*, *ACACA*, and *FASN*) as the potential regulating genes that are responsible for the altitude difference between the HT and other four lowland chicken breeds.

**Conclusion:**

This study provides novel insights into the molecular mechanisms regulating hypoxia adaptation via lipid metabolism in Tibetan chickens and other highland animals.

## INTRODUCTION

Owing to evolutionary and selection pressures, humans and animals living in high-altitude areas have experienced heritable changes in behavior [1], morphological structure [2], physiological and biochemical features [3,4], and molecular regulatory pathways [5] for survival in harsh environments. The Tibetan Plateau with an average altitude of 4,500 m and an area of more than 2 million km^2^ is the largest highland area in the world. The oxygen partial pressure at an altitude of 4,500 m is approximately 52% of the value at sea level [6], which causes a reduction in the oxygen content of the arterial blood and tissue hypoxia in animals migrating to highlands [7]. Compared to their low-altitude relatives, mammals and birds native to high altitudes exhibit a series of derived characteristics of respiratory, cardiovascular, and metabolic traits [8–10].

The Tibetan chicken, an indigenous breed of the Qinghai-Tibetan Plateau, can adapt to extreme environments at high altitudes, including high hatchability under hypoxic incubation [11,12]. Numerous positively selected and differentially expressed genes (DEGs) at high altitudes have been identified in Tibetan chickens using high-throughput sequencing [13,14]. For example, egl-9 family hypoxia inducible factor 1 (*EGLN1*), as an important candidate gene in high altitude human and animal, encodes prolyl hydroxylase 2 serving as a repressor to hypoxia inducible factor α (HIF-1α) that plays important roles in hypoxic responses and adaptation [15–17]. In heart tissues of Tibetan chicken, *EGLN1* downregulates to eliminate the inhibiting effects to HIF-1α [13]. In the subunit 6 of the mitochondrial FiF0-ATP synthase gene (*ATP-6*) of Tibetan chickens, an amino acid substitution resulting from single nucleotide polymorphisms was speculated to ease energy conversion for adaptation to high altitude areas [18]. In terms of epigenetics, the methylation patterns of Tibetan chicken were different from those of low-altitude breeds, in which the former’s methylated cytosines in CG contexts, the most crucial and studied methylation type, were 8.03% lower than those of the latter [19]. According to recent research, differentially methylated genes are involved in regulating the vascular system and ion transport [20]. However, the mechanisms underlying the metabolic adaptation in Tibetan chicken remain unclear.

RNA-sequencing (RNA-Seq) is a technique use to quantitatively describe DEGs that play essential regulatory roles in cellular processes [21]. Recently, it has been used to analyze genes related to liver lipid metabolism in chickens to understand the mechanisms of lipid-related metabolism [22]. Changes in metabolic patterns may be related to the ATP supply to organisms, in which the final product from aerobic respiration and glycolysis is easily affected by the oxygen content of the environment [23]. In this study, we investigated the transcriptomic data from the liver tissues of chickens at different altitudes under different hypoxic environmental conditions using RNA-seq technology to elucidate the regulatory mechanisms that may further elucidate the regulation of genes involved in hypoxic adaptation in other animals and providing molecular therapeutics for human altitude sickness in metabolism.

## MATERIALS AND METHODS

### Animals and sample collection

**Figure 1:**
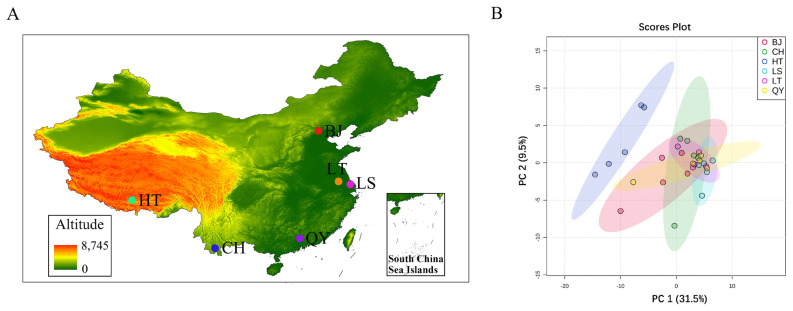
Localities and transcriptome replicates of six chicken populations. (A) Sampling locations. Native Tibetan chickens (HT) originated from altitudes over 4,000 m. Langshan chickens (LS), Beijing You chickens (BJ), Qingyuan Partridge chickens (QY) originated from altitudes under 50 m. Chahua chickens (CH) originated from altitudes under 800 m. Lowland Tibetan chickens (LT) have grown and generated in altitudes under 50 m for about 20 years. (B) Differences in transcriptome replicates of different altitude chicken livers based on principal components analysis (PCA) analysis. The X-coordinate is the first principal component and the Y-coordinate is the second principal component.

Total 30 hens of different six populations at 300-day-old were selected from locations at different altitudes (Figure 1A). Tibetan chickens were selected from two different populations. Native Tibetan chickens (HT, n = 5) were obtained from the Tibetan Autonomous Region of China. Lowland Tibetan chickens (LT, n = 5), Langshan chickens (LS, n = 5), Beijing You chickens (BJ, n = 5), Qingyuan Partridge chickens (QY, n = 5), and Chahua chickens (CH, n = 5) were obtained from the National Chick Genetics Resources in Yangzhou City, Jiangsu Province, China. The animals used in this study were raised in accordance with the National Standards of Laboratory Animal Guidelines for Ethical Review of Animal Welfare. Hens were humanely killed after 12 h of fasting and the liver tissues harvested from the carcasses were immediately frozen in liquid nitrogen. All animals and samples used in this study were collected according to the guidelines for the care and use of experimental animals established by the Animal Use Committee of Zhejiang Academy of Agricultural Sciences (No. 20-022). The samples were delivered in dry ice and stored at −80°C until RNA-seq.

### RNA extraction

Total RNA was extracted from the chicken liver tissue using TRIzol reagent (Invitrogen, Carlsbad, CA, USA). Total RNA quality was checked using a 2100 Bioanalyzer (Agilent, Santa Clara, CA, USA). High-quality RNA samples (OD260/280 = 1.8–2.2) were used to construct sequencing library.

### Preparation, and Illumina Hiseq sequencing

RNA-seq transcriptome libraries were generated following TruSeqTM RNA sample preparation Kit from Illumina (San Diego, CA, USA), using 1 μg of total RNA per sample. mRNA was filtered through polyA selection using by oligo(dT) beads and fragmented into small pieces. The cDNA synthesis, end repair, A-base addition and ligation of Illumina-indexed adaptors were performed according to the manufacturer’s protocol. Libraries were size-selected for cDNA fragments of 200 to 300 bp in length followed by polymerase chain reaction (PCR) amplified. The PCR condition are as follow: 98°C for 3 minutes; 15 cycles of: 98°C for 20 seconds, 60°C for 15 seconds, 72°C for 30 seconds; 72°C for 5 minutes; hold at 4°C. After quantification using TBS380, Paired-end libraries were sequenced using Illumina NovaSeq 6000 sequencing (150 bp×2; Shanghai BIOZERON Co., Ltd., Shanghai, China). The RNA-seq raw data are available in the NCBI Short Read Archive (SRA) database under accession number PRJNA 950191.

### Reads quality control and mapping

The raw reads were dynamically removed the 3′ end, linker sequences, and low-mass sequences by Trimmomatic with parameters (SLIDINGWINDOW:4:15 MINLEN:75) (version 0.36 http://www.usadellab.org/cms/uploads/supplementary/Trimmomatic), after which clean reads were obtained. FastQC software was used to calculate GC content, Q20 and Q30. The clean reads were separately aligned to the chicken reference genome (https://www.ncbi.nlm.nih.gov/genome/?term=Gallus+gallus) in orientation mode using hisat2 (https://ccb.jhu.edu/software/hisat2/index.shtml) software.

### Differential expression analysis

To identify the DEGs between different groups, the expression level of each gene was calculated using the fragments per kilobase of exons per million mapped reads method. The statistical package edgeR (empirical analysis of Digital Gene Expression in R, http://www.bioconductor.org/packages/release/bioc/html/edgeR.html/) was used for the differential expression analysis. DEGs were selected using the following criteria: logarithmic fold change was greater than 2 and false discovery rate should be less than 0.05. To understand the functions of the DEGs, GO functional enrichment and Kyoto encyclopedia of genes and genomes (KEGG) pathway analyses were performed using Goatools (https://github.com/tanghaibao/Goatools) and KOBAS (http://kobas.cbi.pku.edu.cn/home.do), respectively. DEGs were significantly enriched in GO terms and metabolic pathways when their Bonferroni-corrected p-value was less than 0.05.

### Functional enrichment

Gene ontology (GO) functional enrichment and KEGG pathway analyses were performed using Goatools (http://github.com/tanghaibao/Goatools) and KOBAS (http://bioinf.wehi.edu.au/software/goseq/), respectively. The protein–protein interaction (PPI) network of DEGs was constructed using the STRING web server (http://www.string-db.org/) by calculating the combined score (threshold: score >0.9).

### Validation of differentially expressed genes by real time-quantitative polymerase chain reaction

To confirm the repeatability and accuracy of gene expression data from RNA-Seq, real time-quantitative PCR (RT-qPCR) was performed to randomly detect DEGs using TB Green Premix according to the manufacturer’s instructions (TAKARA, Dalian, China). The glyceraldehyde-3-phosphate dehydrogenase gene (*GAPDH*) was set as reference gene and the relative expression level was calculated by the 2^−ΔΔCT^ method. RT-qPCR experiments were performed with five replicates and three technical replicates. The primer sequences for of these DEGs are listed in Table 1.

### Statistical analysis

Data are expressed as mean±standard deviation. All statistical analyses were performed using SPSS software (version 25.0; Chicago, IL, USA), and figures were generated using GraphPad Prism software (version 8.0). Statistical significance was considered when p-value was less than 0.05.

## RESULTS

### Identification of RNA-Seq data

Raw reads from the six chicken populations (Figure 1A) were collected using Illumina NovaSeq 6000 sequencing (Supplementary Table S1). A total of 3,078,778,390 raw reads with an average of 102,625,946 reads and 2,637,622,286 clean reads, with an average of 879,207,432 reads were obtained. Over 90% of the clean reads mapped to the chicken reference genome, and the average Q30 value, was greater than 90.88%, indicating that the samples were of good quality. To assess intra-population duplication and inter-population differences, we conducted principal components analysis (PCA) on the read_counts of all samples. The results indicated that the high and low altitudes were significantly isolated (Figure 1B).

### Analysis of differentially expressed genes

**Figure 2:**
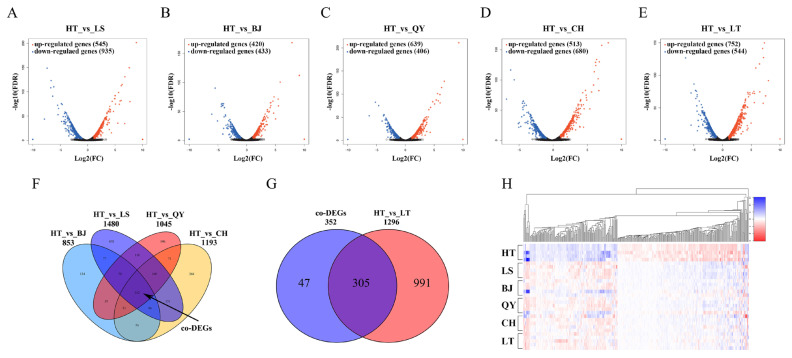
mRNA expression changes in six chicken population in different altitudes. (A–E) Volcano plots showing up- and down-regulated mRNA expression between HT and LT, LS, BJ, QY, and CH groups at different altitudes. (F) Venn diagram of overlapping co-DEGs mRNAs between HT and LS, BJ, QY, CH groups. (G) Venn diagram of overlapping DEGs mRNAs between co-DEGs and HT vs LT. (H) Heat map of co-DEGs mRNAs between HT, LT, LS, BJ, QY and CH groups. Red and blue represented Up- and down-regulated expressions of mRNAs, respectively. HT, native Tibetan chicken; LS, Langshan chicken; BJ, Beijing You chicken; QY, Qingyuan Partridge chicken; CH, Chahua chicken; DEG, differentially expressed genes.

Reads per kilobase of transcript per million mapped reads were use to quantify genes and transcripts from the six chicken populations distributed at different altitudes. DEGs were filtered with a threshold of |fold change|≥2 and p-value ≤0.05. We compared the HT and five lowland populations. In the five comparisons (HT vs LS, HT vs BJ, HT vs QY, HT vs CH, and HT vs LT), 1,480, 853, 1,045, 1,193, and 1,296 DEGs were obtained, among which the upregulated and downregulated genes were 935/545, 433/420, 406/639, 680/513, and 544/752 (Figure 2A–2E). Complete information on all the DEGs is listed in Supplementary Table S2 to S6. To further improve the credibility of DEGs and reduce the effects of breeds, we focused on 352 DEGs coexisted (co-DEGs) in the comparison between HT and the four native lowland breeds (LS, BJ, QY, and CH) and defined them as co-DEGs (Figure 2F). Among the 352 co-DEGs, 305 changes in gene expression were observed when Tibetan chickens were raised and generated in the lowlands for approximately 20 years (Figure 2G). The co-DEG expression profile of the 30 samples is shown as a heat map based on the fold change, which displayed good repeatability in each population (Figure 2H). The expression pattern of co-DEGs in LT was more similar to that in the four lowland breeds than that in HT.

### Functional analysis of co-DEGs

**Figure 3:**
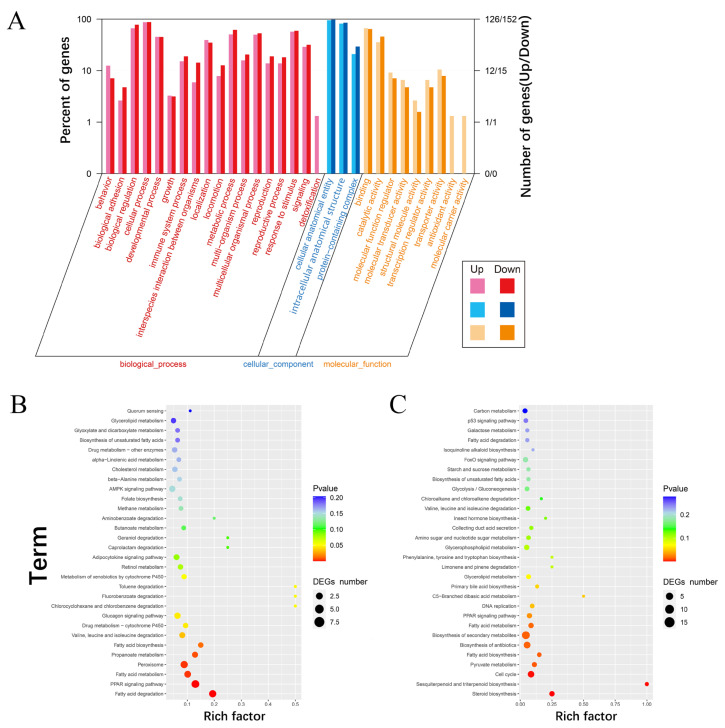
Gene ontology (GO) and KEGG pathway enrichment analysis of co-DEGs. (A) Go classification of co-DEGs. The red color indicates biological process (BP); the blue color indicates cellular component (CC); the orange color indicates molecular function (MF). (B) KEGG pathway enrichment of up-regulated genes among co-DEGs. (C) KEGG pathway enrichment of down-regulated genes among co-DEGs. KEGG, Kyoto encyclopedia of genes and genomes; DEGs, differentially expressed genes.

To further elucidate the functions of the 352 co-DEGs, GO, and KEGG analyses were performed. The up-regulated genes among co-DEGs were significantly enriched in 18 terms in biological process (BP), 9 terms in molecular function (MF) and 3 terms in cellular component (CC) (Figure 3A; Supplementary Table S7). And the down-regulated genes were significantly enriched in 17 terms in BP, 7 terms in MF and 3 terms in CC (Figure 3A; Supplementary Table S8). The top 10 GO terms of up-regulated genes were cellular anatomical entity (GO:0110165), cellular process (GO:0009987), intracellular anatomical structure (GO:0005622), binding (GO: 0005488), biological regulation (GO:0065007), response to stimulus (GO:0050896), metabolic process (GO:0008152), multicellular organismal process (GO:0032501), developmental process (GO:0032502) and localization (GO:0051179), among which 7 terms were subject to BP and remaining 3 terms were subject to CC and MF. The top 10 GO terms of down-regulated genes were cellular anatomical entity (GO: 0110165), cellular process (GO:0009987), intracellular anatomical structure (GO:0005622), biological regulation (GO: 0065007), binding (GO:0005488), metabolic process (GO: 0008152), response to stimulus (GO:0050896), multicellular organismal process (GO:0032501), catalytic activity (GO: 0003824) and developmental process (GO:0032502), among which 6 terms were subjected to BP. In addition, KEGG analysis showed that up-regulated genes among co-DEGs were involved in 126 pathways, among which 13 pathways were significantly enriched (p<0.05) (Figure 3B; Supplementary Table S9). The most significant pathways included peroxisome proliferator-activated receptor (PPAR) signaling pathway, fatty acid degradation, fatty acid metabolism and fatty acid biosynthesis. The down-regulated genes involved in 127 pathways, among which 11 pathways were significantly enriched (p<0.05) (Figure 3C; Supplementary Table S10).

### Analysis of protein-protein interaction network for co-DEGs

**Figure 4:**
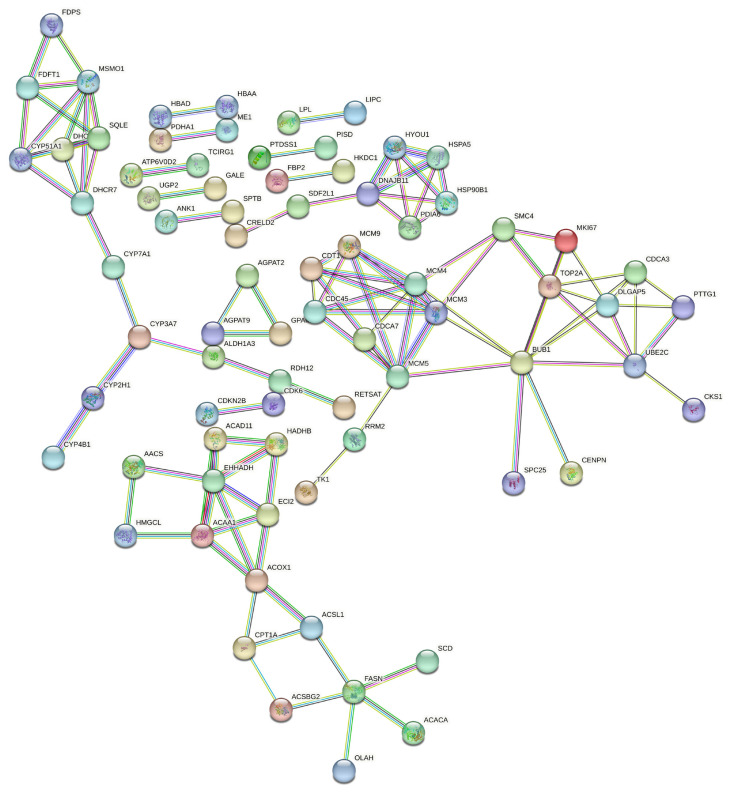
The altitude-related protein-protein interaction (PPI) network in chicken. Nodes represent proteins. Edges represent protein-protein associations. The relationship between the 2 proteins is expressed through the thickness of the line; the thicker the line, the closer the relationship.

To further explore the potential key genes regulating adaptive responses to hypoxia environmental condition from the above 352 co-DEGs, an altitude-related PPI network in chickens was constructed using the STRING database and the minimum required interaction score was set at 0.900. Acyl-CoA synthetase long chain family member 1 (*ACSL1*), carnitine palmitoyltransferase 1a (*CPT1A*), stearoyl-CoA desaturase (*SCD*), acyl-CoA oxidase 1 (*ACOX1*), acyl-CoA synthetase bubblegum family member 2 (*ACSBG2*), acetyl-CoA carboxylase alpha (*ACACA*), and fatty acid synthase (*FASN*) (red circle in Figure 4), which were involved in fatty acid synthesis (FAS) and metabolism, showed a high degree of connection (Figure 4).

### RT-qPCR validation of co-DEGs

**Figure 5:**
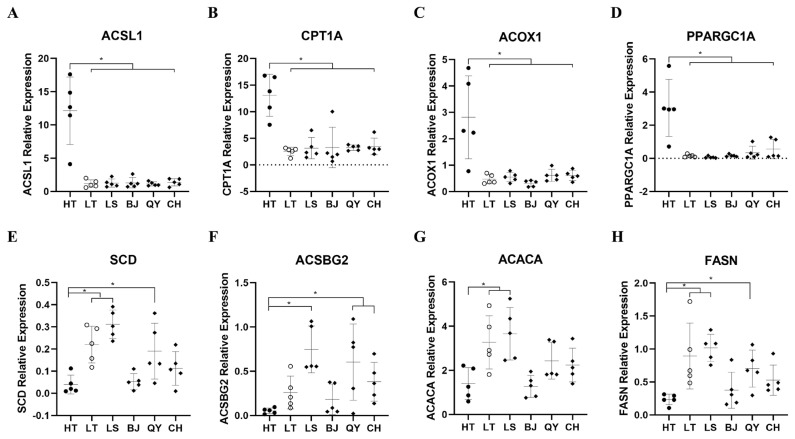
Validation of co-DEGs expression at HT, LT, LS, BJ, QY, and CH groups. RT-qPCR validation of *ACSL1*, *CPT1A*, *ACOX1*, *PPARGC1A*, *SCD*, *ACSBG2*, *ACACA*, and *FASN* gene expression levels in liver samples between HT, LT, LS, BJ, QY, and CH groups. The mRNA expression levels at LT, LS, BJ, QY, and CH groups were normalized to the value at HT group. Error bars indicate the mean±standard deviation of triplicate experiments. HT, native Tibetan chicken; LS, Langshan chicken; BJ, Beijing You chicken; QY, Qingyuan Partridge chicken; CH, Chahua chicken. * p<0.05.

To validate the RNA-Seq transcriptome data, eight genes (*ACSL1*, *CPT1A*, *ACOX1*, peroxisome proliferative activated receptor, gamma, coactivator 1 alpha [*PPARGC1A*], *SCD*, *ACSBG2*, *ACACA*, *FASN*) selected from the three of the most significant pathways, namely fatty acid biosynthesis, fatty acid degradation and the PPAR signaling pathway were measured by RT-qPCR (Figure 5). The RT-qPCR results were consistent with the transcriptome sequencing data, indicating that the RNA-seq data were reliable and accurate. Moreover, the expression levels of these genes in LT were closer in the four native lowland breeds than in the HT breeds. Interestingly, the four up-regulated genes are associated with to fatty acid degradation, whereas the four down-regulated genes are involved in fatty acid biosynthesis. This suggests that the divergence of fatty acid metabolism is the key difference between Tibetan and lowland chickens.

## DISCUSSION

The liver is an essential metabolic organ and the central link for carbohydrate, lipid, and protein metabolism [24]. Excessive nutrients in the liver can be metabolized into glycogen and lipids, which produce energy to maintain peripheral tissue function. These processes require a considerable amount of oxygen, resulting in a steep oxygen gradient throughout the hepatic lobules [25]. Studies have shown that hypoxic adaptation is a complex regulatory mechanism, and its occurrence and development are closely related to hepatic function [25]. Therefore, hypoxia may have an impact on the liver, and the expression of key DEGs in the liver tissue may also be related to hypoxia.

Tibetan chickens, which have a long history of living at high altitude and have experienced strong selection, have well-developed adaptations to low oxygen conditions [12]. Animals that migrate to high altitudes are exposed to hypoxia, which results in liver metabolic accelerated [26,27]. However, the correlation between the liver tissue and adaptations to hypoxia in Tibetan chickens remains limited. In the present study, transcriptome analysis was performed on highland and lowland chicken liver samples to investigate possible regulatory mechanisms for adaptation to high-altitude hypoxia. We believe that the 352 co-DEGs identified by comparing HT vs LS, HT vs BJ, HT vs QY, and HT vs CH are of great interest as potential candidate genes for high-altitude adaptation in Tibetan chickens.

Hypoxia adaptation is a complex process involving multiple genes and various pathways [28]. However, adaptation in metabolism has also come under interest, with lipid metabolism attracting the most attention. A previous study has shown that several genes related to fatty acid oxidation were down-regulated in rats exposed to hypoxia, including peroxisome proliferator activated receptor alpha (*PPARA*) and carnitine palmitoyltransferase 1 (*CPT-1*) [29]. Under hypoxia, fat anabolic pathways are up regulated, which is mediated in part by the upregulation of the PPAR pathway [30]. There were 1,480, 853, 1,045, and 1,193 DEGs between Tibetan chicken and the four lowland chicken breeds, respectively, which means that, except for the difference in altitude, the differences in varieties were still present. To eliminate differences other than those caused by altitude, we identified 352 DEGs by intersecting the four differences. Of the up-regulated and down-regulated genes among co-DEGs, 126 and 127 pathways identified between the HT, LS, BJ, QY, and CH chickens were involved in the PPAR signaling pathway, fatty acid degradation, fatty acid metabolism and fatty acid biosynthesis. The PPAR signaling pathway is generally considered to be related to hypoxia adaptation, especially by inhibiting lipid accumulation in hepatocytes and mediating the activation of endothelial nitric oxide synthase [31,32]. A previous study demonstrated that many genes screened under hypoxic condition were involved in lipid metabolism [33]. This suggests that Tibetan chickens have novel mechanisms for adapting to hypoxia in terms of lipid metabolism.

To gain further insight into the possible genes regulating hypoxic adaptation, a PPI network analysis of 352 co-DEGs was carried out. Several genes responsible for lipid metabolism including *ACSL1*, *CPT1A*, *SCD*, *ACOX1*, *ACSBG2*, *ACACA*, and *FASN* showed a higher degree of connection than other co-DEGs in the PPI network. PPAR-α coactivators, such as *ACSL1*, *CPT1A*, *ACOX1*, *PPARGC1A*, play an important role in regulating the redox environment of cells by upregulating the functions of antioxidant genes and their derivatives and interacts with PPARs to increase fatty acid oxidation (FAO) [34,35]. Carnitine palmitoyl transferase 1 (CPT1), belonging to the CPT1 family, is the key rate-controlling enzyme of FAO, which regulates FAO to facilitate adaptation to circumstance and is required for the transport of long chain fatty acid into mitochondria [36,37]. Similar to CPT1, PPARGC1A is, a key nuclear transcription coactivator that, can bind to many different transcription factors, participate in a series of orderly metabolic processes, and play an important role in the regulation of sugar metabolism and fatty acid oxidation [38]. In our study, the expression levels of *ACSL1*, *CPT1A*, *ACOX1*, and *PPARGC1A* which promote FAO, were significantly higher in Tibetan chickens than in lowland chicken breeds and Tibetan chickens grown in lowlands. In contrast, the expression patterns of the genes regulating FAS were also differed in different populations. Suppression of SCD1 by PPARα, plays an important role in regulating lipogenesis and triglyceride synthesis in the liver [28,31]. *FASN* and *ACACA* are two key genes that regulate FAS [39]. Previous studies have also reported that the expression of lipogenic genes such as fatty acid synthase encoded by *FASN* and acyl-CoA carboxylase encoded by *ACACA* can be induced by FAS [40]. *ACSBG2*, a member of *ACSBG* gene family, is related to lipid metabolism and reported association with fat deposition in chickens [41,42]. In our results, the mRNA levels of the genes involved in FAO were significantly higher in Tibetan chickens than in lowland chickens, whereas the mRNA levels of the genes involved in FAS showed downward trends, suggesting that, compared to lowland chickens, the livers of Tibetan chickens tend to be fat-depleted rather than fat-deposited. In addition, the expression patterns of these genes in Tibetan chickens raised and generated in the lowlands were closer to those in lowland chickens. However, further studies are required to elucidate this mechanism.

In summary, 305 DEGs were identified in the liver tissues of the HT, LS, BJ, QY, and CH chickens using RNA-Seq. Several key genes (*ACSL1*, *CPT1A*, *ACOX1*, *PPARGC1A*, *SCD*, *ACSBG2*, *ACACA*, and *FASN*) were screened and found to be predominantly involved in the PPAR signaling pathway, fatty acid degradation, fatty acid metabolism and fatty acid biosynthesis. These results provide novel insights into the molecular mechanism regulating hypoxia adaptation by lipid metabolism in highland animals, including humans, and a theoretical basis for the development of molecular therapies for human altitude disease.

## Figures and Tables

**Table 1:** Primers used in the study

Gene	Genbank Accession	Primer Sequences (5′→3′)	Annealing (°C)
*GAPDH*	NM_204305.2	CGATCTGAACTACATGGTTTAC TCTGCCCATTTGATGTTGC	60
*ACSL1*	NM_001012578.2	GCTGCCGGAGGTTCCAG CACCAAAACCCACCAGGGTA	60
*CPT1A*	NM_001012898.1	GCTCACTACCGAGACATGGG GACCGGACGGTTTCAGTTCT	60
*ACOX1*	NM_001006205.2	GGCGAAAGGAGATCGAGGC GCCGTCCACGATGAACAAAG	60
*PPARGC1A*	NM_001006457.2	TCTCAGAAAGGGTCTCGTTGC CCAGAGCAGCACACTCGAT	60
*SCD*	NM_204890.2	CCAGAAGCTGGACCTGAGTG GGGCTTGTAGTAGTATCTCCGCT	60
*ACSBG2*	NM_001397597.1	AATGGCAGCTTTGGAGGTGT TTCTGTTTGGGTGGGAGGTG	60
*ACACA*	NM_205505.2	GAATCCAGAAGGGCCAACGA TCCAAGGGAGCAGCTTTTGT	60
*FASN*	NM_205155.4	ATGGAAGCAATGGGCGTGAA TCGATGGTACGGAAGTTGCT	60

*GAPDH*, glyceraldehyde-3-phosphate dehydrogenase; *ACSL1*, Acyl-CoA synthetase long chain family member 1; *CPT1A*, carnitine palmitoyltransferase 1a; *ACOX1*, acyl-CoA oxidase 1; *PPARGC1A*, peroxisome proliferative activated receptor, gamma, coactivator 1 alpha, peroxisome proliferative activated receptor, gamma, coactivator 1 alpha; *SCD*, stearoyl-CoA desaturase; *ACSBG2*, acyl-CoA synthetase bubblegum family member 2; *ACACA*, acetyl-CoA carboxylase alpha; *FASN*, fatty acid synthase.

## SUPPLEMENTARY MATERIAL

**Supplementary Table S1**. Summary of RNA-seq quality data of 30 chicken samples

**Supplementary Table S2**. Different expression genes between HT and LS

**Supplementary Table S3**. Different expression genes between HT and BJ

**Supplementary Table S4**. Different expression genes between HT and QY

**Supplementary Table S5**. Different expression genes between HT and CH

**Supplementary Table S6**. Different expression genes between HT and LS

**Supplementary Table S7**. Go classification of up-regulated co-DEGs

**Supplementary Table S8**. Go classification of down-regulated co-DEGs

**Supplementary Table S9**. KEGG pathway enrichment of up-regulated genes among co-DEGs

**Supplementary Table S10**. KEGG pathway enrichment of down-regulated genes among co-DEGs
