# Supplementary material for: Comparative analysis of liver transcriptome reveals adaptive responses to hypoxia environmental condition in Tibetan chicken
Source: Anim Biosci. 2023 Aug 23;37(1):28–38. doi: 10.5713/ab.23.0126 (PMC10766467; doi:10.5713/ab.23.0126)
Supplement: Supplementary file 1 [file ab-23-0126-Supplementary-Table-1.pdf]

Table S1 Summary of RNA-seq quality data of 30 chicken samples

samples	Raw data	Clean data	Clean base	Mapped rate(%)	Q20(%)	Q30(%)	CG(%)
HT1	1.02E+08	89889648	1.34E+10	92.78	98.51	96.09	46.67
HT2	1.57E+08	1.35E+08	2.02E+10	92.82	98.32	95.67	47.19
HT3	1.32E+08	1.12E+08	1.67E+10	92.46	98.5	96.11	46.95
HT4	1.19E+08	1.04E+08	1.55E+10	93.43	98.41	95.84	47.27
HT5	1.02E+08	85245610	1.27E+10	91.26	98.06	95.04	47.81
LT1	91774428	82538026	1.23E+10	93.04	98.49	96.03	47.02
LT2	1.1E+08	97197980	1.45E+10	92.58	98.28	95.57	47.01
LT3	73414704	64797642	9.67E+09	90.85	95.92	90.88	47.79
LT4	90905394	80113972	1.2E+10	93.14	98.47	96.03	47.12
LT5	1.1E+08	1.01E+08	1.51E+10	91.84	98.49	96.05	46.99
LS1	76486652	59716808	8.92E+09	91.93	98.29	95.72	47.75
LS2	83976432	60791014	9.08E+09	90.94	98.12	95.34	47.83
LS3	49702760	40070242	5.98E+09	92.07	97.74	94.43	47.37
LS4	96214502	82738076	1.24E+10	93.48	98.09	95.22	47.06
LS5	67583342	61728556	9.22E+09	93.5	97.8	94.5	47.47
BJ1	90295048	75804474	1.13E+10	92.84	97.93	94.87	47.02
BJ2	80301698	75688632	1.13E+10	94.06	97.76	94.42	46.81
BJ3	1.09E+08	89721642	1.34E+10	94.26	97.95	94.97	47.56
BJ4	58827914	51628760	7.71E+09	93.62	97.79	94.55	46.87
BJ5	93184732	83213088	1.24E+10	94.32	98.41	96.00	47.5
QY1	1.09E+08	1E+08	1.5E+10	93.21	98.23	95.43	46.77
QY2	95187370	76768028	1.15E+10	93.7	98.57	96.28	47.25
QY3	1.54E+08	1.35E+08	2.02E+10	93.59	98.29	95.59	47.42
QY4	1.22E+08	1.07E+08	1.61E+10	93.38	98.59	96.30	46.89
QY5	1.5E+08	94585972	1.41E+10	93.35	98.59	96.34	47.84
CH1	1.44E+08	1.26E+08	1.88E+10	92.82	98.27	95.47	47.57
CH2	89589748	80465108	1.2E+10	93.2	98.40	95.86	47.28
CH3	1.16E+08	1.04E+08	1.55E+10	93.89	98.52	96.13	46.73
CH4	1.01E+08	87507434	1.31E+10	92.39	98.37	95.76	46.68
CH5	1.1E+08	94481444	1.41E+10	92.39	98.45	95.98	47.21
