# Supplementary material for: Comparative analysis of liver transcriptome reveals adaptive responses to hypoxia environmental condition in Tibetan chicken
Source: Anim Biosci. 2023 Aug 23;37(1):28–38. doi: 10.5713/ab.23.0126 (PMC10766467; doi:10.5713/ab.23.0126)
Supplement: Supplementary file 2 [file ab-23-0126-Supplementary-Table-2.pdf]

Table S2

Geneid	HT1	HT2	HT3	HT4	HT5	LS1	LS2	LS3	LS4	LS5	logFC	type	pvalue
CLC2DL4	16.02	15.421	30.654	16.718	4.623	1.637	2.085	1.568	1.706	0.586	-3.45916	down	1.66E-45
LOC12111	3.334	4.314	4.976	0.771	0.913	1.042	0.777	0.272	0.442	1.206	-1.93467	down	5.42E-14
LOC1211C	2.719	2.124	1.201	0.265	0.41	0.656	0.811	0.284	0.371	0.885	-1.1586	down	4.27E-06
LOC77646	21.883	20.546	22.844	2.642	1.25	2.952	1.076	0.413	0.403	0.495	-3.69415	down	2.00E-49
LOC1211C	2.142	1.796	2.87	0.731	1.053	0.856	0.339	0.532	0.135	0.577	-1.81459	down	2.05E-12
LOC1211C	0.657	0.968	1.156	0.236	0.408	0.538	0.194	0.163	0.083	0.708	-1.02033	down	0.000334
LOC11253	5.471	4.844	26.895	5.635	2.36	2.441	1.803	1.127	0.888	1.035	-2.63087	down	1.55E-26
LOC12111	1.941	2.15	9.188	1.121	0.395	1.221	2.514	0.694	0.886	1.223	-1.17757	down	2.04E-05
LOC12111	0.514	0.62	0.011	0.033	0.014	0.473	0.061	0.836	0	1.054	1.020939	up	2.64E-05
CD69L	18.257	17.274	8	0.32	0.614	0.217	0.16	0.33	0.117	3.122	-3.49254	down	1.71E-49
SHANK3	1.733	1.677	3.229	3.177	3.394	0.974	0.506	1.602	0.739	0.923	-1.47648	down	5.60E-10
LOC1211C	1.419	1.132	2.508	2.285	2.131	0.835	0.332	0.977	0.391	0.747	-1.52812	down	5.59E-10
FBXO18	8.446	8.389	4.878	8.169	9.957	3.229	5.033	4.505	3.452	2.686	-1.07521	down	4.13E-06
ASB13	11.989	11.046	11.358	7.303	7.016	4.175	4.817	4.336	2.932	4.086	-1.25933	down	9.35E-08
PFKFB3	2.176	2.406	3.906	3.272	3.183	4.404	19.424	5.65	4.346	3.508	1.320654	up	4.93E-12
ITIH5	1.822	2.306	5.37	3.77	2.016	0.855	0.657	1.46	1.278	0.834	-1.58704	down	5.02E-11
ELAPOR2	2.989	3.226	1.334	0.728	1.061	5.252	5.341	19.411	20.681	19.088	2.900814	up	1.49E-42
ORC5	0.592	0.753	0.547	0.66	0.955	1.306	2.69	1.282	1.847	1.841	1.352976	up	9.85E-12
ABCD2	39.703	42.832	39.756	27.59	69.468	20.254	10.319	23.589	17.031	20.875	-1.25241	down	1.93E-07
ALG12	12.218	11.172	12.612	15.737	13.32	53.89	36.11	23.015	52.731	41.912	1.674313	up	5.42E-18
CRELD2	16.486	14.861	20.903	18.59	14.191	122.666	57.359	32.135	115.739	82.571	2.271149	up	1.23E-29
LOC42934	0.058	0.036	0.005	0.017	0.006	2.003	0.367	1.681	3.698	2.133	6.282632	up	#######
TRABD	6.048	5.907	9.455	4.535	6.353	20.646	8.69	18.771	20.983	18.303	1.435932	up	9.48E-15
ARL8BL	0.035	0.228	1.109	0	0.25	0.203	0.052	0.077	0	0.092	-1.92316	down	2.93E-05
IQUB	0.993	0.449	2.583	0.444	0.465	0.255	0.219	0.322	0.392	0.347	-1.68129	down	3.50E-09
CPED1	3.776	3.847	5.473	5.571	5.853	0.96	0.602	1.095	0.824	0.763	-2.52906	down	2.35E-27
FOXP2	9.342	8.975	12.218	7.272	8.337	3.941	5.569	5.752	2.786	2.998	-1.13241	down	2.45E-06
SLC38A4	261.667	264.71	386.806	305.956	434.979	138.65	212.371	162.542	153.909	154.073	-1.00965	down	3.88E-05
AMIGO2	1.022	1.06	1.593	1.993	2.793	0.506	0.472	0.48	0.486	0.502	-1.78831	down	6.69E-13
SRGAP1	6.908	6.068	5.45	1.341	3.581	0.674	0.805	1.023	0.71	1.347	-2.35524	down	3.44E-24
TSPAN8	0.838	0.763	1.015	0.629	1.309	1.431	2.947	0.804	1.954	2.26	1.044097	up	2.27E-07
LGR5	0.702	0.822	0.36	0.905	0.909	1.464	2.61	0.745	1.254	1.542	1.041096	up	7.17E-08
LOC1211C	1.294	1.397	2.113	0.585	1.728	0.565	0.291	0.857	0.579	0.598	-1.29872	down	9.19E-05
ACSS3	14.792	15.982	24.455	18.351	32.236	10.762	6.734	9.402	10.061	13.081	-1.08033	down	1.59E-05
PPFIA2	1.5	1.318	2.114	2.201	1.631	0.563	0.529	0.646	0.591	0.764	-1.50108	down	2.49E-10
MRPL42	6.038	5.043	3.946	4.993	4.485	1.823	2.731	1.99	2.659	2.92	-1.01503	down	2.45E-05

SOCS2	39.881	35.397	32.19	30.561	21.379	10.245	4.46	10.529	4.905	18.807	-1.70336	down	2.02E-13
TMCC3	1.068	1.135	3.019	1.988	2.977	0.528	0.638	0.789	0.454	0.472	-1.82029	down	3.68E-14
GAS2L3	0.237	0.183	0.572	0.315	0.079	1.634	1.024	0.431	0.617	0.387	1.558798	up	8.83E-15
TMEM140	11.304	11.298	13.268	13.195	12.776	6.289	5.86	6.947	6.488	5.009	-1.01524	down	3.46E-05
LOC42788	2.828	2.523	1.719	3.084	4.224	1.496	1.694	0.794	1.174	1.629	-1.08246	down	8.28E-05
HIST1H2B	2.92	2.73	1.329	2.143	6.321	1.973	1.563	0.69	1.036	1.652	-1.15878	down	0.000182
HIST1H4B	0.406	0.159	0.387	0.333	1	0.472	0.364	0	0	0.107	-1.27239	down	0.013367
HIST1H46	0.241	0.263	0	0.198	3.553	0	0.482	0.177	0.479	0.425	-1.44193	down	0.000804
LOC10085	0.424	0.324	0.282	0.349	0.799	1.029	0.847	0.468	1.967	1.4	1.388694	up	7.10E-08
GBE	0.033	0.079	2.065	1.056	0.533	0.779	5.198	1.412	1.063	0.663	1.274087	up	3.86E-12
CYP2D6	199.941	202.347	258.873	189.449	422.847	141.574	63.327	158.998	133.191	118.588	-1.0485	down	2.58E-05
SHISA8	23.212	22.677	3.234	3.106	14.658	28.645	32.788	31.828	31.165	29.079	1.198427	up	4.12E-10
SREBF2	42.254	45.929	5.589	5.938	33.978	53.715	41.729	63.535	61.64	56.63	1.052284	up	1.88E-08
MEI1	1.876	1.816	1.172	1.415	2.148	0.615	0.527	0.723	0.491	0.543	-1.53783	down	7.90E-11
CHADL	1.099	1.125	1.179	1.815	0.233	0.382	0.143	0.553	0.593	0.583	-1.27216	down	5.77E-07
ADSL	312.305	295.773	586.536	605.692	277.771	98.709	136.389	253.666	124.352	178.972	-1.39151	down	2.78E-09
NPTXR	2.066	1.895	0.571	0.682	0.203	13.656	7.08	6.451	8.217	14.681	3.207626	up	4.11E-49
DNAL4	2.011	1.833	1.659	2.273	2.822	9.511	8.447	5.626	5.815	4.765	1.688216	up	1.81E-17
FAM20CL	433.81	459.966	1.954	25.555	0.424	1766.83	1088.799	1044.416	1260.087	1377.028	2.826274	up	1.43E-39
SUN2	18.332	17.316	9.806	6.471	7.075	41.904	35.651	44.456	29.959	34.953	1.663574	up	2.14E-17
KDELR3	0.398	0.521	0.373	0.423	0.529	0.647	6.771	1.085	0.837	0.68	2.156247	up	8.37E-21
LOC10174	0.027	0.053	0.021	0.331	0.11	0.078	1.611	0.771	0.267	0.142	2.393447	up	2.23E-14
CARD10	1.29	1.45	2.944	2.441	0.998	0.476	0.612	0.501	0.541	0.66	-1.70745	down	3.41E-12
MCM5	0.54	0.535	0.614	0.596	0.998	1.953	2.196	1.838	1.839	2.037	1.585547	up	7.45E-15
HMOX1	166.243	202.617	337.571	220.403	993.546	142.566	234.301	188.206	167.534	177.784	-1.07683	down	1.70E-05
TIMP3	39.829	43.096	67.127	36.323	78.951	22.946	8.032	23.36	32.535	39.435	-1.07079	down	2.90E-05
TCP11X2	19.061	15.21	20.763	18.17	16.985	3.182	9.882	8.159	5.731	5.035	-1.49523	down	8.73E-11
HSP90B1	157.01	148.585	216.797	181.815	205.894	946.722	489.385	290.086	1028.581	669.386	1.911646	up	9.54E-23
ASCL1	5.587	5.175	8.032	2.459	3.31	0.822	0.502	1.062	0.499	0.801	-2.7347	down	4.90E-28
PAH	238.822	233.114	374.235	380.978	642.348	115.909	99.674	290.163	198.916	155.344	-1.12023	down	4.51E-06
IGF1	1.396	2.968	5.042	4.753	9.936	0.218	0.373	0.44	0.248	3.094	-2.46069	down	6.73E-20
CGTL	10.907	8.938	16.05	5.069	31.518	31.503	83.284	50.992	6.708	24.429	1.441823	up	1.29E-14
CHRM2	15.188	12.909	22.297	16.437	5.351	2.944	3.612	6.461	2.374	3.983	-1.89724	down	1.54E-16
WNT5B	1.95	1.371	0.924	2.039	1.837	0.469	1.255	0.178	0.496	0.468	-1.50099	down	1.20E-09
LOC42513	2.991	3.243	1.902	1.998	5.493	1.138	0.681	1.003	0.955	1.144	-1.66601	down	3.02E-12
RERGL	5.582	5.401	10.87	7.756	7.569	2.194	1.817	2.641	1.513	1.929	-1.88043	down	2.38E-15
SLCO1B1	1.066	0.722	0.939	0.309	0.386	3.321	4.02	9.351	1.395	5.877	2.806152	up	8.55E-39
LDHB	371.616	323.912	870.705	415.305	520.845	74.726	68.808	69.715	43.43	63.058	-2.96833	down	8.53E-37

GYS2	30.424	29.039	25.668	32.003	48.728	11.73	15.1	22.574	17.997	14.487	-1.01821	down	2.35E-05
SSPN	0.694	0.719	3.161	1.225	0.776	6.655	3.59	7.196	7.605	3.445	2.1146	up	2.00E-27
BHLHE41	1.708	1.624	7.576	3.005	1.425	19.223	9.756	21.05	19.762	9.477	2.369249	up	7.62E-33
MPPED1	1.909	1.919	3.455	3.065	2.405	0.887	0.596	0.897	1.167	1.307	-1.39267	down	1.46E-08
SULT4A1	3.1	3.017	2.947	1.635	1.33	8.728	6.7	10.343	10.798	11.767	2.006131	up	1.15E-23
PNPLA3	156.704	150.92	24.41	32.198	22.297	315.267	234.61	375.033	414.558	521.491	2.267383	up	4.30E-28
SAMM50	16.024	13.413	17.797	17.674	22.152	7.743	7.16	8.727	9.075	9.411	-1.04755	down	2.09E-05
PRR5	1.498	1.569	1.701	1.876	2.152	0.777	1.036	0.971	0.864	0.636	-1.03703	down	3.80E-05
KIAA0930	11.283	11.171	9.327	8.652	9.075	21.578	20.838	16.406	17.228	23.626	1.009511	up	2.72E-08
CREBL2	30.39	29.002	35.281	28.826	29.692	9.985	14.543	13.61	12.397	17.108	-1.17926	down	8.67E-07
VWF	7.41	10.663	8.509	15.154	9.204	2.66	2.195	6	6.786	6.973	-1.04917	down	1.03E-05
PHC1	14.72	13.451	17.715	15.388	16.7	6.919	9.693	6.974	7.578	6.437	-1.05212	down	1.38E-05
A2ML3	9.361	12.466	9.122	12.015	43.861	6.316	6.154	9.576	6.267	6.287	-1.32721	down	2.39E-08
CASR	1.936	1.763	1.204	1.279	0.076	0.293	0.045	0.674	0.976	0.167	-1.53582	down	1.56E-10
CSTA	1.679	0.305	2.748	1.533	0.767	0.95	0.699	0.206	1.019	0.123	-1.22904	down	0.001245
GPR162	0.25	0.278	0.697	0.226	0.295	1.11	0.431	0.883	0.472	1.156	1.212234	up	9.95E-10
GNB3	2.711	2.118	0.809	0.681	1.682	0.383	0.508	0.249	0.243	0.348	-2.20531	down	1.87E-17
CDCA3	0.108	0.236	0.287	0.119	0.371	0.263	0.703	0.717	1.649	0.667	1.830235	up	1.57E-12
RBP5	40.65	39.033	69.173	47.182	67.993	20.339	26.237	18.905	18.406	18.948	-1.36033	down	1.42E-08
EPHA1	0.234	0.347	0.187	0.234	0.209	0.623	2.168	1.236	1.751	1.197	2.521084	up	3.10E-29
GSTK1	25.813	24.89	36.138	24.733	60.593	12.164	10.011	10.564	15.311	11.821	-1.5238	down	1.22E-10
KEL	0.295	0.55	1.339	0.171	0.418	0.139	0.299	0.115	0.155	0.172	-1.6503	down	3.62E-09
STYK1	1.827	1.717	1.142	1.657	0.924	0.177	0.345	0.433	0.605	0.322	-1.94626	down	2.25E-16
LOC10085	0.216	0.094	1.373	0	0.074	0	0	0	0	0	-8.46107	down	1.73E-09
HSD3B1	3.125	2.257	3.378	3.893	2.655	1.219	0.958	2.941	0.689	0.571	-1.26245	down	1.12E-07
HAO2	41.002	37.026	43.088	67.589	79.114	10.9	9.947	30.643	11.048	13.841	-1.80994	down	2.65E-15
SIDT1	0.279	0.324	0.715	0.305	0.456	3.71	0.17	3.757	2.522	0.395	2.341046	up	6.11E-29
MAB21L3	0.579	0.43	0.396	1.773	0.466	1.58	3.153	1.203	1.405	1.052	1.202545	up	1.68E-09
SLC22A15	1.153	1.048	0.659	2.636	0.798	3.108	5.423	2.483	3.051	1.878	1.340181	up	1.43E-11
ZBTB20	3.827	3.739	8.413	3.953	6.59	1.728	2.509	3.224	1.742	1.892	-1.2569	down	2.59E-07
CLDND1	82.942	76.685	23.146	32.862	17.209	234.789	134.213	115.35	158.264	199.934	1.855376	up	2.20E-20
APOV1	9688.97	12281.13	57.327	2791.273	5.816	29748	22979.76	18748.89	20159.39	20455.32	2.174837	up	2.17E-25
CEP97	2.13	1.641	4.852	1.729	1.752	1.115	1.224	1.161	0.898	1.072	-1.14515	down	8.02E-06
LOC41841	17.461	16.654	13.158	18.336	30.201	2.677	3.007	7.749	3.19	4.151	-2.20513	down	1.98E-21
NECTIN3	4.335	3.927	5.972	3.293	3.891	1.09	1.879	2.043	1.781	1.989	-1.28572	down	1.19E-07
CREG1	48.616	45.349	90.265	84.383	124.389	18.102	22.309	24.952	32.169	20.405	-1.73648	down	9.55E-14
MAEL	1.904	1.824	2.163	2.202	3.364	0.562	0.597	0.866	1.151	1.004	-1.45356	down	9.22E-10
BCL9	3.518	3.118	7.03	3.542	2.862	1.407	2.48	1.811	1.3	1.267	-1.27944	down	1.10E-07

ROBO1	0.809	0.627	0.597	0.355	1.754	1.603	1.792	2.65	2.367	0.786	1.150036	up	5.16E-10
ABCC13	12.232	11.302	9.863	13.435	9.035	4.681	5.214	4.371	5.831	5.159	-1.14521	down	1.07E-06
ADAMTS1	0.289	0.327	0.705	0.609	0.702	5.558	4.094	1.061	1.548	2.248	2.460476	up	1.10E-31
MAP3K7C	6.165	6.29	15.608	12.845	8.111	15.553	59.736	50.094	36.091	33.581	1.992358	up	6.73E-24
EVA1C	7.313	8.262	15.975	6.286	11.069	2.275	5.334	3.609	3.082	2.54	-1.53781	down	2.03E-10
GART-B	0.305	0.262	1.808	0.246	0.803	0.278	0.257	0.351	0.259	0.193	-1.35233	down	1.43E-06
SETD4	0.563	0.783	0.785	1.249	2.097	0.184	0.696	0.807	0.615	0.242	-1.10477	down	2.34E-05
CBR3	25.821	26.229	39.048	34.234	40.938	7.947	13.139	14.425	13.008	12.25	-1.45205	down	6.57E-10
IGSF5	26.265	24.783	26.048	20.034	26.62	5.894	13.704	12.941	11.955	9.871	-1.1866	down	5.34E-07
PCP4	19.784	17.63	42.577	28.453	30.131	5.336	12.392	6.397	7.115	9.907	-1.75168	down	3.48E-13
AGPAT3	34.164	32.233	24.234	30.034	25.402	12.04	9.329	16.244	19.981	12.319	-1.06294	down	6.88E-06
MID1IP1	25.443	24.255	10.061	8.648	11.947	88.095	38.895	60.409	28.159	80.976	1.88369	up	3.35E-21
OTC	0.045	0.074	0	0	0	0.264	0.272	0.15	0.203	1.018	3.94667	up	7.83E-25
NR0B1	37.5	36.706	148.366	39.949	35.715	16.378	18.11	19.448	21.789	13.722	-1.73729	down	8.88E-13
SLC51AL	1.037	0.838	1.068	1.955	3.483	0.071	0.073	0	0.17	0.064	-4.45257	down	2.79E-52
MAP3K15	1.168	1.278	2.428	1.267	1.369	8.547	11.423	7.425	7.695	9.771	2.577778	up	2.62E-36
PDHA2	12.365	12.837	21.312	13.524	16.276	79.762	81.851	72.513	74.789	94.179	2.40102	up	1.35E-32
ADGRG2	0.147	0.183	0.172	0.12	0.105	1.937	3.445	1.546	2.48	1.531	3.902153	up	8.89E-62
PHKA2	38.898	36.35	43.953	27.253	34.641	18.764	13.076	17.99	16.664	19.637	-1.0721	down	1.21E-05
PPEF1	1.317	1.221	4.377	0.956	0.985	0.791	0.929	0.719	0.952	0.54	-1.17074	down	1.05E-05
SCML2	0.429	0.393	0.627	0.352	0.456	1.18	1.028	0.619	1.146	0.875	1.101278	up	5.76E-09
GRPR	0.273	0.29	0.054	0.028	0.035	1.341	0.511	0.678	4.374	1.803	3.668828	up	2.42E-39
LOC12110	1.293	1.071	29.826	154.672	238.651	0.23	0.43	0.887	0.428	0.569	-7.38314	down	#######
LOC10705	27.588	27.701	135.478	188.365	451.459	0.472	0.354	0.387	0.592	0.393	-8.55853	down	#######
PRKX	1.764	1.589	1.375	2.309	1.236	4.959	3.493	5.425	7.438	6.048	1.725136	up	2.82E-18
LOC41865	31.573	29.276	14.798	28.114	9.619	3.412	5.508	10.022	6.682	10.833	-1.63676	down	3.29E-13
SLC25A6	931.934	901.092	889.85	906.808	623.308	354.641	315.193	331.68	319.349	456.527	-1.25872	down	7.34E-08
RGN	167.399	184.424	233.925	226.051	288.396	88.596	84.797	94.284	94.975	109.541	-1.2203	down	3.26E-07
LYGL	0.491	0.321	1.074	0.941	0.252	1.011	3.552	2.075	1.056	0.648	1.436455	up	4.52E-10
LYG2	5.389	7.857	3.234	13.497	12.139	0.675	118.183	1.27	1.7	14.144	1.690751	up	2.50E-16
RNF149	9.356	9.945	12.002	7.396	9.082	30.12	6.001	16.452	22.604	23.11	1.040486	up	3.99E-09
IL1R2	1.412	1.015	4.233	3.323	1.28	1.443	17.07	3.535	1.604	1.914	1.182277	up	5.24E-10
IL1R1	19.591	19.643	52.678	44.227	11.897	15.294	192.196	36.115	24.802	28.134	1.002259	up	5.48E-08
IL1RL1	1.863	2.123	4.115	1.225	2.715	7.536	1.44	7.609	7.34	6.298	1.327331	up	2.97E-13
GAS6	3.859	3.339	4.603	2.792	3.884	1.499	2.211	0.61	2.81	1.258	-1.13886	down	5.68E-06
LOC77101	171.429	145.53	349.521	449.305	214.164	78.114	91.448	77.638	74.232	143.14	-1.51739	down	6.98E-11
RAB20	33.363	32.669	54.294	22.269	35.116	10.877	18.445	11.217	10.018	10.884	-1.53218	down	9.60E-11
LOC12110	1.523	2.514	0.799	7.567	0.711	3.173	4.408	11.99	7.771	8.591	1.453852	up	3.98E-12

GPR183	1.298	1.255	2.634	1.577	2.759	0.824	0.671	0.867	0.844	0.676	-1.29352	down	3.72E-07
DOCK9	5.304	5.281	7.746	4.802	5.196	1.275	1.686	1.71	3.206	2.573	-1.43834	down	1.56E-09
HS6ST3	1.605	1.497	1.513	1.551	0.979	0.182	0.066	0.277	0.262	0.406	-2.57732	down	6.47E-26
DZIP1	1.114	0.845	1.35	1.568	0.73	0.241	0.426	0.497	0.459	0.579	-1.34643	down	1.51E-07
DCT	2.22	2.581	0.314	0.343	0.081	0.016	0.068	0.174	0.045	0.015	-4.10132	down	1.37E-45
CLN5	20.479	20.833	12.296	4.602	13.526	7.569	3.686	6.617	6.47	8.661	-1.11998	down	3.83E-06
ACOD1	1.389	1.414	1.721	1.603	0.649	0.98	8.661	0.588	3.833	2.001	1.24462	up	2.88E-10
CPB2	157.071	155.027	189.176	180.56	178.72	62.815	96.127	74.69	66.801	76.997	-1.18905	down	4.81E-07
WDFY2	23.291	23.107	18.114	26.044	29.474	9.659	10.164	11.178	9.379	10.488	-1.23848	down	1.17E-07
SLC25A15	0.622	0.779	3.087	0.835	2.95	5.561	2.11	3.71	0.922	4.527	1.02411	up	1.98E-09
UFM1	41.632	32.28	35.356	36.185	37.831	85.224	81.039	45.721	103.973	69.339	1.071866	up	4.50E-09
POSTN	0.715	0.878	4.066	3.799	2.149	0.338	0.362	1.105	0.302	0.26	-2.29144	down	4.52E-21
LOC1211(	0.429	0.25	1.373	0.466	0.715	0.235	0.311	0.163	0.243	0.17	-1.52262	down	6.44E-09
N4BP2L1	95.971	85.03	142.452	114.608	74.546	27.338	31.936	28.326	37.117	25.045	-1.77515	down	1.38E-14
FLT1	1.535	2.033	1.541	2.408	2.083	0.568	0.293	1.047	0.665	0.7	-1.55097	down	1.09E-10
SGCG	15.113	13.654	3.805	0.356	1.199	1.649	0.033	0.025	0.033	0.293	-4.0659	down	3.01E-60
GJB2	0.427	0.523	0.025	0.386	1.49	1.226	6.745	1.53	0.932	1.141	2.019443	up	2.50E-21
ACAT1	280.209	264.862	449.211	387.065	396.999	114.943	90.006	122.844	144.627	151.408	-1.51131	down	1.35E-10
CCDC82	2.427	2.113	3.038	2.11	1.63	0.571	0.966	1.029	0.59	0.624	-1.58089	down	1.24E-11
MTMR2	4.154	3.765	3.765	4.976	4.601	2.069	2.284	1.984	1.991	2.239	-1.0083	down	3.41E-05
ENDOD1	0.612	0.607	0.903	0.924	1.226	0.129	0.177	0.348	0.186	0.13	-2.13313	down	4.24E-16
TAF1D	25.24	23.33	26.332	31.761	22.81	11.504	12.835	10.12	9.386	12.344	-1.20422	down	2.95E-07
CEP295	6.341	5.798	9.992	10.59	7.067	4.84	3.444	2.56	2.496	4.035	-1.19509	down	6.52E-07
LOC1211(	9.46	8.531	58.342	69.061	164.156	9.853	7.41	10.309	15.466	16.152	-2.38665	down	2.65E-24
LOC1211(	3.787	3.934	19.381	27.141	67.661	5.354	3.765	9.385	10.109	10.615	-1.63567	down	9.58E-12
LOC1017	0.023	0.03	0	0	0	0.3	1.132	0.404	0.523	0.242	5.48964	up	3.03E-38
LOC10174	22.551	17.406	62.447	35.295	34.441	92.787	61.243	126.526	126.929	79.333	1.499773	up	5.08E-16
THRSPB	67.486	71.497	246.224	149.037	160.855	1250.897	593.496	605.933	663.949	913.488	2.53468	up	1.93E-36
THRSP	672.36	706.424	1527.01	988.249	1502.24	3627.776	2545.859	2768.304	2046.923	3670.442	1.441778	up	5.40E-15
CAPN5	2.419	2.309	4.736	2.929	5.462	0.76	0.579	0.995	0.739	0.954	-2.14716	down	2.09E-19
TSKU	1.372	1.845	1.784	5.612	10.349	0.377	0.639	3.262	1.484	0.342	-1.77911	down	6.27E-14
WNT11	0.476	0.429	2.908	1.358	1.013	1.907	0.831	4.77	5.38	1.863	1.253523	up	1.65E-12
ART1L2	0.708	0.716	0.231	1.773	3.341	0.187	0.338	1.705	0.64	0.468	-1.01886	down	0.000183
IL18BP	0.253	0.332	0.323	0.75	0.573	1.18	0.835	1.677	0.655	0.669	1.167055	up	9.43E-06
PDE2A	1.409	1.431	4.124	1.677	1.598	1.016	0.976	0.836	0.773	1.132	-1.11243	down	2.18E-05
WDR73	14.715	15.02	14.284	14.094	12.586	3.366	9.695	3.692	2.566	6.074	-1.47708	down	1.73E-10
ADAM15	2.879	2.467	2.28	10.016	2.523	0.182	0.281	0.242	0.093	0.764	-3.68613	down	9.43E-48
SLCO2B1	22.211	22.886	18.128	38.38	42.452	9.848	12.95	14.469	7.215	9.807	-1.40783	down	8.43E-10

POLD3	5.774	6.135	5.492	5.728	4.171	2.084	2.5	3.128	2.024	2.476	-1.16028	down	1.24E-06
HBE	0	0.145	18.385	0	0.152	0	0	0	0.074	0	-7.88597	down	8.44E-47
HBBA	251.682	258.768	745.477	87.668	273.636	42.104	49.13	42.56	53.267	32.597	-2.88017	down	6.16E-34
HBE1	46.694	46.334	122.225	11.314	34.039	4.44	4.997	6.576	6.062	5.152	-3.25852	down	2.29E-40
TAF10	33.266	32.073	29.405	36.015	40.872	16.212	21.779	9.95	9.855	12.467	-1.28841	down	3.90E-08
TPP1	197.54	210.865	179.416	267.845	237.865	61.586	70.843	50.609	52.19	79.108	-1.7986	down	2.34E-15
GIMAP1-(	7.613	7.95	5.13	5.913	2.9	1.461	2.319	2.584	1.87	1.325	-1.62557	down	2.95E-12
ELP6	3.722	3.527	2.005	2.384	1.855	6.252	7.831	7.185	6.28	5.383	1.28692	up	7.77E-11
SCAP	12.353	12.424	5.509	6.9	7	26.141	26.447	26.828	29.2	29.763	1.646853	up	5.64E-17
ACKR2	1.96	1.81	4.688	2.679	3.108	16.238	30.462	16.586	15.055	12.946	2.679527	up	1.12E-38
CYP8B1	3.027	2.459	7.577	4.087	5.467	30.86	58.114	31.524	28.451	24.463	2.938447	up	7.31E-45
CHDSD	1.816	1.503	2.053	1.127	7.993	0.495	0.843	0.776	0.454	1.686	-1.76717	down	9.73E-13
CATH3	0.349	0.513	1.804	1.719	0.09	0.254	0.261	0.385	0.087	0.115	-2.01684	down	1.79E-06
CATH2	0.158	0.155	1.513	4.098	0	0	0	0.524	0	0.105	-3.22523	down	2.72E-15
CATH1	0.102	0.201	1.342	3.694	0.21	0.297	0.077	0.902	0.203	0.067	-1.84033	down	2.28E-09
CRHR2	16.495	14.321	2.114	7.903	10.444	1.089	3.343	1.62	1.445	1.883	-2.45002	down	2.08E-27
DLEC1	3.841	3.28	5.44	4.057	2.006	0.769	0.657	0.37	0.64	0.681	-2.57701	down	5.57E-28
ACAA1	153.55	145.844	192.261	192.372	200.895	37.707	22	25.904	32.852	31.939	-2.55669	down	1.10E-28
SLC22A13	34.412	31.009	18.528	18.896	43.051	12.083	14.26	11.019	14.824	12.359	-1.1765	down	6.16E-07
CSRNP1	2.287	1.889	3.407	2.989	2.69	10.908	3.242	3.601	6.177	4.177	1.083241	up	1.38E-09
RHEB	72.887	56.797	74.462	57.61	56.586	30.01	34.434	29.178	24.751	31.493	-1.08688	down	6.47E-06
IDI2	138.152	139.362	14.966	10.862	128.774	183.315	152.909	331.1	322.405	213.49	1.477398	up	2.50E-14
KLF6	3.151	3.422	10.461	4.934	7.704	10.567	7.258	14.558	20.331	10.215	1.084494	up	4.49E-10
MAP3K8	0.832	0.711	0.888	1.681	0.814	1.054	7.931	1.231	1.322	1.176	1.367033	up	1.67E-11
APBB1IP	1	1.071	3.128	1.33	4.054	2.752	4.632	4.935	5.13	4.106	1.025927	up	6.93E-09
NELL3	0.006	0	0.06	0.01	0.184	0.303	2.21	0.6	0.851	0.741	4.151663	up	3.72E-62
OTUD1	16.248	16.067	19.749	7.161	12.621	4.611	6.491	5.743	7.242	7.427	-1.18879	down	1.12E-06
RSU1	2.554	2.116	3.427	2.316	2.365	6.665	7.578	9.106	6.062	6.362	1.484846	up	7.92E-15
NMT2	24.091	21.284	18.727	21.706	23.506	7.963	6.64	10.41	10.706	8.16	-1.31678	down	1.81E-08
ACBD7	1.497	1.217	3.527	1.728	6.917	0.647	1.091	1.16	0.944	0.561	-1.75624	down	5.69E-12
OLAH	0.248	0.282	1.006	0.256	1.495	0.116	0.252	0.159	0.151	0.148	-1.98604	down	3.31E-13
CROT	68.879	62.909	58.579	57.833	59.509	19.772	33.334	18.552	21.874	25.779	-1.3668	down	3.17E-09
ABCB1LA	27.561	25.71	20.052	17.421	19.97	2.416	2.113	3.07	2.068	3.04	-3.12264	down	3.18E-41
CYP51A1	48.663	52.847	8.028	7.111	62.297	126.564	68.059	162.03	187.807	146.96	1.950008	up	1.06E-22
CDK6	8.803	8.955	11.844	8.675	7.442	40.749	23.458	29.564	34.021	27.011	1.759457	up	8.60E-20
TFPI2	7.336	7.157	6.972	5.455	5.069	2.097	1.734	3.131	1.562	1.989	-1.60494	down	1.35E-11
PDK4	72.111	69.423	92.979	153.728	64.769	33.434	38.561	10.515	11.364	11.852	-2.09916	down	9.58E-21
AHR1A	15.052	13.985	18.876	11.902	18.648	3.252	8.31	7.865	5.483	4.282	-1.42628	down	9.64E-10

HDAC9	0.261	0.154	0.35	0.245	0.251	0.982	0.508	0.785	1.189	0.732	1.730455	up	1.37E-18
WIPF3	1.121	1.131	1.22	0.407	0.626	1.584	4.428	2.252	1.53	1.279	1.296498	up	8.39E-12
FKBP14	2.634	2.28	3.848	1.517	2.599	5.975	7.613	6.269	7.478	5.421	1.346511	up	8.65E-13
SATB1	0.905	0.762	1.234	1.188	0.641	3.556	4.324	2.685	2.557	3.062	1.773578	up	2.44E-19
LRRC3B	2.343	2.84	6.435	2.755	3.355	1.845	1.03	1.565	3.245	1.135	-1.00677	down	0.000248
CMTM8	0.437	0.571	0.522	0.61	0.359	0.127	4.514	0	0.954	0.058	1.176058	up	3.38E-06
SNRK	8.746	8.68	13.306	8.944	10.578	4.67	4.639	5.431	4.694	4.777	-1.05342	down	2.09E-05
ACAD11	94.158	91.985	133.79	127.816	125.149	34.284	29.19	36.247	40.134	39.013	-1.67936	down	4.40E-13
ACKR4	6.965	6.728	9.821	10.779	6.816	2.733	3.845	4.144	4.042	2.087	-1.28637	down	5.76E-08
MOBP	0.408	0.31	0.237	0.469	0.393	1.509	0.126	0.543	0.483	2.687	1.554825	up	7.62E-15
AMPH	0.024	0.056	1.094	0.01	2.771	0.035	0.018	0.054	0.121	0.128	-3.45543	down	3.23E-28
LOC12110	0.547	0.596	2.177	0.599	0.468	0.332	0.137	0.402	0.498	0.18	-1.49889	down	2.79E-05
INHBA	4.865	4.491	2.44	9.618	6.196	22.2	20.561	38.414	31.687	19.754	2.263785	up	3.11E-28
EGFR	58.5	50.992	62.672	47.766	47.527	19.507	16.664	23.862	16.552	14.999	-1.54609	down	2.44E-11
PDIA4	95.545	97.275	183.461	161.217	159.512	381.549	234.363	179.207	450.653	304.108	1.1529	up	1.41E-10
RAMP3	4.135	3.957	5.695	4.89	4.29	2.195	1.8	3.236	1.873	1.696	-1.08818	down	1.10E-05
IGFBP1	40.494	39.916	40.039	34.635	17.005	4.736	4.805	3.07	3.069	2.958	-3.20649	down	4.79E-43
KCNG2	0.662	0.506	0.242	0.203	0.111	1.162	0.152	1.545	1.332	1.753	1.782709	up	6.85E-19
SALL3	0.028	0.029	0.303	0.176	0.231	0.655	1.4	0.573	0.66	0.484	2.290568	up	2.55E-28
DCDC2	1.066	0.95	2.24	1.084	1.124	1.827	9.092	3.313	6.397	5.066	1.990154	up	7.36E-24
NRSN1	2.815	2.74	1.361	1.638	0.533	2.974	6.4	4.435	8.636	13.668	1.990048	up	2.06E-22
EDN1	1.594	1.199	0.428	0.802	0.45	0.029	0.269	0.308	0.732	0.131	-1.60312	down	4.64E-09
ELOVL2	182.236	179.616	5.847	23.01	45.086	281.426	198.972	285.006	346.787	328.72	1.725248	up	1.01E-17
SYCP2L	87.486	81.32	3.059	10.072	17.064	148.524	166.782	133.964	154.098	146.111	1.913086	up	7.50E-21
GCNT2	1.552	1.459	0.351	0.81	1.005	2.421	3.089	7.464	6.11	2.27	2.043263	up	3.06E-23
TXNDC5	2.63	2.896	5.819	4.605	3.285	8.634	8.256	3.994	10.79	7.711	1.033721	up	7.42E-09
F13A1	0.857	0.865	0.604	1.348	0.503	0.267	0.495	0.297	0.511	0.291	-1.16424	down	6.16E-06
PPP1R3G	6.647	6.35	8.518	6.672	18.704	3.667	2.32	2.483	2.417	2.934	-1.76208	down	8.78E-14
ECI2	248.06	223.024	457.551	325.633	209.669	35.524	47.238	29.506	41.847	43.671	-2.88781	down	1.31E-35
IRF4	0.969	0.337	1.172	0.62	0.602	0.244	0.574	0.475	0.297	0.237	-1.01605	down	0.000276
SERPINB10	0.068	0.607	0.524	2.123	0.433	0.147	0.101	0.596	0.369	0.423	-1.19616	down	6.50E-06
FAM134B	17.834	16.626	17.402	21.612	27.89	3.744	14.618	10.772	7.463	7.806	-1.19073	down	3.21E-07
ANKRD33	0.689	0.86	0.748	1.736	0.884	1.458	1.313	3.177	2.347	2.397	1.11989	up	1.55E-08
CMBL	109.934	119.002	147.194	84.218	175.479	7.76	16.171	12.332	18.124	7.591	-3.3587	down	1.56E-46
SRD5A1	3.159	2.519	9.173	6.425	4.217	1.78	1.625	1.079	1.701	1.011	-1.82411	down	2.73E-14
UPP1	0.126	0.165	1.374	0.38	0.605	0.092	0.221	0.418	0.251	0.139	-1.23751	down	0.00024
LOC42850	0.078	0.638	0.187	0.064	0.361	1.363	0.643	0.258	0.62	1.237	1.630067	up	8.12E-10
MOCOS	33.131	32.776	10.736	22.622	11.783	84.499	49.994	69.201	75.174	84.16	1.708852	up	7.69E-18

SEC61B	63.379	70.841	78.921	65.259	55.47	208.757	162.321	95.617	183.93	132.453	1.229857	up	3.26E-11
IFI6	18.124	21.104	45.414	28.49	5.721	155.445	62.773	101.063	53.014	22.718	1.732679	up	2.68E-19
GLIPR2	0.355	0.398	0.878	0.698	0.456	2.305	1.614	1.118	1.699	1.455	1.554655	up	9.06E-15
MBP	2.256	2.727	2.505	2.876	2.993	1.201	1.075	1.832	1.279	1.233	-1.01214	down	3.36E-05
ZNF516	0.444	0.453	0.787	0.547	0.696	1.161	1.026	1.314	1.121	1.588	1.083869	up	2.29E-09
C18orf63	0.005	0.011	0.022	0.094	0.028	0.238	0.212	1.251	0.233	0.273	3.744813	up	3.13E-44
FBXO15	0.747	1.086	0.582	1.092	1.945	0.193	0.174	0.366	0.297	0.767	-1.59851	down	2.42E-09
LDLRAD4	4	4.112	6.699	4.179	11.312	2.763	2.507	3.067	2.142	3.454	-1.12063	down	7.06E-06
PSMG2	13.81	11.588	11.217	9.959	23.015	2.562	8.224	4.799	3.909	3.327	-1.60828	down	6.66E-12
CIDEA	199.64	214.587	173.713	209.271	309.342	61.684	39.395	70.373	70.861	76.201	-1.79663	down	5.62E-15
VAPA	21.73	20.852	21.143	22.633	16.869	6.862	10.39	12.334	9.375	8.911	-1.10849	down	2.59E-06
TGIF1	12.25	10.563	11.097	3.061	3.781	1.692	3.741	3.861	3.519	2.564	-1.4058	down	2.29E-09
MYOM1	1.006	0.83	1.409	0.757	2.832	0.236	0.825	0.498	0.126	0.274	-1.79999	down	5.50E-14
LPIN2	29.822	30.149	58.891	30.487	110.317	8.027	14.06	19.408	9.093	10.085	-2.09744	down	1.92E-19
LOC10705	1.786	1.615	0.971	0.692	1.461	0.232	0.196	0.769	0.49	0.498	-1.57615	down	6.32E-10
ABHD3	12.343	10.311	30.543	4.203	14.329	1.897	2.88	1.687	2.166	1.395	-2.83834	down	8.64E-33
CEBPD	43.782	44.448	65.219	31.349	55.313	24.669	36.007	18.54	16.962	20.603	-1.03986	down	2.56E-05
MCM4	0.945	0.866	1.037	0.742	1.931	1.666	2.518	1.831	2.796	2.829	1.075403	up	5.65E-09
C8orf22	2183.87	2049.309	595.971	1564.483	746.212	86.735	185.207	272.821	186.138	277.216	-2.82422	down	4.66E-36
PCMTD1	28.718	25.198	38.701	31.912	30.82	8.658	16.565	16.49	11.532	8.993	-1.31958	down	1.84E-08
FAM110B	2.094	2.421	2.344	2.525	3.412	1.101	0.677	1.588	1.072	0.767	-1.2969	down	8.82E-08
CYP7A1	5.926	9.393	3.053	30.473	24.168	74.403	31.367	126.704	113.35	123.243	2.683485	up	1.72E-37
TTPA	212.277	187.82	400.848	222.359	240.997	80.535	150.304	139.805	85.404	104.501	-1.17342	down	1.39E-06
CYP7B1	83.541	77.824	80.029	79.809	69.066	39.26	27.48	44.425	48.441	34.516	-1.00749	down	3.07E-05
MTFR1	103.881	100.861	62.933	67.583	61.468	36.276	28.852	33.473	35.517	31.501	-1.26025	down	4.76E-08
ADHFE1	30.126	28.277	38.093	46.478	60.293	16.851	13.91	16.672	17.681	24.948	-1.17434	down	1.11E-06
SULF1	0.055	0.016	0.034	0.04	0.006	0.124	0.082	0.175	0.953	3.88	5.064155	up	2.72E-78
XKR9	0.272	0.235	0.097	0.395	0.345	0.649	1.155	0.575	0.622	0.558	1.401607	up	1.66E-11
TRPA1	1.316	1.061	0.084	2.772	4.407	0	0.458	0.415	0.315	0	-3.01519	down	3.16E-33
C2H8ORF8	1.46	1.54	1.494	1.885	1.56	0.347	1.116	0.526	0.858	0.59	-1.20662	down	9.52E-06
PKIA	1.626	1.396	0.728	0.167	0.522	1.183	5.025	1.57	2.221	0.403	1.227624	up	2.22E-06
ZC2HC1A	0.892	0.837	1.093	0.79	0.957	4.119	2.784	1.941	4.135	2.774	1.784556	up	7.71E-20
HEY1	1.193	1.244	2.104	1.338	1.043	0.633	1.111	0.64	0.384	0.255	-1.19387	down	5.12E-06
FABP5	9.668	10.196	10.394	16.943	14.285	1.997	5.391	3.768	4.271	3.543	-1.69628	down	2.71E-13
FABP4	0.671	0.603	1.803	3.858	0.861	0.122	0.251	0.37	0.167	0.111	-2.92663	down	1.86E-14
CA13	2.575	2.243	4.942	1.926	2.63	0.631	1.799	1.149	1.868	0.497	-1.26741	down	5.71E-07
ATP6V0D2	0.331	0.185	0.978	0.233	0	0.756	3.221	0.625	4.294	1.591	2.598779	up	1.40E-30
WWP1	17.708	16.534	26.756	14.9	10.589	31.087	57.379	47.674	45.778	37.265	1.34153	up	5.96E-13

SLC7A7	0.798	0.679	5.493	2.529	0.477	0.612	2.399	0.796	0.445	0.592	-1.0415	down	7.03E-05
DECR1	72.529	74.177	81.753	108.895	106.914	47.28	28.413	43.641	35.086	45.752	-1.15017	down	1.42E-06
NDUFAF6	2.735	2.142	3.444	3.039	3.739	1.331	2.035	0.956	1.349	1.068	-1.16325	down	2.15E-06
PTDSS1	16.268	15.45	18.831	8.89	8.963	47.81	76.374	35.723	64.34	53.467	2.02141	up	1.87E-24
CPQ	22.699	22.404	10.172	11.779	18.934	82.08	46.018	83.059	75.664	58.795	2.0069	up	1.31E-23
NCALD	15.006	14.897	22.578	15.2	31.005	2.475	2.875	5.101	5.589	1.9	-2.45934	down	7.28E-25
FZD6	1.898	1.767	1.607	2.416	1.571	2.508	7.497	3.904	3.167	2.81	1.102409	up	9.19E-09
RIMS2	0.112	0.099	0.68	0.433	0.142	0.49	1.163	0.647	0.788	0.495	1.286383	up	1.07E-11
DCSTAMP	4.222	3.077	3.015	0.466	0.332	0.259	0.88	0.859	0.223	0.331	-2.12024	down	1.39E-19
EIF3E	85.086	78.72	76.534	76.216	55.66	21.184	28.354	38.662	30.822	35.413	-1.26911	down	5.37E-08
EBAG9	42.514	43.319	23.402	20.343	17.986	72.562	56.209	43.898	69.488	81.476	1.132991	up	1.31E-09
SYBU	0.732	0.648	0.466	0.86	0.411	1.39	0.92	1.304	1.323	1.948	1.142033	up	1.67E-09
ENPP2	7.94	8.56	13.287	15.452	12.036	5.544	3.219	6.08	5.411	5.715	-1.14096	down	2.50E-06
FBXO32	4.269	4.22	6.173	4.274	4.779	1.164	2.832	2.683	2.475	1.823	-1.11096	down	4.94E-06
ANXA13	0.635	0.453	0.643	0.783	0.652	1.176	1.643	0.637	3.669	1.486	1.44208	up	1.01E-11
FER1L6	0.202	0.302	0.074	0.204	0.107	0.168	0.51	0.751	1.124	0.411	1.73163	up	1.72E-15
SQLE	22.329	24.556	0.467	0.583	24.186	58.9	25.412	84.509	95.225	74.915	2.232549	up	6.25E-28
NDRG1	187.047	174.209	179.567	230.082	172.537	52.907	108.617	110.977	79.856	60.099	-1.19368	down	2.44E-07
ST3GAL1	0.976	1.181	2.165	1.029	1.286	4.147	1.3	3.441	2.541	3.347	1.154054	up	1.50E-10
LOC42838	0.05	0.033	0	0.041	0	0.29	0.224	1.318	0.544	0.197	4.320809	up	8.53E-18
RHPN1	0.708	0.818	1.538	0.937	1.039	0.107	0.234	0.223	0.128	0.121	-2.62468	down	1.76E-23
TSTA3	4.979	5.299	4.769	6.335	5.727	16.96	18.115	11.288	15.292	17.113	1.538663	up	2.53E-15
LOC12111	3.227	3.889	5.916	2.652	5.582	3.583	0.254	1.686	0.801	3.475	-1.11745	down	5.33E-05
PRORSD1I	6.63	6.325	5.499	4.573	6.769	14.413	14.662	8.358	12.974	12.816	1.085203	up	5.63E-09
VRK2	4.257	4.074	12.523	14.009	10.787	3.788	3.663	2.367	2.167	2.113	-1.69477	down	8.20E-13
FANCL	3.15	2.907	15.648	18.086	12.899	2.307	2.375	1.795	1.678	1.267	-2.4828	down	1.77E-25
KIAA1841	0.506	0.395	1.208	0.72	0.837	1.527	1.707	1.099	1.874	1.584	1.086561	up	2.21E-09
TPCN3	2.131	2.126	3.603	1.762	2.567	1.416	1.242	0.9	0.915	0.712	-1.23237	down	1.29E-06
BUB1	4.253	4.887	8.572	9.029	2.457	26.804	15.305	39.741	32.061	23.476	2.23411	up	1.23E-28
LOC42123	1.271	1.004	1.146	1.936	0.635	0.656	0.531	0.156	0.392	0.368	-1.50837	down	1.99E-10
CAPN13	0.264	0.107	0.381	1.645	0.336	0.147	0.019	0.111	0.063	0.066	-2.73591	down	3.96E-20
EPCAM	93.8	87.003	56.492	75.803	53.6	34.794	38.792	39.931	35.482	33.73	-1.00487	down	1.82E-05
PPP1R21	12.373	10.366	18.607	13.276	13.017	4.267	7.638	6.333	5.342	4.87	-1.24928	down	1.78E-07
EHBP1	19.949	18.589	22.644	19.693	24.768	4.36	16.312	10.205	9.623	7.89	-1.12634	down	1.89E-06
UGP2	111.949	111.93	146.823	110.221	109.185	456.006	485.414	492.32	670.636	611.511	2.202363	up	3.10E-28
LGALSL	9.208	9.694	5.269	8.972	11.029	2.908	3.944	2.433	3.628	2.256	-1.54169	down	1.90E-11
LOC12111	5.27	4.914	2.967	4.306	7.809	2.115	2.133	1.403	2.678	1.559	-1.353	down	3.74E-08
C1D	6.996	5.996	6.685	4.14	6.324	1.832	2.725	2.315	3.336	3.186	-1.16984	down	3.76E-06

RRBP1	20.908	22.272	26.808	27.12	8.192	38.156	57.325	38.05	38.248	51.178	1.082224 up	4.18E-09
CAPN2	0.891	1.053	1.373	1.57	0.767	0.167	1.141	0.666	0.428	0.36	-1.03222 down	5.85E-05
TLR5	1.701	1.462	1.014	2.244	0.513	0.184	0.649	0.268	0.254	0.186	-2.16619 down	5.25E-22
LPGAT1	63.84	62.964	73.877	58.585	71.183	17.364	44.714	36.379	22.573	26.277	-1.16557 down	7.55E-07
PKDCCA	2.933	2.845	3.748	2.575	1.91	7.223	3.665	3.819	5.598	8.101	1.019375 up	1.47E-08
SLC3A1	16.011	13.798	12.194	19.325	17.52	6.376	7.746	10.437	4.533	7.766	-1.09699 down	3.33E-06
RBKS	28.163	25.999	35.634	21.235	24.175	10.866	16.163	13.808	11.327	9.529	-1.13192 down	2.70E-06
LOC42141	15.524	14.791	15.424	24.637	22.95	54.448	24.185	42.38	55.062	40.274	1.212966 up	6.05E-11
KCNK5	4.649	5.259	11.513	6.235	8.324	15.99	7.78	14.237	21.363	16.994	1.085591 up	5.47E-10
LOC42144	0.941	0.879	0.961	1.47	1.036	0.54	0.686	0.492	0.4	0.507	-1.00875 down	4.98E-05
RMDN2	24.209	22.274	32.058	23.218	23.641	11.977	11.176	11.038	12.504	9.872	-1.14843 down	2.00E-06
TFB2M	8.136	7.106	11.662	6.718	12.565	4.222	5.845	2.682	3.349	5.021	-1.12876 down	6.00E-06
SMYD3	1.008	1.405	1.052	0.624	0.424	0.409	0.643	0.583	0.213	0.371	-1.02253 down	0.000134
LOC42150	4.702	4.941	6.705	1.748	5.821	1.756	1.071	3.589	1.793	3.457	-1.03541 down	6.08E-05
NID1	18.275	18.658	17.282	10.093	19.969	44.758	46.851	33.327	45.061	32.037	1.261337 up	1.10E-11
SLC35F3	0.254	0.206	0.501	0.338	1.305	0.088	0.163	0.027	0.348	0.256	-1.55649 down	2.47E-07
ACTA1	0.222	0.116	0.195	0.146	0.206	1.035	0.433	0.343	0.353	0.176	1.397711 up	3.20E-08
C1orf131	2.91	3.293	3.071	1.971	2.484	5.08	6.491	4.068	8.329	7.451	1.194115 up	1.17E-10
GNPAT	11.337	11.076	5.99	4.546	8.803	17.159	18.122	14.953	28.422	24.656	1.306988 up	5.12E-12
THBS2	0.071	0.079	0.148	0.158	1.474	0.332	0.043	0.173	0.206	0.047	-1.26348 down	1.62E-05
SULT6B1L	792.155	764.842	742.008	734.089	989.757	358.656	346.605	408.192	441.462	273.983	-1.13724 down	1.66E-06
SULT	1527.22	1357.682	2707.046	1942.924	485.372	790.145	348.621	491.157	351.757	1486.811	-1.20934 down	7.95E-07
DACT2	0.361	0.382	0.406	0.794	2.125	0.093	0.175	0.117	0.084	0.126	-2.76306 down	2.50E-23
MPC1L	355.379	296.186	600.226	259.974	295.607	84.903	48.723	55.171	75.961	114.843	-2.25132 down	5.46E-22
LOC42158	1.671	1.488	1.635	0.86	0.951	1.611	0.271	0.577	0.82	0	-1.0092 down	0.000274
FNDC1	2.801	2.213	0.846	1.502	0.292	0.138	0.076	0.279	0.22	0.058	-3.30303 down	1.41E-40
LRP11	0.028	0	0.033	0.068	0.085	0.081	2.286	0.826	0.041	0.11	3.934728 up	4.40E-39
ESR1	11.763	14.742	12.469	13.01	22.164	6.28	4.12	9.943	6.297	6.63	-1.15607 down	1.70E-06
IL22RA2	2.385	1.611	1.09	8.393	9.165	1.479	0.952	0.798	3.126	0.839	-1.65358 down	1.07E-12
IL20RA	73.231	65.395	52.569	84.321	45.451	29.849	17.19	21.405	30.012	26.798	-1.35753 down	3.65E-09
MAP3K5	3.516	2.933	4.614	2.564	1.944	7.11	9.652	5.307	9.014	9.16	1.369592 up	2.71E-13
SGK1	10.604	9.899	24.114	13.805	25.396	67.672	68.656	66.318	74.922	46.598	1.951337 up	8.40E-24
SLC2A12	0.173	0.198	0.189	0.284	0.344	1.57	1.035	2.357	2.648	1.51	2.935231 up	1.68E-38
LOC10705	32.969	32.892	24.991	42.267	11.618	17.864	327.958	56.538	35.008	44.294	1.734552 up	1.88E-17
ARHGAP1	0.751	0.758	3.643	1.347	2.211	0.516	0.673	0.887	0.562	0.6	-1.42617 down	2.00E-08
GJA1	19.079	19.274	11.943	15.972	6.838	39.526	79.863	62.182	66.731	55.155	2.053355 up	5.51E-24
MCM9	1.662	1.497	0.398	0.382	0.229	0.26	0.321	0.197	0.293	0.295	-1.60586 down	7.50E-11
DCBLD1	3.256	3.62	2.803	2.341	1.677	21.21	5.341	8.825	15.605	8.885	2.127471 up	3.62E-26

LOC42174	143.135	116.205	363.465	245.924	407.447	30.296	61.067	70.502	40.007	45.786	-2.36538	down	2.18E-24
SLC16A10	16.435	16.164	23.918	18.022	32.807	6.453	8.557	18.224	12.4	5.908	-1.05838	down	1.62E-05
DDO	1.907	2.007	1.419	2.226	1.731	8.002	3.188	3.658	2.772	7.442	1.431263	up	1.64E-13
METTL24	0.877	1.038	0.1	1.441	0.622	1.276	1.501	2.671	2.032	1.515	1.140295	up	9.21E-08
SESN1	64.62	59.962	64.097	50.996	49.991	24.073	35.389	30.397	21.692	24.586	-1.0893	down	4.46E-06
LOC10705	0.945	1.029	0.556	0.385	0.835	0.373	0.304	0.253	0.474	0.292	-1.14243	down	1.23E-05
LOC12111	0.106	0.208	0.201	0.764	0.204	0.077	0.437	1.23	1.859	0.911	1.602629	up	4.08E-13
GPR63	0.386	0.402	1.207	0.161	1.275	0.276	0.313	0.433	0.389	0.198	-1.09009	down	0.00012
ME1	8.539	9.374	9.18	7.501	16.873	79.319	38.784	39.469	69.798	133.493	2.809611	up	6.69E-42
RWDD2A	3.585	3.795	3.749	2.782	5.311	20.834	14.662	13.325	20.129	30.237	2.36709	up	1.10E-31
SH3BGRL2	33.767	31.02	42.679	24.48	19.65	8.743	4.01	16.305	7.24	8.479	-1.75929	down	3.68E-14
BAG2	0.505	0.393	0.553	0.415	1.428	0.368	0.473	0.348	0.251	0.125	-1.07126	down	0.001594
HMGCLL1	3.515	3.311	3.414	2.888	1.877	0.886	2.247	1.241	1.373	1.392	-1.07112	down	2.04E-05
TINAG	7.763	6.937	7.455	11.514	4.473	2.204	2.08	5.755	3.385	1.888	-1.31644	down	1.89E-08
GCLC	22.984	19.994	29.604	15.821	16.098	11.828	8.373	9.82	9.171	11.242	-1.05097	down	2.33E-05
GSTA3	16.969	18.236	59.154	35.31	163.226	24.627	21.827	20.499	28.336	23.15	-1.3062	down	1.69E-07
ANGPT2	1.215	1.389	0.84	1.271	1.399	0.304	0.57	0.975	0.573	0.551	-1.03895	down	4.05E-05
FAM110C	2.976	2.726	2.712	4.42	5.076	1.168	3.184	0.886	1.176	1.581	-1.1631	down	7.68E-07
MBOAT2	54.307	53.068	2.92	4.222	2.508	68.713	48.774	92.363	93.585	86.045	1.734689	up	9.63E-18
ASAP2	1.022	1.398	2.847	2.443	3.496	0.685	1.411	1.2	1.265	0.815	-1.05897	down	2.75E-05
KLF11	6.606	6.064	5.977	3.564	2.631	1.125	2.963	2.014	1.209	1.122	-1.5581	down	2.07E-11
RRM2	0.136	0.204	0.619	0.895	0.879	1.047	1.427	1.63	2.028	2.079	1.585314	up	2.90E-14
ATP6V1C2	0.951	0.739	1.831	1.71	2.036	3.98	4.484	1.705	4.705	3.14	1.309095	up	8.14E-12
PDIA6	57.434	55.471	70.146	63.546	69.986	175.734	107.89	77.796	234.711	140.44	1.218228	up	2.94E-11
LPIN1	17.605	17.842	32.539	2.685	42.513	5.935	6.76	14.02	13.751	8.374	-1.21245	down	1.49E-06
VSNL1	18.663	15.883	23.938	16.742	23.218	7.058	6.976	6.501	6.709	5.875	-1.5715	down	2.17E-11
TTC32	9.556	8.712	8.732	6.738	2.95	16.108	20.966	10.484	14.419	12.478	1.020961	up	5.19E-08
LOC10174	0.139	0.194	0.166	0.072	0.59	0.279	0.6	0.422	0.83	2.16	1.881426	up	5.33E-17
RHOB	166.363	172.302	92.283	194.081	174.943	54.769	16.948	22.549	31.964	17.479	-2.47676	down	9.25E-28
HS1BP3	8.096	6.814	8.001	7.622	9.342	2.85	3.664	3.822	3.157	3.466	-1.23319	down	2.06E-07
APOB	1455.8	1551.823	478.735	607.197	484.529	2542.741	1321.578	2266.195	2258.436	2302.005	1.223575	up	1.39E-10
CGREF1	0.196	0.154	0.062	0.644	0.845	2.908	2.583	1.21	1.44	1.707	2.370015	up	6.43E-22
SLC5A6	43.773	41.637	47.298	34.546	44.871	29.371	18.05	15.476	17.489	25.328	-1.00471	down	4.95E-05
DTNB	12.246	11.435	2.323	2.942	2.779	0.791	0.934	1.274	1.443	1.14	-2.5057	down	1.33E-28
KIF3C	1.136	1.361	1.274	1.236	1.068	9.508	7.326	2.099	5.194	6.163	2.316936	up	9.18E-30
HADHA	118.676	122.57	136.401	163.422	167.546	62.38	30.752	35.237	64.614	67.317	-1.44481	down	6.75E-10
HADHB	362.649	336.843	399.756	407.366	334.114	108.004	64.79	51.773	116.347	132.107	-1.96029	down	1.39E-17
LOC12111	6.675	7.37	25.456	52.844	35.568	12.293	4.61	2.761	8.91	7.642	-1.82032	down	1.18E-14

GVINP1	13.324	13.946	18.782	19.544	16.333	4.223	3.611	3.299	4.156	4.525	-2.04758	down	4.92E-19
LOC77027	0.949	1.031	0.455	0.826	1.47	0.094	0.12	0.106	0.192	0.032	-3.10879	down	7.28E-32
C3H8ORF8	4.545	4.435	4.744	7.777	3.666	9.622	12.08	14.107	6.836	7.797	1.002949	up	7.52E-08
ZNF395	2.428	2.581	6.638	7.327	10.156	2.709	3.355	3.155	2.552	2.288	-1.05075	down	2.47E-05
LOC42638	0.052	0.183	0.718	0.776	0.394	0.619	1.523	1.075	0.783	0.424	1.057483	up	8.65E-08
FZD3	8.35	7.459	8.094	6.618	7.626	5.091	2.881	2.664	3.935	3.732	-1.05928	down	1.53E-05
MSRA	11.61	11.261	12.555	13.88	16.305	4.31	7.766	4.559	3.547	5.093	-1.37605	down	3.56E-09
SOX7	3.242	3.065	2.072	1.882	2.475	40.338	43.381	27.201	21.715	35.929	3.725789	up	2.77E-63
XKR6	0.687	0.589	1.533	0.802	0.901	0.374	0.595	0.57	0.378	0.336	-1.00032	down	8.43E-05
TDH	348.897	331.718	393.93	562	483.821	184.25	160.393	273.475	190.124	207.18	-1.06223	down	8.76E-06
BLK	0.486	0.712	1.101	0.58	0.771	4.389	3.901	3.96	4.048	4.254	2.491686	up	2.92E-32
FDFT1	37.273	38.699	4.171	6.444	60.069	149.077	37.386	273.467	262.946	137.363	2.552261	up	3.63E-35
DEFB4A	0.275	0.763	1.804	6.43	0	0.1	0.103	0.151	0.341	0.181	-3.39644	down	2.37E-21
MCM3	0.205	0.191	0.262	0.324	0.466	0.819	1.019	1.065	1.315	1.236	1.909602	up	3.98E-20
CRISP2	0.086	0.075	2.056	1.533	0.56	0.209	0	0	0.085	0.076	-3.5244	down	8.68E-22
RHAG	1.114	1.21	2.461	0.895	1.691	0.332	0.19	0.279	0.226	0.301	-2.46816	down	1.37E-17
CYP2AC1	632.367	539.155	1236.022	975.454	1033.102	96.653	149.108	100.393	93.204	121.268	-2.97765	down	1.20E-37
SUPT3H	5.372	5.091	5.659	4.89	14.773	2.103	1.353	2.923	2.01	2.449	-1.72279	down	4.04E-13
RCAN2	2.931	2.809	2.735	5.073	3.064	1.917	1.031	1.404	1.029	1.71	-1.22758	down	2.32E-07
ADGRF5	2.642	2.89	2.883	2.995	1.282	0.46	0.579	0.724	0.745	0.735	-1.96686	down	2.63E-17
TNFRSF21	11.611	11.588	13.062	11.351	13.154	23.777	40.82	28.919	27.289	30.058	1.311832	up	2.99E-12
OPHN1	2.993	2.726	4.357	3.718	4.075	1.785	1.746	2.111	1.486	1.619	-1.03018	down	3.86E-05
RXFP2	0.231	0.124	0.379	0.123	0.279	0.415	1.099	0.36	0.297	0.305	1.120623	up	1.92E-07
ARR3	1.942	1.912	0.361	1.283	1.343	0.396	0.354	0.28	0.234	0.383	-2.05105	down	2.10E-15
LOC42214	0.586	0.777	0.778	0.914	0.602	2.668	1.644	2.984	3.105	1.944	1.753807	up	1.74E-17
RAB33A	0.709	0.497	1.612	0.333	0.364	0.368	0.379	0.335	0.05	0.2	-1.39658	down	0.00022
CCNB3	0.287	0.137	0.458	0.193	0.295	1.064	0.352	0.749	0.986	0.655	1.470738	up	2.18E-10
DLG3	4.19	3.511	4.511	4.103	6.356	3.151	1.832	1.668	2.002	1.725	-1.12694	down	4.91E-06
SLC6A14	0.466	0.499	0.399	2.202	1.155	0.415	39.085	1.324	0.775	1.415	3.186282	up	1.04E-46
GPC3	0.465	0.397	0.123	0.324	0.362	0.656	1.774	0.529	1.134	1.172	1.652781	up	5.68E-14
XKRX	2.891	2.664	4.81	3.368	3.8	10.449	7.917	7.812	11.71	7.992	1.387538	up	8.83E-14
LOC77161	0.701	0.55	0.764	0.479	0.465	1.005	1.858	1.214	1.05	1.096	1.071223	up	3.24E-08
ZNF185	1.259	0.894	0.224	0.225	0.477	1.29	1.576	1.389	2.082	1.925	1.422561	up	1.78E-12
NSDHL	29.648	29.047	3.061	3.52	36.354	47.213	29.141	78.178	72.634	56.764	1.482163	up	1.57E-14
RASL11AL	2.111	2.087	2.54	1.697	2.458	0.649	0.738	1.604	0.676	1.084	-1.19624	down	4.84E-06
SLC16A2	78.76	83.669	98.773	104.677	105.799	21.539	15.335	24.847	23.647	25.164	-2.09329	down	7.94E-20
IRS4	24.867	22.638	34.105	10.963	15.878	3.501	6.046	5.639	3.607	5.767	-2.14243	down	2.20E-20
COL4A5	0.178	0.12	1.061	0.118	0.092	0.086	0.141	0.12	0.089	0.15	-1.41321	down	1.84E-06

LOC11253	3.063	2.663	2.736	2.793	3.338	0.054	0.056	0.494	0.148	0	-4.26933	down	7.48E-42
IDS	14.08	11.138	9.806	16.463	11.422	4.497	9.065	6.819	4.093	5.332	-1.07754	down	3.82E-06
KLHL2	2.451	2.537	2.763	2.055	4.084	6.202	7.473	8.368	7.571	5.587	1.341255	up	8.70E-13
MSMO1	201.642	197.348	11.709	32.025	267.106	449.86	263.289	649.554	589.28	500.233	1.788533	up	1.18E-19
MGARP	12.176	11.256	20.313	18.498	21.268	6.329	5.172	9.222	9.517	7.578	-1.14279	down	2.98E-06
LOC42244	1.917	2.364	2.407	2.242	0.454	1.287	1.436	0.488	0.659	0.633	-1.05848	down	0.000105
SMAD1	5.108	4.88	9.267	2.854	4.077	1.405	2.215	1.704	1.753	1.824	-1.55622	down	7.04E-11
TTC29	2.221	1.929	0.238	0.327	0.153	0.299	0.009	0.645	0.278	0.205	-1.75774	down	7.03E-14
ARHGAP1	1.335	1.045	3.268	1.784	2.793	0.817	1.046	0.963	1.437	0.725	-1.03483	down	0.000125
SH3D19	10.415	9.615	10.782	9.98	13.972	4.924	6.445	5.309	5.116	3.936	-1.08963	down	5.29E-06
HGSNAT	23.972	24.866	23.62	18.959	24.172	8.884	9.982	12.816	11.717	12.254	-1.05441	down	1.30E-05
SLC20A2	29.565	28.28	29.227	25.668	42.152	9.551	7.908	12.339	8.557	11.518	-1.63483	down	2.03E-12
ETNPPL	11.28	12.171	27.882	108.246	165.325	32.513	18.592	32.067	25.652	30.274	-1.22388	down	2.91E-07
SGMS2	8.309	7.447	20.464	10.621	13.39	5.797	5.865	5.319	7.295	5.326	-1.02469	down	6.49E-05
GSTCD	2.36	2.068	0.734	0.766	1.821	3.272	2.565	2.682	3.542	4.68	1.110804	up	4.24E-09
ARHGEF38	1.002	0.876	0.197	0.258	0.592	0.196	0.146	0.116	0.089	0.227	-1.91121	down	1.18E-13
ACSL1	167.114	151.745	183.9	262.414	249.984	29.2	26.078	23.141	29.707	24.551	-2.93567	down	2.81E-37
WWC2	28.127	23.253	60.659	14.844	11.517	10.171	9.198	8.755	6.237	8.021	-1.7072	down	9.37E-13
DCTD	8.876	9.32	6.528	9.438	11.396	4.708	4.187	3.102	3.399	2.974	-1.31012	down	2.39E-08
VEGFC	7.835	8.058	18.325	5.016	10.031	5.382	2.402	5.949	4.398	4.233	-1.13921	down	1.12E-05
AFF1	19.534	19.478	21.029	16.734	23.485	7.302	15.741	8.788	7.795	8.438	-1.06064	down	8.88E-06
PLACL2	2.75	3.512	6.256	7.048	3.026	8.573	35.211	15.021	7.738	3.889	1.640202	up	1.34E-15
GPAT3	7.264	7.386	8.397	9.107	17.669	3.41	2.726	1.977	3.012	2.82	-1.83669	down	4.63E-15
DUSP4	0.599	1.122	0.629	0.47	1.118	4.198	3.303	1.621	3.244	2.12	1.877792	up	1.35E-18
PPP1R3B	34.493	34.88	47.797	51.492	57.78	125.856	108.95	216.58	155.714	79.99	1.601336	up	6.66E-17
LOC43030	0.074	0.101	0.177	0.084	0.065	0.299	7.787	0.529	7.115	0.226	4.97927	up	1.83E-89
SHROOM3	0.496	0.407	1.226	0.995	0.284	0.115	0.131	0.252	0.166	0.046	-2.25503	down	1.57E-19
STBD1	171.185	170.553	139.042	90.513	109.251	32.618	39.1	50.019	29.005	33.415	-1.88572	down	1.14E-16
NPFFR2	0.86	1.092	0.498	0.707	0.482	0.266	0.469	0.345	0.181	0.344	-1.17846	down	5.55E-05
ADAMTS3	1.836	1.893	2.519	1.536	1.694	2.806	7.85	2.351	2.878	3.49	1.031153	up	1.65E-08
SULT1B	30.586	33.569	49.609	13.958	87.131	4.221	6.518	6.186	2.209	4.042	-3.21237	down	7.48E-42
FABP2	0	0	0	1.883	0.102	0	0.149	0	0	0.394	-1.86052	down	0.000603
ENPEP	8.84	8.815	9.553	7.139	5.576	13.337	45.574	19.391	25.39	15.73	1.580656	up	4.93E-16
ELOVL6	237.411	217.439	293.623	330.61	345.785	734.435	486.874	545.832	673.025	920.714	1.238008	up	1.57E-11
CASP6	17.864	14.593	13.318	12.265	6.664	24.768	25.525	26.298	25.188	29.729	1.023167	up	2.49E-08
EMCN	1.074	1.237	2.19	2.072	1.795	0.771	0.858	1.138	0.498	0.832	-1.02942	down	8.59E-05
CCDC110	0.033	0	0	0.26	0.017	1.019	1.873	0.074	0.646	0.242	3.614803	up	6.97E-35
CYP4V2	99.216	95.434	171.671	142.949	178.717	26.577	21.464	34.347	24.468	22.103	-2.41542	down	1.25E-25

KLKB1	5.685	4.713	6.524	6.038	7.461	1.356	4.059	3.612	1.983	1.601	-1.27005 down	5.28E-08
FAT1	0.372	0.457	0.678	0.415	0.456	1.885	2.089	2.503	2.355	2.504	2.250697 up	4.77E-29
PDGFRL	1.353	1.265	4.402	0.842	1.012	0.619	0.29	0.298	0.461	0.23	-2.22212 down	3.37E-16
DLC1	3.678	3.684	1.923	3.605	1.545	0.933	0.722	1.34	1.034	1.235	-1.45447 down	2.03E-10
LRRC66	0.271	0.239	1.178	0.277	0.231	0.163	0.323	0.124	0.242	0.198	-1.06092 down	0.000747
ATP10D	5.487	6.072	6.017	5.538	7.728	2.756	2.934	2.018	2.648	3.194	-1.18631 down	7.66E-07
LIMCH1	1.223	1.452	1.144	1.488	1.049	0.65	0.312	0.823	0.65	0.638	-1.04726 down	2.21E-05
UCHL1	4.533	3.235	1.078	0.242	16.085	8.395	15.699	11.04	13.274	10.337	1.222425 up	1.24E-10
UGDH	74.996	73.369	72.6	137.357	137.927	35.809	27.056	69.839	69.755	38.212	-1.04399 down	1.21E-05
FAM114A1	3.049	2.595	2.04	3.165	2.268	6.953	8.791	5.904	8.658	5.602	1.452519 up	9.03E-14
TBC1D1	6.054	5.95	6.763	5.365	6.448	13.558	11.582	14.08	12.782	11.904	1.06324 up	4.32E-09
CCKAR	0.033	0.011	0.106	0	0	0.921	0.674	3.125	3.873	2.002	6.095652 up	9.32E-86
RBPJ	16.543	15.854	18.495	3.857	20.887	0.974	2.213	1.57	1.011	1.07	-3.46647 down	3.80E-49
SMIM20	46.548	46.282	63.697	45.784	50.318	18.257	25.982	26.264	16.971	20.238	-1.2298 down	2.70E-07
SEL1L3	23.422	22.452	35.091	17.068	33.257	5.785	7.382	7.896	8.064	8.472	-1.80385 down	1.25E-14
ZCCHC4	7.05	6.119	6.587	7.995	7.072	2.428	3.239	3.681	3.385	2.451	-1.19722 down	6.62E-07
PPARGC1A	6.235	5.475	3.024	3.071	5.326	0.29	0.596	0.378	0.17	0.226	-3.79655 down	1.19E-55
LCORL	3.805	3.206	5.222	1.811	2.599	0.977	1.377	1.671	1.576	1.18	-1.29472 down	9.31E-08
TAPT1	47.392	40.82	61.838	35.368	31.753	12.171	12.923	15.844	13.299	17.22	-1.60362 down	7.10E-12
PROM1	2.899	2.262	1.806	3.563	2.459	0.745	1.833	0.851	1.295	1.527	-1.05453 down	8.45E-06
FGFBP2	1.379	0.822	0.581	1.2	1.166	0.177	0.729	0.358	0.362	0.375	-1.36109 down	7.81E-06
KIAA0232	37.057	33.87	37.078	21.903	38.876	13.627	15.193	20.384	17.101	13.965	-1.0722 down	9.86E-06
HAUS3	0.566	0.536	0.789	0.756	0.582	2.81	1.697	1.272	2.441	1.976	1.657319 up	1.11E-16
FGFR3	4.415	4.249	5.699	4.139	10.984	13.814	17.462	11.003	12.658	14.365	1.232726 up	1.67E-11
SPON2	0.232	0.346	0.632	3.372	0.765	0.217	12.07	0.584	0.575	0.502	1.382425 up	1.99E-10
ATOH8	15.211	13.715	16.493	12.129	13.617	4.527	5.528	5.495	2.116	3.919	-1.72091 down	8.41E-14
SMYD1	1.567	1.467	0.174	1.386	0.571	2.714	33.438	4.114	8.355	4.191	3.352766 up	1.49E-48
SMOX	2.88	2.474	1.268	1.19	1.487	2.388	2.001	4.224	5.274	4.885	1.013044 up	7.44E-08
DNAAF9	11.125	10.15	14.327	9.582	12.81	3.62	5.093	6.252	3.647	3.679	-1.37924 down	4.86E-09
NAT8	10.089	11.661	7.855	21.419	23.949	2.284	3.359	3.462	7.404	2.072	-2.01225 down	2.03E-17
LOC10705	0.718	0.515	1.035	0.787	0.738	1.742	2.51	2.339	2.378	1.67	1.486729 up	3.90E-12
TMEM109	8.362	9.296	10.342	13.653	11.82	4.953	5.543	5.143	2.021	4.347	-1.28066 down	8.98E-08
DAGLA	1.444	1.387	0.965	0.936	0.73	1.345	6.943	1.03	1.01	1.722	1.14081 up	3.79E-09
TBX10	0.043	0.114	0.173	0.214	0.357	0.253	1.301	0.192	0	0.516	1.323201 up	2.86E-05
TCIRG1	11.835	12.294	15.393	16.677	12.762	31.059	81.745	23.646	21.852	27.976	1.433539 up	1.04E-13
ALDH3B1	0.558	0.584	0.83	1.865	1.491	0.46	0.111	0.205	0.24	0.172	-2.16035 down	6.71E-15
SPTB	1.182	1.253	2.213	0.42	1.11	0.19	0.333	0.224	0.393	0.268	-2.12955 down	4.21E-18
C15orf52	0.882	0.954	1.441	0.955	1.406	0.642	0.472	0.377	0.259	0.583	-1.27119 down	9.38E-07

LOC77049	0.888	0.937	1.205	0.879	1.057	0.499	0.393	0.579	0.421	0.426	-1.09755	down	0.000113
ANO9	5.533	4.989	3.658	2.954	6.964	2.435	2.558	3.127	2.01	1.826	-1.01087	down	3.40E-05
B4GALNT4	0.042	0.094	0.181	0.159	0.182	1.19	0.379	0.595	0.36	0.401	2.14382	up	1.85E-21
LOC77061	77.416	82.614	158.739	190.458	73.271	323.004	364.608	369.858	128.282	118.969	1.163409	up	3.80E-10
DHCR7	20.697	21.731	2.016	2.903	33.831	60.049	33.799	78.16	97.967	55.142	2.001732	up	1.10E-23
RCN1	5.229	5.745	5.444	5.877	7.058	21.777	16.122	10.689	24.479	16.144	1.603549	up	9.14E-17
PRRG4	1.373	1.063	4.372	1.151	2.014	4.749	11.5	5.346	9.405	5.64	1.876794	up	2.17E-22
MICAL2	0.179	0.201	0.409	0.404	0.156	0.728	2.296	1.109	0.683	0.702	2.028224	up	1.46E-23
CTR9	19.514	17.788	20.673	15.939	12.404	6.85	8.223	8.99	7.839	7.847	-1.11864	down	2.94E-06
AMPD3	1.358	1.168	1.381	0.697	1.199	0.417	0.311	0.446	0.335	0.267	-1.70535	down	2.68E-12
SWAP70	6.877	6.093	8.907	7.418	11.679	4.006	4.799	4.516	3.416	3.337	-1.0292	down	3.15E-05
PDE3B	12.752	11.286	18.888	14.52	16.245	4.346	4.169	5.969	5.009	4.967	-1.59087	down	1.32E-11
USH1C	0.565	0.732	0.705	0.752	0.89	0.977	1.692	1.568	1.982	1.452	1.072854	up	1.91E-08
SAA	18.068	17.014	34.513	38.923	20.811	49.009	3797.536	153.112	22.718	54.667	4.978351	up	2.43E-96
TMEM86A	132.445	119.628	82.819	229.35	159.732	15.28	34.54	34.549	15.206	16.854	-2.63644	down	9.45E-32
KCNQ1	1.006	0.859	0.601	0.536	0.536	0.759	1.841	1.684	1.581	1.844	1.122508	up	8.46E-09
TRPM5	0.198	0.373	0.151	0.965	0.144	5.28	18.214	3.866	18.324	8.598	4.885969	up	1.81E-89
CD81	133.864	112.47	84.628	149.555	110.353	203.118	361.18	200.926	262.176	235.476	1.095794	up	5.19E-09
CTSD	96.128	125.637	250.72	233.262	767.082	63.109	21.81	81.663	99.061	81.816	-2.08366	down	7.01E-19
DUSP8	0.615	0.733	1.304	0.308	0.427	0.966	2.782	1.976	3.317	3.118	1.84241	up	1.87E-21
EFCAB4B	0.997	1.011	6.004	0.917	0.843	0.638	0.743	0.715	0.677	0.636	-1.51793	down	4.92E-09
PNPLA2	44.613	45.171	100.424	37.369	99.724	14.794	11.352	20.073	14.611	13.153	-2.14528	down	5.87E-20
LRRC27	6.991	6.273	3.024	7.334	3.841	1.307	2.721	2.52	1.639	1.946	-1.43798	down	2.05E-10
RNH1	14.679	15.225	17.425	14.465	15.323	28.4	51.59	20.544	24.4	30.511	1.011236	up	2.68E-08
CHKA	20.577	15.45	22.142	9.008	3.506	38.663	309.043	40.806	65.064	49.585	2.831497	up	6.03E-41
LRP5	9.98	9.821	11.022	8.817	10.885	4.928	3.473	5.688	4.98	5.117	-1.06267	down	1.31E-05
TESMIN	0.48	0.358	1.521	0.412	0.316	0.293	0.385	0.257	0.26	0.166	-1.17858	down	7.45E-06
CPT1A	107.933	94.737	63.556	70.728	127.52	9.061	11.133	13.294	8.85	13.877	-3.04646	down	6.36E-40
SMTNL1	0.484	0.481	0.17	1.098	0.795	0.282	0.29	0.256	0.211	0.204	-1.28113	down	9.33E-06
PGR2/3	0.309	0.121	0.147	1.826	0.127	0	0.092	0.136	0	0	-3.44358	down	3.03E-11
SMIM38	1.844	1.327	0.147	1.819	0.474	0	0	0	0	0	-10.1334	down	8.13E-22
FGF19	0.264	0.104	0.196	0.174	0.036	2.743	138.887	2.487	1.925	2.116	7.571346	up	#######
HSD17B12	62.841	63.515	54.042	59.362	45.414	132.776	87.097	96.568	142.548	143.811	1.079824	up	3.57E-09
MDK	0.867	1.267	3.207	3.519	3.744	0.816	0.802	0.45	1.038	0.505	-1.80199	down	4.49E-12
DLL4	8.998	8.845	6.018	8.046	6.421	4.404	3.131	3.815	3.391	3.543	-1.06761	down	6.66E-06
CHAC1	25.729	26.957	18.29	109.452	33.208	19.676	6.933	8.43	0.843	3.727	-2.43111	down	2.32E-27
ITPKA	13.728	13.649	17.334	12.274	17.675	6.219	3.547	5.784	8.27	5.559	-1.3454	down	2.17E-08
LTK	0.571	0.468	1.745	0.501	0.445	0.363	0.305	0.188	0.319	0.165	-1.4735	down	3.60E-08

SPTBN5	0	0	0.003	0.035	0	0.014	2.528	0.007	0	0.009	5.897353	up	8.62E-85
EHD4	0.987	0.714	1.411	1.028	0.875	1.829	3.628	2.57	1.157	1.829	1.134103	up	1.18E-09
PAPLN	5.324	4.912	26.664	11.121	42.818	5.514	4.567	3.385	3.659	3.275	-2.15447	down	1.09E-19
PSEN1	10.218	12.069	20	9.139	27.322	6.477	5.339	7.93	9.313	7.492	-1.10723	down	1.25E-05
TTC9	0.429	0.374	1.343	0.658	0.411	1.581	4.583	5.235	1.363	5.322	2.48998	up	1.46E-29
PLEKHD1	1.744	1.588	1.985	2.026	0.927	1.002	0.737	0.82	0.782	0.758	-1.01173	down	4.33E-05
RDH11	4.659	5.179	3.221	1.487	5.496	9.572	6.908	13.333	16.852	11.635	1.540233	up	1.14E-15
LGALSL2	0.62	0.766	1.617	0.324	1.405	0.144	0.359	0.311	0.28	0.168	-1.90256	down	5.62E-12
BMF	5.662	5.645	5.91	6.368	3.424	1.177	1.426	2.889	1.323	1.463	-1.70548	down	9.41E-14
LOC11253	1.76	1.317	4.659	1.746	0.481	1.076	0.954	0.454	0.604	0.84	-1.34167	down	1.99E-07
DTD2	20.628	21.732	17.03	14.625	7.478	3.458	5.908	11.377	6.127	6.274	-1.2978	down	3.63E-08
LOC11253	0.651	0.908	0.449	1.141	0.49	0	0.13	0	0.086	0.802	-1.83272	down	8.14E-08
SLC25A21	0.987	0.983	1.093	1.166	1.457	0.484	0.453	0.346	0.429	0.475	-1.37643	down	1.15E-08
TTC6	0.807	0.437	2.69	0.156	0.257	0.108	0.195	0.184	0.138	0.098	-2.57967	down	2.28E-23
CLEC14A	2.212	2.435	1.824	0.706	2.085	0.644	1.326	0.804	0.543	0.721	-1.19668	down	6.08E-06
ACSS1B	42.926	41.52	46.602	47.98	76.447	2.126	7.838	5.925	3.123	2.039	-3.60091	down	3.55E-53
FOS	4.941	4.658	12.706	1.15	4.038	44.037	3.29	2.852	2.656	2.712	1.014512	up	1.18E-09
FLVCR2	0.555	0.363	0.771	0.585	1.293	5.843	0.819	0.311	0.485	6.536	1.97052	up	1.96E-23
LOC11253	0.714	0.992	0.992	1.122	1.565	0.589	0.35	0.377	0.552	0.509	-1.17811	down	3.34E-06
GALC	0.376	0.449	0.833	0.893	1.745	0.155	0.22	0.162	0.454	0.28	-1.75304	down	1.86E-11
GPR65	3.171	3.009	4.794	4.244	3.273	1.565	2.469	1.628	1.419	1.575	-1.09461	down	7.25E-06
TRIP11	8.883	7.779	10.737	6.351	6.682	15.847	16.74	24.77	25.909	20.984	1.366369	up	2.25E-13
SPIA9	59.899	55.12	78.31	50.268	58.189	22.068	16.91	27.033	28.801	26.668	-1.31277	down	3.81E-08
SPIA3	23.911	21.353	55.387	26.868	31.373	3.644	371.02	9.655	5.176	5.894	1.315199	up	6.88E-12
LOC10704	0.496	0.568	1.415	0.464	0.156	0.241	0.31	0.152	0.041	0.018	-2.01683	down	2.63E-12
BDKRB1	0.133	0.237	0.303	0.219	0.098	0.139	1.313	0.799	0.511	0.176	1.564519	up	3.80E-10
LOC12111	3.707	3.001	0.024	3.645	0	4.686	0	0	0	0	-1.14612	down	2.05E-06
DIO3	1.688	1.42	0.299	0.352	1.486	0.936	0.241	0.059	0.08	0.424	-1.58909	down	2.51E-08
ANKRD9	2.95	3.165	5.967	2.546	9.757	133.891	46.319	40.224	106.165	70.61	4.025555	up	1.53E-73
AMN	2.966	4.119	6.067	4.949	11.505	2.29	1.47	0	0.861	0.391	-2.56124	down	1.53E-25
ASPG	18.309	17.346	24.985	31.987	42.107	15.181	10.953	10.695	12.03	14.083	-1.09796	down	6.78E-06
ZBTB42	5.144	5.403	5.407	4.619	5.924	2.204	3.64	2.171	2.352	2.467	-1.04557	down	1.58E-05
SYNE2	5.652	5.467	11.555	7.251	7.614	2.897	4.769	4.168	3.381	2.998	-1.04322	down	2.59E-05
DHRS7	868.149	797.527	1003.922	724.93	1188.741	197.97	205.575	267.985	190.678	228.158	-2.07156	down	1.96E-19
DLGAP5	0.257	0.168	0.303	0.322	0.349	0.479	0.555	0.659	1.074	0.761	1.331351	up	1.03E-10
GCH1	87.373	94.623	99.629	67.794	186.64	37.366	29.501	40.608	34.846	34.486	-1.60019	down	9.17E-12
DNAAF2	4.219	5.045	5.3	4.931	3.484	2.219	2.904	1.478	2.081	2.051	-1.0979	down	6.48E-06
SOS2	9.302	9.379	11.862	14.758	15.676	4.644	4.803	5.127	6.055	5.056	-1.24718	down	1.44E-07

CDKL1	2.515	2.163	2.554	3.303	3.6	1.239	0.801	1.966	1.278	1.359	-1.08879 down	1.88E-05
ABHD12B	9.122	10.475	17.701	10.405	16.707	38.007	42.572	21.32	23.157	35.199	1.314946 up	1.09E-12
LOC10705	0.159	0.295	0.232	0.327	0.436	1.003	0.675	1.053	0.922	0.945	1.662547 up	4.67E-12
LOC11253	2.635	2.219	2.157	2.217	1.423	0.504	0.724	0.486	1.304	0.263	-1.69726 down	1.57E-12
LOC12110	0.982	0.989	1.19	1.01	0.727	0.296	0.278	0.371	0.606	0.525	-1.23639 down	1.74E-06
GRID1	0.591	0.53	1.061	0.147	0.01	0.115	0.209	0.123	0.035	0.221	-1.72715 down	4.03E-12
SNCG	0.037	0.016	0.07	0.041	1.68	0	0.056	0.083	0	0.016	-3.5306 down	1.05E-21
SYT15	0.881	1.287	0.587	0.657	0.042	0.686	0.123	0.045	0.346	0.135	-1.36812 down	1.75E-06
MAT1A	854.212	791.908	1019.408	1058.651	1312.731	367.404	427.45	544.539	503.246	383.039	-1.17829 down	7.29E-07
IPMK	10.129	10.308	20.627	10.968	13.361	5.421	5.224	6.683	6.217	7.212	-1.0881 down	1.49E-05
SIRT1	13.331	12.185	22.975	11.512	15.861	6.103	7.773	8.284	7.049	5.932	-1.11015 down	7.37E-06
DNAJC12	47.04	41.616	64.729	49.829	55.959	4.536	12.112	13.888	7.331	5.336	-2.58457 down	2.42E-29
LOC10174	0.43	0.384	1.682	0.08	0.121	0.313	0.498	0.216	0.117	0.026	-1.20137 down	0.000214
PAPSS2	103.418	92.74	139.544	99.126	136.152	58.517	46.205	41.675	61.101	72.655	-1.02721 down	3.74E-05
MINPP1	4.607	5.521	6.141	5.124	8.359	3.935	1.2	2.32	2.736	4	-1.06774 down	3.38E-05
ASAH2	0.194	0.184	2.521	0.231	0.199	0.282	0.184	0.116	0.087	0.232	-1.87967 down	5.57E-09
PRKG1	0.334	0.412	0.524	1.475	0.744	5.952	0.309	4.229	0.349	0.294	1.672539 up	4.74E-17
HKDC1	0.042	0.172	0.528	0.207	0.076	41.757	34.517	160.708	116.367	127.175	8.865835 up	#######
DUSP13	0.226	0.207	0.18	0.185	0.973	0.459	4.528	0.1	1.344	0.774	2.021368 up	1.96E-15
NTL	0.863	0.834	1.408	0.486	0.702	0.376	0.567	0.407	0.44	0.347	-1.00471 down	0.000132
FUT11	2.008	2.139	5.104	1.282	1.514	0.995	0.832	1.744	1.04	0.733	-1.17193 down	1.46E-05
RP11-400	128.75	134.467	41.282	34.419	60.482	0.119	5.076	5.539	1.48	46.856	-2.75723 down	3.10E-33
CYP2C18	1704.44	1566.283	682.717	855.24	1306.675	0.066	37.281	42.313	10.931	332.88	-3.85209 down	2.13E-59
TLL2	1.238	1.179	1.329	0.949	1.964	0.177	0.555	0.39	0.368	0.445	-1.78033 down	1.49E-13
TMEM150	0.686	0.742	0.967	1.236	0.623	2.035	2.609	1.455	1.704	1.951	1.196219 up	5.75E-09
SCD	143.146	170.585	16.981	51.148	16.442	1155.364	377.206	1623.133	1515.368	1498.13	3.953132 up	2.55E-68
CYP2C23b	588.344	527.607	295.271	352.718	570.35	49.712	65.791	124.326	125.23	52.728	-2.48213 down	6.22E-28
CYP2C23a	1174.55	1044.819	1141.807	758.226	1003.909	222.783	129.211	394.946	255.545	224.419	-2.06205 down	2.44E-19
MSMB	5.047	4.939	8.814	1.882	4.879	5.739	31.197	13.567	27.044	13.493	1.832353 up	9.23E-21
WDFY4	1.093	1.128	2.098	1.624	1.811	0.575	0.604	1.009	0.693	0.573	-1.16552 down	2.50E-06
LOC11253	0	0	0	0	0	0	3.51	0	0.02	0	9.465566 up	2.65E-71
ANKRD22	0.151	0.049	0.06	0.248	0.039	0.494	1.807	0.416	0.637	0.348	2.747512 up	9.08E-18
ACTA2	7.16	6.919	10.318	9.822	12.616	17.852	28.591	20.232	17.739	19.032	1.143135 up	4.66E-10
CH25H	0.517	0.301	1.075	0.614	0.531	0.292	0.258	0.253	0.171	0.19	-1.38022 down	5.06E-05
LIPA	43.175	45.902	83.607	73.074	96.269	32.472	23.88	34.595	40.515	28.694	-1.09461 down	9.22E-06
PANK1	87.26	75.486	129.212	87.423	116.202	23.7	21.823	29.43	23.997	24.785	-2.00183 down	5.09E-18
PPP1R3C	27.675	27.046	37.051	57.057	54.179	44.449	43.966	121.608	146.91	89.297	1.136232 up	5.23E-10
MYOF	0.269	0.221	1.019	0.173	0.076	0.276	0.182	0.139	0.136	0.07	-1.12561 down	0.000148

CEP55	0.339	0.1	2.713	0.188	0.101	0.35	0.147	0.193	0.217	0.101	-1.76629	down	3.82E-09
ENTPD7	2.728	2.122	4.355	1.684	1.938	6.714	6.385	4.042	6.066	6.361	1.204535	up	3.90E-11
PYROXD2	5.419	5.462	4.379	4.948	6.659	2.482	2.075	2.037	2.173	1.563	-1.37857	down	8.90E-09
LOXL4	0.866	0.908	1.01	0.969	0.905	0.217	0.358	0.263	0.163	0.118	-2.05262	down	2.57E-14
CRTAC1	6.912	8.008	0.079	0.244	0.017	0.528	0.025	0.073	0.098	0.196	-4.04463	down	5.41E-52
PI4K2A	22.469	22.979	17.273	12.637	18.764	7.263	7.265	9.154	7.495	8.113	-1.26026	down	8.25E-08
HOGA1	4.35	4.49	2.699	2.135	5.657	0.205	0.762	0.935	0.224	0.746	-2.74865	down	6.16E-27
ANKRD2	0.61	0.412	1.112	0.388	0.283	0	0.206	0.564	0.43	0.182	-1.0186	down	0.001172
TLX1	0.975	1.072	0.882	0.947	0.046	0	0	0	0	0	-9.61728	down	1.16E-27
PDZD7	1.084	0.786	1.142	1.255	0.335	0.628	0.402	0.309	0.208	0.446	-1.20527	down	4.08E-06
WBP1L	16.82	17.486	52.345	19.546	24.976	11.711	11.059	14.033	12.009	15.119	-1.03683	down	7.48E-05
NEURL1	0.37	0.551	0.898	0.955	1.038	0.429	0.366	0.409	0.276	0.422	-1.00113	down	0.000211
GSTO2	5.481	5.978	4.51	2.385	10.692	1.629	2.242	1.584	1.547	2.755	-1.57334	down	2.30E-11
SHOC2	6.921	5.886	6.142	5.648	6.191	3.048	3.604	2.606	3.066	2.875	-1.01815	down	2.54E-05
GPAM	50.126	44.294	35.149	35.867	21.312	304.405	192.447	292.423	273.042	279.146	2.844609	up	9.98E-42
ABLIM1	15.021	13.123	13.275	9.412	9.536	5.506	6.834	6.891	4.71	5.26	-1.04761	down	1.21E-05
HTRA1	29.947	28.065	52.562	37.033	53.256	15.275	21.413	19.639	20.136	18.337	-1.08321	down	1.16E-05
LOC10175	0.605	0.138	0.27	0.482	0.762	2.026	2.484	0.04	0.255	0.629	1.265742	up	3.36E-10
FAM196A	0.873	1.004	4.073	1.217	0.36	0.102	0.22	0.119	0.221	0.261	-3.02084	down	1.87E-34
MKI67	0.168	0.176	0.41	0.273	0.327	0.59	0.525	0.733	1.054	0.577	1.358199	up	1.73E-12
ALDH18A1	4.614	5.091	9.894	5.147	5.39	13.033	20.708	8.228	13.24	13.205	1.18267	up	7.12E-11
C2orf88	0.235	0.165	0.319	0.522	4.145	0.197	1.022	0.947	0.286	0.171	-1.03659	down	5.42E-05
FRZB	56.751	58.945	50.548	40.422	64.142	21.951	16.831	30.655	24.744	21.409	-1.22822	down	2.18E-07
ABCA12	5.11	4.197	11.634	1.146	27.632	0.397	0.188	1.081	0.216	0.129	-4.62437	down	2.88E-77
ATIC	4205.01	4235.959	2567.995	3001.741	2519.42	320.178	454.888	923.917	469.378	598.851	-2.57859	down	4.47E-30
LOC10704	2.429	8.065	4.108	4.732	2.095	0.655	0.764	0.397	1.4	0.356	-2.58308	down	5.18E-25
RAB17	4.15	3.431	3.02	7.218	6.459	1.741	3.623	2.527	1.896	1.68	-1.08183	down	7.38E-06
UGT1A1	39.561	37.223	44.172	46.488	71.827	22.381	18.193	32.517	22.043	20.723	-1.04627	down	1.71E-05
LOC12111	5.572	5.416	23.484	11.677	68.075	0.651	1.307	3.505	0.533	0.473	-4.14113	down	6.44E-62
AHR2	5.188	4.649	7.267	7.84	9.885	2.078	1.684	3.151	1.611	2.699	-1.6334	down	3.87E-12
AHR1B	4.608	3.848	6.309	2.694	3.261	0.395	1.375	0.723	0.698	1.113	-2.26595	down	1.33E-21
SLC19A1	67.212	62.132	29.781	38.601	23.339	23.079	14.787	16.427	16.746	27.869	-1.16027	down	5.17E-07
COL6A2	3.344	4.803	5.366	4.699	6.948	15.474	9.922	16.524	15.528	11.633	1.456975	up	1.08E-14
LSS	53.271	57.707	3.582	3.99	53.273	82.644	56.644	113.581	108.527	79.265	1.35872	up	1.60E-12
LOC76858	0.209	0.219	1.432	0.791	2.062	0	0.627	0.185	0.208	0.11	-2.05548	down	1.48E-09
COQ10B	31.709	32.659	22.38	37.282	58.093	5.842	5.447	4.115	5.872	5.05	-2.79012	down	1.91E-33
GPR1	2.92	2.966	4.328	3.504	5.74	1.416	1.647	1.571	1.844	1.226	-1.33612	down	3.53E-08
RAPH1	8.514	7.701	11.651	7.638	9.274	3.48	4.854	4.636	3.766	3.601	-1.13849	down	2.51E-06

LOC42411	16.941	16.13	19.558	15.841	26.377	5.251	7.599	8.75	9.035	5.943	-1.3745	down	5.06E-09
IDH1	256.182	246.123	290.413	244.149	481.266	77.416	60.615	135.418	114.306	83.506	-1.68769	down	3.68E-13
PIKFYVE	8.55	7.751	16.078	7.097	11.397	2.861	3.086	3.406	3.364	2.956	-1.6983	down	7.00E-13
CDCA7	0.208	0.484	1.145	0.514	0.696	1.244	1.327	1.541	1.729	0.977	1.160651	up	5.54E-10
RAPGEF4	21.935	20.538	17.359	11.14	36.575	2.607	1.865	3.445	3.669	2.983	-2.88357	down	2.50E-35
ITGA6	3.56	3.082	4.726	1.729	2.62	1.191	1.961	1.039	1.106	1.634	-1.18061	down	1.52E-06
SLC25A12	0.935	0.847	3.287	1.644	2.092	0.628	1.059	0.806	0.738	0.743	-1.14674	down	9.57E-06
G6PC2	2.067	1.947	0.812	2.106	1.925	0.461	1.177	0.882	0.616	0.637	-1.23001	down	3.38E-07
SPC25	0.417	0.448	0.332	0.441	0.949	0.52	1.294	1.38	1.391	1.022	1.114449	up	3.80E-07
CERS6	0.521	0.63	0.077	0.368	0.429	1.189	0.372	0.214	0.866	1.75	1.114712	up	1.30E-08
FAP	1.378	1.241	2.817	1.846	3.322	0.556	0.955	1.142	0.918	1.02	-1.20684	down	1.83E-06
DPP4	4.234	3.722	3.601	5.071	9.228	18.989	20.524	16.93	15.112	20.832	1.83699	up	8.81E-21
SLC4A10	0.187	0.138	0.278	0.215	0.281	1.594	1.035	0.673	0.742	1.2	2.249302	up	1.86E-26
ITGB6	0.971	0.877	0.5	0.447	0.438	1.788	6.532	2.773	1.845	2.422	2.246473	up	4.60E-27
LOC42419	0.762	0.715	1.71	0.465	1.364	0.142	0.176	0.086	0.175	0.052	-2.98088	down	3.07E-22
TUB4A	3.194	3.174	6.562	4.328	5.309	2.27	2.301	1.936	1.187	2.189	-1.19078	down	3.36E-06
IHH	4.952	4.974	10.832	8.392	14.985	4.455	3.14	3.899	2.408	3.716	-1.32463	down	8.31E-08
MNR2	0.339	0.423	1.029	0.304	0.379	0.627	2.075	0.883	1.101	1.504	1.321346	up	9.57E-10
PLCD4	0.512	0.711	0.995	0.719	1.412	0.618	0.337	0.276	0.248	0.33	-1.26317	down	4.09E-06
SLC11A1	1.672	2.549	6.26	6.021	11.971	2.208	1.937	2.168	2.586	2.39	-1.33429	down	8.97E-08
IGFBP2	19.814	18.98	11.072	14.401	21.131	0.644	0.071	0.941	0.816	0.605	-4.79235	down	3.16E-79
ARHGEF1	0.476	0.488	0.145	0.235	0.179	1.49	0.488	1.016	1.08	0.629	1.62347	up	1.55E-14
SEMA5B	1.118	1.356	3.459	4.95	3.75	0.931	0.769	1.217	0.935	1.42	-1.47193	down	5.58E-10
MYLK	24.439	24.724	27.659	15.53	18.65	4.256	14.778	9.669	6.613	7.482	-1.37487	down	2.91E-09
SLC12A8	0.247	0.246	0.306	0.554	0.298	1.862	0.843	0.414	0.618	0.991	1.515052	up	1.70E-13
GPD1L2	375.957	349.679	245.855	278.078	253.696	133.936	129.507	161.782	191.568	127.423	-1.0143	down	1.79E-05
DPP10	0.176	0.291	0.436	0.244	0.029	1.283	0.723	1.228	0.184	0.527	1.741844	up	6.93E-16
CXCR4	1.752	1.712	4.595	1.824	1.896	0.952	0.91	0.825	0.464	0.648	-1.63124	down	5.67E-10
NXPH2	4.48	3.663	2.488	1.492	3.923	1.595	1.642	1.196	1.311	0.778	-1.29817	down	6.39E-08
UPP2	40.223	37.093	61.261	47.83	61.272	24.595	20.167	38.162	22.789	14.955	-1.0374	down	2.61E-05
DAPL1	1.429	2.167	2.585	1.387	2.801	0.567	0.778	1.146	0.709	0.514	-1.47999	down	8.84E-07
NTNG1	1.586	1.549	4.667	1.87	2.384	0.803	0.689	0.862	1.233	1.062	-1.37381	down	1.09E-07
RGS2	6.387	5.571	17.322	7.222	13.428	80.023	10.345	6.865	6.344	7.963	1.159503	up	3.13E-11
HTATIP2	4.119	4.442	3.439	3.446	5.307	4.395	14.958	8.206	7.19	12.357	1.182397	up	4.93E-10
HSD17B7	23.99	25.32	1.669	1.756	35.321	74.255	30.819	72.635	75.305	41.26	1.740609	up	7.94E-19
SPATA46	8.132	6.29	7.082	8.79	5.473	4.313	4.561	3.358	1.393	2.327	-1.16464	down	1.81E-06
REG4	0.831	0.502	1.374	0.473	0.853	0	0.479	0	0.317	0.084	-2.18989	down	7.73E-08
LOC12111	7.605	7.426	6.721	3.82	6.56	3.481	3.178	3.634	1.95	2.919	-1.0833	down	1.17E-05

FASLG	0.394	0.358	0.569	0.236	0.405	0.787	1.581	1.518	1.327	1.72	1.818523	up	1.93E-19
SUCO	6.512	6.015	8.369	5.144	6.028	49.062	21.115	25.849	52.674	35.352	2.520721	up	2.89E-35
FMO3	553.076	547.561	600.864	963.532	1308.063	356.313	187.873	515.064	400.437	409.218	-1.08807	down	6.59E-06
RGS8	6.23	6.334	1.454	1.004	4.903	2.482	1.659	1.442	0.639	1.587	-1.35081	down	4.40E-09
RGSL1	1.999	2.292	0.7	0.614	1.794	0.687	0.691	0.63	1.004	0.478	-1.08301	down	1.32E-05
SOAT1	7.702	6.988	20.825	8.325	17.535	2.545	2.832	3.933	3.928	4.475	-1.79255	down	7.40E-14
ABL2	2.044	1.783	3.941	1.973	2.067	8.843	7.134	3.257	4.835	4.673	1.283036	up	2.08E-12
TOR3A	22.63	20.597	13.745	7.774	1.6	104.719	88.903	63.31	80.289	105.908	2.739553	up	4.83E-39
LOC42443	5.333	4.888	7.653	5.026	8.108	14.335	13.586	6.913	17.027	13.085	1.066479	up	3.32E-09
KIAA0040	27.146	26.189	17.705	19.883	28.757	9.781	7.585	8.865	7.351	9.67	-1.46824	down	2.09E-10
RABGAP1l	9.058	8.331	14.313	9.315	13.418	3.876	4.481	5.516	5.777	4.997	-1.14296	down	3.05E-06
SERPINC1	259.983	261.638	354.37	334.689	510.496	158.476	125.249	209.253	171.882	170.531	-1.04287	down	2.22E-05
LRRC39	3.84	3.319	3.018	3.376	3.004	7.295	5.797	10.753	10.226	4.938	1.236115	up	5.94E-11
TRMT13	7.791	7.305	6.02	7.143	6.554	15.709	10.251	21.217	21.125	11.231	1.191811	up	2.37E-10
DPYD	25.495	22.231	34.998	31.027	35.721	18.816	9.493	12.153	10.283	0.093	-1.5558	down	2.41E-11
ALG14	3.916	4.103	3.079	2.195	3.494	1.714	2.088	1.171	1.055	1.752	-1.10901	down	7.33E-06
LOC42449	6.844	6.306	8.694	4.807	4.841	3.515	2.164	2.456	2.893	1.97	-1.27637	down	2.46E-07
ZNF644	6.697	6.357	12.756	6.691	7.442	3.493	4.159	3.979	4.035	3.595	-1.05207	down	2.75E-05
CYR61	1.014	1.197	3.071	1.956	2.309	8.755	5.911	0.827	2.43	1.397	1.016594	up	2.46E-08
LPAR3	0.723	0.746	3.989	2.287	2.613	1.677	9.581	5.098	3.078	2.798	1.10152	up	7.59E-10
SSX2IP	2.355	1.78	1.613	1.374	0.639	4.687	6.361	4.468	3.551	5.138	1.640359	up	1.62E-16
VTG2	4938.7	5884.15	43.669	1565.017	4.114	14235.24	6981.924	11442.81	11216.08	11992.47	2.167553	up	2.01E-25
LOC12111	3.51	7.943	0	1.355	0	23.959	5.114	22.763	22.115	21.071	2.890729	up	2.50E-40
VTG3	625.379	748.039	2.05	89.471	0.449	2305.642	712.959	1334.77	1635.498	1641.845	2.380531	up	7.05E-30
SPATA1	21.159	19.818	12.843	23.628	2.939	37.123	24.963	34.265	27.995	48.663	1.105765	up	4.82E-09
VTG1	2389.74	3147.829	3.494	185.475	1.276	6856.405	1820.97	4086.828	5269.13	5628.846	2.046527	up	2.95E-23
ARTN	0.496	0.4	0.669	0.361	1.137	0.278	0.372	0.421	0.152	0.076	-1.23435	down	3.81E-05
MMACHC	5.189	4.085	8.857	3.444	4.665	2.987	1.98	2.296	2.193	2.952	-1.08019	down	3.13E-05
RBP	330.724	399.221	17.878	55.352	2.696	1189.007	622.57	689.115	781.522	889.539	2.372026	up	1.05E-29
FAAH	128.693	126.67	82.223	114.864	123.711	38.924	30.064	43.837	40.384	41.593	-1.56444	down	7.26E-12
CYP4A22	50.13	52.443	87.296	66.467	105.941	20.041	23.754	38.029	23.365	17.522	-1.56179	down	2.76E-11
CYP4B7	400.359	358.574	324.755	325.616	466.218	40.144	35.758	31.088	22.851	51.014	-3.37435	down	1.98E-47
PODN	2.283	1.903	5.682	1.607	1.414	0.38	0.497	0.304	0.561	0.247	-2.69296	down	1.35E-29
TCEANC2	1.101	0.955	1.181	0.826	1.548	1.671	2.33	2.692	2.59	2.242	1.037778	up	7.51E-08
DHCR24	22.675	25.84	0.604	0.787	51.673	106.502	55.119	113.639	140.644	109.105	2.369683	up	2.08E-31
PCSK9	1.734	1.224	2	1.29	0.982	0.298	0.716	0.271	0.298	0.523	-1.77707	down	3.18E-12
C8B	89.547	86.71	117.459	114.207	87.322	42.439	64.624	25.271	37.36	51.031	-1.16587	down	8.27E-07
DAB1	0.954	0.815	3.121	1.119	2.971	0.398	0.71	0.255	0.308	0.698	-1.9202	down	8.89E-15

TACSTD2	1.413	1.345	2.08	0.809	0.924	0	0.706	0.094	0.553	0.622	-1.73171	down	2.24E-08
HOOK1	6.839	5.512	9.275	6.471	4.374	14.775	15.86	23.646	28.553	21.586	1.685021	up	2.11E-18
KANK4	4.823	5.238	3.25	8.253	10.794	15.178	24.817	14.847	11.689	4.92	1.14271	up	1.69E-09
ANGPTL3	47.918	48.945	26.193	51.599	48.217	327.727	187.975	395.832	432.861	193.293	2.786448	up	4.62E-40
NGEF	6.018	5.911	5.608	6.3	6.487	12.555	14.1	12.799	14.173	14.525	1.168164	up	2.95E-10
NEU2	1.117	0.817	0.314	0.576	0.788	0.733	2.396	1.933	1.131	1.909	1.164375	up	3.18E-08
KLHL24	23.395	22.179	23.13	14.804	19.244	5.321	4.06	6.629	4.458	5.884	-1.96298	down	1.55E-17
EHHADH	274.411	257.616	291.099	309.829	356.991	9.661	40.381	66.589	56.016	56.638	-2.70002	down	4.81E-32
DNAJB11	20.71	21.364	20.588	17.848	19.015	66.25	39.741	31.886	65.383	52.905	1.363898	up	3.96E-13
RNF168	11.239	10.541	9.913	10.11	9.755	4.307	5.154	4.808	5.087	4.44	-1.11531	down	2.69E-06
PTTG1IP	11.334	11.758	9.053	8.323	10.311	3.848	4.905	5.517	3.788	3.325	-1.24757	down	1.17E-07
GPR35	1.564	1.353	1.322	0.83	1.952	0.63	0.505	0.318	0.645	0.476	-1.44589	down	1.56E-07
PER2	31.449	25.95	43.329	19.482	12.35	12.643	7.923	6.961	10.411	18.511	-1.23155	down	4.71E-07
CLSTN2	0.41	0.553	1.235	1.126	0.979	0.334	0.122	0.433	0.15	0.49	-1.48972	down	1.34E-09
PXYLP1	2.951	2.654	2.915	4.503	1.882	9.326	7.994	6.572	8.625	9.47	1.493834	up	1.03E-14
EPHA4	0.37	0.304	0.501	0.657	0.911	2.086	0.687	1.05	1.605	1.389	1.311813	up	4.89E-12
SGPP2	0.885	0.962	0.263	1.936	3.467	0.218	0.067	0.033	0.089	0.099	-3.87895	down	1.32E-40
MOGAT1	1.422	1.726	2.128	2.632	8.621	1.495	1.206	1.058	1.786	1.447	-1.24063	down	1.26E-06
CCL20	1.441	1.034	1.665	0.076	0.477	0.135	1.462	0.103	0.046	0.061	-1.37446	down	1.47E-05
PID1	3.95	4.85	3.242	4.344	2.06	0.973	1.548	1.743	1.026	1.364	-1.47032	down	1.86E-08
TFDP2	1.074	1.12	2.805	0.755	0.915	0.389	0.576	0.559	0.773	0.681	-1.16179	down	8.88E-06
GK5	17.146	17.654	25.66	10.469	12.64	5.246	5.185	3.995	9.295	7.361	-1.42674	down	3.03E-09
PCOLCE2	2.885	2.765	3.479	2.576	4.171	0.466	0.178	0.576	0.66	0.627	-2.66039	down	2.68E-27
PAQR9	70.988	65.971	74.423	53.695	72.103	34.01	27.013	36.489	27.937	39.825	-1.02864	down	2.66E-05
PLOD2	13.752	12.993	15.679	16.286	8.696	31.915	39.39	37.146	40.977	31.638	1.4255	up	7.74E-14
HRASLS	2.403	2.567	1.734	5.152	3.432	8.604	11.08	5.135	1.864	7.657	1.167229	up	1.78E-09
RTP2	5.707	6.898	5.74	12.564	6.633	1.792	2.745	1.524	1.074	1.943	-2.04746	down	3.53E-18
PDCD1	0.224	0.244	0.436	0.245	0.332	0.651	0.857	1.207	0.42	0.657	1.353426	up	1.05E-08
C2orf72	45.018	46.835	41.902	41.539	44.64	15.881	26.43	21.395	19.976	17.29	-1.12308	down	1.84E-06
PSMD1	30.125	31.36	32.263	25.944	42.149	13.863	10.372	17.671	16.316	20.137	-1.04636	down	2.06E-05
NPPC	0.462	0.14	0.227	0.205	0.146	1.553	0.693	0.314	1.131	1.127	2.025044	up	5.73E-15
ALPI	2.276	2.978	1.982	7.011	6.817	0.557	0.382	0.375	0.148	0.281	-3.59134	down	1.70E-43
ECE2	0.866	0.899	1.083	0.442	0.614	0.261	0.492	0.099	0.223	0.473	-1.33174	down	1.88E-06
AHSG	647.406	586.191	341.982	385.887	516.601	2564.689	853.369	2411.685	2269.034	1806.507	1.998981	up	9.45E-24
TNFSF10	262.013	262.616	127.053	270.563	196.663	78.831	81.783	156.96	91.026	110.525	-1.10793	down	1.41E-06
MECOM	1.22	1.131	1.726	1.258	1.06	0.61	0.635	0.624	0.337	0.51	-1.23393	down	4.89E-07
LOC10705	0.51	0.516	1.065	0.257	2.652	0.359	0.723	0.629	0.338	0.384	-1.03767	down	0.000102
SMC4	0.936	0.823	1.281	0.925	1.01	2.202	2.211	2.15	3.552	2.036	1.287451	up	1.19E-11

IFT80	2.329	2.327	3.494	2.548	2.656	17.861	11.605	11.334	16.993	14.675	2.439632	up	4.22E-33
MLF1	1.58	1.77	10.575	6.262	8.683	0.596	1.096	1.549	0.625	1.197	-2.51033	down	7.65E-25
C3orf33	4.902	5.566	11.013	5.524	7.193	1.274	4.039	2.549	1.704	1.572	-1.61799	down	4.91E-11
ARHGEF26	23.545	19.621	29.884	21.264	16.511	8.824	4.923	6.409	10.255	10.175	-1.44912	down	9.65E-10
SUCNR1	10.112	8.721	20.68	8.036	5.365	1.216	1.189	3.826	0.913	3.724	-2.28304	down	1.34E-21
SERP1	88.999	94.913	104.069	99.373	99.605	289.942	218.497	118.552	210.243	188.488	1.07476	up	3.14E-09
SHF	1.134	1.214	1.478	0.564	1.231	0.452	0.714	0.319	0.38	0.324	-1.35855	down	6.14E-08
SLC28A2	1.9	1.83	2.645	2.501	4.872	0.854	1.736	0.886	0.967	0.673	-1.42525	down	7.99E-09
DUOX1	0.118	0.177	0.05	0.051	0	7.144	2.168	10.037	5.626	2.834	6.116065	up	#######
KIAA0101	2.021	2.952	1.287	0.996	2.421	7.644	8.779	13.374	18.331	5.869	2.479636	up	2.34E-29
PATL2	0.431	0.311	0.936	0.477	0.321	1.234	1.627	1.247	0.96	0.903	1.268258	up	2.32E-10
CD276	1.304	1.262	1.756	1.389	1.793	3.769	3.303	2.594	2.482	3.08	1.020507	up	4.11E-08
CYP1A1	6.762	6.585	8.984	8.907	42.226	7.843	1.852	6.138	5.047	2.856	-1.62975	down	1.31E-11
CHRNA3	0.746	0.418	1.464	0.81	0.164	0.116	0.479	0.176	0.132	0.07	-1.88289	down	2.81E-09
CHRNA5	2.166	1.387	3.673	1.847	1.455	0.937	1.035	0.672	0.826	0.912	-1.26361	down	8.21E-07
LOC10174	5.183	4.329	2.433	2.012	1.942	0.958	1.366	1.192	1.341	1.226	-1.38535	down	7.46E-09
RORA	3.251	2.92	3.995	3.056	3.04	1.382	1.736	1.92	1.515	0.911	-1.12296	down	3.07E-06
TRPM1	0.314	0.362	0.075	0.487	0.137	0.497	9.422	3.86	0.709	0.782	3.468432	up	5.76E-52
LIPC	10.231	9.369	17.981	17.582	21.798	4.187	1.018	1.234	4.499	3.57	-2.40688	down	1.13E-24
ALDH1A2	1.457	1.178	1.603	2.328	1.631	0.648	0.455	0.804	1.256	0.761	-1.06181	down	2.95E-05
DYX1C1	2.47	2.886	2.81	3.534	3.216	0.995	2.048	1.411	1.468	1.281	-1.04967	down	2.98E-05
SEMA6D	5.433	4.993	4.704	5.885	5.547	0.538	1.332	0.975	0.807	0.405	-2.70937	down	1.39E-32
FBN1	1.163	1.455	1.829	1.864	2.128	2.93	2.555	4.697	4.074	3.89	1.104051	up	1.35E-09
HDC	0.242	0.498	1.184	0.318	0.708	0.211	0.272	0.133	0.3	0.224	-1.36781	down	3.96E-06
GATM	49.717	47.347	38.575	28.987	47.337	118.633	31.433	102.181	138.273	101.911	1.21609	up	3.61E-11
HOMER2	6.603	5.654	6.905	7.428	8.865	2.154	3.335	1.504	1.192	3.054	-1.65704	down	8.02E-13
LOC41547	0.36	0.319	0.574	0.463	0.388	1.031	0.931	0.654	0.981	0.759	1.0481	up	2.13E-08
PEX11A	28.78	25.19	13.222	20.54	14.935	6.614	5.818	3.715	5.104	4.214	-2.01117	down	4.02E-19
MFGE8	138.896	146.087	39.039	75.693	26.068	351.31	344.212	168.314	210.768	249.822	1.637165	up	2.56E-16
HAPLN3	0.659	0.835	0.305	0.786	0.48	1.268	2.357	1.783	1.668	1.039	1.403241	up	1.22E-10
PGPEP1L	6.832	6.269	12.13	11.861	8.377	2.007	2.054	1.364	1.189	1.622	-2.46415	down	2.19E-26
ADAMTS1	1.168	1.212	0.396	1.154	0.274	0.86	4.259	1.343	1.972	1.02	1.16821	up	6.41E-09
ALDH1A3	0.407	0.44	0.452	0.311	0.185	4.13	0.142	2.526	3.317	2.173	2.771768	up	2.37E-37
MTFMT	7.824	6.491	14.997	9.538	12.852	3.92	5.511	4.172	4.503	4.48	-1.19461	down	1.72E-06
ZWILCH	2.149	1.449	4.436	3.396	5.162	7.529	6.786	7.294	7.389	6.94	1.114788	up	5.99E-10
CORO2B	0.557	0.608	0.694	4.147	1.16	0.395	0.942	0.599	0.554	0.396	-1.31061	down	5.20E-08
PAQR5	1.348	1.26	1.339	1.931	1.86	0.598	0.712	0.567	0.523	0.424	-1.4526	down	5.40E-09
KIF23	0.095	0.124	0.14	0.258	0.15	0.287	1.106	0.371	0.486	0.589	1.881249	up	3.11E-17

UACA	7.015	6.751	9.124	5.227	7.991	3.494	2.461	2.757	4.199	3.429	-1.14367	down	3.44E-06
FAM96A	3.886	3.568	3.803	4.241	2.962	1.34	2.471	1.674	1.859	1.282	-1.09719	down	4.69E-06
TMED3	10.988	11.981	10.281	14.372	11.035	58.8	33.928	22.154	41.503	31.871	1.682236	up	8.20E-18
PLA2G15	14.593	15.094	25.23	13.9	19.878	5.57	7.118	8.11	9.565	8.621	-1.18587	down	1.31E-06
ADGRG1	2.077	2.992	2.734	2.146	2.178	1.091	1.167	0.668	1.146	1.504	-1.12022	down	9.55E-06
ENKD1	2.688	2.642	2.644	2.197	2.406	1.067	1.809	1.11	1.556	0.664	-1.01846	down	4.83E-05
CETP	25.686	27.129	125.438	13.017	74.071	0.635	0.238	0.35	2.208	0.236	-6.17516	down	#######
CPNE2	1.128	0.923	3.481	1.064	2.541	0.858	0.73	0.82	1.031	0.745	-1.1259	down	4.63E-05
CX3CL1	8.187	8.904	5.292	11.119	18.702	2.359	2.947	4.222	2.851	2.562	-1.80454	down	9.58E-15
EXOC3L1	1.275	1.501	1.576	3.319	4.536	1.217	1.574	1.103	0.76	1.185	-1.06327	down	1.62E-05
KIAA0895I	3.127	2.669	3.743	2.059	2.17	1.356	1.458	1.279	1.399	1.039	-1.07536	down	3.50E-05
LCAT	92.519	82.067	354.367	283.575	528.767	19.604	14.135	33.27	37.446	40.168	-3.21321	down	4.49E-42
LOC41566	48.739	55.791	52.034	72.387	96.728	4.96	1.713	6.328	8.815	25.298	-2.78909	down	4.00E-33
LOC10174	652.428	670.99	572.533	1307.915	921.024	159.936	32.534	128.319	59.45	468.036	-2.28174	down	1.92E-23
LOC11253	29.432	32.367	17.144	21.458	6.47	16.164	2.917	13.138	2.616	14.967	-1.10152	down	6.00E-06
LOC41566	31.199	32.997	32.033	30.862	30.096	15.188	9.305	17.192	13.281	13.628	-1.19627	down	5.30E-07
LOC10085	673.037	787.092	345.96	334.899	411.752	353.188	88.455	288.255	156.673	336.968	-1.06098	down	7.44E-06
LOC10174	10.563	11.234	12.576	16.408	21.977	5.735	5.577	5.485	9.02	8.043	-1.10341	down	5.59E-06
PDPR	9.716	9.018	13.894	7.72	13.274	4.706	5.047	4.777	4.742	4.971	-1.14509	down	3.10E-06
MLKL	0.054	0.188	0.457	0.029	0.018	0.678	0.591	1.107	0.071	0.355	1.902144	up	7.78E-14
BCAR1	12.196	13.811	19.346	12.079	27.427	6.695	4.627	8.953	5.406	7.79	-1.34203	down	2.81E-08
MT4	711.399	809.728	1038.719	734.886	291.299	7.267	536.949	93.183	11.755	54.038	-2.35039	down	4.99E-26
MT3	310.257	378.472	491.141	215.847	100.131	3.012	298.913	17.352	2.261	12.022	-2.16493	down	3.12E-22
GALR1L	2.159	2.201	1.509	3.049	1.385	0.791	0.672	1.136	0.584	0.663	-1.42046	down	5.18E-10
SLC6A2	2.224	2.327	2.448	1.587	1.529	4.918	4.081	6.646	5.773	3.099	1.276867	up	9.27E-12
TOX3	1.191	0.994	0.419	0.881	0.163	0.173	0.119	0.482	0.158	0.367	-1.48614	down	3.21E-09
SNX20	1.878	1.814	1.478	1.113	1.48	0.572	1.014	0.674	0.824	0.49	-1.11799	down	2.41E-05
CDCA9	20.937	20.591	13.664	15.924	17.513	3.942	2.624	4.166	3.289	3.216	-2.36193	down	1.55E-24
RGS9BP	1.13	1.118	2.26	1.351	2.091	0.571	0.671	0.597	0.501	0.641	-1.41365	down	2.37E-08
FAAP24	6.26	6.445	6.689	4.976	7.044	2.262	1.83	3.326	4.873	3.123	-1.02693	down	3.07E-05
RHPN2	46.278	43.744	61.878	37.868	43.953	17.396	16.613	25.088	30.918	22.462	-1.05512	down	1.88E-05
LOC12111	12.41	12.138	20.236	16.872	27.169	35.953	34.443	44.859	41.423	33.125	1.095422	up	1.00E-09
CEBPA	38.394	43.118	55.499	55.669	106.879	134.743	87.5	162.218	145.012	109.388	1.092649	up	1.14E-09
CEBPG	18.194	17.805	20.768	15.3	20.131	47.529	25.786	44.658	33.016	51.679	1.136268	up	3.55E-10
PDCD2L	9.731	8.954	15.511	6.181	15.106	5.377	4.98	4.752	4.205	5.099	-1.18423	down	2.21E-06
LOC7697C	73.157	72.687	118.776	84.236	242.712	38.68	37.649	47.308	58.168	41.465	-1.40573	down	4.45E-09
CES1L2	38.947	39.648	81.674	126.021	111.344	31.839	28.034	32.323	34.662	26.363	-1.3758	down	4.42E-09
TERB1	3.686	3.169	6.137	3.53	1.611	0.621	1.255	1.176	1.18	1.146	-1.75253	down	6.01E-13

DYNLRB2	2.244	2.109	0.585	1.207	1.131	0.891	0.489	0.45	0.851	0.646	-1.12775 down	8.58E-05
CMC2	1.814	1.737	1.986	2.023	1.73	3.063	4.804	2.572	5.63	2.651	1.010439 up	1.47E-07
CENPN	0.316	0.447	0.405	0.375	0.612	0.816	1.366	0.967	1.306	0.81	1.286772 up	1.03E-09
CDH13	0.334	0.208	0.212	0.419	0.249	0.747	1.36	0.905	0.849	0.623	1.653409 up	1.30E-16
CRISPLD2	0.337	0.417	0.91	0.618	0.785	2.389	1.225	1.38	1.057	1.08	1.215937 up	5.78E-11
SLC7A5	13.905	12.708	34.749	16.312	13.231	4.494	2.959	11.73	4.345	7.995	-1.52781 down	2.69E-10
CA5A	214.2	216.023	174.242	126.055	187.64	21.677	10.907	45.496	14.858	27.178	-2.93427 down	4.22E-37
CIDEC	25.144	27.625	46.895	23.439	53.649	17.667	9.119	17.577	17.536	9.382	-1.31008 down	8.96E-08
MVD	13.917	17.885	4.657	2.51	30.493	34.61	38.568	42.454	42.22	33.021	1.458251 up	2.46E-14
PIEZO1	0.392	0.614	1.099	0.866	0.511	1.059	1.941	2.47	0.967	1.63	1.21094 up	1.13E-10
CDT1	0.437	0.767	1.142	0.632	1.276	1.4	2.28	1.698	1.946	1.616	1.070567 up	1.97E-08
SPIRE2	9.4	10.136	19.47	2.665	10.706	1.581	3.019	2.848	2.049	0.727	-2.35641 down	2.49E-23
CDH3	0.43	0.614	2.023	0.942	1.539	0.341	0.644	0.345	0.285	0.327	-1.51201 down	2.06E-08
LOC12111	0.211	0.11	0.134	0.277	0.477	1.596	0.506	0.279	0.964	0.501	1.665465 up	4.74E-10
CHST4	7.214	6.093	8.923	6.024	4.14	2.379	3.886	4.081	2.811	1.834	-1.11137 down	3.50E-06
LOC1211C	0.174	0.228	15.743	0.613	18.161	0.072	1.006	0.713	1.507	1.182	-2.96104 down	2.41E-30
NT5DC2	16.51	16.917	14.552	32.542	49.533	4.787	10.098	8.763	7.831	6.515	-1.77513 down	8.50E-15
SMIM4	37.41	36.755	18.075	56.563	44.851	10.751	18.349	14.098	14.281	9.666	-1.52806 down	1.70E-11
ITIH3	533.19	482.107	639.242	820.104	691.524	142.604	269.062	220.432	213.384	197.466	-1.60207 down	2.39E-12
MUSTN1	1.16	0.901	1.501	1.907	1.043	0.211	0.326	0.16	0.144	0.191	-2.65179 down	2.49E-12
PFKFB4	2.673	2.431	3.511	2.165	3.002	1.077	1.757	1.301	1.21	1.341	-1.04301 down	2.92E-05
MAPKAPK	15.768	13.765	16.003	8.712	10.04	4.098	8.542	6.033	3.814	6.222	-1.16291 down	1.10E-06
MANF	17.274	19.109	30.432	24.052	29.633	84.998	61.412	33.503	68.067	59.511	1.351475 up	3.05E-13
LOC1211C	5.142	5.183	15.106	2.359	0.336	3.913	19.682	11.064	16.514	8.564	1.086585 up	5.15E-10
LOC1211C	2.474	2.67	6.941	1.457	0.379	0.027	0.802	0.163	0.88	0.122	-2.80043 down	3.81E-27
LOC1211C	0.236	0.288	1.114	0.723	0.129	0.16	0.33	0.104	0.187	0.125	-1.45352 down	2.12E-06
GBP	3.028	3.094	19.819	18.221	3.153	2.927	4.878	4.073	4.058	2.793	-1.33679 down	4.45E-08
GMPPB	15.331	18.192	17.541	15.616	16.166	66.875	68.243	27.75	45.684	34.824	1.554626 up	8.45E-16
LOC7707S	8.973	8.853	14.203	12.134	9.396	4.721	6.259	4.19	3.741	4.617	-1.18658 down	8.81E-07
MST1R	0.552	0.61	1.135	0.754	0.866	3.474	5.51	2.288	3.157	6.588	2.422238 up	4.97E-32
USP4	34.851	31.939	33.217	25.706	30.698	11.885	9.562	9.751	10.088	13.575	-1.5114 down	9.13E-11
GPX1	84.297	82.259	179.925	76.365	144.983	64.968	59.878	39.686	45.716	48.826	-1.13208 down	6.51E-06
PCBP4	1.595	1.723	2.925	3.276	1.419	0.767	0.939	0.942	0.509	0.921	-1.42231 down	8.63E-09
OASL	7.621	9.268	20.363	15.717	5.168	32.377	22.943	41.331	10.893	8.964	1.002838 up	2.11E-08
ABHD6	87.008	72.637	40.937	70.693	36.2	19.581	11.718	19.023	23.964	22.48	-1.66785 down	1.50E-13
IP6K2	70.166	60.534	91.078	45.614	15.293	12.325	17.839	43.372	25.815	13.381	-1.32626 down	1.92E-08
TXNRD3	18.787	18.707	19.534	14.57	28.075	9.563	7.117	9.695	11.082	9.551	-1.08422 down	9.62E-06
CFAP100	0.785	0.514	1.225	0.417	0.571	0.333	0.172	0.18	0.195	0.367	-1.49011 down	5.23E-07

P4HTM	4.254	4.248	4.653	5.244	4.76	1.303	2.415	1.729	1.579	2.719	-1.24841	down	3.53E-07
C3orf67	0.125	0.057	0.03	0	0.038	0.018	0.467	0.11	2.564	0.823	3.966734	up	6.41E-47
PTPRG	50.355	49.553	38.55	32.76	66.168	13.08	19.32	31.356	17.259	18.287	-1.2573	down	7.90E-08
LRIG1	4.02	3.732	5.636	3.626	5.105	2.033	2.093	1.568	1.814	1.618	-1.27677	down	1.30E-07
FOXP1	6.095	5.713	10.718	5.358	7.942	2.81	4.113	3.547	3.049	2.887	-1.12655	down	4.76E-06
GPR27	0.309	0.392	0.087	0.463	0.149	3.915	4.33	1.324	2.708	2.568	3.401821	up	1.38E-45
PROK2	0.259	0.207	0.046	0.118	0	1.925	8.573	1.078	2.028	2.809	4.692378	up	7.34E-63
GHRL	0.524	0.374	1.213	0.782	0.831	0.415	0.499	0	0.284	0.126	-1.48845	down	5.00E-05
HBEGF	30.34	31.798	43.511	22.292	20.809	17.645	8.682	9.025	10.497	15.815	-1.27032	down	2.18E-07
PCDH1	28.233	28.955	33.112	20.709	27.176	10.237	15.842	13.385	11.074	14.711	-1.08251	down	7.38E-06
TENM2	2.348	2.093	5.216	7.734	11.746	0.056	0.045	0.036	0.076	0.069	-6.66591	down	#######
GABRB2	0	0	1.108	0	0.028	0	0.026	0.008	0.003	0	-4.76377	down	8.84E-42
PTTG2	0.163	0.266	0.486	0.201	0.251	0.473	0.609	0.808	1.293	0.322	1.355191	up	1.25E-07
IL12B	2.194	1.867	1.124	2.5	1.549	0.706	1.319	0.5	0.373	1	-1.24315	down	9.33E-08
SLC26A2	5.439	5.176	4.765	3.805	3.095	22.195	13.116	16.658	27.119	13.412	2.053458	up	7.32E-25
PPARGC1I	2.895	2.622	3.296	3.963	3.711	2.091	0.697	1.128	1.407	1.63	-1.24502	down	2.58E-07
ARHGEF37	0.512	0.66	0.698	2.18	1.861	0.585	0.536	0.742	0.421	0.666	-1.00146	down	5.94E-05
DUSP1	33.482	31.109	90.424	20.028	34.609	31.653	15.21	15.262	15.18	19.192	-1.1194	down	1.93E-05
STC2	18.675	18.883	15.306	31.688	29.823	5.736	3.018	5.142	5.316	6.002	-2.18125	down	2.92E-21
SFXN1	25.158	22.442	21.096	19.761	21.703	10.536	11.983	9.378	10.498	12.484	-1.00521	down	3.10E-05
ARL2	1.357	1.882	1.043	1.326	0.505	0.272	20.954	5.16	0.139	1.637	2.202874	up	9.86E-24
MXD3	0.861	1.164	1.964	0.786	1.454	0.028	1.089	0.253	0.19	0.278	-1.7581	down	4.33E-11
NIPAL4	13.302	12.474	17.19	10.635	16.111	4.073	5.875	4.28	8.863	7.28	-1.19858	down	8.74E-07
CYFIP2	8.04	7.819	5.916	6.604	5.009	2.458	2.104	4.499	2.572	1.953	-1.29689	down	2.05E-08
GRIA1	0.327	0.317	0.163	1.681	1.982	0	0.388	0.18	0	0.108	-2.71616	down	2.55E-27
LTC4S	13.217	12.784	9.406	18.945	36.352	3.834	10.065	15.5	4.972	6.608	-1.14618	down	1.52E-06
RASGEF1C	0.176	0.124	0.211	0.266	0.188	0.166	1.032	0.821	0.418	0.655	1.674815	up	2.58E-16
TRPC7	0.315	0.344	1.872	1.381	0.405	0.019	0.118	0.116	0.078	0	-3.68517	down	5.07E-33
LOC10174	4.412	4.148	7.51	4.91	7.225	0.48	1.431	1.494	1.087	0.504	-2.49592	down	3.35E-25
LECT2	0.954	1.249	2.462	8.815	0.51	0.444	0.229	1.348	0.758	0.403	-2.13464	down	1.41E-16
MEIKIN	1.515	1.395	0.998	1.307	0.519	0.327	1.121	0.371	0.39	0.617	-1.01949	down	9.26E-05
SLC22A5	40.76	42.726	34.423	30.492	37.282	6.563	5.192	7.437	4.763	7.521	-2.56033	down	9.92E-29
LEAP2	96.682	98.368	85.891	140.103	52.303	509.645	281.178	291.486	318.676	536.646	2.033312	up	4.25E-24
YIPF5	30.659	28.106	20.453	18.425	20.253	83.872	52.57	49.752	92.515	72.755	1.575821	up	3.12E-16
GFRA3	290.416	277.802	351.34	274.504	381.394	37.282	28.377	30.579	29.531	33.032	-3.31044	down	1.41E-45
CTBPL	47.748	43.473	50.015	34.277	41.846	20.034	19.96	21.331	22.529	22.004	-1.03791	down	2.05E-05
EGR1	81.128	64.473	88.761	3.013	5.115	23.017	22.595	9.537	8.492	16.719	-1.59332	down	1.35E-11
BHLHA15	0.159	0.415	0.505	0.326	0.163	9.579	3.327	1.05	1.497	2.408	3.505626	up	1.39E-35

BAIAP2L1	23.712	23.194	33.089	21.851	33.955	10.48	13.943	11.758	11.949	9.508	-1.23633	down	2.69E-07
NPTX2	0.437	0.313	0.673	0.648	1.722	0.041	0.063	0.186	0.126	0.093	-2.8854	down	5.17E-22
PDGFA	2.159	2.052	4.094	1.986	2.271	0.299	1.001	1.815	1.226	1.222	-1.17441	down	4.19E-05
SUN1	20.199	18.727	24.003	20.917	17.977	9.057	9.502	10.463	8.699	9.009	-1.12356	down	2.76E-06
CYP3A5	1487.01	1384.543	900.646	1710.507	882.877	311.087	337.059	698.817	518.987	596.491	-1.3702	down	1.58E-09
FOXK1	7.523	6.795	10.068	6.397	7.552	2.769	3.357	3.239	3.332	2.738	-1.31218	down	3.62E-08
FBXL18	5.826	5.42	5.054	3.805	4.18	1.672	2.095	3.102	1.748	1.932	-1.20257	down	3.46E-07
LOC10174	1.163	1.458	0.216	0.653	0.756	3.27	18.897	3.42	2.001	2.737	2.834871	up	2.06E-36
PEMT	160.853	158.596	118.057	108.361	86.333	341.446	284.273	219.616	276.424	296.962	1.166132	up	3.71E-10
LOC42766	0.308	0.201	0.335	2.208	1.53	0.283	1.455	3.928	3.28	1.923	1.24526	up	8.86E-10
CACNA1H	0.06	0.042	0.158	0.103	0.147	1.716	0.249	0.633	0.533	0.446	2.798121	up	9.82E-37
SOX8	0.807	0.688	2.121	0.341	1.305	0.213	0.183	0.27	0.388	0.387	-1.86491	down	8.08E-10
MEIOB	1.591	1.449	0.294	0.209	0.039	0.204	0.286	0.477	0.392	0.302	-1.10639	down	1.40E-05
NOXO1	14.631	16.587	5.846	5.302	5.513	1.859	10.535	2.505	1.243	2.964	-1.32514	down	2.27E-09
PGP	11.572	11.695	8.662	4.9	13.466	20.483	12.38	28.864	27.721	18.961	1.107921	up	1.20E-09
EEF2K	24.731	23.253	33.177	17.948	14.461	8.14	10.077	11.192	9.403	10.644	-1.19928	down	6.16E-07
CDR2	0.863	0.893	1.639	0.497	0.67	2.501	2.799	1.859	3.091	2.422	1.472895	up	5.76E-15
IGSF6	4.958	5.297	6.411	4.695	5.123	1.981	2.217	2.873	1.926	2.188	-1.24318	down	3.09E-07
KDELR2	39.185	35.153	50.202	31.225	37.151	87.675	98.138	51.373	86.523	65.163	1.011304	up	1.43E-08
RMI2	16.163	14.381	11.234	5.409	5.134	2.157	2.558	3.271	4.318	1.572	-1.91442	down	6.70E-17
SOCS1	5.662	4.121	7.918	8.084	8.578	18.845	22.849	9.603	8.295	13.734	1.093357	up	3.06E-09
EMP2	1.571	1.827	2.055	2.182	1.972	0.907	0.747	1.073	0.904	0.592	-1.18486	down	2.16E-06
GRIN2A	0.055	0.015	0.03	0.006	0.005	0.004	0.881	1.303	3.566	0.017	5.637874	up	#######
RHBDF1	1.817	1.82	3.071	2.347	1.169	4.686	24.037	3.987	2.77	4.707	1.974243	up	8.08E-23
HBAD	93.174	97.936	309.947	23.94	123.835	15.089	19.212	12.749	17.22	8.256	-3.16119	down	1.32E-39
HBA1	365.186	383.036	1151.922	127.271	387.325	73.897	87.582	62.036	89.701	52.366	-2.72358	down	1.45E-30
PDIA2	0.22	0.352	0.575	0.382	0.314	12.79	1.871	0.216	0.766	0.566	3.133203	up	2.33E-45
FAM234A	40.478	42.628	33.31	40.372	66.442	20.825	16.71	22.469	16.818	15.802	-1.26903	down	6.18E-08
LOC41665	7.203	7.536	6.535	1.798	2.021	0.066	0.011	0.05	0.015	0.09	-6.72654	down	#######
UBALD1	19.961	17.346	30.164	20.536	22.256	7.893	11.186	12.64	12.441	10.822	-1.00385	down	6.39E-05
C16orf96	1.74	1.911	1.353	1.788	1.298	0.784	0.621	0.594	0.432	0.328	-1.55027	down	1.40E-09
NME4	747.074	821.07	1052.988	918.346	1253.88	452.097	453.437	447.796	401.557	533.704	-1.06658	down	1.24E-05
DECR2	121.269	119.623	149.746	120.838	144.37	53.065	50.788	51.724	31.654	56.046	-1.43074	down	1.00E-09
MCRIP2	62.663	56.778	49.221	43.999	43.835	34.649	20.501	22.004	23.537	22.913	-1.05318	down	1.23E-05
ROGDI	7.316	5.888	6.138	4.855	4.172	2.552	2.973	3.055	2.155	2.59	-1.08989	down	9.09E-06
GPRC5B	5.146	4.835	6.084	6.555	6.005	1.737	1.927	2.744	2.712	1.953	-1.36983	down	6.26E-09
SDF2L1	19.198	16.741	25.485	18.423	18.859	62.915	56.723	30.94	59.723	50.352	1.400875	up	7.58E-14
YPEL1	47.695	42.577	36.551	30.211	20.401	11.76	13.958	18.175	14.776	17.89	-1.21259	down	2.06E-07

C22orf39	11.056	13.217	12.622	9.195	14.275	6.655	7.184	3.918	3.484	4.631	-1.22216	down	5.46E-07
CDC45	0.14	0.165	0.367	0.161	0.287	0.508	0.796	0.648	1.18	0.775	1.797981	up	2.30E-16
CLDN5	8.478	13.541	14.928	11.196	19.093	2.606	2.887	8.652	6.44	6.105	-1.33277	down	3.39E-08
COMT	25.212	23.734	31.14	30.333	38.819	7.573	7.1	11.43	10.209	9.408	-1.70661	down	1.91E-13
ULK1	6.747	6.858	6.248	5.161	3.376	6.324	18.629	14.962	10.304	10.472	1.095965	up	4.41E-09
RIMBP2	2.897	2.804	1.685	3.33	2.154	6.265	4.69	7.095	5.43	3.324	1.058145	up	1.73E-08
TMEM132	0.374	0.359	0.091	0.18	0.082	2.356	1.299	1.253	1.499	0.796	2.723948	up	4.30E-35
AACS	7.247	6.549	6.818	7.214	4.705	57.284	29.239	70.049	86.794	53.55	3.189882	up	3.09E-50
SNRNP35	10.437	10.707	10.709	7.557	7.286	2.921	3.987	4.179	3.227	4.351	-1.32273	down	1.97E-08
ATP2A2	15.283	13.796	24.41	14.236	13.583	40.338	31.162	31.416	39.465	48.626	1.232105	up	1.08E-11
RAD9B	1.605	1.591	1.683	1.202	0.539	0.139	0.143	0.07	0.111	0.168	-3.38082	down	2.46E-32
PPTC7	26.451	23.969	28.714	22.315	24.565	7.445	10.752	6.846	7.239	7.171	-1.67525	down	3.33E-13
TCTN1	6.185	6.3	6.677	6.643	8.329	1.884	4.539	3.772	3.234	3.054	-1.05	down	1.44E-05
TMEM116	11.563	10.646	6.492	14.387	17.031	2.741	2.238	4.622	3.145	3.023	-1.93039	down	3.81E-17
UNG	10.68	12.674	7.614	8.913	9.779	0.542	0.93	2.009	1.11	1.585	-3.00632	down	4.38E-34
ACACB	1.373	1.365	0.944	2.486	1.391	0.02	0.014	0.01	0.005	0.037	-6.37714	down	1.10E-94
MVK	13.946	14.6	8.189	6.607	17.499	24.066	11.438	30.807	30.773	25.502	1.010619	up	2.24E-08
FAM222A	1.655	1.592	0.582	0.878	1.87	0.281	0.072	0.319	0.137	0.082	-2.87696	down	7.05E-31
CRYBB3	0.418	0.41	1.289	0.129	0.429	0.228	0.078	0.115	0.104	0.069	-2.16161	down	1.61E-06
VPS29L	2.106	2.448	1.502	1.318	6.329	0.457	0.801	0.139	0.874	0.249	-2.44066	down	7.58E-20
LOC76964	0.785	0.7	0.802	0.736	1.862	0.344	0.25	0.475	0.462	0.394	-1.34123	down	1.43E-07
CHCHD10	310.225	289.108	416.78	254.481	521.347	228.271	151.275	143.77	123.264	161.236	-1.14942	down	2.73E-06
SLC2A11	1.59	1.464	1.87	1.186	2.764	0.641	1.393	0.648	0.571	0.856	-1.10986	down	1.54E-05
SLC2A11L	3.606	3.592	3.219	2.567	6.134	0.468	0.895	1.014	0.662	0.516	-2.42536	down	1.78E-22
LOC76955	17.466	12.831	48.828	14.684	24.095	6.117	3.853	10.657	4.331	3.985	-2.02614	down	2.86E-17
GSTT1L	3.5	3.63	9.075	10.313	21.286	3.043	3.485	3.531	4.748	3.296	-1.40066	down	9.99E-09
TBX6	2.077	2.257	1.313	2.702	4.297	0.971	0.543	0.925	0.7	0.44	-1.81961	down	1.85E-14
LOC41695	1.175	1.185	0.618	1.032	0.658	3.555	4.943	4.52	3.464	3.592	2.103266	up	1.03E-24
PISD	20.194	19.222	12.543	12.178	6.629	67.946	50.359	64.096	65.822	65.567	2.148592	up	2.37E-26
PIK3IP1	19.954	20.004	14.361	16.247	13.432	6.298	6.209	8.127	7.653	7.254	-1.24075	down	1.05E-07
C12orf43	54.569	49.266	46.281	62.024	38.725	17.056	25.497	29.672	17.14	17.919	-1.22544	down	1.01E-07
LOC10705	1.432	1.276	1.73	1.217	2.865	0.826	1.146	0.762	0.613	0.782	-1.04416	down	0.00017
LOC10085	4.583	5.225	3.024	4.197	7.868	2.858	1.079	2.889	0.65	2.766	-1.28106	down	1.02E-06
CFAP73	1.994	2.183	1.377	1.07	0.606	0.829	0.457	0.494	0.748	0.269	-1.36854	down	1.45E-07
SELENOM	15.619	16.483	16.71	13.648	18.306	65.714	54.118	24.489	39.387	32.327	1.419389	up	7.30E-14
PLA2G3	1.791	1.725	5.824	4.58	6.661	8.833	29.941	4.647	5.354	7.707	1.456255	up	2.09E-14
PRODH	0.862	0.852	4.639	2.415	3.652	5.736	10.256	4.698	1.678	5.367	1.158722	up	1.42E-10
SLC35E4	0.209	0.239	0.312	0.204	0.174	8.713	5.883	1.238	1.076	3.238	4.140097	up	1.07E-66

TCN2	106.731	126.53	35.771	38.128	31.915	837.867	536.313	208.465	251.494	307.452	2.658989 up	1.29E-36
HORMAD:	1.423	1.088	1.587	0.976	1.965	0.377	0.328	0.466	0.325	0.268	-1.99346 down	7.21E-17
LOC41701	111.681	109.563	124.638	98.657	150.498	17.363	15.595	27.885	20.788	20.106	-2.54808 down	1.60E-28
MED13L	16.8	14.332	20.78	10.864	12.264	5.087	6.859	7.194	5.673	5.723	-1.29701 down	4.91E-08
LOC10704	0.367	0.261	0.508	1.415	1.097	1.021	3.034	2.041	1.03	1.032	1.160018 up	1.40E-08
LOC11253	0.943	0.745	1.425	1.364	3.358	6.107	3.923	1.902	2.714	8.654	1.571714 up	3.21E-16
LOC1211C	0.534	0.73	2.048	1.755	7.974	0.847	1.381	1.499	0.771	1.025	-1.23878 down	1.64E-05
MHCY2	0.315	0.289	0.251	0.078	0.907	0.344	0.142	0.174	3.603	0.146	1.258466 up	2.15E-10
MHCY14	1.109	0.968	2.202	1.215	0.577	1.399	0.554	0.131	0.118	0.215	-1.32692 down	7.44E-07
LOC1211C	1.131	0.841	0.951	0.317	0.172	0.262	0.058	0.057	0.089	0.034	-2.75837 down	5.14E-21
MHCY9	1.902	1.998	3.085	1.291	0.376	0.584	0.68	0.231	0.537	0.323	-1.87508 down	3.72E-13
LENG9L7	4.913	5.016	7.793	9.996	2.767	0.346	0.178	0.175	0.551	0.366	-4.23338 down	2.50E-58
MHCY15	1.035	0.816	1.204	0.981	0.6	0	0.099	0.117	0	0	-4.39232 down	5.05E-42
MHCY7	1.2	0.974	2.181	1.171	0.861	0.473	0.075	0.276	0.099	0.165	-2.54797 down	1.18E-21
MHCY4	2.728	2.974	3.873	1.116	1.813	1.497	0.547	1.175	1.723	1.082	-1.05298 down	4.10E-05
LOC10085	2.662	2.712	4.583	2.073	3.45	0.369	0	0.14	3.299	0.201	-1.94776 down	3.81E-15
ZNFY1	1.434	1.495	1.681	1.217	0.89	0.275	0.779	0.382	0.36	0.021	-1.88337 down	1.64E-13
OZFL	0.651	0.807	1.588	0.602	1.435	0.316	0.508	0.269	0.31	0.734	-1.24814 down	7.81E-06
ZNFY3	0.19	0.124	0.239	0.221	0.844	0.598	1.585	0.871	0.926	0.876	1.58259 up	2.02E-13
LOC1211C	0.996	0.879	4.17	0.918	0.279	1.01	1.47	0.2	0.3	0.159	-1.20478 down	1.34E-05
LOC1211C	0.866	0.54	1.248	0.39	0.508	0.15	0.062	0.045	0	0.027	-3.62152 down	3.55E-24
LOC1211C	1.782	1.403	1.563	0.448	0.336	0.265	0.054	0	0	0	-4.09504 down	6.87E-27
LOC1211C	10.943	11.057	14.532	7.957	1.516	1.571	2.13	1.032	1.806	1.663	-2.48702 down	5.68E-26
MHCY8	2.228	1.938	2.656	1.732	1.521	0.298	0.162	0.213	0.658	0.429	-2.51376 down	2.15E-23
MHCY6	4.16	4.452	5.831	2.379	5.454	0.781	0.574	0.592	4.36	0.886	-1.63015 down	5.55E-11
MOGL	13.688	10.911	1.441	3.719	2.789	2.257	3.704	2.567	2.263	2.304	-1.31322 down	4.94E-09
LOC1211C	2.086	2.181	22.64	26.21	25.371	0.392	3.146	2.035	0.535	0.393	-3.59272 down	1.10E-50
KIFC1	0.701	0.582	4.305	3.332	4.287	10.828	26.782	18.342	11.197	9.372	2.534101 up	2.82E-35
LOC10705	0	0.065	0.342	0.244	0.475	0.384	1.137	0.51	0.328	0.305	1.2387 up	3.22E-06
ZNF692	1.482	1.501	5.842	5.499	6.48	9.701	15.644	14.34	8.402	9.842	1.477203 up	7.09E-15
IL4I1	0.662	0.6	1.835	0.561	0.701	1.149	9.912	0.753	1.971	1.493	1.808205 up	2.05E-18
TRIM7.1	0.057	0.037	2.341	0.117	0.059	0.042	0.086	0.094	0.014	0	-3.44026 down	3.45E-22
TRIM27.1	0.821	0.682	1.079	0.278	0.455	0.266	0.293	0.23	0.479	0.241	-1.13282 down	0.0001
BG-like-ai	47.812	44.835	79.746	36.209	43.512	16.591	32.753	33.281	11.993	14.009	-1.21466 down	4.30E-07
BLB1	8.097	12.531	19.195	28.102	17.274	34.848	36.859	45.211	30.562	36.756	1.112601 up	1.61E-09
CENPA	0.1	0.263	0.24	1.484	0.309	0	0.301	0.221	0.399	0	-1.37455 down	0.007491
CYP21A1	0.111	0.145	0.044	1.045	0.596	0.04	0.207	0.061	0.165	0	-2.02543 down	2.83E-08
LOC1211C	1.894	1.973	0.277	0.921	0	0.394	0.232	0.256	0.307	0	-2.08618 down	8.71E-12

LOC10704	0.733	0.625	0.711	0.838	1.08	0.278	0.286	0.141	0.506	0.294	-1.40256 down	1.43E-05
LOC10705	0	0.044	0.451	1.396	0.958	1.406	0.549	2.499	2.448	2.507	1.721823 up	1.47E-14
LOC11253	0.695	0.379	0.103	0.085	0.04	0.412	1.832	0.227	0.115	0.034	1.006058 up	2.31E-05
MAN1B1	82.815	78.378	81.445	67.277	71.142	29.165	19.006	25.674	29.88	29.908	-1.5117 down	7.79E-11
EXFABP	4.471	7.057	6.987	22.369	8.956	28.302	115.803	18.003	8.327	9.518	1.85214 up	4.02E-19
STPG3	2.738	3.512	3.672	2.191	2.479	0.629	1.133	0.795	1.109	0.618	-1.76696 down	1.37E-11
NSMF	0.468	0.31	1.023	0.218	1.076	5.713	11.808	6.052	6.083	6.523	3.545012 up	1.65E-60
GSL	1.128	1.641	1.741	1.002	1.773	4.989	4.102	1.98	1.12	2.977	1.057516 up	2.54E-08
LOC10705	75.951	77.659	59.169	58.121	65.695	25.398	30.823	20.69	13.31	16.725	-1.65409 down	3.12E-13
SLC25A25	43.558	44.396	31.78	31.397	45.136	8.669	7.843	5.927	3.958	4.94	-2.64669 down	3.69E-31
ST6GALNA	1.965	2.037	2.057	1.959	2.018	0.835	0.926	1.023	1.031	0.699	-1.15183 down	9.53E-06
FPGS	41.104	41.489	85.376	36.875	85.68	7.738	11.777	13.717	7.223	5.158	-2.67101 down	1.79E-30
CDK9	14.879	15.08	17.502	11.347	19.713	5.865	9.671	7.955	7.084	6.693	-1.07504 down	1.02E-05
TOR2A	10.551	8.878	16.937	11.525	17.075	8.811	4.825	5.699	5.398	6.887	-1.03873 down	4.46E-05
TTC16	2.846	2.249	4.675	3.31	4.541	2.318	1.395	1.461	1.387	1.772	-1.07993 down	3.58E-05
CERCAM	0.422	0.537	0.712	0.848	1.455	0.127	0.279	0.192	0.21	0.279	-1.86543 down	6.48E-12
DNM1	9.543	8.703	6.504	6.356	9.262	4.877	3.283	3.862	2.363	2.174	-1.28534 down	3.90E-08
PHYHD1	7.328	7.702	5.413	8.719	10.513	2.445	2.711	3.507	1.913	3.003	-1.5465 down	2.47E-11
CRAT	191.389	171.33	169.04	167.34	187.563	69.511	71.256	51.719	59.092	73.303	-1.44846 down	3.79E-10
LAMC3	0.196	0.069	0.073	0.098	0.439	0.183	0.073	1.221	0.132	0.712	1.402276 up	6.36E-11
ADAMTS1	21.921	23.567	26.096	30.191	29.099	10.182	15.386	17.435	7.452	10.512	-1.10201 down	3.17E-06
FAM69B	0.345	0.319	0.485	0.751	0.563	9.893	1.247	0.537	0.887	3.188	2.674579 up	1.40E-33
AGPAT2	9.837	11.148	19.814	12.831	6.98	248.358	98.425	66.197	109.974	164.529	3.503588 up	3.01E-59
LOC41711	1.094	1.081	2.958	1.086	1.088	6.034	4.984	5.002	4.719	3.956	1.756173 up	6.77E-20
HSPA5	203.11	183.635	232.322	172.83	247.172	1219.4	708.311	355.638	1083.971	887.601	2.033836 up	4.93E-25
MVB12B	4.325	3.783	5.199	4.809	4.784	17.955	16.428	9.4	13.8	16.138	1.686511 up	2.42E-18
TMEM220	16.139	14.299	14.24	13.163	16.744	4.897	8.566	6.62	5.598	5.086	-1.27736 down	8.24E-08
HS3ST3A1	5.418	5.768	2.626	4.097	4.397	1.667	0.452	0.803	0.584	0.817	-2.36598 down	1.38E-24
FN3K	3.913	3.747	7.322	10.982	14.227	3.272	3.321	2.714	2.918	3.542	-1.34969 down	1.66E-08
NARF	4.369	4.995	11.249	8.829	20.171	3.419	2.891	3.383	5.169	3.781	-1.41184 down	5.58E-09
ST6GALNA	1.081	0.874	0.426	0.423	0.529	0.446	0.156	0.642	0.222	0.185	-1.01128 down	4.36E-05
UBALD2	4.79	4.902	4.134	2.595	2.886	1.674	1.804	2.657	1.967	1.342	-1.03126 down	4.56E-05
CDK3	3.108	2.939	1.859	3.759	2.426	0.336	0.447	0.359	0.121	0.484	-3.00821 down	9.52E-34
TEN1	4.687	5.166	5.237	10.568	7.423	0.774	0.531	0.912	0.685	0.598	-3.23873 down	8.15E-40
ACOX1	304.321	272.828	426.332	464.053	441.56	78.782	65.767	78.942	85.015	82.687	-2.28692 down	1.94E-23
FBF1	14.52	14.705	15.185	12.617	14.558	5.081	3.408	4.812	1.358	4.523	-1.89963 down	1.81E-16
MRPL38	52.316	48.259	56.053	35.782	44.87	19.727	17.731	17.303	17.693	19.665	-1.36497 down	7.64E-09
TRIM65	5.692	5.816	6.734	4.437	5.341	1.619	2.737	2.145	1.361	1.933	-1.51586 down	1.30E-10

TRIM47	3.695	4.001	8.674	2.812	5.102	1.936	1.238	1.842	1.158	2.193	-1.53666	down	3.95E-10
CCDC57	1.045	0.92	0.555	0.711	0.769	2.066	0.909	1.424	2.397	2.402	1.200302	up	8.24E-10
FASN	110.775	119.381	100.058	62.645	146.049	589.898	273.248	670.939	763.68	710.609	2.480862	up	3.00E-34
NOTUM	0.406	0.444	1.542	0.408	0.375	0.236	0.349	0.134	0.161	0.388	-1.32079	down	4.68E-06
MMD	27.091	24.877	49.734	33.386	37.437	14.569	14.328	18.713	19.364	16.545	-1.04658	down	2.85E-05
PCTP	2.597	2.63	1.359	1.574	1.48	0.91	0.653	0.962	0.621	0.876	-1.26008	down	4.97E-07
RGS9	1.299	1.369	1.497	0.838	0.902	0.399	0.559	0.771	0.399	0.368	-1.24065	down	3.25E-07
ABCA8	66.088	65.042	30.608	67.383	91.882	13.031	15.581	35.694	16.12	11.471	-1.80444	down	1.04E-15
SDK2	0.273	0.481	0.456	0.484	0.421	1.621	5.969	2.389	4.135	2.2	2.944416	up	3.78E-42
CBX2	0.516	0.535	0.24	0.106	0.309	3.225	1.805	1.472	1.987	2.159	2.638348	up	4.48E-30
RBFOX3	0.214	0.159	0.327	0.123	0.086	2.469	1.034	0.885	0.621	1.28	2.783711	up	2.31E-35
ENPP7	0.952	1.027	0.061	0.529	0.026	2.959	0.694	0.681	0.741	0.883	1.197527	up	1.69E-07
TIMP2	102.314	120.649	120.318	83.634	148.091	748.968	318.258	283.957	395.058	488.265	1.958298	up	1.47E-23
USP36	3.854	3.259	7.155	3.036	4.335	17.875	9.829	7.963	12.79	12.743	1.499682	up	6.78E-16
SOCS3	3.048	2.755	5.606	4.452	1.599	6.51	60.093	3.532	2.81	6.109	2.178463	up	5.04E-26
LOC10174	2.754	2.926	1.487	2.643	2.202	3.093	13.042	2.406	2.644	2.977	1.007962	up	2.97E-07
TK1	1.116	0.548	2.311	0.917	3.497	5.279	8.696	4.925	8.426	6.263	2.000773	up	8.70E-22
P4HB	266.978	272.602	344.816	341.594	418.659	943.514	453.005	450.36	851.604	662.427	1.03107	up	6.84E-09
EME1	2.304	2.977	4.167	1.075	0.883	0.909	1.112	1.206	1.047	1.005	-1.11072	down	2.66E-05
LOC42210	13.492	13.127	23.518	13.071	13.669	4.013	3.788	3.6	4.246	5.553	-1.85824	down	4.61E-15
KRABZFP	0.342	0.28	0.204	0.421	0.176	0.373	1.024	1.131	0.34	0.79	1.35903	up	0.000102
GRIN2C	1.068	1.271	0.191	0.296	0.839	0.042	0.017	0.063	0.119	0.551	-2.20313	down	1.02E-18
OTOP2	2.11	2.263	1.681	2.181	2.123	2.121	13.918	4.296	2.356	2.24	1.266788	up	1.38E-10
OTOP3	0.434	0.426	0.49	0.694	0.793	1.228	1.21	2.341	1.377	0.987	1.330633	up	4.41E-11
HN1	39.239	38.608	28.761	35.62	42.532	12.133	9.266	9.838	10.308	11.199	-1.80848	down	2.20E-15
SLC16A5	100.899	98.569	67.509	91.142	104.637	16.345	9.886	10.422	8.113	12.544	-3.01328	down	4.61E-39
CASP1	8.277	8.805	8.323	8.909	10.936	23.263	20.295	18.534	18.234	14.895	1.073278	up	5.01E-09
LIMK1	2.35	2.518	4.895	3.684	3.94	10.182	7.611	13.113	9.07	6.187	1.408469	up	6.29E-14
GATSL2	2.567	2.397	5.105	1.018	4.584	0.488	0.831	1.053	0.794	0.528	-2.08335	down	3.38E-17
MIS12	0.314	0.78	0.4	0.774	1.29	2.01	1.317	2.632	3.43	2.735	1.767296	up	8.41E-14
COL26A1	1.246	1.351	0.641	1.001	1.188	0.441	0.186	0.623	0.609	0.536	-1.17845	down	1.76E-06
TMEM120	19.329	20.747	33.045	32.708	21.598	7.144	16.283	8.175	6.533	8.275	-1.45706	down	4.01E-10
MMP28	0.074	0.039	0.71	0.659	0.183	0.972	0.667	1.376	0.31	0.333	1.133178	up	9.43E-08
LOC12110	1.481	1.211	2.801	0.355	3.169	0.898	18.119	0.545	1.41	1.792	1.335552	up	1.63E-10
SLC43A2	16.872	16.964	21.818	30.092	19.542	7.127	13.093	9.491	12.573	9.956	-1.01104	down	2.25E-05
SERPINF1	74.377	64.558	89.8	202.538	74.576	7.706	10.588	7.088	3.23	13.751	-3.57767	down	5.67E-53
SRR	5.107	4.768	4.455	3.419	3.831	1.92	2.364	2.469	1.083	1.439	-1.21783	down	8.73E-07
TMEM199	14.771	17.083	13.655	32.582	31.142	14.173	140.033	12.208	35.611	25.355	1.057662	up	4.16E-08

SEBOX	6.22	6.517	4.5	18.195	17.428	5.959	84.308	3.94	20.462	13.415	1.276762 up	1.02E-10
SARM1	4.158	4.11	1.827	8.341	3.139	3.164	76.002	4.134	2.717	4.524	2.068956 up	1.49E-22
DHRS13	17.405	23.36	14.841	18.293	13.772	7.445	9.456	10.3	6.529	6.36	-1.12876 down	1.55E-06
CORO6	0.518	0.397	0.179	0.407	0.901	1.997	1.045	0.596	1.162	1.664	1.426307 up	2.99E-11
TUSC5	0.404	0.396	0.868	0.473	1.275	0.191	0.045	0.045	0.06	0.067	-3.0502 down	2.50E-25
PIGL	3.194	2.85	2.576	3.648	2.578	0.754	1.305	1.739	1.607	1.889	-1.02479 down	4.64E-05
SHPK	43.598	39.798	57.792	46.978	39.871	13.567	36.992	20.949	17.181	18.081	-1.09473 down	4.29E-06
TRPV1	3.903	3.058	4.633	3.998	3.328	0.856	2.607	1.7	1.342	1.291	-1.27856 down	7.17E-08
ALDH3A2	214.585	215.271	241.807	224.643	354.676	111.33	89.293	159.618	121.431	125.262	-1.04344 down	1.93E-05
DOC2B	0.011	0	0.009	0	0	1.649	2.003	0.375	0.214	0.942	7.697107 up	2.29E-83
MED13	24.129	20.91	40.145	20.433	24.737	4.655	12.565	10.843	5.283	5.566	-1.74402 down	5.03E-14
ACACA	39.426	40.517	32.023	29.127	40.439	134.914	85.318	138.859	146.245	139.834	1.829427 up	8.63E-21
C17orf78	0.122	0.175	0.135	0.12	0.374	0.495	0.146	0.483	0.459	1.155	1.558902 up	5.96E-10
TADA2A	2.004	1.77	1.433	1.767	3.035	4.469	2.98	4.066	4.867	4.441	1.056506 up	1.15E-08
DUSP14	0.586	0.629	0.73	0.309	0.787	2.12	1.282	1.359	1.596	2.121	1.477659 up	1.46E-13
CLUH	25.099	24.566	25.932	21.027	30.204	11.615	7.672	13.765	12.69	13.233	-1.10463 down	5.21E-06
MAP1LC3/	63.198	61.985	105.722	71.471	115.107	30.35	60.937	45.386	30.559	32.582	-1.06304 down	1.39E-05
ASIP	0.562	0.735	1.598	0.198	0.165	0.35	0.12	0	0.08	0.106	-2.30348 down	1.70E-06
E2F1	4.03	4.223	6.779	5.061	4.318	13.758	7.109	9.506	10.466	9.603	1.046942 up	5.46E-09
RBPJL	0.057	0.025	0.045	0.046	0.039	0.219	11.004	0.291	0.056	0.199	5.761766 up	8.33E-78
PI3	9.954	13.416	15.077	52.646	13.145	20.472	3254.778	33.517	10.109	50.09	5.014286 up	1.39E-95
LOC1211C	8.342	7.655	10.199	6.202	3.307	2.166	1.971	3.131	3.301	4.204	-1.27288 down	3.41E-07
EPB42	0.861	1.048	2.935	0.467	0.938	0.226	0.129	0.076	0.205	0.091	-3.09486 down	8.35E-26
GATA5	9.765	8.934	4.151	9.352	21.945	1.789	1.842	3.118	3.737	1.308	-2.19835 down	2.20E-21
SLC17A9	6.602	6.125	11.001	8.875	6.182	19.15	35.512	13.969	16.846	15.045	1.373826 up	3.72E-13
TNFRSF6B	0.199	0.104	0.175	0.049	0.02	0.116	1.823	0.22	0.436	0.553	2.513985 up	3.52E-20
STK35	0.575	0.597	1.312	0.41	1.007	0.32	0.43	0.392	0.428	0.324	-1.04045 down	0.000213
SDCBP2	1.478	1.624	2.398	1.742	2.292	0.812	1.044	1.477	0.526	0.552	-1.1111 down	3.59E-05
LOC77197	0.556	0.685	0.352	0.43	0.017	1.452	1.887	1.299	0.683	1.296	1.695166 up	2.32E-14
WFDC2	5.532	6.109	0.358	1.952	0	26.28	30.865	9.101	12.95	23.734	2.882776 up	2.40E-40
WFDC8	75.251	79.538	7.492	29.801	0.154	283.791	499.134	177.227	156.408	238.391	2.817258 up	5.73E-39
SPINT4	2.528	2.408	0.318	0.474	0	6.905	7.508	2.055	4.185	2.928	2.040569 up	1.26E-19
LOC7719S	0.826	0.42	0.122	0.151	0.157	1.469	2.704	1.147	1.276	2.1	2.371861 up	3.46E-22
UBE2U	0.241	0.158	0.346	0.516	0.843	1.054	2.677	2.663	2.158	0.892	2.163603 up	7.13E-18
RBM38	1.867	1.777	4.031	1.699	2.14	0.516	0.462	0.545	1.196	0.57	-1.8061 down	1.59E-12
GCNT7	10.093	9.912	13.793	9.201	10.606	3.756	10.507	3.365	3.458	5.499	-1.01162 down	2.92E-05
PTPN1	12.27	12.121	23.011	6.668	12.016	5.466	5.714	7.926	6.725	6.147	-1.04715 down	4.14E-05
ESPN	0.409	0.47	0.315	0.23	0.162	1.777	2.313	0.539	0.235	1.934	2.096237 up	5.11E-23

ACOT7	0.806	0.538	3.143	1.19	1.926	0.814	0.616	0.689	0.703	0.456	-1.2125	down	1.65E-05
MMP23A	1.292	1.102	1.579	1.297	5.424	0.554	0.865	0.67	0.574	0.637	-1.69475	down	1.85E-12
MIB2	37.939	37.536	44.728	17.633	58.931	10.917	5.984	10.307	13.207	11.146	-1.93203	down	1.33E-16
LOC41940	8.82	8.759	5.918	5.218	47.946	1.24	0.868	0.82	0.966	0.866	-4.00804	down	4.04E-60
FNDC10	3.834	3.489	2.521	2.027	6.311	2.208	1.347	1.24	1.792	1.22	-1.21914	down	4.60E-07
LOC77106	34.474	35.519	33.965	35.915	49.009	7.788	1.193	4.093	4.38	6.694	-2.96725	down	3.32E-37
PUSL1	4.524	4.485	5.459	5.175	2.754	2.377	3.096	1.602	2.219	1.327	-1.07603	down	9.35E-06
SLC25A33	27.624	24.697	21.936	16.748	18.365	4.788	14.253	9.454	3.978	5.724	-1.51756	down	3.33E-11
TMEM201	7.806	6.809	12.526	8.249	8.709	2.83	3.694	4.027	3.41	4.367	-1.26647	down	1.58E-07
RBP7	0.105	0.445	1.376	1.506	0.645	0.076	0.157	0.347	0.156	0.276	-2.00496	down	4.89E-08
RNF186	11.188	11.918	0.265	5.554	0.043	26.899	3.707	6.836	9.006	22.868	1.258583	up	2.05E-10
DHRS3	40.916	41.077	33.891	44.079	38.887	12.33	22.409	19.424	14.481	12.977	-1.28462	down	2.13E-08
VPS13D	12.151	11.979	19.294	11.279	18.837	6.338	7.674	7.668	7.373	5.923	-1.07206	down	1.40E-05
MTHFR	13.337	12.16	30.133	12.285	4.395	23.598	32.887	32.728	23.689	40.523	1.085214	up	6.94E-10
C1orf167	0.864	0.686	0.831	0.8	0.699	2.241	1.674	1.512	1.332	2.223	1.209925	up	1.19E-10
DRAXIN	0.724	0.636	0.465	0.337	0.311	2.248	1.02	1.335	0.698	3.48	1.82603	up	1.04E-18
EPHB2	3.285	3.747	3.113	2.948	5.027	9.683	3.673	6.641	9.703	8.248	1.066233	up	3.88E-09
P3H1	8.357	8.893	9.095	6.283	8.827	27.159	15.335	13.244	20.95	17.463	1.183333	up	1.19E-10
ECE1	39.205	38.167	53.402	47.779	35.696	18.137	19.46	22.59	14.945	17.369	-1.2117	down	3.23E-07
LOC11253	13.755	12.357	22.05	16.679	10.052	5.474	7.669	6.474	4.558	5.188	-1.35068	down	1.17E-08
SLC23A2	7.856	7.871	14.955	8.454	12.955	4.789	4.93	4.606	5.182	4.511	-1.11676	down	6.97E-06
DPYSL2	1.602	1.773	1.878	1.072	1.27	7.828	3.544	9.066	5.276	7.949	2.147307	up	7.88E-27
SLC25A37	2.889	2.729	5.262	4.998	8.816	1.759	4.341	1.12	1.567	1.17	-1.30995	down	4.12E-08
DMTN	0.926	0.799	1.2	0.544	0.68	0.222	0.335	0.337	0.394	0.188	-1.48793	down	1.81E-08
LOC10174	25.547	25.293	7.821	17.495	25.19	5.7	7.995	11.523	6.823	7.345	-1.36342	down	3.90E-09
ADAM32L	1.354	1.2	1.01	1.38	1.989	0.349	0.282	0.34	0.357	0.452	-1.9586	down	9.67E-14
IDO2	10.394	12.035	5.352	19.063	27.269	2.5	2.069	5.761	4.049	2.424	-2.14068	down	7.82E-21
GPAT4	11.693	10.885	16.073	14.55	14.604	5.752	6.057	7.527	6.748	6.726	-1.04714	down	2.09E-05
ANK1	1.223	1.303	2.635	0.715	1.221	0.355	0.382	0.339	0.473	0.317	-1.92442	down	1.78E-15
ARID5A	2.36	2.292	4.974	1.837	1.388	1.954	20.84	3.091	2.261	2.84	1.269407	up	1.83E-11
MTHFD2	1.543	1.265	4.583	0.984	0.851	3.216	5.829	4.165	2.721	3.526	1.076101	up	2.50E-09
SLC4A5	0.936	0.828	2.248	1.427	1.07	3.065	4.532	4.054	4.529	2.744	1.538983	up	5.06E-16
ADD2	1.232	1.137	1.521	0.989	0.687	0.597	0.572	0.485	0.569	0.315	-1.1314	down	1.16E-05
PCYOX1	69.723	64.571	62.483	51.171	59.549	33.91	23.371	33.35	26.639	32.815	-1.03477	down	1.97E-05
FAM136A	14.819	16.737	21.821	15.24	29.508	6.144	7.442	7.306	9.457	9.836	-1.28786	down	2.49E-07
CASP14	1.235	1.087	1.424	1.278	0.897	0.093	0.064	0	0.021	0.112	-4.32827	down	4.91E-37
RETSAT	181.098	181.873	305.999	306.703	245.919	87.614	41.452	69.409	39.471	63.976	-2.0165	down	2.17E-18
FABP3	13.543	18.173	2.598	3.493	0.203	211.299	48.427	14.83	34.071	77.256	3.343541	up	9.01E-51

SERINC2	1.761	1.695	1.951	0.682	1.747	5.184	3.791	3.216	3.559	2.753	1.239039	up	7.93E-11
LOC1211C	3.245	3.671	0.699	2.366	1.904	0.284	3.033	0.431	0.436	0.483	-1.34764	down	1.08E-08
RHCE	8.15	6.385	11.823	9.087	7.332	2.795	2.916	6.299	2.346	3.906	-1.22776	down	5.67E-07
TRNAU1A	12.629	11.351	6.146	7.127	5.097	2.263	4.328	4.309	2.518	2.687	-1.39458	down	6.89E-10
TRIM63	1.622	1.584	1.214	2.149	1.629	0.352	1.922	0.481	0.385	0.527	-1.15959	down	1.32E-06
STMN1	5.07	4.523	8.573	3.977	4.092	31.618	11.591	13.583	19.817	15.299	1.808501	up	9.89E-21
PAQR7	24.346	28.129	11.665	9.039	4.872	106.511	50.059	57.97	44.506	73.304	2.090144	up	6.09E-25
INPP5B	21.031	19.515	23.347	16.589	19.166	9.411	9.051	9.28	8.52	7.621	-1.18309	down	7.33E-07
IFI27L2	1.02	1.144	1.74	27.689	12.533	41.075	81.962	82.611	91.244	42.844	2.944571	up	2.19E-42
TMEM35B	35.636	43.246	43.684	26.653	25.922	18.647	17.768	16.617	13.343	13.653	-1.12989	down	2.99E-06
FAM167B	18.468	19.179	15.698	20.949	9.892	4.369	6.747	5.249	5.599	5.721	-1.6043	down	2.79E-12
AZIN2	3.941	3.49	3.609	4.982	5.168	1.169	3.429	2.341	1.566	1.2	-1.12618	down	2.35E-06
MYCL	25.236	25.063	17.182	6.684	21.801	5.512	4.924	7.661	5.017	3.579	-1.84587	down	1.15E-15
GALE	203.78	179.069	256.203	94.255	87.192	56.338	31.094	39.589	32.376	20.232	-2.19145	down	2.26E-21
HMGCL	1321.12	1321.949	1041.953	914.18	1355.956	427.151	149.662	328.356	287.771	191.262	-2.10508	down	2.97E-20
GRHL3	0.226	0.247	0.18	0.171	0.184	1.071	0.297	0.416	0.506	1.034	1.716455	up	2.40E-15
IL22RA1	1.048	0.851	0.331	0.475	0.408	4.707	2.41	5.303	1.992	4.008	2.562972	up	3.21E-31
NRGN	0.84	1.062	2.496	1.081	1.437	0.652	0.88	0.556	0.361	0.222	-1.3709	down	1.41E-06
SPA17	5.129	5.559	3.796	3.066	5.694	1.885	2.794	2.172	2.264	2.052	-1.05728	down	5.01E-05
KCNJ5	0.108	0.154	0.25	0.129	0.242	0.657	0.412	0.346	0.439	1.166	1.768304	up	2.82E-16
TMEM45L	0.867	1.134	1.972	3.459	2.645	0.18	0.26	0.382	0.148	0.915	-2.41532	down	2.77E-19
SCN3B	0.701	0.619	1.075	0.612	0.851	2.04	2.974	1.595	2.855	2.401	1.619526	up	3.61E-17
SC5D	74.838	77.584	17.882	25.125	74.554	113.729	87.256	127.655	107.906	104.04	1.001642	up	5.77E-08
GRIK4	0.608	0.373	0.121	0.672	0.273	0.996	1.637	0.608	0.746	0.577	1.154848	up	3.92E-08
POU2AF1	0.549	0.302	1.32	0.361	0.259	3.099	0.493	1.429	0.567	0.391	1.097746	up	2.67E-09
ZBTB32	25.252	27.628	28.602	8.546	14.028	3.019	6.637	5.143	11.153	14.211	-1.37331	down	8.81E-09
APOA5	226.944	249.026	159.85	201.021	232.501	57.848	99.198	118.758	88.819	57.889	-1.33965	down	4.40E-09
FXYD6	1.202	0.866	2.08	1.34	3.3	0.607	1.102	0.947	0.767	0.486	-1.16771	down	7.23E-06
TMPRSS13	0.618	0.535	0.832	1.276	1.996	0.489	0.571	0.26	0.46	0.204	-1.40357	down	3.37E-08
SCN4B	1.577	2.38	1.504	1.283	2.197	0.5	0.398	0.379	0.341	0.701	-1.94463	down	1.43E-14
HYOU1	31.471	30.812	41.361	31.712	27.66	148.51	75.324	49.344	123.82	102.49	1.615407	up	2.24E-17
PLET1	3.093	2.981	3.518	1.77	3.432	0.804	0	0.305	0.274	1.094	-2.57592	down	3.70E-22
BGLAP	2.805	2.936	1.638	3.227	1.825	1.088	0.7	2.269	0.093	0.37	-1.45853	down	4.01E-07
LOC1211C	0	0	0.072	0.374	3.318	1.456	7.668	4.015	5.671	2.462	2.497041	up	4.33E-31
LOC10174	0.02	0.013	0.032	0.083	1.055	0.322	1.388	1.955	3.001	0.372	2.54357	up	2.43E-27
LOC10705	0	0	0.044	0.036	0.484	0.335	0.804	1.208	0.979	0.275	2.663899	up	1.90E-28
LOC42949	0	0	0	0	0	0	1.105	0	0	0	7.794416	up	3.99E-09
LOC10085	0	0	0	0	0	0.074	2.126	0.112	0.403	0	9.087463	up	4.85E-21

LOC42691	0	0	0	0	0	0	3.442	0	0.507	0.112	9.667466	up	6.00E-19
LOC10704	2.808	2.714	2.033	1.224	3.365	0.836	1.115	0.516	1.141	0.59	-1.53135	down	1.47E-09
SHE	8.942	10.176	8.832	9.624	11.635	2.995	3.192	8.92	3.654	4.207	-1.09913	down	4.90E-06
CKS1B	1.373	1.796	3.682	5.166	4.156	10.829	7.361	9.405	8.612	7.535	1.435151	up	1.05E-12
FDPS	41.825	42.11	7.962	5.635	47.361	154.846	56.025	162.615	192.107	155.799	2.315756	up	5.57E-30
SYT11	0.614	0.468	2.816	2.184	2.532	0.507	0.618	0.788	0.655	0.508	-1.48412	down	4.95E-09
GOLPH3L	1.159	1.435	1.649	0.385	0.582	0.359	0.48	0.381	0.392	0.586	-1.2432	down	1.91E-05
LOC1211C	0.115	0.075	0.214	0	0.118	0.112	0	0.508	1.067	0.203	1.846323	up	1.02E-08
PCP4L1	2.075	1.357	5.083	3.804	18.36	2.554	0	2.113	2.219	1.896	-1.80404	down	3.77E-09
APOA2	1836.68	2018.685	3427.281	2772.508	4123.305	773.666	1447.882	1051.99	835.568	973.913	-1.47994	down	2.58E-10
TMOD4	0.723	1.269	1.885	0.999	1.756	0.478	0.492	0.58	0.359	0.347	-1.55357	down	9.94E-08
LOC1211C	1.377	2.274	3.499	15.089	1.684	18.797	11.396	6.655	8.989	14.89	1.343757	up	3.86E-11
THEM4	1.379	1.504	1.63	1.751	3.047	0.365	0.376	0.738	0.208	0.441	-2.12683	down	2.14E-13
PFDN2	11.692	13.549	20.304	0.767	14.309	0.543	9.13	4.665	6.857	0.164	-1.50475	down	1.17E-09
TULP1	0.396	0.339	3.696	0.217	0.174	0.069	0.057	0.021	0.094	0.025	-4.15476	down	2.99E-41
FKBP5	111.755	99.425	223.982	47.184	74.273	23.682	11.499	19.264	25.978	37.382	-2.24024	down	2.58E-21
LHFPL5	0.335	0.709	1.11	0.573	0.928	0.526	0.077	0.455	0.179	0.511	-1.06202	down	0.00102
ELF3	1.802	1.886	5.136	1.871	1.286	8.391	19.21	10.127	7.216	5.259	2.066567	up	2.83E-25
CSRP1	1.668	1.898	3.368	2.702	4.32	25.746	19.446	3.733	3.121	15.891	2.282901	up	1.66E-29
CEPT1	131.831	117.218	135.056	88.154	103.395	26.219	30.618	50.856	23.912	44.189	-1.71129	down	1.22E-13
PI16	2.037	1.634	2.846	1.917	0.557	1.913	0.148	0.399	0.155	0.434	-1.55858	down	3.02E-10
REN	2.055	1.905	4.734	0.853	0.88	0.44	1.6	0.756	1.131	0.732	-1.16138	down	8.65E-06
LOC1211C	2.336	2.667	13.532	2.99	5.007	2.704	2.166	0.491	1.163	1.088	-1.80071	down	2.96E-13
SLC45A3	1.655	1.516	4.072	1.441	3.063	0.653	0.573	0.617	0.548	0.447	-2.04742	down	1.28E-17
SLC26A9	0.066	0.086	0.01	0.003	0	8.813	2.088	0.814	1.502	5.503	6.783287	up	#######
MAPKAPK	60.878	52.263	54.944	27.143	55.236	24.291	26	24.543	19.564	24.952	-1.06937	down	1.09E-05
C1orf116	34.199	36.157	48.71	39.146	50.886	15.002	22.439	18.493	14.232	18.246	-1.24182	down	1.87E-07
PLXNA2	1.893	1.903	2.659	1.743	2.081	0.879	1.734	1.265	0.624	0.547	-1.0249	down	2.60E-05
CAMK1G	0.091	0.094	0.055	0.136	0.055	1.111	0.832	0.46	1.337	0.888	3.409547	up	3.51E-47
LAMB3	0.296	0.278	0.164	0.519	0.172	3.308	2.016	1.648	4.42	2.746	3.301971	up	5.93E-49
G0S2	17.283	17.63	33.545	71.809	20.964	365.138	200.748	243.328	437.686	173.969	3.139533	up	1.41E-48
MOV10	20.693	22.727	15.294	15.974	9.551	37.94	41.272	40.901	30.533	34.68	1.137458	up	1.38E-09
SLC16A1	80.737	81.864	85.098	65.917	80.697	6.935	8.302	11.161	11.565	8.783	-3.07629	down	2.88E-40
LOC41988	20.543	18.647	21.886	12.32	13.109	3.788	3.048	6.47	3.998	8.414	-1.74981	down	7.42E-14
SYCP1	5.077	4.565	9.032	3.579	2.613	0.792	0.782	0.98	0.717	1.804	-2.29156	down	1.68E-21
TSHB	2.692	2.656	5.24	1.229	0.966	0.311	0.256	0.283	0.467	0.903	-2.52291	down	2.91E-18
PACSIN1	3.057	2.979	2.103	5.131	1.149	14.404	5.632	6.285	6.538	9.943	1.569375	up	2.06E-15
ITPR3	1.748	2.077	1.917	1.958	2.4	5.251	3.012	4.16	3.912	4.009	1.009889	up	2.25E-08

TSPO2	1.038	0.781	1.984	0.782	0.498	0.176	0.285	0.115	0.275	0.137	-2.35723	down	1.26E-16
APOBEC2	2.557	1.79	4.867	3.089	2.416	1.6	0.919	0.903	1.575	1.452	-1.1899	down	9.48E-06
CHIA-M31	0.124	0.809	0.066	1.151	1.037	0.33	1.728	2.954	2.005	5.983	2.026534	up	1.78E-20
CHIA	323.094	320.506	459.022	400.362	363.803	1092.784	1274.999	1202.371	671.778	1673.335	1.663883	up	4.50E-18
LOC76878	0.737	0.599	0.428	1.424	0.491	4.058	3.671	3.735	2.394	5.943	2.426593	up	1.44E-28
LOC10705	1.478	1.284	2.353	0.381	0.126	0.126	0.629	0	0.108	0.01	-2.68007	down	1.50E-26
LOC10704	1.069	1.001	1.257	0.426	0.349	0.141	0.461	0.125	0.217	0.128	-1.93107	down	3.02E-14
VWA5A1	3.4	3.36	1.917	2.377	3.428	1.069	1.288	0.931	0.807	0.867	-1.54431	down	3.36E-11
DAD1	56.847	61.622	57.988	57.821	49.911	151.483	117.374	68.729	128.398	114.676	1.03083	up	1.46E-08
PLCD3	2.246	2.13	1.433	2.418	1.519	0.695	1.231	0.984	0.738	0.888	-1.10254	down	3.39E-06
LOC12110	1.956	1.825	3.115	1.667	2.567	0.738	1.628	1.279	0.792	0.574	-1.15049	down	2.75E-05
MRC2	0.907	1.665	5.262	2.221	1.584	4.654	2.853	4.375	10.959	12.132	1.586861	up	6.63E-18
SLC4A1	2.92	2.766	7.813	0.831	2.847	0.239	0.616	0.454	0.694	0.423	-2.82128	down	2.19E-30
LOC10705	8.601	8.62	6.678	6.99	10.082	1.837	3.177	4.468	2.84	2.925	-1.42578	down	1.57E-09
SLC35B1	36.478	35.208	36.24	31.353	29.908	119.924	78.34	48.855	97.016	79.003	1.322484	up	1.59E-12
C27H17OI	11.499	8.589	25.485	14.803	13.014	5.849	11.434	4.603	3.689	4.702	-1.27722	down	2.55E-07
PHOSPHC	25.754	25.461	25.759	24.48	19.312	6.95	10.197	8.519	6.827	8.25	-1.56747	down	9.04E-12
GNGT2	8.693	8.45	12.954	10.103	8.589	2.831	3.659	7.58	2.755	2.459	-1.33893	down	3.72E-08
CACNB1	3.613	3.616	3.693	3.325	3.512	1.393	2.185	2.439	1.256	1.249	-1.05885	down	1.34E-05
PPP1R1B	2.268	2.589	3.554	0.991	4.04	1.018	0.94	1.171	1.007	0.733	-1.46411	down	1.88E-08
RARA	10.973	12.41	15.375	11.101	11.083	45.924	35.188	15.275	11.211	25.765	1.129785	up	6.69E-10
TOP2A	0.342	0.255	0.321	0.388	0.281	1.075	0.932	1.328	1.515	1.418	1.978315	up	2.16E-21
KRT40	0.232	0.182	0.664	0.476	0.286	0.843	1.146	0.716	0.76	1.315	1.374898	up	3.69E-10
KRT24	1.121	0.885	1.169	0.46	1.964	1.912	4.573	1.534	2.149	2.321	1.156707	up	1.83E-09
KRT10	0.362	0.403	0.663	0.461	0.465	0.896	2.062	1.718	1.493	1.363	1.675815	up	3.38E-15
P3H4	4.043	4.802	2.653	3.765	2.923	1.313	0.65	1.685	0.793	0.962	-1.75006	down	1.73E-13
FKBP10	5.547	5.356	3.977	5.328	3.06	1.404	1.913	1.972	0.986	2.098	-1.47398	down	2.64E-10
G6PC	546.432	558.921	287.335	471.538	539.08	141.559	97.648	146.715	97.975	115.146	-2.00428	down	6.08E-19
VAT1	115.805	120.68	33.306	87.503	40.757	32.929	16.026	22.408	23.92	24.247	-1.73554	down	6.03E-15
RND2	2.62	1.917	1.709	1.848	1.823	1.441	0.958	0.683	0.697	0.518	-1.20562	down	2.44E-06
NBR1	72.985	64.219	68.511	54.496	56.89	20.494	41.267	33.928	26.075	24.037	-1.12092	down	1.95E-06
SLC1A6	0.116	0.21	0.171	0.125	0.211	0.442	1.686	0.197	0.151	0.766	1.954085	up	4.02E-18
LOC10175	3.827	4.542	4.63	3.06	0.899	4.382	8.956	6.774	7.573	7.52	1.053594	up	1.40E-08
ANGPTL4	12.453	12.116	22.343	19.188	6.518	108.426	62.564	240.185	172.065	41.414	3.10457	up	1.69E-48
C19orf71	0.377	0.308	0.038	0.039	1.017	0	0.071	0.312	0.094	0	-1.88801	down	1.49E-05
LOC10085	0.669	0.945	2.285	1.243	0.617	3.441	2.846	1.454	1.974	2.354	1.066761	up	3.14E-09
MPND	7.891	7.569	12.217	7.376	6.539	3.82	5.016	4.578	2.998	3.881	-1.03514	down	3.29E-05
ACSBG2	14.586	15.643	11.837	8.17	13.21	205.332	228.641	377.474	351.118	237.925	4.46415	up	5.87E-84

ONECUT3	1.369	1.143	1.524	0.913	1.264	0.584	0.725	0.631	0.586	0.511	-1.03143	down	4.16E-05
MEX3D	0.596	0.738	0.777	0.636	0.69	2.159	1.52	1.203	1.096	1.573	1.134376	up	5.10E-09
LRG1	0.422	0.461	0.359	0.44	0.984	0.984	2.532	0.497	0.755	1.301	1.185267	up	5.46E-08
CREB3L3	159.827	156.871	111.394	114.31	126.707	24.907	38.736	41.103	24.78	28.141	-2.08533	down	2.51E-20
ARID3A	2.477	2.706	4.511	3.699	4.502	13.288	8.293	7.199	7.017	6.042	1.225061	up	2.69E-11
MIDN	1.534	1.929	5.572	2.288	1.636	8.601	11.376	4.632	4.851	7.725	1.520404	up	5.56E-16
LOC11253	0.265	0.267	0.78	0.285	0.231	1.129	2.079	0.63	0.507	0.377	1.366718	up	3.53E-10
GAMT	161.068	171.338	127.697	148.859	189.083	85.711	72.837	61.209	83.203	84.928	-1.04082	down	1.37E-05
ADAMTSL	0.983	1.337	0.423	0.162	2.564	0.114	0.088	0.13	0.059	0.259	-3.06303	down	2.55E-24
LOC10085	1.85	1.341	1.179	1.31	0.877	0.331	0.512	0.188	0.368	0.601	-1.71053	down	1.03E-09
OCLN	4.884	6.494	8.754	6.416	8.865	3.21	3.147	4.403	3.391	3.351	-1.01655	down	5.70E-05
USHBP1	1.913	1.846	2.234	1.218	1.441	0.533	1.146	0.668	0.697	0.737	-1.19319	down	3.69E-06
COMP	1.53	1.326	1.717	1.658	0.99	0.419	0.64	1.644	0.276	0.354	-1.11421	down	5.70E-06
CRTC1	7.726	7.552	11.878	8.518	9.137	4.276	5.001	4.871	3.855	4.127	-1.01768	down	3.78E-05
LOC42579	6.757	7.471	7.946	5.609	7.261	2.73	3.768	3.599	2.998	4.379	-1.00375	down	5.26E-05
ARHGEF18	13.662	14.162	13.07	13.147	16.547	4.004	4.869	6.178	4.77	4.635	-1.52904	down	3.43E-11
LOC12110	0.686	0.658	0.66	1.216	0.474	3.513	7.092	2.533	3.08	3.468	2.412329	up	1.70E-29
LPAR2	8.601	8.413	3.964	5.818	5.397	0.724	5.469	0.976	3.626	3.797	-1.1413	down	1.32E-06
FBXL12	6.02	5.866	7.379	3.51	2.899	10.249	13.845	13.391	9.229	9.958	1.142174	up	7.44E-10
ACP5	15.183	16.497	42.327	27.342	39.833	11.939	12.248	9.051	9.34	9.014	-1.45225	down	1.91E-09
CALR	241.781	241.895	342.532	279.632	306.725	1162.188	747.975	442.993	867.762	759.456	1.494583	up	2.07E-15
LOC12110	0	0	0.013	0	0	0.023	1.905	0	0	0	6.746701	up	2.30E-37
LOC12110	0.277	0.127	0.11	0	0.171	0.081	1.079	0.367	0.138	0.073	1.336904	up	1.77E-05
LOC10704	0.236	0.385	0.094	0.075	0.255	0.057	7.018	0.087	0.208	0.207	2.852189	up	2.40E-32
CD163	11.149	13.447	5.876	14.179	56.183	36.076	33.345	46.145	52.384	47.608	1.096056	up	1.25E-09
LOC10175	1.051	0.647	0.043	0.034	0.021	2.691	2.412	2.153	1.2	1.261	2.432455	up	1.08E-28
LOC10704	0.009	0.006	0.5	1.416	0.047	0.013	0.014	0	0.009	0.006	-5.39888	down	3.48E-56
SLC27A2	142.476	149.833	167.679	140.63	245.202	89.508	63.515	95.107	80.836	85.605	-1.02872	down	2.92E-05
LOC10705	0	0.054	2.65	1.966	1.017	0.32	0.165	0.667	0.437	0.254	-1.62297	down	4.31E-08
LOC11253	0.229	0.083	1.827	0.796	0.655	0.297	0.191	0.619	0.101	0.067	-1.48985	down	2.35E-06
LOC12110	0.207	0.406	0.823	0.085	1.31	0.15	0.103	0.228	0.137	0.091	-1.98986	down	1.67E-07
CHIR-AB1	1.081	1.29	0.253	0.444	0.163	0	0	0.07	0.189	0.042	-3.40261	down	2.74E-18
LOC10704	0.688	0.922	0.109	1.526	0.318	0.05	0.052	0	0.034	0.091	-3.94292	down	9.59E-22
LOC12110	0.45	0.741	0.532	1.811	0.606	0.858	1.628	2.98	4.811	3.504	1.73376	up	8.03E-17
LOC12110	0.064	0.063	0.305	1.494	1.179	0.093	0	0.141	0.158	0.337	-2.08306	down	2.33E-09
LOC10704	0.052	0	0.416	0.408	0.536	0.38	0.9	1.267	0.156	0.276	1.074408	up	1.61E-05
LOC12110	0.464	0.203	4.252	0.509	0.636	0.394	0.174	0.854	0.423	0.306	-1.4931	down	5.72E-06
LOC12110	0.033	0.109	1.94	0.247	1.2	0.146	0.6	0.294	0.133	0.176	-1.38407	down	8.13E-05

LOC11253	0.417	0.26	6.651	2.071	3.179	0.144	0.476	0.306	0.197	0.314	-3.12533 down	6.83E-30
LOC11253	1.699	2.017	4.442	3.064	3.784	0.919	1.059	2.023	0.642	0.834	-1.45325 down	5.06E-09
LOC10704	0.451	0.279	1.077	0.453	0.875	0.219	0.113	0.221	0.199	0.364	-1.48598 down	9.37E-06
CHIR-AB-	0.641	0.668	0.557	0.455	0.509	0.127	3.8	3.088	0.319	0.154	1.402195 up	2.29E-09
CHIR-B3	3.536	3.698	0.456	0.184	0.537	0.109	0.149	0.055	0.074	0.099	-4.09934 down	2.47E-39
LOC11253	0.05	0.105	0.97	0.55	1.303	0.015	0.404	0.22	0.129	0.145	-1.7002 down	1.13E-09
LOC1211C	0.944	1.306	1.994	0.686	4.846	0.106	1.522	0.961	0.36	0.431	-1.53083 down	3.12E-08
LOC10705	0	0	0	0	0	0	0	0	2.883	0	9.173927 up	3.08E-33
FKBP11	17.495	22.133	21.819	27.326	17.85	78.643	64.221	32.462	72.211	53.302	1.496429 up	8.41E-15
METTL7A	142.141	140.895	176.986	159.581	210.518	61.529	69.419	79.211	48.829	47.316	-1.43834 down	6.76E-10
TMPRSS12	1.529	1.699	1.16	1.257	2.294	0.424	0.291	0.643	0.651	0.417	-1.70832 down	1.81E-10
COPZ1	17.724	18.797	15.632	14.99	13.592	39.551	44.413	21.568	29.998	26.073	1.001143 up	5.39E-08
TMEM106	1.663	2.036	3.802	2.807	7.495	1.665	2.465	1.42	1.421	1.393	-1.0894 down	2.65E-05
TUBA1A	29.193	32.818	32.372	27.198	37.85	96.707	82.919	64.025	43.838	54.906	1.102705 up	1.63E-09
SLC39A5	38.854	41.23	11.778	22.628	19.009	70.656	60.575	59.751	44.61	55.899	1.126593 up	3.54E-09
CNPY2	21.558	28.126	25.31	18.054	20.555	78.674	68.141	37.674	54.708	49.586	1.345946 up	9.24E-13
STAT2	5.504	5.392	6.718	5.234	3.53	13.084	14.884	11.6	9.483	9.926	1.160666 up	4.04E-10
APOF	37.366	50.766	36.826	63.533	23.793	212.892	228.328	173.722	140.081	137.663	2.072131 up	1.76E-24
RDH16	79.427	82.981	118.563	90.739	178.048	67.785	54.445	58.373	42.309	35.133	-1.09116 down	8.57E-06
TAC3	0.091	0.208	1.231	0.99	1.378	0	0.068	0.05	0.023	0.12	-3.87509 down	6.05E-26
INHBE	4.017	4.779	3.518	12.377	2.036	25.28	33.35	26.628	19.053	32.28	2.353276 up	1.74E-29
GLI1	1.446	1.44	1.145	2.773	0.783	2.828	3.308	3.585	2.996	2.515	1.005027 up	1.46E-07
POU6F1	0.897	0.795	2.112	0.587	0.44	0.347	0.69	0.28	0.11	0.419	-1.38551 down	1.08E-07
LOC1211C	0.594	1.033	0.032	0	0	0.098	0.222	0	0.027	0	-2.24101 down	1.06E-13
LOC10705	10.202	5.451	99.121	66.896	4.471	5.479	49.132	18.324	6.234	6.734	-1.11557 down	6.10E-06
VKORC1	7.719	7.772	7.827	8.078	7.185	24.217	10.389	25.411	20.245	16.081	1.320177 up	5.34E-12
LOC1211C	0.377	0.123	12.832	10.917	8.412	3.569	3.053	1.915	2.886	0.249	-1.48412 down	7.15E-09
LOC1211C	0.186	0.169	0.628	0.801	0.736	3.922	4.639	1.234	2.791	0.303	2.352345 up	6.17E-28
LOC1211C	0.891	0.858	0.65	1.728	0.254	5.617	2.891	2.293	3.023	1.895	1.841991 up	1.13E-17
SYCN	0.239	0.372	0.965	0.211	0.434	1.816	1.237	0.567	0.638	0.606	1.129172 up	1.27E-08
OTX5	3.385	3.379	6.24	1.429	2.499	0.316	0.853	0.685	0.535	0.164	-2.72709 down	4.43E-29
LOC10705	5.249	4.591	10.795	2.095	3.419	0.567	1.303	1.059	0.983	0.277	-2.64063 down	2.98E-25
LOC42622	85.864	97.813	72.44	292.66	10.808	554.449	433.194	182.108	130.083	551.11	1.725823 up	9.31E-18
LOC1211C	2.846	2.578	2.049	1.574	2.642	0.319	1.066	0.483	0.924	0.795	-1.70291 down	2.41E-09
LOC1211C	0.063	0.064	0.521	0.104	0.253	1.883	7.155	4.035	0.964	0.557	3.853442 up	1.41E-63
LOC1211C	0.315	0.103	0.785	0.292	0.405	0.631	1.063	1.044	0.783	0.312	1.010564 up	1.87E-05
LOC1211C	4.879	5.287	3.648	5.295	2.334	0.975	1.562	2.301	2.22	2.95	-1.09897 down	1.13E-05
ALPK2	1.282	0.914	3.265	0.609	0.412	0.018	0.027	0.013	0.054	0.048	-5.29701 down	4.07E-69

LIPG	0.254	0.289	0.405	0.319	0.11	0.818	0.461	1.004	1.635	0.353	1.629504	up	5.26E-14
EPG5	5.395	5.745	5.704	2.195	5.286	2.217	2.134	1.973	2.245	2.421	-1.14589	down	2.56E-06
MYORG	1.622	1.313	2.101	2.313	1.466	0.664	0.82	0.661	0.699	0.963	-1.21023	down	7.54E-07
ENHO	11.656	11.328	6.264	7.549	2.636	46.831	35.714	36.456	14.888	36.251	2.109106	up	4.68E-25
ARID3C	2.61	2.312	1.969	1.971	1.86	13.665	4.958	7.935	5.694	7.732	1.898357	up	1.54E-21
AVDL	0.933	0.862	0.83	1.443	1.353	0.24	0.247	0.121	0.6	0.145	-1.99841	down	1.19E-08
AVD	0.679	0.578	0.703	2.345	0.488	0.593	18.42	1.199	0	0.09	2.081472	up	3.44E-17
CAPSL	11.653	11.464	1.396	10.224	0.591	0.515	1.685	1.407	0.879	0.666	-2.77641	down	4.07E-35
NADK2	232.856	198.344	131.607	143.682	91.779	67.143	56.934	90.641	84.934	67.217	-1.1216	down	1.37E-06
LIFR	1.617	2.598	2.908	2.969	3.382	0.916	0.45	1.709	1.684	1.905	-1.01517	down	6.91E-05
TMEM267	3.666	3.814	5.554	3.101	4.556	1.405	2.385	1.499	1.545	2.054	-1.21861	down	1.08E-06
FGF10	0.093	0.119	0.45	0.247	0.258	0.938	0.258	1.487	3.957	1.539	2.803834	up	4.52E-40
EMB	1.242	1.101	1.835	0.799	1.396	0.642	0.688	0.675	0.635	0.428	-1.05346	down	6.47E-05
GZMA	5.311	6.24	9.637	14.31	8.275	4.1	3.734	3.905	5.49	3.815	-1.05645	down	2.13E-05
DHX29	10.579	9.476	11.964	6.815	6.901	2.828	2.694	3.337	3.088	2.972	-1.61582	down	4.13E-12
SETD9	9.059	9.07	6.774	6.311	6.311	3.152	3.422	2.085	1.721	2.808	-1.50827	down	3.91E-10
PDE4D	0.112	0.118	0.248	0.13	0.123	0.104	1.499	0.138	0.175	0.121	1.472205	up	5.30E-14
LOC1211C	0.048	0.158	0.077	0.079	0.099	1.123	0.361	0.106	0.719	0.191	2.426409	up	1.82E-11
HMGCR	31.138	34.359	11.575	7.257	41.515	66.515	32.559	122.38	126.407	75.31	1.749564	up	3.03E-19
ANKRD31	0.761	0.768	0.969	0.828	0.809	1.622	1.726	2.472	4.461	1.479	1.506799	up	5.23E-15
NSA2	71.983	61.831	59.151	64.012	32.467	21.41	35.055	33.337	23.911	29.162	-1.01851	down	1.67E-05
RCL1	16.498	14.89	16.949	17.368	20.811	7.71	9.787	6.974	8.881	9.141	-1.02566	down	2.92E-05
GLDC	69.204	66.091	99.52	67.77	72.787	265.847	104.16	249.4	271.739	211.33	1.554342	up	1.57E-16
PLIN2	135.306	116.516	114.391	25.832	53.914	137.507	100.929	273.367	320.505	137.55	1.120854	up	6.08E-10
GDA	7.224	7.347	8.35	4.976	17.171	4.558	3.07	4.06	4.477	2.859	-1.24406	down	2.84E-07
ZCCHC6	7.306	6.952	6.718	7.362	8.85	3.045	4.239	3.939	3.532	3.404	-1.03395	down	1.66E-05
FBP2	3.111	2.678	4.789	5.166	1.373	0.699	0.375	0.415	0.581	0.744	-2.6026	down	4.83E-25
GADD45G	9.213	10.267	11.004	14.629	6.816	91.904	47.955	199.461	171.456	91.42	3.535494	up	2.33E-58
TPPP2	1.417	1.78	1.445	2.702	3.261	0.33	0.849	0.5	0.282	0.374	-2.18084	down	2.30E-13
STARD4	19.381	18.173	4.566	6.757	24.614	21.825	12.054	49.37	52.869	19.173	1.079283	up	5.23E-09
PPIP5K2	14.486	14.036	15.709	20.013	19.902	4.388	8.684	10.694	8.281	4.706	-1.19492	down	3.21E-07
PCGF3	7.783	6.775	5.021	7.649	2.945	22.007	17.331	13.612	13.935	15.917	1.456256	up	6.00E-14
MFSD7	1.309	1.008	2.038	1.828	1.356	4.163	2.62	2.888	2.727	3.456	1.0719	up	4.82E-09
LPL	1.707	1.894	5.175	0.471	5.173	0.031	0.079	0	0.053	0.154	-5.48537	down	6.30E-76
PSD3	7.349	6.967	7.679	4.194	8.275	0.963	1.03	1.19	1.144	0.885	-2.72401	down	3.75E-32
NIPSNAP3	129.837	127.06	91.021	126.674	103.25	57.88	61.89	60.219	51.099	56.587	-1.00622	down	1.96E-05
CAST	2.373	2.213	3.657	2.944	3.774	0.992	1.104	1.445	1.364	1.381	-1.25033	down	4.00E-07
RHOBTB3	2.66	2.558	3.444	3.373	4.449	0.824	2.256	0.915	1.016	1.578	-1.32228	down	2.82E-08

POLR3G	1.865	1.277	1.678	1.632	1.082	0.729	0.31	0.61	0.806	1.003	-1.12235	down	2.70E-05
HAPLN1	0.057	0.042	0.177	0.026	0	0.037	1.095	0.168	0.196	0.05	2.336888	up	7.16E-22
MSH3	3.914	3.472	1.639	2.695	1.409	0.91	1.118	1.38	1.182	1.199	-1.18067	down	4.88E-07
LOC76841	3.926	3.775	0.9	4.596	3.422	0.952	1.872	1.706	1.595	0.628	-1.2986	down	4.76E-07
ALDOB	11839.6	10520.93	7994.081	8633.508	7428.55	2121.017	1905.804	3336.957	2598.403	2685.884	-1.87573	down	1.22E-16
KIAA1958	30.304	27.952	9.523	15.084	9.131	37.812	46.787	54.528	49.828	35.641	1.287674	up	3.07E-11
LPAR1	0.395	0.296	0.131	0.359	0.11	0.767	0.493	1.78	1.235	1.815	2.233561	up	2.57E-24
C9orf72	1.262	1.403	2.824	1.869	1.883	0.831	1.005	1.083	0.796	0.833	-1.02201	down	8.98E-05
SPINK4	35.548	33.616	33.127	67.803	24.315	19.651	15.349	19.007	16.267	21.312	-1.08585	down	4.42E-06
DMXL1	4.859	4.246	6.89	4.279	5.9	2.373	2.811	1.988	2.379	2.125	-1.16425	down	1.70E-06
SEMA6A	1.654	1.45	1.538	1.04	2.022	0.468	0.617	0.71	0.729	0.959	-1.14414	down	5.71E-06
MARVELD	11.26	10.087	19.877	4.977	12.841	3.127	4.766	5.972	4.781	4.126	-1.37429	down	1.97E-08
CDKN2A	0.782	0.648	0.831	0.964	1.152	6.033	2.969	16.971	3.341	2.685	2.868591	up	3.15E-38
CDKN2B	0.209	0.561	0.183	0.567	0.773	10.226	5.421	8.444	12.008	3.148	4.094315	up	4.00E-66
TRIM36	0.728	0.511	0.826	1.005	0.549	1.971	2.679	2.305	4.359	2.79	1.960961	up	4.24E-22
LOX	0.565	0.698	0.75	0.542	1.354	0.502	0.047	0.208	0.374	0.58	-1.18959	down	0.000363
LOC10705	14.178	14.894	14.025	12.341	21.783	74.327	29.319	31.809	54.943	47.188	1.621322	up	2.83E-17
LOC11253	0.174	0.152	0.277	0.536	0.879	0.718	1.934	0.224	0.864	1.417	1.351434	up	7.46E-11
IER3	5.451	5.622	10.917	4.388	7.055	18.29	20.831	11.78	10.303	10.536	1.10139	up	9.49E-10
LOC11253	0.285	0.521	0.034	0.234	0.556	0.58	0.896	0.471	0.679	1.052	1.171591	up	6.38E-08
LOC10705	0.256	0.28	1.429	1.569	1.434	0.415	0.128	0.126	0.113	0.113	-2.46612	down	9.88E-15
LOC42523	0.597	0.538	1.462	1.46	1.855	0.583	0.589	0.514	0.333	0.327	-1.33159	down	1.37E-07
LOC10705	0.562	0.629	1.534	1.306	1.396	2.783	4.185	2.168	2.529	0.637	1.179924	up	1.72E-10
LOC12110	0.014	0.009	1.624	1.345	1.382	3.976	7.439	2.968	4.422	0	2.102826	up	5.64E-25
CDIPT	3.413	4.386	3.397	4.007	3.005	9.844	15.797	6.051	13.561	11.426	1.637973	up	9.54E-16
PRODH2	0.182	0.159	0.266	0.1	0.125	0.309	0.227	1.204	0.151	0.16	1.296541	up	3.09E-05
LOC12110	0.373	0.929	0.476	0.859	2.072	0.652	0.112	0.33	0	0	-2.10076	down	6.64E-08
LOC12110	0.534	1.017	0.426	4.591	1.247	0.353	1.31	0.322	0.386	0.257	-1.57046	down	1.52E-08
B3GAT3	3.882	4.217	5.891	3.732	4.477	11.831	12.072	5.01	11.459	7.881	1.119949	up	1.44E-09
LOC12110	2.154	2.677	4.031	2.522	3.319	0.392	0.968	1.426	0.749	1.99	-1.41125	down	4.36E-07
LOC12110	1.881	1.885	2.629	1.665	1.527	0.612	1.399	0.987	0.601	0.92	-1.08423	down	2.10E-05
LOC11253	2.155	2.053	0.757	0.13	4.036	0.369	0.237	0	0.346	1.046	-2.1894	down	7.27E-15
LOC10705	0.496	0.46	0.513	0.25	0.032	0.58	1.434	1.242	0.598	0.665	1.365306	up	5.16E-12
LOC11253	3.581	3.767	1.183	5.585	8.088	14.034	19.388	11.851	6.589	10.261	1.484099	up	5.22E-14
LOC12110	1.695	1.763	2.108	1.031	1.527	3.109	4.349	4.97	3.114	3.281	1.21173	up	3.65E-10
MHCY13	3.428	2.686	3.329	0.991	1.896	0.11	0.056	0.083	0.187	0.298	-4.06104	down	1.54E-36
LOC11253	2.993	2.829	7.512	3.722	2.373	0.911	1.432	0.377	0.339	0.706	-2.36595	down	1.14E-22
LOC12110	15.418	15.792	19.848	25.319	21.906	10.968	8.342	7.939	8.922	10.957	-1.06028	down	1.39E-05

qvalue	function
3.61E-43	CLC2DL4; C-type lectin domain family 2 member B isoform X1
1.38E-12	LOC121113333; C-type lectin domain family 2 member B-like isoform X4
3.77E-05	LOC121106515; C-type lectin domain family 2 member D-like
5.62E-47	LOC776463; C-type lectin domain family 2 member B-like
4.47E-11	LOC121109244; C-type lectin domain family 2 member D-like isoform X3
0.001868	LOC121109239; C-type lectin domain family 2 member B-like isoform X3
1.01E-24	LOC112532872; C-type lectin domain family 2 member D-like isoform X3
0.000154	LOC121113328; C-type lectin domain family 2 member F-like
0.000193	LOC121113330; endogenous retrovirus group K member 8 Gag polyprotein-like
4.88E-47	CD69L; C-type lectin domain family 2 member B
9.26E-09	SHANK3; LOW QUALITY PROTEIN: SH3 and multiple ankyrin repeat domains protein 3 isoform X1
9.25E-09	LOC121109250; BCL-6 corepressor-like protein 1 isoform X1
3.66E-05	FBXO18; F-box DNA helicase 1
1.10E-06	ASB13; ankyrin repeat and SOCS box protein 13
1.03E-10	PFKFB3; 6-phosphofructo-2-kinase/fructose-2, 6-bisphosphatase 3 isoform X1
9.43E-10	ITIH5; inter-alpha-trypsin inhibitor heavy chain H5 isoform X1
2.92E-40	ELAPOR2; endosome/lysosome-associated apoptosis and autophagy regulator family member 2 isoform X1
1.99E-10	ORC5; origin recognition complex subunit 5 isoform X1
2.17E-06	ABCD2; ATP-binding cassette sub-family D member 2
1.95E-16	ALG12; dol-P-Man:Man(7)GlcNAc(2)-PP-Dol alpha-1,6-mannosyltransferase isoform X1
1.02E-27	CRELD2; protein disulfide isomerase CRELD2 precursor
#######	LOC429348; tetraspanin-7-like
2.60E-13	TRABD; traB domain-containing protein isoform X1
0.000212	ARL8BL; ADP-ribosylation factor-like 8B-like
5.24E-08	IQUB; IQ and ubiquitin-like domain-containing protein isoform X4
1.62E-25	CPED1; cadherin-like and PC-esterase domain-containing protein 1 isoform X4
2.27E-05	FOXP2; forkhead box protein P2 isoform X9
0.000274	SLC38A4; sodium-coupled neutral amino acid transporter 4 isoform X2
1.52E-11	AMIGO2; amphoterin-induced protein 2 precursor
1.87E-22	SRGAP1; SLIT-ROBO Rho GTPase-activating protein 1 isoform X2
2.53E-06	TSPAN8; tetraspanin-8
8.64E-07	LGR5; leucine-rich repeat-containing G-protein coupled receptor 5 isoform X3
0.000596	LOC121109191; uncharacterized protein LOC121109191 isoform X1
0.000123	ACSS3; LOW QUALITY PROTEIN: acyl-CoA synthetase short-chain family member 3, mitochondrial isoform X1
4.33E-09	PPFIA2; liprin-alpha-2 isoform X14
0.000181	MRPL42; 39S ribosomal protein L42, mitochondrial

4.85E-12 SOCS2; suppressor of cytokine signaling 2
9.51E-13 TMCC3; transmembrane and coiled-coil domain protein 3 isoform X5
2.44E-13 GAS2L3; GAS2-like protein 3 isoform X1
0.000247 TMEM140; transmembrane protein 140
0.000543 LOC427882; histone H1
0.001093 HIST1H2B8; histone H2B 8
0.044397 HIST1H4B; histone H4 type VIII isoform X1
0.004028 HIST1H46L6; histone H4
8.58E-07 LOC100857439; histone H3
8.16E-11 GBE; eye-globin isoform X1
0.00019 CYP2D6; cytochrome P450 2D49
6.98E-09 SHISA8; protein shisa-8
2.51E-07 SREBF2; sterol regulatory element-binding protein 2
1.46E-09 MEI1; meiosis inhibitor protein 1 isoform X12
5.99E-06 CHADL; chondroadherin-like protein precursor
4.22E-08 ADSL; adenylosuccinate lyase
1.12E-46 NPTXR; neuronal pentraxin receptor
6.26E-16 DNAL4; dynein light chain 4, axonemal
2.16E-37 FAM20CL; extracellular serine/threonine protein kinase FAM20C isoform X1
7.36E-16 SUN2; SUN domain-containing protein 2
3.61E-19 KDELR3; ER lumen protein-retaining receptor 3
5.91E-13 LOC101747255; endosome-associated-trafficking regulator 1
7.22E-11 CARD10; caspase recruitment domain-containing protein 10 isoform X7
2.08E-13 MCM5; DNA replication licensing factor MCM5
0.00013 HMOX1; heme oxygenase 1
0.00021 TIMP3; metalloproteinase inhibitor 3 precursor
1.60E-09 TCP11X2; T-complex protein 11-like protein 2 isoform X2
4.71E-21 HSP90B1; endoplasmin precursor
3.55E-26 ASCL1; achaete-scute homolog 1
3.96E-05 PAH; phenylalanine-4-hydroxylase
2.77E-18 IGF1; insulin-like growth factor I preproprotein
3.49E-13 CGTL; cystine/glutamate transporter isoform X4
4.94E-15 CHRM2; muscarinic acetylcholine receptor M2 isoform X2
1.90E-08 WNT5B; protein Wnt-5b precursor
6.44E-11 LOC425137; aldo-keto reductase family 1, member B1-like isoform X3
6.96E-14 RERGL; ras-related and estrogen-regulated growth inhibitor-like protein
1.21E-36 SLCO1B1; solute carrier organic anion transporter family member 1C1 isoform X1
1.09E-34 LDHB; L-lactate dehydrogenase B chain isoform X1

0.000175 GYS2; glycogen [starch] synthase, liver isoform X2
1.40E-25 SSPN; sarcospan isoform X1
7.66E-31 BHLHE41; class E basic helix-loop-helix protein 41
1.97E-07 MPPED1; metallophosphoesterase domain-containing protein 1
6.02E-22 SULT4A1; sulfotransferase 4A1 isoform X2
3.15E-26 PNPLA3; patatin-like phospholipase domain-containing protein 2 isoform X3
0.000157 SAMM50; sorting and assembly machinery component 50 homolog
0.000269 PRR5; proline-rich protein 5 isoform X2
3.52E-07 KIAA0930; uncharacterized protein KIAA0930 homolog isoform X3
8.78E-06 CREBL2; cAMP-responsive element-binding protein-like 2
8.33E-05 VWF; von Willebrand factor isoform X2
0.000109 PHC1; polyhomeotic-like protein 1
3.12E-07 A2ML3; alpha-2-macroglobulin-like isoform X6
2.78E-09 CASR; extracellular calcium-sensing receptor
0.005912 CSTA; cystatin-A
1.59E-08 GPR162; probable G-protein coupled receptor 162
6.49E-16 GNB3; guanine nucleotide-binding protein G(I)/G(S)/G(T) subunit beta-3
3.47E-11 CDCA3; cell division cycle-associated protein 3 isoform 2
1.92E-07 RBP5; retinol-binding protein 5
2.46E-27 EPHA1; ephrin type-A receptor 1 precursor
2.20E-09 GSTK1; glutathione S-transferase kappa 1 isoform X1
5.39E-08 KEL; kell blood group glycoprotein isoform X4
7.15E-15 STYK1; tyrosine-protein kinase STYK1
2.67E-08 LOC100859872; uncharacterized protein LOC100859872 isoform X1
1.30E-06 HSD3B1; hydroxy-delta-5-steroid dehydrogenase, 3 beta- and steroid delta-isomerase 1
7.68E-14 HAO2; hydroxyacid oxidase 2
4.76E-27 SIDT1; SID1 transmembrane family member 1 isoform X2
2.60E-08 MAB21L3; protein mab-21-like 3 isoform X1
2.84E-10 SLC22A15; solute carrier family 22 member 15 isoform X3
2.86E-06 ZBTB20; zinc finger and BTB domain-containing protein 20 isoform X2
9.28E-19 CLDND1; claudin domain-containing protein 1 isoform X2
1.30E-23 APOV1; apovitellenin-1 precursor
6.68E-05 CEP97; centrosomal protein of 97 kDa isoform X1
8.91E-20 LOC418414; uncharacterized protein C3orf85 homolog
1.38E-06 NECTIN3; nectin-3 isoform X2
2.36E-12 CREG1; protein CREG1 precursor
1.48E-08 MAEL; protein maelstrom homolog isoform X3
1.28E-06 BCL9; B-cell CLL/lymphoma 9 protein isoform X2

8.62E-09 ROBO1; roundabout homolog 1 isoform X7
1.07E-05 ABCC13; multidrug resistance-associated protein 1 isoform X2
1.03E-29 ADAMTS1; LOW QUALITY PROTEIN: A disintegrin and metalloproteinase with thrombospondin motifs 1 isoform X1
3.61E-22 MAP3K7CL; MAP3K7 C-terminal-like protein isoform X4
3.58E-09 EVA1C; protein eva-1 homolog C isoform X5
1.40E-05 GART-B; trifunctional purine biosynthetic protein adenosine-3 isoform X2
0.000174 SETD4; SET domain-containing protein 4 isoform X3
1.08E-08 CBR3; carbonyl reductase [NADPH] 1
5.58E-06 IGSF5; immunoglobulin superfamily member 5 isoform X3
8.11E-12 PCP4; calmodulin regulator protein PCP4
5.81E-05 AGPAT3; 1-acyl-sn-glycerol-3-phosphate acyltransferase gamma
1.47E-19 MID1IP1; mid1-interacting protein 1
4.48E-23 OTC; ornithine transcarbamylase, mitochondrial isoform X1
1.99E-11 NR0B1; nuclear receptor subfamily 0 group B member 1 isoform X2
9.09E-50 SLC51AL; organic solute transporter subunit alpha
3.19E-34 MAP3K15; mitogen-activated protein kinase kinase kinase 15
1.35E-30 PDHA2; pyruvate dehydrogenase E1 component subunit alpha, somatic form, mitochondrial precursor
3.81E-59 ADGRG2; adhesion G-protein coupled receptor G2 isoform X1
9.67E-05 PHKA2; phosphorylase b kinase regulatory subunit alpha, liver isoform isoform X3
8.51E-05 PPEF1; serine/threonine-protein phosphatase with EF-hands 1
8.20E-08 SCML2; sex comb on midleg-like protein 2 isoform X5
3.62E-37 GRPR; gastrin-releasing peptide receptor
####### LOC121106447; uncharacterized protein LOC121106447
####### LOC107052718; LOW QUALITY PROTEIN: uncharacterized protein LOC107052718
1.03E-16 PRKX; cAMP-dependent protein kinase catalytic subunit PRKX isoform X2
7.70E-12 LOC418658; arylsulfatase D isoform X2
8.82E-07 SLC25A6; ADP/ATP translocase 3
3.53E-06 RGN; regucalcin isoform X3
7.62E-09 LYGL; lysozyme g isoform X1
7.91E-15 LYG2; lysozyme g isoform X1
5.89E-08 RNF149; E3 ubiquitin-protein ligase RNF149 isoform X1
8.73E-09 IL1R2; interleukin-1 receptor type 2 isoform X1
6.74E-07 IL1R1; interleukin-1 receptor type 1 precursor
6.98E-12 IL1RL1; interleukin-1 receptor-like 1 isoform LV precursor
4.89E-05 GAS6; growth arrest-specific protein 6
1.30E-09 LOC771012; coagulation factor X
1.76E-09 RAB20; ras-related protein Rab-20
8.39E-11 LOC121107687; SPARC

4.01E-06 GPR183; G-protein coupled receptor 183 isoform X2
2.43E-08 DOCK9; dedicator of cytokinesis protein 9 isoform X17
4.04E-24 HS6ST3; heparan-sulfate 6-O-sulfotransferase 3
1.72E-06 DZIP1; zinc finger protein DZIP1 isoform X4
3.09E-43 DCT; L-dopachrome tautomerase precursor
3.41E-05 CLN5; ceroid-lipofuscinosis neuronal protein 5 precursor
4.99E-09 ACOD1; cis-aconitate decarboxylase
5.09E-06 CPB2; carboxypeptidase B2
1.35E-06 WDFY2; WD repeat and FYVE domain-containing protein 2 isoform X1
3.05E-08 SLC25A15; mitochondrial ornithine transporter 1 isoform X1
6.57E-08 UFM1; ubiquitin-fold modifier 1
1.98E-19 POSTN; periostin isoform X14
9.12E-08 LOC121106501; uncharacterized protein LOC121106501 isoform X2
3.70E-13 N4BP2L1; NEDD4-binding protein 2-like 1 isoform X1
1.98E-09 FLT1; vascular endothelial growth factor receptor 1 precursor
1.23E-57 SGCG; gamma-sarcoglycan isoform X4
1.11E-19 GJB2; gap junction beta-2 protein
2.42E-09 ACAT1; acetyl-CoA acetyltransferase, mitochondrial
2.48E-10 CCDC82; coiled-coil domain-containing protein 82 isoform X2
0.000243 MTMR2; myotubularin-related protein 2
1.32E-14 ENDOD1; endonuclease domain-containing 1 protein
3.22E-06 TAF1D; TATA box-binding protein-associated factor RNA polymerase I subunit D
6.73E-06 CEP295; centrosomal protein of 295 kDa isoform X1
1.46E-22 LOC121107448; uncharacterized protein LOC121107448
1.94E-10 LOC121107447; uncharacterized protein LOC121107447 isoform X1
4.19E-36 LOC101750591; uncharacterized protein LOC101750591
1.56E-14 LOC101748650; thyroid hormone-inducible hepatic protein-like
2.38E-34 THRSPB; thyroid hormone responsive spot 14 beta
1.54E-13 THRSP; thyroid hormone-inducible hepatic protein
8.25E-18 CAPN5; calpain-5
1.59E-12 TSKU; tsukushin precursor
3.62E-11 WNT11; protein Wnt-11 isoform X4
0.001098 ART1L2; erythroblast NAD(P)(+)--arginine ADP-ribosyltransferase isoform X1
7.73E-05 IL18BP; interleukin-18-binding protein isoform X2
0.000163 PDE2A; LOW QUALITY PROTEIN: cGMP-dependent 3',5'-cyclic phosphodiesterase isoform X1
3.08E-09 WDR73; WD repeat-containing protein 73 isoform X1
2.37E-45 ADAM15; disintegrin and metalloproteinase domain-containing protein 9 isoform X3
1.36E-08 SLCO2B1; solute carrier organic anion transporter family member 2B1 isoform X2

1.23E-05 POLD3; DNA polymerase delta subunit 3 isoform X2
1.99E-44 HBE; hemoglobin subunit epsilon
6.60E-32 HBBA; hemoglobin subunit beta
3.77E-38 HBE1; hemoglobin subunit epsilon 1
4.92E-07 TAF10; transcription initiation factor TFIID subunit 10
6.86E-14 TPP1; tripeptidyl-peptidase 1
6.31E-11 GIMAP1-GIMAP5; GTPase IMAP family member 1
1.44E-09 ELP6; elongator complex protein 6
1.87E-15 SCAP; sterol regulatory element-binding protein cleavage-activating protein isoform X2
1.58E-36 ACKR2; atypical chemokine receptor 2
1.55E-42 CYP8B1; cytochrome P450, family 8, subfamily B
2.16E-11 CHDSD; D-threo-3-hydroxyaspartate dehydratase isoform X3
1.72E-05 CATH3; cathelicidin-3 precursor
7.85E-14 CATH2; cathelicidin-2 precursor
3.48E-08 CATH1; cathelicidin-1 precursor
1.45E-25 CRHR2; corticotropin-releasing factor receptor 2
4.02E-26 DLEC1; deleted in lung and esophageal cancer protein 1 isoform X3
8.45E-27 ACAA1; 3-ketoacyl-CoA thiolase, peroxisomal
6.37E-06 SLC22A13L; solute carrier family 22 member 13-like
2.17E-08 CSRNP1; cysteine/serine-rich nuclear protein 1 isoform X2
5.50E-05 RHEB; GTP-binding protein Rheb isoform X1
6.56E-13 IDI2; isopentenyl-diphosphate Delta-isomerase 1 isoform X2
7.58E-09 KLF6; Krueppel-like factor 6
3.28E-10 MAP3K8; mitogen-activated protein kinase kinase kinase 8 isoform X1
9.78E-08 APBB1IP; amyloid beta A4 precursor protein-binding family B member 1-interacting protein
1.69E-59 NELL3; uncharacterized protein NELL3
1.12E-05 OTUD1; OTU domain-containing protein 1
2.20E-13 RSU1; ras suppressor protein 1
2.43E-07 NMT2; glycylpeptide N-tetradecanoyltransferase 2
1.17E-10 ACBD7; acyl-CoA-binding domain-containing protein 7 isoform X2
7.73E-12 OLAH; S-acyl fatty acid synthase thioesterase, medium chain isoform X1
4.76E-08 CROT; peroxisomal carnitine O-octanoyltransferase
5.46E-39 ABCB1LA; phosphatidylcholine translocator ABCB4 isoform X5
5.24E-21 CYP51A1; leucine-rich repeat and death domain-containing protein 1 isoform X2
3.50E-18 CDK6; cyclin-dependent kinase 6
2.68E-10 TFPI2; tissue factor pathway inhibitor 2 isoform X2
4.09E-19 PDK4; pyruvate dehydrogenase kinase, isozyme 4
1.55E-08 AHR1A; aryl hydrocarbon receptor isoform X3

5.11E-17 HDAC9; histone deacetylase 9 isoform X11
1.71E-10 WIPF3; WAS/WASL-interacting protein family member 3 isoform X2
1.94E-11 FKBP14; peptidyl-prolyl cis-trans isomerase FKBP14
9.58E-18 SATB1; DNA-binding protein SATB1 isoform X1
0.001434 LRRC3B; leucine-rich repeat-containing protein 3B
3.03E-05 CMTM8; CKLF-like MARVEL transmembrane domain-containing protein 8
0.000157 SNRK; SNF-related serine/threonine-protein kinase isoform X1
1.01E-11 ACAD11; acyl-CoA dehydrogenase family member 11
7.07E-07 ACKR4; atypical chemokine receptor 4
2.12E-13 MOBP; rab effector MyRIP isoform X5
2.37E-26 AMPH; amphiphysin isoform X1
0.000204 LOC121109702; uncharacterized protein LOC121109702
2.29E-26 INHBA; inhibin beta A chain isoform X1
4.71E-10 EGFR; epidermal growth factor receptor isoform X2
2.53E-09 PDIA4; protein disulfide-isomerase A4 isoform X1
8.85E-05 RAMP3; receptor activity-modifying protein 3 isoform X1
9.52E-41 IGFBP1; insulin-like growth factor-binding protein 1 precursor
2.60E-17 KCNG2; potassium voltage-gated channel subfamily G member 2 isoform X1
1.89E-26 SALL3; sal-like protein 3 isoform X1
3.94E-22 DCDC2; doublecortin domain-containing protein 2 isoform X4
9.99E-21 NRSN1; neurensin-1
6.76E-08 EDN1; endothelin-1
3.56E-16 ELOVL2; elongation of very long chain fatty acids protein 2
3.25E-19 SYCP2L; synaptonemal complex protein 2-like isoform X3
1.53E-21 GCNT2; N-acetyllactosaminide beta-1,6-N-acetylglucosaminyl-transferase isoform X2
1.04E-07 TXNDC5; thioredoxin domain-containing protein 5 isoform X2
5.26E-05 F13A1; coagulation factor XIII A chain
2.18E-12 PPP1R3G; protein phosphatase 1 regulatory subunit 3G
1.56E-33 ECI2; enoyl-CoA delta isomerase 2 isoform X3
0.001574 IRF4; interferon regulatory factor 4
5.52E-05 SERPINB10B; heterochromatin-associated protein MENT isoform X1
3.49E-06 FAM134B; reticulophagy regulator 1 isoform X4
2.09E-07 ANKRD33B; ankyrin repeat domain-containing protein 33B isoform X2
3.58E-44 CMBL; carboxymethylenebutenolidase homolog
7.14E-13 SRD5A1; 3-oxo-5-alpha-steroid 4-dehydrogenase 1 isoform X3
0.001395 UPP1; uridine phosphorylase 1
1.31E-08 LOC428505; SUN domain-containing protein 3 isoform X1
2.75E-16 MOCOS; molybdenum cofactor sulfurase isoform X1

6.20E-10	SEC61B; protein transport protein Sec61 subunit beta
1.04E-17	IFI6; interferon alpha inducible protein 6
2.49E-13	GLIPR2; Golgi-associated plant pathogenesis-related protein 1
0.00024	MBP; myelin basic protein
3.50E-08	ZNF516; zinc finger protein 516 isoform X2
6.53E-42	C18orf63; uncharacterized protein C18orf63 homolog isoform X3
3.69E-08	FBXO15; F-box only protein 15 isoform X6
5.94E-05	LDLRAD4; low-density lipoprotein receptor class A domain-containing protein 4 isoform X8
1.37E-10	PSMG2; proteasome assembly chaperone 2
1.60E-13	CIDEA; cell death activator CIDE-A
2.39E-05	VAPA; vesicle-associated membrane protein-associated protein A isoform 2
3.50E-08	TGIF1; homeobox protein AKR
1.40E-12	MYOM1; myomesin-1
7.61E-18	LPIN2; phosphatidate phosphatase LPIN2 isoform X1
1.04E-08	LOC107052005; translation initiation factor IF-2
8.64E-31	ABHD3; phospholipase ABHD3 isoform X1
0.000189	CEBPD; CCAAT/enhancer-binding protein delta
8.07E-08	MCM4; DNA replication licensing factor MCM4
5.58E-34	C8orf22; pancreatic progenitor cell differentiation and proliferation factor-like protein
2.47E-07	PCMTD1; protein-L-isoaspartate O-methyltransferase domain-containing protein 1 isoform X1
1.04E-06	FAM110B; protein FAM110B isoform X1
2.32E-35	CYP7A1; cholesterol 7-alpha-monooxygenase
1.36E-05	TTPA; alpha-tocopherol transfer protein
0.000221	CYP7B1; cytochrome P450 7B1
5.92E-07	MTFR1; mitochondrial fission regulator 1 isoform X2
1.11E-05	ADHFE1; hydroxyacid-oxoacid transhydrogenase, mitochondrial isoform X2
1.92E-75	SULF1; extracellular sulfatase Sulf-1 isoform X2
3.28E-10	XKR9; XK-related protein 9 isoform X2
3.26E-31	TRPA1; transient receptor potential cation channel subfamily A member 1
7.79E-05	C2H8ORF84; somatomedin-B and thrombospondin type-1 domain-containing protein isoform X1
2.07E-05	PKIA; cAMP-dependent protein kinase inhibitor alpha
3.15E-18	ZC2HC1A; zinc finger C2HC domain-containing protein 1A isoform X2
4.45E-05	HEY1; hairy/enhancer-of-split related with YRPW motif protein 1 isoform X2
6.43E-12	FABP5; fatty acid binding protein 5
4.94E-13	FABP4; fatty acid-binding protein, adipocyte
5.93E-06	CA13; carbonic anhydrase 13
1.25E-28	ATP6V0D2; V-type proton ATPase subunit d 2
1.36E-11	WWP1; NEDD4-like E3 ubiquitin-protein ligase WWP1 isoform X3

0.000469 SLC7A7; Y+L amino acid transporter 2
1.39E-05 DECR1; 2,4-dienoyl-CoA reductase [(3E)-enoyl-CoA-producing], mitochondrial
2.02E-05 NDUFAF6; NADH dehydrogenase (ubiquinone) complex I, assembly factor 6 isoform X4
1.05E-22 PTDSS1; phosphatidylserine synthase 1 isoform X1
6.82E-22 CPQ; carboxypeptidase Q isoform X1
4.21E-23 NCALD; neurocalcin-delta
1.28E-07 FZD6; frizzled-6 isoform X2
2.15E-10 RIMS2; regulating synaptic membrane exocytosis protein 2 isoform X50
5.56E-18 DCSTAMP; LOW QUALITY PROTEIN: dendritic cell-specific transmembrane protein
6.62E-07 EIF3E; eukaryotic translation initiation factor 3 subunit E
2.07E-08 EBAG9; receptor-binding cancer antigen expressed on SiSo cells isoform X2
2.59E-08 SYBU; syntabulin isoform X9
2.31E-05 ENPP2; ectonucleotide pyrophosphatase/phosphodiesterase family member 2 isoform X14
4.30E-05 FBXO32; F-box only protein 32
2.04E-10 ANXA13; annexin A13
5.12E-14 FER1L6; fer-1-like protein 6 isoform X1
4.45E-26 SQLE; squalene monooxygenase
2.71E-06 NDRG1; protein NDRG1 isoform X1
2.67E-09 ST3GAL1; CMP-N-acetylneuraminate-beta-galactosamide- alpha-2,3-sialyltransferase 1
3.04E-16 LOC428383; leucine-rich repeat protein lrrA-like
9.11E-22 RHPN1; rhophilin-1 isoform X1
7.33E-14 TSTA3; GDP-L-fucose synthase isoform X2
0.000365 LOC121113091; uncharacterized protein LOC121113091
8.04E-08 PRORSD1P; PrdX-deacylase domain 1
1.84E-11 VRK2; serine/threonine-protein kinase VRK2 isoform X7
1.08E-23 FANCL; E3 ubiquitin-protein ligase FANCL isoform X1
3.40E-08 KIAA1841; uncharacterized protein KIAA1841 homolog isoform X6
1.28E-05 TPCN3; two-pore calcium channel 3 isoform X2
9.40E-27 BUB1; mitotic checkpoint serine/threonine-protein kinase BUB1
3.51E-09 LOC421232; GDNF-inducible zinc finger protein 1
1.65E-18 CAPN13; calpain-13 isoform X2
0.000138 EPCAM; epithelial cell adhesion molecule precursor
2.01E-06 PPP1R21; protein phosphatase 1 regulatory subunit 21 isoform X2
1.80E-05 EHBP1; EH domain-binding protein 1 isoform X21
2.29E-26 UGP2; UTP--glucose-1-phosphate uridylyltransferase isoform X1
3.70E-10 LGALSL; galectin-related protein
4.74E-07 LOC121110286; translation initiation factor IF-2-like isoform X1
3.36E-05 C1D; nuclear nucleic acid-binding protein C1D

6.14E-08	RRBP1; ribosome-binding protein 1 isoform X2
0.000397	CAPN2; calpain-2 catalytic subunit
2.46E-20	TLR5; toll-like receptor 5 precursor
7.73E-06	LPGAT1; acyl-CoA:lysophosphatidylglycerol acyltransferase 1 isoform X2
1.99E-07	PKDCCA; extracellular tyrosine-protein kinase PKDCC
2.99E-05	SLC3A1; neutral and basic amino acid transport protein rBAT
2.48E-05	RBKS; ribokinase isoform X2
1.13E-09	LOC421419; uncharacterized protein LOC421419 isoform 2
9.10E-09	KCNK5; potassium channel subfamily K member 5 isoform X2
0.000343	LOC421441; uncharacterized protein LOC421441
1.90E-05	RMDN2; regulator of microtubule dynamics protein 2 isoform X3
5.13E-05	TFB2M; dimethyladenosine transferase 2, mitochondrial
0.000835	SMYD3; histone-lysine N-methyltransferase SMYD3 isoform X3
0.000411	LOC421506; uncharacterized protein LOC421506
2.20E-10	NID1; nidogen-1 isoform X1
2.73E-06	SLC35F3; putative thiamine transporter SLC35F3 isoform X2
4.11E-07	ACTA1; actin, alpha skeletal muscle isoform X1
2.12E-09	C1orf131; uncharacterized protein C1orf131 homolog
1.06E-10	GNPAT; dihydroxyacetone phosphate acyltransferase isoform X1
0.000125	THBS2; thrombospondin-2 isoform X2
1.61E-05	SULT6B1L; sulfotransferase 6B1
8.10E-06	SULT; sulfotransferase
1.26E-21	DACT2; dapper homolog 2
2.56E-20	MPC1L; mitochondrial pyruvate carrier 1
0.00156	LOC421583; solute carrier family 22 member 2
2.35E-38	FNDC1; fibronectin type III domain-containing protein 1
6.46E-37	LRP11; low-density lipoprotein receptor-related protein 11
1.64E-05	ESR1; estrogen receptor
2.37E-11	IL22RA2; interleukin-22 receptor subunit alpha-2 isoform X2
5.42E-08	IL20RA; interleukin-20 receptor subunit alpha isoform X1
6.43E-12	MAP3K5; mitogen-activated protein kinase kinase kinase 5
4.47E-22	SGK1; serine/threonine-protein kinase Sgk1
2.35E-36	SLC2A12; solute carrier family 2, facilitated glucose transporter member 12
6.50E-16	LOC107056139; pantetheinase-like precursor
2.65E-07	ARHGAP18; rho GTPase-activating protein 18 isoform X3
2.98E-22	GJA1; gap junction alpha-1 protein
1.39E-09	MCM9; DNA helicase MCM9 isoform X1
2.32E-24	DCBLD1; discoidin, CUB and LCCL domain-containing protein 1 isoform X2

1.21E-22 LOC421740; sulfotransferase family 3A, member 1-like
0.000125 SLC16A10; monocarboxylate transporter 10 isoform X2
3.97E-12 DDO; D-aspartate oxidase
1.08E-06 METTL24; methyltransferase-like protein 24 isoform X4
3.93E-05 SESN1; sestrin-1 isoform X10
9.79E-05 LOC107053100; translation initiation factor IF-2-like isoform X1
9.40E-12 LOC121113177; uncharacterized protein LOC121113177
0.000753 GPR63; probable G-protein coupled receptor 63
1.22E-39 ME1; NADP-dependent malic enzyme
1.03E-29 RWDD2A; RWD domain-containing protein 2A
9.52E-13 SH3BGRL2; SH3 domain-binding glutamic acid-rich-like protein 2 isoform X1
0.007316 BAG2; BAG family molecular chaperone regulator 2
0.000154 HMGCLL1; 3-hydroxymethyl-3-methylglutaryl-CoA lyase, cytoplasmic isoform X1
2.52E-07 TINAG; tubulointerstitial nephritis antigen isoform X1
0.000174 GCLC; glutamate--cysteine ligase catalytic subunit
1.91E-06 GSTA3; glutathione S-transferase
0.000285 ANGPT2; angiopoietin-2
7.85E-06 FAM110C; protein FAM110C
3.40E-16 MBOAT2; lysophospholipid acyltransferase 2 isoform X1
0.000201 ASAP2; arf-GAP with SH3 domain, ANK repeat and PH domain-containing protein 2 isoform X7
4.02E-10 KLF11; Krueppel-like factor 11 isoform X2
7.57E-13 RRM2; ribonucleoside-diphosphate reductase subunit M2
1.67E-10 ATP6V1C2; V-type proton ATPase subunit C 2 isoform X2
5.63E-10 PDIA6; protein disulfide-isomerase A6
1.46E-05 LPIN1; phosphatidate phosphatase LPIN1 isoform X7
4.21E-10 VSNL1; visinin-like protein 1 isoform X2
6.42E-07 TTC32; tetratricopeptide repeat protein 32 isoform X2
1.77E-15 LOC101748329; LOW QUALITY PROTEIN: rho-related GTP-binding protein RhoB-like
6.56E-26 RHOB; rho-related GTP-binding protein RhoB
2.31E-06 HS1BP3; HCLS1-binding protein 3 isoform X1
2.49E-09 APOB; apolipoprotein B precursor
3.01E-20 CGREF1; cell growth regulator with EF hand domain protein 1
0.000341 SLC5A6; sodium-dependent multivitamin transporter
1.00E-26 DTNB; dystrobrevin beta isoform X11
7.67E-28 KIF3C; kinesin-like protein KIF3C
1.10E-08 HADHA; trifunctional enzyme subunit alpha, mitochondrial
4.87E-16 HADHB; trifunctional enzyme subunit beta, mitochondrial
3.20E-13 LOC121110416; protein aveugle-like isoform X1

1.89E-17 GVINP1; interferon-induced very large GTPase 1
6.90E-30 LOC770271; uncharacterized protein LOC770271
9.01E-07 C3H8ORF80; nuclear GTPase SLIP-GC
0.000183 ZNF395; zinc finger protein 395 isoform X2
1.03E-06 LOC426385; serine/threonine-protein kinase 35-like isoform X1
0.000118 FZD3; frizzled-3 isoform X1
5.30E-08 MSRA; mitochondrial peptide methionine sulfoxide reductase isoform X14
1.33E-60 SOX7; transcription factor SOX-7
0.000552 XKR6; XK-related protein 6
7.25E-05 TDH; L-threonine 3-dehydrogenase, mitochondrial
2.83E-30 BLK; tyrosine-protein kinase Blk isoform X1
4.14E-33 FDFT1; squalene synthase
1.05E-19 DEFB4A; gallinacin-2 precursor
1.66E-18 MCM3; DNA replication licensing factor MCM3 isoform X1
4.02E-20 CRISP2; serotriflin isoform X1
4.79E-16 RHAG; ammonium transporter Rh type A
1.63E-35 CYP2AC1; cytochrome P450 2AC1
9.32E-12 SUPT3H; transcription initiation protein SPT3 homolog isoform X2
2.58E-06 RCAN2; calcipressin-2 isoform X2
8.98E-16 ADGRF5; adhesion G protein-coupled receptor F5
6.38E-11 TNFRSF21; tumor necrosis factor receptor superfamily member 21 isoform X1
0.000273 OPHN1; oligophrenin-1 isoform X3
2.16E-06 RXFP2; relaxin receptor 1
6.17E-14 ARR3; arrestin-C
6.05E-16 LOC422147; putative P2Y purinoceptor 10
0.001292 RAB33A; RAB33A, member RAS oncogene family
3.81E-09 CCNB3; G2/mitotic-specific cyclin-B3 isoform X2
4.28E-05 DLG3; disks large homolog 3 isoform X3
2.43E-44 SLC6A14; sodium- and chloride-dependent neutral and basic amino acid transporter B(0+) isoform X2
1.45E-12 GPC3; glypican-3
2.19E-12 XKRX; XK-related protein 2 isoform X2
4.17E-07 LOC771612; mitochondrial fission factor homolog B isoform X3
3.88E-11 ZNF185; zinc finger protein 185 isoform X5
4.18E-13 NSDHL; sterol-4-alpha-carboxylate 3-dehydrogenase, decarboxylating
4.22E-05 RASL11AL; ras-like protein family member 11A-like
3.24E-18 SLC16A2; monocarboxylate transporter 8
9.28E-19 IRS4; insulin receptor substrate 4
1.76E-05 COL4A5; collagen alpha-5(IV) chain isoform X1

1.34E-39 LOC112532392; glyceraldehyde-3-phosphate dehydrogenase, testis-specific-like
3.40E-05 IDS; iduronate 2-sulfatase isoform X4
1.95E-11 KLHL2; kelch-like protein 2 isoform X4
4.75E-18 MSMO1; methylsterol monooxygenase 1
2.71E-05 MGARP; protein MGARP isoform X6
0.000667 LOC422442; uncharacterized protein LOC422442 isoform X2
1.31E-09 SMAD1; mothers against decapentaplegic homolog 1 isoform X2
1.78E-12 TTC29; tetratricopeptide repeat protein 29 isoform X3
0.000782 ARHGAP10; rho GTPase-activating protein 10
4.58E-05 SH3D19; SH3 domain-containing protein 19 isoform X8
0.000103 HGSNAT; heparan-alpha-glucosaminide N-acetyltransferase
4.43E-11 SLC20A2; sodium-dependent phosphate transporter 2
3.19E-06 ETNPPL; ethanolamine-phosphate phospho-lyase isoform X2
0.000435 SGMS2; phosphatidylcholine:ceramide cholinephosphotransferase 2
6.23E-08 GSTCD; glutathione S-transferase C-terminal domain-containing protein isoform X2
2.91E-12 ARHGEF38; rho guanine nucleotide exchange factor 38
3.69E-35 ACSL1; long-chain-fatty-acid--CoA ligase 1
2.08E-11 WWC2; protein WWC2 isoform X1
3.12E-07 DCTD; deoxycytidylate deaminase isoform X2
9.04E-05 VEGFC; vascular endothelial growth factor C
7.34E-05 AFF1; LOW QUALITY PROTEIN: AF4/FMR2 family member 1 isoform X1
4.01E-14 PLACL2; PLAC8 like 2
1.32E-13 GPAT3; glycerol-3-phosphate acyltransferase 3 isoform X1
5.05E-17 DUSP4; dual specificity protein phosphatase 4
2.19E-15 PPP1R3B; protein phosphatase 1 regulatory subunit 3B isoform X1
1.75E-86 LOC430303; prolow-density lipoprotein receptor-related protein 1 isoform X3
6.25E-18 SHROOM3; protein Shroom3 isoform X7
3.70E-15 STBD1; starch-binding domain-containing protein 1 isoform X2
0.000377 NPFFR2; neuropeptide FF receptor 2
2.22E-07 ADAMTS3; A disintegrin and metalloproteinase with thrombospondin motifs 3 isoform X3
1.34E-39 SULT1B; sulfotransferase family, cytosolic, 1B isoform X1
0.003132 FABP2; fatty acid-binding protein, intestinal
1.52E-14 ENPEP; glutamyl aminopeptidase
3.10E-10 ELOVL6; elongation of very long chain fatty acids protein 6
3.24E-07 CASP6; caspase-6
0.00056 EMCN; endomucin precursor
7.78E-33 CCDC110; coiled-coil domain-containing protein 110
7.67E-24 CYP4V2; cytochrome P450 family 4 subfamily V member 2

6.52E-07 KLKB1; plasma kallikrein isoform X2
3.74E-27 FAT1; protocadherin Fat 1 isoform X8
1.05E-14 PDGFRL; platelet-derived growth factor receptor-like protein isoform X5
3.58E-09 DLC1; rho GTPase-activating protein 7 isoform X4
0.003781 LRRC66; leucine-rich repeat-containing protein 66 isoform X2
7.83E-06 ATP10D; phospholipid-transporting ATPase VD isoform X1
0.000166 LIMCH1; LIM and calponin homology domains-containing protein 1 isoform X14
2.24E-09 UCHL1; ubiquitin carboxyl-terminal hydrolase isozyme L1
9.65E-05 UGDH; UDP-glucose 6-dehydrogenase
2.24E-12 FAM114A1; protein NOXP20 isoform X1
6.33E-08 TBC1D1; TBC1 domain family member 1
8.44E-83 CCKAR; cholecystokinin receptor type A
1.05E-46 RBPJ; recombining binding protein suppressor of hairless isoform X1
2.96E-06 SMIM20; small integral membrane protein 20
3.39E-13 SEL1L3; protein sel-1 homolog 3
6.83E-06 ZCCHC4; rRNA N6-adenosine-methyltransferase ZCCHC4 isoform X2
4.13E-53 PPARGC1A; peroxisome proliferator-activated receptor gamma coactivator 1-alpha isoform X1
1.10E-06 LCORL; ligand-dependent nuclear receptor corepressor-like protein isoform X1
1.46E-10 TAPT1; transmembrane anterior posterior transformation protein 1 homolog isoform X2
7.01E-05 PROM1; prominin-1 isoform X15
6.51E-05 FGFBP2; fibroblast growth factor-binding protein 2 precursor
8.05E-05 KIAA0232; uncharacterized protein KIAA0232 homolog isoform X5
3.63E-15 HAUS3; HAUS augmin-like complex subunit 3
3.28E-10 FGFR3; fibroblast growth factor receptor 3 isoform X7
3.52E-09 SPON2; spondin-2 isoform X2
2.10E-12 ATOH8; protein atonal homolog 8 isoform X1
3.87E-46 SMYD1; histone-lysine N-methyltransferase SMYD1
8.93E-07 SMOX; spermine oxidase isoform X1
7.06E-08 DNAAF9; uncharacterized protein C20orf194 homolog isoform X1
6.99E-16 NAT8; putative N-acetyltransferase 8B
8.23E-11 LOC107056412; membrane-spanning 4-domains subfamily A member 12-like isoform X3
1.06E-06 TMEM109; transmembrane protein 109
5.62E-08 DAGLA; diacylglycerol lipase-alpha isoform X2
0.000208 TBX10; T-box transcription factor TBX10 isoform X1
2.57E-12 TCIRG1; V-type proton ATPase 116 kDa subunit a3
1.89E-13 ALDH3B1; aldehyde dehydrogenase family 3 member B1
1.53E-16 SPTB; spectrin beta chain, erythrocytic isoform X1
9.46E-06 C15orf52; coiled-coil domain-containing protein 9B isoform X6

0.000717	LOC770492; ras-related and estrogen-regulated growth inhibitor
0.000243	ANO9; anoctamin-9 isoform X1
8.33E-20	B4GALNT4; N-acetyl-beta-glucosaminyl-glycoprotein 4-beta-N-acetylgalactosaminyltransferase 1 isoform X1
6.48E-09	LOC770612; interferon-induced transmembrane protein 3
5.79E-22	DHCR7; 7-dehydrocholesterol reductase
2.98E-15	RCN1; reticulocalbin-1 isoform X1
1.05E-20	PRRG4; transmembrane gamma-carboxyglutamic acid protein 4 isoform X1
7.62E-22	MICAL2; F-actin-monooxygenase MICAL2 isoform X20
2.67E-05	CTR9; RNA polymerase-associated protein CTR9 homolog
5.77E-11	AMPD3; AMP deaminase 3 isoform X2
0.000226	SWAP70; switch-associated protein 70 isoform X2
2.64E-10	PDE3B; cGMP-inhibited 3',5'-cyclic phosphodiesterase B
2.54E-07	USH1C; harmonin isoform X1
3.05E-93	SAA; serum amyloid A isoform X2
8.90E-30	TMEM86A; lysoplasmalogenase-like protein TMEM86A
1.18E-07	KCNQ1; potassium voltage-gated channel subfamily KQT member 1
1.75E-86	TRPM5; transient receptor potential cation channel subfamily M member 5 isoform X1
7.47E-08	CD81; CD81 antigen
2.65E-17	CTSD; cathepsin D precursor
8.44E-20	DUSP8; dual specificity protein phosphatase 8 isoform X2
7.14E-08	EFCAB4B; EF-hand calcium-binding domain-containing protein 4A isoform X2
2.43E-18	PNPLA2; patatin-like phospholipase domain-containing protein 2
3.60E-09	LRRC27; p53-induced death domain-containing protein 1
3.48E-07	RNH1; ribonuclease inhibitor isoform X2
1.02E-38	CHKA; choline kinase alpha isoform X2
0.000103	LRP5; low-density lipoprotein receptor-related protein 5 isoform X1
6.23E-05	TESMIN; tesmin isoform X2
9.87E-38	CPT1A; carnitine O-palmitoyltransferase 1, liver isoform isoform X2
7.67E-05	SMTNL1; smoothelin-like protein 1
5.79E-10	PGR2/3; P2X purinoceptor 3 precursor
3.77E-20	SMIM38; small integral membrane protein 38
#######	FGF19; fibroblast growth factor 19 precursor
5.31E-08	HSD17B12; very-long-chain 3-oxoacyl-CoA reductase
9.38E-11	MDK; midkine isoform X1
5.64E-05	DLL4; delta-like protein 4 isoform X2
1.61E-25	CHAC1; glutathione-specific gamma-glutamylcyclotransferase 1
2.85E-07	ITPKA; inositol-trisphosphate 3-kinase A
4.59E-07	LTK; leukocyte tyrosine kinase receptor isoform X1

7.40E-82 SPTBN5; spectrin beta chain, non-erythrocytic 5
1.87E-08 EHD4; EH domain-containing protein 4
4.40E-18 PAPLN; papilin isoform X2
9.93E-05 PSEN1; presenilin-1 isoform X2
1.19E-27 TTC9; tetratricopeptide repeat protein 9A
0.000303 PLEKHD1; pleckstrin homology domain-containing family D member 1 isoform X4
3.44E-14 RDH11; retinol dehydrogenase 12
1.16E-10 LGALSL2; galectin-related protein A isoform X3
2.33E-12 BMF; bcl-2-modifying factor isoform X2
2.24E-06 LOC112532477; fibrinogen-like protein 1-like protein
4.62E-07 DTD2; D-aminoacyl-tRNA deacylase 2 isoform X3
9.71E-07 LOC112532533; uncharacterized protein LOC112532533
1.59E-07 SLC25A21; mitochondrial 2-oxodicarboxylate carrier isoform X8
1.15E-21 TTC6; tetratricopeptide repeat protein 6 isoform X3
5.20E-05 CLEC14A; C-type lectin domain family 14 member A
1.20E-50 ACSS1B; acetyl-coenzyme A synthetase 2-like, mitochondrial isoform X2
1.87E-08 FOS; proto-oncogene c-Fos
1.01E-21 FLVCR2; feline leukemia virus subgroup C receptor-related protein 2 isoform X2
3.00E-05 LOC112532559; translation initiation factor IF-2-like isoform X1
3.63E-10 GALC; galactocerebrosidase isoform X2
6.09E-05 GPR65; psychosine receptor
5.38E-12 TRIP11; thyroid receptor-interacting protein 11
4.82E-07 SPIA9; alpha-1-antitrypsin isoform X2
1.41E-10 SPIA3; alpha-1-antiproteinase isoform X1
5.68E-11 LOC107049127; serpin A3-4-like isoform X1
6.47E-09 BDKRB1; B1 bradykinin receptor
1.93E-05 LOC121110856; uncharacterized protein LOC121110856
3.26E-07 DIO3; thyroxine 5-deiodinase
9.26E-71 ANKRD9; ankyrin repeat domain-containing protein 9
9.36E-24 AMN; protein amnionless isoform X1
5.73E-05 ASPG; 60 kDa lysophospholipase isoform X2
0.000122 ZBTB42; zinc finger and BTB domain-containing protein 42
0.000191 SYNE2; nesprin-2 isoform X1
7.73E-18 DHRS7; dehydrogenase/reductase SDR family member 7
1.88E-09 DLGAP5; disks large-associated protein 5
1.87E-10 GCH1; GTP cyclohydrolase 1
5.50E-05 DNAAF2; protein kintoun
1.64E-06 SOS2; son of sevenless homolog 2 isoform X2

0.000143	CDKL1; cyclin-dependent kinase-like 1
2.42E-11	ABHD12B; protein ABHD12B isoform X1
9.74E-11	LOC107051813; prostaglandin D2 receptor-like
3.46E-11	LOC112532663; uncharacterized protein LOC112532663 isoform X1
1.68E-05	LOC121106456; protein MANBAL-like
8.49E-11	GRID1; glutamate receptor ionotropic, delta-1 isoform X3
4.80E-20	SNCG; gamma-synuclein
1.68E-05	SYT15; synaptotagmin-15 isoform X6
7.49E-06	MAT1A; S-adenosylmethionine synthase isoform type-1
0.000115	IPMK; inositol polyphosphate multikinase
6.18E-05	SIRT1; NAD-dependent protein deacetylase sirtuin-1
1.93E-27	DNAJC12; dnaJ homolog subfamily C member 12
0.001263	LOC101748577; E3 SUMO-protein ligase EGR2
0.000266	PAPSS2; bifunctional 3'-phosphoadenosine 5'-phosphosulfate synthase 2 isoform X3
0.000242	MINPP1; multiple inositol polyphosphate phosphatase 1 precursor
7.98E-08	ASAH2; LOW QUALITY PROTEIN: putative neutral ceramidase C isoform X1
1.58E-15	PRKG1; cGMP-dependent protein kinase 1 isoform X3
#######	HKDC1; hexokinase HKDC1 isoform X3
5.80E-14	DUSP13; dual specificity protein phosphatase 13
0.000822	NTL; neurotrypsin isoform X1
0.000113	FUT11; alpha-(1,3)-fucosyltransferase 11 precursor
3.22E-31	RP11-400G3.5; cytochrome P450 2C9-like
8.26E-57	CYP2C18; cytochrome P450 family 2 subfamily C member 18
3.64E-12	TLL2; tolloid-like protein 2 isoform X6
8.20E-08	TMEM150B; transmembrane protein 150A isoform X1
1.39E-65	SCD; stearoyl-CoA desaturase
4.45E-26	CYP2C23b; cytochrome P450 2H2 precursor
9.58E-18	CYP2C23a; cytochrome P450 2H1 precursor
3.95E-19	MSMB; beta-microseminoprotein
2.31E-05	WDFY4; WD repeat- and FYVE domain-containing protein 4 isoform X2
1.55E-68	LOC112532694; bone morphogenetic protein 7-like
3.22E-16	ANKRD22; ankyrin repeat domain-containing protein 22 isoform X1
7.84E-09	ACTA2; actin, aortic smooth muscle isoform X1
0.000348	CH25H; cholesterol 25-hydroxylase
7.58E-05	LIPA; lysosomal acid lipase/cholesteryl ester hydrolase
1.84E-16	PANK1; pantothenate kinase 1 isoform X3
8.72E-09	PPP1R3C; protein phosphatase 1 regulatory subunit 3C
0.000908	MYOF; myoferlin isoform X13

5.65E-08 CEP55; centrosomal protein of 55 kDa isoform X2
7.37E-10 ENTPD7; ectonucleoside triphosphate diphosphohydrolase 7
1.24E-07 PYROXD2; pyridine nucleotide-disulfide oxidoreductase domain-containing protein 2
6.72E-13 LOXL4; lysyl oxidase homolog 4 precursor
1.73E-49 CRTAC1; cartilage acidic protein 1
9.82E-07 PI4K2A; LOW QUALITY PROTEIN: phosphatidylinositol 4-kinase type 2-alpha
4.15E-25 HOGA1; 4-hydroxy-2-oxoglutarate aldolase, mitochondrial
0.005618 ANKRD2; ankyrin repeat domain-containing protein 2 isoform X2
8.19E-26 TLX1; T-cell leukemia homeobox protein 1
3.61E-05 PDZD7; PDZ domain-containing protein 7 isoform X4
0.000496 WBP1L; WW domain binding protein 1-like isoform X1
0.001249 NEURL1; E3 ubiquitin-protein ligase NEURL1 isoform X5
4.45E-10 GSTO2; glutathione S-transferase omega-1
0.000188 SHOC2; leucine-rich repeat protein SHOC-2
1.75E-39 GPAM; glycerol-3-phosphate acyltransferase 1, mitochondrial isoform X1
9.61E-05 ABLIM1; actin-binding LIM protein 1 isoform X27
9.31E-05 HTRA1; serine protease HTRA1
5.79E-09 LOC101750892; deleted in malignant brain tumors 1 protein-like isoform X2
2.06E-32 FAM196A; inhibitory synaptic factor 2A isoform X4
3.80E-11 MKI67; proliferation marker protein Ki-67 isoform X4
1.32E-09 ALDH18A1; delta-1-pyrroline-5-carboxylate synthase isoform X7
0.00037 C2orf88; small membrane A-kinase anchor protein isoform X1
2.43E-06 FRZB; secreted frizzled-related protein 3 precursor
1.88E-74 ABCA12; ATP-binding cassette sub-family A member 12 isoform X2
3.84E-28 ATIC; bifunctional purine biosynthesis protein ATIC
3.03E-23 LOC107048987; putative methyltransferase DDB_G0268948
6.18E-05 RAB17; ras-related protein Rab-17 isoform X1
0.000131 UGT1A1; UDP-glucuronosyltransferase 1A1 isoform X4
2.84E-59 LOC121111295; UDP-glucuronosyltransferase 1A1-like
8.17E-11 AHR2; aryl hydrocarbon receptor 2 isoform X4
6.08E-20 AHR1B; aryl hydrocarbon receptor 1 beta isoform X2
5.44E-06 SLC19A1; reduced folate transporter isoform X2
2.94E-13 COL6A2; collagen alpha-2(VI) chain precursor
3.51E-11 LSS; lanosterol synthase
2.32E-08 LOC768589; baculoviral IAP repeat-containing protein 5.1 isoform X1
2.01E-31 COQ10B; coenzyme Q-binding protein COQ10 homolog B, mitochondrial
4.51E-07 GPR1; G-protein coupled receptor 1 isoform X2
2.32E-05 RAPH1; ras-associated and pleckstrin homology domains-containing protein 1 isoform X4

7.32E-08 LOC424111; uncharacterized protein LOC424111
8.55E-12 IDH1; isocitrate dehydrogenase [NADP] cytoplasmic isoform X2
1.58E-11 PIKFYVE; 1-phosphatidylinositol 3-phosphate 5-kinase isoform X6
9.20E-09 CDCA7; cell division cycle-associated protein 7 isoform X2
2.91E-33 RAPGEF4; rap guanine nucleotide exchange factor 4 isoform X5
1.49E-05 ITGA6; integrin alpha-6 precursor
7.83E-05 SLC25A12; calcium-binding mitochondrial carrier protein Aralar1 isoform X4
3.66E-06 G6PC2; glucose-6-phosphatase 2 isoform X5
4.09E-06 SPC25; kinetochore protein Spc25
1.78E-07 CERS6; ceramide synthase 6 isoform X2
1.75E-05 FAP; prolyl endopeptidase FAP
3.78E-19 DPP4; dipeptidyl peptidase 4
1.21E-24 SLC4A10; sodium-driven chloride bicarbonate exchanger isoform X6
3.11E-25 ITGB6; integrin beta-6 isoform X1
1.47E-20 LOC424199; uncharacterized protein LOC424199 isoform X1
3.01E-05 TUB4A; tubulin alpha-5 chain isoform X2
9.88E-07 IHH; indian hedgehog protein precursor
1.54E-08 MNR2; motor neuron and pancreas homeobox protein 1
3.63E-05 PLCD4; 1-phosphatidylinositol 4,5-bisphosphate phosphodiesterase delta-4 isoform X7
1.06E-06 SLC11A1; natural resistance-associated macrophage protein 1 isoform X4
2.34E-76 IGFBP2; insulin-like growth factor-binding protein 2 isoform X1
4.16E-13 ARHGEF1; uncharacterized protein ARHGEF1 isoform X3
9.25E-09 SEMA5B; semaphorin-5B isoform X5
4.39E-08 MYLK; myosin light chain kinase, smooth muscle isoform X3
4.11E-12 SLC12A8; solute carrier family 12 member 8 isoform X1
0.000136 GPD1L2; glycerol-3-phosphate dehydrogenase 1-like protein
2.11E-14 DPP10; inactive dipeptidyl peptidase 10 isoform X2
9.38E-09 CXCR4; C-X-C chemokine receptor type 4
7.75E-07 NXPH2; neurexophilin-2 isoform X1
0.000192 UPP2; uridine phosphorylase 2 isoform X2
8.93E-06 DAPL1; death-associated protein-like 1
1.27E-06 NTNG1; netrin-G1 isoform X5
5.96E-10 RGS2; regulator of G-protein signaling 2
8.27E-09 HTATIP2; oxidoreductase HTATIP2 isoform X1
3.00E-17 HSD17B7; 3-keto-steroid reductase/17-beta-hydroxysteroid dehydrogenase 7 isoform 2
1.74E-05 SPATA46; spermatogenesis-associated protein 46 isoform X1
9.25E-07 REG4; regenerating islet-derived protein 4 precursor
9.34E-05 LOC121111405; phospholipid phosphatase 6-like

7.65E-18 FASLG; tumor necrosis factor ligand superfamily member 6 isoform X1
3.31E-33 SUCO; SUN domain-containing ossification factor isoform X5
5.58E-05 FMO3; flavin containing monooxygenase 3
6.45E-08 RGS8; regulator of G-protein signaling 8 isoform X2
0.000104 RGSL1; regulator of G-protein signaling protein-like isoform X1
1.86E-12 SOAT1; sterol O-acyltransferase 1 isoform X4
4.53E-11 ABL2; tyrosine-protein kinase ABL2 isoform X2
6.97E-37 TOR3A; torsin-3A
4.97E-08 LOC424430; quinone oxidoreductase-like protein 2
3.66E-09 KIAA0040; uncharacterized protein KIAA0040 homolog
2.76E-05 RABGAP1L; rab GTPase-activating protein 1-like isoform X8
0.000166 SERPINC1; antithrombin-III precursor
1.11E-09 LRRC39; leucine-rich repeat-containing protein 39 isoform X2
4.12E-09 TRMT13; tRNA:m(4)X modification enzyme TRM13 homolog
4.66E-10 DPYD; dihydropyrimidine dehydrogenase [NADP(+)] isoform X3
6.15E-05 ALG14; UDP-N-acetylglucosamine transferase subunit ALG14 homolog
2.73E-06 LOC424491; very-long-chain enoyl-CoA reductase isoform X3
0.0002 ZNF644; zinc finger protein 644 isoform X3
3.20E-07 CYR61; CCN family member 1 precursor
1.24E-08 LPAR3; lysophosphatidic acid receptor 3 isoform X1
5.16E-15 SSX2IP; afadin- and alpha-actinin-binding protein isoform X3
1.22E-23 VTG2; vitellogenin-2 precursor
4.04E-38 LOC121111362; uncharacterized protein LOC121111362 isoform X1
5.92E-28 VTG3; vitellogenin-3
7.02E-08 SPATA1; spermatogenesis-associated protein 1 isoform X3
1.48E-21 VTG1; vitellogenin-1 precursor
0.00027 ARTN; artemin isoform X1
0.000225 MMACHC; cyanocobalamin reductase / alkylcobalamin dealkylase
8.69E-28 RBP; riboflavin-binding protein precursor
1.49E-10 FAAH; fatty-acid amide hydrolase 1
5.30E-10 CYP4A22; cytochrome P450 4B1
4.89E-45 CYP4B7; cytochrome P450 4B7
1.11E-27 PODN; podocan isoform X3
9.01E-07 TCEANC2; transcription elongation factor A N-terminal and central domain-containing protein 2
1.91E-29 DHCR24; delta(24)-sterol reductase
6.76E-11 PCSK9; proprotein convertase subtilisin/kexin type 9
8.40E-06 C8B; complement component C8 beta chain isoform X1
2.45E-13 DAB1; disabled homolog 1

2.94E-07 TACSTD2; tumor-associated calcium signal transducer 2 precursor
7.80E-17 HOOK1; protein Hook homolog 1
2.61E-08 KANK4; KN motif and ankyrin repeat domain-containing protein 4 isoform X1
7.25E-38 ANGPTL3; angiopoietin-related protein 3 precursor
5.10E-09 NGEF; ephexin-1 isoform X3
4.10E-07 NEU2; sialidase-2 isoform X3
5.40E-16 KLHL24; kelch-like protein 24 isoform X1
4.61E-30 EHHADH; peroxisomal bifunctional enzyme isoform X2
9.15E-12 DNAJB11; dnaJ homolog subfamily B member 11
2.48E-05 RNF168; E3 ubiquitin-protein ligase RNF168
1.36E-06 PTTG1IP; pituitary tumor-transforming gene 1 protein-interacting protein
1.78E-06 GPR35; G-protein coupled receptor 35
4.99E-06 PER2; period circadian protein homolog 2 isoform X3
2.11E-08 CLSTN2; calsyntenin-2 isoform X2
2.79E-13 PXYLP1; 2-phosphoxylose phosphatase 1 isoform X1
1.02E-10 EPHA4; ephrin type-A receptor 4 isoform X5
2.21E-38 SGPP2; sphingosine-1-phosphate phosphatase 2
1.25E-05 MOGAT1; 2-acylglycerol O-acyltransferase 1
0.000114 CCL20; C-C motif chemokine 20 precursor
2.48E-07 PID1; PTB-containing, cubilin and LRP1-interacting protein
7.34E-05 TFDP2; transcription factor Dp-2 isoform X2
4.57E-08 GK5; putative glycerol kinase 5
1.83E-25 PCOLCE2; procollagen C-endopeptidase enhancer 2 isoform X1
0.000195 PAQR9; membrane progestin receptor epsilon
1.94E-12 PLOD2; procollagen-lysine,2-oxoglutarate 5-dioxygenase 2 isoform X2
2.76E-08 HRASLS; phospholipase A and acyltransferase 1
1.28E-16 RTP2; receptor-transporting protein 2-like
1.45E-07 PDCD1; programmed cell death protein 1
1.76E-05 C2orf72; uncharacterized protein C2orf72 homolog
0.000155 PSMD1; 26S proteasome non-ATPase regulatory subunit 1
1.62E-13 NPPC; C-type natriuretic peptide
3.47E-41 ALPI; intestinal-type alkaline phosphatase
1.79E-05 ECE2; endothelin-converting enzyme 2 isoform X3
5.02E-22 AHSG; alpha-2-HS-glycoprotein
1.38E-05 TNFSF10; tumor necrosis factor ligand superfamily member 10 isoform X4
5.16E-06 MECOM; histone-lysine N-methyltransferase MECOM isoform X15
0.000653 LOC107054133; SLIT and NTRK-like protein 3
2.38E-10 SMC4; structural maintenance of chromosomes protein 4

4.30E-31	IFT80; intraflagellar transport protein 80 homolog
4.39E-23	MLF1; myeloid leukemia factor 1 isoform X1
9.23E-10	C3orf33; protein C3orf33 homolog
1.55E-08	ARHGEF26; rho guanine nucleotide exchange factor 26
6.09E-20	SUCNR1; succinate receptor 1 isoform X3
4.72E-08	SERP1; stress-associated endoplasmic reticulum protein 1
7.49E-07	SHF; SH2 domain-containing adapter protein F isoform X1
1.12E-07	SLC28A2; sodium/nucleoside cotransporter 1
#######	DUOX1; dual oxidase 2
1.87E-27	KIAA0101; PCNA-associated factor
4.05E-09	PATL2; protein PAT1 homolog 2 isoform X1
5.15E-07	CD276; CD276 antigen isoform X1
2.61E-10	CYP1A1; cytochrome P450 1A4
4.26E-08	CHRNA3; neuronal acetylcholine receptor subunit alpha-3 precursor
8.35E-06	CHRNA5; neuronal acetylcholine receptor subunit alpha-5 isoform X1
1.05E-07	LOC101749938; uncharacterized protein LOC101749938
2.78E-05	RORA; nuclear receptor ROR-alpha isoform X4
1.80E-49	TRPM1; transient receptor potential cation channel subfamily M member 1 isoform X5
6.44E-23	LIPC; hepatic triacylglycerol lipase isoform X1
0.000213	ALDH1A2; retinal dehydrogenase 2 isoform 2
0.000215	DYX1C1; dynein assembly factor 4, axonemal
1.37E-30	SEMA6D; semaphorin-6D isoform X8
2.13E-08	FBN1; fibrillin-1 isoform X2
3.52E-05	HDC; histidine decarboxylase isoform X2
6.85E-10	GATM; glycine amidinotransferase, mitochondrial
1.81E-11	HOMER2; homer protein homolog 2
2.81E-07	LOC415472; uncharacterized protein LOC415472
1.55E-17	PEX11A; peroxisomal membrane protein 11A isoform X2
8.06E-15	MFGE8; lactadherin isoform 1 precursor
2.20E-09	HAPLN3; hyaluronan and proteoglycan link protein 3
1.41E-24	PGPEP1L; pyroglutamyl-peptidase 1-like protein isoform X1
9.08E-08	ADAMTS17; A disintegrin and metalloproteinase with thrombospondin motifs 17 isoform X3
3.14E-35	ALDH1A3; aldehyde dehydrogenase family 1 member A3
1.65E-05	MTFMT; methionyl-tRNA formyltransferase, mitochondrial isoform X1
9.88E-09	ZWILCH; protein zwilch homolog
6.43E-07	CORO2B; coronin-2B isoform X3
7.75E-08	PAQR5; membrane progestin receptor gamma
1.06E-15	KIF23; kinesin-like protein KIF23 isoform X18

3.08E-05	UACA; uveal autoantigen with coiled-coil domains and ankyrin repeats
4.10E-05	FAM96A; cytosolic iron-sulfur assembly component 2A
2.93E-16	TMED3; transmembrane emp24 domain-containing protein 3 isoform 1 precursor
1.30E-05	PLA2G15; phospholipase A2 group XV
7.81E-05	ADGRG1; adhesion G-protein coupled receptor G1
0.000333	ENKD1; enkurin domain-containing protein 1 isoform X1
#######	CETP; cholesteryl ester transfer protein precursor
0.000321	CPNE2; copine-2
2.62E-13	CX3CL1; fractalkine precursor
0.000125	EXOC3L1; exocyst complex component 3-like protein
0.00025	KIAA0895L; uncharacterized protein KIAA0895-like homolog isoform X3
8.42E-40	LCAT; phosphatidylcholine-sterol acyltransferase precursor
4.10E-31	LOC415662; C-factor-like isoform X3
9.89E-22	LOC101747680; C-factor-like
5.13E-05	LOC112533303; C-factor-like
5.54E-06	LOC415664; uncharacterized oxidoreductase-like isoform X1
6.22E-05	LOC100857820; C-factor
4.83E-05	LOC101748539; uncharacterized oxidoreductase C663.09c-like
2.80E-05	PDPR; pyruvate dehydrogenase phosphatase regulatory subunit, mitochondrial
1.95E-12	MLKL; mixed lineage kinase domain-like protein
3.64E-07	BCAR1; breast cancer anti-estrogen resistance protein 1 isoform X2
3.17E-24	MT4; metallothionein
1.50E-20	MT3; metallothionein-3
8.64E-09	GALR1L; galanin receptor type 1-like isoform X2
1.88E-10	SLC6A2; sodium-dependent noradrenaline transporter
4.81E-08	TOX3; TOX high mobility group box family member 3 isoform 1
0.000179	SNX20; sorting nexin-20 isoform X1
8.77E-23	CDCA9; borealin-2
3.09E-07	RGS9BP; regulator of G-protein signaling 9-binding protein
0.000221	FAAP24; Fanconi anemia core complex-associated protein 24 isoform X1
0.000143	RHPN2; rhophilin-2 isoform X1
1.60E-08	LOC121111686; translation initiation factor IF-2-like isoform X1
1.82E-08	CEBPA; CCAAT/enhancer-binding protein alpha
6.10E-09	CEBPG; CCAAT/enhancer-binding protein gamma
2.07E-05	PDCD2L; programmed cell death protein 2-like
6.50E-08	LOC769704; fatty acyl-CoA hydrolase precursor, medium chain isoform X1
6.46E-08	CES1L2; fatty acyl-CoA hydrolase precursor, medium chain
1.37E-11	TERB1; telomere repeats-binding bouquet formation protein 1 isoform X3

0.00056	DYNLRB2; dynein light chain roadblock-type 2
1.68E-06	CMC2; COX assembly mitochondrial protein 2 homolog isoform X2
1.64E-08	CENPN; centromere protein N
4.19E-15	CDH13; cadherin-13 isoform X6
1.08E-09	CRISPLD2; cysteine-rich secretory protein LCCL domain-containing 2 isoform X1
4.66E-09	SLC7A5; large neutral amino acids transporter small subunit 1
5.45E-35	CA5A; carbonic anhydrase 5A, mitochondrial isoform X2
1.06E-06	CIDEC; cell death activator CIDE-3 isoform X1
6.48E-13	MVD; diphosphomevalonate decarboxylase
2.05E-09	PIEZO1; piezo-type mechanosensitive ion channel component 1 isoform X2
2.62E-07	CDT1; DNA replication factor Cdt1 isoform X1
1.26E-21	SPIRE2; protein spire homolog 2 isoform X1
2.72E-07	CDH3; B-cadherin
7.97E-09	LOC121111704; uncharacterized protein LOC121111704
3.13E-05	CHST4; carbohydrate sulfotransferase 4
2.09E-28	LOC121106611; uncharacterized protein LOC121106611 isoform X2
2.35E-13	NT5DC2; 5'-nucleotidase domain-containing protein 2 isoform X1
3.34E-10	SMIM4; small integral membrane protein 4
5.19E-11	ITIH3; inter-alpha-trypsin inhibitor heavy chain H3 isoform X3
5.39E-11	MUSTN1; musculoskeletal embryonic nuclear protein 1
0.000211	PFKFB4; 6-phosphofructo-2-kinase/fructose-2, 6-bisphosphatase 4 isoform X9
1.10E-05	MAPKAPK3; MAP kinase-activated protein kinase 3 isoform X1
7.17E-12	MANF; mesencephalic astrocyte-derived neurotrophic factor isoform X2
8.62E-09	LOC121106469; guanylate-binding protein 2-like isoform X2
2.59E-25	LOC121106623; guanylate-binding protein 1-like
2.00E-05	LOC121106627; guanylate-binding protein 1-like
5.55E-07	GBP; guanylate binding protein
2.57E-14	GMPPB; mannose-1-phosphate guanyltransferase beta
8.91E-06	LOC770794; acylamino-acid-releasing enzyme isoform X1
4.74E-30	MST1R; macrophage-stimulating protein receptor isoform X1
1.67E-09	USP4; ubiquitin carboxyl-terminal hydrolase 4
5.53E-05	GPX1; glutathione peroxidase 1
1.21E-07	PCBP4; poly(rC)-binding protein 4 isoform X3
2.78E-07	OASL; 59 kDa 2'-5'-oligoadenylate synthase-like protein
3.65E-12	ABHD6; monoacylglycerol lipase ABHD6 isoform X2
2.55E-07	IP6K2; inositol hexakisphosphate kinase 2 isoform X1
7.86E-05	TXNRD3; thioredoxin reductase 3
5.49E-06	CFAP100; cilia- and flagella-associated protein 100 isoform X1

3.80E-06	P4HTM; transmembrane prolyl 4-hydroxylase
1.54E-44	C3orf67; protein CFAP20DC isoform X1
9.43E-07	PTPRG; receptor-type tyrosine-protein phosphatase gamma precursor
1.49E-06	LRIG1; leucine-rich repeats and immunoglobulin-like domains protein 1 isoform X1
4.16E-05	FOXP1; forkhead box protein P1 isoform X7
3.09E-43	GPR27; probable G-protein coupled receptor 27
3.42E-60	PROK2; prokineticin-2
0.000343	GHRL; appetite-regulating hormone preproprotein
2.43E-06	HBEGF; proheparin-binding EGF-like growth factor precursor
6.18E-05	PCDH1; protocadherin-1 precursor
#######	TENM2; teneurin-2 isoform 2
1.57E-39	GABRB2; gamma-aminobutyric acid receptor subunit beta-2 isoform X2
1.45E-06	PTTG2; securin isoform 1
1.10E-06	IL12B; interleukin-12 subunit beta precursor
4.22E-23	SLC26A2; sulfate transporter isoform X1
2.85E-06	PPARGC1B; peroxisome proliferator-activated receptor gamma coactivator 1-beta isoform X4
0.000402	ARHGEF37; rho guanine nucleotide exchange factor 37 isoform X3
0.000146	DUSP1; dual specificity protein phosphatase 1
1.28E-19	STC2; stanniocalcin-2
0.000223	SFXN1; sideroflexin-1 isoform X2
5.22E-22	ARL2; ADP-ribosylation factor-like protein 3 isoform X8
8.18E-10	MXD3; lateral signaling target protein 2 homolog isoform X3
8.84E-06	NIPAL4; magnesium transporter NIPA4 isoform X1
2.72E-07	CYFIP2; cytoplasmic FMR1-interacting protein 2 isoform X1
1.75E-25	GRIA1; glutamate receptor 1 precursor
1.48E-05	LTC4S; leukotriene C4 synthase
8.12E-15	RASGEF1C; ras-GEF domain-containing family member 1C
5.13E-31	TRPC7; short transient receptor potential channel 7 isoform X1
1.99E-23	LOC101749540; small integral membrane protein 32
4.53E-15	LECT2; myeloid protein 1 precursor
0.0006	MEIKIN; meiosis-specific kinetochore protein isoform X3
7.66E-27	SLC22A5; solute carrier family 22 member 5
2.30E-22	LEAP2; liver-expressed antimicrobial peptide 2 precursor
9.76E-15	YIPF5; protein YIPF5
3.10E-43	GFRA3; GDNF family receptor alpha-3
0.000154	CTBPL; C-terminal binding protein-like
2.69E-10	EGR1; early growth response protein 1
1.65E-33	BHLHA15; class A basic helix-loop-helix protein 15

2.96E-06	BAIAP2L1; brain-specific angiogenesis inhibitor 1-associated protein 2-like protein 1
2.44E-20	NPTX2; neuronal pentraxin-2 isoform X1
0.000294	PDGFA; platelet-derived growth factor subunit A preproprotein
2.53E-05	SUN1; SUN domain-containing protein 1 isoform X17
2.46E-08	CYP3A5; cytochrome P450 family 3 subfamily A member 5
4.61E-07	FOXK1; forkhead box protein K1 isoform X1
3.73E-06	FBXL18; F-box/LRR-repeat protein 18 isoform X5
2.52E-34	LOC101747873; urotensin-2 receptor
6.36E-09	PEMT; phosphatidylethanolamine N-methyltransferase isoform X2
1.43E-08	LOC427665; chemokine-like receptor 1 isoform X1
1.24E-34	CACNA1H; voltage-dependent T-type calcium channel subunit alpha-1H isoform X2
1.31E-08	SOX8; transcription factor SOX-8
0.00011	MEIOB; meiosis-specific with OB domain-containing protein isoform X1
3.48E-08	NOXO1; NADPH oxidase organizer 1
1.90E-08	PGP; glycerol-3-phosphate phosphatase
6.37E-06	EEF2K; eukaryotic elongation factor 2 kinase isoform X4
1.63E-13	CDR2; cerebellar degeneration-related protein 2
3.36E-06	IGSF6; immunoglobulin superfamily member 6 isoform X1
1.94E-07	KDELR2; ER lumen protein-retaining receptor 2
2.20E-15	RMI2; recQ-mediated genome instability protein 2
4.60E-08	SOCS1; suppressor of cytokine signaling 1
2.03E-05	EMP2; epithelial membrane protein 2
2.65E-99	GRIN2A; glutamate receptor ionotropic, NMDA 2A isoform X5
4.00E-21	RHBDF1; inactive rhomboid protein 1 isoform X2
2.00E-37	HBAD; hemoglobin subunit alpha-D
1.28E-28	HBA1; hemoglobin subunit alpha-A
5.00E-43	PDIA2; protein disulfide-isomerase A2
7.53E-07	FAM234A; protein FAM234A
#######	LOC416655; protein PERCC1 isoform X1
0.000429	UBALD1; UBA-like domain-containing protein 1
2.19E-08	C16orf96; uncharacterized protein C16orf96 homolog isoform X3
9.82E-05	NME4; nucleoside diphosphate kinase, mitochondrial
1.60E-08	DECR2; peroxisomal 2,4-dienoyl-CoA reductase [(3E)-enoyl-CoA-producing] isoform X1
9.79E-05	MCRIP2; MAPK regulated corepressor interacting protein 2 isoform X1
7.49E-05	ROGDI; protein rogdi homolog isoform X1
8.88E-08	GPRC5B; G-protein coupled receptor family C group 5 member B isoform X2
1.90E-12	SDF2L1; stromal cell-derived factor 2-like protein 1
2.31E-06	YPEL1; protein yippee-like 1

5.70E-06 C22orf39; UPF0545 protein C22orf39 homolog
7.30E-15 CDC45; cell division control protein 45 homolog isoform X2
4.35E-07 CLDN5; claudin-5
4.61E-12 COMT; catechol O-methyltransferase
6.45E-08 ULK1; serine/threonine-protein kinase ULK1 isoform X1
2.32E-07 RIMBP2; RIMS-binding protein 2 isoform X14
4.83E-33 TMEM132B; transmembrane protein 132B isoform X5
9.16E-48 AACS; acetoacetyl-CoA synthetase
2.62E-07 SNRNP35; U11/U12 small nuclear ribonucleoprotein 35 kDa protein
2.17E-10 ATP2A2; sarcoplasmic/endoplasmic reticulum calcium ATPase 2 isoform 2
2.40E-30 RAD9B; cell cycle checkpoint control protein RAD9B isoform X2
7.77E-12 PPTC7; protein phosphatase PTC7 homolog
0.000112 TCTN1; tectonic-1 isoform X2
1.28E-15 TMEM116; transmembrane protein 116 isoform X3
4.76E-32 UNG; uracil-DNA glycosylase
1.20E-91 ACACB; acetyl-CoA carboxylase 2 isoform X5
2.94E-07 MVK; mevalonate kinase
6.35E-29 FAM222A; protein FAM222A isoform X3
1.56E-05 CRYBB3; beta-crystallin B3
3.10E-18 VPS29L; vacuolar protein sorting 29 homolog
1.63E-06 LOC769646; uncharacterized protein LOC769646 isoform X4
2.51E-05 CHCHD10; coiled-coil-helix-coiled-coil-helix domain-containing protein 10, mitochondrial
0.000119 SLC2A11; solute carrier family 2, facilitated glucose transporter member 11
8.64E-21 SLC2A11L1; solute carrier family 2, facilitated glucose transporter member 11-like
9.75E-16 LOC769554; solute carrier family 2, facilitated glucose transporter member 11
1.39E-07 GSTT1L; glutathione S-transferase theta-1 isoform X1
4.93E-13 TBX6; T-box-containing protein TBX6L isoform X1
5.86E-23 LOC416959; melanotransferrin isoform X2
1.53E-24 PISD; phosphatidylserine decarboxylase proenzyme, mitochondrial isoform X1
1.22E-06 PIK3IP1; phosphoinositide-3-kinase-interacting protein 1
1.18E-06 C12orf43; protein CUSTOS isoform X1
0.001033 LOC107051583; uncharacterized protein LOC107051583 isoform X1
1.03E-05 LOC100858984; dynein light chain 2, cytoplasmic
1.65E-06 CFAP73; cilia- and flagella-associated protein 73 isoform X1
1.84E-12 SELENOM; selenoprotein M precursor
5.55E-13 PLA2G3; group 3 secretory phospholipase A2 isoform X1
2.55E-09 PRODH; proline dehydrogenase 1, mitochondrial isoform X1
5.61E-64 SLC35E4; solute carrier family 35 member E4

1.62E-34 TCN2; transcobalamin-2
2.36E-15 HORMAD2; HORMA domain-containing protein 2 isoform X9
1.20E-26 LOC417013; acyl-CoA dehydrogenase family member 11 isoform X2
6.10E-07 MED13L; mediator of RNA polymerase II transcription subunit 13-like
1.90E-07 LOC107049644; rho GTPase-activating protein 32-like
1.00E-14 LOC112531211; uncharacterized protein LOC112531211 isoform X1
0.000126 LOC121106953; uncharacterized protein LOC121106953 isoform X1
3.76E-09 MHCY2; LOW QUALITY PROTEIN: class I histocompatibility antigen, F10 alpha chain
7.63E-06 MHCY14; major histocompatibility complex-Y, class I heavy chain, 14 precursor
2.24E-19 LOC121106942; C-type lectin domain family 2 member I-like isoform X5
8.62E-12 MHCY9; major histocompatibility complex Y, class I heavy chain 9 isoform X3
9.05E-56 LENG9L7; leukocyte receptor cluster member 9-like, MHC-Y region, 7
9.35E-40 MHCY15; major histocompatibility complex-Y, class I heavy chain, 15 precursor
5.39E-20 MHCY7; major histocompatibility complex-Y, class I heavy chain, 7 precursor
0.000288 MHCY4; class I histocompatibility antigen, F10 alpha chain isoform X5
1.09E-13 LOC100859628; class I histocompatibility antigen, F10 alpha chain-like isoform X6
3.98E-12 ZNFY1; zinc finger, MHC-Y region, 1
6.51E-05 OZFL; OZF like, MHCY region
4.86E-12 ZNFY3; MHCY region zinc finger protein 3 isoform X2
0.000106 LOC121106918; class I histocompatibility antigen, F10 alpha chain-like isoform X2
1.93E-22 LOC121106502; C-type lectin domain family 2 member I-like isoform X5
4.61E-25 LOC121106943; C-type lectin domain family 2 member E-like isoform X1
3.59E-24 LOC121106928; class I histocompatibility antigen, F10 alpha chain-like isoform X1
1.10E-21 MHCY8; major histocompatibility complex Y, class I heavy chain 8 isoform X2
1.04E-09 MHCY6; LOW QUALITY PROTEIN: major histocompatibility complex Y, class I heavy chain 6
7.16E-08 MOGL; erythroid membrane-associated protein-like
3.33E-48 LOC121106920; uncharacterized protein LOC121106920 isoform X18
3.27E-33 KIFC1; kinesin-like protein KIFC1 isoform X2
2.90E-05 LOC107054697; C-type lectin domain family 2 member D-like isoform X1
1.99E-13 ZNF692; zinc finger protein 692
7.60E-17 IL4I1; L-amino-acid oxidase precursor
1.65E-20 TRIM7.1; tripartite motif-containing protein 7 isoform X5
0.000644 TRIM27.1; tripartite motif-containing 27 isoform X1
4.59E-06 _
2.50E-08 BLB1; Major histocompatibility complex class II beta chain BLB1, (similar to HLA class II, D beta chain) precursor
0.0276 CENPA; histone H3-like centromeric protein A
3.66E-07 CYP21A1; steroid 21-hydroxylase precursor
1.78E-10 LOC121106935; collagen alpha-1(I) chain-like

0.000112 LOC107049645; uncharacterized protein LOC107049645 isoform X2
3.94E-13 LOC107050152; ATPase SWSAP1
0.000172 LOC112531351; zinc finger CCCH domain-containing protein 11A-like isoform X4
1.44E-09 MAN1B1; endoplasmic reticulum mannosyl-oligosaccharide 1,2-alpha-mannosidase isoform X2
1.55E-17 EXFABP; extracellular fatty acid-binding protein precursor
2.71E-10 STPG3; protein STPG3 isoform X1
6.91E-58 NSMF; NMDA receptor synaptonuclear signaling and neuronal migration factor isoform X2
3.30E-07 GSL; glutamine synthetase
7.32E-12 LOC107052201; alpha-1-acid glycoprotein
3.36E-29 SLC25A25; calcium-binding mitochondrial carrier protein SCaMC-2 isoform X4
7.80E-05 ST6GALNAC6; alpha-N-acetylgalactosaminide alpha-2,6-sialyltransferase 6
1.57E-28 FPGS; folylpolyglutamate synthase, mitochondrial isoform X9
8.28E-05 CDK9; cyclin-dependent kinase 9 isoform X1
0.000311 TOR2A; prosalusin isoform X1
0.000254 TTC16; tetratricopeptide repeat protein 16
1.34E-10 CERCAM; inactive glycosyltransferase 25 family member 3 isoform X2
4.93E-07 DNM1; dynamin-1 isoform X8
4.76E-10 PHYHD1; phytanoyl-CoA dioxygenase domain-containing protein 1
6.47E-09 CRAT; carnitine O-acetyltransferase isoform X1
1.18E-09 LAMC3; laminin subunit gamma-3 isoform X3
2.86E-05 ADAMTS13; A disintegrin and metalloproteinase with thrombospondin motifs 13 isoform X3
1.48E-31 FAM69B; divergent protein kinase domain 1B isoform X1
1.14E-56 AGPAT2; 1-acyl-sn-glycerol-3-phosphate acyltransferase beta isoform X2
2.78E-18 LOC417113; carboxyl-terminal PDZ ligand of neuronal nitric oxide synthase protein
2.89E-23 HSPA5; endoplasmic reticulum chaperone BiP isoform X1
8.92E-17 MVB12B; multivesicular body subunit 12B isoform X2
9.82E-07 TMEM220; transmembrane protein 220 isoform X3
7.79E-23 HS3ST3A1; heparan sulfate glucosamine 3-O-sulfotransferase 3A1
2.24E-07 FN3K; fructosamine-3-kinase isoform X2
7.99E-08 NARF; nuclear prelamin A recognition factor
0.000305 ST6GALNAC1; alpha-N-acetylgalactosaminide alpha-2,6-sialyltransferase 1 isoform X1
0.000317 UBALD2; UBA-like domain-containing protein 2
1.01E-31 CDK3; cyclin-dependent kinase 3 isoform X2
1.25E-37 TEN1; CST complex subunit TEN1 isoform X2
9.99E-22 ACOX1; peroxisomal acyl-coenzyme A oxidase 1 isoform X3
5.77E-15 FBF1; fas-binding factor 1 homolog isoform X12
1.07E-07 MRPL38; 39S ribosomal protein L38, mitochondrial
2.34E-09 TRIM65; tripartite motif-containing protein 65

6.71E-09 TRIM47; E3 ubiquitin-protein ligase TRIM47
1.33E-08 CCDC57; coiled-coil domain-containing protein 57 isoform X9
3.28E-32 FASN; fatty acid synthase
4.09E-05 NOTUM; palmitoleoyl-protein carboxylesterase NOTUM
0.000207 MMD; monocyte to macrophage differentiation factor isoform X1
5.25E-06 PCTP; phosphatidylcholine transfer protein isoform X2
3.53E-06 RGS9; regulator of G-protein signaling 9 isoform X1
3.13E-14 ABCA8; ATP-binding cassette sub-family A member 10
7.17E-40 SDK2; protein sidekick-2 isoform X2
3.84E-28 CBX2; chromobox protein homolog 2
2.71E-33 RBFOX3; RNA binding protein fox-1 homolog 3 isoform X8
1.91E-06 ENPP7; ectonucleotide pyrophosphatase/phosphodiesterase family member 7
7.62E-22 TIMP2; metalloproteinase inhibitor 2 precursor
2.07E-14 USP36; ubiquitin carboxyl-terminal hydrolase 36 isoform X3
3.19E-24 SOCS3; suppressor of cytokine signaling 3
3.25E-06 LOC101747660; probable low-specificity L-threonine aldolase 2
4.02E-20 TK1; thymidine kinase, cytosolic
9.66E-08 P4HB; protein disulfide-isomerase precursor
0.000195 EME1; crossover junction endonuclease EME1 isoform X1
1.32E-13 LOC422106; uncharacterized protein LOC422106 isoform X1
0.000655 KRABZFP; uncharacterized protein LOC769812 precursor
3.82E-17 GRIN2C; glutamate receptor ionotropic, NMDA 2C isoform X3
2.49E-09 OTOP2; proton channel OTOP2
8.32E-10 OTOP3; proton channel OTOP3
6.44E-14 HN1; jupiter microtubule associated homolog 1 isoform 3
6.71E-37 SLC16A5; monocarboxylate transporter 6
7.25E-08 CASP1; caspase-1 isoform X1
1.59E-12 LIMK1; LIM domain kinase 1 isoform X1
1.14E-15 GATSL2; cytosolic arginine sensor for mTORC1 subunit 2 isoform X7
2.10E-12 MIS12; protein MIS12 homolog
1.69E-05 COL26A1; collagen alpha-1(XXVI) chain isoform X9
6.81E-09 TMEM120A; ion channel TACAN
1.10E-06 MMP28; matrix metalloproteinase-28 isoform X1
2.91E-09 LOC121106433; C-C motif chemokine 3-like
0.000168 SLC43A2; large neutral amino acids transporter small subunit 4 isoform X2
1.89E-50 SERPINF1; pigment epithelium-derived factor isoform X1
8.83E-06 SRR; serine racemase isoform X7
5.21E-07 TMEM199; transmembrane protein 199

1.86E-09 SEBOX; homeobox protein SEBOX isoform X1
7.27E-21 SARM1; NAD(+) hydrolase SARM1
1.51E-05 DHRS13; dehydrogenase/reductase SDR family member 13 isoform X1
5.73E-10 CORO6; coronin-6 isoform X2
1.50E-23 TUSC5; trafficking regulator of GLUT4 1
0.000321 PIGL; N-acetylglucosaminyl-phosphatidylinositol de-N-acetylase isoform X1
3.78E-05 SHPK; sedoheptulokinase isoform X2
8.64E-07 TRPV1; transient receptor potential cation channel subfamily V member 1
0.000146 ALDH3A2; aldehyde dehydrogenase family 3 member A2 isoform X1
1.78E-80 DOC2B; double C2-like domain-containing protein beta isoform X1
1.29E-12 MED13; mediator of RNA polymerase II transcription subunit 13 isoform X3
3.71E-19 ACACA; acetyl-CoA carboxylase isoform X1
9.83E-09 C17orf78; uncharacterized protein C17orf78
1.59E-07 TADA2A; transcriptional adapter 2-alpha isoform X1
3.57E-12 DUSP14; dual specificity protein phosphatase 14
4.52E-05 CLUH; clustered mitochondria protein homolog isoform X2
0.000109 MAP1LC3A; microtubule-associated proteins 1A/1B light chain 3A
1.64E-05 ASIP; agouti-signaling protein precursor
7.83E-08 E2F1; transcription factor E2F1 isoform X1
5.66E-75 RBPJL; recombining binding protein suppressor of hairless-like protein isoform X3
1.62E-92 PI3; elafin
3.68E-06 LOC121107299; uncharacterized protein LOC121107299
5.20E-24 EPB42; protein 4.2
9.83E-20 GATA5; transcription factor GATA-5 isoform X1
8.62E-12 SLC17A9; solute carrier family 17 member 9
1.47E-18 TNFRSF6B; tumor necrosis factor receptor superfamily member 6B isoform X1
0.001256 STK35; serine/threonine-protein kinase 35 isoform X1
0.000255 SDCBP2; syndecan binding protein 2
6.13E-13 LOC771972; uncharacterized protein LOC771972 isoform X1
3.91E-38 WFDC2; WAP four-disulfide core domain protein 3 isoform X4
8.20E-37 WFDC8; balbiani ring protein 3 isoform X2
5.05E-18 SPINT4; kunitz-type protease inhibitor 4
1.65E-20 LOC771994; protein qua-1
2.56E-16 UBE2U; ubiquitin-conjugating enzyme E2 C
3.50E-11 RBM38; RNA-binding protein 38
0.000211 GCNT7; beta-1,3-galactosyl-O-glycosyl-glycoprotein beta-1,6-N-acetylglucosaminyltransferase 7
0.00029 PTPN1; tyrosine-protein phosphatase non-receptor type 1 isoform X5
2.54E-21 ESPN; espin isoform X6

0.000127	ACOT7; cytosolic acyl coenzyme A thioester hydrolase isoform X1
4.04E-11	MMP23A; matrix metalloproteinase-23 isoform X2
4.28E-15	MIB2; E3 ubiquitin-protein ligase MIB2
1.61E-57	LOC419409; Golgi integral membrane protein 4 isoform X1
4.89E-06	FNDC10; fibronectin type III domain-containing protein 10
4.34E-35	LOC771069; C-factor isoform X2
7.68E-05	PUSL1; tRNA pseudouridine synthase-like 1 isoform X1
6.34E-10	SLC25A33; solute carrier family 25 member 33 isoform X1
1.79E-06	TMEM201; transmembrane protein 201 isoform X2
6.08E-07	RBP7; retinoid-binding protein 7
3.61E-09	RNF186; E3 ubiquitin-protein ligase RNF186
2.81E-07	DHRS3; short-chain dehydrogenase/reductase 3 isoform X2
0.00011	VPS13D; vacuolar protein sorting-associated protein 13D isoform X1
1.13E-08	MTHFR; methylenetetrahydrofolate reductase
2.16E-09	C1orf167; uncharacterized protein C1orf167 homolog isoform X5
3.91E-17	DRAXIN; draxin isoform X1
5.75E-08	EPHB2; ephrin type-B receptor 2 precursor
2.15E-09	P3H1; prolyl 3-hydroxylase 1 precursor
3.51E-06	ECE1; endothelin-converting enzyme 1 isoform X4
1.61E-07	LOC112530142; uncharacterized protein LOC112530142
5.88E-05	SLC23A2; solute carrier family 23 member 2 isoform X1
5.27E-25	DPYSL2; dihydropyrimidinase-related protein 2 isoform X2
5.16E-07	SLC25A37; mitoferrin-1 isoform X1
2.43E-07	DMTN; dematin isoform X2
5.77E-08	LOC101749127; uncharacterized protein LOC101749127 isoform X1
2.38E-12	ADAM32L2; disintegrin and metalloproteinase domain-containing protein 32
3.38E-19	IDO2; indoleamine 2,3-dioxygenase 2 isoform X2
0.000157	GPAT4; glycerol-3-phosphate acyltransferase 4
5.26E-14	ANK1; ankyrin-1 isoform X13
3.57E-10	ARID5A; AT-rich interactive domain-containing protein 5A isoform X1
3.80E-08	MTHFD2; bifunctional methylenetetrahydrofolate dehydrogenase/cyclohydrolase, mitochondrial
1.56E-14	SLC4A5; electrogenic sodium bicarbonate cotransporter 4 isoform X2
9.31E-05	ADD2; beta-adducin
0.000149	PCYOX1; prenylcysteine oxidase 1
2.76E-06	FAM136A; protein FAM136A
6.30E-35	CASP14; caspase-14
7.99E-17	RETSAT; all-trans-retinol 13,14-reductase
2.77E-48	FABP3; fatty acid-binding protein, heart

1.46E-09 SERINC2; serine incorporator 2 isoform X1
1.49E-07 LOC121107469; collagen alpha-1(I) chain-like
5.89E-06 RHCE; blood group Rh(CE) polypeptide
1.13E-08 TRNAU1AP; tRNA selenocysteine 1-associated protein 1 isoform X4
1.31E-05 TRIM63; E3 ubiquitin-protein ligase TRIM63 isoform X2
4.21E-19 STMN1; stathmin isoform X1
3.53E-23 PAQR7; membrane progestin receptor alpha isoform X1
7.52E-06 INPP5B; type II inositol 1,4,5-trisphosphate 5-phosphatase isoform X5
4.25E-40 IFI27L2; interferon alpha-inducible protein 27, mitochondrial
2.71E-05 TMEM35B; transmembrane protein 35B
5.99E-11 FAM167B; protein FAM167B isoform X2
2.18E-05 AZIN2; antizyme inhibitor 2 isoform X3
3.47E-14 MYCL; protein L-Myc
1.00E-19 GALE; UDP-glucose 4-epimerase isoform X1
1.24E-18 HMGCL; hydroxymethylglutaryl-CoA lyase, mitochondrial isoform 4
6.99E-14 GRHL3; grainyhead-like protein 3 homolog isoform X4
2.94E-29 IL22RA1; interleukin-22 receptor subunit alpha-1 precursor
1.38E-05 NRGN; neurogranin isoform X1
0.000344 SPA17; sperm surface protein Sp17
8.84E-15 KCNJ5; G protein-activated inward rectifier potassium channel 4 isoform X2
1.08E-17 TMEM45L; transmembrane protein 45B isoform X2
1.21E-15 SCN3B; sodium channel subunit beta-3 isoform X1
7.07E-07 SC5D; lathosterol oxidase
4.94E-07 GRIK4; glutamate receptor ionotropic, kainate 4
4.05E-08 POU2AF1; POU domain class 2-associating factor 1 isoform X2
1.23E-07 ZBTB32; zinc finger and BTB domain-containing protein 16
6.45E-08 APOA5; apolipoprotein A-V
6.08E-05 FXYD6; FXYD domain-containing ion transport regulator 6 isoform X2
4.33E-07 TMPRSS13; transmembrane protease serine 13 isoform X5
3.85E-13 SCN4B; sodium channel subunit beta-4 isoform X3
7.66E-16 HYOU1; hypoxia up-regulated protein 1 isoform X1
1.76E-20 PLET1; uncharacterized protein PLET1
4.29E-06 BGLAP; osteocalcin preproprotein
3.92E-29 LOC121107551; sperm-associated antigen 4 protein-like isoform X2
1.67E-25 LOC101747704; sperm-associated antigen 4 protein-like isoform X3
1.42E-26 LOC107057474; sperm-associated antigen 4 protein-like isoform X3
5.89E-08 LOC429492; feather keratin 3
2.11E-19 LOC100859427; feather keratin 1-like

2.29E-17	LOC426914; feather keratin 1-like
2.30E-08	LOC107049496; solute carrier family 27 member 3
4.27E-05	SHE; SH2 domain-containing adapter protein E isoform X1
2.32E-11	CKS1B; cyclin-dependent kinases regulatory subunit 1
4.73E-28	FDPS; farnesyl pyrophosphate synthase
7.18E-08	SYT11; synaptotagmin-11 isoform X1
0.000145	GOLPH3L; Golgi phosphoprotein 3-like
1.41E-07	LOC121107589; uncharacterized protein LOC121107589 isoform X4
5.60E-08	PCP4L1; Purkinje cell protein 4 like 1
4.49E-09	APOA2; apolipoprotein A-II isoform X1
1.16E-06	TMOD4; tropomodulin-4
7.31E-10	LOC121107545; prefoldin subunit 2-like
5.14E-12	THEM4; acyl-coenzyme A thioesterase THEM4 isoform X2
1.86E-08	PFDN2; prefoldin subunit 2 isoform X1
5.19E-39	TULP1; tubby-related protein 1 isoform X1
1.14E-19	FKBP5; peptidyl-prolyl cis-trans isomerase FKBP5 isoform X1
0.004967	LHFPL5; LHFPL tetraspan subfamily member 5 protein isoform X1
1.69E-23	ELF3; ETS-related transcription factor Elf-3
1.35E-27	CSRP1; cysteine and glycine-rich protein 1
2.99E-12	CEPT1; choline/ethanolaminephosphotransferase 1 isoform X2
5.20E-09	PI16; peptidase inhibitor 16 isoform X6
7.16E-05	REN; renin isoform X4
6.97E-12	LOC121107619; Krueppel-like factor 15 isoform X1
4.48E-16	SLC45A3; solute carrier family 45 member 3
#######	SLC26A9; solute carrier family 26 member 9
8.76E-05	MAPKAPK2; MAP kinase-activated protein kinase 2
2.11E-06	C1orf116; specifically androgen-regulated gene protein
0.000191	PLXNA2; plexin-A2 isoform X3
8.54E-45	CAMK1G; calcium/calmodulin-dependent protein kinase type 1G
1.59E-46	LAMB3; laminin subunit beta-3
3.71E-46	G0S2; G0/G1 switch protein 2
2.17E-08	MOV10; putative helicase MOV-10 isoform X1
4.60E-38	SLC16A1; monocarboxylate transporter 1
1.86E-12	LOC419888; bile acid receptor-like
7.61E-20	SYCP1; synaptonemal complex protein 1 isoform X1
1.06E-16	TSHB; thyrotropin subunit beta isoform X1
6.09E-14	PACSIN1; protein kinase C and casein kinase substrate in neurons protein 1
2.95E-07	ITPR3; inositol 1,4,5-trisphosphate receptor type 3 isoform X1

4.08E-15 TSPO2; translocator protein 2
7.77E-05 APOBEC2; C-U-editing enzyme APOBEC-2
7.54E-19 CHIA-M31; acidic mammalian chitinase isoform X1
1.63E-16 CHIA; acidic mammalian chitinase precursor
1.09E-26 LOC768786; acidic mammalian chitinase isoform X1
9.84E-25 LOC107057545; uncharacterized protein LOC107057545 isoform X3
7.85E-13 LOC107049117; maestro heat-like repeat-containing protein family member 7 isoform X1
6.39E-10 VWA5A1; von Willebrand factor A domain-containing protein 5A isoform X8
1.98E-07 DAD1; dolichyl-diphosphooligosaccharide--protein glycosyltransferase subunit DAD1
3.04E-05 PLCD3; 1-phosphatidylinositol 4,5-bisphosphate phosphodiesterase delta-3 isoform X5
0.000201 LOC121107700; centromere protein V-like
2.38E-16 MRC2; C-type mannose receptor 2
1.91E-28 SLC4A1; band 3 anion transport protein isoform X3
2.45E-08 LOC107050147; pyruvate dehydrogenase kinase, isozyme 2 isoform X2
3.50E-11 SLC35B1; solute carrier family 35 member B1
2.82E-06 C27H17ORF105; uncharacterized protein C17orf105 homolog
1.84E-10 PHOSPHO1; phosphoethanolamine/phosphocholine phosphatase
4.73E-07 GNGT2; guanine nucleotide-binding protein G(I)/G(S)/G(O) subunit gamma-T2
0.000106 CACNB1; LOW QUALITY PROTEIN: voltage-dependent L-type calcium channel subunit beta-1 isoform X1
2.51E-07 PPP1R1B; protein phosphatase 1 regulatory subunit 1B isoform X1
1.10E-08 RARA; retinoic acid receptor alpha
9.65E-20 TOP2A; DNA topoisomerase 2-alpha
6.33E-09 KRT40; keratin, type I cytoskeletal 20
2.83E-08 KRT24; keratin, type I cytoskeletal 19
9.73E-14 KRT10; keratin, type I cytoskeletal 13
4.18E-12 P3H4; endoplasmic reticulum protein SC65
4.58E-09 FKBP10; peptidyl-prolyl cis-trans isomerase FKBP10
2.32E-17 G6PC; glucose-6-phosphatase
1.70E-13 VAT1; synaptic vesicle membrane protein VAT-1 homolog
2.26E-05 RND2; rho-related GTP-binding protein RhoN
1.85E-05 NBR1; next to BRCA1 gene 1 protein isoform X5
1.46E-16 SLC1A6; excitatory amino acid transporter 4 isoform X2
1.91E-07 LOC101750889; platelet glycoprotein Ib alpha chain-like
4.29E-46 ANGPTL4; angiopoietin-related protein 4
0.000116 C19orf71; uncharacterized protein C19orf71 homolog
4.72E-08 LOC100857637; transducin-like enhancer protein 1 isoform X3
0.000235 MPND; MPN domain-containing protein isoform X3
4.78E-81 ACSBG2; long-chain-fatty-acid--CoA ligase ACSBG2 isoform X4

0.000292 ONECUT3; one cut domain family member 3
7.37E-08 MEX3D; RNA-binding protein MEX3D
6.72E-07 LRG1; leucine-rich alpha-2-glycoprotein isoform X1
1.05E-18 CREB3L3; cyclic AMP-responsive element-binding protein 3-like protein 3 isoform X2
5.17E-10 ARID3A; AT-rich interactive domain-containing protein 3A
1.70E-14 MIDN; midnolin-A isoform X1
6.08E-09 LOC112530455; translation initiation factor IF-2-like isoform X1
0.000107 GAMT; guanidinoacetate N-methyltransferase isoform X1
1.41E-22 ADAMTSL5; ADAMTS-like protein 5 isoform X3
1.65E-08 LOC100859819; cocaine- and amphetamine-regulated transcript protein isoform X5
0.000387 OCLN; occludin
3.30E-05 USHBP1; Usher syndrome type-1C protein-binding protein 1 isoform X4
4.91E-05 COMP; cartilage oligomeric matrix protein
0.000268 CRTC1; CREB-regulated transcription coactivator 1 isoform X8
0.000361 LOC425795; ectoderm-neural cortex protein 1
6.51E-10 ARHGEF18; rho guanine nucleotide exchange factor 18 isoform X7
1.37E-27 LOC121107774; zinc finger protein 34-like isoform X1
1.30E-05 LPAR2; lysophosphatidic acid receptor 2
1.21E-08 FBXL12; F-box/LRR-repeat protein 12
2.94E-08 ACP5; tartrate-resistant acid phosphatase type 5 isoform X1
6.09E-14 CALR; calreticulin
3.07E-35 LOC121107827; coiled-coil domain-containing protein 81-like isoform X4
0.000135 LOC121107800; uncharacterized protein LOC121107800 isoform X1
2.36E-30 LOC107049386; uncharacterized protein LOC107049386 isoform X2
1.98E-08 CD163; CD163 molecule like 1 precursor
8.29E-27 LOC101751912; butyrophilin subfamily 3 member A2-like isoform X8
1.23E-53 LOC107049467; uncharacterized protein LOC107049467 isoform X2
0.000212 SLC27A2; bile acyl-CoA synthetase
5.39E-07 LOC107050037; leukocyte immunoglobulin-like receptor subfamily B member 3A
2.18E-05 LOC112531104; osteoclast-associated immunoglobulin-like receptor isoform X3
1.89E-06 LOC121107808; leukocyte immunoglobulin-like receptor subfamily B member 1
1.00E-16 CHIR-AB1; immunoglobulin-like receptor CHIR-AB1 precursor
4.42E-20 LOC107049866; osteoclast-associated immunoglobulin-like receptor
2.62E-15 LOC121107843; uncharacterized protein LOC121107843 isoform X1
3.56E-08 LOC121107862; platelet glycoprotein VI-like
0.000124 LOC107049967; uncharacterized protein LOC107049967 isoform X1
4.92E-05 LOC121107894; platelet glycoprotein VI-like isoform X1
0.000536 LOC121107814; LOW QUALITY PROTEIN: leukocyte immunoglobulin-like receptor subfamily A member 6

5.77E-28 LOC112531135; platelet glycoprotein VI-like
7.32E-08 LOC112531119; platelet glycoprotein VI-like isoform X5
7.69E-05 LOC107049516; leukocyte immunoglobulin-like receptor subfamily A member 2 isoform X1
3.50E-08 CHIR-AB-600; platelet glycoprotein VI isoform X3
3.66E-37 CHIR-B3; immunoglobulin-like receptor CHIR-B3 isoform X1
1.80E-08 LOC112531212; leukocyte immunoglobulin-like receptor subfamily A member 2 isoform X4
4.02E-07 LOC121107890; leukocyte immunoglobulin-like receptor subfamily B member 3
3.22E-31 LOC107056248; olfactory receptor 14J1-like
2.33E-13 FKBP11; peptidyl-prolyl cis-trans isomerase FKBP11
1.10E-08 METTL7A; methyltransferase-like protein 7A
3.20E-09 TMPRSS12; transmembrane protease serine 12 isoform X1
6.65E-07 COPZ1; coatomer subunit zeta-1 isoform X2
0.000195 TMEM106C; transmembrane protein 106C
2.54E-08 TUBA1A; tubulin alpha-1 chain
5.29E-08 SLC39A5; zinc transporter ZIP5
2.06E-11 CNPY2; protein canopy homolog 2
6.85E-09 STAT2; signal transducer and activator of transcription 2 isoform X2
9.88E-23 APOF; apolipoprotein F
7.10E-05 RDH16; retinol dehydrogenase 16
3.81E-24 TAC3; tachykinin-3 isoform X2
1.40E-27 INHBE; inhibin beta E chain
1.66E-06 GLI1; zinc finger protein GLI1
1.26E-06 POU6F1; LOW QUALITY PROTEIN: POU domain, class 6, transcription factor 1
2.60E-12 LOC121106503; maestro heat-like repeat-containing protein family member 2A isoform X2
5.21E-05 LOC107051274; Ig gamma chain C region isoform X1
1.11E-10 VKORC1; vitamin K epoxide reductase complex, subunit 1 precursor
1.01E-07 LOC121108153; cold shock protein CS66-like
4.43E-26 LOC121108155; RUS family member 1-like isoform X2
3.99E-16 LOC121108156; translation initiation factor IF-2-like isoform X1
1.74E-07 SYCN; syncollin
3.49E-27 OTX5; cone-rod homeobox protein isoform X1
1.77E-23 LOC107050328; uncharacterized protein LOC107050328
3.30E-16 LOC426220; avidin isoform X1
3.67E-08 LOC121108197; histidine triad nucleotide-binding protein 1-like
6.98E-61 LOC121108210; uncharacterized protein LOC121108210
0.000142 LOC121108481; serine/arginine repetitive matrix protein 1-like isoform X2
9.06E-05 LOC121108245; methyl-CpG-binding domain protein 2-like
2.29E-66 ALPK2; alpha-protein kinase 2

1.35E-12 LIPG; endothelial lipase
2.35E-05 EPG5; ectopic P granules protein 5 homolog
7.72E-06 MYORG; myogenesis-regulating glycosidase
2.76E-23 ENHO; adropin
6.99E-20 ARID3C; AT-rich interactive domain-containing protein 3C isoform X2
1.63E-07 AVDL; avidin isoform X1
1.16E-15 AVD; avidin precursor
4.61E-33 CAPSL; calcyphosin-like protein isoform X2
1.34E-05 NADK2; NAD kinase 2, mitochondrial isoform X2
0.000461 LIFR; leukemia inhibitory factor receptor precursor
1.08E-05 TMEM267; transmembrane protein 267 isoform X4
7.15E-38 FGF10; fibroblast growth factor 10 isoform X1
0.000434 EMB; embigin
0.00016 GZMA; granzyme A precursor
8.68E-11 DHX29; ATP-dependent RNA helicase DHX29
6.65E-09 SETD9; SET domain-containing protein 9 isoform X3
1.36E-12 PDE4D; cAMP-specific 3',5'-cyclic phosphodiesterase 4D isoform X9
3.57E-10 LOC121108525; vegetative cell wall protein gp1-like
1.18E-17 HMGCR; 3-hydroxy-3-methylglutaryl-Coenzyme A reductase
1.49E-13 ANKRD31; ankyrin repeat domain-containing protein 31 isoform X8
0.000128 NSA2; ribosome biogenesis protein NSA2 homolog
0.000211 RCL1; RNA 3'-terminal phosphate cyclase-like protein
5.01E-15 GLDC; glycine dehydrogenase (decarboxylating), mitochondrial precursor
1.00E-08 PLIN2; perilipin-2
3.11E-06 GDA; guanine deaminase isoform X3
0.000128 ZCCHC6; terminal uridylyltransferase 7 isoform X2
2.84E-23 FBP2; fructose-1,6-bisphosphatase isozyme 2
8.62E-56 GADD45G; growth arrest and DNA damage-inducible protein GADD45 gamma
5.48E-12 TPPP2; tubulin polymerization-promoting protein family member 2
7.52E-08 STARD4; stAR-related lipid transfer protein 4
3.49E-06 PPIP5K2; inositol hexakisphosphate and diphosphoinositol-pentakisphosphate kinase 2 isoform X1
1.53E-12 PCGF3; polycomb group RING finger protein 3
7.02E-08 MFSD7; solute carrier family 49 member A3
3.95E-73 LPL; lipoprotein lipase precursor
3.61E-30 PSD3; PH and SEC7 domain-containing protein 3 isoform X4
0.000149 NIPSNAP3A; protein NipSnap homolog 3A
4.28E-06 CAST; calpastatin
3.65E-07 RHOBTB3; rho-related BTB domain-containing protein 3 isoform X3

0.000198 POLR3G; DNA-directed RNA polymerase III subunit RPC7 isoform X2
3.33E-20 HAPLN1; hyaluronan and proteoglycan link protein 1 isoform X2
5.16E-06 MSH3; DNA mismatch repair protein Msh3 isoform X2
5.04E-06 LOC768418; zinc finger FYVE domain-containing protein 16-like
3.93E-15 ALDOB; fructose-bisphosphate aldolase B
5.86E-10 KIAA1958; uncharacterized protein KIAA1958 homolog isoform X1
1.42E-22 LPAR1; lysophosphatidic acid receptor 1 isoform X1
0.000583 C9orf72; guanine nucleotide exchange C9orf72 homolog isoform X1
3.90E-05 SPINK4; serine protease inhibitor Kazal-type 4
1.64E-05 DMXL1; dmX-like protein 1 isoform X1
4.91E-05 SEMA6A; semaphorin-6A isoform X3
2.62E-07 MARVELD2; MARVEL domain-containing protein 2
4.31E-36 CDKN2A; ARF tumor suppressor
2.04E-63 CDKN2B; cyclin-dependent kinase 4 inhibitor B
2.00E-20 TRIM36; E3 ubiquitin-protein ligase TRIM36 isoform X1
0.002006 LOX; protein-lysine 6-oxidase precursor
9.67E-16 LOC107050717; calumenin isoform X1
1.38E-09 LOC112533551; uncharacterized protein LOC112533551 isoform X2
1.53E-08 IER3; radiation-inducible immediate-early gene IEX-1 isoform X1
7.75E-07 LOC112533553; epithelial discoidin domain-containing receptor 1 isoform X1
2.70E-13 LOC107050652; putative killer cell immunoglobulin-like receptor-like protein KIR3DX1
1.57E-06 LOC425238; uncharacterized protein LOC425238 isoform X6
3.05E-09 _
3.28E-23 LOC121108719; uncharacterized protein LOC121108719 isoform X2
2.90E-14 CDIPT; CDP-diacylglycerol--inositol 3-phosphatidyltransferase isoform X1
0.000222 PRODH2; hydroxyproline dehydrogenase isoform X1
8.05E-07 LOC121108733; protein spinster homolog 1-like isoform X2
2.06E-07 LOC121108741; vasodilator-stimulated phosphoprotein-like isoform X1
2.25E-08 B3GAT3; LOW QUALITY PROTEIN: galactosylgalactosylxylosylprotein 3-beta-glucuronosyltransferase 3 isoform X1
4.65E-06 LOC121108844; histidine triad nucleotide-binding protein 1-like
0.000158 LOC121108855; uncharacterized protein LOC121108855 isoform X2
2.04E-13 LOC112530182; soluble scavenger receptor cysteine-rich domain-containing protein SSC5D-like
1.07E-10 LOC107051395; mucin-2-like isoform X1
1.34E-12 LOC112530174; mucin-22-like isoform X1
6.27E-09 LOC121108934; sphingomyelin phosphodiesterase 5-like
1.92E-34 MHCY13; major histocompatibility complex-Y, class I heavy chain, 13 precursor
5.60E-21 LOC112530071; NF-kappa-B inhibitor delta-like isoform X1
0.000109 LOC121108947; LOW QUALITY PROTEIN: medium-chain specific acyl-CoA dehydrogenase, mitochondrial isoform X1
