# Supplementary material for: Comparative analysis of liver transcriptome reveals adaptive responses to hypoxia environmental condition in Tibetan chicken
Source: Anim Biosci. 2023 Aug 23;37(1):28–38. doi: 10.5713/ab.23.0126 (PMC10766467; doi:10.5713/ab.23.0126)
Supplement: Supplementary file 3 [file ab-23-0126-Supplementary-Table-3.pdf]

Table S3

Geneid	HT1	HT2	HT3	HT4	HT5	BJ1	BJ2	BJ3	BJ4	BJ5	logFC	type	pvalue
LOC12111	3.334	4.314	4.976	0.771	0.913	1.454	0.884	0.787	0.627	1.96	-1.324	down	1.38E-08
LOC1211C	2.719	2.124	1.201	0.265	0.41	0.321	0.422	0.51	0.262	0.23	-1.94196	down	2.20E-16
LOC7764€	21.883	20.546	22.844	2.642	1.25	4.489	0.351	4.43	4.018	11.053	-1.50646	down	1.22E-11
LOC1211C	30.716	35.254	57.809	35.949	49.649	13.648	10.928	24.605	32.682	13.677	-1.13189	down	3.94E-07
CD69L	18.257	17.274	8	0.32	0.614	5.864	5.308	2.866	0.57	5.454	-1.14801	down	3.71E-08
ELAPOR2	2.989	3.226	1.334	0.728	1.061	1.529	18.424	13.468	0.775	4.048	2.033464	up	6.95E-23
ALG12	12.218	11.172	12.612	15.737	13.32	50.082	44.247	26.88	24.259	28.194	1.416392	up	5.15E-12
CRELD2	16.486	14.861	20.903	18.59	14.191	97.527	90.434	31.47	46.911	41.482	1.855984	up	7.28E-19
ARL8BL	0.035	0.228	1.109	0	0.25	0.158	0.321	0.067	0.059	0.173	-1.05513	down	0.007844
IQUB	0.993	0.449	2.583	0.444	0.465	0.447	0.194	0.169	0.371	0.725	-1.36989	down	7.21E-08
CPED1	3.776	3.847	5.473	5.571	5.853	1.651	2.275	1.213	2.67	1.315	-1.42572	down	4.51E-11
CAV1	0.264	1.08	1.577	1.139	1.492	2.616	3.106	1.53	3.242	0.917	1.038679	up	1.14E-05
AMIGO2	1.022	1.06	1.593	1.993	2.793	0.911	0.946	0.493	0.994	0.647	-1.08312	down	1.53E-06
IFNG	0.452	0.295	0.408	0.099	0.062	0.92	0.832	0.465	0.663	1.165	1.616291	up	3.80E-10
TSPAN8	0.838	0.763	1.015	0.629	1.309	2.31	4.434	2.497	3.639	1.734	1.681055	up	4.44E-14
LGR5	0.702	0.822	0.36	0.905	0.909	3.341	3.261	2.301	9.376	1.661	2.429261	up	1.82E-28
GLIPR1	0	0.025	0.03	0.083	0.221	1.004	0.376	0.147	0.193	0.264	2.450033	up	7.39E-19
LOC1211C	1.294	1.397	2.113	0.585	1.728	0.66	0.53	0.312	0.877	0.642	-1.23487	down	1.80E-05
MRPL42	6.038	5.043	3.946	4.993	4.485	2.816	2.224	1.65	2.283	2.467	-1.09865	down	2.29E-07
LOC4278€	2.828	2.523	1.719	3.084	4.224	1.863	0.789	0.645	1.654	1.377	-1.1834	down	5.11E-07
HIST1H4D	18.688	19.768	25.828	12.18	20.519	11.189	4.688	14.724	10.306	5.61	-1.05989	down	3.65E-06
HIST1H2B	2.92	2.73	1.329	2.143	6.321	0.591	0.64	2.215	1.766	0.931	-1.32923	down	1.58E-06
HIST1H46	0.241	0.263	0	0.198	3.553	0.547	0.165	1.165	0	0.16	-1.06087	down	0.00689
HIST1H2A	1.223	1.111	1.677	2.4	0.698	0.308	0.695	0.328	0.115	0.27	-2.04741	down	3.21E-11
EMP1	0.074	0.109	0.309	0.304	0.38	1.151	0.718	0.321	1.001	0.275	1.555342	up	2.84E-10
GUCY2C	0.047	0.025	0.142	0.054	0.319	0.277	0.029	0.064	2.693	0.028	2.386736	up	2.41E-21
MEI1	1.876	1.816	1.172	1.415	2.148	0.229	0.347	0.489	0.454	0.455	-2.0911	down	2.48E-20
CHADL	1.099	1.125	1.179	1.815	0.233	0.825	0.232	0.392	0.607	0.343	-1.18241	down	8.32E-08
MGAT3	1.404	1.447	1.943	1.9	1.813	4.218	3.441	1.801	4.798	2.826	1.005498	up	6.88E-07
NPTXR	2.066	1.895	0.571	0.682	0.203	5.885	8.598	13.678	0.495	7.821	2.750288	up	1.10E-36
FAM20CL	433.81	459.966	1.954	25.555	0.424	523.552	814.782	1189.31	2.464	596.951	1.762418	up	5.08E-17
SUN2	18.332	17.316	9.806	6.471	7.075	35.698	29.788	47.574	8.305	27.757	1.337632	up	3.12E-11
CBY1	0.478	0.826	1.929	0.42	0.631	6.682	2.896	3.326	3.35	3.045	2.170187	up	1.99E-22
KDELR3	0.398	0.521	0.373	0.423	0.529	2.307	1.677	1.402	0.753	1.138	1.695051	up	2.93E-13
CARD10	1.29	1.45	2.944	2.441	0.998	0.658	0.409	0.737	1.43	0.919	-1.13441	down	4.45E-07
CSF2RB	0.238	0.393	0.341	0.579	0.196	0.884	0.906	0.276	1.113	0.661	1.133981	up	6.59E-07

CRCBL	2.141	1.914	3.887	2.123	2.544	5.738	5.454	3.142	6.986	4.644	1.041765	up	1.28E-07
MCM5	0.54	0.535	0.614	0.596	0.998	4.472	2.415	1.251	0.81	0.872	1.579247	up	6.41E-13
C12orf75	0.21	0.128	0.558	0.214	0.482	0.581	0.577	0.293	2.19	0.215	1.27361	up	2.62E-09
HSP90B1	157.01	148.585	216.797	181.815	205.894	1031.764	844.788	253.937	664.194	449.421	1.833716	up	1.42E-18
NT5DC3	1.175	1.159	1.403	1.178	1.545	4.061	2.549	2.225	2.215	2.11	1.025985	up	4.55E-07
ASCL1	5.587	5.175	8.032	2.459	3.31	1.635	1.028	1.576	1.949	2.449	-1.50734	down	3.79E-11
IGF1	1.396	2.968	5.042	4.753	9.936	4.404	10.814	5.873	22.206	9.644	1.135489	up	1.93E-08
CGTL	10.907	8.938	16.05	5.069	31.518	22.47	31.378	64.413	193.834	26.296	2.222918	up	4.55E-27
EPS8	1.2	1.249	2.784	1.733	1.661	4.167	5.372	3.376	6.386	2.295	1.323331	up	3.48E-11
SLCO1B1	1.066	0.722	0.939	0.309	0.386	2.499	2.245	1.513	1.486	0.896	1.334753	up	3.19E-10
LDHB	371.616	323.912	870.705	415.305	520.845	249.346	228.279	158.51	283.675	310.132	-1.02471	down	5.04E-06
SSPN	0.694	0.719	3.161	1.225	0.776	2.286	1.461	3.553	3.895	2.002	1.004597	up	7.03E-08
BHLHE41	1.708	1.624	7.576	3.005	1.425	5.591	3.345	9.224	7.864	5.082	1.019844	up	3.63E-08
ENDOUL	1.525	1.284	0.447	0.403	1.314	1.945	4.394	0.998	0.683	4.242	1.301144	up	9.99E-10
PNPLA3	156.704	150.92	24.41	32.198	22.297	131.398	219.914	449.518	39.295	104.253	1.288777	up	1.45E-10
LOC10175	1.536	1.246	1.217	1.653	1.183	2.687	4.124	4.745	1.209	3.465	1.247039	up	5.06E-10
A2ML3	9.361	12.466	9.122	12.015	43.861	5.962	3.372	6.905	8.565	6.027	-1.49358	down	8.89E-12
CASR	1.936	1.763	1.204	1.279	0.076	0.084	0.449	0.669	0.025	0.148	-2.18219	down	1.67E-20
CNP1	3.375	3.785	4.095	2.708	1.362	1.456	0.452	1.358	1.565	1.036	-1.38443	down	4.38E-09
GPR162	0.25	0.278	0.697	0.226	0.295	1.614	2.098	2.076	0.551	3.262	2.455757	up	4.86E-29
CDCA3	0.108	0.236	0.287	0.119	0.371	2.452	1.034	0.244	0.183	0.215	1.875983	up	2.01E-11
RBP5	40.65	39.033	69.173	47.182	67.993	21.529	17.971	23.836	27.752	31.234	-1.10999	down	7.05E-07
EPHA1	0.234	0.347	0.187	0.234	0.209	0.912	1.032	0.915	0.552	0.91	1.83089	up	7.65E-16
GSTK1	25.813	24.89	36.138	24.733	60.593	16.681	14.309	15.3	22.613	11.989	-1.08969	down	7.53E-07
LOC10085	0.216	0.094	1.373	0	0.074	0.163	0	0	0	0.071	-2.88213	down	3.43E-07
HAO2	41.002	37.026	43.088	67.589	79.114	11.438	21.814	27.654	30.319	26.975	-1.17999	down	7.38E-08
SIDT1	0.279	0.324	0.715	0.305	0.456	0.771	0.591	0.588	0.559	2.181	1.17177	up	1.12E-08
APOV1	9688.972	12281.13	57.327	2791.273	5.816	7899.035	21270.79	22273.24	38.92	13753.57	1.393892	up	2.25E-11
SLC19A2	8.997	8.025	8.924	11.297	8.364	2.352	0.768	1.35	0.54	0.948	-2.9353	down	2.38E-39
DPT	0.487	0.599	1.194	0.384	0.64	2.096	2.272	1.335	3.035	1.333	1.606456	up	1.32E-13
TBX19	5.311	4.359	5.546	4.173	2.871	2.136	1.668	2.204	1.026	3.627	-1.06176	down	3.11E-06
CD200L	0	0	0.023	0.313	0.211	1.857	0.15	2.036	0	0.029	2.884768	up	1.04E-22
LOC12111	0	0.009	0.011	0	0.044	0.209	0.015	0.467	1.54	0	5.01818	up	2.67E-40
CREG1	48.616	45.349	90.265	84.383	124.389	47.715	43.932	20.845	38.565	43.222	-1.01639	down	3.11E-06
MAEL	1.904	1.824	2.163	2.202	3.364	0.567	0.68	0.925	1.899	1.039	-1.16405	down	1.08E-07
ROBO1	0.809	0.627	0.597	0.355	1.754	1.518	0.686	1.313	4.573	0.492	1.050086	up	2.65E-07
MAP3K7C	6.165	6.29	15.608	12.845	8.111	28.686	27.337	31.476	62.465	15.117	1.751658	up	9.71E-18
GART-B	0.305	0.262	1.808	0.246	0.803	0.187	0.111	0.129	0.312	0.17	-1.90752	down	1.38E-14

KCNJ15	0.115	0.131	0.336	0.186	0.268	0.477	0.293	0.179	0.867	1.03	1.453498	up	8.31E-12
PCP4	19.784	17.63	42.577	28.453	30.131	4.538	8.201	2.305	3.321	14.463	-2.0775	down	1.01E-19
ABCG1	0.276	0.304	0.853	0.272	0.215	1.465	0.527	0.4	1.609	0.411	1.198208	up	5.88E-09
AGPAT3	34.164	32.233	24.234	30.034	25.402	12.93	13.398	17.827	8.359	14.746	-1.11875	down	2.18E-07
LOC10705	0.573	0.409	0.995	0.235	0.749	1.386	3.836	0.126	0.838	0.103	1.085459	up	8.53E-07
MAOB	8.732	8.255	8.987	4.828	8.704	8.903	10.683	24.451	40.925	8.794	1.246734	up	2.88E-10
NR0B1	37.5	36.706	148.366	39.949	35.715	32.383	28.581	19.765	34.57	21.274	-1.12675	down	9.75E-07
SLC51AL	1.037	0.838	1.068	1.955	3.483	1.077	0.512	0.141	1.838	0.266	-1.12725	down	1.81E-07
MAP3K15	1.168	1.278	2.428	1.267	1.369	4.962	6.997	6.304	5.724	2.466	1.815859	up	4.53E-19
PDHA2	12.365	12.837	21.312	13.524	16.276	46.124	73.838	56.195	48.92	21.254	1.690513	up	5.26E-17
ADGRG2	0.147	0.183	0.172	0.12	0.105	1.645	2.659	1.249	0.658	0.461	3.189284	up	1.72E-42
GRPR	0.273	0.29	0.054	0.028	0.035	0.735	3.597	1.252	0	0.44	3.137743	up	1.15E-27
LOC41865	31.573	29.276	14.798	28.114	9.619	1.418	1.554	9.993	8.866	6.765	-1.98709	down	5.33E-20
CRLF2	0.753	0.548	2.546	0.64	0.487	0.556	5.798	0.457	0.559	5.604	1.382251	up	6.73E-13
IL1R2	1.412	1.015	4.233	3.323	1.28	4.061	7.949	1.498	3.984	15.804	1.563334	up	8.48E-15
IL1R1	19.591	19.643	52.678	44.227	11.897	46.635	92.94	38.971	55.463	127.264	1.287113	up	6.10E-11
IL1RL1	1.863	2.123	4.115	1.225	2.715	3.483	21.87	4.849	1.648	13.623	1.916615	up	1.95E-21
RAB20	33.363	32.669	54.294	22.269	35.116	16.306	19.708	15.097	14.101	22.079	-1.02559	down	5.53E-06
LOC12110	1.523	2.514	0.799	7.567	0.711	11.837	17.777	17.946	13.482	18.231	2.595263	up	4.36E-31
DOCK9	5.304	5.281	7.746	4.802	5.196	3.154	2.751	1.776	2.225	2.289	-1.21565	down	2.17E-08
HS6ST3	1.605	1.497	1.513	1.551	0.979	0.667	0.507	0.387	0.573	0.721	-1.32193	down	1.40E-09
DCT	2.22	2.581	0.314	0.343	0.081	0.026	0.288	0.316	0	1.464	-1.40123	down	6.86E-10
ACOD1	1.389	1.414	1.721	1.603	0.649	3.21	4.909	1.788	0.95	5.79	1.296126	up	3.37E-10
LOC11253	1.608	1.246	0.952	2.225	0.38	0.18	1.266	0.174	0.448	0.43	-1.35802	down	9.15E-10
CKAP2	0.174	0.142	0.531	0.19	0.312	1.558	0.652	0.462	0.221	0.244	1.214455	up	8.61E-08
AMER2	0.285	0.256	0.885	0.153	0.26	0.125	0.769	0.669	1.749	0.467	1.037073	up	6.30E-08
SGCG	15.113	13.654	3.805	0.356	1.199	0.151	0.284	1.041	0.169	0.121	-4.26849	down	2.51E-67
GJB2	0.427	0.523	0.025	0.386	1.49	3.346	3.078	7.292	0.126	2.719	2.536157	up	9.87E-32
ACAT1	280.209	264.862	449.211	387.065	396.999	186.729	148.465	124.989	231.673	161.215	-1.05979	down	9.60E-07
MMP1	0	0	0.037	0	0	10.455	4.913	0.022	7.896	0.506	9.146175	up	#######
MMP7	0.724	0.148	0.721	0.521	0.093	1.076	4.677	0.568	1.839	0.628	1.991004	up	2.62E-15
TAF1D	25.24	23.33	26.332	31.761	22.81	12.061	8.9	10.156	11.147	12.533	-1.24041	down	6.67E-09
AQP11	9.811	9.128	6.632	12.259	13.171	3.597	1.998	3.93	7.132	4.457	-1.27213	down	3.15E-09
CAPN5	2.419	2.309	4.736	2.929	5.462	1.005	0.981	0.332	1.576	1.115	-1.8327	down	1.02E-16
TSKU	1.372	1.845	1.784	5.612	10.349	3.625	0.78	4.43	66.622	0.695	1.860856	up	2.55E-18
WNT11	0.476	0.429	2.908	1.358	1.013	5.505	0.355	1.985	6.478	0.411	1.25186	up	4.14E-10
UVRAG	13.895	12.961	11.419	16.883	12.342	5.042	3.794	6.507	6.879	7.29	-1.19345	down	2.51E-08
ART1L1	0.309	0.424	0.443	0.381	0.507	1.783	0.6	0.388	0.993	0.674	1.1026	up	1.44E-05

LRTOMT	1.506	1.612	1.663	0.788	1.582	4.612	2.381	4.23	6.144	2.58	1.479308 up	8.20E-12
ADAM15	2.879	2.467	2.28	10.016	2.523	0.39	0.208	0.151	0.026	0.171	-4.40662 down	1.89E-66
SLCO2B1	22.211	22.886	18.128	38.38	42.452	14.75	5.634	10.098	19.466	15.019	-1.1488 down	4.88E-08
P2RY6	0.615	0.827	0.98	1.685	0.807	2.476	1.783	2.012	2.604	2.203	1.171911 up	1.95E-07
DGAT2	0.817	0.582	0.914	0.955	0.795	2.387	1.599	1.523	2.161	1.109	1.110558 up	1.78E-07
HBE	0	0.145	18.385	0	0.152	0	0	0	0.125	0.147	-6.07601 down	1.04E-48
HBBA	251.682	258.768	745.477	87.668	273.636	72.813	40.39	93.927	76.331	92.35	-2.10543 down	2.09E-20
HBE1	46.694	46.334	122.225	11.314	34.039	9.482	5.154	13.908	10.575	14.429	-2.28286 down	3.47E-23
TAF10	33.266	32.073	29.405	36.015	40.872	14.419	11.778	12.652	10.242	16.576	-1.386 down	1.72E-10
TPP1	197.54	210.865	179.416	267.845	237.865	54.807	60.679	56.797	63.377	76.953	-1.80653 down	4.08E-17
GIMAP1-(	7.613	7.95	5.13	5.913	2.9	3.316	1.733	1.797	1.806	1.95	-1.47624 down	5.37E-12
ACKR2	1.96	1.81	4.688	2.679	3.108	8.257	5.109	26.738	3.641	5.031	1.775357 up	9.94E-20
CYP8B1	3.027	2.459	7.577	4.087	5.467	14.955	9.325	50.683	5.915	8.959	1.989664 up	8.04E-24
ALS2L	3.734	3.541	5.261	5.932	3.806	2.045	2.939	1.874	2.011	1.763	-1.06659 down	1.04E-06
CHDSD	1.816	1.503	2.053	1.127	7.993	1.128	0.072	0.815	3.076	0.802	-1.29746 down	5.98E-09
CATH2	0.158	0.155	1.513	4.098	0	0.359	0.81	0.076	1.475	0.079	-1.0803 down	6.59E-05
CATH1	0.102	0.201	1.342	3.694	0.21	0	0.366	0.247	0.259	0.152	-2.43228 down	7.68E-15
GIMAP6	26.516	28.234	6.858	3.539	4.856	9.302	5.387	3.706	5.945	4.875	-1.26056 down	6.59E-10
VILL	0.411	0.406	0.348	0.145	0.621	0.447	1.374	1.021	0.853	0.683	1.17884 up	1.11E-08
DLEC1	3.841	3.28	5.44	4.057	2.006	1.007	1.622	1.96	0.895	3.068	-1.12237 down	7.27E-07
ACAA1	153.55	145.844	192.261	192.372	200.895	41.272	43.597	46.987	43.014	89.999	-1.74025 down	1.40E-15
APBB1IP	1	1.071	3.128	1.33	4.054	5.828	3.312	4.074	8.747	5.069	1.352398 up	1.36E-11
NELL3	0.006	0	0.06	0.01	0.184	0.531	1.391	0.43	0.323	0.293	3.487855 up	2.09E-45
THNSL1	13.847	12.135	4.25	15.547	8.683	4.46	4.146	3.733	4.222	7.161	-1.19885 down	6.85E-09
RSU1	2.554	2.116	3.427	2.316	2.365	6.013	7.569	6.147	6.052	3.183	1.180283 up	3.30E-09
ACBD7	1.497	1.217	3.527	1.728	6.917	0.756	0.166	0.566	1.404	0.221	-2.25575 down	2.54E-21
OLAH	0.248	0.282	1.006	0.256	1.495	0.163	0.049	0.108	0.284	0.048	-2.325 down	3.44E-20
CROT	68.879	62.909	58.579	57.833	59.509	21.51	20.677	35.884	27.192	27.683	-1.2107 down	2.69E-08
ABCB1LA	27.561	25.71	20.052	17.421	19.97	5.618	2.327	4.299	2.47	4.426	-2.53186 down	9.32E-31
CYP51A1	48.663	52.847	8.028	7.111	62.297	50.246	188.232	176.312	18.671	35.93	1.391241 up	1.94E-12
CDK6	8.803	8.955	11.844	8.675	7.442	38.874	32.997	28.237	8.772	9.571	1.373328 up	8.31E-12
PDK4	72.111	69.423	92.979	153.728	64.769	33.202	13.438	12.775	68.738	59.593	-1.27074 down	1.03E-09
ICA1	0.639	0.604	0.692	0.156	0.307	0.585	1.39	0.971	0.368	1.819	1.09637 up	1.24E-07
THSD7A	0.688	0.703	0.86	0.597	0.484	0.823	3.484	1.4	1.902	6.213	2.050865 up	4.55E-23
LOC39515	2.725	2.891	0.059	0	0	9.216	12.433	0.036	6.174	10.63	2.760671 up	1.45E-31
VWDE	3.3	3.238	1.375	2.446	0.372	0.266	7.602	7.824	1.805	12.28	1.471984 up	5.06E-13
HDAC9	0.261	0.154	0.35	0.245	0.251	1.456	0.646	0.455	0.88	0.427	1.611683 up	3.04E-14
WIPF3	1.121	1.131	1.22	0.407	0.626	2.989	0.977	1.576	4.18	1.012	1.25166 up	1.17E-09

NR1D2	17.133	15.292	32.307	19.308	26.853	15.341	8.268	6.459	12.927	9.143	-1.08869	down	5.53E-07
EOMES	1.415	1.14	1.455	0.616	1.143	4.246	2.487	1.764	3.38	1.548	1.217816	up	5.26E-09
LOC10175	0.982	0.675	1.017	0.363	0.454	0.334	0.101	0.237	0.499	0.341	-1.20448	down	0.000105
ACAD11	94.158	91.985	133.79	127.816	125.149	46.325	32.917	31.58	65.987	55.431	-1.30264	down	1.28E-09
NPHP3	6.772	6.017	8.602	6.839	5.552	3.304	2.843	2.476	3.523	3.038	-1.15344	down	1.07E-07
LZTFL1	0.199	0.303	0.242	0.25	1.074	0.09	0.311	0.153	0.291	0.184	-1.00348	down	0.000231
KIF15	0.065	0.038	0.103	0.331	0.214	1.368	0.537	0.305	0.414	0.228	1.918043	up	4.94E-16
BMPER	0.655	0.781	2.369	2.089	2.146	5.288	6.176	2.324	3.44	3.094	1.337233	up	3.50E-11
PPP1R17	0.696	0.307	1.52	0.705	1.363	3.133	3.493	2.062	2.466	1.749	1.489815	up	5.20E-13
AMPH	0.024	0.056	1.094	0.01	2.771	1.514	0.087	0.106	9.219	0.012	1.466436	up	2.47E-12
INHBA	4.865	4.491	2.44	9.618	6.196	26.063	11.794	20.012	12.722	12.118	1.582679	up	4.53E-14
IGFBP1	40.494	39.916	40.039	34.635	17.005	6.383	4.343	5.715	20.567	34.853	-1.25982	down	4.08E-09
LOC1211C	0.224	0.212	3.531	0.102	2.508	0.169	0.229	0.192	0.169	0.939	-1.95045	down	2.93E-12
SALL3	0.028	0.029	0.303	0.176	0.231	0.321	0.202	1.012	0.838	0.556	1.926196	up	1.33E-20
KIAA0319	0.168	0.151	0.705	0.482	0.205	0.275	0.798	0.933	0.454	1.12	1.062924	up	4.76E-08
DCDC2	1.066	0.95	2.24	1.084	1.124	3.988	6.431	2.913	3.303	1.315	1.472771	up	2.36E-13
ELOVL2	182.236	179.616	5.847	23.01	45.086	210.463	322.092	354.56	12.502	194.073	1.327472	up	1.34E-10
SYCP2L	87.486	81.32	3.059	10.072	17.064	94.731	123.019	200.78	4.862	92.297	1.373703	up	2.25E-11
GCNT2	1.552	1.459	0.351	0.81	1.005	1.298	3.146	5.082	0.26	1.366	1.106368	up	2.49E-08
LOC10705	0	0	0	0	0	0.67	0.648	0.01	1.02	0	8.878358	up	7.97E-60
PPP1R3G	6.647	6.35	8.518	6.672	18.704	2.418	3.858	2.582	6.744	6.011	-1.11723	down	4.85E-07
ECI2	248.06	223.024	457.551	325.633	209.669	63.63	55.001	26.728	42.897	131.908	-2.19295	down	1.50E-23
SERPINB1	0.182	0.238	0.512	0.287	0.359	0.934	0.687	0.284	1.066	0.305	1.051474	up	4.88E-06
SERPINB1(	0.068	0.607	0.524	2.123	0.433	0.822	2.97	0.261	3.573	0.67	1.142552	up	8.55E-07
CTNND2	3.216	2.687	0.587	0.945	0.31	0.248	0.507	0.968	0.256	0.502	-1.64037	down	4.90E-14
CMBL	109.934	119.002	147.194	84.218	175.479	21.862	11.568	12.189	82.96	29.728	-2.00588	down	1.58E-20
SRD5A1	3.159	2.519	9.173	6.425	4.217	1.999	1.317	1.52	2.017	1.213	-1.65956	down	9.79E-14
UBE2QL1	0.955	1.081	1.225	0.892	1.098	0.312	0.232	0.938	0.384	0.225	-1.32633	down	1.41E-07
LOC76974	0.102	0.12	0.049	0.117	0.563	0.253	1.122	0.627	1.513	0.926	2.217425	up	1.46E-18
LOC1211C	0.586	0.332	0.218	0.257	0.241	1.635	0.559	0.716	1.058	0.465	1.437094	up	1.53E-07
MOCOS	33.131	32.776	10.736	22.622	11.783	52.359	59.292	65.638	17.412	59.737	1.196094	up	5.33E-09
PSMG2	13.81	11.588	11.217	9.959	23.015	6.06	7.382	5.233	6.431	8.869	-1.03428	down	3.26E-06
TGIF1	12.25	10.563	11.097	3.061	3.781	3.637	3.843	2.339	4.807	3.757	-1.14828	down	1.06E-07
COLEC12	0.88	0.745	0.979	1.147	1.169	5.12	5.122	1.963	6.115	2.303	2.066408	up	3.08E-21
ABHD3	12.343	10.311	30.543	4.203	14.329	2.037	2.289	2.008	1.751	2.319	-2.78483	down	7.59E-34
MCM4	0.945	0.866	1.037	0.742	1.931	5.9	3.503	1.734	1.405	0.917	1.2848	up	7.42E-10
C8orf22	2183.872	2049.309	595.971	1564.483	746.212	157.922	187.591	268.858	270.689	646.791	-2.22061	down	3.80E-25
CYP7A1	5.926	9.393	3.053	30.473	24.168	74.577	50.26	69.243	51.835	58.236	2.058487	up	3.12E-22

GDAP1	0.009	0.029	0.084	0.78	0.108	0.667	0.935	0.008	0.045	1.377	1.581167	up	2.64E-11
FABP4	0.671	0.603	1.803	3.858	0.861	0	0	0.567	0.568	0.333	-2.4049	down	2.05E-13
ATP6V0D2	0.331	0.185	0.978	0.233	0	1.391	2.127	1.118	0.12	0.422	1.581348	up	2.98E-12
WWP1	17.708	16.534	26.756	14.9	10.589	49.146	49.748	18.574	36.312	22.794	1.029675	up	2.41E-07
PTDSS1	16.268	15.45	18.831	8.89	8.963	26.989	43.502	41.046	12.685	39.314	1.257437	up	1.17E-10
ANXA13	0.635	0.453	0.643	0.783	0.652	2.189	1.919	2.424	1.759	0.773	1.516006	up	2.29E-11
SQLE	22.329	24.556	0.467	0.583	24.186	16.938	113.347	71.684	4.413	12.068	1.598745	up	2.72E-15
LOC12111	3.227	3.889	5.916	2.652	5.582	2.067	2.259	1.312	0.575	1.769	-1.41316	down	3.56E-09
CFAP36	2.004	1.618	0.259	0.801	0.482	0.818	0.296	0.314	0.367	0.519	-1.15638	down	2.89E-07
FANCL	3.15	2.907	15.648	18.086	12.899	5.882	4.503	4.028	4.945	5.421	-1.08826	down	1.10E-06
BUB1	4.253	4.887	8.572	9.029	2.457	21.36	24.709	21.137	17.285	7.567	1.656505	up	5.85E-16
LOC12111	2.872	2.802	2.659	7.763	7.404	1.719	0.438	1.132	1.591	4.074	-1.39156	down	7.98E-11
CAPN13	0.264	0.107	0.381	1.645	0.336	0.143	0.103	0.109	0.32	0.062	-1.88363	down	4.33E-14
PPP1R21	12.373	10.366	18.607	13.276	13.017	6.981	6.021	5.132	6.721	6.814	-1.09466	down	6.09E-07
UGP2	111.949	111.93	146.823	110.221	109.185	253.878	318.758	280.826	303.237	190.158	1.190539	up	1.74E-09
ITPKC	3.123	3.323	7.669	2.814	3.549	12.403	13.807	6.731	10.367	7.498	1.310714	up	2.90E-11
GINS1	0.504	0.431	0.617	0.7	1.273	3.333	1.03	0.972	0.918	0.807	1.001021	up	2.15E-05
TLR5	1.701	1.462	1.014	2.244	0.513	0.157	0.545	0.491	0.626	0.828	-1.38765	down	8.67E-11
LOC42141	15.524	14.791	15.424	24.637	22.95	45.167	31.445	44.462	42.075	27.833	1.033046	up	2.40E-07
MAD2L1B	3.289	2.865	4.357	2.523	8.968	2.109	1.578	1.349	2.144	1.27	-1.38009	down	7.01E-10
PLD5	0.29	0.361	0.031	0.571	1.529	0.148	0.257	0.138	0.074	0.113	-1.9229	down	1.75E-17
LOC10085	1.531	1.704	0.49	1.099	0.669	2.511	2.449	2.158	2.98	2.252	1.168116	up	1.12E-07
DACT2	0.361	0.382	0.406	0.794	2.125	0.024	0.098	0.215	0.216	0.179	-2.46636	down	1.35E-21
MPC1L	355.379	296.186	600.226	259.974	295.607	191.184	127.226	68.629	93.566	192.505	-1.42497	down	5.76E-11
TFB1M	11.896	9.725	5.261	6.318	6.16	2.761	3.44	3.74	3.13	4.973	-1.12499	down	2.02E-07
SGK1	10.604	9.899	24.114	13.805	25.396	41.136	67.959	47.834	19.99	27.411	1.285518	up	2.49E-11
SLC2A12	0.173	0.198	0.189	0.284	0.344	0.428	1.336	2.647	0.292	0.375	2.091086	up	2.05E-21
LOC10705	32.969	32.892	24.991	42.267	11.618	62.125	220.01	52.715	47.614	133.081	1.832629	up	1.83E-18
TMEM200	0.75	0.771	2.214	0.551	2.284	3.634	4.091	4.56	2.416	1.01	1.257171	up	4.57E-11
GJA1	19.079	19.274	11.943	15.972	6.838	34.225	31.704	31.052	25.008	36.936	1.120231	up	4.86E-08
MCM9	1.662	1.497	0.398	0.382	0.229	0.304	0.329	0.224	0.227	0.222	-1.67042	down	2.17E-13
LOC10705	0.945	1.029	0.556	0.385	0.835	0.35	0.362	0.29	0.434	0.324	-1.08914	down	2.15E-06
LOC12110	4.275	4.195	3.113	3.885	2.768	1.267	1.202	2.803	1.003	1.964	-1.14577	down	2.02E-07
ME1	8.539	9.374	9.18	7.501	16.873	30.521	54.254	41.482	38.642	8.06	1.748612	up	8.65E-18
RWDD2A	3.585	3.795	3.749	2.782	5.311	9.592	11.618	10.689	8.742	3.541	1.200488	up	1.52E-09
TTK	0.133	0.043	0.098	0.164	0.234	1	0.33	0.202	0.306	0.17	1.572119	up	1.74E-10
GFRAL	1.187	1.504	0.189	0.175	0.026	0.071	0.422	0.097	0	0.087	-2.17789	down	6.85E-18
GSTA3	16.969	18.236	59.154	35.31	163.226	116.175	95.944	70.919	740.225	32.579	1.849921	up	1.65E-19

LOC10085	9.884	9.81	15.876	10.265	56.394	14.45	7.48	14.967	6.133	4.029	-1.11918	down	1.16E-06
FAM110C	2.976	2.726	2.712	4.42	5.076	0.948	1.006	1.991	1.266	1.435	-1.42952	down	1.13E-10
PXDN	0.391	0.6	0.96	0.915	0.742	1.783	1.688	1.137	2.05	1.276	1.13576	up	2.45E-08
KLF11	6.606	6.064	5.977	3.564	2.631	1.134	2.776	2.252	2.018	1.924	-1.29743	down	4.93E-09
RRM2	0.136	0.204	0.619	0.895	0.879	6.142	2.247	1.126	0.823	0.501	1.985703	up	3.36E-18
PDIA6	57.434	55.471	70.146	63.546	69.986	224.049	204.904	78.995	141.77	107.376	1.257875	up	6.67E-10
VSNL1	18.663	15.883	23.938	16.742	23.218	5.342	5.93	5.854	4.292	12.364	-1.54291	down	2.81E-12
RHOB	166.363	172.302	92.283	194.081	174.943	30.295	10.927	12.454	86.132	22.745	-2.299	down	2.38E-27
HADHA	118.676	122.57	136.401	163.422	167.546	60.719	59.753	35.407	44.403	68.381	-1.39919	down	5.78E-11
HADHB	362.649	336.843	399.756	407.366	334.114	76.575	113.424	53.52	59.205	166.525	-1.97184	down	9.21E-20
LOC12111	6.675	7.37	25.456	52.844	35.568	3.012	0.373	2.215	0.353	1.241	-4.15128	down	5.38E-64
GVINP1	13.324	13.946	18.782	19.544	16.333	1.373	5.031	6.353	0.794	16.693	-1.43757	down	1.28E-10
LOC77027	0.949	1.031	0.455	0.826	1.47	0.137	0.058	0.217	0.15	0.423	-2.25817	down	3.66E-21
NUGGC	0.893	0.71	1.748	0.503	0.446	2.847	1.423	1.7	1.534	1.139	1.006353	up	5.85E-07
PBK	0.135	0.159	0.086	0.133	0.138	2.258	0.524	0.338	0.502	0.347	2.598824	up	4.97E-19
LOC42638	0.052	0.183	0.718	0.776	0.394	1.808	1.337	1.292	1.054	0.545	1.505289	up	1.83E-12
SOX7	3.242	3.065	2.072	1.882	2.475	2.122	12.415	29.88	3.526	13.069	2.259733	up	5.70E-28
BLK	0.486	0.712	1.101	0.58	0.771	2.516	4.199	2.116	0.947	3.225	1.831456	up	4.76E-18
FDFT1	37.273	38.699	4.171	6.444	60.069	39.912	139.499	123.093	10.416	43.839	1.282485	up	6.14E-11
MCM3	0.205	0.191	0.262	0.324	0.466	4.603	1.967	0.628	0.582	0.29	2.474428	up	1.07E-27
CRISP2	0.086	0.075	2.056	1.533	0.56	0.065	0.205	0.139	0.292	0.285	-2.1224	down	4.14E-13
RHAG	1.114	1.21	2.461	0.895	1.691	0.115	0.104	0.513	0.386	0.377	-2.29788	down	7.50E-18
CYP2AC1	632.367	539.155	1236.022	975.454	1033.102	112.742	67.787	188.38	278.751	424.841	-2.04179	down	2.61E-20
RCAN2	2.931	2.809	2.735	5.073	3.064	1.106	0.893	1.022	1.228	1.226	-1.60041	down	1.28E-13
ADGRF5	2.642	2.89	2.883	2.995	1.282	0.796	0.821	1.012	1.022	0.511	-1.60741	down	1.65E-13
OPHN1	2.993	2.726	4.357	3.718	4.075	1.753	1.78	1.411	1.827	1.614	-1.09112	down	8.38E-07
ARR3	1.942	1.912	0.361	1.283	1.343	0.308	0.279	0.123	0.615	0.792	-1.68984	down	3.06E-13
KIF4B	0.161	0.146	0.335	0.14	0.257	1.712	0.586	0.389	0.455	0.337	1.738623	up	2.08E-14
CCNB3	0.287	0.137	0.458	0.193	0.295	1.45	0.347	0.631	0.619	0.466	1.355324	up	6.58E-08
LOC4287C	9.701	12.224	15.971	9.433	16.981	7.947	4.627	5.36	2.958	9.374	-1.08722	down	1.30E-06
SLC6A14	0.466	0.499	0.399	2.202	1.155	1.649	9.661	3.763	2.21	23.206	3.099017	up	7.08E-45
FAM162BI	1.129	1.318	2.071	3.171	3.112	4.272	4.429	4.083	3.751	8.234	1.196994	up	2.72E-09
GPC3	0.465	0.397	0.123	0.324	0.362	0.287	1.702	0.885	0.979	1.16	1.58209	up	3.47E-12
NSDHL	29.648	29.047	3.061	3.52	36.354	24.747	81.906	87.987	10.473	12.993	1.101665	up	1.35E-08
TRPC5	0.089	0.079	0.564	0.559	0.091	0.917	0.301	0.144	1.378	0.28	1.124967	up	1.88E-07
LOC12111	1.993	1.467	3.592	1.296	2.075	8.116	6.426	2.752	8.013	3.513	1.466858	up	2.11E-12
SH2D1A	1.818	1.263	2.994	1.244	1.728	7.544	5.903	2.224	7.42	3.282	1.543027	up	6.71E-14
RXFP1	0.169	0.153	0.661	0.267	0.333	0.867	0.757	0.589	1.187	0.695	1.368413	up	3.67E-10

APELA	35.455	33.235	49.143	85.758	30.52	189.728	112.628	108.794	83.382	122.956	1.399199 up	1.20E-11
MSMO1	201.642	197.348	11.709	32.025	267.106	195.206	551.187	513.451	55.822	106.537	1.002577 up	2.28E-07
TBC1D9	0.493	0.451	0.835	0.353	1.96	1.363	2.211	3.378	2.528	2.116	1.501609 up	2.56E-14
SLC20A2	29.565	28.28	29.227	25.668	42.152	11.598	8.855	16.397	20.818	10.2	-1.1904 down	4.74E-08
CYP2U1	17.059	17.236	31.779	23.594	35.501	60.739	34.293	52.018	76.616	30.302	1.02074 up	1.63E-07
ARHGEF38	1.002	0.876	0.197	0.258	0.592	0.246	0.214	0.072	0.076	0.096	-2.04704 down	7.15E-18
LRP2BP	1.878	1.793	1.608	1.752	1.209	0.345	0.727	0.225	0.378	0.503	-1.91721 down	3.78E-17
ACSL1	167.114	151.745	183.9	262.414	249.984	40.506	24.44	39.468	55.84	97.486	-1.97769 down	6.92E-20
DCTD	8.876	9.32	6.528	9.438	11.396	3.069	1.542	2.077	2.381	1.848	-2.06063 down	2.96E-21
GPAT3	7.264	7.386	8.397	9.107	17.669	3.902	3.187	1.85	4.248	4.389	-1.50294 down	4.19E-12
DUSP4	0.599	1.122	0.629	0.47	1.118	2.065	3.007	2.838	0.461	0.567	1.181465 up	4.10E-08
LOC43030	0.074	0.101	0.177	0.084	0.065	6.617	1.081	0.623	4.575	0.72	4.750551 up	2.62E-79
SHROOM3	0.496	0.407	1.226	0.995	0.284	0.303	0.175	0.301	0.334	0.374	-1.19379 down	2.25E-07
NAAA	2.407	2.572	5.92	3.516	4.807	21.651	4.76	7.771	11.638	4.801	1.396745 up	1.27E-11
SULT1B	30.586	33.569	49.609	13.958	87.131	6.606	5.638	7.81	16.864	7.224	-2.283 down	1.60E-24
FABP2	0	0	0	1.883	0.102	0	0	0.192	0.169	0.099	-2.09747 down	5.09E-05
ENPEP	8.84	8.815	9.553	7.139	5.576	14.277	31.891	18.308	7.278	18.556	1.177565 up	2.42E-09
CCDC110	0.033	0	0	0.26	0.017	0.453	1.586	0.354	0.395	0.463	3.369677 up	3.75E-30
FAT1	0.372	0.457	0.678	0.415	0.456	3.303	4.236	3.207	2.901	2.21	2.734724 up	2.54E-37
PDGFRL	1.353	1.265	4.402	0.842	1.012	0.832	0.356	0.224	0.491	0.594	-1.82732 down	7.65E-14
BEND4	0.055	0.015	0.031	0.173	0.128	0.212	0.104	0.18	1.002	0.07	1.950418 up	2.40E-13
CCKAR	0.033	0.011	0.106	0	0	0.113	3.324	0.45	0	0.099	4.68641 up	1.52E-50
RBPJ	16.543	15.854	18.495	3.857	20.887	2.047	2.934	2.592	4.5	3.562	-2.27393 down	9.52E-25
PPARGC1A	6.235	5.475	3.024	3.071	5.326	1.803	0.549	2.119	4.343	1.294	-1.19393 down	1.66E-08
SPON2	0.232	0.346	0.632	3.372	0.765	1.105	18.347	9.839	0.476	62.306	4.104706 up	1.20E-68
SMYD1	1.567	1.467	0.174	1.386	0.571	0.944	5.013	4.042	0.302	9.17	1.913461 up	1.80E-18
ADRA1D	0.198	0.328	0.399	0.206	1.296	2.246	1.182	4.372	9.225	0.377	2.839452 up	2.59E-38
NAT8	10.089	11.661	7.855	21.419	23.949	5.395	2.064	11.147	6.644	4.781	-1.31977 down	3.46E-09
LOC43042	0.19	0.304	0.613	0.303	0.444	0.788	1.316	0.733	1.553	0.889	1.507104 up	2.12E-12
LOC10705	0.718	0.515	1.035	0.787	0.738	3.059	3.254	2.311	1.737	3.392	1.856959 up	1.65E-16
CD6	0.451	0.307	0.719	0.143	0.41	1.415	0.639	0.57	0.97	0.499	1.009891 up	5.74E-06
TCIRG1	11.835	12.294	15.393	16.677	12.762	23.807	39.235	39.121	13.128	56.92	1.320262 up	2.34E-11
SPTB	1.182	1.253	2.213	0.42	1.11	0.344	0.227	0.545	0.58	0.44	-1.53002 down	3.25E-11
LUZP2	0.036	0.071	0.215	0	0.037	0.102	0	2.343	0.7	0.749	3.421094 up	3.49E-36
DHCR7	20.697	21.731	2.016	2.903	33.831	26.964	65.285	80.303	11.188	17.493	1.309653 up	2.51E-11
E2F8	0.192	0.203	0.289	0.412	0.164	1.934	0.754	0.402	0.542	0.339	1.65218 up	2.04E-12
RCN1	5.229	5.745	5.444	5.877	7.058	17.237	14.608	7.792	9.703	9.9	1.01294 up	6.07E-07
PRRG4	1.373	1.063	4.372	1.151	2.014	6.741	7.108	6.789	2.87	3.946	1.460457 up	6.47E-14

OVCH1	1.939	2.019	1.478	6.119	0.603	0.063	0.272	1.519	0.525	1.132	-1.79049	down	4.57E-16
MICAL2	0.179	0.201	0.409	0.404	0.156	0.628	1.496	0.837	0.464	0.689	1.605066	up	3.53E-15
CALCA	3.594	2.378	11.033	2.739	2.269	1.569	1.808	1.628	2.142	1.613	-1.32886	down	2.14E-08
USH1C	0.565	0.732	0.705	0.752	0.89	1.698	1.364	1.748	3.511	1.024	1.357465	up	1.41E-10
SAA	18.068	17.014	34.513	38.923	20.811	72.612	159.088	40.735	88.162	176.564	2.054266	up	5.88E-23
TMEM86A	132.445	119.628	82.819	229.35	159.732	22.421	10.231	47.227	92.146	49.602	-1.70778	down	8.32E-16
KCNQ1	1.006	0.859	0.601	0.536	0.536	1.613	1.543	1.798	0.788	2.041	1.136281	up	4.13E-08
TRPM5	0.198	0.373	0.151	0.965	0.144	5.803	3.078	9.272	0.21	3.648	3.583914	up	8.76E-54
SYT8	0.099	0.016	0.632	0.041	2.063	0.168	0.076	0.191	0.084	0.246	-1.89107	down	5.09E-09
CTSD	96.128	125.637	250.72	233.262	767.082	167.902	217.654	82.576	120.839	121.602	-1.05153	down	2.29E-06
CHKA	20.577	15.45	22.142	9.008	3.506	23.065	45.735	44.327	7.754	85.201	1.543716	up	6.55E-15
CPT1A	107.933	94.737	63.556	70.728	127.52	12.765	11.441	15.539	6.06	50.468	-2.27034	down	4.63E-25
SMTNL1	0.484	0.481	0.17	1.098	0.795	0.044	0.099	0.355	0.393	0.326	-1.3115	down	5.59E-07
PGR2/3	0.309	0.121	0.147	1.826	0.127	0	0.126	0.06	0.104	0	-3.1032	down	6.77E-12
SMIM38	1.844	1.327	0.147	1.819	0.474	0	0	0	0	0	-10.1334	down	1.94E-25
FGF19	0.264	0.104	0.196	0.174	0.036	0.858	5.821	3.112	0.209	1.171	3.842637	up	2.84E-52
HSD17B12	62.841	63.515	54.042	59.362	45.414	150.562	140.273	129.654	82.814	76.763	1.024362	up	3.27E-07
ACCS	6.002	4.903	2.753	3.512	1.412	1.812	5.657	5.567	11.746	25.272	1.429336	up	4.57E-12
MDK	0.867	1.267	3.207	3.519	3.744	0.981	0.391	0.689	1.338	0.758	-1.5991	down	1.20E-11
CHAC1	25.729	26.957	18.29	109.452	33.208	13.942	5.668	5.861	10.676	15.833	-2.03902	down	2.58E-22
NUSAP1	0.097	0.255	0.245	0.373	0.183	1.341	0.448	0.235	0.714	0.257	1.373327	up	7.82E-08
SPTBN5	0	0	0.003	0.035	0	0	0.017	0.06	0.011	1.806	5.464759	up	1.79E-75
EHD4	0.987	0.714	1.411	1.028	0.875	2.027	2.81	2.948	2.232	2.877	1.3615	up	9.03E-12
GALNT16	0.883	0.742	1.649	1.57	0.711	0.19	0.491	0.272	0.412	1.268	-1.07564	down	3.89E-06
RDH11	4.659	5.179	3.221	1.487	5.496	5.435	23.507	16.82	2.079	3.762	1.364208	up	4.34E-12
LGALSL2	0.62	0.766	1.617	0.324	1.405	0.271	0.202	0.354	0.31	0.112	-1.91744	down	7.07E-14
DTD2	20.628	21.732	17.03	14.625	7.478	7.099	5.793	7.663	7.448	7.321	-1.20591	down	2.88E-08
TTC6	0.807	0.437	2.69	0.156	0.257	0.116	0.271	0.112	0.267	0.267	-2.06787	down	1.25E-17
ACSS1B	42.926	41.52	46.602	47.98	76.447	4.774	13.978	12.278	10.933	19.583	-2.05336	down	1.48E-20
FOS	4.941	4.658	12.706	1.15	4.038	2.266	1.395	3.317	2.009	1.774	-1.35284	down	1.27E-08
FLVCR2	0.555	0.363	0.771	0.585	1.293	0.562	6.759	0.575	0.673	8.014	2.215336	up	4.81E-26
DIO2	49.66	52.581	203.045	10.549	71.573	483.769	555.359	349.563	265.855	59.932	2.145829	up	1.01E-26
GALC	0.376	0.449	0.833	0.893	1.745	0.278	0.652	0.213	0.36	0.438	-1.14416	down	1.41E-06
SPIA3	23.911	21.353	55.387	26.868	31.373	78.152	151.597	99.443	107.686	438.762	2.462254	up	1.47E-32
BDKRB1	0.133	0.237	0.303	0.219	0.098	2.092	3.839	11.458	0.29	0.34	4.179079	up	1.12E-63
LOC12111	3.707	3.001	0.024	3.645	0	0	0	0	0	0	-11.0199	down	1.55E-72
DEGS2	0.522	0.63	0.949	0.369	0.763	1.333	1.655	1.143	1.906	0.62	1.040852	up	2.40E-07
SLC25A47	18.037	15.637	23.019	20.484	14.354	9.012	2.912	6.682	7.023	6.053	-1.53045	down	1.06E-12

DIO3	1.688	1.42	0.299	0.352	1.486	0.182	0.466	0.362	0.59	0.718	-1.17633	down	2.98E-06
ANKRD9	2.95	3.165	5.967	2.546	9.757	29.264	24.054	58.018	12.839	11.266	2.473356	up	6.43E-33
APOPT1	5.998	5.942	9.947	4.947	6.936	14.999	15.273	13.309	16.111	11.512	1.076104	up	3.93E-08
ASPG	18.309	17.346	24.985	31.987	42.107	12.542	10.48	10.875	10.813	14.642	-1.18268	down	5.96E-08
PLD4	0.179	0.096	0.478	0.199	0.249	1.001	0.463	0.376	0.624	0.334	1.216742	up	4.11E-08
DHRS7	868.149	797.527	1003.922	724.93	1188.741	373.206	348.602	314.081	584.164	494.667	-1.11591	down	2.73E-07
C14orf105	0.082	0.25	0.109	0.09	0.056	0.247	0.195	1.053	0	0.027	1.367031	up	3.50E-06
DLGAP5	0.257	0.168	0.303	0.322	0.349	1.562	0.642	0.517	0.854	0.347	1.483885	up	6.88E-11
GCH1	87.373	94.623	99.629	67.794	186.64	66.694	37.659	50.226	48.292	54.327	-1.0595	down	1.37E-06
CDKN3	0.665	0.479	0.185	4.291	0.786	0.904	0.68	1.124	0.225	0.198	-1.03162	down	1.69E-05
LOC12110	1.873	1.988	1.398	1.382	1.803	0.735	1.554	0.128	0.649	0.702	-1.16307	down	2.04E-07
GRID1	0.591	0.53	1.061	0.147	0.01	0.026	0.057	0.036	0.043	0.102	-3.12329	down	3.08E-37
SNCG	0.037	0.016	0.07	0.041	1.68	0.028	0.026	0.024	0.021	0.062	-3.47749	down	3.03E-24
ADIRF	0.06	0	0.048	0.147	0.123	0.473	0.122	1.267	0.202	0.296	2.626424	up	3.91E-11
FAM35A	3.565	3.083	2.749	3.501	3.209	8.006	7.905	9.961	10.855	2.066	1.267847	up	4.10E-10
DNAJC12	47.04	41.616	64.729	49.829	55.959	19.129	10.369	10.815	15.223	28.404	-1.62643	down	6.49E-14
RTKN2	2.035	1.723	2.468	1.555	1.758	0.696	1.165	0.609	0.897	0.904	-1.15833	down	1.89E-07
PRAP1	0.624	1.679	3.203	1.881	1.496	11.701	3.476	4.218	13.506	3.714	2.042701	up	4.04E-19
HKDC1	0.042	0.172	0.528	0.207	0.076	28.209	158.752	27.056	25.945	0.668	7.868058	up	#######
SCD	143.146	170.585	16.981	51.148	16.442	598.792	605.881	1007.392	287.655	275.015	2.8004	up	1.71E-37
CYP2C23b	588.344	527.607	295.271	352.718	570.35	83.351	39.148	129.057	229.171	111.055	-1.97984	down	3.01E-20
CYP2C23a	1174.55	1044.819	1141.807	758.226	1003.909	252.729	162.357	422.425	1046.388	252.493	-1.2619	down	4.01E-09
LOC10085	589.347	629.937	1289.746	1120.126	1296.512	1878.656	1773.152	2637.083	3325.161	1520.785	1.176688	up	1.51E-09
ANXA8L1	0.235	0.095	0.144	0.372	0.186	4.018	1.537	2.797	6.162	1.041	3.907354	up	1.26E-53
PANK1	87.26	75.486	129.212	87.423	116.202	32.205	20.495	20.94	45.178	51.322	-1.54238	down	1.19E-12
PPP1R3C	27.675	27.046	37.051	57.057	54.179	103.981	75.165	137.15	84.31	38.621	1.113411	up	1.57E-08
CRTAC1	6.912	8.008	0.079	0.244	0.017	0.99	1.502	0.159	0.028	1.08	-2.01989	down	1.42E-20
HOGA1	4.35	4.49	2.699	2.135	5.657	1.055	0.867	1.582	2.344	2.241	-1.25636	down	3.51E-08
TLX1	0.975	1.072	0.882	0.947	0.046	0	0	0	0.15	0.088	-4.0144	down	1.36E-23
PDZD7	1.084	0.786	1.142	1.255	0.335	0.198	0.418	0.394	0.336	0.428	-1.37276	down	9.15E-09
CYP17A1	1.837	2.736	1.617	3.953	6.331	1.451	0.964	0.957	1.228	1.055	-1.54175	down	1.01E-11
STN1	0.283	0.516	0.439	0.869	1.469	1.501	2.453	0.503	1.715	1.27	1.056297	up	8.65E-07
GSTO2	5.481	5.978	4.51	2.385	10.692	3.183	1.506	2.555	3.904	1.219	-1.23151	down	1.29E-08
GPAM	50.126	44.294	35.149	35.867	21.312	157.974	276.741	183.855	51.984	93.401	2.032366	up	1.77E-22
SLC18A2	0.169	0.118	0.305	0.232	0.058	0.37	0.427	0.185	1.184	0.291	1.472825	up	1.78E-09
LOC10175	0.605	0.138	0.27	0.482	0.762	2.251	3.243	0.654	1.101	0.727	1.818971	up	7.93E-17
MKI67	0.168	0.176	0.41	0.273	0.327	2.315	0.834	0.536	0.871	0.402	1.868667	up	5.91E-18
C2orf88	0.235	0.165	0.319	0.522	4.145	0.4	0.415	0.222	0.657	0.181	-1.51982	down	3.96E-11

CPS1	1.805	1.697	1.321	1.582	1.761	0.532	0.614	1.182	0.736	0.684	-1.12247	down	5.19E-07
ABCA12	5.11	4.197	11.634	1.146	27.632	2.831	0.602	0.819	4.763	1.866	-2.19147	down	4.12E-23
ATIC	4205.008	4235.959	2567.995	3001.741	2519.42	991.928	929.182	1166.148	1963.965	2389.048	-1.15167	down	4.92E-08
LOC10704	2.429	8.065	4.108	4.732	2.095	0.441	0.951	0.956	0.508	1.934	-2.1603	down	1.99E-20
RAB17	4.15	3.431	3.02	7.218	6.459	1.787	0.313	2.409	1.121	0.581	-1.96589	down	6.98E-18
LOC12111	5.572	5.416	23.484	11.677	68.075	6.562	1.877	2.484	10.723	0.666	-2.35571	down	3.40E-26
AHR2	5.188	4.649	7.267	7.84	9.885	1.406	0.717	1.517	4.078	1.576	-1.90535	down	2.48E-18
AHR1B	4.608	3.848	6.309	2.694	3.261	0.403	0.642	0.235	0.65	0.892	-2.87403	down	1.63E-35
COL6A2	3.344	4.803	5.366	4.699	6.948	24.272	15.127	19.226	8.346	12.648	1.661785	up	3.17E-16
NABP1	6.99	5.47	11.952	4.284	6.75	20.719	23.054	8.845	10.804	12.83	1.105043	up	1.40E-08
COQ10B	31.709	32.659	22.38	37.282	58.093	8.771	6.21	6.684	11.885	13.577	-1.95017	down	2.06E-19
SGO2	1.704	1.395	0.717	1.761	1.607	0.607	0.373	0.662	0.562	0.776	-1.26806	down	1.39E-08
GPR1	2.92	2.966	4.328	3.504	5.74	2.317	1.982	1.827	1.648	1.94	-1.00185	down	7.70E-06
PDE11A	19.234	16.791	0.348	4.895	0.061	0.366	18.573	55.316	0.192	20.092	1.193657	up	1.67E-09
CDCA7	0.208	0.484	1.145	0.514	0.696	3.765	2.74	0.845	0.759	0.186	1.443356	up	7.20E-12
RAPGEF4	21.935	20.538	17.359	11.14	36.575	4.138	5.352	7.209	12.068	3.499	-1.73672	down	1.64E-15
SPC25	0.417	0.448	0.332	0.441	0.949	1.956	1.036	0.748	0.757	1.152	1.125206	up	1.91E-06
CERS6	0.521	0.63	0.077	0.368	0.429	1.638	0.533	0.573	1.117	0.648	1.152927	up	3.42E-07
DPP4	4.234	3.722	3.601	5.071	9.228	7.15	13.904	11.194	17.373	15.52	1.332899	up	3.04E-11
SLC4A10	0.187	0.138	0.278	0.215	0.281	0.818	0.669	0.631	1.065	0.766	1.840573	up	4.02E-17
ITGB6	0.971	0.877	0.5	0.447	0.438	7.98	21.905	2.774	0.551	5.905	3.594731	up	1.28E-56
LOC42419	0.762	0.715	1.71	0.465	1.364	0.243	0.06	0.302	0.132	0.504	-2.01067	down	7.95E-14
TUBA4AL	0.608	0.895	1.271	1.208	1.119	0.373	0.389	0.538	0.386	0.729	-1.07719	down	3.96E-05
IGFBP2	19.814	18.98	11.072	14.401	21.131	3.131	3.605	3.463	8.268	8.886	-1.64232	down	1.97E-14
SLC12A8	0.247	0.246	0.306	0.554	0.298	0.435	2.19	1.441	0.231	1.641	1.843489	up	9.42E-18
SCTR	4.74	4.324	0.287	2.085	0.549	2.707	5.888	8.779	0.249	13.363	1.370018	up	5.23E-11
DPP10	0.176	0.291	0.436	0.244	0.029	0.179	1.338	1.881	0.55	0.543	1.928633	up	4.91E-19
UPP2	40.223	37.093	61.261	47.83	61.272	39.112	16.797	7.597	24.556	26.223	-1.1158	down	1.39E-07
DAPL1	1.429	2.167	2.585	1.387	2.801	0.441	0.465	0.752	0.55	1.159	-1.6213	down	6.12E-09
RGS5	9.618	13.28	20.968	9.174	30.972	29.974	35.47	40.075	48.061	26.394	1.099075	up	8.41E-09
SELP	0.522	0.502	0.636	0.581	0.621	1.148	1.372	0.84	2.514	1.33	1.330257	up	5.32E-10
REG4	0.831	0.502	1.374	0.473	0.853	0	0.131	0.185	0	0.19	-2.98225	down	3.93E-13
FASLG	0.394	0.358	0.569	0.236	0.405	0.598	0.786	0.922	0.69	1.186	1.08992	up	5.53E-08
SUCO	6.512	6.015	8.369	5.144	6.028	17.744	19.366	14.933	4.859	13.22	1.128611	up	8.44E-09
NUF2	0.068	0.122	0.256	0.306	0.226	1.304	0.45	0.376	0.545	0.235	1.568233	up	6.31E-10
RGS8	6.23	6.334	1.454	1.004	4.903	0.373	0.51	0.433	0.11	2.246	-2.43834	down	2.96E-28
RGSL1	1.999	2.292	0.7	0.614	1.794	0.473	0.213	0.594	0.372	0.49	-1.78598	down	7.10E-15
TOR3A	22.63	20.597	13.745	7.774	1.6	16.462	49.049	49.393	2.723	64.146	1.453987	up	3.87E-13

KIAA0040	27.146	26.189	17.705	19.883	28.757	10.234	7.185	10.39	12.787	12.853	-1.16287	down	5.41E-08
CLCA1	0.1	0.033	0.03	0.164	0.026	0.354	0.703	0	0.021	3.505	3.679834	up	1.83E-42
LPAR3	0.723	0.746	3.989	2.287	2.613	8.344	5.034	4.716	3.458	6.361	1.429754	up	3.42E-13
VTG2	4938.704	5884.15	43.669	1565.017	4.114	4958.423	10314.09	10142.92	11.309	6504.932	1.360505	up	8.32E-11
VTG3	625.379	748.039	2.05	89.471	0.449	471.039	1458.83	1486.522	1.81	751.098	1.508519	up	4.48E-13
SPATA1	21.159	19.818	12.843	23.628	2.939	44.838	48.952	49.475	8.492	42.159	1.270345	up	6.27E-10
RBP	330.724	399.221	17.878	55.352	2.696	391.933	814.793	877.174	3.768	505.262	1.685956	up	9.13E-16
TSPAN1	6.873	6.38	8.724	4.707	3.56	3.359	16.466	18.913	8.057	14.187	1.011614	up	9.84E-08
VDHAP	1.251	0.844	1.074	0.787	1.064	0.266	0.26	0.434	0.298	0.659	-1.38652	down	5.23E-08
FAAH	128.693	126.67	82.223	114.864	123.711	51.546	55.691	58.035	40.907	67.43	-1.07434	down	5.24E-07
CYP4B7	400.359	358.574	324.755	325.616	466.218	40.948	44.423	58.98	62.789	162.722	-2.34222	down	1.71E-26
PODN	2.283	1.903	5.682	1.607	1.414	0.579	0.964	0.613	0.61	1.162	-1.713	down	4.98E-14
LEXM	0	0.063	0	0	0.231	0.547	1.086	0.683	0.163	0.223	3.178477	up	1.42E-20
DHCR24	22.675	25.84	0.604	0.787	51.673	53.461	134.302	100.96	18.307	18.366	1.679545	up	8.15E-17
DAB1	0.954	0.815	3.121	1.119	2.971	0.592	1.051	0.557	0.803	0.585	-1.32233	down	1.40E-08
CYP2J21	25.335	21.882	0.637	57.917	23.295	59.991	37.732	72.078	55.682	66.401	1.177255	up	2.49E-08
KANK4	4.823	5.238	3.25	8.253	10.794	15.651	13.566	15.318	7.276	14.231	1.029148	up	2.52E-07
ANGPTL3	47.918	48.945	26.193	51.599	48.217	160.629	206.205	193.833	64.167	105.86	1.713029	up	1.02E-16
DNAJC6	2.165	1.793	2.369	0.473	3.857	1.329	0.418	1.74	0.962	0.871	-1.00162	down	2.02E-05
PTGER3	0.949	0.873	0.52	2.608	1.158	0.134	0.455	0.115	0.452	0.176	-2.19288	down	6.54E-17
KLHL24	23.395	22.179	23.13	14.804	19.244	10.129	7.363	7.189	10.282	8.025	-1.25706	down	4.58E-09
GPC2	0.672	0.742	1.413	0.707	1.554	3.462	1.128	1.178	2.86	1.881	1.045724	up	1.64E-07
EHHADH	274.411	257.616	291.099	309.829	356.991	102.712	77.039	50.263	180.011	134.585	-1.45196	down	7.57E-12
DNAJB11	20.71	21.364	20.588	17.848	19.015	57.265	61.655	27.732	29.816	36.927	1.100357	up	5.49E-08
LOC10174	1.723	1.618	3.961	1.096	0.913	0.503	1.052	0.134	1.365	0.496	-1.38986	down	1.19E-08
SGPP2	0.885	0.962	0.263	1.936	3.467	0.051	0.307	0.377	0.178	0.223	-2.72005	down	4.41E-27
MOGAT1	1.422	1.726	2.128	2.632	8.621	1.086	1.349	0.48	3.597	1.342	-1.07302	down	1.49E-06
PCOLCE2	2.885	2.765	3.479	2.576	4.171	1.6	1.081	0.997	2.413	1.731	-1.02077	down	3.79E-06
PDCD1	0.224	0.244	0.436	0.245	0.332	1.521	0.56	0.36	0.421	0.617	1.229311	up	7.26E-07
LOC42491	4.145	3.584	2.734	3.011	5.463	1.41	1.874	1.511	2.109	2.011	-1.08647	down	1.06E-06
C2orf72	45.018	46.835	41.902	41.539	44.64	20.037	10.983	20.971	24.602	21.58	-1.16363	down	6.10E-08
ALPI	2.276	2.978	1.982	7.011	6.817	1.253	0.392	1.54	1.189	0.717	-2.04768	down	1.78E-19
VWA5B2	2.086	2.077	3.704	2.969	1.017	0.754	1.022	0.925	1.1	1.602	-1.13269	down	4.58E-07
AHSG	647.406	586.191	341.982	385.887	516.601	498.171	1356.407	3487.775	930.332	1222.916	1.596827	up	7.51E-16
ECT2	0.326	0.373	0.202	0.082	0.205	1.129	0.575	0.315	0.199	0.35	1.108857	up	1.71E-06
GHSR	0.047	0.122	0.13	0.269	0.096	1.537	0.311	0.384	0.674	0.302	2.263843	up	3.40E-15
SERPINI1	0.052	0.102	0.316	0.227	0.372	1.114	1.059	0.183	0.614	0.428	1.663813	up	8.55E-12
LOC10705	0.51	0.516	1.065	0.257	2.652	0.081	0.454	0.746	0.13	0.256	-1.5818	down	1.57E-10

SMC4	0.936	0.823	1.281	0.925	1.01	6.352	2.381	2.265	2.867	1.689	1.643532	up	7.41E-15
IFT80	2.329	2.327	3.494	2.548	2.656	12.946	5.686	5.694	4.24	3.61	1.268397	up	5.94E-10
MLF1	1.58	1.77	10.575	6.262	8.683	1.408	1.197	0.946	1.263	1.219	-2.25768	down	1.09E-22
TUBA3E	0.192	0.163	0.031	0	0.099	0.152	1.021	1.056	0	0.038	2.213109	up	1.01E-15
DUOX1	0.118	0.177	0.05	0.051	0	1.278	0.574	3.59	0.586	0.742	4.078547	up	3.63E-60
KIAA0101	2.021	2.952	1.287	0.996	2.421	4.118	10.818	15.801	0.798	2.071	1.795556	up	9.99E-17
CHRNA3	0.746	0.418	1.464	0.81	0.164	0.241	0.245	0.257	0.451	0.529	-1.0617	down	0.000135
TRPM1	0.314	0.362	0.075	0.487	0.137	0.666	2.468	1.489	0.193	1.704	2.24131	up	1.19E-24
LIPC	10.231	9.369	17.981	17.582	21.798	0.453	1.581	0.296	1.466	1.44	-3.87631	down	2.88E-60
DYX1C1	2.47	2.886	2.81	3.534	3.216	0.899	1.331	1.277	1.83	1.597	-1.10454	down	1.14E-06
SCG3	0.277	0.258	0.134	0.235	0.216	0.285	0.67	0.178	0.469	1.133	1.284251	up	1.24E-08
SEMA6D	5.433	4.993	4.704	5.885	5.547	1.207	1.069	1.664	3.317	2.453	-1.45135	down	1.23E-11
GATM	49.717	47.347	38.575	28.987	47.337	93.461	97.016	146.527	97.738	27.973	1.126293	up	1.06E-08
FSD2	1.02	0.747	1.987	0.602	0.74	0.452	0.465	0.439	0.62	0.308	-1.15606	down	1.90E-06
LMNAL	0.118	0.145	0.135	0.634	0.477	0.592	0.716	0.846	0.775	1.245	1.464793	up	1.12E-11
LOC41547	0.36	0.319	0.574	0.463	0.388	1.875	1.688	1.346	1.682	1.331	1.910216	up	2.61E-19
PEX11A	28.78	25.19	13.222	20.54	14.935	3.615	10.017	7.78	1.368	17.946	-1.33384	down	6.47E-10
ADAMTS1	1.168	1.212	0.396	1.154	0.274	1.323	3.367	1.921	0.632	2.921	1.272628	up	1.17E-09
ALDH1A3	0.407	0.44	0.452	0.311	0.185	0.407	0.774	2.622	0.096	0.235	1.201285	up	2.02E-09
KIF23	0.095	0.124	0.14	0.258	0.15	0.803	0.643	0.176	0.297	1.037	1.939412	up	5.49E-17
CNGB1	0.177	0.252	0.256	0.185	0.165	0.4	0.32	0.69	1.075	0.135	1.335734	up	3.09E-09
CETP	25.686	27.129	125.438	13.017	74.071	16.97	0.893	2.012	62.52	2.264	-1.64805	down	7.94E-14
CX3CL1	8.187	8.904	5.292	11.119	18.702	5.147	1.822	7.263	7.967	3.401	-1.02787	down	2.68E-06
KIAA0895I	3.127	2.669	3.743	2.059	2.17	1.43	0.911	0.8	1.333	1.458	-1.21404	down	1.00E-07
LOC76966	157.557	155.461	204.823	77.302	74.399	55.941	30.583	78.649	50.393	33.503	-1.42661	down	1.04E-10
LOC41566	31.199	32.997	32.033	30.862	30.096	13.675	11.884	16.268	11.005	22.93	-1.05289	down	1.59E-06
LOC10085	673.037	787.092	345.96	334.899	411.752	193.712	177.507	302.472	179.142	248.954	-1.2122	down	1.42E-08
MT4	711.399	809.728	1038.719	734.886	291.299	28.4	222.373	39.099	77.222	654.22	-1.81196	down	1.35E-16
MT3	310.257	378.472	491.141	215.847	100.131	6.797	97.399	13.188	18.926	565.561	-1.09168	down	1.38E-06
CDCA9	20.937	20.591	13.664	15.924	17.513	7.5	4.312	6.5	13.208	7.682	-1.17675	down	2.84E-08
RGS9BP	1.13	1.118	2.26	1.351	2.091	0.867	0.573	0.586	0.87	0.796	-1.10551	down	1.57E-06
PDCD2L	9.731	8.954	15.511	6.181	15.106	26.137	9.536	15.597	56.696	17.331	1.175162	up	3.86E-09
CES1L2	38.947	39.648	81.674	126.021	111.344	25.02	29.577	17.252	11.35	20.481	-1.93925	down	4.33E-19
CES1L1	111.626	109.5	127.726	130.396	178.969	90.662	95.019	58.683	25.74	56.14	-1.0126	down	3.02E-06
TERB1	3.686	3.169	6.137	3.53	1.611	0.862	1.075	0.5	1.161	0.635	-2.09756	down	3.03E-20
BEAN1	0.894	0.509	0.588	0.831	0.599	1.585	2.625	1.014	2.568	2.545	1.593918	up	1.66E-11
CENPN	0.316	0.447	0.405	0.375	0.612	1.588	1.166	0.66	0.831	1.113	1.312009	up	7.06E-09
GINS2	0.197	0.297	0.676	0.237	0.436	1.613	0.899	0.468	0.873	0.496	1.236377	up	2.89E-08

CA5A	214.2	216.023	174.242	126.055	187.64	60.389	44.678	114.103	105.318	52.922	-1.2826	down	3.66E-09
ZFPM1	15.468	15.391	15.633	9.886	14.325	6.229	4.379	7.041	5.872	7.81	-1.17405	down	8.40E-08
CIDEC	25.144	27.625	46.895	23.439	53.649	9.743	5.64	13.383	10.091	15.646	-1.69723	down	3.73E-14
CDT1	0.437	0.767	1.142	0.632	1.276	4.199	2.04	1.452	0.711	1.318	1.191184	up	1.68E-08
SPIRE2	9.4	10.136	19.47	2.665	10.706	3.049	1.74	6.43	8.798	5.185	-1.05525	down	6.57E-06
URAH	4.095	5.451	2.337	2.956	5.202	0.161	0.702	1.257	0.281	0.939	-2.58324	down	5.45E-27
CHST4	7.214	6.093	8.923	6.024	4.14	2.596	2.434	2.587	3.657	4.335	-1.05311	down	1.52E-06
LOC1211C	0.174	0.228	15.743	0.613	18.161	0.817	0.076	0.672	0.042	0	-4.4373	down	4.20E-62
MUSTN1	1.16	0.901	1.501	1.907	1.043	0.082	0.445	0.07	0.491	0.647	-1.90512	down	1.47E-09
CISH	9.888	11.063	24.749	31.079	68.892	91.601	99.798	109.655	91.715	85.513	1.715113	up	1.25E-17
LOC1211C	5.142	5.183	15.106	2.359	0.336	2.078	1.636	1.298	1.108	1.766	-1.83388	down	2.32E-15
LOC1211C	2.474	2.67	6.941	1.457	0.379	0.481	0.151	0.107	0.188	0.165	-3.66615	down	1.33E-45
LOC1211C	2.451	2.595	15.727	11.153	0.737	7.65	1.733	1.539	1.721	1.364	-1.22122	down	2.09E-08
LOC1211C	0.236	0.288	1.114	0.723	0.129	1.05	1.399	1.229	0.586	1.528	1.216267	up	5.61E-09
USP4	34.851	31.939	33.217	25.706	30.698	8.776	11.129	10.087	8.642	16.04	-1.51633	down	3.20E-12
GPX1	84.297	82.259	179.925	76.365	144.983	58.872	47.226	49.308	55.016	67.315	-1.03172	down	5.40E-06
ALAS1	50.567	46.966	63.98	31.99	55.316	19.643	24.203	24.227	14.335	27.682	-1.17638	down	1.35E-07
PTPDC1	1.203	0.904	0.404	0.457	0.725	0.27	0.234	0.288	0.269	0.197	-1.54989	down	5.64E-11
DNASE1L3	5.72	5.065	11.301	8.801	1.546	22.061	16.499	12.83	19.664	12.157	1.359176	up	3.86E-11
ABHD6	87.008	72.637	40.937	70.693	36.2	16.266	15.231	17.733	3.754	22.219	-2.03153	down	3.67E-21
IP6K2	70.166	60.534	91.078	45.614	15.293	14.238	16.362	16.762	31.211	27.848	-1.40937	down	9.11E-11
MCM2	1.357	1.178	1.039	1.587	1.152	4.954	3.443	1.15	1.856	1.251	1.002624	up	2.85E-06
GPR27	0.309	0.392	0.087	0.463	0.149	1.647	0.893	0.948	0.431	1.172	1.858795	up	2.95E-14
PROK2	0.259	0.207	0.046	0.118	0	0.879	1.088	1.055	0.097	1.226	2.776187	up	3.71E-22
SHQ1	1.429	1.269	1.196	0.851	1.468	6.258	0.94	0.943	3.26	1.07	1.004633	up	5.17E-06
CNTN4	0.072	0.089	0.267	0.223	0.213	0.326	0.327	0.316	1.15	0.285	1.471006	up	1.61E-10
BHLHE40	14.579	15.981	27.992	29.928	54.212	12.91	10.914	11.784	8.473	9.483	-1.41348	down	1.40E-10
GHRL	0.524	0.374	1.213	0.782	0.831	0.323	0.292	0.092	0.242	0.472	-1.38681	down	1.21E-05
HBEGF	30.34	31.798	43.511	22.292	20.809	15.055	14.946	15.468	13.525	11.448	-1.07833	down	1.27E-06
KCNMB1	2.165	2.251	3.823	3.079	2.85	16.309	11.782	4.528	6.391	1.707	1.522663	up	5.44E-13
TENM2	2.348	2.093	5.216	7.734	11.746	0.603	0.14	0.016	0.646	0.381	-4.02426	down	3.48E-67
GABRB2	0	0	1.108	0	0.028	0.004	0.014	0.086	0.006	0.007	-3.22535	down	1.44E-27
PTTG2	0.163	0.266	0.486	0.201	0.251	1.336	1.04	0.393	0.551	0.403	1.442121	up	1.61E-07
SLC26A2	5.439	5.176	4.765	3.805	3.095	11.036	10.55	13.523	10.097	12.225	1.365882	up	1.23E-11
PPARGC1E	2.895	2.622	3.296	3.963	3.711	1.518	0.994	1.114	1.251	1.792	-1.30514	down	1.71E-09
DUSP1	33.482	31.109	90.424	20.028	34.609	13.714	16.092	24.269	25.735	19	-1.08523	down	4.07E-06
STC2	18.675	18.883	15.306	31.688	29.823	13.457	5.593	7.862	5.799	4.11	-1.63504	down	1.55E-14
MXD3	0.861	1.164	1.964	0.786	1.454	0.411	0.352	0.388	0.227	0.285	-1.90204	down	7.09E-14

NIPAL4	13.302	12.474	17.19	10.635	16.111	4.205	8.519	6.903	3.168	11.833	-1.00936	down	9.66E-06
TRPC7	0.315	0.344	1.872	1.381	0.405	0.357	0.04	0.114	0.756	0.482	-1.30105	down	5.37E-08
SLC25A48	0.412	0.413	0.579	0.905	0.698	2.547	1.4	1.331	1.086	0.759	1.242776	up	6.33E-09
SLC22A5	40.76	42.726	34.423	30.492	37.282	11.801	12.725	11.207	9.045	31.103	-1.29097	down	2.63E-09
EGR1	81.128	64.473	88.761	3.013	5.115	17.532	14.954	3.694	4.934	2.033	-2.49046	down	4.97E-30
BHLHA15	0.159	0.415	0.505	0.326	0.163	1.707	8.926	0.613	0.269	1.731	3.074511	up	1.38E-26
NPTX2	0.437	0.313	0.673	0.648	1.722	0.479	0.36	0.204	0.262	0.279	-1.25712	down	5.37E-07
CYP3A5	1487.008	1384.543	900.646	1710.507	882.877	838.664	527.106	718.703	600.335	421.572	-1.03506	down	6.20E-07
LOC10174	1.163	1.458	0.216	0.653	0.756	0.834	3.449	3.573	0.23	3.632	1.463468	up	8.17E-12
PEMT	160.853	158.596	118.057	108.361	86.333	285.296	302.234	334.265	64.057	290.833	1.013944	up	2.72E-07
CACNA1H	0.06	0.042	0.158	0.103	0.147	0.073	0.251	1.063	0.086	0.197	1.701517	up	4.05E-16
SOX8	0.807	0.688	2.121	0.341	1.305	0.637	0.625	0.566	0.124	0.485	-1.10892	down	5.92E-05
MEIOB	1.591	1.449	0.294	0.209	0.039	0	0.143	0.43	0.108	0.177	-2.05535	down	7.81E-16
PLK1	0.239	0.073	0.279	0.157	0.425	1.477	0.342	0.246	0.619	0.331	1.358209	up	2.05E-08
IL21R	0.252	0.279	0.617	0.485	0.398	1.699	0.762	0.589	1.196	0.614	1.256702	up	6.76E-09
RMI2	16.163	14.381	11.234	5.409	5.134	4.248	3.263	3.297	7.056	5.57	-1.15862	down	4.98E-08
GRIN2A	0.055	0.015	0.03	0.006	0.005	0.011	0.834	1.517	4.143	0.007	5.812011	up	#######
RHBDF1	1.817	1.82	3.071	2.347	1.169	5.265	10.8	4.679	5.378	7.454	1.71498	up	3.57E-17
HBAD	93.174	97.936	309.947	23.94	123.835	34.779	19.679	57.395	20.778	42.241	-1.89152	down	3.67E-16
HBA1	365.186	383.036	1151.922	127.271	387.325	109.165	60.76	183.864	113.451	157.73	-1.95	down	1.86E-17
PDIA2	0.22	0.352	0.575	0.382	0.314	1.137	2.956	2.388	0.394	1.554	2.190252	up	8.41E-24
RGS11	0.095	0.046	0.357	0.116	0.243	0.535	1.257	0.502	0.2	0.445	1.772018	up	3.14E-12
LOC41665	7.203	7.536	6.535	1.798	2.021	0.043	0.047	0.191	0.077	0.196	-5.48858	down	9.97E-95
C16orf96	1.74	1.911	1.353	1.788	1.298	0.633	0.36	0.9	0.912	0.822	-1.15627	down	7.09E-07
LOC12110	1.23	1.319	0.49	1.095	0.035	0.619	0.035	0	0.116	0.068	-2.30783	down	5.02E-16
GPRC5B	5.146	4.835	6.084	6.555	6.005	3.666	1.119	2.629	2.844	1.368	-1.29955	down	1.36E-09
SDF2L1	19.198	16.741	25.485	18.423	18.859	62.544	76.897	22.084	25.208	46.558	1.240879	up	7.12E-10
CDC45	0.14	0.165	0.367	0.161	0.287	1.33	0.515	0.297	0.544	0.333	1.426533	up	2.02E-09
AACS	7.247	6.549	6.818	7.214	4.705	29.561	33.803	32.778	13.811	14.601	1.936632	up	1.02E-20
RAD9B	1.605	1.591	1.683	1.202	0.539	0.27	0.098	0.107	0.431	0.315	-2.43396	down	4.18E-23
PPTC7	26.451	23.969	28.714	22.315	24.565	8.467	5.961	6.495	8.824	12.295	-1.58357	down	2.27E-13
TMEM116	11.563	10.646	6.492	14.387	17.031	4.892	2.625	5.472	11.633	5.026	-1.01976	down	1.41E-06
UNG	10.68	12.674	7.614	8.913	9.779	3.14	2.371	2.518	1.682	6.282	-1.63434	down	1.95E-13
ACACB	1.373	1.365	0.944	2.486	1.391	0.026	0.134	0.018	0.04	1.579	-2.06955	down	1.81E-20
CRYBB3	0.418	0.41	1.289	0.129	0.429	0	0	0.252	0.088	0.414	-1.82006	down	4.57E-06
VPS29L	2.106	2.448	1.502	1.318	6.329	0.819	0.804	0.607	0.799	1.809	-1.50105	down	4.00E-10
SLC2A11L	3.606	3.592	3.219	2.567	6.134	0.832	1.034	1.331	1.556	2.096	-1.48029	down	1.24E-10
SLC5A1	0.49	0.334	0.639	0.377	0.557	1.098	1.653	0.664	1.006	0.383	1.001501	up	1.69E-06

LOC41695	1.175	1.185	0.618	1.032	0.658	2.244	3.417	3.341	0.668	1.672	1.27989 up	3.67E-10
PISD	20.194	19.222	12.543	12.178	6.629	40.899	72.75	50.289	8.22	27.89	1.499152 up	2.00E-13
LOC10085	4.583	5.225	3.024	4.197	7.868	0.964	1.406	2.844	0.998	1.169	-1.7534 down	1.30E-12
PRODH	0.862	0.852	4.639	2.415	3.652	11.897	3.529	3.449	6.676	11.119	1.561551 up	1.06E-14
SLC35E4	0.209	0.239	0.312	0.204	0.174	0.916	0.561	1.537	0.088	0.311	1.580327 up	1.77E-12
TCN2	106.731	126.53	35.771	38.128	31.915	98.267	199.566	250.832	22.431	111.908	1.010279 up	3.35E-07
MTFP1	5.55	5.931	6.95	4.004	8.355	1.919	1.95	3.763	2.798	2.479	-1.25376 down	9.51E-08
HORMAD1	1.423	1.088	1.587	0.976	1.965	0.618	0.347	0.292	0.878	1.214	-1.07051 down	1.35E-06
LOC41701	111.681	109.563	124.638	98.657	150.498	36.678	18.07	32.181	51.766	62.322	-1.56564 down	4.54E-13
NEFH	0	0.044	0.198	0.195	0.116	0.986	1.689	0.12	0.938	0.404	2.891991 up	6.73E-31
MHCY14	1.109	0.968	2.202	1.215	0.577	0.435	0.303	0.214	1.578	0.396	-1.05173 down	4.38E-06
LOC12110	1.131	0.841	0.951	0.317	0.172	0.189	0.013	0.012	0.283	0.115	-2.46939 down	1.12E-21
MHCY9	1.902	1.998	3.085	1.291	0.376	0.455	0.357	0.051	2.187	0.658	-1.22128 down	4.64E-08
LENG9L7	4.913	5.016	7.793	9.996	2.767	0.247	0.284	0.249	0.604	0.196	-4.26578 down	7.27E-64
OZFL	0.651	0.807	1.588	0.602	1.435	0.43	0.375	0.38	0.643	0.592	-1.06911 down	1.52E-05
YLEC17	0.083	0.109	0.066	0.341	0	1.127	0.509	0	1.264	0.494	2.49249 up	1.36E-08
LOC12110	1.892	1.345	2.562	4.688	0.933	6.679	5.943	0.129	16.773	5.865	1.631311 up	5.14E-14
MHCY12	0.498	0.523	0.722	0.468	0.858	0.966	2.11	3.107	1.897	1.993	1.71302 up	7.64E-16
LOC12110	0.996	0.879	4.17	0.918	0.279	0.12	0.062	0.058	0.153	0.075	-3.93747 down	4.76E-44
LOC12110	0.866	0.54	1.248	0.39	0.508	0.093	0	0	0.175	0.02	-3.60169 down	4.10E-29
LOC12110	1.782	1.403	1.563	0.448	0.336	0	0	0	0.123	0.036	-5.07734 down	8.01E-37
MHCY32	0.15	0.147	0.268	0.446	8.325	0.615	7.123	9.289	1.046	8.113	1.487422 up	6.18E-15
BG8	13.328	12.282	19.78	22.633	26.128	8.824	3.601	9.34	8.81	9.464	-1.23347 down	2.04E-08
LOC12110	2.086	2.181	22.64	26.21	25.371	4.482	2.729	2.945	6.535	3.827	-1.93532 down	1.74E-18
BTN3A3L2	1.23	0.66	7.03	5.052	5.355	0.39	1.157	0.261	1.874	0.439	-2.22818 down	4.92E-21
IL4I1	0.662	0.6	1.835	0.561	0.701	1.909	2.14	1.257	0.795	2.646	1.003962 up	5.57E-07
TRIM7.1	0.057	0.037	2.341	0.117	0.059	0.016	0.029	0.055	0.073	0.085	-3.31423 down	6.60E-24
TRIM39.2	0.25	0.286	0.629	0.256	0.171	1.342	0.425	0.482	0.951	0.392	1.17143 up	1.24E-06
TRIM27.1	0.821	0.682	1.079	0.278	0.455	0.398	0.213	0.239	0.287	0.284	-1.21921 down	1.12E-06
DMB2	1.748	1.707	3.574	4.117	2.439	8.066	5.178	3.955	7.202	4.581	1.092861 up	1.37E-07
CENPA	0.1	0.263	0.24	1.484	0.309	0.341	0.205	0	0.34	0.299	-1.01267 down	0.009572
CYP21A1	0.111	0.145	0.044	1.045	0.596	0.219	0	0.214	0.047	0.11	-1.70955 down	1.38E-07
CD1C	0.026	0.034	0.062	0.191	0.133	1.081	0.29	0.149	0.306	0.128	2.118918 up	7.85E-11
LOC10704	0.733	0.625	0.711	0.838	1.08	0.217	0.065	0.369	0.324	0.411	-1.52099 down	1.18E-07
LOC12110	0.633	0.666	0.864	0.595	0.186	0.948	1.55	1.004	3.83	2.064	1.672588 up	2.21E-13
FCN2	1.003	1.114	0.965	0.611	1.238	0.564	0.281	0.265	0.513	0.494	-1.21792 down	5.64E-08
MAN1B1	82.815	78.378	81.445	67.277	71.142	40.386	41.208	30.472	30.277	48.01	-1.00131 down	3.45E-06
NSMF	0.468	0.31	1.023	0.218	1.076	7.394	5.613	5.838	5.414	4	3.188626 up	7.39E-49

GSL	1.128	1.641	1.741	1.002	1.773	3.491	2.848	6.753	2.494	2.328	1.297499	up	1.45E-10
TNFSF8	0.152	0.298	1.059	0.343	0.312	1.248	0.622	0.587	1.801	0.414	1.108553	up	1.25E-05
PAPPA	0.604	0.53	1.522	0.694	1.565	0.449	0.285	0.289	0.657	0.379	-1.25322	down	3.01E-08
LOC10705	75.951	77.659	59.169	58.121	65.695	32.309	13.742	30.682	21.326	36.257	-1.32535	down	6.14E-10
SLC25A25	43.558	44.396	31.78	31.397	45.136	11.838	6.734	5.877	11.422	16.048	-1.91838	down	2.86E-19
FPGS	41.104	41.489	85.376	36.875	85.68	13.027	6.884	17.993	29.853	23.406	-1.67208	down	5.45E-14
CERCAM	0.422	0.537	0.712	0.848	1.455	0.267	0.369	0.516	0.547	0.283	-1.00181	down	5.37E-05
DNM1	9.543	8.703	6.504	6.356	9.262	1.549	2.175	2.785	3.886	4.179	-1.4695	down	1.23E-11
CRAT	191.389	171.33	169.04	167.34	187.563	66.877	55.02	56.081	57.349	124.466	-1.30121	down	1.45E-09
LAMC3	0.196	0.069	0.073	0.098	0.439	3.125	0.365	1.197	1.566	0.882	3.020349	up	2.11E-38
NTNG2	0.19	0.177	0.662	0.494	1.06	0.125	0.154	0.213	0.034	0.129	-1.9713	down	2.32E-13
AGPAT2	9.837	11.148	19.814	12.831	6.98	34.997	50.091	93.308	20.033	31.145	1.921244	up	9.25E-22
LOC41711	1.094	1.081	2.958	1.086	1.088	3.212	5.793	6.985	1.852	1.853	1.429858	up	1.64E-13
HSPA5	203.11	183.635	232.322	172.83	247.172	942.039	1023.581	307.563	377.802	667.845	1.675379	up	3.54E-16
CDK3	3.108	2.939	1.859	3.759	2.426	0.63	0.458	0.852	0.758	1.265	-1.8288	down	3.11E-16
TEN1	4.687	5.166	5.237	10.568	7.423	0.714	0.806	1.426	2.334	2.676	-2.0552	down	3.24E-20
ACOX1	304.321	272.828	426.332	464.053	441.56	117.08	104.585	96.402	142.968	172.337	-1.59176	down	1.67E-13
FBF1	14.52	14.705	15.185	12.617	14.558	5.194	5.171	5.043	3.947	8.496	-1.36177	down	3.96E-10
MRPL38	52.316	48.259	56.053	35.782	44.87	22.364	16.78	20.845	17.046	33.462	-1.10255	down	4.94E-07
TRIM65	5.692	5.816	6.734	4.437	5.341	2.056	1.629	1.832	1.944	2.347	-1.51395	down	5.88E-12
SMIM5	0.681	0.722	0.237	1.602	0.909	1.587	0.993	2.094	2.015	1.902	1.04847	up	7.62E-07
FASN	110.775	119.381	100.058	62.645	146.049	430.797	372.854	635.789	339.337	152.815	1.84167	up	1.39E-19
NOTUM	0.406	0.444	1.542	0.408	0.375	0.218	0.311	0.303	0.412	0.251	-1.08406	down	2.91E-05
KCNJ16	0.169	0.154	0.382	0.186	0.522	0.586	0.991	1.508	0.239	0.308	1.358891	up	3.07E-11
GAA	12.109	13.411	8.185	7.424	9.144	3.399	5.303	4.396	3.57	5.519	-1.17989	down	5.27E-08
CBX2	0.516	0.535	0.24	0.106	0.309	0.512	0.858	1.35	0.036	0.896	1.095821	up	1.38E-06
SOCS3	3.048	2.755	5.606	4.452	1.599	7.823	12.654	8.3	5.831	17.532	1.578062	up	7.63E-15
TK1	1.116	0.548	2.311	0.917	3.497	11.755	4.511	7.544	3.024	2.602	1.810396	up	5.71E-17
KRABZFP	0.342	0.28	0.204	0.421	0.176	0.484	0.525	0.33	0.724	1.78	1.430113	up	2.86E-05
GRIN2C	1.068	1.271	0.191	0.296	0.839	0.317	0.053	0.154	0.087	0.136	-2.28698	down	3.41E-23
HN1	39.239	38.608	28.761	35.62	42.532	15.285	11.912	12.55	14.215	16.348	-1.39379	down	5.94E-11
SLC16A5	100.899	98.569	67.509	91.142	104.637	19.784	10.459	19.322	17.678	32.512	-2.21373	down	2.09E-24
MIS12	0.314	0.78	0.4	0.774	1.29	5.118	2.376	1.455	0.638	1.245	1.604801	up	2.21E-10
TMEM120	19.329	20.747	33.045	32.708	21.598	11.188	12.035	9.331	10.51	19.661	-1.0225	down	3.40E-06
LOC12110	1.481	1.211	2.801	0.355	3.169	2.865	2.209	1.132	6.897	5.875	1.073189	up	5.87E-07
SRR	5.107	4.768	4.455	3.419	3.831	1.267	1.38	1.967	2.575	3.216	-1.05206	down	4.05E-06
SEBOX	6.22	6.517	4.5	18.195	17.428	17.277	18.035	29.625	25.603	35.505	1.253611	up	5.18E-10
TUSC5	0.404	0.396	0.868	0.473	1.275	0.411	0.052	0.088	0.29	0.09	-1.86984	down	2.07E-14

MED13	24.129	20.91	40.145	20.433	24.737	9.752	5.993	10.354	18.07	11.909	-1.21685	down	3.36E-08
ACACA	39.426	40.517	32.023	29.127	40.439	114.091	77.48	106.663	108.663	57.537	1.355226	up	2.36E-11
EVI2A	0.625	0.324	0.788	0.835	0.294	1.681	1.092	0.754	2.558	1.007	1.305656	up	2.85E-08
FBXO39	0.27	0.238	0.364	0.239	0.256	1.083	0.574	0.391	0.739	0.485	1.256095	up	2.72E-08
ASIP	0.562	0.735	1.598	0.198	0.165	0.182	0	0	0	0.319	-2.68899	down	1.84E-09
TOX2	0.328	0.221	0.563	0.379	0.442	1.405	0.745	0.366	1.441	0.498	1.202475	up	4.47E-08
ACSS2	10.983	11.935	6.329	9.149	7.517	17.773	17.63	14.411	16.519	31.324	1.088738	up	9.75E-08
RBPJL	0.057	0.025	0.045	0.046	0.039	0.043	0.578	0.218	0.064	1.681	3.576628	up	4.31E-30
PI3	9.954	13.416	15.077	52.646	13.145	33.304	95.854	18.569	74.643	555.963	2.900446	up	5.33E-40
TTPAL	2.052	2.341	4.009	2.487	3.081	6.475	7.232	5.284	6.876	4.523	1.120986	up	1.30E-08
LOC1211C	8.342	7.655	10.199	6.202	3.307	2.352	1.523	2.974	4.514	4.326	-1.18612	down	1.26E-07
EPB42	0.861	1.048	2.935	0.467	0.938	0.234	0.088	0.266	0.058	0.359	-2.63042	down	5.79E-23
GATA5	9.765	8.934	4.151	9.352	21.945	2.827	2.723	3.668	5.47	4.351	-1.50768	down	4.23E-12
LOC77197	0.556	0.685	0.352	0.43	0.017	0.482	3.331	3.807	0.028	1.671	2.188848	up	1.18E-22
LOC10085	0.582	0.533	0.463	0.064	0.199	0.702	1.467	1.235	0.328	1.038	1.371098	up	2.88E-08
WFDC2	5.532	6.109	0.358	1.952	0	4.591	12.128	24.181	0.02	16.448	2.039486	up	4.66E-22
WFDC8	75.251	79.538	7.492	29.801	0.154	98.455	189.967	284.111	0.849	208.013	2.023145	up	8.69E-22
SPINT4	2.528	2.408	0.318	0.474	0	2.411	4.265	2.869	0	6.554	1.490057	up	8.69E-11
LOC77199	0.826	0.42	0.122	0.151	0.157	3.741	1.534	5.05	0.518	1.912	2.924237	up	3.18E-32
UBE2U	0.241	0.158	0.346	0.516	0.843	2.843	1.334	1.259	1.145	1.054	1.857014	up	6.59E-13
MMP9	0.016	0.307	0.258	0.786	0.2	0.569	0.597	0.266	2.909	0.675	1.675374	up	1.31E-11
PCK1	3.422	4.317	65.761	5.388	29.476	19.21	0.941	1.68	2.021	28.834	-1.04032	down	2.61E-05
UTS2	1.114	0.865	5.154	1.43	4.218	1.576	0.783	0.269	1.061	0.759	-1.52172	down	2.11E-08
PER3	5.702	5.382	9.939	8.716	14.131	6.621	2.988	1.702	4.982	2.424	-1.22866	down	7.63E-09
ESPN	0.409	0.47	0.315	0.23	0.162	0.244	0.551	2.101	0.073	0.549	1.146871	up	9.04E-09
AJAP1	0	0	0.02	0.011	0.007	0.189	0.316	0.441	0	1.008	5.509637	up	2.57E-63
KBP	3.424	3.238	8.425	4.669	3.62	2.396	2.344	2.228	2.37	2.115	-1.02898	down	6.31E-06
MIB2	37.939	37.536	44.728	17.633	58.931	22.934	15.682	13.569	15.976	12.737	-1.28226	down	3.61E-09
LOC4194C	8.82	8.759	5.918	5.218	47.946	1.745	2.549	1.066	2.165	3.827	-2.75501	down	2.57E-34
LOC77106	34.474	35.519	33.965	35.915	49.009	12.033	10.524	10.344	22.617	17.891	-1.3634	down	1.90E-10
SLC25A33	27.624	24.697	21.936	16.748	18.365	6.412	5.48	8.249	6.738	16.14	-1.34607	down	7.24E-10
RNF186	11.188	11.918	0.265	5.554	0.043	3.038	18.444	11.765	0.176	25.988	1.036142	up	8.10E-07
CELA2A	0.203	0.266	0.485	0.467	0.334	1.012	1.039	0.51	0.413	0.645	1.042008	up	7.73E-05
GUCA2B	0.771	0.466	0.52	1.755	0.366	0.84	4.035	2.749	2.461	0.353	1.427293	up	3.14E-10
WNT4	3.383	3.229	3.599	5.356	3.76	1.382	1.901	3.4	0.986	1.953	-1.00583	down	1.89E-05
LOC11253	13.755	12.357	22.05	16.679	10.052	5.899	5.607	6.328	7.793	7.68	-1.16889	down	1.08E-07
DPYSL2	1.602	1.773	1.878	1.072	1.27	3.735	15.529	9.05	2.403	3.58	2.174221	up	8.16E-26
LOC10174	25.547	25.293	7.821	17.495	25.19	7.457	4.24	13.702	8.075	9.572	-1.23524	down	1.57E-08

ANK1	1.223	1.303	2.635	0.715	1.221	0.569	0.207	0.532	0.781	0.711	-1.34022	down	3.55E-09
ADD2	1.232	1.137	1.521	0.989	0.687	0.724	0.234	0.387	0.744	0.653	-1.02008	down	6.30E-06
SLC20A1	16.074	16.555	17.413	10.516	25.991	6.252	6.266	6.704	8.278	6.289	-1.35683	down	6.15E-10
CASP14	1.235	1.087	1.424	1.278	0.897	0.096	0.065	0.062	0.108	0.486	-2.84985	down	1.70E-25
RETSAT	181.098	181.873	305.999	306.703	245.919	56.354	69.272	106.836	72.562	125.863	-1.50337	down	9.08E-12
LOC10174	0.118	0.173	0.798	1.21	1.15	0.167	0.03	0.455	0.898	0.088	-1.07194	down	7.87E-05
TRNAU1A	12.629	11.351	6.146	7.127	5.097	2.935	2.496	3.472	4.774	5.26	-1.16094	down	3.79E-08
STMN1	5.07	4.523	8.573	3.977	4.092	21.921	17.245	12.619	8.53	4.702	1.30916	up	9.30E-11
LSM10	1.925	2.961	1.947	3.035	2.03	1.708	0.905	0.854	0.44	1.341	-1.18011	down	1.25E-06
FAM167B	18.468	19.179	15.698	20.949	9.892	3.047	4.491	8.094	8.394	5.534	-1.50978	down	3.92E-12
MYCL	25.236	25.063	17.182	6.684	21.801	4.71	1.501	2.528	1.006	6.096	-2.59848	down	1.12E-31
MFSD2A	110.116	114.057	68.368	54.029	85.642	51.118	36.85	44.544	22.121	50.095	-1.07801	down	4.74E-07
GALE	203.78	179.069	256.203	94.255	87.192	42.774	16.683	15.521	30.321	61.757	-2.29614	down	8.31E-26
HMGCL	1321.124	1321.949	1041.953	914.18	1355.956	266.891	61.369	145.98	249.551	524.148	-2.25459	down	2.42E-25
IL22RA1	1.048	0.851	0.331	0.475	0.408	3.335	3.845	5.391	1.224	4.696	2.56852	up	3.04E-30
ADAMTS1	0.388	0.684	1.047	1.074	0.912	2.198	2.764	0.88	2.429	1.501	1.250253	up	1.68E-09
NCAPD3	0.182	0.129	0.23	0.318	0.234	1.273	0.567	0.315	0.386	0.256	1.351579	up	3.26E-09
SCN3B	0.701	0.619	1.075	0.612	0.851	1.603	1.744	2.098	2.211	1.139	1.187782	up	1.85E-09
GRAMD1E	0.84	0.805	1.354	0.858	1.062	2.386	2.074	1.957	2.558	1.472	1.085876	up	3.97E-08
TMEM136	1.429	1.257	0.602	0.75	1.073	0.449	0.356	0.51	0.468	0.452	-1.19152	down	2.52E-07
THY1	0.058	0.304	0.046	0.238	0.219	1.095	0.099	0.355	0.164	0.096	1.060087	up	0.000215
LOC10175	0.148	0.061	0.842	0.061	0.038	1.093	0.399	0.251	2.012	0.534	1.894429	up	3.56E-15
POU2AF1	0.549	0.302	1.32	0.361	0.259	2.449	1.022	0.964	3.848	1.056	1.740676	up	3.16E-16
ZPR1	128.277	139.38	105.824	94.012	102.903	39.162	39.17	36.233	33.628	56.92	-1.47552	down	5.90E-12
APOA5	226.944	249.026	159.85	201.021	232.501	51.602	62.635	54.692	42.59	78.505	-1.88246	down	2.34E-18
FXYD2	7.766	4.487	6.425	4.393	4.04	1.827	1.238	1.558	2.391	1	-1.75765	down	2.25E-12
MPZL2	0.597	0.53	0.725	0.468	0.643	1.675	1.964	1.387	2.047	1.016	1.447362	up	1.72E-11
LOC10174	1.523	1.158	0.721	0.072	0.888	0.073	0.09	0.034	0.11	0.023	-3.70441	down	2.12E-47
HYOU1	31.471	30.812	41.361	31.712	27.66	121.52	125.526	41.781	63.412	72.051	1.38001	up	1.30E-11
NCAM1	0.22	0.186	0.881	0.494	0.472	1.269	0.456	0.825	2.286	0.583	1.264312	up	4.35E-10
BGLAP	2.805	2.936	1.638	3.227	1.825	0.212	0.383	0.542	0.158	0	-3.25794	down	8.68E-23
PEAR1	0.597	0.886	0.858	0.622	0.745	1.655	1.768	3.069	1.102	2.688	1.470165	up	7.88E-13
CD244	1.468	1.276	3.088	2.971	1.845	4.763	3.115	2.411	7.753	4.78	1.099482	up	1.05E-07
SLAMF1	0.106	0.374	0.607	0.313	0.413	1.343	0.953	0.552	2.08	0.819	1.661711	up	1.95E-12
S100A12	2.745	1.976	3.443	9.983	3.384	4.119	14.113	2.584	13.366	19.946	1.329758	up	7.38E-10
CKS1B	1.373	1.796	3.682	5.166	4.156	15.299	9.609	9.836	5.016	4.3	1.4456	up	1.74E-11
FDPS	41.825	42.11	7.962	5.635	47.361	23.583	126.491	140.223	9.627	23.745	1.159504	up	1.75E-09
SYT11	0.614	0.468	2.816	2.184	2.532	1.079	0.722	0.761	0.915	0.818	-1.00318	down	1.90E-05

CIART	0.214	0.482	2.613	0.762	2.785	0.458	0.365	0.298	0.282	0.613	-1.76335	down	2.44E-11
CA14	0.214	0.361	1.585	0.528	2.013	0.416	0.188	0.325	0.259	0.304	-1.65243	down	1.12E-08
TMOD4	0.723	1.269	1.885	0.999	1.756	0.67	0.404	0.444	0.278	0.685	-1.4167	down	8.62E-08
LOC1211(	1.377	2.274	3.499	15.089	1.684	20.717	8.788	15.131	15.213	17.106	1.685409	up	1.91E-14
THEM4	1.379	1.504	1.63	1.751	3.047	0.189	0.171	2.259	0.142	0.249	-1.62755	down	4.36E-09
LOC1211(	1.707	2.146	2.676	0.723	3.534	0.393	0.409	1.107	0.632	0.529	-1.81117	down	5.84E-13
PFDN2	11.692	13.549	20.304	0.767	14.309	1.127	5.471	0.841	0.632	0.555	-2.81234	down	4.46E-31
TULP1	0.396	0.339	3.696	0.217	0.174	0.203	0.598	0.064	0	0.206	-2.16545	down	4.38E-17
ELF3	1.802	1.886	5.136	1.871	1.286	5.692	15.097	9.486	7.195	2.973	1.754718	up	2.49E-18
PTPRVP	0.289	0.28	0.601	0.352	0.379	0.606	1.029	0.785	0.996	0.419	1.010558	up	4.41E-07
CSRP1	1.668	1.898	3.368	2.702	4.32	29.26	21.538	18.016	25.828	23.04	3.075481	up	9.35E-45
UBE2T	0.274	0.426	0.464	0.084	0.633	1.629	0.421	0.529	0.638	0.714	1.061401	up	2.49E-05
NUAK2	0.135	0.399	0.658	0.145	0.237	0.491	0.762	1.098	1.124	0.954	1.489598	up	6.88E-12
LOC1211(	2.336	2.667	13.532	2.99	5.007	1.482	2.102	2.009	1.175	2.784	-1.47338	down	4.67E-10
SLC26A9	0.066	0.086	0.01	0.003	0	0.183	1.18	2.093	0.022	0.484	4.544442	up	7.79E-72
CD34	0.186	0.228	0.376	0.266	0.089	0.985	1.462	0.252	1.137	0.382	1.876634	up	1.57E-15
CAMK1G	0.091	0.094	0.055	0.136	0.055	1.524	0.563	0.619	0.335	1.033	3.225815	up	2.50E-40
LAMB3	0.296	0.278	0.164	0.519	0.172	3.814	1.746	2.039	0.622	3.277	3.003893	up	2.31E-39
G0S2	17.283	17.63	33.545	71.809	20.964	101.004	71.885	284.476	32.925	75.446	1.810968	up	3.56E-19
SLC16A1	80.737	81.864	85.098	65.917	80.697	24.553	14.706	17.98	20.49	26.952	-1.91329	down	8.57E-19
CHIA-M3:	0.124	0.809	0.066	1.151	1.037	0.676	4.257	3.799	0.14	0.388	1.53733	up	1.65E-12
LOC7687ε	0.737	0.599	0.428	1.424	0.491	2.752	6.011	3.5	0.776	6.599	2.41467	up	4.39E-27
LOC10704	1.069	1.001	1.257	0.426	0.349	0.321	0.215	0.438	0.631	0.377	-1.04749	down	7.39E-06
VWA5A1	3.4	3.36	1.917	2.377	3.428	1.009	0.664	0.838	0.966	1.675	-1.49015	down	6.81E-12
DBF4B	2.769	2.891	0.507	1.102	0.954	0.759	0.422	0.681	0.466	0.58	-1.49804	down	2.98E-11
GH	0.916	0.632	0.729	1.296	0.784	1.613	1.353	1.375	2.326	2.271	1.035769	up	1.57E-05
ACE	0	0.011	0.035	0.244	0.162	0.02	0.134	0.852	0.015	1.023	2.164654	up	1.66E-19
SLC4A1	2.92	2.766	7.813	0.831	2.847	0.549	0.328	0.858	1.114	0.93	-2.18291	down	3.80E-21
COL1A1	0.472	0.573	0.814	0.776	1.076	1.867	1.239	2.396	1.599	1.627	1.232727	up	1.09E-09
LOC10705	8.601	8.62	6.678	6.99	10.082	2.455	2.34	5.014	3.147	6.569	-1.06909	down	2.06E-06
LOC4297ε	1.094	0.858	0.37	0.95	0.371	0.164	0.275	0.229	0.594	0.399	-1.13072	down	5.09E-07
PPP1R1B	2.268	2.589	3.554	0.991	4.04	0.437	0.864	1.095	1.021	1.029	-1.59508	down	4.21E-11
NR1D1	1.125	1.077	2.894	1.733	3.465	0.728	0.514	0.562	0.34	0.777	-1.8155	down	7.91E-14
TOP2A	0.342	0.255	0.321	0.388	0.281	3.632	1.148	0.66	0.73	0.855	2.142684	up	1.41E-21
CCR7	0.262	0.268	0.371	0.206	0.549	1.79	0.803	0.615	1.694	0.756	1.769514	up	3.05E-15
KRT40	0.232	0.182	0.664	0.476	0.286	1.181	1.067	1.074	1.138	0.46	1.416503	up	1.05E-09
KRT24	1.121	0.885	1.169	0.46	1.964	2.953	4.328	4.234	3.86	1.648	1.60338	up	1.28E-14
P3H4	4.043	4.802	2.653	3.765	2.923	1.277	1.065	1.675	1.675	1.67	-1.30408	down	2.97E-09

G6PC	546.432	558.921	287.335	471.538	539.08	89.978	65.461	188.641	338.293	192.746	-1.45746	down	6.57E-12
VAT1	115.805	120.68	33.306	87.503	40.757	33.983	31.596	30.897	34.211	32.3	-1.28817	down	3.63E-10
RND2	2.62	1.917	1.709	1.848	1.823	0.397	0.443	0.797	0.734	1.412	-1.38919	down	3.65E-09
BRCA1	0.071	0.057	0.165	0.166	0.28	1.267	0.402	0.232	0.259	0.211	1.67516	up	1.32E-13
SLC1A6	0.116	0.21	0.171	0.125	0.211	0.142	0.457	0.241	0.393	1.028	1.435126	up	2.38E-10
LOC10085	11.151	12.064	21.638	20.943	25.843	63.854	34.885	28.511	41.923	46.82	1.236906	up	7.06E-10
C19orf71	0.377	0.308	0.038	0.039	1.017	0	0.048	0	0	0.047	-4.15704	down	2.70E-13
CTSG	2.03	1.576	3.425	1.188	4.771	11.61	4.998	3.023	10.196	2.628	1.320706	up	3.01E-10
ACSBG2	14.586	15.643	11.837	8.17	13.21	73.262	161.678	173.031	28.239	46.218	2.926614	up	2.03E-42
LRG1	0.422	0.461	0.359	0.44	0.984	1.467	1.383	1.169	0.62	2.123	1.341136	up	3.65E-09
CREB3L3	159.827	156.871	111.394	114.31	126.707	30.241	19.427	43.168	31.453	78.004	-1.72577	down	1.49E-15
PCSK4	2.348	2.734	1.991	1.566	1.895	0.518	0.437	1.222	0.542	1.225	-1.41618	down	1.17E-09
ADAMTSL	0.983	1.337	0.423	0.162	2.564	0.111	0.04	0.152	0.266	0.253	-2.72664	down	9.13E-24
LOC10085	1.85	1.341	1.179	1.31	0.877	0.032	0.291	0.357	0.386	0.678	-1.90761	down	2.72E-13
COMP	1.53	1.326	1.717	1.658	0.99	0.731	0.549	0.633	0.572	0.729	-1.16658	down	2.59E-07
LOC1211C	0.686	0.658	0.66	1.216	0.474	2.72	2.518	2.296	2.645	2.183	1.741288	up	9.81E-16
ACP5	15.183	16.497	42.327	27.342	39.833	8.749	12.867	8.847	7.404	10.247	-1.55293	down	4.42E-12
LOC76984	32.556	33.04	8.417	20.533	10.931	17.282	1.757	7.379	10.772	12.139	-1.09634	down	4.68E-08
LOC10175	1.051	0.647	0.043	0.034	0.021	0	0	0.152	0	0.027	-3.29102	down	9.30E-31
LOC10704	0.009	0.006	0.5	1.416	0.047	0.016	0.127	0.044	0.023	0.077	-2.76364	down	4.82E-28
LOC10705	0	0.054	2.65	1.966	1.017	0.311	0.141	0.159	0.279	0.109	-2.50318	down	7.20E-19
LOC11253	0.229	0.083	1.827	0.796	0.655	0.144	0.13	0.025	0.173	0.076	-2.70064	down	4.87E-18
LOC10085	1.032	1.377	7.206	2.828	7.798	0.596	3.438	1.779	1.817	2.088	-1.05816	down	2.24E-05
LOC1211C	0.207	0.406	0.823	0.085	1.31	0.351	0.035	0.133	0.117	0.308	-1.57938	down	1.42E-06
CHIR-A2	0.295	0.709	0.51	0.769	0.91	0.836	1.914	0.998	1.584	1.612	1.119635	up	4.64E-06
CHIR-AB1	1.081	1.29	0.253	0.444	0.163	0.108	3.057	1.044	1.561	2.113	1.285448	up	5.02E-08
LOC10704	0.688	0.922	0.109	1.526	0.318	0	4.115	1.162	0.699	1.909	1.14491	up	1.35E-06
LOC11253	0.152	0.376	0.701	0.57	0.122	1.686	0.675	1.078	1.031	0.722	1.432071	up	3.15E-10
LOC1211C	0.478	0.516	1.675	1.258	1.253	0.487	0.294	0.693	0.364	0.617	-1.07569	down	6.10E-05
LOC1211C	0.064	0.063	0.305	1.494	1.179	0	0.489	0.339	0.378	0.253	-1.087	down	0.0002
LOC10704	0.052	0	0.416	0.408	0.536	2.04	1.015	0.756	1.547	0.855	2.133611	up	6.90E-18
LOC1211C	0.033	0.109	1.94	0.247	1.2	0.113	0.171	0.097	0.226	0.165	-2.18532	down	3.52E-11
LOC11253	0.417	0.26	6.651	2.071	3.179	0.854	0.507	0.498	0.605	1.594	-1.63086	down	4.67E-11
LOC1211C	0.429	0.345	1.971	0.678	1.017	0.374	0.405	0.191	0.279	0.524	-1.32193	down	5.02E-06
CHIR-AB-	0.641	0.668	0.557	0.455	0.509	5.744	0.984	1.717	2.913	0.925	2.115829	up	7.32E-19
LOC10705	1.089	0.786	1.705	1.358	0.231	1.829	2.498	3.082	3.117	1.267	1.18919	up	5.91E-08
LOC10704	1.145	0.624	1.317	2.51	0.523	6.125	1.042	1.444	3.771	1.83	1.215071	up	1.51E-07
LOC1211C	2.839	1.928	2.347	2.692	1.346	0.742	0.67	0.738	1.294	1.624	-1.13704	down	6.39E-05

RACGAP1	0.029	0.075	0.275	0.201	0.207	1.042	0.324	0.222	0.292	0.1	1.325567 up	1.47E-07
LOC10704	0.392	0.257	0.769	0.198	0.434	1.025	0.833	1.019	1.354	0.299	1.141964 up	1.02E-07
APOF	37.366	50.766	36.826	63.533	23.793	79.245	93.365	210.24	18.573	91.251	1.214618 up	6.91E-10
LOC1211C	0	0	0	0	0	1.06	0.064	0.482	0.106	0.062	8.474923 up	1.99E-14
LOC1211C	0.594	1.033	0.032	0	0	0.122	0.014	0.026	0.046	0.054	-2.63974 down	3.03E-19
LOC1125З	3.567	3.559	6.025	0.829	1.517	0.49	1.179	1.217	2.257	1.787	-1.16049 down	2.09E-06
LOC1211C	0.327	0.157	0.104	0.125	0.38	1.283	0.245	0.547	1.144	0.238	1.656728 up	2.60E-10
LOC1211C	0.186	0.169	0.628	0.801	0.736	1.51	1.393	0.401	0.218	1.55	1.007693 up	2.14E-06
LOC1211C	0.891	0.858	0.65	1.728	0.254	0.448	0.405	0.693	0.084	0.515	-1.02857 down	5.28E-05
LOC1070Ƽ	2.671	2.885	17.939	1.035	2.537	2.54	2.501	1.873	0.939	2.074	-1.44665 down	5.09E-09
OTX5	3.385	3.379	6.24	1.429	2.499	0.879	0.318	0.73	0.911	1.458	-1.97743 down	5.06E-18
LOC1070Ƽ	5.249	4.591	10.795	2.095	3.419	1.936	0.522	1.158	1.828	2.172	-1.77898 down	2.39E-14
LOC42622	85.864	97.813	72.44	292.66	10.808	418.575	250.192	319.647	17.904	261.465	1.179871 up	2.14E-08
LOC1211C	2.846	2.578	2.049	1.574	2.642	1.116	0.504	0.74	1.205	0.814	-1.41545 down	1.67E-08
LOC1211C	0.063	0.064	0.521	0.104	0.253	10.983	1.295	0.971	1.465	3.704	4.189081 up	3.20E-69
ALPK2	1.282	0.914	3.265	0.609	0.412	0.344	0.354	0.012	0.175	0.862	-1.88855 down	6.51E-16
CNTFR	0.717	0.899	0.809	0.629	1.301	2.653	1.003	2.112	1.396	1.6	1.008084 up	9.01E-07
LOC1008Ƽ	2.842	2.447	3.327	2.85	2.704	12.468	9.426	3.227	15.78	3.481	1.646788 up	9.60E-15
AVD	0.679	0.578	0.703	2.345	0.488	4.384	5.976	3.017	0.805	14.014	2.555241 up	2.91E-26
GZMA	5.311	6.24	9.637	14.31	8.275	3.845	3.4	5.232	4.527	4.73	-1.00992 down	6.59E-06
CENPK	0.269	0.07	0.236	0.111	0.111	1.036	0.358	0.13	0.182	0.133	1.201165 up	4.88E-05
S100Z	2.605	2.791	0.713	2.97	4.582	0.719	0.533	2.389	1.185	0.452	-1.37116 down	2.02E-09
LOC1008Ƽ	0.425	0.43	0.534	0.438	0.616	1.859	1.607	0.768	0.931	0.803	1.286852 up	4.20E-10
GLDC	69.204	66.091	99.52	67.77	72.787	313.447	292.806	296.23	150.796	173.542	1.708518 up	3.08E-17
FBP2	3.111	2.678	4.789	5.166	1.373	1.773	0.513	0.484	1.556	1.098	-1.65709 down	7.08E-14
GADD45G	9.213	10.267	11.004	14.629	6.816	49.684	39.216	80.349	6.346	51.417	2.128049 up	2.74E-24
NFIL3	5.33	4.545	11.533	5.205	5.75	22.122	16.569	12.354	21.025	11.302	1.365081 up	6.64E-12
LOC1211C	0.579	0.824	0.686	3.402	0.581	0.277	0.171	0.546	0.392	0.179	-1.95259 down	5.98E-17
SHB	3.992	3.684	5.439	2.551	2.615	4.686	30.397	4.013	2.651	25.411	1.876927 up	2.97E-20
LPL	1.707	1.894	5.175	0.471	5.173	0.18	0.152	0.154	6.212	0.063	-1.0922 down	9.58E-07
PSD3	7.349	6.967	7.679	4.194	8.275	2.711	2.338	1.71	5.192	2.666	-1.23716 down	7.62E-09
LOC76841	3.926	3.775	0.9	4.596	3.422	0.539	0.304	0.287	0.604	0.472	-2.9105 down	2.19E-27
KIAA1958	30.304	27.952	9.523	15.084	9.131	54.049	34.481	53.703	11.632	42.204	1.091708 up	1.02E-07
SMC2	0.127	0.124	0.223	0.357	0.39	1.988	0.602	0.393	0.322	0.233	1.531012 up	2.75E-11
TUSC1	1.55	2.127	1.489	1.287	1.782	0.382	0.448	0.878	1.17	1.136	-1.03581 down	8.60E-06
CDKN2A	0.782	0.648	0.831	0.964	1.152	5.433	2.641	4.103	2.782	2.354	1.982611 up	3.69E-19
CDKN2B	0.209	0.561	0.183	0.567	0.773	2.913	1.241	2.484	2.267	2.137	2.265204 up	1.63E-22
CCDC112	2.162	1.871	1.46	1.92	1.839	0.47	0.737	0.653	0.85	0.628	-1.4694 down	4.10E-10

LOC10705	14.178	14.894	14.025	12.341	21.783	46.038	39.06	36.862	27.053	25.709	1.177944	up	4.24E-09
LOC1211C	0.075	0.077	0.274	0.159	0.155	0.18	1.124	1.822	1.52	0.067	2.662863	up	1.69E-31
LOC1211C	0.549	0.406	0.475	0.393	1.006	1.433	1.173	0.968	1.092	1.303	1.075857	up	1.27E-06
CHIR-IG1-	1.543	1.607	2.131	4.347	4.334	12.123	2.738	2.554	6.292	5.341	1.056665	up	1.05E-06
LOC1211C	1.098	1.293	3.497	0.782	3.759	1.492	1.048	0.283	0.372	0.944	-1.3322	down	2.91E-06
LOC10705	0.256	0.28	1.429	1.569	1.434	0.065	0.262	0.165	0.289	0.141	-2.42348	down	7.80E-17
PKMYT1	0.051	0.117	0.041	0.126	0.235	1.385	0.443	0.271	0.216	0.126	2.088791	up	4.39E-12
LOC1211C	0.534	1.017	0.426	4.591	1.247	0	0.994	0.61	0	0.193	-2.11757	down	2.47E-14
LOC1211C	0.814	0.875	0.538	0.68	1.176	0.062	0.212	0.306	0.07	0.137	-2.36782	down	2.58E-19
LOC1211C	1.881	1.885	2.629	1.665	1.527	0.804	0.458	0.864	1.292	0.848	-1.16726	down	3.20E-07
LOC10705	0.452	0.371	1.943	1.664	2.801	0.558	0.655	0.234	0.619	0.44	-1.52693	down	9.91E-12
LOC11253	3.871	3.576	1.459	0.728	4.37	0.703	0.453	3.537	0.75	1.436	-1.02503	down	3.86E-05
LOC10704	0.593	0.745	1.764	0.615	0.224	3.975	1.676	2.907	3.091	1.192	1.702856	up	7.99E-16
LOC1211C	0.997	0.47	0.794	1.803	0	0.587	0.082	0	0	0	-2.59385	down	5.97E-17
LOC1211C	1.695	1.763	2.108	1.031	1.527	3.971	2.352	3.7	4.09	4.792	1.218	up	3.58E-09
LOC1211C	1.262	1.789	1.597	1.671	1.944	2.699	2.969	4.603	4.488	4.353	1.209246	up	2.33E-09
MHCY13	3.428	2.686	3.329	0.991	1.896	0.384	0.308	0.364	0.51	0.971	-2.27872	down	6.53E-19
LOC11253	2.993	2.829	7.512	3.722	2.373	0.425	0.71	0.736	1.137	2.528	-1.81036	down	2.38E-15
LOC1211C	15.418	15.792	19.848	25.319	21.906	8.149	8.034	7.607	10.941	13.39	-1.0302	down	2.22E-06

qvalue	function
2.70E-07	LOC121113333; C-type lectin domain family 2 member B-like isoform X4
1.23E-14	LOC121106515; C-type lectin domain family 2 member D-like
3.80E-10	LOC776463; C-type lectin domain family 2 member B-like
6.01E-06	LOC121109235; thymidine phosphorylase isoform X4
6.80E-07	CD69L; C-type lectin domain family 2 member B
8.00E-21	ELAPOR2; endosome/lysosome-associated apoptosis and autophagy regulator family member 2 isoform X1
1.71E-10	ALG12; dol-P-Man:Man(7)GlcNAc(2)-PP-Dol alpha-1,6-mannosyltransferase isoform X1
5.36E-17	CRELD2; protein disulfide isomerase CRELD2 precursor
0.034352	ARL8BL; ADP-ribosylation factor-like 8B-like
1.26E-06	IQUB; IQ and ubiquitin-like domain-containing protein isoform X4
1.29E-09	CPED1; cadherin-like and PC-esterase domain-containing protein 1 isoform X4
0.000125	CAV1; caveolin-1
2.05E-05	AMIGO2; amphoterin-induced protein 2 precursor
9.50E-09	IFNG; interferon gamma precursor
1.94E-12	TSPAN8; tetraspanin-8
3.50E-26	LGR5; leucine-rich repeat-containing G-protein coupled receptor 5 isoform X3
5.39E-17	GLIPR1; GLI pathogenesis related 1 precursor
0.000186	LOC121109191; uncharacterized protein LOC121109191 isoform X1
3.63E-06	MRPL42; 39S ribosomal protein L42, mitochondrial
7.66E-06	LOC427882; histone H1
4.53E-05	HIST1H4D; histone H4
2.11E-05	HIST1H2B8; histone H2B 8
0.030783	HIST1H46L6; histone H4
9.39E-10	HIST1H2A4L3; histone H2A-III
7.27E-09	EMP1; epithelial membrane protein 1
2.37E-19	GUCY2C; heat-stable enterotoxin receptor isoform X3
2.18E-18	MEI1; meiosis inhibitor protein 1 isoform X12
1.44E-06	CHADL; chondroadherin-like protein precursor
1.00E-05	MGAT3; beta-1,4-mannosyl-glycoprotein 4-beta-N-acetylglucosaminyltransferase isoform X2
3.38E-34	NPTXR; neuronal pentraxin receptor
3.05E-15	FAM20CL; extracellular serine/threonine protein kinase FAM20C isoform X1
9.16E-10	SUN2; SUN domain-containing protein 2
2.15E-20	CBY1; protein chibby homolog 1
1.13E-11	KDELR3; ER lumen protein-retaining receptor 3
6.74E-06	CARD10; caspase recruitment domain-containing protein 10 isoform X7
9.62E-06	CSF2RB; cytokine receptor common subunit beta isoform X1

2.13E-06 CRCBL; cytokine receptor common subunit beta isoform X2
2.35E-11 MCM5; DNA replication licensing factor MCM5
5.76E-08 C12orf75; overexpressed in colon carcinoma 1 protein homolog
1.03E-16 HSP90B1; endoplasmin precursor
6.87E-06 NT5DC3; 5'-nucleotidase domain-containing protein 3
1.10E-09 ASCL1; achaete-scute homolog 1
3.72E-07 IGF1; insulin-like growth factor I preproprotein
7.51E-25 CGTL; cystine/glutamate transporter isoform X4
1.01E-09 EPS8; epidermal growth factor receptor kinase substrate 8 isoform X1
8.09E-09 SLCO1B1; solute carrier organic anion transporter family member 1C1 isoform X1
6.08E-05 LDHB; L-lactate dehydrogenase B chain isoform X1
1.23E-06 SSPN; sarcospan isoform X1
6.67E-07 BHLHE41; class E basic helix-loop-helix protein 41
2.35E-08 ENDOUL; poly(U)-specific endoribonuclease-A isoform X1
3.83E-09 PNPLA3; patatin-like phospholipase domain-containing protein 2 isoform X3
1.24E-08 LOC101751545; cyclin-dependent kinase inhibitor 1 isoform X3
2.81E-10 A2ML3; alpha-2-macroglobulin-like isoform X6
1.50E-18 CASR; extracellular calcium-sensing receptor
9.21E-08 CNP1; C-type natriuretic peptide 1 precursor
9.57E-27 GPR162; probable G-protein coupled receptor 162
6.09E-10 CDCA3; cell division cycle-associated protein 3 isoform 2
1.03E-05 RBP5; retinol-binding protein 5
4.01E-14 EPHA1; ephrin type-A receptor 1 precursor
1.08E-05 GSTK1; glutathione S-transferase kappa 1 isoform X1
5.28E-06 LOC100859872; uncharacterized protein LOC100859872 isoform X1
1.29E-06 HAO2; hydroxyacid oxidase 2
2.24E-07 SIDT1; SID1 transmembrane family member 1 isoform X2
6.82E-10 APOV1; apovitellenin-1 precursor
8.46E-37 SLC19A2; thiamine transporter 1
5.35E-12 DPT; dermatopontin
3.93E-05 TBX19; T-box transcription factor TBX19
1.18E-20 CD200L; uncharacterized protein LOC418424 precursor
1.01E-37 LOC121110262; uncharacterized protein LOC121110262 isoform X1
3.93E-05 CREG1; protein CREG1 precursor
1.82E-06 MAEL; protein maelstrom homolog isoform X3
4.17E-06 ROBO1; roundabout homolog 1 isoform X7
6.27E-16 MAP3K7CL; MAP3K7 C-terminal-like protein isoform X4
6.35E-13 GART-B; trifunctional purine biosynthetic protein adenosine-3 isoform X2

2.64E-10	KCNJ15; ATP-sensitive inward rectifier potassium channel 15 isoform X2
8.31E-18	PCP4; calmodulin regulator protein PCP4
1.22E-07	ABCG1; ATP-binding cassette sub-family G member 1 isoform X5
3.48E-06	AGPAT3; 1-acyl-sn-glycerol-3-phosphate acyltransferase gamma
1.22E-05	LOC107055472; translation initiation factor IF-2 isoform X1
7.36E-09	MAOB; amine oxidase [flavin-containing] B
1.37E-05	NR0B1; nuclear receptor subfamily 0 group B member 1 isoform X2
2.94E-06	SLC51AL; organic solute transporter subunit alpha
3.42E-17	MAP3K15; mitogen-activated protein kinase kinase kinase 15
3.15E-15	PDHA2; pyruvate dehydrogenase E1 component subunit alpha, somatic form, mitochondrial precursor
7.41E-40	ADGRG2; adhesion G-protein coupled receptor G2 isoform X1
2.04E-25	GRPR; gastrin-releasing peptide receptor
4.49E-18	LOC418658; arylsulfatase D isoform X2
2.45E-11	CRLF2; cytokine receptor-like factor 2 isoform X2
3.96E-13	IL1R2; interleukin-1 receptor type 2 isoform X1
1.71E-09	IL1R1; interleukin-1 receptor type 1 precursor
1.95E-19	IL1RL1; interleukin-1 receptor-like 1 isoform LV precursor
6.58E-05	RAB20; ras-related protein Rab-20
9.76E-29	LOC121107687; SPARC
4.13E-07	DOCK9; dedicator of cytokinesis protein 9 isoform X17
3.22E-08	HS6ST3; heparan-sulfate 6-O-sulfotransferase 3
1.65E-08	DCT; L-dopachrome tautomerase precursor
8.53E-09	ACOD1; cis-aconitate decarboxylase
2.17E-08	LOC112532134; uncharacterized protein LOC112532134 isoform X1
1.49E-06	CKAP2; cytoskeleton-associated protein 2
1.11E-06	AMER2; APC membrane recruitment protein 2
3.73E-64	SGCG; gamma-sarcoglycan isoform X4
2.37E-29	GJB2; gap junction beta-2 protein
1.35E-05	ACAT1; acetyl-CoA acetyltransferase, mitochondrial
#######	MMP1; interstitial collagenase
1.29E-13	MMP7; matrilysin precursor
1.37E-07	TAF1D; TATA box-binding protein-associated factor RNA polymerase I subunit D
6.82E-08	AQP11; aquaporin-11
5.80E-15	CAPN5; calpain-5
1.77E-16	TSKU; tsukushin precursor
1.03E-08	WNT11; protein Wnt-11 isoform X4
4.73E-07	UVRAG; UV radiation resistance-associated gene protein isoform X11
0.000154	ART1L1; ADP-ribosyltransferase 1 like 1 precursor

2.62E-10 LRTOMT; leucine rich transmembrane and O-methyltransferase domain containing isoform X1
2.37E-63 ADAM15; disintegrin and metalloproteinase domain-containing protein 9 isoform X3
8.76E-07 SLCO2B1; solute carrier organic anion transporter family member 2B1 isoform X2
3.14E-06 P2RY6; P2Y purinoceptor 3
2.89E-06 DGAT2; diacylglycerol O-acyltransferase 2 isoform X1
5.64E-46 HBE; hemoglobin subunit epsilon
1.84E-18 HBBA; hemoglobin subunit beta
4.21E-21 HBE1; hemoglobin subunit epsilon 1
4.50E-09 TAF10; transcription initiation factor TFIID subunit 10
2.48E-15 TPP1; tripeptidyl-peptidase 1
1.77E-10 GIMAP1-GIMAP5; GTPase IMAP family member 1
8.24E-18 ACKR2; atypical chemokine receptor 2
1.04E-21 CYP8B1; cytochrome P450, family 8, subfamily B
1.45E-05 ALS2L; ALS2 C-terminal-like protein isoform X6
1.23E-07 CHDSD; D-threo-3-hydroxyaspartate dehydratase isoform X3
0.000586 CATH2; cathelicidin-2 precursor
3.60E-13 CATH1; cathelicidin-1 precursor
1.59E-08 GIMAP6; GTPase IMAP family member 5
2.23E-07 VILL; villin-like protein isoform X2
1.05E-05 DLEC1; deleted in lung and esophageal cancer protein 1 isoform X3
7.08E-14 ACAA1; 3-ketoacyl-CoA thiolase, peroxisomal
4.16E-10 APBB1IP; amyloid beta A4 precursor protein-binding family B member 1-interacting protein
1.03E-42 NELL3; uncharacterized protein NELL3
1.40E-07 THNSL1; threonine synthase-like 1 isoform X1
7.14E-08 RSU1; ras suppressor protein 1
2.49E-19 ACBD7; acyl-CoA-binding domain-containing protein 7 isoform X2
2.91E-18 OLAH; S-acyl fatty acid synthase thioesterase, medium chain isoform X1
5.05E-07 CROT; peroxisomal carnitine O-octanoyltransferase
1.98E-28 ABCB1LA; phosphatidylcholine translocator ABCB4 isoform X5
6.84E-11 CYP51A1; leucine-rich repeat and death domain-containing protein 1 isoform X2
2.64E-10 CDK6; cyclin-dependent kinase 6
2.41E-08 PDK4; pyruvate dehydrogenase kinase, isozyme 4
2.08E-06 ICA1; islet cell autoantigen 1 isoform X1
5.39E-21 THSD7A; thrombospondin type-1 domain-containing protein 7A isoform X6
3.39E-29 LOC395159; Schwann cell-specific EGF-like repeat autocrine factor precursor
1.89E-11 VWDE; von Willebrand factor D and EGF domain-containing protein
1.34E-12 HDAC9; histone deacetylase 9 isoform X11
2.71E-08 WIPF3; WAS/WASL-interacting protein family member 3 isoform X2

8.21E-06 NR1D2; nuclear receptor subfamily 1 group D member 2 isoform X2
1.10E-07 EOMES; eomesodermin homolog isoform X1
0.000877 LOC101750511; BCL-6 corepressor-like
2.95E-08 ACAD11; acyl-CoA dehydrogenase family member 11
1.80E-06 NPHP3; nephrocystin-3 isoform X2
0.001742 LZTFL1; leucine zipper transcription factor-like protein 1 isoform X1
2.66E-14 KIF15; kinesin-like protein KIF15
1.02E-09 BMPER; BMP-binding endothelial regulator protein isoform X1
1.93E-11 PPP1R17; protein phosphatase 1 regulatory subunit 17
8.57E-11 AMPH; amphiphysin isoform X1
1.98E-12 INHBA; inhibin beta A chain isoform X1
8.64E-08 IGFBP1; insulin-like growth factor-binding protein 1 precursor
1.01E-10 LOC121109603; uncharacterized protein LOC121109603
1.24E-18 SALL3; sal-like protein 3 isoform X1
8.57E-07 KIAA0319; dyslexia-associated protein KIAA0319 homolog isoform X4
9.17E-12 DCDC2; doublecortin domain-containing protein 2 isoform X4
3.58E-09 ELOVL2; elongation of very long chain fatty acids protein 2
6.81E-10 SYCP2L; synaptonemal complex protein 2-like isoform X3
4.70E-07 GCNT2; N-acetyllactosaminide beta-1,6-N-acetylglucosaminyl-transferase isoform X2
5.92E-57 LOC107051671; UPF0500 protein C1orf216 homolog
7.29E-06 PPP1R3G; protein phosphatase 1 regulatory subunit 3G
1.86E-21 ECI2; enoyl-CoA delta isomerase 2 isoform X3
5.91E-05 SERPINB1; leukocyte elastase inhibitor isoform X1
1.22E-05 SERPINB10B; heterochromatin-associated protein MENT isoform X1
2.12E-12 CTNND2; catenin delta-2 isoform X5
1.42E-18 CMBL; carboxymethylenebutenolidase homolog
4.00E-12 SRD5A1; 3-oxo-5-alpha-steroid 4-dehydrogenase 1 isoform X3
2.33E-06 UBE2QL1; ubiquitin-conjugating enzyme E2Q-like protein 1
1.05E-16 LOC769743; SUN domain-containing protein 3-like
2.51E-06 LOC121109782; SUN domain-containing protein 3-like isoform X1
1.11E-07 MOCOS; molybdenum cofactor sulfurase isoform X1
4.11E-05 PSMG2; proteasome assembly chaperone 2
1.79E-06 TGIF1; homeobox protein AKR
2.98E-19 COLEC12; collectin-12
2.10E-31 ABHD3; phospholipase ABHD3 isoform X1
1.76E-08 MCM4; DNA replication licensing factor MCM4
5.40E-23 C8orf22; pancreatic progenitor cell differentiation and proliferation factor-like protein
3.33E-20 CYP7A1; cholesterol 7-alpha-monooxygenase

7.88E-10 GDAP1; ganglioside-induced differentiation-associated protein 1 isoform X1
8.09E-12 FABP4; fatty acid-binding protein, adipocyte
1.03E-10 ATP6V0D2; V-type proton ATPase subunit d 2
3.81E-06 WWP1; NEDD4-like E3 ubiquitin-protein ligase WWP1 isoform X3
3.16E-09 PTDSS1; phosphatidylserine synthase 1 isoform X1
6.92E-10 ANXA13; annexin A13
1.33E-13 SQLE; squalene monooxygenase
7.66E-08 LOC121113091; uncharacterized protein LOC121113091
4.51E-06 CFAP36; cilia- and flagella-associated protein 36 isoform X1
1.53E-05 FANCL; E3 ubiquitin-protein ligase FANCL isoform X1
3.13E-14 BUB1; mitotic checkpoint serine/threonine-protein kinase BUB1
2.19E-09 LOC121110173; carbohydrate sulfotransferase 9-like isoform X1
1.90E-12 CAPN13; calpain-13 isoform X2
8.95E-06 PPP1R21; protein phosphatase 1 regulatory subunit 21 isoform X2
3.92E-08 UGP2; UTP--glucose-1-phosphate uridylyltransferase isoform X1
8.60E-10 ITPKC; inositol-trisphosphate 3-kinase B isoform X2
0.000219 GINS1; DNA replication complex GINS protein PSF1
2.37E-09 TLR5; toll-like receptor 5 precursor
3.80E-06 LOC421419; uncharacterized protein LOC421419 isoform 2
1.68E-08 MAD2L1BP; MAD2L1-binding protein
1.12E-15 PLD5; inactive phospholipase D5 isoform X5
1.88E-06 LOC100857421; ankyrin repeat domain-containing protein 26-like isoform X1
1.36E-19 DACT2; dapper homolog 2
1.63E-09 MPC1L; mitochondrial pyruvate carrier 1
3.24E-06 TFB1M; dimethyladenosine transferase 1, mitochondrial isoform X2
7.44E-10 SGK1; serine/threonine-protein kinase Sgk1
2.03E-19 SLC2A12; solute carrier family 2, facilitated glucose transporter member 12
1.30E-16 LOC107056139; pantetheinase-like precursor
1.31E-09 TMEM200A; transmembrane protein 200A
8.74E-07 GJA1; gap junction alpha-1 protein
8.53E-12 MCM9; DNA helicase MCM9 isoform X1
2.81E-05 LOC107053100; translation initiation factor IF-2-like isoform X1
3.24E-06 LOC121106461; transcription regulator protein BACH2
5.63E-16 ME1; NADP-dependent malic enzyme
3.47E-08 RWDD2A; RWD domain-containing protein 2A
4.55E-09 TTK; dual specificity protein kinase TTK
4.55E-16 GFRAL; GDNF family receptor alpha-like isoform X3
1.33E-17 GSTA3; glutathione S-transferase

1.61E-05 LOC100859645; glutathione S-transferase isoform X1
3.04E-09 FAM110C; protein FAM110C
4.64E-07 PXDN; peroxidasin homolog isoform X2
1.03E-07 KLF11; Krueppel-like factor 11 isoform X2
2.32E-16 RRM2; ribonucleoside-diphosphate reductase subunit M2
1.61E-08 PDIA6; protein disulfide-isomerase A6
9.73E-11 VSNL1; visinin-like protein 1 isoform X2
4.05E-25 RHOB; rho-related GTP-binding protein RhoB
1.63E-09 HADHA; trifunctional enzyme subunit alpha, mitochondrial
7.68E-18 HADHB; trifunctional enzyme subunit beta, mitochondrial
5.86E-61 LOC121110416; protein aveugle-like isoform X1
3.42E-09 GVINP1; interferon-induced very large GTPase 1
3.50E-19 LOC770271; uncharacterized protein LOC770271
8.64E-06 NUGGC; nuclear GTPase SLIP-GC
3.73E-17 PBK; lymphokine-activated killer T-cell-originated protein kinase isoform X1
6.46E-11 LOC426385; serine/threonine-protein kinase 35-like isoform X1
1.04E-25 SOX7; transcription factor SOX-7
3.24E-16 BLK; tyrosine-protein kinase Blk isoform X1
1.71E-09 FDFT1; squalene synthase
1.93E-25 MCM3; DNA replication licensing factor MCM3 isoform X1
1.56E-11 CRISP2; serotriflin isoform X1
4.90E-16 RHAG; ammonium transporter Rh type A
2.28E-18 CYP2AC1; cytochrome P450 2AC1
5.23E-12 RCAN2; calcipressin-2 isoform X2
6.61E-12 ADGRF5; adhesion G protein-coupled receptor F5
1.20E-05 OPHN1; oligophrenin-1 isoform X3
1.17E-11 ARR3; arrestin-C
9.38E-13 KIF4B; chromosome-associated kinesin KIF4
1.15E-06 CCNB3; G2/mitotic-specific cyclin-B3 isoform X2
1.78E-05 LOC428702; gap junction alpha-3 protein-like
3.40E-42 SLC6A14; sodium- and chloride-dependent neutral and basic amino acid transporter B(0+) isoform X2
5.95E-08 FAM162BL; protein FAM162B isoform X2
1.18E-10 GPC3; glypican-3
2.65E-07 NSDHL; sterol-4-alpha-carboxylate 3-dehydrogenase, decarboxylating
3.04E-06 TRPC5; short transient receptor potential channel 5
7.38E-11 LOC121110638; uncharacterized protein LOC121110638
2.83E-12 SH2D1A; SH2 domain-containing protein 1A isoform X1
9.23E-09 RXFP1; relaxin receptor 1 isoform X1

3.74E-10 APELA; apelin receptor early endogenous ligand precursor
3.62E-06 MSMO1; methylsterol monooxygenase 1
1.14E-12 TBC1D9; TBC1 domain family member 9 isoform X3
8.55E-07 SLC20A2; sodium-dependent phosphate transporter 2
2.66E-06 CYP2U1; cytochrome P450 2U1
4.69E-16 ARHGEF38; rho guanine nucleotide exchange factor 38
2.32E-15 LRP2BP; LRP2-binding protein isoform X1
5.80E-18 ACSL1; long-chain-fatty-acid--CoA ligase 1
2.88E-19 DCTD; deoxycytidylate deaminase isoform X2
1.41E-10 GPAT3; glycerol-3-phosphate acyltransferase 3 isoform X1
7.47E-07 DUSP4; dual specificity protein phosphatase 4
8.55E-76 LOC430303; prolow-density lipoprotein receptor-related protein 1 isoform X3
3.58E-06 SHROOM3; protein Shroom3 isoform X7
3.92E-10 NAAA; N-acylethanolamine-hydrolyzing acid amidase isoform X1
2.18E-22 SULT1B; sulfotransferase family, cytosolic, 1B isoform X1
0.000469 FABP2; fatty acid-binding protein, intestinal
5.33E-08 ENPEP; glutamyl aminopeptidase
7.75E-28 CCDC110; coiled-coil domain-containing protein 110
8.29E-35 FAT1; protocadherin Fat 1 isoform X8
3.17E-12 PDGFRL; platelet-derived growth factor receptor-like protein isoform X5
9.33E-12 BEND4; BEN domain-containing protein 4 isoform X1
9.23E-48 CCKAR; cholecystokinin receptor type A
1.32E-22 RBPJ; recombining binding protein suppressor of hairless isoform X1
3.22E-07 PPARGC1A; peroxisome proliferator-activated receptor gamma coactivator 1-alpha isoform X1
1.96E-65 SPON2; spondin-2 isoform X2
1.29E-16 SMYD1; histone-lysine N-methyltransferase SMYD1
8.82E-36 ADRA1D; alpha-1D adrenergic receptor
7.47E-08 NAT8; putative N-acetyltransferase 8B
7.42E-11 LOC430422; protein O-GlcNAcase
9.26E-15 LOC107056412; membrane-spanning 4-domains subfamily A member 12-like isoform X3
6.81E-05 CD6; T-cell differentiation antigen CD6 isoform X1
7.04E-10 TCIRG1; V-type proton ATPase 116 kDa subunit a3
9.50E-10 SPTB; spectrin beta chain, erythrocytic isoform X1
1.04E-33 LUZP2; leucine zipper protein 2 isoform X1
7.50E-10 DHCR7; 7-dehydrocholesterol reductase
7.16E-11 E2F8; transcription factor E2F8 isoform X2
8.93E-06 RCN1; reticulocalbin-1 isoform X1
2.75E-12 PRRG4; transmembrane gamma-carboxyglutamic acid protein 4 isoform X1

2.47E-14 OVCH1; ovochymase-2 isoform X1
1.70E-13 MICAL2; F-actin-monooxygenase MICAL2 isoform X20
4.08E-07 CALCA; calcitonin isoform 1 preproprotein
3.76E-09 USH1C; harmonin isoform X1
6.81E-21 SAA; serum amyloid A isoform X2
4.30E-14 TMEM86A; lysoplasmalogenase-like protein TMEM86A
7.50E-07 KCNQ1; potassium voltage-gated channel subfamily KQT member 1
5.96E-51 TRPM5; transient receptor potential cation channel subfamily M member 5 isoform X1
1.06E-07 SYT8; synaptotagmin-8
2.97E-05 CTSD; cathepsin D precursor
3.11E-13 CHKA; choline kinase alpha isoform X2
6.53E-23 CPT1A; carnitine O-palmitoyltransferase 1, liver isoform isoform X2
8.28E-06 SMTNL1; smoothelin-like protein 1
2.20E-10 PGR2/3; P2X purinoceptor 3 precursor
2.80E-23 SMIM38; small integral membrane protein 38
1.78E-49 FGF19; fibroblast growth factor 19 precursor
5.07E-06 HSD17B12; very-long-chain 3-oxoacyl-CoA reductase
1.52E-10 ACCS; 1-aminocyclopropane-1-carboxylate synthase-like protein 1 isoform X3
3.75E-10 MDK; midkine isoform X1
2.78E-20 CHAC1; glutathione-specific gamma-glutamylcyclotransferase 1
1.36E-06 NUSAP1; nucleolar and spindle-associated protein 1
4.87E-72 SPTBN5; spectrin beta chain, non-erythrocytic 5
2.85E-10 EHD4; EH domain-containing protein 4
4.81E-05 GALNT16; polypeptide N-acetylgalactosaminyltransferase 16 isoform X2
1.46E-10 RDH11; retinol dehydrogenase 12
2.96E-12 LGALSL2; galectin-related protein A isoform X3
5.38E-07 DTD2; D-aminoacyl-tRNA deacylase 2 isoform X3
8.02E-16 TTC6; tetratricopeptide repeat protein 6 isoform X3
1.36E-18 ACSS1B; acetyl-coenzyme A synthetase 2-like, mitochondrial isoform X2
2.51E-07 FOS; proto-oncogene c-Fos
7.20E-24 FLVCR2; feline leukemia virus subgroup C receptor-related protein 2 isoform X2
1.63E-24 DIO2; type II iodothyronine deiodinase isoform a
1.90E-05 GALC; galactocerebrosidase isoform X2
3.64E-30 SPIA3; alpha-1-antiproteinase isoform X1
1.08E-60 BDKRB1; B1 bradykinin receptor
3.62E-69 LOC121110856; uncharacterized protein LOC121110856
3.80E-06 DEGS2; sphingolipid delta(4)-desaturase/C4-monooxygenase DES2
3.83E-11 SLC25A47; solute carrier family 25 member 47 isoform X5

3.79E-05	DIO3; thyroxine 5-deiodinase
1.64E-30	ANKRD9; ankyrin repeat domain-containing protein 9
7.18E-07	APOPT1; cytochrome c oxidase assembly factor 8 isoform X1
1.05E-06	ASPG; 60 kDa lysophospholipase isoform X2
7.48E-07	PLD4; 5'-3' exonuclease PLD4 isoform X2
4.28E-06	DHRS7; dehydrogenase/reductase SDR family member 7
4.37E-05	C14orf105; uncharacterized protein CCDC198 isoform X2
1.91E-09	DLGAP5; disks large-associated protein 5
1.85E-05	GCH1; GTP cyclohydrolase 1
0.000177	CDKN3; cyclin-dependent kinase inhibitor 3 isoform X1
3.26E-06	LOC121106444; protein MANBAL-like
9.86E-35	GRID1; glutamate receptor ionotropic, delta-1 isoform X3
4.03E-22	SNCG; gamma-synuclein
1.13E-09	ADIRF; adipogenesis regulatory factor
1.02E-08	FAM35A; shieldin complex subunit 2 isoform X2
2.75E-12	DNAJC12; dnaJ homolog subfamily C member 12
3.06E-06	RTKN2; rhotekin-2 isoform X3
3.08E-17	PRAP1; proline rich acidic protein 1 precursor
#######	HKDC1; hexokinase HKDC1 isoform X3
5.71E-35	SCD; stearoyl-CoA desaturase
2.59E-18	CYP2C23b; cytochrome P450 2H2 precursor
8.53E-08	CYP2C23a; cytochrome P450 2H1 precursor
3.45E-08	LOC100858647; beta-microseminoprotein
8.21E-51	ANXA8L1; annexin A8
4.23E-11	PANK1; pantothenate kinase 1 isoform X3
3.05E-07	PPP1R3C; protein phosphatase 1 regulatory subunit 3C
1.31E-18	CRTAC1; cartilage acidic protein 1
6.47E-07	HOGA1; 4-hydroxy-2-oxoglutarate aldolase, mitochondrial
1.69E-21	TLX1; T-cell leukemia homeobox protein 1
1.85E-07	PDZD7; PDZ domain-containing protein 7 isoform X4
3.18E-10	CYP17A1; steroid 17-alpha-hydroxylase/17,20 lyase precursor
1.23E-05	STN1; CST complex subunit STN1 isoform X4
2.55E-07	GSTO2; glutathione S-transferase omega-1
1.93E-20	GPAM; glycerol-3-phosphate acyltransferase 1, mitochondrial isoform X1
4.02E-08	SLC18A2; synaptic vesicular amine transporter isoform X2
4.60E-15	LOC101750892; deleted in malignant brain tumors 1 protein-like isoform X2
3.94E-16	MKI67; proliferation marker protein Ki-67 isoform X4
1.14E-09	C2orf88; small membrane A-kinase anchor protein isoform X1

7.77E-06 CPS1; carbamoyl-phosphate synthase [ammonia], mitochondrial isoform X7
4.95E-21 ABCA12; ATP-binding cassette sub-family A member 12 isoform X2
8.81E-07 ATIC; bifunctional purine biosynthesis protein ATIC
1.77E-18 LOC107048987; putative methyltransferase DDB_G0268948
4.60E-16 RAB17; ras-related protein Rab-17 isoform X1
5.15E-24 LOC121111295; UDP-glucuronosyltransferase 1A1-like
1.74E-16 AHR2; aryl hydrocarbon receptor 2 isoform X4
4.67E-33 AHR1B; aryl hydrocarbon receptor 1 beta isoform X2
1.74E-14 COL6A2; collagen alpha-2(VI) chain precursor
2.73E-07 NABP1; SOSS complex subunit B2
1.64E-17 COQ10B; coenzyme Q-binding protein COQ10 homolog B, mitochondrial
2.72E-07 SGO2; shugoshin 2 isoform X3
8.83E-05 GPR1; G-protein coupled receptor 1 isoform X2
3.79E-08 PDE11A; dual 3',5'-cyclic-AMP and -GMP phosphodiesterase 11A isoform X3
2.32E-10 CDCA7; cell division cycle-associated protein 7 isoform X2
8.20E-14 RAPGEF4; rap guanine nucleotide exchange factor 4 isoform X5
2.53E-05 SPC25; kinetochore protein Spc25
5.27E-06 CERS6; ceramide synthase 6 isoform X2
8.95E-10 DPP4; dipeptidyl peptidase 4
2.45E-15 SLC4A10; sodium-driven chloride bicarbonate exchanger isoform X6
9.07E-54 ITGB6; integrin beta-6 isoform X1
3.27E-12 LOC424199; uncharacterized protein LOC424199 isoform X1
0.000378 TUBA4AL; tubulin alpha-4A chain
8.95E-13 IGFBP2; insulin-like growth factor-binding protein 2 isoform X1
6.11E-16 SLC12A8; solute carrier family 12 member 8 isoform X1
1.48E-09 SCTR; secretin receptor precursor
3.70E-17 DPP10; inactive dipeptidyl peptidase 10 isoform X2
2.30E-06 UPP2; uridine phosphorylase 2 isoform X2
1.26E-07 DAPL1; death-associated protein-like 1
1.70E-07 RGS5; regulator of G-protein signaling 5
1.30E-08 SELP; P-selectin isoform X5
1.49E-11 REG4; regenerating islet-derived protein 4 precursor
9.79E-07 FASLG; tumor necrosis factor ligand superfamily member 6 isoform X1
1.71E-07 SUCO; SUN domain-containing ossification factor isoform X5
1.53E-08 NUF2; kinetochore protein Nuf2 isoform X1
5.49E-26 RGS8; regulator of G-protein signaling 8 isoform X2
3.36E-13 RGSL1; regulator of G-protein signaling protein-like isoform X1
1.47E-11 TOR3A; torsin-3A

9.60E-07 KIAA0040; uncharacterized protein KIAA0040 homolog
7.68E-40 CLCA1; calcium-activated chloride channel regulator 1-like
1.30E-11 LPAR3; lysophosphatidic acid receptor 3 isoform X1
2.28E-09 VTG2; vitellogenin-2 precursor
1.69E-11 VTG3; vitellogenin-3
1.52E-08 SPATA1; spermatogenesis-associated protein 1 isoform X3
4.71E-14 RBP; riboflavin-binding protein precursor
1.68E-06 TSPAN1; tetraspanin-1
9.31E-07 VDHAP; vitamin D3 hydroxylase-associated protein
7.82E-06 FAAH; fatty-acid amide hydrolase 1
2.72E-24 CYP4B7; cytochrome P450 4B7
2.15E-12 PODN; podocan isoform X3
1.31E-18 LEXM; lymphocyte expansion molecule isoform X5
4.71E-15 DHCR24; delta(24)-sterol reductase
2.74E-07 DAB1; disabled homolog 1
4.70E-07 CYP2J21; cytochrome P450, family 2, subfamily J, polypeptide 21 isoform X3
3.98E-06 KANK4; KN motif and ankyrin repeat domain-containing protein 4 isoform X1
5.80E-15 ANGPTL3; angiopoietin-related protein 3 precursor
0.000207 DNAJC6; putative tyrosine-protein phosphatase auxilin
3.83E-15 PTGER3; prostaglandin E2 receptor EP3 subtype
9.59E-08 KLHL24; kelch-like protein 24 isoform X1
2.68E-06 GPC2; glypican-5 isoform X1
2.43E-10 EHHADH; peroxisomal bifunctional enzyme isoform X2
9.73E-07 DNAJB11; dnaJ homolog subfamily B member 11
2.37E-07 LOC101748451; lysophosphatidic acid receptor 6-like isoform X1
7.36E-25 SGPP2; sphingosine-1-phosphate phosphatase 2
2.00E-05 MOGAT1; 2-acylglycerol O-acyltransferase 1
4.69E-05 PCOLCE2; procollagen C-endopeptidase enhancer 2 isoform X1
1.05E-05 PDCD1; programmed cell death protein 1
1.48E-05 LOC424919; deoxyribodipyrimidine photo-lyase isoform X1
1.07E-06 C2orf72; uncharacterized protein C2orf72 homolog
1.42E-17 ALPI; intestinal-type alkaline phosphatase
6.91E-06 VWA5B2; von Willebrand factor A domain-containing protein 5B2 isoform X1
3.96E-14 AHSG; alpha-2-HS-glycoprotein
2.27E-05 ECT2; protein ECT2 isoform X3
1.65E-13 GHSR; growth hormone secretagogue receptor type 1
2.71E-10 SERPINI1; neuroserpin precursor
4.14E-09 LOC107054133; SLIT and NTRK-like protein 3

3.50E-13 SMC4; structural maintenance of chromosomes protein 4
1.45E-08 IFT80; intraflagellar transport protein 80 homolog
1.22E-20 MLF1; myeloid leukemia factor 1 isoform X1
5.16E-14 TUBA3E; tubulin alpha-3 chain
2.83E-57 DUOX1; dual oxidase 2
5.71E-15 KIAA0101; PCNA-associated factor
0.001091 CHRNA3; neuronal acetylcholine receptor subunit alpha-3 precursor
1.64E-22 TRPM1; transient receptor potential cation channel subfamily M member 1 isoform X5
2.36E-57 LIPC; hepatic triacylglycerol lipase isoform X1
1.58E-05 DYX1C1; dynein assembly factor 4, axonemal
2.47E-07 SCG3; secretogranin-3 isoform 2 precursor
3.81E-10 SEMA6D; semaphorin-6D isoform X8
2.13E-07 GATM; glycine amidinotransferase, mitochondrial
2.52E-05 FSD2; fibronectin type III and SPRY domain-containing protein 2
3.49E-10 LMNAL; lamin-L(III) isoform X2
2.05E-17 LOC415472; uncharacterized protein LOC415472
1.57E-08 PEX11A; peroxisomal membrane protein 11A isoform X2
2.71E-08 ADAMTS17; A disintegrin and metalloproteinase with thrombospondin motifs 17 isoform X3
4.49E-08 ALDH1A3; aldehyde dehydrogenase family 1 member A3
3.27E-15 KIF23; kinesin-like protein KIF23 isoform X18
6.74E-08 CNGB1; cyclic nucleotide-gated cation channel beta-1 isoform X1
3.27E-12 CETP; cholesteryl ester transfer protein precursor
3.45E-05 CX3CL1; fractalkine precursor
1.71E-06 KIAA0895L; uncharacterized protein KIAA0895-like homolog isoform X3
2.81E-09 LOC769668; estradiol 17-beta-dehydrogenase 2-like
2.12E-05 LOC415664; uncharacterized oxidoreductase-like isoform X1
2.77E-07 LOC100857820; C-factor
7.61E-15 MT4; metallothionein
1.86E-05 MT3; metallothionein-3
5.32E-07 CDCA9; borealin-2
2.10E-05 RGS9BP; regulator of G-protein signaling 9-binding protein
8.23E-08 PDCD2L; programmed cell death protein 2-like
3.29E-17 CES1L2; fatty acyl-CoA hydrolase precursor, medium chain
3.83E-05 CES1L1; fatty acyl-CoA hydrolase precursor, medium chain isoform X1
2.59E-18 TERB1; telomere repeats-binding bouquet formation protein 1 isoform X3
5.08E-10 BEAN1; protein BEAN1
1.44E-07 CENPN; centromere protein N
5.40E-07 GINS2; DNA replication complex GINS protein PSF2 isoform X1

7.84E-08 CA5A; carbonic anhydrase 5A, mitochondrial isoform X2
1.45E-06 ZFPM1; zinc finger protein ZFPM1 isoform X5
1.64E-12 CIDEC; cell death activator CIDE-3 isoform X1
3.25E-07 CDT1; DNA replication factor Cdt1 isoform X1
7.66E-05 SPIRE2; protein spire homolog 2 isoform X1
8.91E-25 URAH; 5-hydroxyisourate hydrolase isoform X1
2.05E-05 CHST4; carbohydrate sulfotransferase 4
3.61E-59 LOC121106611; uncharacterized protein LOC121106611 isoform X2
3.37E-08 MUSTN1; musculoskeletal embryonic nuclear protein 1
8.02E-16 CISH; cytokine-inducible SH2-containing protein
1.15E-13 LOC121106469; guanylate-binding protein 2-like isoform X2
6.79E-43 LOC121106623; guanylate-binding protein 1-like
4.01E-07 LOC121106625; guanylate-binding protein 1-like
1.16E-07 LOC121106627; guanylate-binding protein 1-like
1.10E-10 USP4; ubiquitin carboxyl-terminal hydrolase 4
6.45E-05 GPX1; glutathione peroxidase 1
2.25E-06 ALAS1; 5-aminolevulinate synthase, nonspecific, mitochondrial precursor
1.60E-09 PTPDC1; protein tyrosine phosphatase domain-containing protein 1 isoform X5
1.11E-09 DNASE1L3; deoxyribonuclease gamma precursor
3.50E-19 ABHD6; monoacylglycerol lipase ABHD6 isoform X2
2.48E-09 IP6K2; inositol hexakisphosphate kinase 2 isoform X1
3.65E-05 MCM2; DNA replication licensing factor MCM2 isoform X1
1.30E-12 GPR27; probable G-protein coupled receptor 27
3.93E-20 PROK2; prokineticin-2
6.22E-05 SHQ1; protein SHQ1 homolog
4.22E-09 CNTN4; contactin-4 isoform X1
3.74E-09 BHLHE40; class E basic helix-loop-helix protein 40
0.000132 GHRL; appetite-regulating hormone preproprotein
1.74E-05 HBEGF; proheparin-binding EGF-like growth factor precursor
2.02E-11 KCNMB1; calcium-activated potassium channel subunit beta-1
4.74E-64 TENM2; teneurin-2 isoform 2
2.52E-25 GABRB2; gamma-aminobutyric acid receptor subunit beta-2 isoform X2
2.63E-06 PTTG2; securin isoform 1
3.81E-10 SLC26A2; sulfate transporter isoform X1
3.87E-08 PPARGC1B; peroxisome proliferator-activated receptor gamma coactivator 1-beta isoform X4
5.02E-05 DUSP1; dual specificity protein phosphatase 1
7.09E-13 STC2; stanniocalcin-2
2.96E-12 MXD3; lateral signaling target protein 2 homolog isoform X3

0.000108	NIPAL4; magnesium transporter NIPA4 isoform X1
9.54E-07	TRPC7; short transient receptor potential channel 7 isoform X1
1.30E-07	SLC25A48; solute carrier family 25 member 48
5.77E-08	SLC22A5; solute carrier family 22 member 5
1.00E-27	EGR1; early growth response protein 1
2.21E-24	BHLHA15; class A basic helix-loop-helix protein 15
7.99E-06	NPTX2; neuronal pentraxin-2 isoform X1
9.10E-06	CYP3A5; cytochrome P450 family 3 subfamily A member 5
2.61E-10	LOC101747873; urotensin-2 receptor
4.26E-06	PEMT; phosphatidylethanolamine N-methyltransferase isoform X2
2.20E-14	CACNA1H; voltage-dependent T-type calcium channel subunit alpha-1H isoform X2
0.000536	SOX8; transcription factor SOX-8
4.06E-14	MEIOB; meiosis-specific with OB domain-containing protein isoform X1
3.93E-07	PLK1; serine/threonine-protein kinase PLK1
1.38E-07	IL21R; interleukin-21 receptor precursor
8.89E-07	RMI2; recQ-mediated genome instability protein 2
#######	GRIN2A; glutamate receptor ionotropic, NMDA 2A isoform X5
2.21E-15	RHBDF1; inactive rhomboid protein 1 isoform X2
2.00E-14	HBAD; hemoglobin subunit alpha-D
1.18E-15	HBA1; hemoglobin subunit alpha-A
1.08E-21	PDIA2; protein disulfide-isomerase A2
1.08E-10	RGS11; regulator of G-protein signaling 11 isoform X1
4.07E-91	LOC416655; protein PERCC1 isoform X1
1.03E-05	C16orf96; uncharacterized protein C16orf96 homolog isoform X3
2.69E-14	LOC121106812; cyclin-F-like isoform X1
3.14E-08	GPRC5B; G-protein coupled receptor family C group 5 member B isoform X2
1.70E-08	SDF2L1; stromal cell-derived factor 2-like protein 1
4.49E-08	CDC45; cell division control protein 45 homolog isoform X2
9.57E-19	AACS; acetoacetyl-CoA synthetase
4.99E-21	RAD9B; cell cycle checkpoint control protein RAD9B isoform X2
8.90E-12	PPTC7; protein phosphatase PTC7 homolog
1.90E-05	TMEM116; transmembrane protein 116 isoform X3
7.80E-12	UNG; uracil-DNA glycosylase
1.62E-18	ACACB; acetyl-CoA carboxylase 2 isoform X5
5.56E-05	CRYBB3; beta-crystallin B3
9.98E-09	VPS29L; vacuolar protein sorting 29 homolog
3.32E-09	SLC2A11L1; solute carrier family 2, facilitated glucose transporter member 11-like
2.25E-05	SLC5A1; sodium/glucose cotransporter 1

9.23E-09 LOC416959; melanotransferrin isoform X2
7.95E-12 PISD; phosphatidylserine decarboxylase proenzyme, mitochondrial isoform X1
4.61E-11 LOC100858984; dynein light chain 2, cytoplasmic
4.91E-13 PRODH; proline dehydrogenase 1, mitochondrial isoform X1
6.25E-11 SLC35E4; solute carrier family 35 member E4
5.18E-06 TCN2; transcobalamin-2
1.63E-06 MTFP1; mitochondrial fission process protein 1 isoform X1
1.83E-05 HORMAD2; HORMA domain-containing protein 2 isoform X9
1.71E-11 LOC417013; acyl-CoA dehydrogenase family member 11 isoform X2
1.47E-28 NEFH; neurofilament heavy polypeptide isoform X1
5.36E-05 MHCY14; major histocompatibility complex-Y, class I heavy chain, 14 precursor
1.14E-19 LOC121106942; C-type lectin domain family 2 member I-like isoform X5
8.39E-07 MHCY9; major histocompatibility complex Y, class I heavy chain 9 isoform X3
7.43E-61 LENG9L7; leukocyte receptor cluster member 9-like, MHC-Y region, 7
0.000161 OZFL; OZF like, MHCY region
2.67E-07 YLEC17; c-type lectin-like protein, MHC-Y region, 17
2.20E-12 LOC121106436; class I histocompatibility antigen, F10 alpha chain-like isoform X1
4.01E-14 MHCY12; major histocompatibility complex Y, class I heavy chain 12 isoform X1
2.16E-41 LOC121106918; class I histocompatibility antigen, F10 alpha chain-like isoform X2
8.18E-27 LOC121106502; C-type lectin domain family 2 member I-like isoform X5
2.52E-34 LOC121106943; C-type lectin domain family 2 member E-like isoform X1
2.95E-13 MHCY32; major histocompatibility complex Y, class I heavy chain 32
3.92E-07 BG8; MHC B-G antigen isoform 1 precursor
1.25E-16 LOC121106920; uncharacterized protein LOC121106920 isoform X18
4.64E-19 BTN3A3L2; uncharacterized protein BTN3A3L2 isoform X1
8.25E-06 IL4I1; L-amino-acid oxidase precursor
8.63E-22 TRIM7.1; tripartite motif-containing protein 7 isoform X5
1.70E-05 TRIM39.2; tripartite motif containing 39
1.55E-05 TRIM27.1; tripartite motif-containing 27 isoform X1
2.28E-06 DMB2; major histocompatibility complex, class II, DM beta 2 isoform X1
0.040286 CENPA; histone H3-like centromeric protein A
2.29E-06 CYP21A1; steroid 21-hydroxylase precursor
2.15E-09 CD1C; CD1c molecule precursor
1.98E-06 LOC107049645; uncharacterized protein LOC107049645 isoform X2
8.68E-12 LOC121107090; erythropoietin receptor-like
9.96E-07 FCN2; ficolin-2 isoform X12
4.32E-05 MAN1B1; endoplasmic reticulum mannosyl-oligosaccharide 1,2-alpha-mannosidase isoform X2
4.16E-46 NSMF; NMDA receptor synaptonuclear signaling and neuronal migration factor isoform X2

3.83E-09 GSL; glutamine synthetase
0.000136 TNFSF8; tumor necrosis factor ligand superfamily member 8
5.60E-07 PAPPA; pappalysin-1
1.50E-08 LOC107052201; alpha-1-acid glycoprotein
2.24E-17 SLC25A25; calcium-binding mitochondrial carrier protein SCaMC-2 isoform X4
2.32E-12 FPGS; folylpolyglutamate synthase, mitochondrial isoform X9
0.000491 CERCAM; inactive glycosyltransferase 25 family member 3 isoform X2
3.81E-10 DNM1; dynamin-1 isoform X8
3.33E-08 CRAT; carnitine O-acetyltransferase isoform X1
7.34E-36 LAMC3; laminin subunit gamma-3 isoform X3
9.06E-12 NTNG2; netrin-G2 isoform X1
9.44E-20 AGPAT2; 1-acyl-sn-glycerol-3-phosphate acyltransferase beta isoform X2
6.59E-12 LOC417113; carboxyl-terminal PDZ ligand of neuronal nitric oxide synthase protein
1.93E-14 HSPA5; endoplasmic reticulum chaperone BiP isoform X1
1.72E-14 CDK3; cyclin-dependent kinase 3 isoform X2
2.75E-18 TEN1; CST complex subunit TEN1 isoform X2
6.70E-12 ACOX1; peroxisomal acyl-coenzyme A oxidase 1 isoform X3
9.88E-09 FBF1; fas-binding factor 1 homolog isoform X12
7.43E-06 MRPL38; 39S ribosomal protein L38, mitochondrial
1.93E-10 TRIM65; tripartite motif-containing protein 65
1.09E-05 SMIM5; small integral membrane protein 5
1.14E-17 FASN; fatty acid synthase
0.000285 NOTUM; palmitoleoyl-protein carboxylesterase NOTUM
9.04E-10 KCNJ16; inward rectifier potassium channel 16
9.38E-07 GAA; lysosomal alpha-glucosidase isoform X1
1.86E-05 CBX2; chromobox protein homolog 2
3.59E-13 SOCS3; suppressor of cytokine signaling 3
3.39E-15 TK1; thymidine kinase, cytosolic
0.000281 KRABZFP; uncharacterized protein LOC769812 precursor
4.16E-21 GRIN2C; glutamate receptor ionotropic, NMDA 2C isoform X3
1.66E-09 HN1; jupiter microtubule associated homolog 1 isoform 3
2.83E-22 SLC16A5; monocarboxylate transporter 6
5.71E-09 MIS12; protein MIS12 homolog
4.26E-05 TMEM120A; ion channel TACAN
8.66E-06 LOC121106433; C-C motif chemokine 3-like
5.00E-05 SRR; serine racemase isoform X7
1.27E-08 SEBOX; homeobox protein SEBOX isoform X1
9.36E-13 TUSC5; trafficking regulator of GLUT4 1

6.23E-07	MED13; mediator of RNA polymerase II transcription subunit 13 isoform X3
7.10E-10	ACACA; acetyl-CoA carboxylase isoform X1
5.34E-07	EVI2A; protein EVI2A precursor
5.11E-07	FBXO39; F-box only protein 39 isoform X2
4.12E-08	ASIP; agouti-signaling protein precursor
8.10E-07	TOX2; TOX high mobility group box family member 2 isoform X1
1.67E-06	ACSS2; acetyl-coenzyme A synthetase, cytoplasmic isoform X8
8.80E-28	RBPJL; recombining binding protein suppressor of hairless-like protein isoform X3
1.98E-37	PI3; elafin
2.56E-07	TTPAL; alpha-tocopherol transfer protein-like isoform X1
2.11E-06	LOC121107299; uncharacterized protein LOC121107299
6.76E-21	EPB42; protein 4.2
1.42E-10	GATA5; transcription factor GATA-5 isoform X1
1.32E-20	LOC771972; uncharacterized protein LOC771972 isoform X1
5.38E-07	LOC100859272; WAP four-disulfide core domain protein 2 isoform X1
4.91E-20	WFDC2; WAP four-disulfide core domain protein 3 isoform X4
8.93E-20	WFDC8; balbiani ring protein 3 isoform X2
2.37E-09	SPINT4; kunitz-type protease inhibitor 4
7.76E-30	LOC771994; protein qua-1
2.41E-11	UBE2U; ubiquitin-conjugating enzyme E2 C
4.03E-10	MMP9; matrix metalloproteinase-9 precursor
0.00026	PCK1; phosphoenolpyruvate carboxykinase, cytosolic [GTP]
4.04E-07	UTS2; urotensin-2 precursor
1.55E-07	PER3; period circadian protein homolog 3 isoform X14
1.83E-07	ESPN; espin isoform X6
2.33E-60	AJAP1; adherens junction-associated protein 1
7.40E-05	KBP; probable glutamate receptor isoform X1
7.76E-08	MIB2; E3 ubiquitin-protein ligase MIB2
7.24E-32	LOC419409; Golgi integral membrane protein 4 isoform X1
4.92E-09	LOC771069; C-factor isoform X2
1.73E-08	SLC25A33; solute carrier family 25 member 33 isoform X1
1.16E-05	RNF186; E3 ubiquitin-protein ligase RNF186
0.000678	CELA2A; chymotrypsin-like elastase family member 2A precursor
7.98E-09	GUCA2B; guanylate cyclase activator 2A (guanylin) precursor
0.000196	WNT4; protein Wnt-4 precursor
1.82E-06	LOC112530142; uncharacterized protein LOC112530142
1.21E-23	DPYSL2; dihydropyrimidinase-related protein 2 isoform X2
3.05E-07	LOC101749127; uncharacterized protein LOC101749127 isoform X1

7.65E-08 ANK1; ankyrin-1 isoform X13
7.40E-05 ADD2; beta-adducin
1.50E-08 SLC20A1; sodium-dependent phosphate transporter 1
2.48E-23 CASP14; caspase-14
2.86E-10 RETSAT; all-trans-retinol 13,14-reductase
0.000688 LOC101749201; peptide methionine sulfoxide reductase MsrA isoform X3
6.94E-07 TRNAU1AP; tRNA selenocysteine 1-associated protein 1 isoform X4
2.53E-09 STMN1; stathmin isoform X1
1.72E-05 LSM10; U7 snRNA-associated Sm-like protein LSm10
1.33E-10 FAM167B; protein FAM167B isoform X2
2.66E-29 MYCL; protein L-Myc
7.13E-06 MFSD2A; sodium-dependent lysophosphatidylcholine symporter 1
1.22E-23 GALE; UDP-glucose 4-epimerase isoform X1
3.47E-23 HMGCL; hydroxymethylglutaryl-CoA lyase, mitochondrial isoform 4
6.38E-28 IL22RA1; interleukin-22 receptor subunit alpha-1 precursor
3.82E-08 ADAMTS15; A disintegrin and metalloproteinase with thrombospondin motifs 15
7.05E-08 NCAPD3; condensin-2 complex subunit D3 isoform X3
4.14E-08 SCN3B; sodium channel subunit beta-3 isoform X1
7.25E-07 GRAMD1B; protein Aster-B isoform X17
3.98E-06 TMEM136; transmembrane protein 136 isoform X2
0.001637 THY1; thy-1 membrane glycoprotein isoform X1
1.71E-13 LOC101751887; NF-kappa-B inhibitor delta
1.74E-14 POU2AF1; POU domain class 2-associating factor 1 isoform X2
1.93E-10 ZPR1; zinc finger protein ZPR1 isoform X2
1.65E-16 APOA5; apolipoprotein A-V
7.84E-11 FXYD2; sodium/potassium-transporting ATPase subunit gamma
5.24E-10 MPZL2; myelin protein zero-like protein 2 precursor
1.12E-44 LOC101748511; uncharacterized protein LOC101748511 isoform X4
4.01E-10 HYOU1; hypoxia up-regulated protein 1 isoform X1
1.07E-08 NCAM1; neural cell adhesion molecule 1 isoform X14
9.92E-21 BGLAP; osteocalcin preproprotein
2.85E-11 PEAR1; platelet endothelial aggregation receptor 1
1.78E-06 CD244; natural killer cell receptor 2B4 isoform X2
6.84E-11 SLAMF1; signaling lymphocytic activation molecule
1.76E-08 S100A12; protein MRP-126
5.29E-10 CKS1B; cyclin-dependent kinases regulatory subunit 1
3.94E-08 FDPS; farnesyl pyrophosphate synthase
0.000197 SYT11; synaptotagmin-11 isoform X1

7.33E-10 CIART; circadian-associated transcriptional repressor isoform X1
2.25E-07 CA14; carbonic anhydrase 14 isoform X3
1.49E-06 TMOD4; tropomodulin-4
8.71E-13 LOC121107545; prefoldin subunit 2-like
9.19E-08 THEM4; acyl-coenzyme A thioesterase THEM4 isoform X2
2.16E-11 LOC121106470; deaminated glutathione amidase-like isoform X7
9.86E-29 PFDN2; prefoldin subunit 2 isoform X1
2.65E-15 TULP1; tubby-related protein 1 isoform X1
1.74E-16 ELF3; ETS-related transcription factor Elf-3
6.69E-06 PTPRVP; receptor-type tyrosine-protein phosphatase V isoform X3
4.36E-42 CSRP1; cysteine and glycine-rich protein 1
0.000249 UBE2T; ubiquitin-conjugating enzyme E2 T isoform X2
2.23E-10 NUAK2; NUAK family SNF1-like kinase 2
1.15E-08 LOC121107619; Krueppel-like factor 15 isoform X1
1.59E-68 SLC26A9; solute carrier family 26 member 9
7.91E-14 CD34; hematopoietic progenitor cell antigen CD34 isoform X4
9.96E-38 CAMK1G; calcium/calmodulin-dependent protein kinase type 1G
8.40E-37 LAMB3; laminin subunit beta-3
2.74E-17 G0S2; G0/G1 switch protein 2
6.22E-17 SLC16A1; monocarboxylate transporter 1
5.85E-11 CHIA-M31; acidic mammalian chitinase isoform X1
7.36E-25 LOC768786; acidic mammalian chitinase isoform X1
8.52E-05 LOC107049117; maestro heat-like repeat-containing protein family member 7 isoform X1
2.21E-10 VWA5A1; von Willebrand factor A domain-containing protein 5A isoform X8
8.80E-10 DBF4B; protein DBF4 homolog B isoform X3
0.000164 GH; somatotropin precursor
1.33E-17 ACE; angiotensin-converting enzyme precursor
3.61E-19 SLC4A1; band 3 anion transport protein isoform X3
2.53E-08 COL1A1; collagen alpha-1(I) chain isoform X1
2.70E-05 LOC107050147; pyruvate dehydrogenase kinase, isozyme 2 isoform X2
7.63E-06 LOC429785; G protein-activated inward rectifier potassium channel 1
1.21E-09 PPP1R1B; protein phosphatase 1 regulatory subunit 1B isoform X1
3.27E-12 NR1D1; LOW QUALITY PROTEIN: nuclear receptor subfamily 1 group D member 1 isoform X1
1.41E-19 TOP2A; DNA topoisomerase 2-alpha
1.49E-13 CCR7; C-C chemokine receptor type 7 precursor
2.46E-08 KRT40; keratin, type I cytoskeletal 20
5.89E-13 KRT24; keratin, type I cytoskeletal 19
6.48E-08 P3H4; endoplasmic reticulum protein SC65

2.14E-10	G6PC; glucose-6-phosphatase
9.15E-09	VAT1; synaptic vesicle membrane protein VAT-1 homolog
7.82E-08	RND2; rho-related GTP-binding protein RhoN
5.37E-12	BRCA1; breast cancer type 1 susceptibility protein isoform X5
6.13E-09	SLC1A6; excitatory amino acid transporter 4 isoform X2
1.69E-08	LOC100857333; platelet glycoprotein Ib alpha chain isoform X1
1.04E-11	C19orf71; uncharacterized protein C19orf71 homolog
7.66E-09	CTSG; mast cell protease 1A
8.27E-40	ACSBG2; long-chain-fatty-acid--CoA ligase ACSBG2 isoform X4
7.82E-08	LRG1; leucine-rich alpha-2-glycoprotein isoform X1
7.50E-14	CREB3L3; cyclic AMP-responsive element-binding protein 3-like protein 3 isoform X2
2.71E-08	PCSK4; proprotein convertase subtilisin/kexin type 4 isoform X3
1.17E-21	ADAMTSL5; ADAMTS-like protein 5 isoform X3
1.05E-11	LOC100859819; cocaine- and amphetamine-regulated transcript protein isoform X5
4.08E-06	COMP; cartilage oligomeric matrix protein
5.04E-14	LOC121107774; zinc finger protein 34-like isoform X1
1.47E-10	ACP5; tartrate-resistant acid phosphatase type 5 isoform X1
8.45E-07	LOC769841; sulfotransferase 2B1 isoform X2
1.98E-28	LOC101751912; butyrophilin subfamily 3 member A2-like isoform X8
8.85E-26	LOC107049467; uncharacterized protein LOC107049467 isoform X2
5.32E-17	LOC107050037; leukocyte immunoglobulin-like receptor subfamily B member 3A
3.30E-16	LOC112531104; osteoclast-associated immunoglobulin-like receptor isoform X3
0.000227	LOC100859870; leukocyte immunoglobulin-like receptor subfamily A member 2
1.91E-05	LOC121107808; leukocyte immunoglobulin-like receptor subfamily B member 1
5.65E-05	CHIR-A2; immunoglobulin-like receptor CHIR-A2 precursor
8.95E-07	CHIR-AB1; immunoglobulin-like receptor CHIR-AB1 precursor
1.83E-05	LOC107049866; osteoclast-associated immunoglobulin-like receptor
7.99E-09	LOC112531107; platelet glycoprotein VI-like
0.00055	LOC121107881; uncharacterized protein LOC121107881 isoform X2
0.001538	LOC121107862; platelet glycoprotein VI-like
4.56E-16	LOC107049967; uncharacterized protein LOC107049967 isoform X1
1.02E-09	LOC121107814; LOW QUALITY PROTEIN: leukocyte immunoglobulin-like receptor subfamily A member 6
1.33E-09	LOC112531135; platelet glycoprotein VI-like
6.06E-05	LOC121107865; LOW QUALITY PROTEIN: leukocyte immunoglobulin-like receptor subfamily A member 2
5.36E-17	CHIR-AB-600; platelet glycoprotein VI isoform X3
1.04E-06	LOC107050473; LOW QUALITY PROTEIN: platelet glycoprotein VI-like isoform X1
2.47E-06	LOC107049819; uncharacterized protein LOC107049819
0.000572	LOC121107930; CLK4-associating serine/arginine rich protein-like

2.42E-06 RACGAP1; rac GTPase-activating protein 1 isoform X3
1.73E-06 LOC107049666; integrin beta-7 isoform X2
1.66E-08 APOF; apolipoprotein F
8.99E-13 LOC121108060; maestro heat-like repeat-containing protein family member 2A isoform X2
2.36E-17 LOC121106503; maestro heat-like repeat-containing protein family member 2A isoform X2
2.74E-05 LOC112531456; uncharacterized protein LOC112531456 isoform X1
6.66E-09 LOC121108145; uncharacterized protein LOC121108145 isoform X1
2.79E-05 LOC121108155; RUS family member 1-like isoform X2
0.000485 LOC121108156; translation initiation factor IF-2-like isoform X1
1.06E-07 LOC107055390; mRNA decay activator protein ZFP36-like isoform X1
3.40E-16 OTX5; cone-rod homeobox protein isoform X1
1.06E-12 LOC107050328; uncharacterized protein LOC107050328
4.09E-07 LOC426220; avidin isoform X1
3.25E-07 LOC121108197; histidine triad nucleotide-binding protein 1-like
5.81E-66 LOC121108210; uncharacterized protein LOC121108210
3.47E-14 ALPK2; alpha-protein kinase 2
1.28E-05 CNTFR; ciliary neurotrophic factor receptor subunit alpha isoform X7
4.47E-13 LOC100857191; C-C motif chemokine 26
4.45E-24 AVD; avidin precursor
7.68E-05 GZMA; granzyme A precursor
0.000453 CENPK; centromere protein K
4.49E-08 S100Z; protein S100-Z
1.04E-08 LOC100858941; uncharacterized protein LOC100858941 isoform X3
1.92E-15 GLDC; glycine dehydrogenase (decarboxylating), mitochondrial precursor
2.96E-12 FBP2; fructose-1,6-bisphosphatase isozyme 2
3.66E-22 GADD45G; growth arrest and DNA damage-inducible protein GADD45 gamma
2.16E-10 NFIL3; nuclear factor interleukin-3-regulated protein
3.53E-15 LOC121108275; uncharacterized protein LOC121108275
2.58E-18 SHB; SH2 domain-containing adapter protein B isoform X1
1.35E-05 LPL; lipoprotein lipase precursor
1.55E-07 PSD3; PH and SEC7 domain-containing protein 3 isoform X4
3.77E-25 LOC768418; zinc finger FYVE domain-containing protein 16-like
1.73E-06 KIAA1958; uncharacterized protein KIAA1958 homolog isoform X1
8.16E-10 SMC2; structural maintenance of chromosomes protein 2
9.73E-05 TUSC1; tumor suppressor candidate gene 1 protein
2.83E-17 CDKN2A; ARF tumor suppressor
1.79E-20 CDKN2B; cyclin-dependent kinase 4 inhibitor B
1.02E-08 CCDC112; coiled-coil domain-containing protein 112 isoform X3

8.95E-08 LOC107050717; calumenin isoform X1
3.90E-29 LOC121108653; uncharacterized protein LOC121108653
1.75E-05 LOC121108657; platelet glycoprotein VI-like
1.47E-05 CHIR-IG1-5; immunoglobulin-like receptor CHIR-AB1-like precursor
3.71E-05 LOC121108673; osteoclast-associated immunoglobulin-like receptor
4.53E-15 LOC107050652; putative killer cell immunoglobulin-like receptor-like protein KIR3DX1
1.47E-10 PKMYT1; membrane-associated tyrosine- and threonine-specific cdc2-inhibitory kinase isoform X2
1.10E-12 LOC121108741; vasodilator-stimulated phosphoprotein-like isoform X1
2.04E-17 LOC121108762; uncharacterized protein LOC121108762 isoform X1
4.96E-06 LOC121108855; uncharacterized protein LOC121108855 isoform X2
3.11E-10 LOC107050463; clumping factor A-like
0.000369 LOC112531297; uncharacterized protein LOC112531297 isoform X1
4.14E-14 LOC107049937; uncharacterized protein LOC107049937
3.53E-15 LOC121108923; mental retardation GTPase activating protein homolog 4-like
7.70E-08 LOC121108934; sphingomyelin phosphodiesterase 5-like
5.16E-08 LOC121108936; CXXC-type zinc finger protein 1-like
4.85E-17 MHCY13; major histocompatibility complex-Y, class I heavy chain, 13 precursor
1.17E-13 LOC112530071; NF-kappa-B inhibitor delta-like isoform X1
2.89E-05 LOC121108947; LOW QUALITY PROTEIN: medium-chain specific acyl-CoA dehydrogenase, mitochondrial isoform X1
