# Supplementary material for: Comparative analysis of liver transcriptome reveals adaptive responses to hypoxia environmental condition in Tibetan chicken
Source: Anim Biosci. 2023 Aug 23;37(1):28–38. doi: 10.5713/ab.23.0126 (PMC10766467; doi:10.5713/ab.23.0126)
Supplement: Supplementary file 4 [file ab-23-0126-Supplementary-Table-4.pdf]

Table S4

Geneid	HT1	HT2	HT3	HT4	HT5	QY1	QY2	QY3	QY4	QY5	logFC	type	pvalue
LOC11253	5.471	4.844	26.895	5.635	2.36	1.632	2.276	1.172	9.133	2.588	-1.42767	down	2.44E-10
LOC12111	0.514	0.62	0.011	0.033	0.014	0.033	0.015	0.025	5.044	3.135	2.786195	up	1.43E-28
GOLGB1	6.026	6.207	4.936	4.485	5.972	15.528	15.258	13.601	11.27	7.899	1.201854	up	9.88E-09
ELAPOR2	2.989	3.226	1.334	0.728	1.061	4.755	16.838	24.015	39.849	0.575	3.202999	up	2.30E-46
ALG12	12.218	11.172	12.612	15.737	13.32	52.359	51.735	62.099	47.778	27.195	1.890125	up	6.22E-19
CRELD2	16.486	14.861	20.903	18.59	14.191	90.801	88.226	122.963	67.198	30.143	2.231457	up	1.29E-25
LOC42934	0.058	0.036	0.005	0.017	0.006	0.226	6.019	2.046	1.664	0.037	6.298595	up	#######
TRABD	6.048	5.907	9.455	4.535	6.353	16.179	27.276	29.964	28.331	7.342	1.755871	up	1.58E-17
IQUB	0.993	0.449	2.583	0.444	0.465	0.518	0.411	0.257	0.289	0.504	-1.31581	down	3.27E-08
CPED1	3.776	3.847	5.473	5.571	5.853	0.998	1.469	2.286	0.85	1.486	-1.78958	down	2.73E-17
CAV1	0.264	1.08	1.577	1.139	1.492	3.46	3.637	1.481	2.246	3.318	1.348118	up	2.12E-08
AMIGO2	1.022	1.06	1.593	1.993	2.793	0.403	0.562	1.141	0.568	0.761	-1.29927	down	1.61E-09
SRGAP1	6.908	6.068	5.45	1.341	3.581	1.089	1.331	1.163	0.987	1.317	-1.98678	down	6.59E-21
IFNG	0.452	0.295	0.408	0.099	0.062	1.117	0.135	0.931	1.548	0.89	1.808135	up	4.25E-12
TSPAN8	0.838	0.763	1.015	0.629	1.309	2.291	2.703	2.209	1.268	4.488	1.507722	up	1.03E-10
LUM	2.031	1.65	5.504	2.221	5.855	11.213	6.372	4.589	9.072	7.653	1.171985	up	8.89E-09
IKBIP	2.156	1.726	2.719	2.608	2.517	6.175	4.049	7.217	6.366	4.117	1.251438	up	3.49E-09
OC3	0.122	0.08	0.13	0.201	0.292	0.295	2.241	1.48	0.322	0.601	2.574496	up	8.16E-19
HIST1H2A	1.223	1.111	1.677	2.4	0.698	0.657	0.382	0.535	0.645	0.937	-1.17028	down	2.96E-06
LOC1211C	0.85	0.86	0.923	1.207	0.238	3.616	1.825	2.156	5.627	4.267	2.099324	up	3.81E-16
LOC10085	0.424	0.324	0.282	0.349	0.799	1.254	0.557	0.429	1.51	0.976	1.115833	up	0.000163
HIST1H2B	0.184	0.241	0.366	0	0.378	0.742	0.31	0.167	0.801	2.901	2.068984	up	6.37E-09
GUCY2C	0.047	0.025	0.142	0.054	0.319	1.275	0.021	0.04	0.074	0.139	1.392317	up	9.42E-09
GBE	0.033	0.079	2.065	1.056	0.533	3.363	1.482	1.224	2.566	3.681	1.7081	up	1.63E-16
LOC41797	0	0.266	23.459	10.244	2.214	28.767	14.963	13.677	21.578	23.987	1.508739	up	3.91E-14
MEI1	1.876	1.816	1.172	1.415	2.148	0.668	0.295	0.47	0.504	1.168	-1.43896	down	9.90E-13
ADSL	312.305	295.773	586.536	605.692	277.771	138.019	306.537	93.652	67.652	381.319	-1.07386	down	1.04E-07
NPTXR	2.066	1.895	0.571	0.682	0.203	12.176	10.128	18.313	9.761	1.711	3.26422	up	1.28E-46
DNAL4	2.011	1.833	1.659	2.273	2.822	4.503	4.603	3.865	4.916	3.699	1.025958	up	2.07E-06
FAM20CL	433.81	459.966	1.954	25.555	0.424	951.043	1171.525	1421.014	951.698	62.39	2.305907	up	2.21E-25
SUN2	18.332	17.316	9.806	6.471	7.075	73.888	55.795	90.124	67.725	12.366	2.345587	up	2.80E-27
CBY1	0.478	0.826	1.929	0.42	0.631	1.568	2.8	1.613	0.378	2.79	1.093761	up	1.01E-06
LGALS1A	0.474	0.362	3.586	1.688	1.461	3.504	3.287	3.018	3.375	4.75	1.243591	up	5.29E-08
CARD10	1.29	1.45	2.944	2.441	0.998	0.388	0.633	0.253	1.006	2.184	-1.03035	down	3.02E-07
CSF2RB	0.238	0.393	0.341	0.579	0.196	0.515	0.846	0.387	1.356	0.516	1.048978	up	5.49E-06
MCM5	0.54	0.535	0.614	0.596	0.998	1.493	0.867	2.049	2.099	1.749	1.329281	up	2.88E-09

HSP90B1	157.01	148.585	216.797	181.815	205.894	923.701	881.427	1112.967	633.097	424.869	2.127235	up	9.87E-24
ASCL1	5.587	5.175	8.032	2.459	3.31	0.928	1.435	1.053	1.821	2.063	-1.74982	down	7.90E-16
SVOPL	0.209	0.232	0.035	0.036	0	0.872	0.526	1.031	0.55	0.031	2.543922	up	1.26E-25
CGTL	10.907	8.938	16.05	5.069	31.518	56.49	16.347	18.949	36.495	55.798	1.34457	up	1.46E-10
MICAL3	1.468	1.482	2.451	1.217	1.927	6.166	7.396	2.904	5.164	2.804	1.515189	up	1.45E-13
USP41	2.549	2.52	5.669	4.382	2.167	16.05	1.499	15.925	18.368	2.584	1.654324	up	4.53E-15
AKR1E2	1.799	2.14	3.315	2.908	6.322	11.16	7.221	12.9	11.927	5.554	1.564401	up	7.36E-14
LOC42513	2.991	3.243	1.902	1.998	5.493	14.044	8.068	22.769	15.227	9.667	2.158311	up	1.92E-23
EPS8	1.2	1.249	2.784	1.733	1.661	4.612	3.739	3.756	3.3	3.818	1.155592	up	2.15E-08
SLCO1B1	1.066	0.722	0.939	0.309	0.386	8.918	6.359	13.109	2.689	3.238	3.323947	up	6.62E-48
SOX5	1.501	1.272	1.747	1.25	1.303	0.64	0.424	0.656	0.491	0.569	-1.34566	down	7.48E-11
LDHB	371.616	323.912	870.705	415.305	520.845	184.775	85.323	122.895	119.769	122.322	-1.97827	down	2.08E-20
SSPN	0.694	0.719	3.161	1.225	0.776	5.504	6.84	7.282	3.613	2.437	1.964542	up	9.02E-22
BHLHE41	1.708	1.624	7.576	3.005	1.425	13.051	18.109	18.666	9.861	5.34	2.083569	up	3.73E-24
ENDOUL	1.525	1.284	0.447	0.403	1.314	0.565	6.701	2.508	2.954	0.484	1.408757	up	4.74E-11
MPPED1	1.909	1.919	3.455	3.065	2.405	1.268	0.984	0.991	0.979	1.176	-1.23957	down	3.38E-09
SULT4A1	3.1	3.017	2.947	1.635	1.33	11.165	18.066	7.892	11.603	1.322	2.05634	up	3.33E-22
PNPLA3	156.704	150.92	24.41	32.198	22.297	508.952	522.261	375.766	635.84	39.196	2.429317	up	2.56E-28
PARVG	0.833	1.199	1.977	1.356	0.976	2.526	1.517	2.455	4.073	3.186	1.116771	up	2.79E-07
KIAA0930	11.283	11.171	9.327	8.652	9.075	21.445	21.115	25.227	28.751	15.668	1.180335	up	2.31E-08
MANSC1	1.901	1.486	1.474	2.536	0.862	6.846	7.687	1.586	4.648	2.287	1.480418	up	1.88E-11
A2ML3	9.361	12.466	9.122	12.015	43.861	7.223	5.842	6.614	5.788	8.172	-1.36784	down	3.31E-11
CASR	1.936	1.763	1.204	1.279	0.076	0.084	1.298	0.384	0.648	0.154	-1.2834	down	2.86E-09
CNP1	3.375	3.785	4.095	2.708	1.362	1.102	1.626	0.755	0.668	1.861	-1.34924	down	8.00E-10
CDCA3	0.108	0.236	0.287	0.119	0.371	1.135	0.568	1.029	2.77	1.387	2.614134	up	3.62E-22
LRRC23	6.919	5.962	3.119	4.935	3.728	2.547	1.849	1.805	1.9	2.573	-1.20786	down	2.30E-09
EPHA1	0.234	0.347	0.187	0.234	0.209	2.226	0.699	1.804	0.339	0.532	2.204571	up	5.63E-21
GSTK1	25.813	24.89	36.138	24.733	60.593	10.749	9.968	10.155	13.778	17.547	-1.46882	down	1.35E-12
KEL	0.295	0.55	1.339	0.171	0.418	0.133	0.166	0.121	0.069	0.214	-1.97223	down	4.02E-16
LOC10085	0.216	0.094	1.373	0	0.074	0	0	0	0	0.212	-3.02145	down	5.18E-09
CLDND1	82.942	76.685	23.146	32.862	17.209	108.993	174.134	187.339	183.529	24.19	1.542295	up	6.46E-13
APOV1	9688.972	12281.13	57.327	2791.273	5.816	15162.62	18392.71	21437.53	21450.65	728.6	1.636313	up	1.32E-13
DPT	0.487	0.599	1.194	0.384	0.64	1.791	1.511	1.701	0.663	1.133	1.039988	up	1.68E-06
LOC41841	17.461	16.654	13.158	18.336	30.201	9.683	5.641	2.332	5.478	21.892	-1.08933	down	1.69E-08
CD200L	0	0	0.023	0.313	0.211	0.165	0.033	0.32	3.128	1.761	3.293422	up	1.52E-28
CREG1	48.616	45.349	90.265	84.383	124.389	18.777	30.037	56.41	23.292	61.699	-1.04689	down	2.92E-07
BCL9	3.518	3.118	7.03	3.542	2.862	2.204	1.658	1.524	2.094	2.001	-1.08153	down	2.36E-07
ADAMTS1	0.289	0.327	0.705	0.609	0.702	1.063	2.229	2.083	0.906	0.797	1.425463	up	1.79E-11

MAP3K7C	6.165	6.29	15.608	12.845	8.111	63.409	57.368	34.885	35.61	41.286	2.246061	up	5.75E-26
HUNK	1.435	1.244	1.577	1.201	1.166	6.388	6.651	0.923	10.719	1.544	1.984572	up	1.34E-20
EVA1C	7.313	8.262	15.975	6.286	11.069	3.802	1.839	2.757	3.007	6.212	-1.47275	down	2.19E-12
GART-B	0.305	0.262	1.808	0.246	0.803	0.533	0.25	0.216	0.106	0.338	-1.24373	down	1.26E-07
PCP4	19.784	17.63	42.577	28.453	30.131	4.676	7.574	14.22	7.624	8.153	-1.71363	down	2.96E-15
BACE2	2.567	2.413	3.574	2.026	3.129	0.176	1.279	2.123	0.363	2.66	-1.0538	down	2.22E-07
MX1	1.326	1.437	2.837	1.887	0.579	19.364	1.502	12.96	21.936	2.253	2.845731	up	3.75E-38
SIK1	4.647	4.614	6.964	4.199	7.148	6.818	14.688	14.165	5.574	18.398	1.113008	up	8.40E-08
MAOB	8.732	8.255	8.987	4.828	8.704	14.444	13.781	17.373	12.533	35.844	1.250099	up	6.57E-09
MID1IP1	25.443	24.255	10.061	8.648	11.947	28.246	239.392	160.169	333.219	11.122	3.264355	up	2.49E-48
SLC51AL	1.037	0.838	1.068	1.955	3.483	0.452	0.357	0.562	0.618	0.866	-1.55197	down	5.75E-13
MAP3K15	1.168	1.278	2.428	1.267	1.369	8.403	9.61	7.966	8.484	5.406	2.407603	up	2.21E-29
PDHA2	12.365	12.837	21.312	13.524	16.276	83.531	95.712	84.458	78.601	40.438	2.326269	up	7.09E-28
ADGRG2	0.147	0.183	0.172	0.12	0.105	0.324	3.094	3.171	0.126	0.27	3.255377	up	2.02E-44
GRPR	0.273	0.29	0.054	0.028	0.035	1.019	0.921	0.725	2.62	0.37	3.046626	up	2.13E-25
LOC12110	1.293	1.071	29.826	154.672	238.651	0.567	9.914	2.438	21.032	23.116	-2.89837	down	8.81E-41
LOC10705	27.588	27.701	135.478	188.365	451.459	1.024	22.428	1.025	42.69	54.356	-2.77286	down	2.58E-37
LOC41865	31.573	29.276	14.798	28.114	9.619	6.821	12.477	3.457	7.989	11.436	-1.42643	down	8.93E-13
SLC25A6	931.934	901.092	889.85	906.808	623.308	422.91	495.523	320.503	355.71	528.598	-1.00221	down	4.87E-07
LYGL	0.491	0.321	1.074	0.941	0.252	7.219	0.322	2.305	6.663	1.088	2.512865	up	1.70E-25
RNF149	9.356	9.945	12.002	7.396	9.082	20.837	29.757	26.548	25.509	8.149	1.213363	up	2.63E-09
IL1RL1	1.863	2.123	4.115	1.225	2.715	1.657	18.753	3.84	3.66	3.589	1.386981	up	1.63E-12
IL18R1	0.65	0.527	1.464	0.586	0.686	2.051	0.792	1.337	2.519	1.452	1.057744	up	4.27E-07
FHL2	0.393	0.649	1.044	0.6	0.789	1.45	0.99	1.692	0.785	2.067	1.005999	up	7.86E-06
GRTP1	2.924	3.094	3.3	3.176	4.44	2.686	14.922	15.123	10.118	5.413	1.510688	up	5.09E-13
RAB20	33.363	32.669	54.294	22.269	35.116	11.456	10.876	15.597	15.068	22.699	-1.23119	down	2.72E-09
LOC12110	1.523	2.514	0.799	7.567	0.711	10.243	11.87	11.418	12.406	14.159	2.195731	up	1.03E-21
HS6ST3	1.605	1.497	1.513	1.551	0.979	0.495	0.43	0.293	0.407	0.803	-1.55521	down	7.10E-14
DCT	2.22	2.581	0.314	0.343	0.081	0.627	0.482	0.116	0.927	0	-1.3619	down	9.05E-11
ACOD1	1.389	1.414	1.721	1.603	0.649	2.704	1.941	6.058	3.153	5.159	1.487947	up	1.35E-11
EPSTI1	2.942	3	5.379	3.795	2.701	15.504	2.402	10.742	13.549	3.515	1.359071	up	1.18E-10
LPAR6	4.271	4.735	7.376	9.321	4.387	13.796	20.485	10.492	24.288	8.664	1.368948	up	7.86E-11
PHF11	2.079	2.185	3.607	1.956	2.179	6.808	2.482	6.389	6.318	3.08	1.062296	up	3.76E-07
CKAP2	0.174	0.142	0.531	0.19	0.312	0.666	0.293	0.668	1.696	0.842	1.62282	up	1.97E-12
POSTN	0.715	0.878	4.066	3.799	2.149	6.136	4.625	5.677	2.639	6.716	1.151643	up	3.16E-08
LOC12110	0.429	0.25	1.373	0.466	0.715	0.227	0.197	0.179	0.193	0.586	-1.22314	down	2.53E-08
HSPH1	6.916	5.581	5.768	2.693	6.959	19.795	9.986	10.068	12.184	9.054	1.129581	up	7.82E-08
RASL11A	0.774	0.8	0.714	1.373	0.335	1.249	1.558	2.323	1.903	1.647	1.118165	up	5.68E-06

SKA3	0.118	0.139	0.414	0.253	0.243	0.4	0.346	0.616	1.085	0.606	1.383616	up	4.74E-08
ACAT1	280.209	264.862	449.211	387.065	396.999	143.337	84.728	161.285	123.086	255.053	-1.21231	down	1.69E-09
GUCY1A2	0.961	0.748	1.116	0.745	0.729	0.468	0.76	1.806	1.047	4.861	1.055726	up	2.29E-06
MMP7	0.724	0.148	0.721	0.521	0.093	1.715	4.682	1.647	0.251	4.862	2.572955	up	6.04E-24
FUT4	0.408	0.413	0.414	0.213	0.076	0.389	0.667	0.63	1.233	0.658	1.228177	up	8.65E-06
LOC12110	3.787	3.934	19.381	27.141	67.661	86.921	13.48	17.957	52.202	92.276	1.108386	up	2.14E-07
LOC10174	22.551	17.406	62.447	35.295	34.441	103.174	99.947	96.716	62.981	77.211	1.353991	up	2.39E-11
THRSPB	67.486	71.497	246.224	149.037	160.855	477.592	371.975	204.742	187.805	504.311	1.329108	up	1.02E-10
AQP11	9.811	9.128	6.632	12.259	13.171	5.976	1.64	2.268	2.439	9.856	-1.20115	down	5.23E-10
TSKU	1.372	1.845	1.784	5.612	10.349	3.146	0.651	1.784	0.88	39.799	1.141801	up	2.38E-06
WNT11	0.476	0.429	2.908	1.358	1.013	2.168	0.586	2.316	0.74	7.96	1.154274	up	6.49E-08
IL18BP	0.253	0.332	0.323	0.75	0.573	0.654	0.513	0.615	1.163	2.448	1.271505	up	8.16E-06
LOC10705	0.265	0.304	0.422	0.3	0.613	1.844	0.484	0.603	1.468	1.568	1.645397	up	9.63E-11
LOC12110	0.205	0.323	1.583	0.317	0.099	0.155	0.366	0.855	1.944	3.977	1.528014	up	2.84E-12
ADAM15	2.879	2.467	2.28	10.016	2.523	1.981	0.106	0.636	0.334	1.28	-2.21578	down	3.84E-26
P2RY6	0.615	0.827	0.98	1.685	0.807	2.838	1.882	1.844	2.379	2.762	1.251304	up	1.29E-07
HBE	0	0.145	18.385	0	0.152	0	0	0.09	0	0.146	-6.27686	down	7.93E-57
HBBA	251.682	258.768	745.477	87.668	273.636	49.203	54.295	45.462	35.51	50.132	-2.78521	down	1.46E-35
HBE1	46.694	46.334	122.225	11.314	34.039	3.605	5.186	5.68	4.098	6.994	-3.34949	down	2.04E-47
TAF10	33.266	32.073	29.405	36.015	40.872	15.486	17.657	10.488	20.862	16.682	-1.08016	down	1.07E-07
TPP1	197.54	210.865	179.416	267.845	237.865	65.897	58.107	74.62	69.834	72.553	-1.68109	down	2.37E-16
GIMAP8	0.238	0.252	0.397	0.446	0.174	0.675	0.369	0.81	0.788	1.339	1.398484	up	3.98E-09
SCAP	12.353	12.424	5.509	6.9	7	22.59	21.977	17.272	22.16	19.046	1.22152	up	1.88E-08
ACKR2	1.96	1.81	4.688	2.679	3.108	21.904	8.131	5.899	12.101	22.323	2.303855	up	2.06E-26
CYP8B1	3.027	2.459	7.577	4.087	5.467	41.336	15.074	10.772	22.623	41.512	2.53731	up	2.54E-31
VILL	0.411	0.406	0.348	0.145	0.621	0.792	1.415	0.85	0.471	1.224	1.296973	up	3.84E-09
DLEC1	3.841	3.28	5.44	4.057	2.006	1.195	1.203	0.531	0.923	1.69	-1.74777	down	6.94E-17
ACAA1	153.55	145.844	192.261	192.372	200.895	52.883	37.335	24.236	48.737	64.66	-1.95744	down	4.86E-21
PTCHD3	0.554	0.319	0.091	0.157	0	0.193	0.269	0.643	1.006	0.923	1.43239	up	7.08E-09
APBB1IP	1	1.071	3.128	1.33	4.054	4.678	5.582	5.624	2.835	4.625	1.140931	up	1.86E-08
NELL3	0.006	0	0.06	0.01	0.184	0.443	1.691	0.663	0.032	0.279	3.554241	up	4.23E-47
RSU1	2.554	2.116	3.427	2.316	2.365	10.359	6.536	6.112	8.731	7.206	1.60736	up	4.10E-14
ACBD7	1.497	1.217	3.527	1.728	6.917	0.815	0.546	0.993	0.912	3.05	-1.23622	down	7.16E-09
OLAH	0.248	0.282	1.006	0.256	1.495	0.174	0.099	0.204	0.152	0.623	-1.38898	down	7.24E-10
ABCB1LA	27.561	25.71	20.052	17.421	19.97	7.165	1.791	1.269	3.113	13.98	-2.01872	down	1.68E-23
CYP51A1	48.663	52.847	8.028	7.111	62.297	50.349	60.763	112.371	170.641	21.476	1.215648	up	9.42E-09
CDK6	8.803	8.955	11.844	8.675	7.442	34.017	35.947	46.349	40.159	11.143	1.874171	up	3.84E-19
SAMD9L	2.251	2.167	5.343	4.254	2.407	8.012	1.421	11.008	13.573	4.171	1.217126	up	4.89E-09

COL1A2	1.117	1.323	1.497	2.017	2.457	4.079	3.291	5.305	3.249	4.994	1.313883	up	9.64E-10
LOC39515	2.725	2.891	0.059	0	0	0.03	0.041	6.945	0.029	8.313	1.435497	up	2.12E-08
AGR2	0.451	0.557	0.399	0.371	0.978	0.404	1.014	1.003	0.515	3.951	1.319732	up	1.44E-06
HDAC9	0.261	0.154	0.35	0.245	0.251	0.616	0.746	1.189	0.404	0.657	1.514516	up	8.64E-13
IGF2BP3	0.18	0.133	0.703	0.327	0.257	0.749	0.404	0.701	1.229	0.677	1.230077	up	8.40E-09
FKBP14	2.634	2.28	3.848	1.517	2.599	6.221	7.276	6.125	5.399	2.903	1.116295	up	4.98E-08
SATB1	0.905	0.762	1.234	1.188	0.641	4.143	4.566	3.683	2.868	1.21	1.798842	up	1.66E-17
EOMES	1.415	1.14	1.455	0.616	1.143	1.99	0.884	2.487	4.039	5.485	1.366701	up	6.03E-10
LOC12110	0.908	0.781	0.761	0.785	1.717	0.732	0	0.203	0	0.377	-1.91221	down	4.14E-12
ACAD11	94.158	91.985	133.79	127.816	125.149	49.151	36.127	17.236	44.263	67.871	-1.41628	down	2.95E-12
TGM4	0.156	0.204	0.348	0.885	0.417	2.403	0.263	0.17	0.642	0.738	1.066805	up	1.47E-05
TRANK1	16.125	15.35	13.587	10.786	8.698	83.575	41.981	41.49	52.68	22.169	1.905899	up	5.46E-19
LOC12110	0.547	0.596	2.177	0.599	0.468	0.514	0.154	0.221	0.757	0.494	-1.0339	down	0.000147
INHBA	4.865	4.491	2.44	9.618	6.196	19.707	17.362	13.778	20.26	11.576	1.582226	up	3.06E-13
PDIA4	95.545	97.275	183.461	161.217	159.512	342.908	328.544	349.203	238.596	208.572	1.074421	up	1.43E-07
KCNG2	0.662	0.506	0.242	0.203	0.111	0.859	2.459	1.399	1.352	0.131	1.843493	up	9.06E-18
DCDC2	1.066	0.95	2.24	1.084	1.124	5.142	6.184	5.45	2.281	3.333	1.791562	up	7.29E-18
NRSN1	2.815	2.74	1.361	1.638	0.533	4.188	11.396	10.311	6.999	2.857	1.975516	up	1.13E-19
ID4	2.554	3.021	38.134	8.279	11.246	3.469	4.68	3.648	1.162	12.033	-1.33906	down	1.96E-09
ELOVL2	182.236	179.616	5.847	23.01	45.086	350.735	230.654	467.285	376.55	58.731	1.767714	up	9.29E-16
SYCP2L	87.486	81.32	3.059	10.072	17.064	142.927	128.403	222.498	173.603	33.815	1.817119	up	1.26E-16
TXNDC5	2.63	2.896	5.819	4.605	3.285	10.634	8.469	8.406	7.256	5.301	1.05845	up	2.78E-07
F13A1	0.857	0.865	0.604	1.348	0.503	0.296	0.372	0.341	0.397	0.446	-1.17122	down	4.37E-08
ECI2	248.06	223.024	457.551	325.633	209.669	128.923	77.076	44.085	89.265	77.053	-1.81379	down	6.55E-18
SERPINB1	0.182	0.238	0.512	0.287	0.359	1.015	0.33	0.441	1.66	0.924	1.466622	up	2.32E-10
SERPINB5	0.017	0.022	0.027	0.083	0.069	0.027	0.076	1.159	0.067	0.1	2.684929	up	4.26E-18
LOC42090	1.399	1.359	0.971	1.558	1.669	3.179	3.603	3.358	2.702	1.587	1.052105	up	1.17E-06
CMBL	109.934	119.002	147.194	84.218	175.479	49.215	24.525	11.843	18.376	143.53	-1.36125	down	4.48E-12
SRD5A1	3.159	2.519	9.173	6.425	4.217	1.124	1.375	1.387	1.389	2.422	-1.72708	down	5.84E-16
MOCOS	33.131	32.776	10.736	22.622	11.783	47.915	54.534	57.045	56.422	18.431	1.077429	up	6.93E-07
SEC61B	63.379	70.841	78.921	65.259	55.47	191.319	166.803	230.618	177.893	120.65	1.410094	up	1.94E-11
IFI6	18.124	21.104	45.414	28.49	5.721	1834.018	39.632	297.163	992.608	49.492	4.756573	up	1.21E-87
GLIPR2	0.355	0.398	0.878	0.698	0.456	1.625	1.396	1.875	1.865	1.649	1.592698	up	4.81E-13
ZNF516	0.444	0.453	0.787	0.547	0.696	1.343	1.733	1.898	1.78	1.036	1.410664	up	1.21E-11
FAM69C	0	0	0	0	0	0.601	0.419	0.385	2.34	0.13	9.599913	up	1.60E-59
C18orf63	0.005	0.011	0.022	0.094	0.028	1.964	1.796	1.147	5.567	0.118	6.005046	up	#######
FBXO15	0.747	1.086	0.582	1.092	1.945	0.281	0.448	0.232	0.473	1.097	-1.10555	down	1.92E-07
CIDEA	199.64	214.587	173.713	209.271	309.342	105.49	79.15	57.272	81.96	211.935	-1.04628	down	7.05E-08

MTCL1	0.365	0.243	0.586	0.275	0.43	0.569	0.975	0.566	0.634	2.588	1.486996	up	1.80E-11
MYOM1	1.006	0.83	1.409	0.757	2.832	0.695	0.183	0.172	0.396	1.859	-1.04695	down	1.23E-07
LPIN2	29.822	30.149	58.891	30.487	110.317	17.549	8.439	8.3	29.296	49.508	-1.19912	down	2.91E-09
COLEC12	0.88	0.745	0.979	1.147	1.169	1.811	4.47	3.597	3.421	3.006	1.727561	up	2.19E-15
ABHD3	12.343	10.311	30.543	4.203	14.329	5.756	2.553	2.038	2.65	2.958	-2.1682	down	4.15E-23
LAMA3	0.63	0.857	1.484	1.121	2.179	0.458	0.503	0.739	0.915	0.423	-1.04435	down	1.14E-06
DSC1	0.063	0.069	0.006	0.006	0	2.287	2.764	0.136	2.704	0	5.727738	up	1.60E-89
MCM4	0.945	0.866	1.037	0.742	1.931	2.033	1.252	2.873	3.59	2.526	1.151886	up	7.44E-08
C8orf22	2183.872	2049.309	595.971	1564.483	746.212	449.194	298.74	131.044	778.326	1283.637	-1.27961	down	9.59E-12
FAM110B	2.094	2.421	2.344	2.525	3.412	0.781	0.685	1.136	0.745	2.11	-1.22876	down	1.24E-09
CYP7A1	5.926	9.393	3.053	30.473	24.168	188.93	68.66	71.468	176.197	43.269	2.909244	up	1.93E-38
XKR9	0.272	0.235	0.097	0.395	0.345	1.098	0.384	0.46	0.711	0.817	1.365123	up	5.25E-09
MSC	0.485	0.571	1.7	0.459	0.424	0.196	0.164	0.471	0.115	0.359	-1.47596	down	1.36E-08
GDAP1	0.009	0.029	0.084	0.78	0.108	0.085	0.771	0.053	0.097	2.053	1.593937	up	6.74E-10
FABP4	0.671	0.603	1.803	3.858	0.861	0	0.189	0.356	0.133	1.239	-2.02105	down	5.19E-13
CA13	2.575	2.243	4.942	1.926	2.63	1.47	1.09	0.675	0.561	1.426	-1.45408	down	2.14E-11
ATP6V0D2	0.331	0.185	0.978	0.233	0	0.552	1.488	0.444	1.57	0.396	1.362987	up	3.40E-09
WWP1	17.708	16.534	26.756	14.9	10.589	39.279	47.707	35.113	39.273	22.612	1.088981	up	8.11E-08
PTDSS1	16.268	15.45	18.831	8.89	8.963	33.557	55.652	43.331	39.356	12.514	1.43074	up	2.64E-12
CPQ	22.699	22.404	10.172	11.779	18.934	15.145	34.489	81.848	45.895	12.47	1.142584	up	5.72E-08
NCALD	15.006	14.897	22.578	15.2	31.005	7.843	3.825	5.502	2.83	18.96	-1.34074	down	3.38E-11
RIMS2	0.112	0.099	0.68	0.433	0.142	0.595	0.213	0.787	1.246	0.995	1.384685	up	8.25E-11
DCSTAMP	4.222	3.077	3.015	0.466	0.332	0.793	1.239	0.388	1.404	1.19	-1.1473	down	2.73E-08
EBAG9	42.514	43.319	23.402	20.343	17.986	89.547	91.73	110.904	83.299	21.334	1.427091	up	1.67E-11
SAMD12	1.124	1.049	1.62	2.375	2.304	4.233	4.87	3.604	2.878	2.762	1.114312	up	9.17E-08
SQLE	22.329	24.556	0.467	0.583	24.186	17.793	27.324	51.071	100.714	4.119	1.478791	up	4.86E-12
TSTA3	4.979	5.299	4.769	6.335	5.727	13.135	17.748	16.667	13.251	6.626	1.314395	up	4.60E-10
LOC12111	3.227	3.889	5.916	2.652	5.582	1.712	1.527	2.78	0	1.799	-1.44309	down	1.41E-10
PRORSD1I	6.63	6.325	5.499	4.573	6.769	12.918	18.102	15.936	10.303	10.074	1.17606	up	2.74E-08
CFAP36	2.004	1.618	0.259	0.801	0.482	0.524	0.264	0.197	0.1	0.641	-1.57828	down	3.61E-13
KIAA1841	0.506	0.395	1.208	0.72	0.837	1.893	1.069	1.506	2.204	1.25	1.110602	up	9.66E-08
BUB1	4.253	4.887	8.572	9.029	2.457	32.663	31.021	29.543	30.263	11.047	2.203868	up	5.83E-25
LOC42123	1.271	1.004	1.146	1.936	0.635	0.364	0.382	0.439	0.313	0.32	-1.71793	down	4.60E-16
LOC10174	0.314	0.441	0.501	0.258	1.015	1.122	1.01	0.926	1.989	1.46	1.361684	up	6.90E-08
CLIP4	5.842	5.73	4.468	4.168	2.778	7.426	13.959	6.238	14.11	7.238	1.091006	up	2.52E-07
EHBP1	19.949	18.589	22.644	19.693	24.768	8.485	7.515	5.928	7.621	13.56	-1.29304	down	1.65E-10
WDPCP	0.147	0.144	0.135	0.085	0.177	1.627	0.755	0.207	0.188	0.11	2.06114	up	3.87E-21
UGP2	111.949	111.93	146.823	110.221	109.185	486.032	807.157	304.292	231.618	219.705	1.795722	up	4.73E-18

SLC1A4	0.663	0.798	0.625	0.501	0.447	1.573	1.002	1.507	2.161	1.388	1.32922	up	8.98E-10
JAG1	7.158	6.278	16.697	4.057	4.059	3.502	3.309	2.597	3.942	3.279	-1.20148	down	2.80E-08
RRBP1	20.908	22.272	26.808	27.12	8.192	47.095	69.155	56.66	57.051	31.255	1.310697	up	3.49E-10
GINS1	0.504	0.431	0.617	0.7	1.273	1.468	0.827	1.362	1.318	2.138	1.011804	up	3.69E-05
TLR5	1.701	1.462	1.014	2.244	0.513	0.285	0.454	0.53	0.193	0.439	-1.86418	down	1.70E-19
PTPN14	0.724	0.699	1.214	1.186	0.788	1.803	2.61	2.492	1.38	1.122	1.027858	up	5.72E-07
ABCG8	6.836	6.659	11.368	4.54	4.994	16.97	11.548	17.06	16.207	8.1	1.022594	up	4.58E-07
TTC7A	2.626	3.069	3.554	3.948	3.48	9.603	9.302	7.221	6.956	5.028	1.192067	up	1.21E-08
KCNK17	0.334	0.341	1.401	0.517	0.656	0.8	1.569	1.948	2.045	0.988	1.176511	up	6.67E-09
NFKBIE	1.106	0.82	1.526	1.434	1.085	2.18	2.487	2.623	4.297	3.682	1.353826	up	1.11E-09
EIF2AK2	6.762	6.172	8.032	8.465	7.513	29.652	11.189	19.756	20.865	9.009	1.292	up	1.00E-09
QPCT	0.654	0.652	2.296	1.503	0.514	0.396	0.368	0.643	0.451	0.938	-1.00565	down	1.82E-06
PLD5	0.29	0.361	0.031	0.571	1.529	2.814	0.321	0.247	2.748	0.052	1.150527	up	9.49E-08
GREM2	0.261	0.43	1.984	0.788	0.728	0.546	0.316	0.088	0.041	0.01	-2.06038	down	2.78E-17
SLC35F3	0.254	0.206	0.501	0.338	1.305	0.234	0.109	0.147	0.067	0.238	-1.70543	down	5.32E-12
GNPAT	11.337	11.076	5.99	4.546	8.803	16.358	10.217	31.965	23.469	7.339	1.097498	up	2.54E-07
THBS2	0.071	0.079	0.148	0.158	1.474	1.105	0.233	0.55	1.243	1.021	1.103209	up	3.08E-07
SULT	1527.22	1357.682	2707.046	1942.924	485.372	835.848	809.635	343.058	797.79	867.082	-1.1344	down	3.20E-08
DACT2	0.361	0.382	0.406	0.794	2.125	0.154	0.215	0.251	0.168	0.429	-1.73685	down	2.60E-14
MPC1L	355.379	296.186	600.226	259.974	295.607	155.893	76.926	93.805	133.758	64.275	-1.78444	down	4.68E-17
TBXT	0.389	0.238	0.47	0.402	0.245	0.617	0.183	0.342	1.748	0.866	1.104586	up	2.38E-06
LOC42158	1.671	1.488	1.635	0.86	0.951	1.753	0.091	0.122	0.127	0.635	-1.27416	down	3.97E-09
FNDC1	2.801	2.213	0.846	1.502	0.292	0.331	0.192	0.51	0.335	0.449	-2.07163	down	7.60E-23
ARFGEF3	0.892	0.781	0.814	0.915	0.082	1.538	1.891	3.244	1.097	0.274	1.205996	up	9.80E-09
PERPB	0.023	0.046	0.704	0.134	0.67	0.526	1.361	0.946	0	1.744	1.534228	up	5.89E-11
MAP3K5	3.516	2.933	4.614	2.564	1.944	7.968	9.191	8.567	8.371	3.158	1.258303	up	8.56E-10
SGK1	10.604	9.899	24.114	13.805	25.396	33.986	53.412	55.42	45.261	24.454	1.342303	up	2.29E-11
LOC11253	0.387	0.875	1.289	0.636	1.374	0.426	0.396	0.64	0.334	0.382	-1.06462	down	4.14E-05
HEY2	0.109	0.091	0.21	0.076	0.096	1.063	0.436	1.006	0.761	0.245	2.582503	up	8.98E-24
GJA1	19.079	19.274	11.943	15.972	6.838	72.824	66.245	51.81	63.143	41.254	2.013928	up	2.04E-20
MCM9	1.662	1.497	0.398	0.382	0.229	0.259	0.181	0.303	0.255	0.44	-1.53201	down	3.34E-13
DCBLD1	3.256	3.62	2.803	2.341	1.677	12.214	14.084	18.832	25.398	1.502	2.394312	up	1.86E-28
LOC42174	143.135	116.205	363.465	245.924	407.447	83.574	69.143	52.256	63.24	244.328	-1.31608	down	9.75E-11
METTL24	0.877	1.038	0.1	1.441	0.622	2.44	1.197	0.481	1.8	2.304	1.010737	up	7.02E-05
LOC12111	0.106	0.208	0.201	0.764	0.204	0.792	1.88	2.004	0.315	0.052	1.762337	up	3.27E-14
FHL5	0.057	0.111	0.69	0.362	0.262	0.675	0.559	0.319	0.483	1.063	1.061724	up	4.03E-06
NT5E	0.293	0.453	1.344	0.723	0.527	0.904	1.697	1.353	2.114	2.262	1.317176	up	5.69E-10
PRSS35	1.603	1.477	1.682	1.094	1.724	5.564	5.024	4.065	2.656	2.807	1.407481	up	1.91E-11

ME1	8.539	9.374	9.18	7.501	16.873	88.073	45.52	46.485	45.481	24.372	2.279699	up	2.56E-26
RWDD2A	3.585	3.795	3.749	2.782	5.311	22.445	14.412	13.16	12.631	7.97	1.877004	up	9.15E-19
HMGCLL1	3.515	3.311	3.414	2.888	1.877	1.549	1.791	1.151	0.667	0.947	-1.29668	down	1.91E-09
FAM110C	2.976	2.726	2.712	4.42	5.076	2.824	1.424	0.755	1.046	2.456	-1.07394	down	6.20E-08
CMPK2	8.775	8.064	19.72	12.554	3.339	57.084	7.901	36.548	52.743	11.315	1.658461	up	2.72E-15
RSAD2	1.418	0.976	3.298	2.576	1.341	48.047	0.923	9.027	32.739	1.765	3.266338	up	4.87E-46
MBOAT2	54.307	53.068	2.92	4.222	2.508	75.145	80.345	76.168	125.974	4.794	1.630828	up	1.06E-13
RRM2	0.136	0.204	0.619	0.895	0.879	1.662	0.721	2.11	4.538	2.683	2.097655	up	7.03E-20
ATP6V1C2	0.951	0.739	1.831	1.71	2.036	3.244	3.588	4.11	3.371	2.696	1.226298	up	7.55E-09
PDIA6	57.434	55.471	70.146	63.546	69.986	202.203	155.347	193.89	138.058	116.389	1.34798	up	1.16E-10
TRIB2	3.396	3.099	3.902	2.595	2.973	4.982	6.735	4.559	12.285	5.04	1.073355	up	2.84E-07
VSNL1	18.663	15.883	23.938	16.742	23.218	11.108	6.626	5.213	8.721	7.542	-1.32797	down	1.39E-10
APOB	1455.796	1551.823	478.735	607.197	484.529	2281.361	2575.289	2771.347	3000.189	683.29	1.304972	up	1.27E-09
TMEM214	11.343	11.863	15.822	8.264	9.733	25.076	30.208	37.382	22.301	13.843	1.175508	up	8.64E-09
DTNB	12.246	11.435	2.323	2.942	2.779	1.849	4.553	2.258	4.403	2.345	-1.0417	down	1.13E-07
KIF3C	1.136	1.361	1.274	1.236	1.068	5.073	4.121	5.085	2.147	1.098	1.5276	up	5.72E-13
HADHA	118.676	122.57	136.401	163.422	167.546	90.287	56.265	65.784	66.753	45.929	-1.12447	down	3.14E-08
HADHB	362.649	336.843	399.756	407.366	334.114	195.958	153.603	122.618	134.355	120.863	-1.33946	down	6.30E-11
LOC12111	6.675	7.37	25.456	52.844	35.568	5.046	7.681	5.218	5.529	7.71	-2.03611	down	1.34E-21
C3H8ORF8	4.545	4.435	4.744	7.777	3.666	20.105	4.908	17.089	19.809	6.353	1.439411	up	2.89E-11
PBK	0.135	0.159	0.086	0.133	0.138	0.825	0.363	0.947	1.535	0.744	2.751952	up	3.25E-22
LOC12111	1.558	1.463	1.781	1.265	1.111	3.111	2.009	4.685	6.743	4.571	1.556225	up	5.59E-13
CLU	11.367	12.74	10.805	12.473	30.681	29.377	16.643	63.952	84.649	36.714	1.567156	up	1.45E-13
SOX7	3.242	3.065	2.072	1.882	2.475	23.132	39.68	39.435	32.546	2.99	3.4349	up	1.49E-52
TDH	348.897	331.718	393.93	562	483.821	186.83	238.998	85.949	159.616	339.124	-1.06922	down	6.69E-08
BLK	0.486	0.712	1.101	0.58	0.771	1.661	2.382	4.425	1.432	1.697	1.66643	up	1.27E-14
FDFT1	37.273	38.699	4.171	6.444	60.069	47.952	96.36	127.883	195.111	14.166	1.714982	up	4.61E-16
AvBD13	65.643	71.449	112.278	364.193	337.728	25.396	70.825	12.316	23.056	158.266	-1.71452	down	1.90E-17
MCM3	0.205	0.191	0.262	0.324	0.466	0.648	0.559	1.201	1.585	1.175	1.831966	up	3.73E-16
CRISP2	0.086	0.075	2.056	1.533	0.56	0.046	0.258	0.035	0.091	0.707	-1.9178	down	2.27E-13
RHAG	1.114	1.21	2.461	0.895	1.691	0.245	0.313	0.154	0.1	0.474	-2.51435	down	1.22E-23
CYP2AC1	632.367	539.155	1236.022	975.454	1033.102	313.781	178.289	56.293	198.898	794.734	-1.51797	down	4.25E-14
SUPT3H	5.372	5.091	5.659	4.89	14.773	3.166	3.494	2.454	3.431	4.5	-1.06978	down	2.29E-07
RCAN2	2.931	2.809	2.735	5.073	3.064	1.396	0.491	1.113	0.673	2.991	-1.31712	down	1.55E-11
ADGRF5	2.642	2.89	2.883	2.995	1.282	1.365	0.613	0.735	1.289	0.918	-1.36629	down	1.99E-11
TNFRSF21	11.611	11.588	13.062	11.351	13.154	24.333	30.847	32.524	32.227	15.387	1.154952	up	2.38E-08
ARR3	1.942	1.912	0.361	1.283	1.343	0.278	0.184	0.264	0.23	0.394	-2.33697	down	4.87E-24
KIF4B	0.161	0.146	0.335	0.14	0.257	1.075	0.382	0.857	1.706	1.005	2.268437	up	5.92E-23

LOC42215	10.398	10.186	13.196	10.574	8.087	24.594	25.848	23.877	22.218	12.423	1.05496	up	3.12E-07
CCNB3	0.287	0.137	0.458	0.193	0.295	0.906	0.382	0.84	2.212	1.442	2.073384	up	1.15E-16
GPC3	0.465	0.397	0.123	0.324	0.362	0.489	0.999	1.41	1.35	0.361	1.460996	up	4.78E-10
LOC12111	1.202	1.101	1.287	0.539	0.359	0.247	0.418	0.464	0.173	0.603	-1.23411	down	1.18E-07
XKRX	2.891	2.664	4.81	3.368	3.8	7.501	9.043	11.567	10.052	7.137	1.369186	up	3.54E-11
NOX1	0.261	0.427	0.984	0.355	0.413	0.826	0.7	1.468	0.858	1.178	1.042157	up	9.96E-07
SLC16A2	78.76	83.669	98.773	104.677	105.799	42.405	33.286	36.385	34.526	65.869	-1.15052	down	8.33E-09
LOC12111	1.993	1.467	3.592	1.296	2.075	4.864	2.116	4.816	9.787	12.956	1.727973	up	2.98E-15
SH2D1A	1.818	1.263	2.994	1.244	1.728	5.026	2.184	4.149	9.596	11.843	1.857519	up	7.69E-18
IDS	14.08	11.138	9.806	16.463	11.422	7.015	4.413	4.849	5.538	9.461	-1.00809	down	2.06E-07
LOC10085	0.057	0	0.466	0.155	0.136	0.168	0	2.703	0.015	0.074	1.856097	up	5.47E-15
APELA	35.455	33.235	49.143	85.758	30.52	177.222	158.532	100.182	191.317	87.355	1.609939	up	4.12E-14
MSMO1	201.642	197.348	11.709	32.025	267.106	145.576	207.196	498.275	687.512	56.764	1.168297	up	3.51E-08
LOC10705	17.215	17.186	21.674	18.042	33.892	1.238	1.447	1.038	2.974	3.647	-3.38366	down	7.25E-52
DDX60	2.713	2.607	4.405	23.13	14.791	56.199	8.253	55.698	85.598	6.147	2.152805	up	5.35E-23
PALLD	2.498	2.632	3.731	3.289	3.084	5.45	11.3	7.032	3.411	3.949	1.031325	up	3.44E-07
TBC1D9	0.493	0.451	0.835	0.353	1.96	2.861	2.027	0.521	2.334	2.443	1.314656	up	3.58E-10
SMIM18	0	0.34	0.237	0.305	0	0.419	0.167	0.36	1.117	0.073	1.271279	up	0.000807
CXCL13	0	0	0.275	0.17	0.071	0.223	0.078	0.042	0.656	1.158	2.053011	up	1.55E-06
CXCL13L3	0	0	0.136	0.07	0.088	0.483	0	0.156	1.762	0.844	3.442222	up	1.92E-13
LOC42251	4.013	4.053	11.026	7.756	3.707	28.057	3.977	29.22	29.873	4.204	1.641377	up	3.03E-15
ETNPPL	11.28	12.171	27.882	108.246	165.325	7.637	16.164	13.953	9.97	72.075	-1.43936	down	2.65E-13
ARHGEF38	1.002	0.876	0.197	0.258	0.592	0.265	0.227	0.272	0.225	0.465	-1.00592	down	8.15E-07
LOC10705	2.448	2.171	1.971	2.159	1.051	4.815	4.431	4.353	5.28	2.136	1.100173	up	2.04E-07
ACSL1	167.114	151.745	183.9	262.414	249.984	49.58	20.779	21.227	22.338	119.277	-2.12203	down	2.59E-25
AREG	1.735	1.123	8.198	0.737	0.521	0.33	1.688	0.603	0.447	0.692	-1.71016	down	2.12E-12
PRDM8	0	0.009	0.041	0.032	0	0	0	0	0	1.142	3.720706	up	6.18E-22
SPP1	7.596	7.409	16.908	5.958	9.185	13.028	23.213	23.824	10.79	31.783	1.125031	up	4.00E-08
PLACL2	2.75	3.512	6.256	7.048	3.026	10.082	4.637	23.341	13.156	11.55	1.473967	up	1.82E-11
GPAT3	7.264	7.386	8.397	9.107	17.669	5.735	2.325	2.833	2.541	7.16	-1.27438	down	2.48E-10
DUSP4	0.599	1.122	0.629	0.47	1.118	2.195	2.662	2.41	1.938	0.778	1.340902	up	3.11E-09
PPP1R3B	34.493	34.88	47.797	51.492	57.78	107.722	158.798	109.756	75.471	42.173	1.125119	up	2.92E-08
SHROOM3	0.496	0.407	1.226	0.995	0.284	0.177	0.227	0.133	0.18	0.35	-1.67074	down	1.67E-14
LOC10085	1.014	1.366	0.19	1.03	0.368	0.241	0.067	0.217	0.378	0.882	-1.15027	down	6.31E-06
NPFFR2	0.86	1.092	0.498	0.707	0.482	0.315	0.205	0.221	0.64	0.206	-1.19468	down	6.50E-07
SULT1B	30.586	33.569	49.609	13.958	87.131	3.56	3.986	20.187	7.905	24.301	-1.8417	down	1.94E-18
CCNA2	1.02	1.09	1.575	1.403	1.133	3.155	2.34	2.133	3.508	2.277	1.107792	up	4.40E-07
FABP2	0	0	0	1.883	0.102	0	0.224	0	0	0.098	-2.60541	down	1.50E-07

SEC24D	8.745	8.721	9.665	9.445	8.6	22.487	23.138	20.934	21.969	10.183	1.127568	up	5.75E-08
PITX2	0.311	0.445	0.449	0.399	0.359	0.548	0.502	0.612	1.798	0.536	1.02363	up	1.92E-05
ENPEP	8.84	8.815	9.553	7.139	5.576	14.781	21.618	23.188	13.093	8.321	1.020628	up	7.04E-07
CASP6	17.864	14.593	13.318	12.265	6.664	28.714	32.818	28.38	44.62	10.902	1.168376	up	2.72E-08
TLR3	3.55	3.085	3.735	4.778	5.225	11.463	2.6	8.449	11.843	6.44	1.001557	up	3.36E-06
FAT1	0.372	0.457	0.678	0.415	0.456	2.905	3.13	2.965	0.694	1.932	2.287124	up	1.30E-26
PDGFRL	1.353	1.265	4.402	0.842	1.012	0.577	0.565	0.105	0.153	0.457	-2.25354	down	7.10E-21
FAM114A1	3.049	2.595	2.04	3.165	2.268	7.151	7.769	6.716	6.223	3.363	1.250806	up	4.63E-09
CCKAR	0.033	0.011	0.106	0	0	0.175	7.231	2.233	0.593	0	6.045381	up	6.09E-85
RBPJ	16.543	15.854	18.495	3.857	20.887	1.792	1.803	2.345	2.756	2.957	-2.69785	down	2.77E-35
PPARGC1A	6.235	5.475	3.024	3.071	5.326	0.53	0.367	0.637	0.538	5.35	-1.63929	down	3.21E-17
PROM1	2.899	2.262	1.806	3.563	2.459	1.298	0.77	1.851	0.747	1.475	-1.08013	down	4.98E-08
FGFBP2	1.379	0.822	0.581	1.2	1.166	0.196	0.365	0.443	0.192	0.32	-1.76039	down	2.55E-11
EVC2	0.867	0.646	0.628	0.455	0.383	2.087	1.103	0.843	1.472	1.507	1.233607	up	2.23E-08
ZFYVE28	4.941	4.073	3.344	1.671	1.418	0.965	1.213	1.193	1.04	3.017	-1.05578	down	7.54E-08
HAUS3	0.566	0.536	0.789	0.756	0.582	1.802	1.739	1.858	1.808	1.116	1.36465	up	3.10E-10
FGFR3	4.415	4.249	5.699	4.139	10.984	12.818	19.418	14.956	16.76	8.628	1.299397	up	1.77E-10
SLBP	0.132	0.245	0.946	0.56	0.226	2.482	0.346	1.627	3.706	1.084	2.129478	up	3.72E-20
SPON2	0.232	0.346	0.632	3.372	0.765	6.056	1.414	2.949	2.356	14.643	2.357236	up	1.68E-23
SMYD1	1.567	1.467	0.174	1.386	0.571	1.696	8.744	5.618	9.779	2.699	2.4648	up	3.16E-27
SMOX	2.88	2.474	1.268	1.19	1.487	1.998	5.264	3.229	7.103	1.554	1.041647	up	1.25E-06
LOC10705	3.023	3.262	3.794	3.719	4.613	7.752	6.663	7.745	4.986	10.924	1.047884	up	1.23E-06
GIF	25.405	23.043	65.767	15.246	42.004	64.002	72.876	58.816	53.387	120.744	1.108906	up	3.70E-08
LOC10705	0.718	0.515	1.035	0.787	0.738	1.489	1.692	3.069	1.407	1.786	1.314769	up	1.19E-08
CD6	0.451	0.307	0.719	0.143	0.41	0.7	0.41	0.537	1.414	1.026	1.007777	up	1.12E-05
TCIRG1	11.835	12.294	15.393	16.677	12.762	25.894	36.006	33.754	25.276	21.836	1.049746	up	4.46E-07
ALDH3B2	0.249	0.296	0.459	0.307	0.352	0.779	0.631	0.529	2.206	1.213	1.684921	up	5.75E-13
GPX2	0.167	0.073	0.619	0.046	0.57	0.537	0.562	0.471	0.088	1.641	1.158617	up	0.000123
SPTB	1.182	1.253	2.213	0.42	1.11	0.284	0.353	0.357	0.325	0.675	-1.62903	down	3.89E-14
CDHR5	0.01	0.051	0.185	0.255	0.04	1.667	0.098	3.095	0.591	1.856	3.743293	up	1.20E-49
BUB1B	0.043	0.084	0.136	0.237	0.11	0.499	0.24	0.842	1.368	0.652	2.551741	up	1.14E-24
KNSTRN	0.253	0.165	0.46	0.831	0.89	1.077	0.406	1.532	1.885	2.099	1.42745	up	9.58E-09
B4GALNT4	0.042	0.094	0.181	0.159	0.182	0.646	0.389	1.411	1.468	0.332	2.680721	up	1.29E-29
LOC77061	77.416	82.614	158.739	190.458	73.271	439.566	131.657	442.479	589.078	256.301	1.674256	up	4.18E-15
DHCR7	20.697	21.731	2.016	2.903	33.831	31.784	30.996	49.22	94.145	12.462	1.429123	up	1.63E-11
E2F8	0.192	0.203	0.289	0.412	0.164	0.715	0.432	0.777	1.867	0.989	1.919382	up	3.49E-16
RCN1	5.229	5.745	5.444	5.877	7.058	15.927	18.398	16.116	12.329	8.965	1.289024	up	7.31E-10
PRRG4	1.373	1.063	4.372	1.151	2.014	3.976	8.741	6.718	4.056	2.292	1.369877	up	3.51E-12

MICAL2	0.179	0.201	0.409	0.404	0.156	0.23	1.166	0.896	0.614	0.808	1.457687	up	4.43E-12
AMPD3	1.358	1.168	1.381	0.697	1.199	0.394	0.536	0.364	0.702	0.78	-1.06244	down	3.38E-07
PDE3B	12.752	11.286	18.888	14.52	16.245	4.73	3.587	5.18	4.573	11.03	-1.34032	down	3.74E-11
ABCC8	0.848	1.018	0.962	0.608	1.227	0.324	0.38	0.476	0.264	0.293	-1.42206	down	3.19E-11
SAA	18.068	17.014	34.513	38.923	20.811	88.083	49.259	34.038	136.008	41.123	1.430123	up	1.24E-11
KCNQ1	1.006	0.859	0.601	0.536	0.536	1.802	3.161	1.174	2.365	2.579	1.645696	up	7.36E-14
TRPM5	0.198	0.373	0.151	0.965	0.144	30.656	17.517	7.382	19.624	13.613	5.595873	up	#######
CD81	133.864	112.47	84.628	149.555	110.353	277.415	293.727	265.344	220.968	167.414	1.051707	up	8.05E-07
CTSD	96.128	125.637	250.72	233.262	767.082	93.573	80.509	139.451	120.841	168.931	-1.28762	down	6.15E-10
DUSP8	0.615	0.733	1.304	0.308	0.427	1.848	2.546	2.022	3.122	0.365	1.546458	up	2.85E-14
PNPLA2	44.613	45.171	100.424	37.369	99.724	17.98	16.023	14.123	21.69	42.623	-1.54143	down	1.48E-13
RNH1	14.679	15.225	17.425	14.465	15.323	37.386	32.234	37.499	31.845	22.622	1.067132	up	3.11E-07
CHKA	20.577	15.45	22.142	9.008	3.506	62.428	98.625	112.326	55.35	12.814	2.272551	up	5.39E-27
TESMIN	0.48	0.358	1.521	0.412	0.316	0.36	0.221	0.203	0.228	0.344	-1.18387	down	7.11E-08
CPT1A	107.933	94.737	63.556	70.728	127.52	19.34	13.443	14.415	16.447	43.832	-2.11152	down	1.04E-24
LOC42885	0.182	0.155	0.015	0.045	0	0.573	0.041	1.691	1.038	0.072	3.088729	up	3.13E-27
SMTNL1	0.484	0.481	0.17	1.098	0.795	0.203	0.087	0.329	0.337	0.382	-1.17529	down	3.32E-07
PGR2/3	0.309	0.121	0.147	1.826	0.127	0.05	0.278	0.037	0	0.304	-1.91117	down	1.49E-08
SMIM38	1.844	1.327	0.147	1.819	0.474	0	0	0	0	0	-10.1334	down	2.21E-31
FGF19	0.264	0.104	0.196	0.174	0.036	3.096	12.853	11.022	3.986	1.076	5.362017	up	1.86E-91
TSPAN18	1.105	1.835	2.335	1.816	3.187	0.994	0.717	1.237	0.706	1.005	-1.14062	down	9.18E-07
MDK	0.867	1.267	3.207	3.519	3.744	0.637	0.917	1.206	0.908	1.332	-1.33301	down	1.92E-09
NUSAP1	0.097	0.255	0.245	0.373	0.183	0.641	0.255	0.669	1.553	0.943	1.811975	up	3.37E-13
PLA2G4B	1.367	0.994	1.144	1.118	0.273	3.281	1.11	3.484	0.931	2.064	1.149867	up	1.70E-07
RDH11	4.659	5.179	3.221	1.487	5.496	3.972	3.964	15.183	19.512	2.699	1.177239	up	2.08E-08
TTC6	0.807	0.437	2.69	0.156	0.257	0.172	0.042	0.02	0.154	0.306	-2.63831	down	7.64E-30
CLEC14A	2.212	2.435	1.824	0.706	2.085	0.357	0.849	0.615	0.432	1.462	-1.3168	down	1.92E-09
ACSS1B	42.926	41.52	46.602	47.98	76.447	2.805	4.021	12.79	9.115	14.894	-2.54982	down	6.83E-33
RPS6KL1	1.015	1.189	1.327	1.382	1.898	4.312	2.47	2.48	2.733	3.882	1.220395	up	2.52E-08
GALC	0.376	0.449	0.833	0.893	1.745	0.316	0.276	0.287	0.291	0.572	-1.29979	down	2.44E-09
TRIP11	8.883	7.779	10.737	6.351	6.682	30.36	40.943	18.721	28.163	8.746	1.650376	up	1.24E-15
LOC10704	0.496	0.568	1.415	0.464	0.156	0.111	0.155	0.033	0.251	0.027	-2.41504	down	4.39E-20
LOC12111	3.707	3.001	0.024	3.645	0	0	0	3.463	0	0	-1.58191	down	8.59E-13
DIO3	1.688	1.42	0.299	0.352	1.486	0.259	0.603	0.91	1.039	27.018	2.506568	up	8.87E-25
ANKRD9	2.95	3.165	5.967	2.546	9.757	20.386	26.768	32.056	22.804	13.637	2.245478	up	2.25E-26
AMN	2.966	4.119	6.067	4.949	11.505	1.14	0.59	0.415	0.776	1.102	-2.878	down	1.55E-36
C14orf180	0.085	0.103	0	0.07	0	0.771	0.082	0.081	1.091	0.095	3.014328	up	5.43E-25
DHRS7	868.149	797.527	1003.922	724.93	1188.741	378.054	337.342	223.957	409.228	631.561	-1.21077	down	2.05E-09

DLGAP5	0.257	0.168	0.303	0.322	0.349	1.411	0.671	0.943	2.533	2.841	2.581533	up	2.93E-28
CDKL1	2.515	2.163	2.554	3.303	3.6	1.581	1.002	1.669	1.426	1.269	-1.02428	down	1.08E-06
LOC10705	0.159	0.295	0.232	0.327	0.436	1.261	3.547	1.398	1.951	0.705	2.608419	up	5.10E-26
PTGER2	0.072	0.213	0.202	0.178	0.335	0.584	3.215	0.373	1.059	0.25	2.448559	up	6.38E-19
LRFN5	0.795	1.132	3.481	0.579	0.724	2.057	3.877	4.648	4.148	2.198	1.334163	up	2.02E-11
LOC76841	0.355	0.522	0.8	0.34	0.516	1.084	0.498	1.29	1.298	1.049	1.041463	up	3.08E-06
GRID1	0.591	0.53	1.061	0.147	0.01	0.038	0.086	0.131	0.219	0.023	-2.22321	down	2.31E-22
SNCG	0.037	0.016	0.07	0.041	1.68	0.01	0.042	0.076	0.02	0.049	-3.19432	down	4.86E-25
ADIRF	0.06	0	0.048	0.147	0.123	0.674	0.268	1.158	0.566	0.176	2.894026	up	6.97E-14
DNAJC12	47.04	41.616	64.729	49.829	55.959	21.112	11.283	7.219	24.662	45.233	-1.24283	down	4.13E-10
ASAH2	0.194	0.184	2.521	0.231	0.199	0.34	0.415	0.235	0.237	0.416	-1.01654	down	0.000177
PRKG1	0.334	0.412	0.524	1.475	0.744	0.571	4.856	0.75	1.847	0.566	1.298618	up	4.36E-10
HKDC1	0.042	0.172	0.528	0.207	0.076	123.8	227.712	75.909	194.624	19.786	9.283417	up	#######
CYP2C18	1704.444	1566.283	682.717	855.24	1306.675	736.795	56.517	48.535	173.916	1298.6	-1.40182	down	1.08E-13
TLL2	1.238	1.179	1.329	0.949	1.964	0.343	0.267	0.476	0.551	0.692	-1.51358	down	5.76E-13
TMEM150	0.686	0.742	0.967	1.236	0.623	3.128	3.204	2.688	1.127	1.508	1.452981	up	1.40E-10
SCD	143.146	170.585	16.981	51.148	16.442	982.484	768.767	811.109	783.53	360.397	3.218024	up	3.61E-45
CYP2C23b	588.344	527.607	295.271	352.718	570.35	367.367	141.174	106.077	106.49	301.246	-1.19109	down	9.96E-10
CYP2C23a	1174.55	1044.819	1141.807	758.226	1003.909	589.727	294.127	258.121	307.369	677.556	-1.26832	down	2.16E-10
MSMB	5.047	4.939	8.814	1.882	4.879	24.382	15.449	3.446	11.979	22.656	1.607712	up	4.26E-14
FRMPD2	0.119	0.243	0.231	0.219	0.479	0.56	0.027	1.067	1.565	1.509	1.868689	up	8.03E-17
ANKRD22	0.151	0.049	0.06	0.248	0.039	0.213	0.127	2.515	0.119	0.074	2.467487	up	1.43E-15
ACTA2	7.16	6.919	10.318	9.822	12.616	27.601	22.897	18.46	17.561	25.718	1.2608	up	2.09E-09
IFIT5	26.904	24.599	64.378	48.686	21.755	178.995	10.596	103.756	190.716	19.47	1.434264	up	5.64E-12
PANK1	87.26	75.486	129.212	87.423	116.202	48.687	48.771	18.683	25.531	103.381	-1.01602	down	3.45E-07
CRTAC1	6.912	8.008	0.079	0.244	0.017	1.117	2.541	0	0.026	0.081	-2.01759	down	1.33E-20
PI4K2A	22.469	22.979	17.273	12.637	18.764	10.59	8.324	6.777	8.699	10.325	-1.07369	down	8.16E-08
HOGA1	4.35	4.49	2.699	2.135	5.657	0.683	0.476	0.257	0.268	1.114	-2.78625	down	1.49E-33
TLX1	0.975	1.072	0.882	0.947	0.046	0	0.1	0.081	0.035	0.262	-3.02333	down	1.40E-20
PDZD7	1.084	0.786	1.142	1.255	0.335	0.282	0.616	0.474	0.12	0.23	-1.41556	down	3.51E-10
CYP17A1	1.837	2.736	1.617	3.953	6.331	0.565	0.19	0.704	0.325	1.215	-2.45567	down	7.53E-28
STN1	0.283	0.516	0.439	0.869	1.469	2.066	1.39	2.086	3.795	1.879	1.647767	up	1.34E-13
GSTO2	5.481	5.978	4.51	2.385	10.692	2.183	1.279	1.214	1.858	3.976	-1.46614	down	5.10E-13
GPAM	50.126	44.294	35.149	35.867	21.312	221.085	252.117	278.979	238.062	38.815	2.462128	up	5.21E-30
ACSL5	62.732	60.198	76.617	65.781	50.458	123.054	152.975	211.647	128.487	68.879	1.117233	up	5.68E-08
LOC10175	0.605	0.138	0.27	0.482	0.762	1.374	4.82	1.827	0.964	9.228	3.009694	up	1.26E-38
FAM196A	0.873	1.004	4.073	1.217	0.36	0.159	0.315	1.024	0.222	0.907	-1.51687	down	3.38E-12
MKI67	0.168	0.176	0.41	0.273	0.327	1.065	0.737	0.98	2.425	2.208	2.448874	up	4.75E-28

ALDH18A1	4.614	5.091	9.894	5.147	5.39	16.706	12.094	14.154	15.828	9.185	1.173213	up	9.10E-09
COL3A1	4.995	6.107	6.011	9.004	10.436	15.695	12.615	20.318	13.543	17.062	1.116005	up	1.68E-07
ABCA12	5.11	4.197	11.634	1.146	27.632	1.161	1.529	0.396	0.257	2.995	-2.97071	down	6.05E-41
ATIC	4205.008	4235.959	2567.995	3001.741	2519.42	835.202	1992.128	722.519	626.515	1696.087	-1.49306	down	1.97E-13
LOC12111	5.572	5.416	23.484	11.677	68.075	4.204	0.654	0.868	0.956	31.978	-1.56283	down	5.78E-15
AHR2	5.188	4.649	7.267	7.84	9.885	1.717	0.77	0.871	0.709	8.162	-1.5096	down	1.66E-14
AHR1B	4.608	3.848	6.309	2.694	3.261	0.219	0.474	0.352	0.296	2.354	-2.48577	down	6.69E-31
SLC19A1	67.212	62.132	29.781	38.601	23.339	20.828	17.689	20.437	20.528	23.098	-1.10768	down	1.30E-08
COL6A1	3.225	4.368	4.751	4.444	5.678	8.665	8.856	9.497	7.863	10.518	1.014755	up	1.56E-06
COL6A2	3.344	4.803	5.366	4.699	6.948	15.462	22.203	17.609	21.925	9.735	1.788585	up	1.30E-17
STAT1	19.896	18.228	25.649	25.151	15.937	83.942	22.932	65.174	116.522	36.136	1.630609	up	1.91E-14
NABP1	6.99	5.47	11.952	4.284	6.75	20.169	20.97	22.443	10.686	14.349	1.321839	up	6.59E-11
LOC76858	0.209	0.219	1.432	0.791	2.062	0.304	0	0.279	0.199	0.082	-2.44075	down	4.26E-16
COQ10B	31.709	32.659	22.38	37.282	58.093	6.194	3.661	4.16	5.803	58.642	-1.21483	down	7.09E-11
ICOS	1.272	0.542	1.615	0.572	0.357	1.945	1.322	1.36	3.541	2.306	1.264109	up	3.43E-09
CTLA4	0.131	0.315	0.278	0.036	0	0.599	0.295	0.186	2.905	1.422	2.82263	up	2.46E-20
DNAJC10	8.702	8.265	9.806	6.087	7.538	19.392	18.022	18.247	15.954	10.94	1.030981	up	6.24E-07
ZNF385B	2.603	1.709	5.413	2.57	2.071	6.895	6.596	5.673	6.165	5.23	1.088673	up	7.05E-08
PDE11A	19.234	16.791	0.348	4.895	0.061	25.726	23.091	46.609	26.455	0.521	1.566285	up	1.36E-12
CDCA7	0.208	0.484	1.145	0.514	0.696	1.345	0.738	2.041	1.781	1.561	1.291546	up	1.29E-09
RAPGEF4	21.935	20.538	17.359	11.14	36.575	3.829	2.201	4.268	6.824	14.15	-1.78186	down	1.97E-18
SPC25	0.417	0.448	0.332	0.441	0.949	1.177	1.005	1.246	2.451	1.703	1.549464	up	1.45E-10
IFIH1	5.446	4.954	7.47	6.273	6.549	21.422	5.618	12.07	17.778	6.909	1.055504	up	5.30E-07
DPP4	4.234	3.722	3.601	5.071	9.228	14.731	7.329	10.947	17.148	12.483	1.276375	up	2.44E-09
SLC4A10	0.187	0.138	0.278	0.215	0.281	1.345	0.378	0.778	0.926	0.742	1.91869	up	1.54E-17
ITGB6	0.971	0.877	0.5	0.447	0.438	1.945	6.7	5.226	9.802	0.183	2.88148	up	8.21E-39
LOC10704	0.24	0.24	0.524	0.572	1.416	0.153	0.299	0.391	0.26	0.324	-1.06549	down	7.85E-06
LOC42419	0.762	0.715	1.71	0.465	1.364	0.079	0.154	0.095	0.046	0.154	-3.23577	down	9.71E-32
MNR2	0.339	0.423	1.029	0.304	0.379	2.01	1.142	3.21	2.07	0.91	1.914745	up	1.47E-16
RPL37A	233.691	219.179	201.042	302.53	284.505	41.527	22.562	128.75	173.861	37.024	-1.61999	down	4.19E-15
ARHGEF1	0.476	0.488	0.145	0.235	0.179	2.829	0.866	1.747	2.788	0.79	2.562282	up	2.27E-28
EAF2	6.465	6.932	2.603	3.063	2.765	14.184	5.703	10.522	24.94	4.215	1.448051	up	3.08E-11
KIF5C	0.354	0.314	0.664	0.557	0.308	0.604	0.681	1.095	1.438	0.916	1.105768	up	2.81E-07
NMI	9.6	9.662	15.516	11.147	9.809	33.377	13.953	26.834	32.34	14.375	1.116864	up	8.31E-08
UPP2	40.223	37.093	61.261	47.83	61.272	15.19	43.853	15.792	15.699	19.257	-1.17368	down	3.95E-08
SELP	0.522	0.502	0.636	0.581	0.621	0.9	1.197	0.59	1.296	1.806	1.015018	up	6.46E-06
LOC10705	0.051	0.033	0.067	0.083	0.104	1.051	0.095	0.103	1.915	0.117	3.260052	up	6.44E-30
FASLG	0.394	0.358	0.569	0.236	0.405	1.525	0.789	0.62	1.136	0.507	1.219981	up	1.32E-08

SUCO	6.512	6.015	8.369	5.144	6.028	35.603	34.034	28.632	34.746	5.236	2.107911	up	1.43E-23
RGS8	6.23	6.334	1.454	1.004	4.903	1.91	0.824	0.811	0.971	0.826	-1.89814	down	3.63E-20
RGSL1	1.999	2.292	0.7	0.614	1.794	0.531	0.692	0.413	1.051	0.444	-1.23938	down	3.97E-09
ABL2	2.044	1.783	3.941	1.973	2.067	8.954	13.23	20.465	7.024	1.995	2.129035	up	1.35E-24
TOR3A	22.63	20.597	13.745	7.774	1.6	82.31	95.538	109.014	52.33	4.706	2.373812	up	3.66E-28
ASTN1	0.034	0.059	0.103	0.379	0.081	0.014	0.493	0.832	0.405	1.081	2.09808	up	1.87E-18
KIAA0040	27.146	26.189	17.705	19.883	28.757	13.307	7.957	8.668	8.787	18.655	-1.06065	down	5.19E-08
SLC30A7	10.54	9.781	12.503	9.39	9.728	29.065	22.837	22.273	22.929	15.114	1.111256	up	9.69E-08
LRRC39	3.84	3.319	3.018	3.376	3.004	5.999	6.856	8.323	11.042	3.029	1.08991	up	2.35E-07
TRMT13	7.791	7.305	6.02	7.143	6.554	12.545	17.336	18.951	22.442	6.539	1.160271	up	3.70E-08
CLCA1	0.1	0.033	0.03	0.164	0.026	0	0.056	2.143	0.148	0.185	2.825092	up	1.32E-25
LPAR3	0.723	0.746	3.989	2.287	2.613	6.481	2.565	3.237	4.342	4.967	1.059389	up	1.85E-07
SSX2IP	2.355	1.78	1.613	1.374	0.639	5.076	6.832	4.062	6.004	0.421	1.528255	up	5.93E-13
VTG2	4938.704	5884.15	43.669	1565.017	4.114	8570.167	12506.41	11995.55	10862.04	974.724	1.852519	up	4.26E-17
LOC12111	3.51	7.943	0	1.355	0	6.521	4.775	12.371	15.713	0.038	1.621429	up	1.01E-12
VTG3	625.379	748.039	2.05	89.471	0.449	1541.929	2148.907	1800.128	1573.222	43.115	2.278015	up	6.82E-25
SPATA1	21.159	19.818	12.843	23.628	2.939	26.63	47.931	33.57	56.874	11.853	1.137508	up	1.09E-07
THAP10	0.586	0.516	0.304	0.223	0.166	0.78	0.744	0.726	1.195	0.586	1.164929	up	3.65E-07
VTG1	2389.735	3147.829	3.494	185.475	1.276	4414.393	5833.7	6461.808	6133.076	6.858	1.996128	up	1.43E-19
ARTN	0.496	0.4	0.669	0.361	1.137	0.4	0.15	0.312	0.06	0.583	-1.02275	down	1.95E-05
RBP	330.724	399.221	17.878	55.352	2.696	813.463	904.042	1105.306	1311.58	29.383	2.369264	up	1.31E-26
FAAH	128.693	126.67	82.223	114.864	123.711	54.381	39.15	47.339	50.98	65.285	-1.16393	down	3.63E-09
CYP4B7	400.359	358.574	324.755	325.616	466.218	115.443	101.221	32.683	127.013	348.611	-1.37129	down	3.01E-12
PDZK1IP1	0.576	1.131	1.119	0.385	1.665	1.54	1.175	2.316	0.912	4.119	1.044385	up	9.46E-06
PODN	2.283	1.903	5.682	1.607	1.414	0.916	0.837	0.976	0.868	1.019	-1.48042	down	7.81E-12
TCEANC2	1.101	0.955	1.181	0.826	1.548	1.852	2.233	2.35	2.688	2.24	1.017364	up	3.70E-06
DHCR24	22.675	25.84	0.604	0.787	51.673	25.625	36.099	98.087	83.91	17.69	1.363674	up	7.94E-11
C8B	89.547	86.71	117.459	114.207	87.322	49.303	43.724	31.436	33.322	58.978	-1.19201	down	3.51E-09
DAB1	0.954	0.815	3.121	1.119	2.971	0.328	0.779	0.372	0.644	0.869	-1.584	down	6.72E-13
TACSTD2	1.413	1.345	2.08	0.809	0.924	0.449	0.481	0.571	0.644	0.042	-1.58496	down	1.15E-09
HOOK1	6.839	5.512	9.275	6.471	4.374	21.678	48.023	21.418	21.23	4.533	1.847669	up	3.40E-19
KANK4	4.823	5.238	3.25	8.253	10.794	13.788	15.326	24.206	15.188	7.534	1.232546	up	5.20E-09
ANGPTL3	47.918	48.945	26.193	51.599	48.217	283.848	271.102	357.658	269.657	97.742	2.521838	up	8.41E-31
DNAJC6	2.165	1.793	2.369	0.473	3.857	7.649	2.894	6.284	5.72	1.88	1.196294	up	5.36E-09
LRRC7	0.163	0.131	0.238	0.109	0.105	1.214	0.324	0.269	0.258	0.201	1.596443	up	3.79E-13
PTGER3	0.949	0.873	0.52	2.608	1.158	1.052	0.2	0.18	0.023	0.672	-1.51967	down	1.85E-11
CRYZ	91.59	83.779	102.778	67.333	70.775	38.996	25.983	41.661	37.19	52.573	-1.08363	down	6.84E-08
NEU2	1.117	0.817	0.314	0.576	0.788	1.201	2.118	0.85	1.317	1.986	1.047666	up	1.11E-05

KLHL24	23.395	22.179	23.13	14.804	19.244	7.387	7.272	6.949	7.722	12.849	-1.28447	down	2.14E-10
EHHADH	274.411	257.616	291.099	309.829	356.991	79.287	105.241	73.985	105.192	149.461	-1.53775	down	5.79E-14
DNAJB11	20.71	21.364	20.588	17.848	19.015	52.93	55.667	69.967	46.496	32.695	1.372825	up	6.13E-11
SGPP2	0.885	0.962	0.263	1.936	3.467	0.895	0.59	0.482	0.273	0.872	-1.27019	down	2.14E-09
GK5	17.146	17.654	25.66	10.469	12.64	8.295	11.224	8.385	6.466	6.728	-1.02381	down	1.39E-06
PCOLCE2	2.885	2.765	3.479	2.576	4.171	0.498	0.828	0.806	0.648	1.849	-1.77697	down	5.66E-17
PLOD2	13.752	12.993	15.679	16.286	8.696	37.178	27.549	48.389	40.829	18.349	1.353858	up	1.35E-10
LOC12111	17.118	17.826	22.987	18.719	32.738	1.637	1.075	1.669	2.61	4.155	-3.29428	down	9.52E-50
BDH1B	4.741	5.446	4.386	2.933	11.844	1.979	2.712	2.815	1.567	3.752	-1.19409	down	8.82E-09
RTP2	5.707	6.898	5.74	12.564	6.633	2.252	2.465	0.437	1.473	6.247	-1.54368	down	1.66E-14
PDCD1	0.224	0.244	0.436	0.245	0.332	1.404	0.615	0.648	2.341	1.471	2.12545	up	8.68E-18
NPPC	0.462	0.14	0.227	0.205	0.146	0.746	1.08	0.82	0.647	0.035	1.491934	up	5.14E-08
ALPI	2.276	2.978	1.982	7.011	6.817	1.201	0.933	2.967	2.323	0.629	-1.38663	down	4.46E-11
CLCN2	5.259	5.023	6.373	7.091	8.974	2.931	1.353	1.262	1.523	5.362	-1.39587	down	3.46E-12
AHSG	647.406	586.191	341.982	385.887	516.601	2142.556	1030.178	1808.704	1610.561	1141.74	1.641949	up	2.57E-14
B3GNT5	0.276	0.209	0.584	0.163	0.331	0.684	0.226	0.46	1.333	1.221	1.325237	up	3.68E-10
LAMP3	0.695	0.522	1.546	0.971	1.067	1.884	1.387	1.291	3.334	3.014	1.183404	up	2.01E-08
ECT2	0.326	0.373	0.202	0.082	0.205	0.497	0.316	0.895	1.34	0.473	1.563438	up	1.65E-11
GHSR	0.047	0.122	0.13	0.269	0.096	0.094	0.263	0.199	0.111	1.407	1.635812	up	1.88E-07
LOC10705	0.51	0.516	1.065	0.257	2.652	0.203	0.148	0.246	0.087	1.037	-1.53594	down	5.01E-13
SMC4	0.936	0.823	1.281	0.925	1.01	3.295	2.818	2.897	4.124	3.095	1.704801	up	2.04E-15
IFT80	2.329	2.327	3.494	2.548	2.656	13.841	16.827	7.259	5.647	3.391	1.813928	up	3.73E-18
MLF1	1.58	1.77	10.575	6.262	8.683	2.217	0.904	1.313	1.273	1.961	-1.91196	down	1.65E-18
C3orf33	4.902	5.566	11.013	5.524	7.193	1.948	1.732	3.46	2.798	5.349	-1.16135	down	3.78E-08
ARHGEF26	23.545	19.621	29.884	21.264	16.511	9.765	7.18	11.488	5.14	11.002	-1.31388	down	1.44E-10
TUBA3E	0.192	0.163	0.031	0	0.099	0.464	0.281	1.152	1.686	0.095	2.910028	up	6.79E-26
DUOX1	0.118	0.177	0.05	0.051	0	1.206	0.125	1.108	2.964	0.833	3.960103	up	2.88E-54
KIAA0101	2.021	2.952	1.287	0.996	2.421	3.204	2.802	8.658	15.667	2.324	1.754148	up	2.06E-14
PATL2	0.431	0.311	0.936	0.477	0.321	2.482	0.635	1.903	2.365	0.836	1.729269	up	5.88E-15
CD276	1.304	1.262	1.756	1.389	1.793	2.978	3.016	2.529	3.547	3.187	1.023251	up	2.05E-06
CYP1A2	186.343	193.424	80.067	111.37	352.381	50.892	60.876	103.091	41.106	202.735	-1.00969	down	1.49E-07
CYP1A1	6.762	6.585	8.984	8.907	42.226	3.224	3.432	7.11	2.247	14.662	-1.25984	down	5.97E-10
ISLR	0.579	0.453	0.692	0.622	1.042	1.544	0.887	1.308	1.821	1.237	1.003398	up	6.61E-06
STOML1	1.689	2.009	3.074	2.163	1.329	6.384	2.342	6.463	6.854	3.523	1.316213	up	8.78E-10
PMLL	1.652	1.926	2.885	2.058	1.656	6.284	2.022	7.221	5.797	2.14	1.204735	up	1.12E-08
LOC10705	0.901	0.786	0.867	0.617	1.042	3.18	0.507	3.029	2.883	0.777	1.299314	up	2.13E-08
CHRNA3	0.746	0.418	1.464	0.81	0.164	0.43	0.449	0.21	0.211	0.263	-1.20187	down	2.55E-06
TRPM1	0.314	0.362	0.075	0.487	0.137	1.253	2.272	0.597	1.481	0.259	2.087955	up	1.05E-20

LIPC	10.231	9.369	17.981	17.582	21.798	3.588	1.603	1.163	2.424	0.88	-2.99368	down	1.91E-41
SEMA6D	5.433	4.993	4.704	5.885	5.547	1.91	1.462	2.173	1.762	3.793	-1.25843	down	2.08E-10
GATM	49.717	47.347	38.575	28.987	47.337	121.792	92.243	183.109	101.208	73.933	1.4329	up	1.09E-11
LOC41547	0.36	0.319	0.574	0.463	0.388	0.984	0.686	0.814	1.6	1.437	1.389676	up	2.28E-10
PEX11A	28.78	25.19	13.222	20.54	14.935	6.255	4.815	4.414	5.383	5.88	-1.94032	down	3.65E-21
MFGE8	138.896	146.087	39.039	75.693	26.068	209.686	272.142	331.272	272.039	33.05	1.392963	up	1.05E-10
HAPLN3	0.659	0.835	0.305	0.786	0.48	0.857	2.319	2.385	1.548	0.314	1.274735	up	3.43E-08
PGPEP1L	6.832	6.269	12.13	11.861	8.377	3.093	2.424	1.716	2.728	8.554	-1.29596	down	8.04E-11
ALDH1A3	0.407	0.44	0.452	0.311	0.185	0.595	1.064	1.4	0.726	0.457	1.238447	up	2.07E-08
PCSK6	10.296	9.946	18.096	13.867	8.212	22.718	33.566	32.089	24.935	12.148	1.054093	up	1.80E-07
CORO2B	0.557	0.608	0.694	4.147	1.16	0.461	0.321	0.503	0.271	1.987	-1.01517	down	9.56E-08
TMED3	10.988	11.981	10.281	14.372	11.035	29.089	34.282	38.512	25.331	19.644	1.323973	up	4.57E-10
PRC1	1.274	1.246	2.64	0.912	1.787	3.744	2.803	5.042	3.219	3.443	1.215035	up	4.49E-09
CETP	25.686	27.129	125.438	13.017	74.071	9.939	0.357	11.699	0.66	28.857	-2.36475	down	5.86E-28
CCL17	0.147	0.193	0.47	0.364	0	0.238	1.243	0.358	0.058	0.581	1.074521	up	0.005134
LCAT	92.519	82.067	354.367	283.575	528.767	82.853	23.394	33.627	37.259	393.787	-1.23226	down	2.92E-10
LOC41566	48.739	55.791	52.034	72.387	96.728	10.424	26.289	34.077	31.703	17.351	-1.44225	down	4.33E-12
LOC10174	652.428	670.99	572.533	1307.915	921.024	69.573	78.051	95.681	217.548	49.387	-3.0151	down	2.54E-43
LOC11253	29.432	32.367	17.144	21.458	6.47	2.401	3.734	2.847	5.98	2.822	-2.58688	down	8.10E-31
LOC10085	673.037	787.092	345.96	334.899	411.752	89.709	112.079	102.77	261.395	122.226	-1.89118	down	4.06E-20
LOC10174	10.563	11.234	12.576	16.408	21.977	5.505	4.562	7.264	5.717	12.353	-1.03921	down	1.53E-07
SETD6	20.448	20.309	9.756	6.154	7.196	6.776	5.82	8.443	5.365	4.66	-1.03962	down	1.85E-07
MT4	711.399	809.728	1038.719	734.886	291.299	83.781	144.292	30.548	87.105	180.963	-2.76735	down	1.79E-37
MT3	310.257	378.472	491.141	215.847	100.131	27.035	70.51	12.299	32.914	75.275	-2.77832	down	5.92E-37
SLC6A2	2.224	2.327	2.448	1.587	1.529	9.372	7.735	8.939	11.565	1.786	1.96106	up	2.12E-20
CDCA9	20.937	20.591	13.664	15.924	17.513	5.87	4.339	3.302	6.951	15.241	-1.31161	down	2.38E-11
LOC10085	3.081	2.797	4.203	2.885	2.036	9.127	2.497	11.556	21.729	4.802	1.728075	up	7.84E-16
RGS9BP	1.13	1.118	2.26	1.351	2.091	0.632	0.556	0.725	0.702	1.252	-1.03878	down	9.01E-07
LOC7697C	73.157	72.687	118.776	84.236	242.712	64.129	19.946	35.578	18.688	57.958	-1.59147	down	1.32E-14
CES1L2	38.947	39.648	81.674	126.021	111.344	31.16	13.135	21.091	14.815	55.479	-1.5512	down	1.15E-14
TERB1	3.686	3.169	6.137	3.53	1.611	2.444	0.736	2.161	0.482	1.771	-1.25513	down	2.14E-09
BEAN1	0.894	0.509	0.588	0.831	0.599	1.474	0.962	1.745	0.984	2.453	1.153834	up	4.41E-06
CENPN	0.316	0.447	0.405	0.375	0.612	1.357	0.769	1.414	1.775	1.227	1.599803	up	6.07E-12
LOC12111	0.38	0.345	0.482	0.407	0.357	0.644	1.368	0.73	1.849	0.368	1.32892	up	5.06E-10
CDH13	0.334	0.208	0.212	0.419	0.249	1.108	0.689	0.391	0.441	0.736	1.239763	up	1.92E-08
GINS2	0.197	0.297	0.676	0.237	0.436	0.978	0.409	0.992	1.271	1.135	1.374061	up	1.40E-09
SLC7A5	13.905	12.708	34.749	16.312	13.231	6.573	9.28	8.634	10.873	9.532	-1.01782	down	1.92E-06
CA5A	214.2	216.023	174.242	126.055	187.64	37.675	32.022	34.953	85.811	109.969	-1.6117	down	1.27E-15

CDT1	0.437	0.767	1.142	0.632	1.276	1.1	0.826	2.259	2.989	1.707	1.061019	up	9.72E-07
SPIRE2	9.4	10.136	19.47	2.665	10.706	0.673	3.346	1.499	0.424	6.192	-2.10942	down	1.26E-22
LOC12111	0.211	0.11	0.134	0.277	0.477	0.85	0.901	1.125	0.801	0.499	1.78408	up	1.95E-10
LOC1211C	0.174	0.228	15.743	0.613	18.161	0.702	2.572	1.749	0.118	0.025	-2.7557	down	1.28E-29
NT5DC2	16.51	16.917	14.552	32.542	49.533	6.486	4.984	7.675	8.237	29.479	-1.19353	down	6.55E-10
SMIM4	37.41	36.755	18.075	56.563	44.851	12.022	17.213	10.776	14.377	38.84	-1.05461	down	3.51E-08
ITIH3	533.19	482.107	639.242	820.104	691.524	260.99	241.726	242.741	283.687	356.63	-1.19204	down	3.08E-09
MUSTN1	1.16	0.901	1.501	1.907	1.043	0.292	0.897	0.22	0.229	0.858	-1.3817	down	2.42E-07
MANF	17.274	19.109	30.432	24.052	29.633	56.386	43.74	65.684	40.386	37.786	1.017711	up	7.80E-07
GBP4L	2.605	3.432	8.426	5.351	3.121	10.063	4.616	10.847	21.867	11.713	1.365561	up	6.95E-11
LOC1211C	5.142	5.183	15.106	2.359	0.336	10.226	11.359	13.409	18.488	16.835	1.321815	up	5.01E-11
LOC1211C	2.474	2.67	6.941	1.457	0.379	14.145	5.419	10.38	21.973	3.058	1.981126	up	1.96E-21
LOC1211C	2.451	2.595	15.727	11.153	0.737	16.069	8.335	17.909	37.984	3.712	1.362752	up	1.62E-11
LOC1211C	0.611	0.703	0.724	0.475	0.764	0.013	1.783	0.451	4.863	0.195	1.155296	up	9.08E-08
MST1R	0.552	0.61	1.135	0.754	0.866	3.693	2.81	3.569	2.266	1.463	1.815634	up	9.92E-18
USP4	34.851	31.939	33.217	25.706	30.698	17.573	9.127	11.879	13.336	17.774	-1.16628	down	5.83E-09
OASL	7.621	9.268	20.363	15.717	5.168	103.727	8.431	97.642	126.245	14.252	2.590947	up	7.45E-33
OGN	0.744	0.5	1.068	0.904	0.452	2.078	1.614	1.347	0.97	1.559	1.043906	up	1.63E-06
DNASE1L3	5.72	5.065	11.301	8.801	1.546	8.912	10.59	11.683	24.125	20.983	1.23396	up	8.79E-09
ABHD6	87.008	72.637	40.937	70.693	36.2	25.822	29.74	18.914	25.484	50.519	-1.03088	down	6.79E-08
C3orf67	0.125	0.057	0.03	0	0.038	2.493	0.365	0.219	0.059	0.123	3.678072	up	9.21E-38
GPR27	0.309	0.392	0.087	0.463	0.149	0.366	1.942	0.794	0.547	0.251	1.474752	up	3.04E-09
PROK2	0.259	0.207	0.046	0.118	0	0.719	1.519	1.168	0.819	0.198	2.801827	up	2.24E-22
GHRL	0.524	0.374	1.213	0.782	0.831	0.537	0.054	0.577	0.377	0.188	-1.10136	down	0.000125
NRG2	9.131	9.026	8.668	4.559	7.119	3.028	2.953	3.415	3.001	3.981	-1.23296	down	1.72E-09
MZB1	0.125	0.383	1.398	1.271	0.429	1.55	1.081	0.481	1.554	2.883	1.064842	up	1.81E-05
TENM2	2.348	2.093	5.216	7.734	11.746	0.131	0.494	0.235	1.01	1.345	-3.17797	down	4.80E-47
HMMR	0.161	0.243	0.444	0.214	0.458	0.75	0.362	0.669	1.256	1.063	1.428573	up	4.86E-10
PTTG2	0.163	0.266	0.486	0.201	0.251	1.41	0.457	0.789	1.963	1.523	2.163602	up	1.02E-15
SLC26A2	5.439	5.176	4.765	3.805	3.095	9.588	21.007	19.652	7.435	6.297	1.521638	up	2.53E-13
LOC41614	9.844	10.681	17.565	12.045	8.298	37.834	10.796	35.799	42.31	17.665	1.305182	up	5.11E-10
STC2	18.675	18.883	15.306	31.688	29.823	5.336	3.785	6.927	9.562	13.897	-1.53347	down	2.19E-14
RGS14	1.184	0.92	2.604	1.046	1.656	2.946	3.044	2.189	3.605	3.529	1.046709	up	4.14E-07
MXD3	0.861	1.164	1.964	0.786	1.454	0.247	0.323	0.371	0.393	0.528	-1.73944	down	1.42E-13
CPLX2	2.001	2.203	1.388	0.619	0.305	1.342	2.717	5.934	4.467	1.172	1.261799	up	7.81E-09
RASGEF1C	0.176	0.124	0.211	0.266	0.188	1.061	1.064	1.007	0.152	0.946	2.126305	up	7.97E-22
TRPC7	0.315	0.344	1.872	1.381	0.405	0.815	0.015	0.064	0.665	0.375	-1.15639	down	2.61E-07
SLC22A5	40.76	42.726	34.423	30.492	37.282	11.963	10.382	7.845	12.734	15.138	-1.67709	down	3.24E-16

IRF1	16.405	14.38	24.922	15.38	15.174	32.25	17.84	36.518	57.777	34.635	1.053297	up	4.61E-07
LEAP2	96.682	98.368	85.891	140.103	52.303	328.323	235.969	166.062	158.155	124.794	1.098087	up	3.57E-07
YIPF5	30.659	28.106	20.453	18.425	20.253	60.719	71.467	73.375	61.991	27.745	1.324615	up	2.96E-10
GFRA3	290.416	277.802	351.34	274.504	381.394	127.519	105.519	101.338	157.309	249.937	-1.08701	down	5.01E-08
CPPED1	21.166	19.616	21.668	25.119	27.45	18.282	13.648	6.643	2.475	11.293	-1.13578	down	2.24E-08
TXNDC11	11.298	10.023	15.962	11.888	11.045	39.413	43.276	31.548	19.961	17.391	1.331874	up	8.98E-11
BHLHA15	0.159	0.415	0.505	0.326	0.163	1.791	8.918	0.818	4.644	1.095	3.456761	up	8.33E-34
NPTX2	0.437	0.313	0.673	0.648	1.722	0.182	0.143	0.282	0.167	0.916	-1.16395	down	1.71E-07
CYP3A5	1487.008	1384.543	900.646	1710.507	882.877	328.52	432.955	442.289	353.507	953.732	-1.34203	down	6.19E-12
LOC10174	1.163	1.458	0.216	0.653	0.756	1.547	4.117	4.664	3.986	0.344	1.786306	up	2.04E-15
LOC42766	0.308	0.201	0.335	2.208	1.53	0.483	1.001	0.088	0.128	0.574	-1.00915	down	3.57E-06
CACNA1H	0.06	0.042	0.158	0.103	0.147	1.868	2.35	0.928	1.171	0.128	3.646655	up	2.78E-54
SOX8	0.807	0.688	2.121	0.341	1.305	0.394	0.522	0.267	0.522	0.795	-1.07217	down	1.05E-05
PLK1	0.239	0.073	0.279	0.157	0.425	0.68	0.697	0.868	1.296	1.364	2.059383	up	8.09E-18
IL21R	0.252	0.279	0.617	0.485	0.398	0.679	0.326	0.98	1.7	1.755	1.419194	up	2.82E-10
SDR42E2	0.095	0.134	0.881	0.273	0.195	1.083	0.373	0.613	0.75	0.53	1.083221	up	1.11E-06
CDR2	0.863	0.893	1.639	0.497	0.67	1.945	3.384	3.061	1.338	1.078	1.243181	up	7.52E-10
RMI2	16.163	14.381	11.234	5.409	5.134	2.59	5.354	2.935	5.181	4.539	-1.3446	down	5.80E-11
CIITA	0.57	0.492	0.821	0.551	0.448	1.055	0.9	1.17	2.093	1.129	1.13764	up	6.74E-08
GRIN2A	0.055	0.015	0.03	0.006	0.005	1.315	0.003	1.156	0.063	1.41	5.090386	up	1.56E-82
CARHSP1	2.219	2.095	4.488	3.529	2.904	9.449	5.633	5.132	5.478	5.872	1.050646	up	5.84E-07
HBAD	93.174	97.936	309.947	23.94	123.835	18.127	27.947	21.12	9.019	21.3	-2.73412	down	8.51E-34
HBA1	365.186	383.036	1151.922	127.271	387.325	63.447	98.553	69.982	49.828	107.463	-2.633	down	2.74E-32
PDIA2	0.22	0.352	0.575	0.382	0.314	1.402	1.294	1.789	0.542	0.362	1.545391	up	2.96E-12
ARHGDIG	0.17	0.089	0.703	0	0.244	0.274	1.146	0.268	0.349	0.569	1.108404	up	2.74E-05
LOC41665	7.203	7.536	6.535	1.798	2.021	0.067	0.282	0.198	0.307	0.157	-4.6266	down	5.26E-79
C16orf96	1.74	1.911	1.353	1.788	1.298	0.635	0.815	0.691	0.656	0.572	-1.26257	down	7.35E-09
DECR2	121.269	119.623	149.746	120.838	144.37	40.026	49.394	42.03	72.323	90.626	-1.15557	down	9.97E-09
LOC1211C	1.23	1.319	0.49	1.095	0.035	0.028	0.077	0.124	0.027	1.482	-1.25986	down	1.20E-08
LOC1211C	0.117	0.1	0.196	0.087	0.193	1.723	0.621	0.249	0.492	0.972	2.540891	up	8.39E-25
ROGDI	7.316	5.888	6.138	4.855	4.172	3.405	2.517	2.267	2.264	3.685	-1.00448	down	7.19E-07
GPRC5B	5.146	4.835	6.084	6.555	6.005	2.778	0.747	0.529	1.781	5.572	-1.32698	down	1.20E-11
DNAH3	1.245	1.078	1.391	0.579	1.01	0.67	0.117	0.014	0.227	0.666	-1.64348	down	1.08E-15
SDF2L1	19.198	16.741	25.485	18.423	18.859	88.437	88.19	66.905	63.081	42.342	1.821779	up	3.94E-18
CDC45	0.14	0.165	0.367	0.161	0.287	0.462	0.236	0.966	1.604	1.185	1.986472	up	6.09E-17
CLDN5	8.478	13.541	14.928	11.196	19.093	5.356	6.219	7.423	5.865	7.172	-1.06947	down	2.50E-07
ULK1	6.747	6.858	6.248	5.161	3.376	14.663	13.832	9.097	14.065	8.641	1.086593	up	2.78E-07
RIMBP2	2.897	2.804	1.685	3.33	2.154	5.831	3.442	7.552	7.956	2.81	1.099887	up	4.06E-07

TMEM132	0.374	0.359	0.091	0.18	0.082	0.57	0.381	1.248	1.285	0.265	1.782778	up	6.40E-15
AACS	7.247	6.549	6.818	7.214	4.705	32.389	18.568	50.098	66.268	14.097	2.479176	up	5.79E-30
ATP2A2	15.283	13.796	24.41	14.236	13.583	53.548	45.722	52.86	45.609	31.848	1.497514	up	4.58E-13
P2RX7	4.314	3.019	8.787	4.016	2.9	15.668	4.431	12.886	20.749	13.39	1.542733	up	1.73E-13
WDR66	0.978	0.668	2.082	1.005	0.892	1.224	4.286	2.429	2.856	2.186	1.20575	up	2.36E-09
RAD9B	1.605	1.591	1.683	1.202	0.539	0.115	0.429	0.193	0.239	0.548	-2.11533	down	3.25E-20
PPTC7	26.451	23.969	28.714	22.315	24.565	9.772	6.365	6.363	7.59	18.094	-1.38687	down	4.69E-12
TMEM116	11.563	10.646	6.492	14.387	17.031	4.779	2.337	3.023	3.405	15.478	-1.05055	down	2.05E-08
SELPLG	1.126	1.341	1.74	1.092	1.388	2.47	2.28	2.623	3.885	3.109	1.102751	up	4.77E-07
UNG	10.68	12.674	7.614	8.913	9.779	3.004	2.234	1.807	3.013	5.141	-1.70778	down	2.17E-16
ACACB	1.373	1.365	0.944	2.486	1.391	0.09	0.278	0.051	0.1	0.018	-3.80278	down	1.31E-56
VPS29L	2.106	2.448	1.502	1.318	6.329	1.242	0.636	1.392	0.92	1.147	-1.35957	down	8.49E-10
CHCHD10	310.225	289.108	416.78	254.481	521.347	132.285	180.989	142.294	148.809	267.479	-1.03936	down	3.80E-07
LOC1211C	1.256	1	0.391	0.987	1.794	0.484	0.123	0.43	0.432	1.13	-1.06102	down	1.74E-05
MMP11	1.993	2.144	0.992	1.228	1.358	0.572	0.495	0.465	0.288	0.567	-1.69038	down	2.01E-14
SLC2A11L	3.606	3.592	3.219	2.567	6.134	0.5	0.671	0.766	0.891	2.06	-1.96652	down	1.22E-19
LOC76955	17.466	12.831	48.828	14.684	24.095	7.158	4.199	4.854	3.309	18.978	-1.61463	down	1.38E-14
GSTT1L	3.5	3.63	9.075	10.313	21.286	2.134	2.112	4.555	1.773	4.88	-1.62884	down	1.42E-14
TBX6	2.077	2.257	1.313	2.702	4.297	0.676	0.546	1.011	0.52	1.954	-1.42484	down	1.89E-12
SLC5A1	0.49	0.334	0.639	0.377	0.557	0.765	1.348	0.961	0.487	1.254	1.004797	up	4.15E-06
LOC41695	1.175	1.185	0.618	1.032	0.658	4.352	4.254	4.939	4.7	0.76	2.024338	up	9.70E-21
PISD	20.194	19.222	12.543	12.178	6.629	71.801	90.366	91.411	92.274	8.668	2.324657	up	4.41E-27
MLEC	12.808	13.396	10.653	9.082	10.351	28.342	31.065	25.986	20.223	16.974	1.122821	up	8.89E-08
LOC10085	4.583	5.225	3.024	4.197	7.868	1.848	1.693	1.389	1.347	2.904	-1.43875	down	5.19E-11
HSPB8	0.197	0.188	0.371	0.133	0.46	0.491	0.423	0.37	0.667	1.095	1.172054	up	1.77E-06
PRODH	0.862	0.852	4.639	2.415	3.652	1.632	20.005	1.142	1.072	3.105	1.117628	up	2.84E-09
SLC35E4	0.209	0.239	0.312	0.204	0.174	1.778	1.305	4.43	1.197	1.08	3.09922	up	1.19E-38
TCN2	106.731	126.53	35.771	38.128	31.915	332.772	299.846	413.46	310.692	97.82	2.10092	up	5.23E-22
HORMAD:	1.423	1.088	1.587	0.976	1.965	0.672	0.699	0.43	0.382	0.982	-1.15191	down	2.80E-08
LOC41701	111.681	109.563	124.638	98.657	150.498	46.613	29.092	18.212	36.546	100.294	-1.36659	down	6.45E-12
LOC10704	0.367	0.261	0.508	1.415	1.097	2.019	0.95	2.282	0.883	2.326	1.212428	up	1.34E-07
LOC11253	0.943	0.745	1.425	1.364	3.358	3.948	3.146	3.235	6.435	4.89	1.466041	up	6.66E-12
LOC10704	0.14	0.252	0.335	0.202	0.216	0.509	0.827	1.19	2.273	2.59	2.684721	up	2.42E-23
MHCY2	0.315	0.289	0.251	0.078	0.907	0	0.035	5.335	0.112	9.388	3.011197	up	4.40E-36
MHCY14	1.109	0.968	2.202	1.215	0.577	0	0.283	0.072	0.304	0.058	-3.07305	down	2.72E-33
LOC1211C	1.131	0.841	0.951	0.317	0.172	0.259	0.173	0.101	0.244	0.304	-1.65371	down	1.09E-12
MHCY9	1.902	1.998	3.085	1.291	0.376	0.225	0	0.646	1.616	0.464	-1.55022	down	2.96E-12
LENG9L7	4.913	5.016	7.793	9.996	2.767	0.144	0.067	0.18	0.313	0.117	-5.20605	down	2.04E-86

MHCY7	1.2	0.974	2.181	1.171	0.861	0.01	0.253	0.682	0.96	0.234	-1.57596	down	5.67E-12
ZNFY1	1.434	1.495	1.681	1.217	0.89	0.47	0.053	0.726	0.798	0.714	-1.28109	down	2.65E-09
ZNFY3	0.19	0.124	0.239	0.221	0.844	0.726	1.723	0.747	0.312	0.981	1.469337	up	1.36E-10
LOC1211C	1.892	1.345	2.562	4.688	0.933	0.084	0.385	0.677	0.33	0.235	-2.73507	down	3.91E-32
MHCY12	0.498	0.523	0.722	0.468	0.858	0.086	0.48	2.922	9.805	0.123	2.126303	up	1.30E-21
LOC1211C	0.996	0.879	4.17	0.918	0.279	0.061	0.051	0.229	0.203	0.119	-3.43946	down	6.97E-40
LOC1211C	0.866	0.54	1.248	0.39	0.508	0.05	0.441	0.138	0.261	0.041	-1.92608	down	4.46E-13
LOC1211C	1.782	1.403	1.563	0.448	0.336	0.059	0.531	0	0.058	0	-3.08395	down	3.47E-25
LOC1211C	10.943	11.057	14.532	7.957	1.516	0.668	0.283	3.099	4.585	0.869	-2.27458	down	6.68E-26
MHCY8	2.228	1.938	2.656	1.732	1.521	0.728	0.69	1.095	1.277	0.498	-1.23144	down	1.14E-08
MHCY32	0.15	0.147	0.268	0.446	8.325	0.03	0.168	14.085	1.644	14.556	1.706593	up	2.25E-15
BG8	13.328	12.282	19.78	22.633	26.128	3.624	2.564	6.323	1.646	4.757	-2.31522	down	3.18E-27
MOGL	13.688	10.911	1.441	3.719	2.789	7.049	1.744	0.294	0.217	0.78	-1.69001	down	4.67E-17
LOC1211C	2.086	2.181	22.64	26.21	25.371	3.02	0.494	0.224	1.369	0.867	-3.71459	down	3.74E-59
BTN3A3L2	1.23	0.66	7.03	5.052	5.355	2.042	0.304	2.594	0.72	1.624	-1.4072	down	1.98E-10
BTN3A3L1	0.297	0.36	0.624	0.731	0.914	0.615	7.669	0.064	2.966	0.501	2.011765	up	8.97E-20
KIFC1	0.701	0.582	4.305	3.332	4.287	0.897	11.002	13.505	13.223	13.219	1.972523	up	5.32E-21
IL4I1	0.662	0.6	1.835	0.561	0.701	1.273	0.936	2.575	2.33	3.807	1.324042	up	1.58E-09
TRIM7.1	0.057	0.037	2.341	0.117	0.059	0	0.498	0.312	0.011	0.07	-1.54579	down	9.21E-08
TRIM39.2	0.25	0.286	0.629	0.256	0.171	0.738	0.374	0.567	1.086	1.168	1.302099	up	1.02E-07
TRIM27.1	0.821	0.682	1.079	0.278	0.455	0.242	0.337	0.332	0.082	0.321	-1.33174	down	1.56E-08
DMA	2.389	2.955	4.634	5.282	2.944	7.599	6.397	8.427	11.099	7.491	1.171605	up	6.92E-08
LOC1211C	1.894	1.973	0.277	0.921	0	0.031	0.174	0.164	0.367	0.076	-2.63358	down	7.21E-21
FCN2	1.003	1.114	0.965	0.611	1.238	0.512	0.333	0.328	0.548	0.647	-1.05663	down	3.96E-07
MAN1B1	82.815	78.378	81.445	67.277	71.142	39.576	35.294	37.321	41.207	33.043	-1.03127	down	3.58E-07
NOXA1	4.153	3.657	5.116	3.606	3.823	2.106	1.542	1.481	1.55	2.754	-1.10918	down	6.32E-08
RNF208	0.386	0.367	0.526	0.451	0.358	1.015	0.575	0.665	1.598	1.745	1.420627	up	8.44E-10
STPG3	2.738	3.512	3.672	2.191	2.479	1.017	1.499	0.677	1.853	1.704	-1.11164	down	3.43E-07
NSMF	0.468	0.31	1.023	0.218	1.076	6.192	7.651	5.368	5.713	4.551	3.249396	up	2.53E-49
FAM166A	33.777	34.031	43.765	26.479	43.461	120.746	89.733	88.04	97.237	48.604	1.291633	up	3.87E-10
TUBB4B	52.874	54.095	66.87	41.008	66.365	186.767	143.689	140.012	155.422	78.463	1.324625	up	1.49E-10
TNFSF15	0.273	0.251	0.457	0.315	0.309	1.014	1.045	0.647	0.433	0.755	1.276043	up	4.31E-07
SLC25A25	43.558	44.396	31.78	31.397	45.136	10.877	5.836	4.68	7.972	57.383	-1.17787	down	2.87E-10
FPGS	41.104	41.489	85.376	36.875	85.68	13.877	8.455	8.358	10.047	72.742	-1.35619	down	9.17E-12
DNM1	9.543	8.703	6.504	6.356	9.262	2.138	3.302	2.846	4.893	4.696	-1.17504	down	5.15E-09
LOC1211C	0.464	0.938	0.302	1.56	1.82	0.782	0.142	0.921	0.267	0.083	-1.20988	down	1.92E-06
LOC4171S	0.381	0.311	0.657	0.73	0.326	1.28	0.678	1.424	1.683	1.001	1.332901	up	4.84E-08
FAM69B	0.345	0.319	0.485	0.751	0.563	1.882	2.898	4.418	1.14	0.3	2.108491	up	2.07E-20

AGPAT2	9.837	11.148	19.814	12.831	6.98	112.598	104.361	188.407	97.061	22.273	3.11376	up	1.40E-45
EGFL7	3.296	4.035	7.742	4.319	4.197	9.933	12.758	18.026	9.519	4.716	1.219883	up	1.56E-09
HSPA5	203.11	183.635	232.322	172.83	247.172	1528.954	1345.647	1458.495	1114.045	602.187	2.541478	up	4.24E-32
MVB12B	4.325	3.783	5.199	4.809	4.784	9.005	9.731	28.088	10.058	6.054	1.458338	up	2.43E-12
HS3ST3A1	5.418	5.768	2.626	4.097	4.397	0.843	0.4	0.666	0.392	0.702	-2.89088	down	2.14E-40
FN3K	3.913	3.747	7.322	10.982	14.227	2.805	2.647	2.302	3.91	3.899	-1.36847	down	3.70E-11
CDK3	3.108	2.939	1.859	3.759	2.426	0.832	0.412	0.198	0.301	3.585	-1.40227	down	5.21E-13
TEN1	4.687	5.166	5.237	10.568	7.423	2.064	0.908	1.158	0.935	8.637	-1.27131	down	3.21E-11
ACOX1	304.321	272.828	426.332	464.053	441.56	145.322	114.213	85.249	112.94	276.927	-1.37775	down	6.91E-12
FASN	110.775	119.381	100.058	62.645	146.049	394.388	286.654	367.248	372.764	281.898	1.659918	up	4.30E-15
LOC41738	0.606	0.721	0.922	0.181	0.793	1.733	1.673	2.005	1.134	1.141	1.252531	up	3.85E-07
NOG	0	0.155	0.471	0	0	0	2.327	0.681	2.48	0.175	3.167128	up	9.51E-23
KPNA2	1.883	1.577	2.2	1.866	1.482	2.583	1.84	3.628	7.185	5.551	1.205949	up	3.85E-08
SOX9	0.606	0.523	1.428	0.239	0.572	2.13	1.417	1.528	0.997	1.337	1.13622	up	2.15E-07
SDK2	0.273	0.481	0.456	0.484	0.421	1.246	1.235	2.598	0.878	1.192	1.754686	up	6.65E-16
RNF213	3.711	3.683	12.269	5.923	4.87	19.664	6.717	21.983	23.594	9.437	1.418065	up	3.06E-12
CCDC40	0.266	0.269	0.549	0.487	0.286	0.536	1.823	0.888	0.603	0.286	1.153126	up	1.18E-07
CBX2	0.516	0.535	0.24	0.106	0.309	2.134	1.935	2.857	2.707	0.403	2.552991	up	2.03E-26
RBFOX3	0.214	0.159	0.327	0.123	0.086	0.902	1.195	1.617	1.682	0.23	2.623125	up	6.92E-30
ENPP7	0.952	1.027	0.061	0.529	0.026	1.619	1.563	4.073	5.091	0.152	2.265691	up	5.68E-21
TIMP2	102.314	120.649	120.318	83.634	148.091	322.011	498.974	640.334	533.391	133.628	1.888069	up	1.72E-19
SOCS3	3.048	2.755	5.606	4.452	1.599	15.26	64.479	17.141	12.961	4.021	2.704811	up	1.22E-36
TK1	1.116	0.548	2.311	0.917	3.497	5.895	4.769	9.983	8.655	8.142	2.157497	up	4.55E-22
LOC42210	13.492	13.127	23.518	13.071	13.669	7.246	7.804	7.233	8.019	7.793	-1.01285	down	1.42E-06
KRABZFP	0.342	0.28	0.204	0.421	0.176	1.723	0.288	1.088	1.69	0.59	1.914682	up	4.26E-09
GRIN2C	1.068	1.271	0.191	0.296	0.839	0.322	0.051	0.471	0.267	0.287	-1.38727	down	1.81E-11
OTOP3	0.434	0.426	0.49	0.694	0.793	1.75	0.596	1.287	0.601	2.198	1.179482	up	3.49E-07
HN1	39.239	38.608	28.761	35.62	42.532	17.692	10.363	10.263	13.8	28.366	-1.19883	down	8.79E-10
SLC16A5	100.899	98.569	67.509	91.142	104.637	26.619	14.353	8.407	17.475	62.697	-1.83669	down	4.75E-20
CCL4	0.67	0.776	1.231	0.127	1.059	7.19	1.101	0.719	9.053	1.118	2.310394	up	2.99E-22
LOC10705	0.357	0.379	0.887	0.348	0.24	0.503	0.526	0.716	1.27	1.648	1.074847	up	1.47E-06
CASP1	8.277	8.805	8.323	8.909	10.936	18.695	16.424	25.318	21.072	13.492	1.069941	up	3.89E-07
P2RX1	0.225	0.123	0.807	0.756	0.366	0.469	1.434	0.557	0.267	1.942	1.034359	up	1.04E-05
MIS12	0.314	0.78	0.4	0.774	1.29	3.341	1.412	3.883	4.421	1.671	2.047887	up	3.46E-16
TMEM120	19.329	20.747	33.045	32.708	21.598	11.054	8.401	10.428	10.822	17.53	-1.12965	down	2.15E-08
CCLI5	0.239	0.219	0.991	0.197	0.984	0.27	0.269	1.8	1.477	2.029	1.150634	up	2.88E-06
SERPINF1	74.377	64.558	89.8	202.538	74.576	17.74	24.386	19.975	20.411	44.637	-1.99214	down	2.62E-22
SRR	5.107	4.768	4.455	3.419	3.831	0.949	2.097	1.775	1.93	2.771	-1.17994	down	1.92E-08

MED13	24.129	20.91	40.145	20.433	24.737	12.929	6.523	6.929	9.464	27.675	-1.03709	down	1.73E-07
ACACA	39.426	40.517	32.023	29.127	40.439	120.426	74.149	97.951	91.157	121.275	1.475914	up	6.64E-12
C17orf78	0.122	0.175	0.135	0.12	0.374	1.627	0.191	0.192	0.385	0.551	1.664351	up	1.16E-09
EVI2A	0.625	0.324	0.788	0.835	0.294	1.911	1.23	0.758	1.937	1.694	1.392054	up	4.63E-09
FBXO39	0.27	0.238	0.364	0.239	0.256	0.637	0.28	0.637	1.003	0.697	1.248149	up	7.18E-08
LOC10705	6.771	7.469	7.903	5.264	7.808	14.835	17.9	20.912	19.457	7.839	1.200601	up	6.06E-09
ASIP	0.562	0.735	1.598	0.198	0.165	0.129	0.271	0	0.127	0.475	-1.69614	down	1.14E-06
TOX2	0.328	0.221	0.563	0.379	0.442	0.694	0.507	0.684	1.906	1.132	1.346434	up	1.54E-09
PI3	9.954	13.416	15.077	52.646	13.145	23.654	148.342	17.561	16.105	127.682	1.677083	up	2.07E-14
GDAP1L1	0.38	0.327	1.069	0.882	0.257	0.336	0.167	0.163	0.051	0.31	-1.50053	down	4.91E-11
EPB42	0.861	1.048	2.935	0.467	0.938	0.236	0.232	0.198	0.136	0.187	-2.65346	down	9.50E-26
ZNFX1	6.703	7.556	14.281	12.287	5.833	33.906	5.24	39.423	35.009	8.915	1.392345	up	2.95E-11
HELZ2	6.097	6.136	11.043	5.603	7.422	24.107	8.121	13.314	22.484	5.886	1.025698	up	4.88E-07
RAD21L1	0.284	0.518	0.198	0.719	0.109	3.09	0.16	0.244	0.627	0.21	1.242158	up	5.11E-07
TPX2	0.395	0.488	0.943	0.663	0.899	1.508	1.379	1.139	2.389	2.088	1.326259	up	6.02E-10
LOC77197	0.556	0.685	0.352	0.43	0.017	0.541	1.747	4.464	1.45	0.5	2.090075	up	1.98E-19
LOC10085	0.582	0.533	0.463	0.064	0.199	0.344	2.049	2.821	0.613	0.841	1.853933	up	1.84E-13
WFDC2	5.532	6.109	0.358	1.952	0	11.389	9.356	16.298	11.054	1.386	1.826193	up	2.45E-16
WFDC8	75.251	79.538	7.492	29.801	0.154	178.628	242.241	245.476	225.821	35.544	2.270766	up	9.28E-25
SPINT4	2.528	2.408	0.318	0.474	0	9.906	13.364	23.343	12.281	1.093	3.387408	up	1.36E-45
LOC77199	0.826	0.42	0.122	0.151	0.157	0.888	1.685	1.039	0.532	0.905	1.588106	up	1.07E-09
UBE2U	0.241	0.158	0.346	0.516	0.843	2.102	1.086	2.43	4.966	4.426	2.831773	up	5.51E-28
NPEPL1	18.407	17.491	14.618	14.788	21.113	39.995	54.86	77.928	100.387	21.546	1.769879	up	5.11E-17
ESPN	0.409	0.47	0.315	0.23	0.162	0.886	1.494	4.52	2.29	0.262	2.571449	up	3.14E-30
MIB2	37.939	37.536	44.728	17.633	58.931	18.385	15.064	13.335	16.496	27.457	-1.11668	down	4.38E-08
LOC41940	8.82	8.759	5.918	5.218	47.946	1.154	1.143	1.279	0.752	1.573	-3.69833	down	8.81E-59
LOC77106	34.474	35.519	33.965	35.915	49.009	19.526	7.263	8.487	11.981	30.924	-1.27254	down	9.30E-11
PLEKHN1	0.802	0.886	0.539	2.022	1.036	0.401	0.559	0.411	0.339	0.531	-1.23591	down	6.72E-09
RERE	11.327	10.453	12.247	9.195	9.996	19.512	25.535	35.512	26.059	8.604	1.114357	up	5.23E-08
SLC25A33	27.624	24.697	21.936	16.748	18.365	11.291	8.546	7.145	9.041	18.453	-1.00546	down	2.96E-07
RNF186	11.188	11.918	0.265	5.554	0.043	23.541	24.128	20.036	31.49	0.635	1.784837	up	1.03E-15
CAMK2N1	11.466	14.312	4.295	4.522	3.703	2.732	31.148	37.738	1.094	4.622	1.013739	up	1.38E-06
CELA2A	0.203	0.266	0.485	0.467	0.334	0.688	1.416	0.517	2.057	0.441	1.541695	up	3.58E-09
MTHFR	13.337	12.16	30.133	12.285	4.395	20.253	65.768	35.209	36.767	11.866	1.232047	up	2.89E-10
DRAXIN	0.724	0.636	0.465	0.337	0.311	0.366	1.518	2.47	1.71	0.245	1.349378	up	6.19E-10
EPHB2	3.285	3.747	3.113	2.948	5.027	7.427	6.865	10.194	7.969	4.712	1.036235	up	7.82E-07
LOC11253	13.755	12.357	22.05	16.679	10.052	6.275	5.814	5.032	5.646	11.608	-1.12336	down	2.75E-08
DPYSL2	1.602	1.773	1.878	1.072	1.27	2.779	9.301	26.325	4.427	2.231	2.568032	up	9.06E-33

EGR3	0.164	0.197	0.283	0.202	0	0.573	0.646	0.415	1.493	0.863	2.230964	up	5.65E-16
LOC10174	25.547	25.293	7.821	17.495	25.19	9.613	3.893	6.609	5.884	18.577	-1.18486	down	7.70E-10
ADAM32L	1.354	1.2	1.01	1.38	1.989	0.235	0.424	0.27	0.054	1.267	-1.62139	down	8.88E-14
IDO2	10.394	12.035	5.352	19.063	27.269	0.761	3.314	1.001	0.147	20.837	-1.50771	down	3.61E-15
ANK1	1.223	1.303	2.635	0.715	1.221	0.36	0.35	0.312	0.451	0.732	-1.68418	down	3.47E-15
MTHFD2	1.543	1.265	4.583	0.984	0.851	4.752	4.867	5.961	5.847	2.065	1.347918	up	2.41E-11
NCAPH	0.605	0.485	0.927	0.671	0.544	1.329	1.207	1.256	1.376	1.341	1.008886	up	5.21E-06
ADD2	1.232	1.137	1.521	0.989	0.687	0.323	0.483	0.278	0.272	0.772	-1.38505	down	1.03E-10
FAM136A	14.819	16.737	21.821	15.24	29.508	7.276	6.888	10.542	7.729	13.627	-1.09096	down	1.66E-07
CASP14	1.235	1.087	1.424	1.278	0.897	0.223	0.096	0.142	0.101	0.273	-2.8186	down	1.71E-28
RETSAT	181.098	181.873	305.999	306.703	245.919	66.088	68.214	83.969	62.694	74.078	-1.78268	down	1.68E-17
FABP3	13.543	18.173	2.598	3.493	0.203	67.272	78.133	85.953	97.919	0.39	3.116391	up	1.90E-42
SERINC2	1.761	1.695	1.951	0.682	1.747	2.465	3.095	7.156	2.402	3.432	1.242698	up	5.22E-09
GBP1	8.111	10.391	15.138	12.653	11.533	27.078	12.103	30.205	39.457	19.339	1.148335	up	4.35E-08
TRNP1	1.298	0.955	1.033	1.155	1.055	0.741	0.608	0.426	0.428	0.533	-1.00499	down	7.06E-05
TRIM63	1.622	1.584	1.214	2.149	1.629	0.673	0.313	0.338	0.986	1.348	-1.16313	down	7.37E-09
STMN1	5.07	4.523	8.573	3.977	4.092	13.831	8.847	49.549	29.669	12.85	2.128667	up	1.62E-23
PAQR7	24.346	28.129	11.665	9.039	4.872	45.803	30.536	126.88	67.37	15.928	1.876064	up	5.00E-18
IFI27L2	1.02	1.144	1.74	27.689	12.533	357.099	115.527	107.978	266.134	72.196	4.380105	up	4.10E-75
LCK	1.587	1.444	2.89	1.304	1.592	2.838	2.727	3.019	5.339	4.943	1.096993	up	2.39E-07
AZIN2	3.941	3.49	3.609	4.982	5.168	1.234	1.346	1.645	2.11	3.107	-1.1658	down	8.02E-09
MYCL	25.236	25.063	17.182	6.684	21.801	8.714	8.122	2.015	3.746	4.008	-1.85063	down	1.93E-18
GALE	203.78	179.069	256.203	94.255	87.192	53.449	31.172	18.142	17.67	260.946	-1.10527	down	5.90E-09
HMGCL	1321.124	1321.949	1041.953	914.18	1355.956	297.381	359.57	60.516	137.679	1292.717	-1.47124	down	5.19E-14
GRHL3	0.226	0.247	0.18	0.171	0.184	0.403	1.241	1.505	1.538	0.139	2.253688	up	1.78E-22
IL22RA1	1.048	0.851	0.331	0.475	0.408	5.144	1.585	2.564	4.447	2.369	2.369622	up	2.86E-24
CHEK1	1.146	0.734	1.54	0.373	0.613	2.773	2.202	2.824	1.682	0.894	1.234629	up	9.72E-09
KCNJ5	0.108	0.154	0.25	0.129	0.242	0.166	2.383	0.363	0.769	0.3	2.16631	up	2.98E-21
TMEM45L	0.867	1.134	1.972	3.459	2.645	0.759	0.612	0.315	0.255	2.977	-1.03417	down	3.22E-07
SCN3B	0.701	0.619	1.075	0.612	0.851	3.614	2.049	1.541	2.787	2.116	1.648644	up	7.08E-15
GRAMD1E	0.84	0.805	1.354	0.858	1.062	3.049	2.077	2.012	2.235	2.117	1.223104	up	4.73E-09
ZBTB32	25.252	27.628	28.602	8.546	14.028	4.861	7.698	7.297	6.856	12.33	-1.41415	down	6.15E-12
TAGLN	2.18	2.11	3.114	3.729	3.373	3.948	11.38	5.63	4.205	10.8	1.309567	up	1.26E-09
FXYD2	7.766	4.487	6.425	4.393	4.04	2.114	1.701	1.04	0.878	7.058	-1.08345	down	3.71E-07
MPZL3	0.236	0.336	0.631	0.229	0.396	0.795	0.65	0.857	0.407	1.014	1.024195	up	1.86E-05
MPZL2	0.597	0.53	0.725	0.468	0.643	2.053	1.023	1.207	1.407	1.5	1.277504	up	1.19E-08
LOC10174	1.523	1.158	0.721	0.072	0.888	0.081	0.41	0.032	0.121	0.267	-2.25322	down	1.56E-23
HYOU1	31.471	30.812	41.361	31.712	27.66	110.521	118.105	153.065	82.704	56.735	1.676599	up	9.89E-16

VWA5A2	1.704	1.799	0.955	1.884	1.702	1.564	0.595	0.527	0.437	0.892	-1.00161	down	5.88E-07
LOC1211C	0	0	0.072	0.374	3.318	0.037	2.02	0.372	1.026	7.847	1.584963	up	7.02E-12
LOC10174	0.02	0.013	0.032	0.083	1.055	0.114	1.404	0.208	1.115	1.668	1.901786	up	7.18E-15
LOC10705	0	0	0.044	0.036	0.484	0.088	0.295	0.146	0.355	1.047	1.766578	up	3.20E-12
PEAR1	0.597	0.886	0.858	0.622	0.745	1.049	1.511	2.859	0.844	1.851	1.128717	up	2.30E-07
SLAMF1	0.106	0.374	0.607	0.313	0.413	1.11	1.024	1.092	1.441	1.67	1.802586	up	2.87E-14
LOC10085	5.021	3.63	6.417	2.884	3.286	7.727	4.765	10.362	10.146	13.025	1.115587	up	1.42E-07
S100A6	1.272	2.011	4.726	3.571	0.98	3.674	11.08	6.875	7.546	5.013	1.44429	up	1.78E-10
CKS1B	1.373	1.796	3.682	5.166	4.156	5.243	8.042	7.273	9.947	7.406	1.228774	up	2.92E-08
FDPS	41.825	42.11	7.962	5.635	47.361	31.282	40.889	93.282	159.846	16.05	1.236231	up	5.76E-09
LOC1211C	0.115	0.075	0.214	0	0.118	0.186	0.173	0.279	0.486	2.684	2.855052	up	3.44E-18
CRP	3.37	6.5	5.968	5.443	7.638	10.493	7.381	13.386	10.25	16.434	1.002517	up	5.47E-06
TAGLN2	8.608	9.396	20.528	10.08	11.59	30.675	26.527	30.559	32.146	47.3	1.473675	up	2.14E-12
TMOD4	0.723	1.269	1.885	0.999	1.756	0.928	0.407	0.458	0.364	0.81	-1.1591	down	7.78E-07
LOC1211C	1.377	2.274	3.499	15.089	1.684	7.931	13.861	18.012	15.668	14.866	1.555707	up	6.25E-12
THEM4	1.379	1.504	1.63	1.751	3.047	2.055	0.188	0.127	0.231	0.206	-1.72811	down	7.56E-13
LOC1211C	1.707	2.146	2.676	0.723	3.534	2.064	0.6	0.906	0.57	1.183	-1.01816	down	4.03E-06
PFDN2	11.692	13.549	20.304	0.767	14.309	4.864	0.42	0.453	0.59	9.256	-1.9595	down	5.26E-20
TULP1	0.396	0.339	3.696	0.217	0.174	0.16	0.159	0.097	0.067	0.074	-3.10248	down	5.92E-33
FKBP5	111.755	99.425	223.982	47.184	74.273	38.026	39.52	37.085	38.927	55.699	-1.41139	down	3.04E-11
ELF3	1.802	1.886	5.136	1.871	1.286	16.166	9.234	21.922	10.085	3.758	2.351474	up	1.59E-28
CSRP1	1.668	1.898	3.368	2.702	4.32	18.671	27.775	3.576	9.065	7.147	2.246279	up	4.52E-26
UBE2T	0.274	0.426	0.464	0.084	0.633	0.967	0.231	0.685	1.165	1.182	1.167032	up	6.90E-06
KCNA3	0.153	0.305	0.746	0.993	0.514	0.556	1.007	1.969	1.585	1.702	1.329134	up	7.11E-10
NUAK2	0.135	0.399	0.658	0.145	0.237	1.889	0.944	1.248	0.707	0.788	1.821512	up	8.36E-16
LOC1211C	2.336	2.667	13.532	2.99	5.007	1.804	1.802	1.746	0.564	1.728	-1.79466	down	7.54E-16
SLC45A3	1.655	1.516	4.072	1.441	3.063	0.645	1.059	0.8	0.683	0.893	-1.5245	down	2.50E-12
SLC26A9	0.066	0.086	0.01	0.003	0	0.986	6.569	6.993	3.939	0.019	6.766704	up	#######
C1orf186	0.024	0.101	0.122	0.087	0.049	0.276	1.315	1.397	0.215	0.082	3.083959	up	2.07E-32
RAB7B	0.022	0.035	0.129	0.045	0	0.149	1.365	0.9	0.429	0.246	3.712614	up	5.70E-42
FAM72A	0.454	0.348	0.628	0.421	1.013	1.399	0.754	1.638	3.089	2.41	1.695907	up	9.76E-14
LAMB3	0.296	0.278	0.164	0.519	0.172	2.731	3.113	1.878	0.973	0.755	2.721032	up	1.21E-32
G0S2	17.283	17.63	33.545	71.809	20.964	262.247	366.83	247.575	35.713	47.012	2.572931	up	1.59E-32
GUCA1A	0.963	0.641	1.561	0.964	0.575	0.438	0.236	0.372	0.346	0.707	-1.16229	down	2.46E-07
WNT2B	0.194	0.178	0.433	0.59	0.479	1.315	1.55	2.225	0.23	1.378	1.834842	up	4.97E-15
MOV10	20.693	22.727	15.294	15.974	9.551	40.236	37.529	58.112	50.438	17.159	1.272234	up	2.14E-09
SLC16A1	80.737	81.864	85.098	65.917	80.697	23.397	20.058	27.085	19.215	45.98	-1.53851	down	3.51E-14
OLFML3	0.876	0.75	0.59	0.277	0.658	0.707	1.326	2.596	0.64	1.894	1.183473	up	8.62E-07

PACSIN1	3.057	2.979	2.103	5.131	1.149	9.104	7.941	9.812	8.665	0.833	1.333881	up	6.91E-10
TSPO2	1.038	0.781	1.984	0.782	0.498	0.502	0.428	0.252	0.151	0.273	-1.65914	down	3.88E-12
LOC76878	0.737	0.599	0.428	1.424	0.491	1.719	5.621	0.217	1.623	1.119	1.48386	up	5.87E-11
LOC10705	1.478	1.284	2.353	0.381	0.126	0.117	0.147	0.281	0.361	0.293	-2.22453	down	1.09E-22
LOC10704	1.069	1.001	1.257	0.426	0.349	0.104	0.309	0.265	0.41	0.543	-1.32791	down	9.85E-10
VWA5A1	3.4	3.36	1.917	2.377	3.428	1.368	1.09	1.123	1.014	1.895	-1.15736	down	6.84E-09
MRC2	0.907	1.665	5.262	2.221	1.584	9.852	1.373	6.1	3.367	3.671	1.065401	up	1.43E-07
SLC4A1	2.92	2.766	7.813	0.831	2.847	0.398	0.472	0.484	0.592	1.143	-2.47335	down	1.86E-28
COL1A1	0.472	0.573	0.814	0.776	1.076	1.572	1.564	2.853	1.943	2.361	1.470542	up	8.74E-12
SLC35B1	36.478	35.208	36.24	31.353	29.908	71.09	78.768	90.424	60.114	44.899	1.029189	up	7.66E-07
LOC42978	1.094	0.858	0.37	0.95	0.371	0.358	0.151	0.363	0.384	0.437	-1.10327	down	9.47E-08
C27H17Ol	11.499	8.589	25.485	14.803	13.014	6.113	2.918	2.592	3.14	10.13	-1.55965	down	1.17E-13
TBX21	1.399	1.101	1.808	1.033	1.479	1.896	1.915	2.533	3.866	4.177	1.076366	up	9.07E-07
IKZF3	0.56	0.562	0.882	0.502	0.505	1.048	0.559	0.984	1.574	1.894	1.007633	up	3.96E-06
CSF3	0.957	1.356	2.006	1.048	0.786	1.826	7.925	1.992	0.606	0.817	1.096831	up	1.99E-07
RARA	10.973	12.41	15.375	11.101	11.083	20.898	26.643	47.225	37.125	10.403	1.223298	up	2.87E-09
TOP2A	0.342	0.255	0.321	0.388	0.281	1.472	0.854	1.771	3.94	2.359	2.70781	up	2.56E-32
CCR7	0.262	0.268	0.371	0.206	0.549	1.192	0.703	0.607	1.952	1.748	1.901844	up	9.07E-17
KRT40	0.232	0.182	0.664	0.476	0.286	1.102	1.85	0.787	0.312	1.439	1.574499	up	4.74E-11
KRT24	1.121	0.885	1.169	0.46	1.964	2.928	2.693	3.186	1.926	4.97	1.486971	up	1.30E-11
KRT10	0.362	0.403	0.663	0.461	0.465	0.935	0.977	1.472	1.404	3.249	1.769379	up	3.86E-14
HSPB9	0.747	0.543	1.124	0.444	2.687	4.553	1.961	1.334	2.76	0.859	1.047557	up	1.93E-06
VAT1	115.805	120.68	33.306	87.503	40.757	36.262	25.835	31.587	62.992	31.645	-1.07974	down	1.70E-08
RND2	2.62	1.917	1.709	1.848	1.823	1.031	0.371	0.313	0.457	1.241	-1.53748	down	5.98E-13
LOC10175	3.827	4.542	4.63	3.06	0.899	4.94	6.371	11.567	7.992	17.997	1.526617	up	3.28E-12
LOC10085	11.151	12.064	21.638	20.943	25.843	20.96	37.113	79.227	54.261	67.268	1.497915	up	1.02E-12
ANGPTL4	12.453	12.116	22.343	19.188	6.518	68.685	140.933	75.861	11.894	6.897	2.066877	up	2.24E-23
C19orf71	0.377	0.308	0.038	0.039	1.017	0.076	0.053	0.029	0.075	0.093	-2.43021	down	2.30E-09
LOC10085	0.669	0.945	2.285	1.243	0.617	3.048	2.005	3.232	1.702	2.541	1.12059	up	7.37E-08
NMRK2	0.481	0.525	1.118	0.461	0.535	1.132	0.676	1.969	2.189	1.779	1.31034	up	4.92E-08
ACSBG2	14.586	15.643	11.837	8.17	13.21	150.11	328.688	327.719	249.717	14.661	4.077037	up	4.60E-70
ZAP70	1.62	1.196	2.877	1.026	1.344	2.829	2.023	2.524	4.681	4.762	1.060238	up	5.21E-07
MEX3D	0.596	0.738	0.777	0.636	0.69	1.278	1.54	3.652	1.625	1.062	1.412415	up	8.41E-11
PLIN5	1.736	1.151	3.409	2.248	0.391	6.98	3.877	1.197	4.233	1.594	1.000484	up	2.21E-06
LRG1	0.422	0.461	0.359	0.44	0.984	1.476	1.172	1.127	2.251	1.971	1.582981	up	3.94E-11
CREB3L3	159.827	156.871	111.394	114.31	126.707	42.107	23.945	23.121	31.261	127.309	-1.43338	down	1.66E-13
MIDN	1.534	1.929	5.572	2.288	1.636	5.522	7.55	7.033	4.842	2.333	1.073597	up	4.90E-08
LOC11253	0.265	0.267	0.78	0.285	0.231	0.79	1.285	0.854	0.662	0.382	1.117836	up	8.87E-07

ADAMTSL	0.983	1.337	0.423	0.162	2.564	0.079	0.331	0.036	0.031	0.116	-3.19438	down	1.77E-32
LOC10085	1.85	1.341	1.179	1.31	0.877	0.344	0.512	0.725	0.135	0.814	-1.37215	down	1.97E-09
LOC42010	3.702	4.864	6.514	4.686	2.361	15.263	4.834	22.937	17.291	6.68	1.598243	up	7.19E-14
LOC10705	57.522	65.076	78.023	71.484	31.665	187.848	57.045	257.873	251.174	80.709	1.458175	up	6.53E-12
TMEM221	4.372	4.066	4.68	2.364	2.874	5.926	9.376	13.213	6.437	3.6	1.070348	up	1.72E-07
LOC10705	6.294	8.386	4.573	4.719	3.256	1.778	2.738	1.281	1.963	2.807	-1.36511	down	9.74E-11
LOC12110	0.686	0.658	0.66	1.216	0.474	3.053	2.528	3.832	3.389	2.869	2.08335	up	1.77E-20
CALR	241.781	241.895	342.532	279.632	306.725	705.202	786.033	951.987	576.401	496.13	1.315513	up	1.93E-10
LOC76984	32.556	33.04	8.417	20.533	10.931	6.221	15.854	7.113	8.477	14.518	-1.01521	down	1.40E-07
CD163	11.149	13.447	5.876	14.179	56.183	44.72	43.367	51.738	57.966	105.311	1.587773	up	1.50E-13
LOC10175	1.051	0.647	0.043	0.034	0.021	0	0.054	0.004	0	0.047	-4.03322	down	4.64E-45
LOC10175	0.026	0.009	0.166	0.053	0	0.335	0.278	0.284	1.409	0.615	3.497906	up	1.39E-35
LOC10704	0.009	0.006	0.5	1.416	0.047	0.285	0.021	0.014	0	0	-2.60917	down	8.83E-29
LOC10704	0.168	0.184	0.805	0.438	2.796	0.113	0.158	0.238	0.089	0.443	-2.07131	down	1.96E-14
CHIR-AB1	1.081	1.29	0.253	0.444	0.163	0.385	2.18	0.617	5.081	2.757	1.768495	up	7.73E-13
LOC10705	1.023	2.554	3.072	1.757	5.92	0.562	0.366	1.973	0.331	1.787	-1.51223	down	6.15E-11
LOC11253	0.152	0.376	0.701	0.57	0.122	0.464	0.038	0.698	1.606	1.201	1.058714	up	1.13E-05
LOC12110	0.45	0.741	0.532	1.811	0.606	1.59	5.201	4.716	0.786	0.459	1.621845	up	1.61E-13
LOC11253	0.109	0	0.414	0.359	0.477	0.331	0.031	2.403	0.151	0.108	1.150998	up	9.97E-07
LOC10704	0.052	0	0.416	0.408	0.536	0.358	0.47	0.76	3.181	0.772	1.968608	up	9.68E-16
LOC11253	0.417	0.26	6.651	2.071	3.179	0.24	0.602	0.337	1.931	2.229	-1.23548	down	7.00E-08
LOC43106	0.041	0.134	0.697	0.337	0.38	1.401	0.015	1.444	0.227	0.877	1.316124	up	4.63E-09
CHIR-AB-	0.641	0.668	0.557	0.455	0.509	1.081	0.328	2.811	1.499	1.149	1.277591	up	8.35E-08
LOC10705	1.089	0.786	1.705	1.358	0.231	1.666	0.38	3.826	2.288	3.332	1.151905	up	8.68E-07
LOC11253	0.05	0.105	0.97	0.55	1.303	0.153	0.101	0.176	0.601	0.325	-1.1321	down	1.92E-06
LOC12110	0.657	0.706	0.224	0	0.145	0	0.053	0.057	0.854	2.543	1.015695	up	0.003117
FKBP11	17.495	22.133	21.819	27.326	17.85	43.392	45.461	61.082	45.694	39.453	1.140608	up	8.92E-08
METTL7A	142.141	140.895	176.986	159.581	210.518	67.374	63.172	70.91	71.063	119.741	-1.0815	down	7.18E-08
LOC10704	0.392	0.257	0.769	0.198	0.434	0.924	0.78	0.85	1.05	1.754	1.383902	up	8.29E-10
STAT2	5.504	5.392	6.718	5.234	3.53	13.563	10.487	17.022	14.494	6.061	1.224071	up	4.95E-09
APOF	37.366	50.766	36.826	63.533	23.793	100.514	91.981	156.486	139.944	63.975	1.381002	up	1.38E-10
LOC10085	24.632	27.589	59.496	64.047	59.54	16.948	17.133	33.077	16.214	21.299	-1.16863	down	1.83E-08
TAC3	0.091	0.208	1.231	0.99	1.378	0.055	0	0.234	0.09	0.269	-2.57943	down	5.70E-21
INHBE	4.017	4.779	3.518	12.377	2.036	22.969	5.71	4.197	17.131	14.947	1.280962	up	2.27E-08
KRT7	0.635	0.432	0.738	1.138	0.496	1.434	1.242	1.486	1.025	2.115	1.085194	up	2.06E-06
LOC12110	0	0	0.094	0	0	0	1.064	0.048	0.811	0.039	4.312425	up	6.35E-20
LOC12110	0.594	1.033	0.032	0	0	0	0.091	0.025	0.214	0.133	-1.83007	down	2.03E-13
OTX5	3.385	3.379	6.24	1.429	2.499	0.726	0.419	0.439	2.326	4.53	-1.00401	down	5.45E-07

LOC42622	85.864	97.813	72.44	292.66	10.808	454.761	368.059	101.555	288.823	202.936	1.339521	up	1.53E-09
LOC1211C	2.846	2.578	2.049	1.574	2.642	2.03	0.554	1.427	0.606	1.025	-1.05021	down	4.21E-06
LOC1211C	0.063	0.064	0.521	0.104	0.253	0.051	0.229	4.981	0.885	0.263	2.666869	up	6.90E-34
ALPK2	1.282	0.914	3.265	0.609	0.412	0.083	0.082	0.07	0.399	0.108	-3.11837	down	3.97E-39
ENHO	11.656	11.328	6.264	7.549	2.636	22.431	39.475	42.317	13.13	5.011	1.63358	up	1.58E-14
ARID3C	2.61	2.312	1.969	1.971	1.86	5.518	7.512	7.164	5.158	2.979	1.401389	up	4.00E-11
CCL19	1.967	1.859	6.144	5.12	2.874	10.663	10.076	5.872	15.028	17.343	1.714887	up	9.64E-16
LOC10085	2.842	2.447	3.327	2.85	2.704	13.747	7.306	7.715	27.291	22.178	2.464595	up	8.71E-29
AVD	0.679	0.578	0.703	2.345	0.488	1.862	1.375	1.606	3.493	3.95	1.357097	up	2.49E-07
NPR3	0.084	0.139	0.074	0.152	0.188	1.419	0.185	0.495	0.574	0.295	2.211274	up	1.06E-22
NIM1K	237.897	193.624	200.797	136.927	290.768	43.697	79.275	49.994	107.587	149.373	-1.30191	down	1.22E-10
HMGCS1	1102.285	1000.148	825.11	665.005	1477.042	207.1	375.858	241.968	540.893	623.999	-1.34923	down	2.72E-11
S100Z	2.605	2.791	0.713	2.97	4.582	1.129	0.696	1.135	1.61	0.754	-1.35865	down	7.61E-11
HMGCR	31.138	34.359	11.575	7.257	41.515	28.551	39.081	67.587	109.179	11.803	1.025611	up	9.07E-07
ARHGEF28	0.245	0.239	0.654	0.201	0.474	0.875	0.454	0.615	1.018	1.018	1.132228	up	7.43E-08
SLC1A1	14.203	12.698	16.007	14.712	14.135	9.271	4.424	5.403	5.622	5.192	-1.26221	down	6.08E-10
GLDC	69.204	66.091	99.52	67.77	72.787	172.195	254.043	213.55	105.607	76.754	1.131068	up	1.96E-08
HACD4	0.264	0.28	0.149	0.46	0.316	1.567	0.247	0.393	0.365	0.39	1.009268	up	4.25E-05
FBP2	3.111	2.678	4.789	5.166	1.373	0.488	0.775	0.266	0.397	0.7	-2.70217	down	7.34E-32
GADD45G	9.213	10.267	11.004	14.629	6.816	79.066	197.59	92.548	72.393	15.852	3.138875	up	1.77E-45
TPPP2	1.417	1.78	1.445	2.702	3.261	0.32	1.402	0.653	0.09	2.626	-1.05799	down	2.15E-06
PPIP5K2	14.486	14.036	15.709	20.013	19.902	6.583	6.67	5.444	6.714	11.962	-1.17079	down	4.32E-09
SHB	3.992	3.684	5.439	2.551	2.615	30.292	2.944	36.582	2.97	3.52	2.061189	up	4.01E-22
TRIM14	11.323	11.023	2.406	3.108	8.678	2.594	2.209	1.281	1.982	9.126	-1.08744	down	8.58E-09
PCGF3	7.783	6.775	5.021	7.649	2.945	12.864	17.144	18.87	20.462	3.917	1.279568	up	1.57E-09
MFSD7	1.309	1.008	2.038	1.828	1.356	2.802	3.946	5.021	4.509	1.37	1.226512	up	3.37E-09
LPL	1.707	1.894	5.175	0.471	5.173	0.111	0.083	0.739	0.067	0.261	-3.51022	down	7.01E-48
PSD3	7.349	6.967	7.679	4.194	8.275	1.064	1.563	1.964	1.137	4.858	-1.70246	down	7.20E-17
RHOBTB3	2.66	2.558	3.444	3.373	4.449	1.231	2.758	0.57	0.7	1.941	-1.19443	down	1.28E-08
ALDOB	11839.59	10520.93	7994.081	8633.508	7428.55	3176.009	3960.248	2516.6	2459.889	7267.715	-1.26004	down	1.55E-10
KIAA1958	30.304	27.952	9.523	15.084	9.131	69.791	54.221	69.576	52.158	26.463	1.565051	up	5.52E-13
SMC2	0.127	0.124	0.223	0.357	0.39	0.985	0.356	0.894	1.812	1.061	2.060211	up	5.26E-19
LPAR1	0.395	0.296	0.131	0.359	0.11	0.969	1.795	1.827	0.202	0.3	1.975866	up	5.66E-18
CDKN2A	0.782	0.648	0.831	0.964	1.152	4.331	1.7	3.352	2.847	2.801	1.778759	up	6.04E-15
CDKN2B	0.209	0.561	0.183	0.567	0.773	2.277	3.668	3.132	2.383	0.247	2.349537	up	6.03E-24
TRIM36	0.728	0.511	0.826	1.005	0.549	2.141	3.377	2.225	2.37	1.32	1.65818	up	9.19E-15
CCDC112	2.162	1.871	1.46	1.92	1.839	0.599	0.393	0.649	1.071	0.925	-1.34581	down	4.15E-10
LOC10705	14.178	14.894	14.025	12.341	21.783	38.804	39.156	45.915	23.351	23.737	1.146568	up	3.90E-08

CHIR-IG1-	1.543	1.607	2.131	4.347	4.334	3.224	1.912	7.084	9.641	13.997	1.360474	up	1.59E-09
LOC10705	0.256	0.28	1.429	1.569	1.434	0.161	0.096	0.173	0.135	0.449	-2.28696	down	3.69E-18
LOC42523	0.597	0.538	1.462	1.46	1.855	0.317	0.336	0.402	0.392	1.012	-1.26386	down	2.02E-09
LOC12110	0.014	0.009	1.624	1.345	1.382	0	1.372	0	0	0	-1.66907	down	6.15E-11
CDIPT	3.413	4.386	3.397	4.007	3.005	9.976	8.018	10.607	13.068	5.286	1.366464	up	7.15E-10
LOC12110	0.373	0.929	0.476	0.859	2.072	0.663	0.504	0.045	0.236	0.221	-1.49365	down	7.44E-07
LOC12110	0.553	0.324	0.44	0.215	0.538	0.774	1.341	0.3	1.45	1.147	1.273714	up	2.34E-07
LOC12110	0.534	1.017	0.426	4.591	1.247	0.118	0.437	0.412	0.538	0	-2.37262	down	4.42E-19
LOC12110	0.396	0.408	0.767	0.295	0.252	0.381	1.316	0.206	1.195	1.507	1.118662	up	1.71E-06
LOC10705	0.145	0.228	0.255	0.096	0.478	0.235	0.523	0.229	1.059	1.004	1.339747	up	7.83E-07
LOC12110	0.106	0.092	0.421	0.029	0.362	0.057	0.238	0.085	1.059	0.764	1.12126	up	9.01E-05
LOC12110	1.085	0.986	3.121	0.05	0.681	0.34	0.271	0.11	1.145	0.297	-1.45118	down	4.68E-07
LOC12110	2.154	2.677	4.031	2.522	3.319	2.822	0.787	0.816	1.065	1.486	-1.0751	down	1.94E-06
LOC12110	1.881	1.885	2.629	1.665	1.527	0.583	0.917	0.845	0.51	1.297	-1.20629	down	1.44E-08
LOC11253	2.155	2.053	0.757	0.13	4.036	0.92	1.425	0.48	0.025	1.187	-1.17649	down	2.05E-07
LOC10705	0.452	0.371	1.943	1.664	2.801	0.772	0.171	0.976	0.276	0.522	-1.41053	down	4.52E-11
LOC12110	1.528	1.676	3.059	0.441	6.025	7.894	5.62	2.605	6.406	5.13	1.119115	up	8.98E-08
LOC10705	0.496	0.46	0.513	0.25	0.032	1.913	1.547	0.076	1.765	0.478	1.719775	up	1.42E-15
MHCY13	3.428	2.686	3.329	0.991	1.896	0.638	0.508	0.959	1.728	0.668	-1.45284	down	2.87E-10
LOC11253	2.993	2.829	7.512	3.722	2.373	1.698	1.075	0.863	0.63	1.267	-1.81115	down	5.75E-17
LOC12110	15.418	15.792	19.848	25.319	21.906	10.589	6.201	8.25	9.039	7.973	-1.22467	down	1.89E-09

qvalue	function
5.19E-09	LOC112532872; C-type lectin domain family 2 member D-like isoform X3
2.01E-26	LOC121113330; endogenous retrovirus group K member 8 Gag polyprotein-like
1.62E-07	GOLGB1; golgin subfamily B member 1 isoform X2
1.08E-43	ELAPOR2; endosome/lysosome-associated apoptosis and autophagy regulator family member 2 isoform X1
3.61E-17	ALG12; dol-P-Man:Man(7)GlcNAc(2)-PP-Dol alpha-1,6-mannosyltransferase isoform X1
1.39E-23	CRELD2; protein disulfide isomerase CRELD2 precursor
#######	LOC429348; tetraspanin-7-like
8.07E-16	TRABD; traB domain-containing protein isoform X1
4.94E-07	IQUB; IQ and ubiquitin-like domain-containing protein isoform X4
1.38E-15	CPED1; cadherin-like and PC-esterase domain-containing protein 1 isoform X4
3.31E-07	CAV1; caveolin-1
3.00E-08	AMIGO2; amphoterin-induced protein 2 precursor
4.60E-19	SRGAP1; SLIT-ROBO Rho GTPase-activating protein 1 isoform X2
1.17E-10	IFNG; interferon gamma precursor
2.29E-09	TSPAN8; tetraspanin-8
1.47E-07	LUM; lumican isoform X1
6.19E-08	IKBIP; inhibitor of nuclear factor kappa-B kinase-interacting protein isoform X1
4.69E-17	OC3; uncharacterized protein LOC417943 precursor
3.17E-05	HIST1H2A4L3; histone H2A-III
1.70E-14	LOC121106438; histone H3
0.001114	LOC100857439; histone H3
1.08E-07	HIST1H2B7L4; histone H2B 1/2/3/4/6
1.55E-07	GUCY2C; heat-stable enterotoxin receptor isoform X3
7.57E-15	GBE; eye-globin isoform X1
1.36E-12	LOC417973; uncharacterized protein LOC417973 isoform X3
2.90E-11	MEI1; meiosis inhibitor protein 1 isoform X12
1.44E-06	ADSL; adenylosuccinate lyase
6.18E-44	NPTXR; neuronal pentraxin receptor
2.28E-05	DNAL4; dynein light chain 4, axonemal
2.33E-23	FAM20CL; extracellular serine/threonine protein kinase FAM20C isoform X1
3.49E-25	SUN2; SUN domain-containing protein 2
1.16E-05	CBY1; protein chibby homolog 1
7.73E-07	LGALS1A; beta-galactoside-binding lectin
3.86E-06	CARD10; caspase recruitment domain-containing protein 10 isoform X7
5.53E-05	CSF2RB; cytokine receptor common subunit beta isoform X1
5.17E-08	MCM5; DNA replication licensing factor MCM5

8.96E-22 HSP90B1; endoplasmin precursor
3.42E-14 ASCL1; achaete-scute homolog 1
1.37E-23 SVOPL; putative transporter SVOPL isoform X3
3.20E-09 CGTL; cystine/glutamate transporter isoform X4
4.71E-12 MICAL3; F-actin-monooxygenase MICAL3 isoform X1
1.77E-13 USP41; ubl carboxyl-terminal hydrolase 18 isoform X1
2.47E-12 AKR1E2; aldo-keto reductase family 1, member B1-like
1.67E-21 LOC425137; aldo-keto reductase family 1, member B1-like isoform X3
3.34E-07 EPS8; epidermal growth factor receptor kinase substrate 8 isoform X1
3.75E-45 SLCO1B1; solute carrier organic anion transporter family member 1C1 isoform X1
1.72E-09 SOX5; transcription factor SOX-5 isoform X14
1.37E-18 LDHB; L-lactate dehydrogenase B chain isoform X1
6.76E-20 SSPN; sarcospan isoform X1
3.54E-22 BHLHE41; class E basic helix-loop-helix protein 41
1.12E-09 ENDOUL; poly(U)-specific endoribonuclease-A isoform X1
6.00E-08 MPPED1; metallophosphoesterase domain-containing protein 1
2.59E-20 SULT4A1; sulfotransferase 4A1 isoform X2
3.39E-26 PNPLA3; patatin-like phospholipase domain-containing protein 2 isoform X3
3.59E-06 PARVG; gamma-parvin
3.56E-07 KIAA0930; uncharacterized protein KIAA0930 homolog isoform X3
4.74E-10 MANSC1; MANSC domain-containing protein 1 precursor
7.99E-10 A2ML3; alpha-2-macroglobulin-like isoform X6
5.14E-08 CASR; extracellular calcium-sensing receptor
1.57E-08 CNP1; C-type natriuretic peptide 1 precursor
2.81E-20 CDCA3; cell division cycle-associated protein 3 isoform 2
4.19E-08 LRRC23; leucine-rich repeat-containing protein 23 isoform X1
3.99E-19 EPHA1; ephrin type-A receptor 1 precursor
3.92E-11 GSTK1; glutathione S-transferase kappa 1 isoform X1
1.80E-14 KEL; kell blood group glycoprotein isoform X4
8.92E-08 LOC100859872; uncharacterized protein LOC100859872 isoform X1
1.93E-11 CLDND1; claudin domain-containing protein 1 isoform X2
4.33E-12 APOV1; apovitellenin-1 precursor
1.86E-05 DPT; dermatopontin
2.68E-07 LOC418414; uncharacterized protein C3orf85 homolog
2.12E-26 CD200L; uncharacterized protein LOC418424 precursor
3.74E-06 CREG1; protein CREG1 precursor
3.07E-06 BCL9; B-cell CLL/lymphoma 9 protein isoform X2
4.53E-10 ADAMTS1; LOW QUALITY PROTEIN: A disintegrin and metalloproteinase with thrombospondin motifs 1 isoform X1

6.42E-24 MAP3K7CL; MAP3K7 C-terminal-like protein isoform X4
9.16E-19 HUNK; hormonally up-regulated neu tumor-associated kinase isoform X1
6.26E-11 EVA1C; protein eva-1 homolog C isoform X5
1.72E-06 GART-B; trifunctional purine biosynthetic protein adenosine-3 isoform X2
1.19E-13 PCP4; calmodulin regulator protein PCP4
2.91E-06 BACE2; beta-secretase 2 isoform X2
9.93E-36 MX1; interferon-induced GTP-binding protein Mx isoform X3
1.18E-06 SIK1; serine/threonine-protein kinase SIK2
1.11E-07 MAOB; amine oxidase [flavin-containing] B
1.46E-45 MID1IP1; mid1-interacting protein 1
1.73E-11 SLC51AL; organic solute transporter subunit alpha
3.25E-27 MAP3K15; mitogen-activated protein kinase kinase kinase 15
8.96E-26 PDHA2; pyruvate dehydrogenase E1 component subunit alpha, somatic form, mitochondrial precursor
7.70E-42 ADGRG2; adhesion G-protein coupled receptor G2 isoform X1
2.26E-23 GRPR; gastrin-releasing peptide receptor
2.78E-38 LOC121106447; uncharacterized protein LOC121106447
6.53E-35 LOC107052718; LOW QUALITY PROTEIN: uncharacterized protein LOC107052718
2.62E-11 LOC418658; arylsulfatase D isoform X2
5.98E-06 SLC25A6; ADP/ATP translocase 3
1.82E-23 LYGL; lysozyme g isoform X1
4.75E-08 RNF149; E3 ubiquitin-protein ligase RNF149 isoform X1
4.70E-11 IL1RL1; interleukin-1 receptor-like 1 isoform LV precursor
5.32E-06 IL18R1; interleukin-18 receptor 1 isoform X2
7.64E-05 FHL2; four and a half LIM domains protein 2 isoform X2
1.56E-11 GRTP1; growth hormone-regulated TBC protein 1
4.91E-08 RAB20; ras-related protein Rab-20
7.69E-20 LOC121107687; SPARC
2.40E-12 HS6ST3; heparan-sulfate 6-O-sulfotransferase 3
2.05E-09 DCT; L-dopachrome tautomerase precursor
3.46E-10 ACOD1; cis-aconitate decarboxylase
2.62E-09 EPSTI1; epithelial-stromal interaction protein 1
1.80E-09 LPAR6; lysophosphatidic acid receptor 6
4.74E-06 PHF11; PHD finger protein 11 isoform X1
5.66E-11 CKAP2; cytoskeleton-associated protein 2
4.78E-07 POSTN; periostin isoform X14
3.89E-07 LOC121106501; uncharacterized protein LOC121106501 isoform X2
1.11E-06 HSPH1; heat shock protein 105 kDa
5.71E-05 RASL11A; ras-like protein family member 11A

7.00E-07 SKA3; spindle and kinetochore-associated protein 3 isoform X1
3.13E-08 ACAT1; acetyl-CoA acetyltransferase, mitochondrial
2.49E-05 GUCY1A2; guanylate cyclase soluble subunit alpha-2
5.58E-22 MMP7; matrilysin precursor
8.29E-05 FUT4; alpha-(1,3)-fucosyltransferase 4
2.81E-06 LOC121107447; uncharacterized protein LOC121107447 isoform X1
5.90E-10 LOC101748650; thyroid hormone-inducible hepatic protein-like
2.29E-09 THRSPB; thyroid hormone responsive spot 14 beta
1.06E-08 AQP11; aquaporin-11
2.58E-05 TSKU; tsukushin precursor
9.39E-07 WNT11; protein Wnt-11 isoform X4
7.88E-05 IL18BP; interleukin-18-binding protein isoform X2
2.18E-09 LOC107052453; leucine-rich repeat-containing protein 51-like
8.03E-11 LOC121109083; glutamine-rich protein 2-like isoform X1
4.38E-24 ADAM15; disintegrin and metalloproteinase domain-containing protein 9 isoform X3
1.76E-06 P2RY6; P2Y purinoceptor 3
6.85E-54 HBE; hemoglobin subunit epsilon
3.29E-33 HBBA; hemoglobin subunit beta
1.08E-44 HBE1; hemoglobin subunit epsilon 1
1.48E-06 TAF10; transcription initiation factor TFIID subunit 10
1.09E-14 TPP1; tripeptidyl-peptidase 1
6.97E-08 GIMAP8; GTPase IMAP family member 8 isoform X1
2.97E-07 SCAP; sterol regulatory element-binding protein cleavage-activating protein isoform X2
2.40E-24 ACKR2; atypical chemokine receptor 2
4.26E-29 CYP8B1; cytochrome P450, family 8, subfamily B
6.75E-08 VILL; villin-like protein isoform X2
3.34E-15 DLEC1; deleted in lung and esophageal cancer protein 1 isoform X3
3.47E-19 ACAA1; 3-ketoacyl-CoA thiolase, peroxisomal
1.19E-07 PTCHD3; patched domain-containing protein 3
2.95E-07 APBB1IP; amyloid beta A4 precursor protein-binding family B member 1-interacting protein
2.17E-44 NELL3; uncharacterized protein NELL3
1.43E-12 RSU1; ras suppressor protein 1
1.20E-07 ACBD7; acyl-CoA-binding domain-containing protein 7 isoform X2
1.43E-08 OLAH; S-acyl fatty acid synthase thioesterase, medium chain isoform X1
1.47E-21 ABCB1LA; phosphatidylcholine translocator ABCB4 isoform X5
1.55E-07 CYP51A1; leucine-rich repeat and death domain-containing protein 1 isoform X2
2.27E-17 CDK6; cyclin-dependent kinase 6
8.46E-08 SAMD9L; sterile alpha motif domain-containing protein 9-like

1.87E-08	COL1A2; collagen alpha-2(I) chain precursor
3.31E-07	LOC395159; Schwann cell-specific EGF-like repeat autocrine factor precursor
1.62E-05	AGR2; anterior gradient protein 2 homolog
2.55E-11	HDAC9; histone deacetylase 9 isoform X11
1.40E-07	IGF2BP3; insulin-like growth factor 2 mRNA-binding protein 3
7.32E-07	FKBP14; peptidyl-prolyl cis-trans isomerase FKBP14
8.48E-16	SATB1; DNA-binding protein SATB1 isoform X1
1.22E-08	EOMES; eomesodermin homolog isoform X1
1.14E-10	LOC121106513; protein TOPAZ1-like
8.31E-11	ACAD11; acyl-CoA dehydrogenase family member 11
0.000133	TGM4; protein-glutamine gamma-glutamyltransferase 4
3.18E-17	TRANK1; TPR and ankyrin repeat-containing protein 1 isoform X2
0.001014	LOC121109702; uncharacterized protein LOC121109702
9.64E-12	INHBA; inhibin beta A chain isoform X1
1.94E-06	PDIA4; protein disulfide-isomerase A4 isoform X1
4.76E-16	KCNG2; potassium voltage-gated channel subfamily G member 2 isoform X1
3.89E-16	DCDC2; doublecortin domain-containing protein 2 isoform X4
6.98E-18	NRSN1; neurensin-1
3.60E-08	ID4; DNA-binding protein inhibitor ID-4
3.99E-14	ELOVL2; elongation of very long chain fatty acids protein 2
5.90E-15	SYCP2L; synaptonemal complex protein 2-like isoform X3
3.58E-06	TXNDC5; thioredoxin domain-containing protein 5 isoform X2
6.47E-07	F13A1; coagulation factor XIII A chain
3.50E-16	ECI2; enoyl-CoA delta isomerase 2 isoform X3
4.94E-09	SERPINB1; leukocyte elastase inhibitor isoform X1
2.32E-16	SERPINB5; serpin B5
1.34E-05	LOC420903; MARVEL domain-containing protein 3 isoform X2
1.22E-10	CMBL; carboxymethylenebutenolidase homolog
2.56E-14	SRD5A1; 3-oxo-5-alpha-steroid 4-dehydrogenase 1 isoform X3
8.29E-06	MOCOS; molybdenum cofactor sulfurase isoform X1
4.87E-10	SEC61B; protein transport protein Sec61 subunit beta
2.21E-84	IFI6; interferon alpha inducible protein 6
1.48E-11	GLIPR2; Golgi-associated plant pathogenesis-related protein 1
3.12E-10	ZNF516; zinc finger protein 516 isoform X2
1.64E-56	FAM69C; divergent protein kinase domain 1C
#######	C18orf63; uncharacterized protein C18orf63 homolog isoform X3
2.55E-06	FBXO15; F-box only protein 15 isoform X6
1.01E-06	CIDEA; cell death activator CIDE-A

4.57E-10 MTCL1; microtubule cross-linking factor 1 isoform X7
1.69E-06 MYOM1; myomesin-1
5.21E-08 LPIN2; phosphatidate phosphatase LPIN2 isoform X1
8.90E-14 COLEC12; collectin-12
3.53E-21 ABHD3; phospholipase ABHD3 isoform X1
1.30E-05 LAMA3; LOW QUALITY PROTEIN: laminin subunit alpha-3 isoform X1
3.29E-86 DSC1; desmocollin-2 isoform X4
1.06E-06 MCM4; DNA replication licensing factor MCM4
2.51E-10 C8orf22; pancreatic progenitor cell differentiation and proliferation factor-like protein
2.36E-08 FAM110B; protein FAM110B isoform X1
5.20E-36 CYP7A1; cholesterol 7-alpha-monooxygenase
9.02E-08 XKR9; XK-related protein 9 isoform X2
2.18E-07 MSC; musculin
1.34E-08 GDAP1; ganglioside-induced differentiation-associated protein 1 isoform X1
1.58E-11 FABP4; fatty acid-binding protein, adipocyte
5.33E-10 CA13; carbonic anhydrase 13
6.04E-08 ATP6V0D2; V-type proton ATPase subunit d 2
1.15E-06 WWP1; NEDD4-like E3 ubiquitin-protein ligase WWP1 isoform X3
7.45E-11 PTDSS1; phosphatidylserine synthase 1 isoform X1
8.32E-07 CPQ; carboxypeptidase Q isoform X1
8.13E-10 NCALD; neurocalcin-delta
1.88E-09 RIMS2; regulating synaptic membrane exocytosis protein 2 isoform X50
4.18E-07 DCSTAMP; LOW QUALITY PROTEIN: dendritic cell-specific transmembrane protein
4.27E-10 EBAG9; receptor-binding cancer antigen expressed on SiSo cells isoform X2
1.28E-06 SAMD12; sterile alpha motif domain-containing protein 12 isoform X3
1.32E-10 SQLE; squalene monooxygenase
9.42E-09 TSTA3; GDP-L-fucose synthase isoform X2
3.10E-09 LOC121113091; uncharacterized protein LOC121113091
4.18E-07 PRORSD1P; PrdX-deacylase domain 1
1.13E-11 CFAP36; cilia- and flagella-associated protein 36 isoform X1
1.34E-06 KIAA1841; uncharacterized protein KIAA1841 homolog isoform X6
5.91E-23 BUB1; mitotic checkpoint serine/threonine-protein kinase BUB1
2.03E-14 LOC421232; GDNF-inducible zinc finger protein 1
9.93E-07 LOC101747556; uncharacterized protein LOC101747556
3.25E-06 CLIP4; CAP-Gly domain-containing linker protein 4 isoform X3
3.58E-09 EHBP1; EH domain-binding protein 1 isoform X21
2.79E-19 WDPCP; WD repeat-containing and planar cell polarity effector protein fritz homolog isoform X5
2.56E-16 UGP2; UTP--glucose-1-phosphate uridylyltransferase isoform X1

1.75E-08	SLC1A4; neutral amino acid transporter A
4.26E-07	JAG1; protein jagged-1
7.27E-09	RRBP1; ribosome-binding protein 1 isoform X2
0.0003	GINS1; DNA replication complex GINS protein PSF1
1.03E-17	TLR5; toll-like receptor 5 precursor
6.93E-06	PTPN14; tyrosine-protein phosphatase non-receptor type 14
5.65E-06	ABCG8; ATP-binding cassette sub-family G member 8 isoform X2
1.95E-07	TTC7A; tetratricopeptide repeat protein 7A isoform X5
1.13E-07	KCNK17; potassium channel subfamily K member 17 isoform X1
2.12E-08	NFKBIE; NF-kappa-B inhibitor epsilon
1.93E-08	EIF2AK2; interferon-induced, double-stranded RNA-activated protein kinase isoform X2
2.01E-05	QPCT; glutaminyl-peptide cyclotransferase isoform X3
1.32E-06	PLD5; inactive phospholipase D5 isoform X5
1.40E-15	GREM2; gremlin-2
1.44E-10	SLC35F3; putative thiamine transporter SLC35F3 isoform X2
3.28E-06	GNPAT; dihydroxyacetone phosphate acyltransferase isoform X1
3.93E-06	THBS2; thrombospondin-2 isoform X2
4.83E-07	SULT; sulfotransferase
9.22E-13	DACT2; dapper homolog 2
2.32E-15	MPC1L; mitochondrial pyruvate carrier 1
2.58E-05	TBXT; brachyury protein
6.96E-08	LOC421583; solute carrier family 22 member 2
6.37E-21	FNDC1; fibronectin type III domain-containing protein 1
1.61E-07	ARFGEF3; brefeldin A-inhibited guanine nucleotide-exchange protein 3 isoform X9
1.38E-09	PERPB; p53 apoptosis effector related to PMP-22
1.67E-08	MAP3K5; mitogen-activated protein kinase kinase kinase 5
5.69E-10	SGK1; serine/threonine-protein kinase Sgk1
0.000332	LOC112532128; uncharacterized protein LOC112532128
8.20E-22	HEY2; hairy/enhancer-of-split related with YRPW motif protein 2
1.35E-18	GJA1; gap junction alpha-1 protein
1.05E-11	MCM9; DNA helicase MCM9 isoform X1
2.51E-26	DCBLD1; discoidin, CUB and LCCL domain-containing protein 1 isoform X2
2.19E-09	LOC421740; sulfotransferase family 3A, member 1-like
0.00053	METTL24; methyltransferase-like protein 24 isoform X4
1.15E-12	LOC121113177; uncharacterized protein LOC121113177
4.19E-05	FHL5; four and a half LIM domains protein 5 isoform X1
1.15E-08	NT5E; 5'-nucleotidase
4.80E-10	PRSS35; inactive serine protease 35 isoform X2

2.94E-24 ME1; NADP-dependent malic enzyme
5.24E-17 RWDD2A; RWD domain-containing protein 2A
3.53E-08 HMGCLL1; 3-hydroxymethyl-3-methylglutaryl-CoA lyase, cytoplasmic isoform X1
8.99E-07 FAM110C; protein FAM110C
1.10E-13 CMPK2; UMP-CMP kinase 2, mitochondrial
2.22E-43 RSAD2; radical S-adenosyl methionine domain-containing protein 2
3.53E-12 MBOAT2; lysophospholipid acyltransferase 2 isoform X1
4.39E-18 RRM2; ribonucleoside-diphosphate reductase subunit M2
1.26E-07 ATP6V1C2; V-type proton ATPase subunit C 2 isoform X2
2.58E-09 PDIA6; protein disulfide-isomerase A6
3.64E-06 TRIB2; tribbles homolog 2
3.06E-09 VSNL1; visinin-like protein 1 isoform X2
2.41E-08 APOB; apolipoprotein B precursor
1.43E-07 TMEM214; transmembrane protein 214 isoform X3
1.56E-06 DTNB; dystrobrevin beta isoform X11
1.73E-11 KIF3C; kinesin-like protein KIF3C
4.75E-07 HADHA; trifunctional enzyme subunit alpha, mitochondrial
1.46E-09 HADHB; trifunctional enzyme subunit beta, mitochondrial
9.90E-20 LOC121110416; protein aveugle-like isoform X1
7.07E-10 C3H8ORF80; nuclear GTPase SLIP-GC
2.55E-20 PBK; lymphokine-activated killer T-cell-originated protein kinase isoform X1
1.69E-11 LOC121110417; uncharacterized protein LOC121110417 isoform X1
4.71E-12 CLU; clusterin precursor
1.06E-49 SOX7; transcription factor SOX-7
9.66E-07 TDH; L-threonine 3-dehydrogenase, mitochondrial
4.78E-13 BLK; tyrosine-protein kinase Blk isoform X1
2.03E-14 FDFT1; squalene synthase
9.65E-16 AvBD13; gallinacin-13 precursor
1.68E-14 MCM3; DNA replication licensing factor MCM3 isoform X1
7.21E-12 CRISP2; serotriflin isoform X1
1.10E-21 RHAG; ammonium transporter Rh type A
1.46E-12 CYP2AC1; cytochrome P450 2AC1
3.00E-06 SUPT3H; transcription initiation protein SPT3 homolog isoform X2
3.97E-10 RCAN2; calcipressin-2 isoform X2
4.98E-10 ADGRF5; adhesion G protein-coupled receptor F5
3.65E-07 TNFRSF21; tumor necrosis factor receptor superfamily member 21 isoform X1
4.60E-22 ARR3; arrestin-C
4.99E-21 KIF4B; chromosome-associated kinesin KIF4

3.98E-06 LOC422151; fibronectin type-III domain-containing protein 3a
5.40E-15 CCNB3; G2/mitotic-specific cyclin-B3 isoform X2
9.76E-09 GPC3; glypican-3
1.62E-06 LOC121110693; atherin-like
8.47E-10 XKRX; XK-related protein 2 isoform X2
1.15E-05 NOX1; NADPH oxidase 1 isoform X1
1.39E-07 SLC16A2; monocarboxylate transporter 8
1.19E-13 LOC121110638; uncharacterized protein LOC121110638
4.09E-16 SH2D1A; SH2 domain-containing protein 1A isoform X1
2.71E-06 IDS; iduronate 2-sulfatase isoform X4
2.13E-13 LOC100859304; uncharacterized protein LOC100859304
1.43E-12 APELA; apelin receptor early endogenous ligand precursor
5.26E-07 MSMO1; methylsterol monooxygenase 1
4.96E-49 LOC107052719; uncharacterized protein LOC107052719 isoform X1
4.53E-21 DDX60; probable ATP-dependent RNA helicase DDX60
4.36E-06 PALLD; palladin isoform X8
7.44E-09 TBC1D9; TBC1 domain family member 9 isoform X3
0.004491 SMIM18; small integral membrane protein 18
1.73E-05 CXCL13; C-X-C motif chemokine 13 precursor
6.14E-12 CXCL13L3; C-X-C motif chemokine ligand 13-like 3 precursor
1.21E-13 LOC422513; probable E3 ubiquitin-protein ligase HERC3 isoform X1
8.38E-12 ETNPPL; ethanolamine-phosphate phospho-lyase isoform X2
9.59E-06 ARHGEF38; rho guanine nucleotide exchange factor 38
2.69E-06 LOC107051757; CXXC-type zinc finger protein 4 isoform X4
2.71E-23 ACSL1; long-chain-fatty-acid--CoA ligase 1
6.07E-11 AREG; amphiregulin isoform X1
4.70E-20 PRDM8; PR domain zinc finger protein 8
5.93E-07 SPP1; osteopontin precursor
4.58E-10 PLACL2; PLAC8 like 2
5.25E-09 GPAT3; glycerol-3-phosphate acyltransferase 3 isoform X1
5.54E-08 DUSP4; dual specificity protein phosphatase 4
4.43E-07 PPP1R3B; protein phosphatase 1 regulatory subunit 3B isoform X1
6.11E-13 SHROOM3; protein Shroom3 isoform X7
6.27E-05 LOC100858497; uncharacterized protein LOC100858497
7.81E-06 NPFFR2; neuropeptide FF receptor 2
1.09E-16 SULT1B; sulfotransferase family, cytosolic, 1B isoform X1
5.46E-06 CCNA2; cyclin-A2
2.03E-06 FABP2; fatty acid-binding protein, intestinal

8.37E-07 SEC24D; protein transport protein Sec24D isoform X2
0.000168 PITX2; pituitary homeobox 2
8.41E-06 ENPEP; glutamyl aminopeptidase
4.15E-07 CASP6; caspase-6
3.57E-05 TLR3; toll-like receptor 3 isoform X1
1.55E-24 FAT1; protocadherin Fat 1 isoform X8
4.94E-19 PDGFRL; platelet-derived growth factor receptor-like protein isoform X5
8.05E-08 FAM114A1; protein NOXP20 isoform X1
9.09E-82 CCKAR; cholecystokinin receptor type A
6.15E-33 RBPJ; recombining binding protein suppressor of hairless isoform X1
1.60E-15 PPARGC1A; peroxisome proliferator-activated receptor gamma coactivator 1-alpha isoform X1
7.31E-07 PROM1; prominin-1 isoform X15
6.27E-10 FGFBP2; fibroblast growth factor-binding protein 2 precursor
3.45E-07 EVC2; limbin isoform X2
1.07E-06 ZFYVE28; lateral signaling target protein 2 homolog isoform X5
6.48E-09 HAUS3; HAUS augmin-like complex subunit 3
3.84E-09 FGFR3; fibroblast growth factor receptor 3 isoform X7
2.39E-18 SLBP; histone RNA hairpin-binding protein
1.47E-21 SPON2; spondin-2 isoform X2
3.87E-25 SMYD1; histone-lysine N-methyltransferase SMYD1
1.43E-05 SMOX; spermine oxidase isoform X1
1.40E-05 LOC107056280; basic proline-rich protein-like isoform X3
5.54E-07 GIF; cobalamin binding intrinsic factor isoform X1
1.93E-07 LOC107056412; membrane-spanning 4-domains subfamily A member 12-like isoform X3
0.000105 CD6; T-cell differentiation antigen CD6 isoform X1
5.52E-06 TCIRG1; V-type proton ATPase 116 kDa subunit a3
1.73E-11 ALDH3B2; LOW QUALITY PROTEIN: aldehyde dehydrogenase family 3 member B1
0.000871 GPX2; glutathione peroxidase 2
1.36E-12 SPTB; spectrin beta chain, erythrocytic isoform X1
7.57E-47 CDHR5; cadherin-related family member 5 precursor
1.11E-22 BUB1B; mitotic checkpoint serine/threonine-protein kinase BUB1 beta
1.58E-07 KNSTRN; small kinetochore-associated protein
1.92E-27 B4GALNT4; N-acetyl-beta-glucosaminyl-glycoprotein 4-beta-N-acetylgalactosaminyltransferase 1 isoform X1
1.65E-13 LOC770612; interferon-induced transmembrane protein 3
4.16E-10 DHCR7; 7-dehydrocholesterol reductase
1.58E-14 E2F8; transcription factor E2F8 isoform X2
1.45E-08 RCN1; reticulocalbin-1 isoform X1
9.73E-11 PRRG4; transmembrane gamma-carboxyglutamic acid protein 4 isoform X1

1.21E-10	MICAL2; F-actin-monooxygenase MICAL2 isoform X20
4.29E-06	AMPD3; AMP deaminase 3 isoform X2
8.91E-10	PDE3B; cGMP-inhibited 3',5'-cyclic phosphodiesterase B
7.72E-10	ABCC8; LOW QUALITY PROTEIN: ATP-binding cassette sub-family C member 8
3.20E-10	SAA; serum amyloid A isoform X2
2.47E-12	KCNQ1; potassium voltage-gated channel subfamily KQT member 1
#######	TRPM5; transient receptor potential cation channel subfamily M member 5 isoform X1
9.48E-06	CD81; CD81 antigen
1.23E-08	CTSD; cathepsin D precursor
1.01E-12	DUSP8; dual specificity protein phosphatase 8 isoform X2
4.80E-12	PNPLA2; patatin-like phospholipase domain-containing protein 2
3.97E-06	RNH1; ribonuclease inhibitor isoform X2
6.46E-25	CHKA; choline kinase alpha isoform X2
1.01E-06	TESMIN; tesmin isoform X2
1.02E-22	CPT1A; carnitine O-palmitoyltransferase 1, liver isoform isoform X2
3.87E-25	LOC428851; acyl-CoA (8-3)-desaturase-like
4.22E-06	SMTNL1; smoothelin-like protein 1
2.38E-07	PGR2/3; P2X purinoceptor 3 precursor
3.74E-29	SMIM38; small integral membrane protein 38
4.36E-88	FGF19; fibroblast growth factor 19 precursor
1.07E-05	TSPAN18; tetraspanin-18
3.54E-08	MDK; midkine isoform X1
1.06E-11	NUSAP1; nucleolar and spindle-associated protein 1
2.28E-06	PLA2G4B; cytosolic phospholipase A2 beta isoform X3
3.26E-07	RDH11; retinol dehydrogenase 12
1.16E-27	TTC6; tetratricopeptide repeat protein 6 isoform X3
3.55E-08	CLEC14A; C-type lectin domain family 14 member A
1.34E-30	ACSS1B; acetyl-coenzyme A synthetase 2-like, mitochondrial isoform X2
3.87E-07	RPS6KL1; LOW QUALITY PROTEIN: ribosomal protein S6 kinase-like 1 isoform X1
4.42E-08	GALC; galactocerebrosidase isoform X2
5.20E-14	TRIP11; thyroid receptor-interacting protein 11
2.80E-18	LOC107049127; serpin A3-4-like isoform X1
2.53E-11	LOC121110856; uncharacterized protein LOC121110856
8.78E-23	DIO3; thyroxine 5-deiodinase
2.60E-24	ANKRD9; ankyrin repeat domain-containing protein 9
3.68E-34	AMN; protein amnionless isoform X1
5.54E-23	C14orf180; nutritionally-regulated adipose and cardiac enriched protein homolog isoform X4
3.76E-08	DHRS7; dehydrogenase/reductase SDR family member 7

3.85E-26	DLGAP5; disks large-associated protein 5
1.24E-05	CDKL1; cyclin-dependent kinase-like 1
5.74E-24	LOC107051813; prostaglandin D2 receptor-like
3.69E-17	PTGER2; prostaglandin E2 receptor EP2 subtype
5.05E-10	LRFN5; leucine-rich repeat and fibronectin type-III domain-containing protein 5
3.29E-05	LOC768416; protein MANBAL
1.85E-20	GRID1; glutamate receptor ionotropic, delta-1 isoform X3
4.99E-23	SNCG; gamma-synuclein
2.36E-12	ADIRF; adipogenesis regulatory factor
8.51E-09	DNAJC12; dnaJ homolog subfamily C member 12
0.001196	ASAH2; LOW QUALITY PROTEIN: putative neutral ceramidase C isoform X1
8.94E-09	PRKG1; cGMP-dependent protein kinase 1 isoform X3
#######	HKDC1; hexokinase HKDC1 isoform X3
3.57E-12	CYP2C18; cytochrome P450 family 2 subfamily C member 18
1.73E-11	TLL2; tolloid-like protein 2 isoform X6
3.08E-09	TMEM150B; transmembrane protein 150A isoform X1
1.48E-42	SCD; stearoyl-CoA desaturase
1.92E-08	CYP2C23b; cytochrome P450 2H2 precursor
4.64E-09	CYP2C23a; cytochrome P450 2H1 precursor
1.46E-12	MSMB; beta-microseminoprotein
3.84E-15	FRMPD2; FERM and PDZ domain-containing protein 2 isoform X5
5.91E-14	ANKRD22; ankyrin repeat domain-containing protein 22 isoform X1
3.82E-08	ACTA2; actin, aortic smooth muscle isoform X1
1.52E-10	IFIT5; interferon-induced protein with tetratricopeptide repeats 5
4.37E-06	PANK1; pantothenate kinase 1 isoform X3
9.11E-19	CRTAC1; cartilage acidic protein 1
1.15E-06	PI4K2A; LOW QUALITY PROTEIN: phosphatidylinositol 4-kinase type 2-alpha
3.07E-31	HOGA1; 4-hydroxy-2-oxoglutarate aldolase, mitochondrial
9.50E-19	TLX1; T-cell leukemia homeobox protein 1
7.29E-09	PDZD7; PDZ domain-containing protein 7 isoform X4
9.45E-26	CYP17A1; steroid 17-alpha-hydroxylase/17,20 lyase precursor
4.38E-12	STN1; CST complex subunit STN1 isoform X4
1.56E-11	GSTO2; glutathione S-transferase omega-1
8.23E-28	GPAM; glycerol-3-phosphate acyltransferase 1, mitochondrial isoform X1
8.28E-07	ACSL5; long-chain-fatty-acid--CoA ligase 5 isoform X1
3.44E-36	LOC101750892; deleted in malignant brain tumors 1 protein-like isoform X2
9.42E-11	FAM196A; inhibitory synaptic factor 2A isoform X4
6.14E-26	MKI67; proliferation marker protein Ki-67 isoform X4

1.50E-07 ALDH18A1; delta-1-pyrroline-5-carboxylate synthase isoform X7
2.25E-06 COL3A1; collagen alpha-1(III) chain precursor
1.95E-38 ABCA12; ATP-binding cassette sub-family A member 12 isoform X2
6.29E-12 ATIC; bifunctional purine biosynthesis protein ATIC
2.24E-13 LOC121111295; UDP-glucuronosyltransferase 1A1-like
6.08E-13 AHR2; aryl hydrocarbon receptor 2 isoform X4
1.10E-28 AHR1B; aryl hydrocarbon receptor 1 beta isoform X2
2.09E-07 SLC19A1; reduced folate transporter isoform X2
1.74E-05 COL6A1; collagen alpha-1(VI) chain precursor
6.73E-16 COL6A2; collagen alpha-2(VI) chain precursor
6.91E-13 STAT1; signal transducer and activator of transcription 1-alpha/beta
1.52E-09 NABP1; SOSS complex subunit B2
1.89E-14 LOC768589; baculoviral IAP repeat-containing protein 5.1 isoform X1
1.64E-09 COQ10B; coenzyme Q-binding protein COQ10 homolog B, mitochondrial
6.08E-08 ICOS; inducible T-cell costimulator isoform X1
1.61E-18 CTLA4; cytotoxic T-lymphocyte protein 4 precursor
7.50E-06 DNAJC10; dnaJ homolog subfamily C member 10
1.01E-06 ZNF385B; zinc finger protein 385B isoform X9
3.94E-11 PDE11A; dual 3',5'-cyclic-AMP and -GMP phosphodiesterase 11A isoform X3
2.46E-08 CDCA7; cell division cycle-associated protein 7 isoform X2
1.10E-16 RAPGEF4; rap guanine nucleotide exchange factor 4 isoform X5
3.17E-09 SPC25; kinetochore protein Spc25
6.44E-06 IFIH1; interferon-induced helicase C domain-containing protein 1
4.43E-08 DPP4; dipeptidyl peptidase 4
7.92E-16 SLC4A10; sodium-driven chloride bicarbonate exchanger isoform X6
2.32E-36 ITGB6; integrin beta-6 isoform X1
7.63E-05 LOC107049146; gap junction gamma-1 protein-like
1.66E-29 LOC424199; uncharacterized protein LOC424199 isoform X1
6.88E-15 MNR2; motor neuron and pancreas homeobox protein 1
1.65E-13 RPL37A; 60S ribosomal protein L37a
3.04E-26 ARHGEF1; uncharacterized protein ARHGEF1 isoform X3
7.47E-10 EAF2; ELL-associated factor 2
3.61E-06 KIF5C; kinesin heavy chain isoform 5C
1.17E-06 NMI; N-myc-interactor isoform X1
5.86E-07 UPP2; uridine phosphorylase 2 isoform X2
6.40E-05 SELP; P-selectin isoform X5
9.98E-28 LOC107053928; tumor necrosis factor ligand superfamily member 18-like
2.12E-07 FASLG; tumor necrosis factor ligand superfamily member 6 isoform X1

1.28E-21	SUCO; SUN domain-containing ossification factor isoform X5
2.35E-18	RGS8; regulator of G-protein signaling 8 isoform X2
6.96E-08	RGSL1; regulator of G-protein signaling protein-like isoform X1
1.30E-22	ABL2; tyrosine-protein kinase ABL2 isoform X2
4.77E-26	TOR3A; torsin-3A
1.05E-16	ASTN1; astrotactin-1 isoform X2
7.60E-07	KIAA0040; uncharacterized protein KIAA0040 homolog
1.35E-06	SLC30A7; zinc transporter 7
3.06E-06	LRRC39; leucine-rich repeat-containing protein 39 isoform X2
5.54E-07	TRMT13; tRNA:m(4)X modification enzyme TRM13 homolog
1.42E-23	CLCA1; calcium-activated chloride channel regulator 1-like
2.46E-06	LPAR3; lysophosphatidic acid receptor 3 isoform X1
1.78E-11	SSX2IP; afadin- and alpha-actinin-binding protein isoform X3
2.12E-15	VTG2; vitellogenin-2 precursor
2.97E-11	LOC121111362; uncharacterized protein LOC121111362 isoform X1
6.84E-23	VTG3; vitellogenin-3
1.50E-06	SPATA1; spermatogenesis-associated protein 1 isoform X3
4.61E-06	THAP10; sterile alpha motif domain-containing protein 13 isoform X4
8.71E-18	VTG1; vitellogenin-1 precursor
0.00017	ARTN; artemin isoform X1
1.55E-24	RBP; riboflavin-binding protein precursor
6.41E-08	FAAH; fatty-acid amide hydrolase 1
8.45E-11	CYP4B7; cytochrome P450 4B7
9.00E-05	PDZK1IP1; PDZK1-interacting protein 1 precursor
2.05E-10	PODN; podocan isoform X3
3.89E-05	TCEANC2; transcription elongation factor A N-terminal and central domain-containing protein 2
1.82E-09	DHCR24; delta(24)-sterol reductase
6.20E-08	C8B; complement component C8 beta chain isoform X1
2.00E-11	DAB1; disabled homolog 1
2.20E-08	TACSTD2; tumor-associated calcium signal transducer 2 precursor
2.03E-17	HOOK1; protein Hook homolog 1
8.95E-08	KANK4; KN motif and ankyrin repeat domain-containing protein 4 isoform X1
1.35E-28	ANGPTL3; angiopoietin-related protein 3 precursor
9.19E-08	DNAJC6; putative tyrosine-protein phosphatase auxilin
1.18E-11	LRRC7; leucine-rich repeat-containing protein 7 isoform X7
4.67E-10	PTGER3; prostaglandin E2 receptor EP3 subtype
9.84E-07	CRYZ; quinone oxidoreductase isoform X1
0.000103	NEU2; sialidase-2 isoform X3

4.61E-09 KLHL24; kelch-like protein 24 isoform X1
1.97E-12 EHHADH; peroxisomal bifunctional enzyme isoform X2
1.43E-09 DNAJB11; dnaJ homolog subfamily B member 11
3.90E-08 SGPP2; sphingosine-1-phosphate phosphatase 2
1.56E-05 GK5; putative glycerol kinase 5
2.78E-15 PCOLCE2; procollagen C-endopeptidase enhancer 2 isoform X1
2.99E-09 PLOD2; procollagen-lysine,2-oxoglutarate 5-dioxygenase 2 isoform X2
6.26E-47 LOC121113436; uncharacterized protein LOC121113436
1.46E-07 BDH1B; D-beta-hydroxybutyrate dehydrogenase, mitochondrial isoform X1
6.08E-13 RTP2; receptor-transporting protein 2-like
4.57E-16 PDCD1; programmed cell death protein 1
7.53E-07 NPPC; C-type natriuretic peptide
1.06E-09 ALPI; intestinal-type alkaline phosphatase
9.61E-11 CLCN2; chloride channel protein 2 isoform X2
9.16E-13 AHSG; alpha-2-HS-glycoprotein
7.64E-09 B3GNT5; lactosylceramide 1,3-N-acetyl-beta-D-glucosaminyltransferase isoform X1
3.16E-07 LAMP3; lysosome-associated membrane glycoprotein 3 isoform X1
4.21E-10 ECT2; protein ECT2 isoform X3
2.49E-06 GHSR; growth hormone secretagogue receptor type 1
1.54E-11 LOC107054133; SLIT and NTRK-like protein 3
8.32E-14 SMC4; structural maintenance of chromosomes protein 4
2.04E-16 IFT80; intraflagellar transport protein 80 homolog
9.35E-17 MLF1; myeloid leukemia factor 1 isoform X1
5.65E-07 C3orf33; protein C3orf33 homolog
3.16E-09 ARHGEF26; rho guanine nucleotide exchange factor 26
7.49E-24 TUBA3E; tubulin alpha-3 chain
2.15E-51 DUOX1; dual oxidase 2
7.42E-13 KIAA0101; PCNA-associated factor
2.28E-13 PATL2; protein PAT1 homolog 2 isoform X1
2.25E-05 CD276; CD276 antigen isoform X1
2.02E-06 CYP1A2; cytochrome P450 1A5 isoform X1
1.21E-08 CYP1A1; cytochrome P450 1A4
6.52E-05 ISLR; immunoglobulin superfamily containing leucine-rich repeat protein
1.71E-08 STOML1; stomatin-like protein 1 isoform X4
1.82E-07 PMLL; protein PML isoform X1
3.32E-07 LOC107052152; microtubule-associated protein 1S-like
2.74E-05 CHRNA3; neuronal acetylcholine receptor subunit alpha-3 precursor
7.20E-19 TRPM1; transient receptor potential cation channel subfamily M member 1 isoform X5

6.28E-39 LIPC; hepatic triacylglycerol lipase isoform X1
4.48E-09 SEMA6D; semaphorin-6D isoform X8
2.85E-10 GATM; glycine amidinotransferase, mitochondrial
4.88E-09 LOC415472; uncharacterized protein LOC415472
2.64E-19 PEX11A; peroxisomal membrane protein 11A isoform X2
2.34E-09 MFGE8; lactadherin isoform 1 precursor
5.15E-07 HAPLN3; hyaluronan and proteoglycan link protein 3
1.84E-09 PGPEP1L; pyroglutamyl-peptidase 1-like protein isoform X1
3.24E-07 ALDH1A3; aldehyde dehydrogenase family 1 member A3
2.39E-06 PCSK6; proprotein convertase subtilisin/kexin type 6 isoform X1
1.33E-06 CORO2B; coronin-2B isoform X3
9.36E-09 TMED3; transmembrane emp24 domain-containing protein 3 isoform 1 precursor
7.84E-08 PRC1; protein regulator of cytokinesis 1
7.47E-26 CETP; cholesteryl ester transfer protein precursor
0.021764 CCL17; chemokine (C-C motif) ligand 17 precursor
6.13E-09 LCAT; phosphatidylcholine-sterol acyltransferase precursor
1.19E-10 LOC415662; C-factor-like isoform X3
9.27E-41 LOC101747680; C-factor-like
1.32E-28 LOC112533303; C-factor-like
2.60E-18 LOC100857820; C-factor
2.06E-06 LOC101748539; uncharacterized oxidoreductase C663.09c-like
2.45E-06 SETD6; N-lysine methyltransferase SETD6 isoform X1
4.59E-35 MT4; metallothionein
1.47E-34 MT3; metallothionein-3
1.40E-18 SLC6A2; sodium-dependent noradrenaline transporter
5.88E-10 CDCA9; borealin-2
3.40E-14 LOC100858381; uncharacterized protein LOC100858381 isoform X1
1.05E-05 RGS9BP; regulator of G-protein signaling 9-binding protein
4.94E-13 LOC769704; fatty acyl-CoA hydrolase precursor, medium chain isoform X1
4.32E-13 CES1L2; fatty acyl-CoA hydrolase precursor, medium chain
3.90E-08 TERB1; telomere repeats-binding bouquet formation protein 1 isoform X3
4.56E-05 BEAN1; protein BEAN1
1.63E-10 CENPN; centromere protein N
1.03E-08 LOC121111713; uncharacterized protein LOC121111713
3.03E-07 CDH13; cadherin-13 isoform X6
2.64E-08 GINS2; DNA replication complex GINS protein PSF2 isoform X1
2.12E-05 SLC7A5; large neutral amino acids transporter small subunit 1
5.29E-14 CA5A; carbonic anhydrase 5A, mitochondrial isoform X2

1.13E-05 CDT1; DNA replication factor Cdt1 isoform X1
1.03E-20 SPIRE2; protein spire homolog 2 isoform X1
4.20E-09 LOC121111704; uncharacterized protein LOC121111704
1.92E-27 LOC121106611; uncharacterized protein LOC121106611 isoform X2
1.31E-08 NT5DC2; 5'-nucleotidase domain-containing protein 2 isoform X1
5.26E-07 SMIM4; small integral membrane protein 4
5.51E-08 ITIH3; inter-alpha-trypsin inhibitor heavy chain H3 isoform X3
3.14E-06 MUSTN1; musculoskeletal embryonic nuclear protein 1
9.23E-06 MANF; mesencephalic astrocyte-derived neurotrophic factor isoform X2
1.61E-09 GBP4L; guanylate-binding protein 1 isoform X1
1.18E-09 LOC121106469; guanylate-binding protein 2-like isoform X2
1.43E-19 LOC121106623; guanylate-binding protein 1-like
4.14E-10 LOC121106625; guanylate-binding protein 1-like
1.27E-06 LOC121106626; guanylate-binding protein 2-like isoform X2
5.19E-16 MST1R; macrophage-stimulating protein receptor isoform X1
9.95E-08 USP4; ubiquitin carboxyl-terminal hydrolase 4
1.44E-30 OASL; 59 kDa 2'-5'-oligoadenylate synthase-like protein
1.82E-05 OGN; mimecan isoform X1
1.46E-07 DNASE1L3; deoxyribonuclease gamma precursor
9.78E-07 ABHD6; monoacylglycerol lipase ABHD6 isoform X2
2.40E-35 C3orf67; protein CFAP20DC isoform X1
5.43E-08 GPR27; probable G-protein coupled receptor 27
1.80E-20 PROK2; prokineticin-2
0.000881 GHRL; appetite-regulating hormone preproprotein
3.19E-08 NRG2; pro-neuregulin-2, membrane-bound isoform isoform X5
0.000159 MZB1; marginal zone B- and B1-cell-specific protein
2.39E-44 TENM2; teneurin-2 isoform 2
9.91E-09 HMMR; hyaluronan mediated motility receptor isoform X1
4.32E-14 PTTG2; securin isoform 1
8.03E-12 SLC26A2; sulfate transporter isoform X1
1.04E-08 LOC416147; E3 ubiquitin/ISG15 ligase TRIM25-like
7.84E-13 STC2; stanniocalcin-2
5.16E-06 RGS14; regulator of G-protein signaling 14
4.64E-12 MXD3; lateral signaling target protein 2 homolog isoform X3
1.31E-07 CPLX2; complexin-2
6.01E-20 RASGEF1C; ras-GEF domain-containing family member 1C
3.36E-06 TRPC7; short transient receptor potential channel 7 isoform X1
1.47E-14 SLC22A5; solute carrier family 22 member 5

5.69E-06 IRF1; interferon regulatory factor 1 isoform X1
4.52E-06 LEAP2; liver-expressed antimicrobial peptide 2 precursor
6.21E-09 YIPF5; protein YIPF5
7.34E-07 GFRA3; GDNF family receptor alpha-3
3.47E-07 CPPED1; serine/threonine-protein phosphatase CPPED1 isoform X3
2.04E-09 TXNDC11; thioredoxin domain-containing protein 11 isoform X3
1.78E-31 BHLHA15; class A basic helix-loop-helix protein 15
2.29E-06 NPTX2; neuronal pentraxin-2 isoform X1
1.66E-10 CYP3A5; cytochrome P450 family 3 subfamily A member 5
8.32E-14 LOC101747873; urotensin-2 receptor
3.76E-05 LOC427665; chemokine-like receptor 1 isoform X1
2.15E-51 CACNA1H; voltage-dependent T-type calcium channel subunit alpha-1H isoform X2
9.81E-05 SOX8; transcription factor SOX-8
4.29E-16 PLK1; serine/threonine-protein kinase PLK1
5.96E-09 IL21R; interleukin-21 receptor precursor
1.28E-05 SDR42E2; putative short-chain dehydrogenase/reductase family 42E member 2 isoform X2
1.48E-08 CDR2; cerebellar degeneration-related protein 2
1.36E-09 RMI2; recQ-mediated genome instability protein 2
9.71E-07 CIITA; MHC class II transactivator isoform X5
2.14E-79 GRIN2A; glutamate receptor ionotropic, NMDA 2A isoform X5
7.07E-06 CARHSP1; calcium-regulated heat-stable protein 1
1.79E-31 HBAD; hemoglobin subunit alpha-D
4.89E-30 HBA1; hemoglobin subunit alpha-A
8.32E-11 PDIA2; protein disulfide-isomerase A2
0.000231 ARHGDIG; rho GDP-dissociation inhibitor 3
6.65E-76 LOC416655; protein PERCC1 isoform X1
1.23E-07 C16orf96; uncharacterized protein C16orf96 homolog isoform X3
1.63E-07 DECR2; peroxisomal 2,4-dienoyl-CoA reductase [(3E)-enoyl-CoA-producing] isoform X1
1.94E-07 LOC121106812; cyclin-F-like isoform X1
8.35E-23 LOC121106813; uncharacterized protein LOC121106813 isoform X1
8.57E-06 ROGDI; protein rogdi homolog isoform X1
3.12E-10 GPRC5B; G-protein coupled receptor family C group 5 member B isoform X2
4.52E-14 DNAH3; dynein heavy chain 3, axonemal isoform X4
2.15E-16 SDF2L1; stromal cell-derived factor 2-like protein 1
2.97E-15 CDC45; cell division control protein 45 homolog isoform X2
3.24E-06 CLDN5; claudin-5
3.57E-06 ULK1; serine/threonine-protein kinase ULK1 isoform X1
5.09E-06 RIMBP2; RIMS-binding protein 2 isoform X14

2.46E-13 TMEM132B; transmembrane protein 132B isoform X5
9.06E-28 AACS; acetoacetyl-CoA synthetase
1.41E-11 ATP2A2; sarcoplasmic/endoplasmic reticulum calcium ATPase 2 isoform 2
5.55E-12 P2RX7; P2X purinoceptor 7
4.29E-08 WDR66; LOW QUALITY PROTEIN: cilia- and flagella-associated protein 251
2.11E-18 RAD9B; cell cycle checkpoint control protein RAD9B isoform X2
1.27E-10 PPTC7; protein phosphatase PTC7 homolog
3.22E-07 TMEM116; transmembrane protein 116 isoform X3
5.86E-06 SELPLG; P-selectin glycoprotein ligand 1
1.00E-14 UNG; uracil-DNA glycosylase
1.08E-53 ACACB; acetyl-CoA carboxylase 2 isoform X5
1.66E-08 VPS29L; vacuolar protein sorting 29 homolog
4.79E-06 CHCHD10; coiled-coil-helix-coiled-coil-helix domain-containing protein 10, mitochondrial
0.000154 LOC121106851; predicted GPI-anchored protein 58
7.26E-13 MMP11; stromelysin-3
7.53E-18 SLC2A11L1; solute carrier family 2, facilitated glucose transporter member 11-like
5.13E-13 LOC769554; solute carrier family 2, facilitated glucose transporter member 11
5.25E-13 GSTT1L; glutathione S-transferase theta-1 isoform X1
5.43E-11 TBX6; T-box-containing protein TBX6L isoform X1
4.30E-05 SLC5A1; sodium/glucose cotransporter 1
6.69E-19 LOC416959; melanotransferrin isoform X2
5.33E-25 PISD; phosphatidylserine decarboxylase proenzyme, mitochondrial isoform X1
1.25E-06 MLEC; malectin isoform X1
1.22E-09 LOC100858984; dynein light chain 2, cytoplasmic
1.96E-05 HSPB8; heat shock protein beta-8
5.11E-08 PRODH; proline dehydrogenase 1, mitochondrial isoform X1
3.31E-36 SLC35E4; solute carrier family 35 member E4
4.00E-20 TCN2; transcobalamin-2
4.26E-07 HORMAD2; HORMA domain-containing protein 2 isoform X9
1.72E-10 LOC417013; acyl-CoA dehydrogenase family member 11 isoform X2
1.83E-06 LOC107049644; rho GTPase-activating protein 32-like
1.76E-10 LOC112531211; uncharacterized protein LOC112531211 isoform X1
2.08E-21 LOC107049794; uncharacterized protein LOC107049794 isoform X3
1.02E-33 MHCY2; LOW QUALITY PROTEIN: class I histocompatibility antigen, F10 alpha chain
5.52E-31 MHCY14; major histocompatibility complex-Y, class I heavy chain, 14 precursor
3.17E-11 LOC121106942; C-type lectin domain family 2 member I-like isoform X5
8.32E-11 MHCY9; major histocompatibility complex Y, class I heavy chain 9 isoform X3
3.36E-83 LENG9L7; leukocyte receptor cluster member 9-like, MHC-Y region, 7

1.53E-10 MHCY7; major histocompatibility complex-Y, class I heavy chain, 7 precursor
4.79E-08 ZNFY1; zinc finger, MHC-Y region, 1
3.01E-09 ZNFY3; MHCY region zinc finger protein 3 isoform X2
6.90E-30 LOC121106436; class I histocompatibility antigen, F10 alpha chain-like isoform X1
9.63E-20 MHCY12; major histocompatibility complex Y, class I heavy chain 12 isoform X1
2.08E-37 LOC121106918; class I histocompatibility antigen, F10 alpha chain-like isoform X2
1.38E-11 LOC121106502; C-type lectin domain family 2 member I-like isoform X5
3.60E-23 LOC121106943; C-type lectin domain family 2 member E-like isoform X1
7.41E-24 LOC121106928; class I histocompatibility antigen, F10 alpha chain-like isoform X1
1.86E-07 MHCY8; major histocompatibility complex Y, class I heavy chain 8 isoform X2
9.10E-14 MHCY32; major histocompatibility complex Y, class I heavy chain 32
3.87E-25 BG8; MHC B-G antigen isoform 1 precursor
2.32E-15 MOGL; erythroid membrane-associated protein-like
3.61E-56 LOC121106920; uncharacterized protein LOC121106920 isoform X18
4.27E-09 BTN3A3L2; uncharacterized protein BTN3A3L2 isoform X1
5.56E-18 BTN3A3L1; erythroid membrane-associated protein-like isoform X4
3.78E-19 KIFC1; kinesin-like protein KIFC1 isoform X2
2.95E-08 IL4I1; L-amino-acid oxidase precursor
1.29E-06 TRIM7.1; tripartite motif-containing protein 7 isoform X5
1.41E-06 TRIM39.2; tripartite motif containing 39
2.48E-07 TRIM27.1; tripartite motif-containing 27 isoform X1
9.94E-07 DMA; B locus M alpha chain 1 precursor
5.00E-19 LOC121106935; collagen alpha-1(I) chain-like
4.98E-06 FCN2; ficolin-2 isoform X12
4.53E-06 MAN1B1; endoplasmic reticulum mannosyl-oligosaccharide 1,2-alpha-mannosidase isoform X2
9.15E-07 NOXA1; NADPH oxidase activator 1 isoform X3
1.65E-08 RNF208; RING finger protein 208
4.35E-06 STPG3; protein STPG3 isoform X1
1.54E-46 NSMF; NMDA receptor synaptonuclear signaling and neuronal migration factor isoform X2
8.00E-09 FAM166A; protein FAM166A
3.25E-09 TUBB4B; tubulin beta-3 chain
5.35E-06 TNFSF15; tumor necrosis factor ligand superfamily member 15
6.05E-09 SLC25A25; calcium-binding mitochondrial carrier protein SCaMC-2 isoform X4
2.40E-10 FPGS; folylpolyglutamate synthase, mitochondrial isoform X9
8.88E-08 DNM1; dynamin-1 isoform X8
2.12E-05 LOC121107102; uncharacterized protein LOC121107102
7.13E-07 LOC417192; torsin family 1, member B-like precursor
1.37E-18 FAM69B; divergent protein kinase domain 1B isoform X1

6.05E-43 AGPAT2; 1-acyl-sn-glycerol-3-phosphate acyltransferase beta isoform X2
2.93E-08 EGFL7; epidermal growth factor-like protein 7 isoform X2
7.42E-30 HSPA5; endoplasmic reticulum chaperone BiP isoform X1
6.93E-11 MVB12B; multivesicular body subunit 12B isoform X2
6.64E-38 HS3ST3A1; heparan sulfate glucosamine 3-O-sulfotransferase 3A1
8.84E-10 FN3K; fructosamine-3-kinase isoform X2
1.59E-11 CDK3; cyclin-dependent kinase 3 isoform X2
7.76E-10 TEN1; CST complex subunit TEN1 isoform X2
1.82E-10 ACOX1; peroxisomal acyl-coenzyme A oxidase 1 isoform X3
1.69E-13 FASN; fatty acid synthase
4.84E-06 LOC417386; pyrroline-5-carboxylate reductase 2
7.89E-21 NOG; noggin precursor
5.74E-07 KPNA2; importin subunit alpha-1
2.82E-06 SOX9; transcription factor SOX-9
2.90E-14 SDK2; protein sidekick-2 isoform X2
8.57E-11 RNF213; E3 ubiquitin-protein ligase RNF213 isoform X1
1.63E-06 CCDC40; coiled-coil domain-containing protein 40
2.39E-24 CBX2; chromobox protein homolog 2
1.06E-27 RBFOX3; RNA binding protein fox-1 homolog 3 isoform X8
4.00E-19 ENPP7; ectonucleotide pyrophosphatase/phosphodiesterase family member 7
1.03E-17 TIMP2; metalloproteinase inhibitor 2 precursor
2.95E-34 SOCS3; suppressor of cytokine signaling 3
3.50E-20 TK1; thymidine kinase, cytosolic
1.60E-05 LOC422106; uncharacterized protein LOC422106 isoform X1
7.45E-08 KRABZFP; uncharacterized protein LOC769812 precursor
4.58E-10 GRIN2C; glutamate receptor ionotropic, NMDA 2C isoform X3
4.42E-06 OTOP3; proton channel OTOP3
1.71E-08 HN1; jupiter microtubule associated homolog 1 isoform 3
3.01E-18 SLC16A5; monocarboxylate transporter 6
2.35E-20 CCL4; chemokine C-C motif ligand 4 precursor
1.65E-05 LOC107054776; uncharacterized protein LOC107054776 isoform X1
4.89E-06 CASP1; caspase-1 isoform X1
9.74E-05 P2RX1; P2X purinoceptor 1 isoform X1
1.57E-14 MIS12; protein MIS12 homolog
3.34E-07 TMEM120A; ion channel TACAN
3.09E-05 CCLI5; uncharacterized protein LOC417533 precursor
2.09E-20 SERPINF1; pigment epithelium-derived factor isoform X1
3.02E-07 SRR; serine racemase isoform X7

2.31E-06 MED13; mediator of RNA polymerase II transcription subunit 13 isoform X3
1.76E-10 ACACA; acetyl-CoA carboxylase isoform X1
2.23E-08 C17orf78; uncharacterized protein C17orf78
8.05E-08 EVI2A; protein EVI2A precursor
1.02E-06 FBXO39; F-box only protein 39 isoform X2
1.03E-07 LOC107057310; protein NDRG3 isoform X2
1.30E-05 ASIP; agouti-signaling protein precursor
2.89E-08 TOX2; TOX high mobility group box family member 2 isoform X1
7.44E-13 PI3; elafin
1.16E-09 GDAP1L1; ganglioside-induced differentiation-associated protein 1-like 1
1.04E-23 EPB42; protein 4.2
7.21E-10 ZNFX1; LOW QUALITY PROTEIN: NFX1-type zinc finger-containing protein 1 isoform X1
5.99E-06 HELZ2; helicase with zinc finger domain 2
6.23E-06 RAD21L1; double-strand-break repair protein rad21-like protein 1 isoform X7
1.22E-08 TPX2; targeting protein for Xklp2 isoform X4
1.19E-17 LOC771972; uncharacterized protein LOC771972 isoform X1
5.91E-12 LOC100859272; WAP four-disulfide core domain protein 2 isoform X1
1.12E-14 WFDC2; WAP four-disulfide core domain protein 3 isoform X4
9.13E-23 WFDC8; balbiani ring protein 3 isoform X2
6.04E-43 SPINT4; kunitz-type protease inhibitor 4
2.05E-08 LOC771994; protein qua-1
7.08E-26 UBE2U; ubiquitin-conjugating enzyme E2 C
2.52E-15 NPEPL1; probable aminopeptidase NPEPL1
5.01E-28 ESPN; espin isoform X6
6.48E-07 MIB2; E3 ubiquitin-protein ligase MIB2
8.04E-56 LOC419409; Golgi integral membrane protein 4 isoform X1
2.11E-09 LOC771069; C-factor isoform X2
1.14E-07 PLEKHN1; pleckstrin homology domain-containing family N member 1
7.65E-07 RERE; arginine-glutamic acid dipeptide repeats protein isoform X3
3.79E-06 SLC25A33; solute carrier family 25 member 33 isoform X1
4.34E-14 RNF186; E3 ubiquitin-protein ligase RNF186
1.56E-05 CAMK2N1; calcium/calmodulin-dependent protein kinase II inhibitor 1
6.32E-08 CELA2A; chymotrypsin-like elastase family member 2A precursor
6.08E-09 MTHFR; methylenetetrahydrofolate reductase
1.24E-08 DRAXIN; draxin isoform X1
9.24E-06 EPHB2; ephrin type-B receptor 2 precursor
4.19E-07 LOC112530142; uncharacterized protein LOC112530142
1.73E-30 DPYSL2; dihydropyrimidinase-related protein 2 isoform X2

2.48E-14 EGR3; LOW QUALITY PROTEIN: early growth response protein 3
1.51E-08 LOC101749127; uncharacterized protein LOC101749127 isoform X1
2.97E-12 ADAM32L2; disintegrin and metalloproteinase domain-containing protein 32
1.44E-13 IDO2; indoleamine 2,3-dioxygenase 2 isoform X2
1.38E-13 ANK1; ankyrin-1 isoform X13
5.94E-10 MTHFD2; bifunctional methylenetetrahydrofolate dehydrogenase/cyclohydrolase, mitochondrial
5.28E-05 NCAPH; condensin complex subunit 2 isoform X2
2.30E-09 ADD2; beta-adducin
2.23E-06 FAM136A; protein FAM136A
2.34E-26 CASP14; caspase-14
8.53E-16 RETSAT; all-trans-retinol 13,14-reductase
6.62E-40 FABP3; fatty acid-binding protein, heart
8.97E-08 SERINC2; serine incorporator 2 isoform X1
6.45E-07 GBP1; guanylate-binding protein 1
0.000532 TRNP1; TMF-regulated nuclear protein 1 isoform X1
1.24E-07 TRIM63; E3 ubiquitin-protein ligase TRIM63 isoform X2
1.43E-21 STMN1; stathmin isoform X1
2.69E-16 PAQR7; membrane progestin receptor alpha isoform X1
4.81E-72 IFI27L2; interferon alpha-inducible protein 27, mitochondrial
3.10E-06 LCK; proto-oncogene tyrosine-protein kinase LCK isoform X3
1.34E-07 AZIN2; antizyme inhibitor 2 isoform X3
1.08E-16 MYCL; protein L-Myc
1.00E-07 GALE; UDP-glucose 4-epimerase isoform X1
1.77E-12 HMGCL; hydroxymethylglutaryl-CoA lyase, mitochondrial isoform 4
1.45E-20 GRHL3; grainyhead-like protein 3 homolog isoform X4
2.73E-22 IL22RA1; interleukin-22 receptor subunit alpha-1 precursor
1.60E-07 CHEK1; serine/threonine-protein kinase Chk1 isoform X1
2.16E-19 KCNJ5; G protein-activated inward rectifier potassium channel 4 isoform X2
4.10E-06 TMEM45L; transmembrane protein 45B isoform X2
2.71E-13 SCN3B; sodium channel subunit beta-3 isoform X1
8.20E-08 GRAMD1B; protein Aster-B isoform X17
1.65E-10 ZBTB32; zinc finger and BTB domain-containing protein 16
2.40E-08 TAGLN; transgelin
4.68E-06 FXYD2; sodium/potassium-transporting ATPase subunit gamma
0.000163 MPZL3; myelin protein zero-like protein 3
1.93E-07 MPZL2; myelin protein zero-like protein 2 precursor
1.39E-21 LOC101748511; uncharacterized protein LOC101748511 isoform X4
4.19E-14 HYOU1; hypoxia up-regulated protein 1 isoform X1

7.11E-06	VWA5A2; von Willebrand factor A domain-containing protein 5A isoform X2
1.85E-10	LOC121107551; sperm-associated antigen 4 protein-like isoform X2
2.74E-13	LOC101747704; sperm-associated antigen 4 protein-like isoform X3
8.94E-11	LOC107057474; sperm-associated antigen 4 protein-like isoform X3
3.01E-06	PEAR1; platelet endothelial aggregation receptor 1
1.01E-12	SLAMF1; signaling lymphocytic activation molecule
1.92E-06	LOC100857714; T-lymphocyte surface antigen Ly-9 isoform X8
3.85E-09	S100A6; protein S100-A6
4.43E-07	CKS1B; cyclin-dependent kinases regulatory subunit 1
9.84E-08	FDPS; farnesyl pyrophosphate synthase
1.91E-16	LOC121107589; uncharacterized protein LOC121107589 isoform X4
5.51E-05	CRP; C-reactive protein precursor
6.13E-11	TAGLN2; transgelin-2 isoform X1
9.21E-06	TMOD4; tropomodulin-4
1.67E-10	LOC121107545; prefoldin subunit 2-like
2.25E-11	THEM4; acyl-coenzyme A thioesterase THEM4 isoform X2
4.18E-05	LOC121106470; deaminated glutathione amidase-like isoform X7
3.32E-18	PFDN2; prefoldin subunit 2 isoform X1
1.17E-30	TULP1; tubby-related protein 1 isoform X1
7.39E-10	FKBP5; peptidyl-prolyl cis-trans isomerase FKBP5 isoform X1
2.19E-26	ELF3; ETS-related transcription factor Elf-3
5.12E-24	CSRP1; cysteine and glycine-rich protein 1
6.78E-05	UBE2T; ubiquitin-conjugating enzyme E2 T isoform X2
1.41E-08	KCNA3; potassium voltage-gated channel subfamily A member 3 isoform X1
3.60E-14	NUAK2; NUAK family SNF1-like kinase 2
3.28E-14	LOC121107619; Krueppel-like factor 15 isoform X1
7.09E-11	SLC45A3; solute carrier family 45 member 3
#######	SLC26A9; solute carrier family 26 member 9
3.77E-30	C1orf186; regulator of hemoglobinization and erythroid cell expansion protein
1.95E-39	RAB7B; ras-related protein Rab-7b isoform X1
3.25E-12	FAM72A; protein FAM72A isoform X1
2.28E-30	LAMB3; laminin subunit beta-3
2.96E-30	G0S2; G0/G1 switch protein 2
3.19E-06	GUCA1A; guanylyl cyclase-activating protein 1
1.94E-13	WNT2B; protein Wnt-2b precursor
3.91E-08	MOV10; putative helicase MOV-10 isoform X1
1.23E-12	SLC16A1; monocarboxylate transporter 1
1.01E-05	OLFML3; olfactomedin-like protein 3 precursor

1.37E-08 PACSIN1; protein kinase C and casein kinase substrate in neurons protein 1
1.07E-10 TSPO2; translocator protein 2
1.37E-09 LOC768786; acidic mammalian chitinase isoform X1
8.93E-21 LOC107057545; uncharacterized protein LOC107057545 isoform X3
1.90E-08 LOC107049117; maestro heat-like repeat-containing protein family member 7 isoform X1
1.16E-07 VWA5A1; von Willebrand factor A domain-containing protein 5A isoform X8
1.93E-06 MRC2; C-type mannose receptor 2
2.51E-26 SLC4A1; band 3 anion transport protein isoform X3
2.29E-10 COL1A1; collagen alpha-1(I) chain isoform X1
9.08E-06 SLC35B1; solute carrier family 35 member B1
1.32E-06 LOC429785; G protein-activated inward rectifier potassium channel 1
3.85E-12 C27H17ORF105; uncharacterized protein C17orf105 homolog
1.06E-05 TBX21; T-box transcription factor TBX21 isoform X1
4.12E-05 IKZF3; zinc finger protein Aiolos isoform X6
2.63E-06 CSF3; myelomonocytic growth factor precursor
5.15E-08 RARA; retinoic acid receptor alpha
4.63E-30 TOP2A; DNA topoisomerase 2-alpha
4.33E-15 CCR7; C-C chemokine receptor type 7 precursor
1.12E-09 KRT40; keratin, type I cytoskeletal 20
3.35E-10 KRT24; keratin, type I cytoskeletal 19
1.35E-12 KRT10; keratin, type I cytoskeletal 13
2.14E-05 HSPB9; heat shock protein beta-9
2.70E-07 VAT1; synaptic vesicle membrane protein VAT-1 homolog
1.79E-11 RND2; rho-related GTP-binding protein RhoN
9.15E-11 LOC101750889; platelet glycoprotein Ib alpha chain-like
2.98E-11 LOC100857333; platelet glycoprotein Ib alpha chain isoform X1
1.94E-21 ANGPTL4; angiopoietin-related protein 4
4.19E-08 C19orf71; uncharacterized protein C19orf71 homolog
1.05E-06 LOC100857637; transducin-like enhancer protein 1 isoform X3
7.23E-07 NMRK2; nicotinamide riboside kinase 2
5.04E-67 ACSBG2; long-chain-fatty-acid--CoA ligase ACSBG2 isoform X4
6.35E-06 ZAP70; tyrosine-protein kinase ZAP-70
1.91E-09 MEX3D; RNA-binding protein MEX3D
2.41E-05 PLIN5; perilipin-3 isoform X2
9.36E-10 LRG1; leucine-rich alpha-2-glycoprotein isoform X1
5.35E-12 CREB3L3; cyclic AMP-responsive element-binding protein 3-like protein 3 isoform X2
7.22E-07 MIDN; midnolin-A isoform X1
1.04E-05 LOC112530455; translation initiation factor IF-2-like isoform X1

3.27E-30 ADAMTSL5; ADAMTS-like protein 5 isoform X3
3.62E-08 LOC100859819; cocaine- and amphetamine-regulated transcript protein isoform X5
2.43E-12 LOC420107; uncharacterized protein LOC420107 isoform X1
1.74E-10 LOC107055361; liprin-alpha-2
2.30E-06 TMEM221; transmembrane protein 221
2.19E-09 LOC107055358; mucin-7-like isoform X3
1.18E-18 LOC121107774; zinc finger protein 34-like isoform X1
4.16E-09 CALR; calreticulin
1.90E-06 LOC769841; sulfotransferase 2B1 isoform X2
4.84E-12 CD163; CD163 molecule like 1 precursor
1.86E-42 LOC101751912; butyrophilin subfamily 3 member A2-like isoform X8
3.17E-33 LOC101751153; E3 ubiquitin-protein ligase TRIM39-like isoform X7
1.25E-26 LOC107049467; uncharacterized protein LOC107049467 isoform X2
7.09E-13 LOC107049945; platelet glycoprotein VI-like isoform X1
2.29E-11 CHIR-AB1; immunoglobulin-like receptor CHIR-AB1 precursor
1.43E-09 LOC107056128; platelet glycoprotein VI-like
0.000105 LOC112531107; platelet glycoprotein VI-like
5.18E-12 LOC121107843; uncharacterized protein LOC121107843 isoform X1
1.15E-05 LOC112531095; immunoglobulin superfamily member 1-like isoform X1
4.11E-14 LOC107049967; uncharacterized protein LOC107049967 isoform X1
1.00E-06 LOC112531135; platelet glycoprotein VI-like
8.05E-08 LOC431060; immunoglobulin superfamily member 1-like isoform X3
1.18E-06 CHIR-AB-600; platelet glycoprotein VI isoform X3
1.02E-05 LOC107050473; LOW QUALITY PROTEIN: platelet glycoprotein VI-like isoform X1
2.12E-05 LOC112531212; leukocyte immunoglobulin-like receptor subfamily A member 2 isoform X4
0.014294 LOC121107883; T-cell-interacting, activating receptor on myeloid cells protein 1-like
1.25E-06 FKBP11; peptidyl-prolyl cis-trans isomerase FKBP11
1.02E-06 METTL7A; methyltransferase-like protein 7A
1.62E-08 LOC107049666; integrin beta-7 isoform X2
8.55E-08 STAT2; signal transducer and activator of transcription 2 isoform X2
3.05E-09 APOF; apolipoprotein F
2.90E-07 LOC100859726; retinol dehydrogenase 16-like
4.00E-19 TAC3; tachykinin-3 isoform X2
3.50E-07 INHBE; inhibin beta E chain
2.26E-05 KRT7; keratin, type II cytoskeletal cochleal isoform X1
4.00E-18 LOC121108105; uncharacterized protein LOC121108105
6.47E-12 LOC121106503; maestro heat-like repeat-containing protein family member 2A isoform X2
6.62E-06 OTX5; cone-rod homeobox protein isoform X1

2.88E-08 LOC426220; avidin isoform X1
4.36E-05 LOC121108197; histidine triad nucleotide-binding protein 1-like
1.49E-31 LOC121108210; uncharacterized protein LOC121108210
1.14E-36 ALPK2; alpha-protein kinase 2
5.82E-13 ENHO; adropin
9.50E-10 ARID3C; AT-rich interactive domain-containing protein 3C isoform X2
4.10E-14 CCL19; C-C motif chemokine 19 precursor
1.24E-26 LOC100857191; C-C motif chemokine 26
3.23E-06 AVD; avidin precursor
8.73E-21 NPR3; atrial natriuretic peptide receptor 3 isoform X4
2.71E-09 NIM1K; serine/threonine-protein kinase NIM1 isoform X2
6.67E-10 HMGCS1; hydroxymethylglutaryl-CoA synthase, cytoplasmic isoform X2
1.75E-09 S100Z; protein S100-Z
1.06E-05 HMGCR; 3-hydroxy-3-methylglutaryl-Coenzyme A reductase
1.06E-06 ARHGEF28; rho guanine nucleotide exchange factor 28
1.22E-08 SLC1A1; excitatory amino acid transporter 3
3.07E-07 GLDC; glycine dehydrogenase (decarboxylating), mitochondrial precursor
0.000341 HACD4; very-long-chain (3R)-3-hydroxyacyl-CoA dehydratase 4
1.27E-29 FBP2; fructose-1,6-bisphosphatase isozyme 2
7.44E-43 GADD45G; growth arrest and DNA damage-inducible protein GADD45 gamma
2.36E-05 TPPP2; tubulin polymerization-promoting protein family member 2
7.55E-08 PPIP5K2; inositol hexakisphosphate and diphosphoinositol-pentakisphosphate kinase 2 isoform X1
3.10E-20 SHB; SH2 domain-containing adapter protein B isoform X1
1.42E-07 TRIM14; tripartite motif-containing protein 14
2.94E-08 PCGF3; polycomb group RING finger protein 3
5.99E-08 MFSD7; solute carrier family 49 member A3
3.84E-45 LPL; lipoprotein lipase precursor
3.46E-15 PSD3; PH and SEC7 domain-containing protein 3 isoform X4
2.05E-07 RHOBTB3; rho-related BTB domain-containing protein 3 isoform X3
3.37E-09 ALDOB; fructose-bisphosphate aldolase B
1.67E-11 KIAA1958; uncharacterized protein KIAA1958 homolog isoform X1
3.08E-17 SMC2; structural maintenance of chromosomes protein 2
3.04E-16 LPAR1; lysophosphatidic acid receptor 1 isoform X1
2.33E-13 CDKN2A; ARF tumor suppressor
5.58E-22 CDKN2B; cyclin-dependent kinase 4 inhibitor B
3.48E-13 TRIM36; E3 ubiquitin-protein ligase TRIM36 isoform X1
8.53E-09 CCDC112; coiled-coil domain-containing protein 112 isoform X3
5.81E-07 LOC107050717; calumenin isoform X1

2.97E-08 CHIR-IG1-5; immunoglobulin-like receptor CHIR-AB1-like precursor
2.03E-16 LOC107050652; putative killer cell immunoglobulin-like receptor-like protein KIR3DX1
3.70E-08 LOC425238; uncharacterized protein LOC425238 isoform X6
1.43E-09 LOC121108719; uncharacterized protein LOC121108719 isoform X2
1.42E-08 CDIPT; CDP-diacylglycerol--inositol 3-phosphatidyltransferase isoform X1
8.84E-06 LOC121108733; protein spinster homolog 1-like isoform X2
3.04E-06 LOC121108736; zinc finger protein castor homolog 1-like isoform X3
2.59E-17 LOC121108741; vasodilator-stimulated phosphoprotein-like isoform X1
1.90E-05 LOC121108764; atherin-like isoform X2
9.25E-06 LOC107057287; nucleolin-like isoform X2
0.000661 LOC121108786; uncharacterized protein LOC121108786
5.77E-06 LOC121108819; uncharacterized protein LOC121108819
2.15E-05 LOC121108844; histidine triad nucleotide-binding protein 1-like
2.30E-07 LOC121108855; uncharacterized protein LOC121108855 isoform X2
2.70E-06 LOC112530182; soluble scavenger receptor cysteine-rich domain-containing protein SSC5D-like
1.07E-09 LOC107050463; clumping factor A-like
1.26E-06 LOC121108875; uncharacterized protein LOC121108875 isoform X6
5.91E-14 LOC107051395; mucin-2-like isoform X1
6.05E-09 MHCY13; major histocompatibility complex-Y, class I heavy chain, 13 precursor
2.81E-15 LOC112530071; NF-kappa-B inhibitor delta-like isoform X1
3.50E-08 LOC121108947; LOW QUALITY PROTEIN: medium-chain specific acyl-CoA dehydrogenase, mitochondrial isoform X1
