# Supplementary material for: Comparative analysis of liver transcriptome reveals adaptive responses to hypoxia environmental condition in Tibetan chicken
Source: Anim Biosci. 2023 Aug 23;37(1):28–38. doi: 10.5713/ab.23.0126 (PMC10766467; doi:10.5713/ab.23.0126)
Supplement: Supplementary file 5 [file ab-23-0126-Supplementary-Table-5.pdf]

Table S5

Geneid	HT1	HT2	HT3	HT4	HT5	CH1	CH2	CH3	CH4	CH5	logFC	type	pvalue
LOC12111	3.334	4.314	4.976	0.771	0.913	0.474	0.33	0.443	0.754	1.429	-2.05894	down	5.50E-17
LOC77646	21.883	20.546	22.844	2.642	1.25	0.657	0.417	0.341	4.347	11.518	-2.00063	down	3.45E-18
LOC12110	2.142	1.796	2.87	0.731	1.053	0.131	0.155	0.259	0.935	2.714	-1.03379	down	4.55E-05
LOC11253	5.471	4.844	26.895	5.635	2.36	0.824	1.349	1.281	3.896	7.554	-1.60046	down	1.11E-10
LOC12110	30.716	35.254	57.809	35.949	49.649	9.117	25.329	16.515	19.888	24.68	-1.13205	down	2.15E-06
CD69L	18.257	17.274	8	0.32	0.614	9.019	1.125	1.358	0.453	6.286	-1.28525	down	1.29E-08
SHANK3	1.733	1.677	3.229	3.177	3.394	0.681	0.608	0.695	0.792	0.681	-1.9325	down	6.62E-17
LOC12110	1.419	1.132	2.508	2.285	2.131	0.599	0.48	0.384	0.563	0.43	-1.94564	down	3.24E-16
ARF5	0.644	0.692	1.245	0.68	0.898	3.929	0.331	3.769	0.219	0.291	1.036942	up	1.30E-07
ELAPOR2	2.989	3.226	1.334	0.728	1.061	0.599	60.782	41.565	24.454	20.012	3.979873	up	8.68E-69
NAPEPLD	5.088	4.775	7.811	5.642	5.797	15.599	11.018	14.67	10.023	13.001	1.143266	up	4.16E-10
GTSE1	0.172	0.113	0.199	0.437	0.096	1.211	0.434	0.696	0.761	0.425	1.78909	up	3.33E-15
ALG12	12.218	11.172	12.612	15.737	13.32	55.116	51.597	58.308	48.506	27.29	1.888036	up	1.49E-21
CRELD2	16.486	14.861	20.903	18.59	14.191	151.045	71.668	126.407	74.457	49.534	2.476048	up	8.45E-34
LOC41774	0.87	0.981	1.051	0.37	0.185	0.316	0.154	0.257	0.515	0.271	-1.18943	down	5.88E-05
IQUB	0.993	0.449	2.583	0.444	0.465	0.398	0.225	0.365	0.362	0.462	-1.44266	down	1.34E-07
CPED1	3.776	3.847	5.473	5.571	5.853	0.618	1.085	0.966	1.486	1.17	-2.20205	down	9.26E-22
AMIGO2	1.022	1.06	1.593	1.993	2.793	0.249	0.447	0.543	0.612	0.846	-1.64765	down	6.79E-12
SRGAP1	6.908	6.068	5.45	1.341	3.581	0.998	2.843	0.793	0.672	2.167	-1.64288	down	2.91E-13
IFNG	0.452	0.295	0.408	0.099	0.062	0.317	0.712	0.94	0.603	1.116	1.483163	up	7.31E-10
LYZ	0.544	0.757	2.059	1.51	1.258	3.96	4.266	2.177	2.985	2.092	1.336207	up	2.16E-10
LOC12110	1.294	1.397	2.113	0.585	1.728	0.721	0.266	0.455	0.802	0.644	-1.29972	down	3.07E-05
PPFIA2	1.5	1.318	2.114	2.201	1.631	0.344	0.688	0.83	0.693	1.211	-1.21747	down	2.18E-07
MGP	7.193	6.635	11.315	12.549	3.21	15.111	19.753	17.301	17.712	20.102	1.137321	up	1.99E-09
HIST1H2B	2.503	4.504	2.076	2.057	5.357	1.098	1.822	0.244	0.298	2.169	-1.5499	down	7.87E-09
HIST1H2B	0.834	0.887	0.415	0.171	1.393	3.099	1.286	1.221	0.995	0.849	1.008735	up	2.35E-05
LOC12110	0.85	0.86	0.923	1.207	0.238	0.526	1.351	0.422	1.18	4.824	1.024872	up	2.70E-05
HIST1H2B	0.184	0.241	0.366	0	0.378	0.74	0.378	0.144	0.175	1.165	1.150958	up	0.000348
APOLD1	0.285	0.261	0.884	1.732	0.819	0.485	0.395	0.567	0.177	0.322	-1.03073	down	8.90E-05
GBE	0.033	0.079	2.065	1.056	0.533	1.875	1.774	1.188	3.172	0.04	1.094758	up	1.40E-10
LOC41797	0	0.266	23.459	10.244	2.214	23.383	19.713	15.409	20.469	0.057	1.126999	up	2.26E-11
MEI1	1.876	1.816	1.172	1.415	2.148	0.395	0.318	0.274	0.432	0.269	-2.31629	down	3.65E-23
CHADL	1.099	1.125	1.179	1.815	0.233	0.34	0.307	0.447	0.399	0.41	-1.51578	down	3.70E-10
ADSL	312.305	295.773	586.536	605.692	277.771	136.866	156.295	103.478	151.042	229.347	-1.4192	down	6.89E-10
NPTXR	2.066	1.895	0.571	0.682	0.203	0.882	8.026	6.964	6	6.474	2.386503	up	2.15E-29
DNAL4	2.011	1.833	1.659	2.273	2.822	3.424	4.872	6.18	5.36	6.934	1.336414	up	5.87E-12

FAM20CL	433.81	459.966	1.954	25.555	0.424	71.277	1363.441	1138.547	1047.744	1258.248	2.404272	up	1.48E-29
SUN2	18.332	17.316	9.806	6.471	7.075	7.402	57.233	48.987	36.912	44.713	1.726428	up	5.34E-18
CARD10	1.29	1.45	2.944	2.441	0.998	0.337	0.14	0.191	0.303	0.345	-2.78867	down	2.30E-30
MCM5	0.54	0.535	0.614	0.596	0.998	1.146	3.726	1.071	1.677	1.032	1.396657	up	7.03E-12
C12orf73	13.17	10.852	12.596	10.129	10.096	5.396	4.577	3.955	4.431	6.632	-1.18541	down	6.39E-07
HSP90B1	157.01	148.585	216.797	181.815	205.894	2004.084	540.326	1100.092	580.61	367.15	2.3351	up	3.79E-31
ASCL1	5.587	5.175	8.032	2.459	3.31	1.84	1.032	0.784	0.838	1.306	-2.08141	down	3.17E-18
PAH	238.822	233.114	374.235	380.978	642.348	91.316	208.43	199.974	210.161	177.224	-1.07547	down	7.95E-06
IGF1	1.396	2.968	5.042	4.753	9.936	2.037	3.741	2.14	0.951	1.939	-1.15627	down	5.02E-06
TRIM24	7.615	7.082	4.887	3.991	3.393	4.644	17.924	13.582	9.219	10.274	1.044814	up	3.45E-08
CGTL	10.907	8.938	16.05	5.069	31.518	30.002	46.911	37.272	45.393	63.747	1.623383	up	1.25E-17
LOC41811	1.899	1.883	2.855	4.507	4.014	1.294	1.723	1.813	1.319	1.05	-1.07369	down	8.28E-06
LOC1211C	1.649	2.021	4.774	3.7	2.608	0.664	2.152	1.614	0.824	1.231	-1.18511	down	2.12E-06
SLCO1B1	1.066	0.722	0.939	0.309	0.386	3.501	1.959	5.744	1.435	0.862	1.978582	up	2.28E-22
SOX5	1.501	1.272	1.747	1.25	1.303	0.515	0.616	0.51	0.564	0.713	-1.27589	down	5.58E-08
ENDOUL	1.525	1.284	0.447	0.403	1.314	8.812	1.747	6.293	5.801	0.396	2.211379	up	1.84E-26
MPPED1	1.909	1.919	3.455	3.065	2.405	0.914	1.38	1.041	1.087	1.175	-1.18739	down	8.10E-07
SULT4A1	3.1	3.017	2.947	1.635	1.33	2.94	4.247	10.085	5.515	7.182	1.316595	up	5.55E-12
PNPLA3	156.704	150.92	24.41	32.198	22.297	76.621	380.097	489.189	268.999	321.288	1.990698	up	4.11E-22
KIAA0930	11.283	11.171	9.327	8.652	9.075	15.679	26.804	22.303	21.111	23.326	1.141463	up	1.24E-09
LOC42619	47.755	44.565	65.099	54	38.406	9.82	24.476	14.135	21.078	19.493	-1.48896	down	8.34E-11
MANSC1	1.901	1.486	1.474	2.536	0.862	2.75	4.059	1.772	5.132	4.576	1.146458	up	7.57E-09
LOC10175	1.536	1.246	1.217	1.653	1.183	0.542	2.004	3.829	9.85	1.347	1.361621	up	7.07E-13
FOXM1	0.088	0.109	0.094	0.081	0.172	0.469	0.677	0.813	1.2	0.444	2.716321	up	8.54E-31
A2ML3	9.361	12.466	9.122	12.015	43.861	7.255	6.724	7.295	6.923	6.451	-1.32521	down	1.74E-08
CASR	1.936	1.763	1.204	1.279	0.076	0.092	0.406	1.116	0.192	0.067	-1.73766	down	1.11E-13
CNP1	3.375	3.785	4.095	2.708	1.362	0.473	1.074	0.784	1.035	1.199	-1.74609	down	1.96E-12
GNB3	2.711	2.118	0.809	0.681	1.682	0.221	0.193	0.323	0.305	0.289	-2.58316	down	1.47E-24
CDCA3	0.108	0.236	0.287	0.119	0.371	2.927	3.005	3.493	4.062	1.469	3.731928	up	1.95E-48
LRRC23	6.919	5.962	3.119	4.935	3.728	2.392	1.181	2.486	2.042	2.078	-1.27634	down	5.52E-08
EPHA1	0.234	0.347	0.187	0.234	0.209	0.29	0.775	0.31	1.088	0.452	1.263825	up	3.10E-09
GSTK1	25.813	24.89	36.138	24.733	60.593	12.92	16.909	12.953	13.8	12.775	-1.31163	down	2.79E-08
KEL	0.295	0.55	1.339	0.171	0.418	0.156	0.027	0.122	0.066	0.368	-1.90067	down	6.47E-13
STYK1	1.827	1.717	1.142	1.657	0.924	0.385	0.591	0.554	0.955	0.771	-1.15704	down	7.71E-07
LOC10085	0.216	0.094	1.373	0	0.074	0	0.074	0.056	0.206	0	-2.36937	down	9.37E-06
HAO2	41.002	37.026	43.088	67.589	79.114	7.782	21.958	24.447	28.712	31.486	-1.22732	down	1.18E-07
MAB21L3	0.579	0.43	0.396	1.773	0.466	1.488	2.123	1.499	1.625	2.004	1.260792	up	2.70E-10
SLC22A15	1.153	1.048	0.659	2.636	0.798	3.578	5.488	3.426	3.478	5.138	1.744937	up	1.09E-17

ZBTB20	3.827	3.739	8.413	3.953	6.59	1.277	2.475	1.599	2.621	1.911	-1.42371	down	2.20E-09
CLDND1	82.942	76.685	23.146	32.862	17.209	25.946	205.85	308.159	185.969	143.588	1.90082	up	8.23E-21
APOV1	9688.972	12281.13	57.327	2791.273	5.816	8688.577	23354.84	23478.16	23683.54	20935.68	2.012192	up	5.78E-22
SLC19A2	8.997	8.025	8.924	11.297	8.364	3.603	1.638	2.271	1.834	3.189	-1.86288	down	2.87E-16
DPT	0.487	0.599	1.194	0.384	0.64	1.242	2.723	1.839	2.619	1.938	1.647392	up	5.04E-16
NIT2	39.094	36.011	24.995	42.309	41.311	11.696	15.774	13.502	16.83	12.726	-1.38118	down	1.17E-09
NFKBIZ	3.886	3.262	4.429	2.939	2.994	16.881	6.573	5.541	4.969	3.061	1.080104	up	2.79E-09
KIAA1524	0.124	0.106	0.175	0.078	0.029	1.139	1.009	0.164	1.454	0.31	2.980687	up	1.58E-36
LOC41841	17.461	16.654	13.158	18.336	30.201	1.595	6.222	2.872	6.604	6.958	-1.98191	down	2.77E-18
CD200L	0	0	0.023	0.313	0.211	7.451	8.096	4.822	7.289	5.72	5.918302	up	1.57E-95
LOC12111	0	0.009	0.011	0	0.044	1.584	1.767	0.943	1.884	0.964	6.694598	up	3.30E-86
CREG1	48.616	45.349	90.265	84.383	124.389	20.135	41.544	23.363	25.985	39.843	-1.3812	down	2.71E-09
ROBO1	0.809	0.627	0.597	0.355	1.754	0.095	0.199	0.128	0.147	0.175	-2.46903	down	1.42E-24
ADAMTS1	0.289	0.327	0.705	0.609	0.702	0.572	0.979	0.533	2.854	0.87	1.140385	up	1.40E-09
MAP3K7C	6.165	6.29	15.608	12.845	8.111	24.463	25.621	19.211	13.582	22.276	1.100999	up	1.52E-09
EVA1C	7.313	8.262	15.975	6.286	11.069	5.972	2.769	2.497	3.694	6.7	-1.17663	down	2.12E-06
CBR3	25.821	26.229	39.048	34.234	40.938	23.002	20.199	14.162	7.617	14.977	-1.05618	down	1.02E-05
PCP4	19.784	17.63	42.577	28.453	30.131	9.503	14.899	7.919	9.19	4.859	-1.5793	down	3.10E-11
MX1	1.326	1.437	2.837	1.887	0.579	2.009	4.719	4.633	2.774	8	1.455836	up	4.75E-14
SIK1	4.647	4.614	6.964	4.199	7.148	8.665	22.969	21.338	22.107	5.429	1.545756	up	3.98E-16
MAOB	8.732	8.255	8.987	4.828	8.704	9.194	25.333	13.051	26.896	22.874	1.300971	up	4.83E-12
MID1IP1	25.443	24.255	10.061	8.648	11.947	9.193	202.099	95.847	27.665	134.486	2.545964	up	4.13E-33
OTC	0.045	0.074	0	0	0	0	0	0	1.125	0.718	3.897553	up	2.18E-25
DMD	0.726	0.853	2.591	1.665	3.339	0.782	1.292	0.708	0.584	1.065	-1.04908	down	2.42E-05
NR0B1	37.5	36.706	148.366	39.949	35.715	5.013	10.594	23.864	3.368	32.718	-1.98075	down	5.07E-17
SLC51AL	1.037	0.838	1.068	1.955	3.483	0.015	0.088	0.048	0.221	0.011	-4.43385	down	6.50E-58
MAP3K15	1.168	1.278	2.428	1.267	1.369	4.167	6.081	7.278	1.918	3.858	1.632918	up	2.53E-17
PDHA2	12.365	12.837	21.312	13.524	16.276	46.25	52.223	68.234	20.674	36.102	1.550083	up	4.61E-16
ADGRG2	0.147	0.183	0.172	0.12	0.105	1.372	0.779	2.511	0.717	0.495	3.005655	up	3.97E-41
LOC1211C	1.293	1.071	29.826	154.672	238.651	36.222	62.315	18.46	12.149	21.391	-1.49905	down	2.94E-11
LOC10705	27.588	27.701	135.478	188.365	451.459	19.677	41.902	13.024	8.698	15.521	-3.07117	down	2.15E-40
RGN	167.399	184.424	233.925	226.051	288.396	51.816	119.103	112.341	122.769	113.115	-1.08355	down	5.20E-06
MRPL30	3.17	3.814	3.279	5.907	4.549	26.705	9.775	3.718	7.903	3.921	1.327958	up	3.40E-12
LYGL	0.491	0.321	1.074	0.941	0.252	7.112	1.387	1.436	1.521	1.886	2.113641	up	9.40E-22
LYG2	5.389	7.857	3.234	13.497	12.139	131.274	41.875	5.341	29.755	5.134	2.34084	up	6.48E-30
IL1R2	1.412	1.015	4.233	3.323	1.28	7.243	6.626	3.662	4.063	3.634	1.16308	up	2.31E-10
IL1RL1	1.863	2.123	4.115	1.225	2.715	5.892	12.473	8.468	13.489	9.759	2.055853	up	2.15E-25
14-Sep	4.587	5.409	10.516	5.927	6.657	10.261	13.933	8.986	28.453	13.509	1.182841	up	5.98E-11

LOC77101	171.429	145.53	349.521	449.305	214.164	164.104	81.372	94.939	118.443	96.565	-1.2597	down	8.93E-08
LOC1211C	1.523	2.514	0.799	7.567	0.711	7.865	6.475	7.834	5.282	13.023	1.625694	up	1.06E-14
HS6ST3	1.605	1.497	1.513	1.551	0.979	0.481	0.213	0.284	0.837	0.323	-1.73831	down	2.07E-13
DCT	2.22	2.581	0.314	0.343	0.081	0.398	0.023	0.264	1.247	0.326	-1.29269	down	5.56E-08
ACOD1	1.389	1.414	1.721	1.603	0.649	26.643	4.629	6.078	4.844	6.586	2.846868	up	2.19E-41
PCDH20	1.115	1.205	0.624	0.357	0.641	0.117	0.515	0.457	0.478	0.273	-1.09714	down	8.09E-06
CPB2	157.071	155.027	189.176	180.56	178.72	86.914	70.611	72.575	83.056	76.294	-1.14382	down	1.32E-06
CKAP2	0.174	0.142	0.531	0.19	0.312	1.292	1.668	1.47	2.363	1.022	2.529941	up	7.47E-31
N4BP2L1	95.971	85.03	142.452	114.608	74.546	39.175	50.532	35.293	49.002	59.138	-1.13664	down	1.54E-06
FLT1	1.535	2.033	1.541	2.408	2.083	0.991	0.994	0.755	0.612	1.085	-1.11258	down	2.34E-06
GJB6	0.039	0.042	0.051	0.028	0.04	0.135	0.559	0.107	2.402	0.333	4.110461	up	1.01E-62
GJB2	0.427	0.523	0.025	0.386	1.49	1.645	6.107	2.822	31.53	4.042	4.014294	up	3.28E-69
ZC3H12C	1.766	1.946	1.532	1.719	1.351	0.455	0.807	0.638	0.523	0.782	-1.37384	down	2.04E-09
SLC35F2	0.489	0.43	1.096	1.131	0.518	0.407	0.393	0.155	0.204	0.304	-1.32153	down	5.45E-07
ENDOD1	0.612	0.607	0.903	0.924	1.226	0.214	0.395	0.431	0.292	0.366	-1.32852	down	1.10E-07
TAF1D	25.24	23.33	26.332	31.761	22.81	12.867	11.808	10.894	11.525	14.28	-1.07689	down	4.31E-06
FZD4	34.172	35.556	27.387	27.321	29.684	7.655	17.11	11.022	15.718	24.384	-1.02204	down	1.01E-05
AQP11	9.811	9.128	6.632	12.259	13.171	3.044	2.877	3.856	5.206	5.277	-1.33168	down	8.91E-09
CAPN5	2.419	2.309	4.736	2.929	5.462	0.518	0.265	0.464	1.345	0.498	-2.52872	down	1.25E-26
TSKU	1.372	1.845	1.784	5.612	10.349	1.378	1.191	0.642	1.163	0.152	-2.21022	down	6.71E-21
WNT11	0.476	0.429	2.908	1.358	1.013	0.055	0.138	0.245	0.262	0.137	-2.87781	down	5.55E-31
ART7B	0.219	0.315	1.671	1.042	2.874	1.272	4.268	3.241	2.793	2.372	1.187351	up	1.50E-09
LOC1211C	0.205	0.323	1.583	0.317	0.099	1.745	2.6	0.376	3.619	3.628	2.241435	up	3.34E-28
PDE2A	1.409	1.431	4.124	1.677	1.598	0.432	1.066	1.427	0.672	0.848	-1.2029	down	1.59E-06
ADAM15	2.879	2.467	2.28	10.016	2.523	0.484	0.209	0.244	0.657	0.113	-3.55846	down	8.66E-48
LOC10705	0.246	0.161	0.033	0.303	0	5.428	0.042	0.16	0.117	0.259	3.006495	up	2.77E-27
HBE	0	0.145	18.385	0	0.152	0	0.761	0	0	0	-4.60855	down	5.23E-42
HBBA	251.682	258.768	745.477	87.668	273.636	26.95	66.282	49.831	28.516	97.289	-2.58853	down	1.57E-28
HBE1	46.694	46.334	122.225	11.314	34.039	1.889	5.324	3.323	3.79	6.931	-3.61555	down	5.72E-50
TAF10	33.266	32.073	29.405	36.015	40.872	22.27	12.977	14.906	9.614	9.548	-1.30801	down	2.10E-08
TPP1	197.54	210.865	179.416	267.845	237.865	73.609	66.489	69.228	42.29	51.302	-1.85198	down	1.80E-16
LOC10705	2.779	3.087	3.012	2.462	3.83	1.402	1.561	1.217	1.965	1.41	-1.00524	down	4.67E-05
AOC1	48.381	46.087	56.519	27.358	115.457	11.105	11.135	9.822	24.553	28.25	-1.79154	down	9.83E-15
GIMAP1-(	7.613	7.95	5.13	5.913	2.9	3.431	4.041	2.285	1.915	2.724	-1.03508	down	8.04E-06
ELP6	3.722	3.527	2.005	2.384	1.855	11.979	4.95	6.835	4.568	5.594	1.329858	up	1.15E-11
SCAP	12.353	12.424	5.509	6.9	7	27.63	16.38	19.536	13.544	21.678	1.160364	up	1.20E-09
ACKR2	1.96	1.81	4.688	2.679	3.108	98.777	14.062	7.437	22.575	6.171	3.38654	up	1.21E-56
CYP8B1	3.027	2.459	7.577	4.087	5.467	188.399	26.446	13.397	42.341	11.072	3.638156	up	2.62E-63

CHDSD	1.816	1.503	2.053	1.127	7.993	1.023	0.084	0.567	0.447	0.71	-2.35382	down	2.33E-22
CATH3	0.349	0.513	1.804	1.719	0.09	0	0.269	0.408	0.083	0	-2.54997	down	7.02E-11
CATH2	0.158	0.155	1.513	4.098	0	0	0.081	0.062	0.075	0	-4.73267	down	1.20E-25
CATH1	0.102	0.201	1.342	3.694	0.21	0.032	0.315	0.478	0	0.185	-2.45205	down	1.12E-15
GIMAP6	26.516	28.234	6.858	3.539	4.856	3.782	6.253	4.603	4.821	4.931	-1.52093	down	6.86E-12
DLEC1	3.841	3.28	5.44	4.057	2.006	2.825	0.629	1.298	1.629	1.084	-1.31837	down	4.25E-08
ACAA1	153.55	145.844	192.261	192.372	200.895	94.721	33.668	54.178	84.062	54.827	-1.46091	down	4.00E-10
SLC22A13	0.622	0.421	0.684	0.309	0.419	0.99	0.871	0.569	2.702	1.262	1.379188	up	1.88E-12
CRYGN	5.002	4.194	4.282	4.503	1.648	1.765	1.974	2.365	2.336	1.351	-1.00309	down	2.71E-05
MAP3K8	0.832	0.711	0.888	1.681	0.814	5.543	1.77	3.528	1.81	1.717	1.543409	up	4.50E-15
PTER	17.892	16.56	37.268	16.7	23.369	9.642	11.691	11.435	11.118	8.84	-1.08412	down	1.42E-05
ACBD7	1.497	1.217	3.527	1.728	6.917	0.739	0.395	0.331	0.752	0.128	-2.66371	down	4.24E-26
OLAH	0.248	0.282	1.006	0.256	1.495	0.223	0.074	0.075	0.198	0.022	-2.46316	down	1.67E-20
ABCB1LA	27.561	25.71	20.052	17.421	19.97	3.137	1.637	2.314	3.203	1.458	-3.23568	down	4.66E-44
CYP51A1	48.663	52.847	8.028	7.111	62.297	61.16	139.871	167.67	59.036	153.572	1.699753	up	1.50E-17
CDK6	8.803	8.955	11.844	8.675	7.442	5.26	30.853	33.17	17.376	19.665	1.217512	up	7.41E-11
LOC42056	34.03	28.089	50.517	42.988	55.16	24.067	23.408	11.11	20.22	23.256	-1.0463	down	1.46E-05
COL1A2	1.117	1.323	1.497	2.017	2.457	2.09	5.87	2.843	3.737	3.387	1.09133	up	7.16E-09
ICA1	0.639	0.604	0.692	0.156	0.307	0.631	0.377	0.848	3.706	2.666	1.776582	up	4.57E-18
VWDE	3.3	3.238	1.375	2.446	0.372	0.939	0.039	0.089	1.561	0.006	-2.02439	down	1.85E-18
HOXA2	1.397	1.516	1.744	0.809	1.141	0.275	0.848	0.372	0.545	0.517	-1.36782	down	2.61E-07
FKBP14	2.634	2.28	3.848	1.517	2.599	5.483	4.306	8.303	4.134	4.033	1.027618	up	1.12E-08
SATB1	0.905	0.762	1.234	1.188	0.641	1.217	2.747	3.498	2.787	3.254	1.512378	up	6.68E-15
LOC10175	0.982	0.675	1.017	0.363	0.454	0.456	0.202	0.268	0.234	0.089	-1.47917	down	1.19E-05
MTURN	13.71	14.464	20.274	17.192	21.214	9.02	10.827	5.695	8.673	7.938	-1.04287	down	1.46E-05
ACAD11	94.158	91.985	133.79	127.816	125.149	35.902	34.805	40.073	57.796	45.553	-1.41978	down	1.10E-09
TGM4	0.156	0.204	0.348	0.885	0.417	6.52	0.176	0.195	0.208	0.24	1.865786	up	5.82E-19
KIF15	0.065	0.038	0.103	0.331	0.214	1.184	0.613	0.937	1.488	0.592	2.672276	up	4.40E-33
TRANK1	16.125	15.35	13.587	10.786	8.698	16.435	28.213	24.42	26.064	36.476	1.027791	up	3.00E-08
ANLN	0.042	0.049	0.153	0.144	0.232	1.391	0.481	0.548	1.179	0.612	2.753947	up	3.08E-34
AMPH	0.024	0.056	1.094	0.01	2.771	0.692	0.087	0.028	0.058	0.297	-1.7627	down	2.45E-10
succinyl-C	1.084	0.979	0.981	1.371	1.874	0.507	0.433	0.633	0.885	0.557	-1.05943	down	1.93E-05
PDIA4	95.545	97.275	183.461	161.217	159.512	769.753	256.27	436.655	383.625	177.296	1.537665	up	2.99E-16
LOC12110	0.224	0.212	3.531	0.102	2.508	0	3.226	2.469	2.899	4.709	1.015696	up	4.74E-09
KCNG2	0.662	0.506	0.242	0.203	0.111	0.179	1.293	1.083	0.577	1.278	1.352476	up	1.91E-11
DCDC2	1.066	0.95	2.24	1.084	1.124	12.21	3.274	2.562	3.453	2.272	1.877891	up	3.42E-22
ID4	2.554	3.021	38.134	8.279	11.246	0.758	3.236	4.15	1.701	6.137	-1.98392	down	8.10E-16
ELOVL2	182.236	179.616	5.847	23.01	45.086	23.378	365.276	417.236	324.005	295.678	1.709809	up	5.14E-17

SYCP2L	87.486	81.32	3.059	10.072	17.064	12.339	174.37	196.927	131.973	145.905	1.732971	up	2.39E-17
GCNT2	1.552	1.459	0.351	0.81	1.005	0.48	12.291	2.804	5.646	2.036	2.166394	up	6.62E-25
F13A1	0.857	0.865	0.604	1.348	0.503	9.979	0.327	0.678	0.432	0.41	1.500307	up	4.12E-14
PPP1R3G	6.647	6.35	8.518	6.672	18.704	2.722	2.383	1.922	4.78	2.497	-1.71254	down	2.73E-13
ECI2	248.06	223.024	457.551	325.633	209.669	233.377	33.997	76.852	129.334	112.729	-1.32016	down	3.96E-08
SERPINB1	0.182	0.238	0.512	0.287	0.359	1.022	0.819	0.928	0.894	0.468	1.385575	up	1.17E-11
LOC4209(	1.399	1.359	0.971	1.558	1.669	1.761	4.036	3.91	2.239	2.3	1.033697	up	7.83E-08
ANKRD33	0.689	0.86	0.748	1.736	0.884	0.087	0.595	0.391	0.09	0.198	-1.84929	down	5.67E-14
CMBL	109.934	119.002	147.194	84.218	175.479	38.464	8.848	10.755	20.711	10.081	-2.83897	down	2.97E-34
SRD5A1	3.159	2.519	9.173	6.425	4.217	1.172	1.815	1.419	2.232	1.799	-1.59473	down	2.18E-11
SEC61B	63.379	70.841	78.921	65.259	55.47	160.142	150.234	137.25	106.181	140.128	1.055503	up	9.52E-09
IFI6	18.124	21.104	45.414	28.49	5.721	17.962	102.612	171.736	61.922	240.44	2.322867	up	3.47E-30
GLIPR2	0.355	0.398	0.878	0.698	0.456	1.765	1.042	0.811	0.846	1.261	1.03827	up	4.79E-08
CIDEA	199.64	214.587	173.713	209.271	309.342	119.029	91.071	70.399	158.342	113.466	-1.00252	down	2.82E-05
TGIF1	12.25	10.563	11.097	3.061	3.781	3.217	6.273	3.364	1.944	3.185	-1.18001	down	4.18E-07
LPIN2	29.822	30.149	58.891	30.487	110.317	19.215	18.388	18.469	35.353	32.42	-1.06809	down	1.97E-05
NDC80	0.325	0.206	0.606	0.396	0.4	0.913	0.81	0.695	1.291	0.99	1.279319	up	4.31E-11
ABHD3	12.343	10.311	30.543	4.203	14.329	1.399	1.456	1.263	2.037	1.606	-3.20741	down	2.92E-41
LAMA3	0.63	0.857	1.484	1.121	2.179	0.331	0.546	0.554	0.246	0.428	-1.5726	down	2.81E-11
DSC1	0.063	0.069	0.006	0.006	0	0	1.922	1.514	0.327	0.76	4.92549	up	2.92E-70
MCM4	0.945	0.866	1.037	0.742	1.931	3.258	5.042	2.312	2.755	1.786	1.455772	up	6.34E-14
C8orf22	2183.872	2049.309	595.971	1564.483	746.212	402.536	481.193	429.337	1133.48	1040.556	-1.03386	down	3.89E-06
FAM110B	2.094	2.421	2.344	2.525	3.412	0.731	1.688	0.922	1.496	0.96	-1.14163	down	1.63E-06
CYP7A1	5.926	9.393	3.053	30.473	24.168	55.731	88.508	70.866	99.027	32.766	2.248208	up	1.10E-27
MTFR1	103.881	100.861	62.933	67.583	61.468	31.079	29.254	36.997	39.523	47.493	-1.10571	down	1.58E-06
XKR9	0.272	0.235	0.097	0.395	0.345	0.768	0.435	0.398	2.415	0.414	1.717044	up	6.28E-17
MSC	0.485	0.571	1.7	0.459	0.424	0.285	0.424	0.095	0.278	0.219	-1.48037	down	3.05E-07
TRPA1	1.316	1.061	0.084	2.772	4.407	0.306	0.133	0.147	0.336	0.223	-3.06815	down	1.16E-35
GDAP1	0.009	0.029	0.084	0.78	0.108	2.345	0.009	0.439	0.57	0.032	1.744055	up	5.12E-16
FABP4	0.671	0.603	1.803	3.858	0.861	0.104	0	0.131	0.08	0.379	-3.48029	down	4.05E-22
CA13	2.575	2.243	4.942	1.926	2.63	0.394	0.58	0.993	0.317	1.138	-2.06311	down	1.07E-17
ATP6V0D2	0.331	0.185	0.978	0.233	0	9.873	1.894	0.406	0.428	0.662	2.93744	up	3.02E-39
TMEM55A	6.209	6.093	7.552	4.539	6.765	3.069	3.701	3.065	2.503	2.854	-1.03605	down	2.22E-05
PTDSS1	16.268	15.45	18.831	8.89	8.963	48.124	31.497	31.759	28.922	15.401	1.186627	up	9.66E-11
NCALD	15.006	14.897	22.578	15.2	31.005	2.025	8.024	7.115	13.728	4.221	-1.49071	down	4.39E-10
RIMS2	0.112	0.099	0.68	0.433	0.142	0.523	1.224	0.956	0.79	1.365	1.725049	up	1.84E-18
LOC1211(	2.144	2.4	1.82	1.739	2.811	0.402	1.58	0.826	1.09	1.454	-1.02734	down	1.88E-05
SAMD12	1.124	1.049	1.62	2.375	2.304	6.49	1.811	2.679	6.302	4.529	1.363761	up	4.27E-13

ENPP2	7.94	8.56	13.287	15.452	12.036	4.742	4.507	5.348	5.614	6.494	-1.10065	down	3.82E-06
SQLE	22.329	24.556	0.467	0.583	24.186	41.824	61.838	93.362	25.589	62.839	1.984682	up	1.95E-22
MYC	10.64	9.605	10.029	2.827	2.24	3.305	6.24	2.611	1.918	2.414	-1.09969	down	3.21E-06
LOC42838	0.05	0.033	0	0.041	0	2.158	0.102	0.272	0.047	0.225	4.444614	up	2.73E-24
RHPN1	0.708	0.818	1.538	0.937	1.039	0.273	0.605	0.309	0.202	0.532	-1.38925	down	1.58E-08
LOC12111	3.227	3.889	5.916	2.652	5.582	3.364	6.021	0	0.567	0.346	-1.04582	down	2.83E-05
NRBP2	4.557	4.985	7.246	8.206	7.364	1.986	2.043	2.628	1.772	1.811	-1.65943	down	9.37E-13
FANCL	3.15	2.907	15.648	18.086	12.899	3.513	6.774	5.6	5.113	4.792	-1.03046	down	3.08E-05
KIAA1841	0.506	0.395	1.208	0.72	0.837	1.673	1.854	2.338	1.778	1.782	1.361085	up	4.60E-13
BUB1	4.253	4.887	8.572	9.029	2.457	5.392	18.385	18.614	3.786	18.622	1.149966	up	1.16E-09
CAPN13	0.264	0.107	0.381	1.645	0.336	0.117	0.207	0.354	0.168	0.091	-1.53932	down	2.16E-09
UGP2	111.949	111.93	146.823	110.221	109.185	216.539	189.765	450.577	133.539	222.789	1.039771	up	8.32E-09
BIRC5	1.724	1.45	1.863	1.889	2.235	4.473	4.472	2.595	4.385	3.675	1.096857	up	1.95E-08
RRBP1	20.908	22.272	26.808	27.12	8.192	45.931	70.737	68.628	34.942	47.445	1.345979	up	2.04E-12
GINS1	0.504	0.431	0.617	0.7	1.273	2.086	3.821	1.36	1.736	0.946	1.495608	up	5.23E-12
CAPN2	0.891	1.053	1.373	1.57	0.767	0.294	0.723	0.863	0.274	0.312	-1.19545	down	1.65E-06
TLR5	1.701	1.462	1.014	2.244	0.513	0.701	0.445	0.365	0.323	0.169	-1.78897	down	1.56E-15
MAD2L1B	3.289	2.865	4.357	2.523	8.968	1.802	2.961	1.874	1.804	1.579	-1.13436	down	3.96E-06
RMDN2	24.209	22.274	32.058	23.218	23.641	10.441	10.726	9.817	11.518	12.188	-1.19711	down	4.34E-07
PLD5	0.29	0.361	0.031	0.571	1.529	0.797	0.07	1.264	2.442	1.674	1.165605	up	3.49E-10
GREM2	0.261	0.43	1.984	0.788	0.728	0	0.696	0.033	0.07	0.123	-2.17837	down	4.79E-18
SLC35F3	0.254	0.206	0.501	0.338	1.305	0.037	0.646	0.094	0.208	0.088	-1.27514	down	1.64E-06
GNPAT	11.337	11.076	5.99	4.546	8.803	7.921	25.925	26.221	15.362	22.003	1.222452	up	1.85E-10
SULT	1527.22	1357.682	2707.046	1942.924	485.372	63.563	446.925	600.487	773.079	1263.754	-1.3493	down	6.74E-09
DACT2	0.361	0.382	0.406	0.794	2.125	0.138	0.164	0.182	0.364	0.202	-1.94885	down	1.89E-14
AGPAT4	9.698	8.375	14.161	17.897	14.144	5.899	4.277	4.258	8.947	8.234	-1.02353	down	2.27E-05
LOC42158	1.671	1.488	1.635	0.86	0.951	0.474	0.166	0.44	0.326	0.036	-2.19159	down	4.34E-17
FNDC1	2.801	2.213	0.846	1.502	0.292	0.207	0.572	0.464	0.356	0.543	-1.83483	down	3.72E-16
PERPB	0.023	0.046	0.704	0.134	0.67	0.576	0.096	1.163	0.955	0.926	1.233941	up	1.19E-09
SGK1	10.604	9.899	24.114	13.805	25.396	50.506	32.213	34.661	26.286	26.002	1.017339	up	6.29E-09
LOC10705	32.969	32.892	24.991	42.267	11.618	165.983	113.197	65.271	42.934	39.269	1.5596	up	2.71E-15
CENPW	0.078	0.051	0.112	0.103	0.193	1.195	0.661	0.894	1.212	0.554	3.060277	up	1.19E-34
HEY2	0.109	0.091	0.21	0.076	0.096	0.106	1.196	0.847	0.666	1.796	2.975211	up	6.38E-33
GJA1	19.079	19.274	11.943	15.972	6.838	121.539	42.91	62.722	26.442	23.413	1.921887	up	9.99E-22
MCM9	1.662	1.497	0.398	0.382	0.229	0.21	0.349	0.244	0.477	0.501	-1.22435	down	2.01E-07
DCBLD1	3.256	3.62	2.803	2.341	1.677	2.729	10.236	18.072	12.552	11.632	2.010961	up	1.85E-23
RFX6	0	0	0.078	0.032	0	2.385	0.07	0.175	0.009	0.026	4.537134	up	1.11E-55
LOC42174	143.135	116.205	363.465	245.924	407.447	80.768	104.04	81.798	96.331	108.662	-1.43619	down	1.42E-09

LOC12111	0.079	0.082	0.125	0.052	0.146	0.429	0.049	0.688	1.067	0.128	2.274544 up	6.82E-20
SLC22A16	3.443	3.339	2.051	5.26	4.68	1.396	2.717	1.69	1.609	1.396	-1.09134 down	1.84E-06
LOC42179	2.157	1.85	1.565	2.206	3.151	2.76	6.454	5.911	6.126	2.341	1.109782 up	1.71E-08
MMS22L	0.133	0.196	0.238	0.24	0.642	1.683	0.444	0.311	0.514	0.252	1.142097 up	1.63E-09
SMIM8	7.547	7.22	5.697	4.983	6.362	2.619	3.99	2.996	3.744	2.012	-1.04992 down	1.81E-05
ME1	8.539	9.374	9.18	7.501	16.873	12.995	29.4	78.108	16.091	36.456	1.749371 up	3.08E-19
RWDD2A	3.585	3.795	3.749	2.782	5.311	4.973	7.219	17.647	3.742	9.282	1.156767 up	4.23E-10
IRAK1BP1	1.349	1.4	0.815	0.382	2.055	0.778	0.239	0.436	0.355	1.178	-1.00578 down	0.000625
FAM110C	2.976	2.726	2.712	4.42	5.076	1.984	1.124	0.6	0.99	1.89	-1.44216 down	3.63E-10
CMPK2	8.775	8.064	19.72	12.554	3.339	12.993	17.707	24.971	11.594	41.41	1.050879 up	6.76E-09
MBOAT2	54.307	53.068	2.92	4.222	2.508	0.805	96.437	58.446	74.193	71.54	1.364925 up	1.75E-11
RRM2	0.136	0.204	0.619	0.895	0.879	2.576	7.632	3.034	5.803	0.703	2.850877 up	4.38E-38
ATP6V1C2	0.951	0.739	1.831	1.71	2.036	6.258	2.913	3.496	2.098	3.28	1.311575 up	6.57E-12
PDIA6	57.434	55.471	70.146	63.546	69.986	443.766	160.773	210.998	172.984	148.341	1.844385 up	3.74E-21
VSNL1	18.663	15.883	23.938	16.742	23.218	5.48	7.677	7.524	14.456	7.951	-1.19192 down	7.44E-07
RHOB	166.363	172.302	92.283	194.081	174.943	9.105	18.289	6.898	99.604	38.484	-2.21432 down	1.13E-22
APOB	1455.796	1551.823	478.735	607.197	484.529	491.842	2407.882	2411.659	2860.176	2346.838	1.200099 up	5.91E-10
TP53I3	0.334	0.656	0.48	1.06	1.572	0.144	0.744	0.196	0.23	0.126	-1.50702 down	3.36E-10
SLC5A6	43.773	41.637	47.298	34.546	44.871	24.558	18.257	15.465	19.786	27.564	-1.00586 down	2.89E-05
MPV17	1.493	1.597	1.738	1.888	2.703	0.64	1.328	0.627	1.077	0.476	-1.18219 down	8.96E-07
DTNB	12.246	11.435	2.323	2.942	2.779	0.655	1.487	1.946	7.326	4.086	-1.03311 down	5.94E-06
KIF3C	1.136	1.361	1.274	1.236	1.068	1.242	3.062	7.431	7.153	7.911	2.140305 up	5.33E-26
HADHB	362.649	336.843	399.756	407.366	334.114	142.64	68.215	202.13	227.903	233.517	-1.0739 down	7.16E-06
LOC12111	6.675	7.37	25.456	52.844	35.568	2.325	3.537	7.855	18.203	12.828	-1.51516 down	1.02E-10
GVINP1	13.324	13.946	18.782	19.544	16.333	2.139	5.369	3.367	7.264	6.636	-1.72529 down	3.51E-14
LOC77027	0.949	1.031	0.455	0.826	1.47	0.055	0.471	0.803	0.161	0.131	-1.54234 down	1.08E-10
LOC77099	0.509	0.541	1.469	0.349	0.762	0.761	1.22	2.549	3.276	4.545	1.765185 up	2.49E-18
PBK	0.135	0.159	0.086	0.133	0.138	1.785	1.08	2.018	1.876	1.048	3.573924 up	5.31E-41
CLU	11.367	12.74	10.805	12.473	30.681	69.891	28.592	17.483	41.302	39.165	1.331215 up	1.32E-12
LOC42638	0.052	0.183	0.718	0.776	0.394	1.849	1.311	0.89	1.048	0.234	1.326531 up	1.05E-11
MSRA	11.61	11.261	12.555	13.88	16.305	4.444	5.524	5.835	4.83	6.136	-1.29321 down	2.61E-08
SOX7	3.242	3.065	2.072	1.882	2.475	2.256	63.323	45.753	34.253	65.192	4.048201 up	6.19E-71
XKR6	0.687	0.589	1.533	0.802	0.901	0.141	0.362	0.446	0.268	0.58	-1.32577 down	2.84E-08
FDFT1	37.273	38.699	4.171	6.444	60.069	115.429	121.862	135.35	27.691	126.706	1.845435 up	3.62E-20
AvBD13	65.643	71.449	112.278	364.193	337.728	146.334	39.63	35.837	82.114	169.176	-1.00776 down	1.89E-05
AvBD7	0.154	0.656	0.922	7.927	0	0.048	0.555	1.024	0.662	0.14	-1.98929 down	1.79E-12
DEFB4A	0.275	0.763	1.804	6.43	0	0.17	0.141	0.696	0.458	0	-2.65784 down	7.09E-19
AvBD1	0.883	0.525	1.279	6.927	0.165	21.023	0.33	4.824	2.756	0.944	1.61078 up	4.46E-14

MCM3	0.205	0.191	0.262	0.324	0.466	1.05	2.855	1.039	1.576	0.483	2.269968	up	1.44E-26
CRISP2	0.086	0.075	2.056	1.533	0.56	0.071	0.413	0.067	0.109	0.13	-2.44033	down	1.60E-16
RHAG	1.114	1.21	2.461	0.895	1.691	0.22	0.104	0.257	0.072	0.206	-3.09374	down	2.44E-28
CYP2AC1	632.367	539.155	1236.022	975.454	1033.102	371.257	73.5	106.195	364.256	214.432	-1.96691	down	3.11E-17
SUPT3H	5.372	5.091	5.659	4.89	14.773	2.118	4.63	1.843	3.711	2.07	-1.31579	down	2.44E-08
RCAN2	2.931	2.809	2.735	5.073	3.064	0.582	1.027	0.942	1.348	1.633	-1.58548	down	3.06E-12
CYP39A1	48.217	48.5	61.485	66.397	81.454	14.97	27.391	34.07	34.469	39.089	-1.0289	down	1.72E-05
ADGRF5	2.642	2.89	2.883	2.995	1.282	0.366	1.204	1.079	0.782	1.134	-1.47422	down	1.03E-10
ARR3	1.942	1.912	0.361	1.283	1.343	0.461	0.056	0.496	0.329	0.164	-2.17976	down	2.81E-18
KIF4B	0.161	0.146	0.335	0.14	0.257	1.595	1.508	1.529	1.895	0.931	2.837634	up	4.05E-38
RAB33A	0.709	0.497	1.612	0.333	0.364	0.031	0.52	0.158	0.338	0.229	-1.4583	down	1.23E-05
CCNB3	0.287	0.137	0.458	0.193	0.295	3.138	0.967	1.448	1.993	1.394	2.70165	up	3.66E-31
CENPI	0	0.033	0.081	0.052	0.144	0.379	0.407	0.578	1.023	0.393	3.144254	up	2.72E-33
GCNA	0.329	0.225	0.656	0.406	0.169	1.316	0.892	0.689	0.928	0.636	1.319024	up	7.94E-11
SLC6A14	0.466	0.499	0.399	2.202	1.155	11.877	0.742	1.359	0.733	0.432	1.680436	up	2.39E-17
GPC3	0.465	0.397	0.123	0.324	0.362	0.044	1.173	2.079	0.565	0.421	1.354946	up	3.30E-10
CLCN5	11.063	10.114	7.517	4.739	6.028	12.946	8.378	32.656	17.207	29.231	1.347408	up	1.38E-12
ZNF185	1.259	0.894	0.224	0.225	0.477	0.221	4.708	3.388	1.185	4.727	2.206466	up	5.92E-25
NSDHL	29.648	29.047	3.061	3.52	36.354	38.351	51.339	82.928	27.677	63.714	1.377217	up	1.46E-12
SLC16A2	78.76	83.669	98.773	104.677	105.799	27.254	35.698	33.335	38.828	42.829	-1.40635	down	1.01E-09
COL4A5	0.178	0.12	1.061	0.118	0.092	0.04	0.142	0.124	0.161	0.148	-1.3441	down	2.13E-06
LOC12111	1.993	1.467	3.592	1.296	2.075	5.379	2.247	5.939	8.868	3.729	1.327286	up	1.04E-12
SH2D1A	1.818	1.263	2.994	1.244	1.728	4.992	2.135	5.499	9.047	3.83	1.494641	up	1.18E-15
IDS	14.08	11.138	9.806	16.463	11.422	8.069	5.933	4.097	6.051	5.102	-1.1046	down	2.03E-06
TRIM2	2.203	2.405	3.025	2.851	3.198	18.64	3.288	2.699	3.377	3.917	1.221926	up	3.29E-11
MSMO1	201.642	197.348	11.709	32.025	267.106	457.78	367.79	616.2	234.458	576.015	1.66581	up	4.22E-17
LOC10705	17.215	17.186	21.674	18.042	33.892	6.275	11.108	11.015	2.19	4.947	-1.6037	down	2.88E-12
DDX60	2.713	2.607	4.405	23.13	14.791	22.66	19.792	21.853	10.182	26.057	1.07732	up	1.59E-08
TBC1D9	0.493	0.451	0.835	0.353	1.96	0.976	1.715	1.78	2.283	4.451	1.452146	up	2.42E-14
DCLK2	0.67	0.617	0.743	3.728	0.698	2.193	4.495	5.361	2.807	4.341	1.571427	up	1.43E-14
SH2D4A	1.039	1.725	1.69	1.879	5.213	8.681	5.475	4.166	6.916	2.829	1.281114	up	5.95E-12
RBPMS	3.35	3.686	11.397	2.669	12.511	2.105	4.955	3.05	2.468	3.718	-1.04427	down	8.96E-05
MMRN1	2.569	2.953	0.155	0.021	0.035	0	0.313	0.079	0.25	0.604	-2.19747	down	7.50E-22
ARHGEF38	1.002	0.876	0.197	0.258	0.592	0.102	0.254	0.286	0.136	0.21	-1.56104	down	7.95E-11
LRP2BP	1.878	1.793	1.608	1.752	1.209	0.691	0.981	0.531	0.639	0.578	-1.26742	down	8.44E-08
ACSL1	167.114	151.745	183.9	262.414	249.984	66.898	29.126	34.108	93.69	34.188	-1.97618	down	5.99E-18
HMGB2	3.299	3.25	3.424	2.955	5.749	10.511	7.619	7.059	9.651	5.967	1.127345	up	1.62E-09
VEGFC	7.835	8.058	18.325	5.016	10.031	6.869	3.839	4.323	3.806	4.218	-1.09532	down	1.89E-05

EREG	1.265	1.313	1.633	0.43	0.762	0.27	0.314	0.17	0.832	0.868	-1.13702	down	0.000105
AREG	1.735	1.123	8.198	0.737	0.521	1.69	0.641	0.928	1.209	0.811	-1.22118	down	1.98E-05
SPARCL1	1.821	1.631	1.46	1.346	1.218	0.439	0.716	0.687	0.968	0.906	-1.00754	down	4.41E-05
PLACL2	2.75	3.512	6.256	7.048	3.026	21.4	7.535	7.357	3.871	13.381	1.244728	up	1.26E-10
GPAT3	7.264	7.386	8.397	9.107	17.669	4.139	2.706	2.076	3.05	3.777	-1.66133	down	7.32E-13
DUSP4	0.599	1.122	0.629	0.47	1.118	1.264	1.706	2.209	1.765	2.043	1.189348	up	5.97E-09
LOC43030	0.074	0.101	0.177	0.084	0.065	0.131	1.92	0.138	1.493	0.077	2.895057	up	4.44E-37
CL2	1028.312	1066.627	1413.226	986.686	1378.971	363.743	595.927	562.662	392.791	536.209	-1.26073	down	7.15E-08
SHROOM3	0.496	0.407	1.226	0.995	0.284	0.087	0.239	0.229	0.448	0.167	-1.53838	down	3.35E-10
DCK	0.47	0.517	0.913	0.896	0.888	2.071	1.739	1.203	2.564	1.071	1.229971	up	3.43E-10
LOC10085	1.014	1.366	0.19	1.03	0.368	0	0.061	0.279	0.228	0.486	-1.90753	down	3.13E-09
ADAMTS3	1.836	1.893	2.519	1.536	1.694	3.998	3.034	4.624	2.541	4.819	1.004178	up	2.71E-08
SULT1B	30.586	33.569	49.609	13.958	87.131	24.043	6.326	5.886	10.696	15.244	-1.7884	down	3.35E-14
CCNA2	1.02	1.09	1.575	1.403	1.133	3.286	4.728	4.76	5.718	4.16	1.863579	up	5.13E-20
MAD2L1	0.749	0.791	1.49	1.444	1.361	2.335	4.114	2.135	1.813	1.979	1.084088	up	3.53E-08
FABP2	0	0	0	1.883	0.102	0	0.205	0.389	0	0	-1.73214	down	0.0003
PITX2	0.311	0.445	0.449	0.399	0.359	1.13	1.258	0.455	0.889	0.931	1.246074	up	5.15E-09
ENPEP	8.84	8.815	9.553	7.139	5.576	52.778	26.94	23.693	14.734	17.03	1.759409	up	2.68E-19
ADH6	295.945	317.948	438.512	463.922	567.162	212.589	217.939	204.379	174.263	224.155	-1.0117	down	2.52E-05
LOC10085	129.699	135.168	237.407	218.604	184.546	76.749	71.761	89.239	61.556	93.384	-1.2052	down	3.47E-07
EMCN	1.074	1.237	2.19	2.072	1.795	0.559	0.692	1.096	0.997	0.816	-1.00743	down	9.12E-05
SLC39A8	8.625	7.925	12.99	9.173	10.658	1.524	3.958	5.67	4.437	5.409	-1.23321	down	2.45E-07
CENPE	0.039	0.025	0.116	0.1	0.115	0.684	0.385	0.573	1.086	0.405	2.971773	up	1.25E-39
CCDC110	0.033	0	0	0.26	0.017	1.527	0.103	0.781	0.827	1.914	4.033108	up	9.40E-47
CYP4V2	99.216	95.434	171.671	142.949	178.717	52.255	56.873	55.711	69.439	76.305	-1.14739	down	1.72E-06
PDGFRL	1.353	1.265	4.402	0.842	1.012	0.622	0.238	0.196	0.774	0.227	-2.10635	down	8.24E-16
UGDH	74.996	73.369	72.6	137.357	137.927	46.843	31.796	68.004	49.27	34.418	-1.10734	down	2.69E-06
FAM114A1	3.049	2.595	2.04	3.165	2.268	6.106	5.775	8.629	5.441	5.437	1.258455	up	5.39E-11
TLR1A	0.625	0.963	1.381	1.748	1.429	6.965	1.792	1.66	2.144	1.486	1.191883	up	1.74E-10
RBPJ	16.543	15.854	18.495	3.857	20.887	2.063	3.947	2.814	4.029	2.018	-2.34618	down	2.12E-24
SEL1L3	23.422	22.452	35.091	17.068	33.257	11.22	15.256	15.706	15.191	7.908	-1.00797	down	5.23E-05
SOD3	16.372	15.254	33.48	40.258	10.83	147.417	13.285	14.556	43.112	17.665	1.022434	up	9.31E-09
PPARGC1A	6.235	5.475	3.024	3.071	5.326	2.419	0.699	0.729	1.539	0.855	-1.88913	down	1.46E-16
NCAPG	0.103	0.105	0.31	0.132	0.412	0.794	0.741	0.902	1.114	0.507	1.928985	up	1.11E-19
BST1	21.71	22.562	2.829	13.861	10.918	70.223	20.699	29.251	14.932	19.403	1.103966	up	1.01E-08
HAUS3	0.566	0.536	0.789	0.756	0.582	1.928	1.569	0.726	1.938	1.655	1.274033	up	6.13E-11
SLBP	0.132	0.245	0.946	0.56	0.226	1.974	1.605	1.665	1.405	1.432	1.935451	up	1.29E-19
SPON2	0.232	0.346	0.632	3.372	0.765	48.742	2.278	2.027	2.446	1.167	3.404308	up	7.99E-53

LOC10704	0	0.035	0.028	0.101	0.109	0.763	0.453	1.058	0.857	0.717	3.792825	up	1.26E-40
LOC1211C	0.089	0.136	0.141	0.024	0.061	0.587	0.548	1.34	1.299	0.937	3.370458	up	9.34E-32
SMYD1	1.567	1.467	0.174	1.386	0.571	21.494	5.464	4.057	2.271	1.884	2.766314	up	1.59E-36
FABP1	424.289	415.437	249.482	445.535	87.222	1668.792	464.842	900.541	872.895	568.93	1.464465	up	5.15E-14
ADRA1D	0.198	0.328	0.399	0.206	1.296	0.183	0.398	0.219	0.087	0.186	-1.17379	down	7.54E-06
CYP26B1	1.016	1.078	0.778	0.472	1.063	0.095	0.64	0.232	0.265	0.632	-1.23917	down	2.50E-07
NAT8	10.089	11.661	7.855	21.419	23.949	2.255	3.975	4.33	2.833	2.686	-2.22084	down	1.09E-21
NAT8B	7.852	7.248	6.827	8.669	7.718	3.071	3.911	2.97	2.94	3.006	-1.26876	down	1.01E-07
MRGPRH	0.016	0.085	0.091	0.067	0.201	1.109	0.485	0.381	0.264	0.309	2.456891	up	3.14E-22
LOC10705	0.718	0.515	1.035	0.787	0.738	2.054	3.761	1.388	1.566	2.227	1.534323	up	6.74E-13
TMEM109	8.362	9.296	10.342	13.653	11.82	1.787	5.514	3.378	4.658	4.45	-1.43403	down	6.20E-10
VWCE	31.219	34.038	39.141	35.891	56.418	10.154	19.796	16.309	13.181	20.782	-1.29393	down	2.32E-08
DAGLA	1.444	1.387	0.965	0.936	0.73	1.225	4.056	4.884	2.731	3.054	1.545187	up	3.80E-15
TCIRG1	11.835	12.294	15.393	16.677	12.762	78.143	57.671	41.009	25.254	26.582	1.729272	up	1.13E-18
ALDH3B1	0.558	0.584	0.83	1.865	1.491	0.898	0.707	0.247	0.461	0.151	-1.11102	down	1.87E-05
SPTB	1.182	1.253	2.213	0.42	1.11	0.221	0.451	0.41	0.258	0.502	-1.74312	down	2.76E-13
NUDT8	6.356	7.809	6.482	5.073	7.233	1.982	3.619	4.461	2.442	2.15	-1.16884	down	1.64E-06
BUB1B	0.043	0.084	0.136	0.237	0.11	1.679	0.68	1.05	1.497	1.014	3.268157	up	7.35E-43
C15orf52	0.882	0.954	1.441	0.955	1.406	0.279	0.574	0.33	0.696	0.57	-1.20133	down	1.45E-06
KNSTRN	0.253	0.165	0.46	0.831	0.89	2.525	1.632	2.169	2.203	1.404	1.932226	up	5.54E-18
CHST14	8.908	8.626	8.228	7.686	11.307	5.04	3.545	4.702	3.655	2.939	-1.17046	down	9.15E-07
ANO9	5.533	4.989	3.658	2.954	6.964	1.517	2.703	1.459	4.951	0.749	-1.08221	down	7.83E-06
LOC77061	77.416	82.614	158.739	190.458	73.271	288.195	237.673	343.976	146.573	444.416	1.326459	up	2.70E-12
DHCR7	20.697	21.731	2.016	2.903	33.831	51.821	61.61	84.723	42.939	93.965	2.045183	up	6.00E-24
E2F8	0.192	0.203	0.289	0.412	0.164	1.011	1.646	1.167	1.517	0.579	2.227678	up	2.80E-24
RCN1	5.229	5.745	5.444	5.877	7.058	18.228	13.43	17.815	10.119	9.638	1.237748	up	4.36E-11
PRRG4	1.373	1.063	4.372	1.151	2.014	7.909	5.701	6.154	4.235	5.371	1.557766	up	7.05E-17
MICAL2	0.179	0.201	0.409	0.404	0.156	3.022	0.361	0.548	0.328	0.229	1.730451	up	4.85E-19
AMPD3	1.358	1.168	1.381	0.697	1.199	0.445	0.64	0.447	0.498	0.853	-1.00797	down	4.01E-05
ST5	10.817	9.261	13.448	11.43	12.529	3.81	6.607	4.69	6.223	7.317	-1.00468	down	2.88E-05
CALCA	3.594	2.378	11.033	2.739	2.269	0.696	1.156	2.21	2.107	2.561	-1.3338	down	2.11E-07
NUCB2	19.675	18.225	28.375	27.841	26.696	87.822	45.066	61.784	46.451	31.262	1.17285	up	1.45E-10
SAA	18.068	17.014	34.513	38.923	20.811	17533.14	98.53	115.818	58.865	126.704	7.115376	up	#######
TMEM86A	132.445	119.628	82.819	229.35	159.732	49.855	29.87	28.558	89.361	48.462	-1.55664	down	5.47E-12
KCNQ1	1.006	0.859	0.601	0.536	0.536	1.902	1.11	1.017	1.634	1.591	1.034799	up	6.54E-08
TRPM5	0.198	0.373	0.151	0.965	0.144	0.558	3.975	0.606	34.832	8.85	4.733012	up	1.24E-86
DUSP8	0.615	0.733	1.304	0.308	0.427	0.431	1.657	2.161	2.971	2.931	1.582124	up	8.54E-17
PNPLA2	44.613	45.171	100.424	37.369	99.724	16.314	17.955	17.458	37.796	32.204	-1.42693	down	3.13E-09

CHKA	20.577	15.45	22.142	9.008	3.506	34.36	51.891	114.759	47.717	44.835	2.054153	up	3.91E-25
TESMIN	0.48	0.358	1.521	0.412	0.316	0.216	0.306	0.293	0.375	0.311	-1.03782	down	0.000107
CPT1A	107.933	94.737	63.556	70.728	127.52	17.973	18.561	17.805	60.026	27.668	-1.70934	down	7.51E-14
FADS1	281.971	282.416	25.638	71.227	106.549	287.946	372.25	309.739	272.887	314.344	1.020114	up	1.49E-07
SMTNL1	0.484	0.481	0.17	1.098	0.795	0.18	0.338	0.091	0.129	0.053	-1.92991	down	1.23E-12
RTN4RL2	0.794	0.567	0.805	1.053	0.575	0.469	0.185	0.549	0.293	0.18	-1.1763	down	1.70E-05
PGR2/3	0.309	0.121	0.147	1.826	0.127	0	0	0.096	0.118	0	-3.53298	down	2.15E-14
INCENP	0.18	0.151	0.358	0.32	0.224	0.97	0.739	0.738	1.127	0.647	1.771281	up	9.47E-19
SMIM38	1.844	1.327	0.147	1.819	0.474	0	0.095	0.072	0	0	-5.02906	down	3.30E-24
FGF19	0.264	0.104	0.196	0.174	0.036	5.234	10.047	3.423	1.579	1.912	4.832793	up	1.63E-78
EHF	0.254	0.388	0.934	0.186	0.392	2.012	0.566	0.805	0.781	0.971	1.251405	up	6.01E-11
ACCS	6.002	4.903	2.753	3.512	1.412	104.894	1.909	8.053	2.509	5.535	2.725178	up	1.22E-38
MDK	0.867	1.267	3.207	3.519	3.744	0.174	1.808	0.696	0.389	0.83	-1.69217	down	3.12E-12
CHAC1	25.729	26.957	18.29	109.452	33.208	1.616	5.083	3.38	24.82	15.646	-2.07941	down	7.57E-21
NUSAP1	0.097	0.255	0.245	0.373	0.183	2.189	1	2.063	2.212	0.983	2.867658	up	2.94E-35
LTK	0.571	0.468	1.745	0.501	0.445	0.329	0.182	0.241	0.231	0.291	-1.54609	down	1.90E-09
SPTBN5	0	0	0.003	0.035	0	2.107	0	0.003	0.009	0	5.626303	up	6.07E-84
EHD4	0.987	0.714	1.411	1.028	0.875	3.094	1.846	1.617	2.123	1.641	1.040522	up	9.81E-09
PCNX1	13.58	13.415	13.087	12.267	14.613	4.699	5.872	5.926	6.307	6.101	-1.21188	down	1.97E-07
PLEKHD1	1.744	1.588	1.985	2.026	0.927	0.748	0.584	1.126	0.568	1.004	-1.03619	down	1.70E-05
GALNT16	0.883	0.742	1.649	1.57	0.711	0.316	0.579	0.517	0.261	0.42	-1.40607	down	5.52E-09
RDH11	4.659	5.179	3.221	1.487	5.496	6.538	16.433	16.495	6.004	16.667	1.632182	up	1.43E-16
LGALSL2	0.62	0.766	1.617	0.324	1.405	0.105	0.333	0.066	0.968	0.714	-1.11238	down	4.71E-05
LOC11253	1.76	1.317	4.659	1.746	0.481	0.62	0.452	0.745	0.723	0.923	-1.5232	down	8.01E-10
SLC25A21	0.987	0.983	1.093	1.166	1.457	0.571	0.546	0.37	0.35	0.422	-1.32981	down	1.57E-08
TTC6	0.807	0.437	2.69	0.156	0.257	0.052	0.224	0.409	0.858	0.138	-1.36807	down	5.05E-07
ACSS1B	42.926	41.52	46.602	47.98	76.447	15.807	16.545	13.258	4.498	3.762	-2.24552	down	5.01E-23
TMEM63C	0.238	0.301	1.349	0.324	0.818	0.319	0.285	0.192	0.197	0.212	-1.32669	down	3.67E-07
SPIA3	23.911	21.353	55.387	26.868	31.373	3157.216	9.092	24.019	10.898	10.597	4.33723	up	1.28E-82
LOC10704	0.496	0.568	1.415	0.464	0.156	10.313	0.028	0.183	0.026	0.062	1.774176	up	9.70E-20
BDKRB1	0.133	0.237	0.303	0.219	0.098	0.684	0.157	4.415	0.182	0.052	2.465351	up	3.09E-26
LOC12111	3.707	3.001	0.024	3.645	0	0	0.062	0.024	0	0	-6.834	down	5.50E-72
AMN	2.966	4.119	6.067	4.949	11.505	0.381	1.266	1.513	0.979	0.351	-2.71974	down	1.34E-29
BRF1	7.157	6.375	4.736	7.189	3.954	2.6	2.314	2.458	2.801	2.71	-1.19057	down	1.93E-07
MTHFD1	168.316	161.333	234.28	152.415	343.279	75.503	94.808	104.881	121.571	122.981	-1.02767	down	2.78E-05
SYNE2	5.652	5.467	11.555	7.251	7.614	3.259	3.689	3.133	4.046	3.757	-1.06951	down	1.34E-05
DHRS7	868.149	797.527	1003.922	724.93	1188.741	543.382	290.476	346.592	456.426	369.27	-1.19195	down	6.16E-07
DLGAP5	0.257	0.168	0.303	0.322	0.349	2.288	1.005	1.47	2.965	1.536	2.722871	up	1.34E-35

GCH1	87.373	94.623	99.629	67.794	186.64	45.496	29.126	36.377	59.513	37.863	-1.36319	down	9.04E-09
VCPKMT	16.614	15.893	17.436	15.95	18.958	7.643	10.068	8.196	6.795	9.665	-1.00191	down	2.88E-05
SOS2	9.302	9.379	11.862	14.758	15.676	3.667	4.791	5.032	5.219	5.137	-1.35433	down	5.14E-09
LOC10705	0.159	0.295	0.232	0.327	0.436	1.264	0.926	0.352	0.329	0.479	1.206285	up	1.34E-07
STYX	7.574	7.182	10.168	10.094	14.456	5.988	3.471	4.773	5.442	5.045	-1.0009	down	5.73E-05
LOC11253	2.635	2.219	2.157	2.217	1.423	0.676	1.09	0.188	0.451	1.073	-1.61326	down	2.36E-12
LOC12110	0.982	0.989	1.19	1.01	0.727	0.487	0.327	0.711	0.54	0.224	-1.0958	down	1.70E-05
GRID1	0.591	0.53	1.061	0.147	0.01	0.091	0.084	0.102	0.129	0.109	-2.17239	down	3.24E-19
SNCG	0.037	0.016	0.07	0.041	1.68	0.015	0.026	0.058	0.06	0.056	-3.07117	down	1.42E-21
DYDC2	0.206	0.215	0.459	0.034	0.465	1.197	0.719	0.385	0.902	0.446	1.400633	up	5.25E-09
SFTPA1	0.947	1.696	3.772	4.466	3.739	4.136	0.769	0.584	0.999	0.361	-1.09342	down	7.71E-05
RET	0.1	0.092	0.218	0.131	0.103	0.095	1.48	0.031	0.064	0.145	1.487648	up	3.02E-11
DNAJC12	47.04	41.616	64.729	49.829	55.959	11.915	14.405	8.946	38.301	30.537	-1.31585	down	2.54E-08
RHOBTB1	21.204	18.61	41.885	14.017	17.196	5.538	10.305	8.888	16.182	10.498	-1.13497	down	5.17E-06
CDK1	1.008	0.716	8.736	0.805	1.597	8.842	3.492	5.91	6.948	3.412	1.152793	up	6.87E-12
RNASE6	2.549	3.117	6.954	5.151	1.073	12.805	6.485	5.348	7.014	10.052	1.145871	up	8.31E-10
PAOX	20.467	22.313	19.85	23.818	24.42	12.288	8.817	7.795	10.319	11.735	-1.1215	down	1.76E-06
PAPSS2	103.418	92.74	139.544	99.126	136.152	15.978	32.503	34.112	65.485	53.173	-1.50442	down	1.06E-10
PRKG1	0.334	0.412	0.524	1.475	0.744	0.369	4.307	0.522	3.949	5.579	2.075904	up	1.58E-23
HKDC1	0.042	0.172	0.528	0.207	0.076	1.387	32.799	33.195	1.966	8.901	6.24743	up	#######
NPFFR1	0.055	0.18	0.088	0.113	0	1.245	0.028	0	0	0	1.535037	up	7.50E-08
CYP2C18	1704.444	1566.283	682.717	855.24	1306.675	165.665	125.172	249.372	244.733	306.652	-2.486	down	2.44E-28
SCD	143.146	170.585	16.981	51.148	16.442	32.762	573.02	718.921	468.663	673.992	2.631017	up	8.47E-35
CYP2C23b	588.344	527.607	295.271	352.718	570.35	7.027	91.417	129.965	195.161	225.046	-1.84754	down	1.80E-16
CYP2C23a	1174.55	1044.819	1141.807	758.226	1003.909	48.417	372.047	354.753	363.186	616.023	-1.54607	down	1.01E-11
MSMB	5.047	4.939	8.814	1.882	4.879	9.452	2.223	27.339	4.975	19.062	1.302407	up	1.78E-12
LOC11253	0	0	0	0	0	0.843	0.144	0	1.566	0	8.998872	up	5.34E-74
ANKRD22	0.151	0.049	0.06	0.248	0.039	0.91	1.899	0.677	0.144	0.273	2.82369	up	1.68E-20
PANK1	87.26	75.486	129.212	87.423	116.202	28.022	21.038	28.686	79.692	39.349	-1.33247	down	2.79E-08
KIF11	0.544	0.55	1.01	0.769	0.515	1.802	1.134	1.355	1.644	1.028	1.038183	up	8.11E-09
CPN1	48.282	50.567	48.407	36.398	76.073	32.849	14.685	17.223	26.066	23.959	-1.17806	down	8.06E-07
LOXL4	0.866	0.908	1.01	0.969	0.905	0.444	0.015	0.408	0.413	0.959	-1.05519	down	5.11E-05
CRTAC1	6.912	8.008	0.079	0.244	0.017	0.031	0.017	0.013	0.503	0.224	-4.26676	down	1.42E-60
PI4K2A	22.469	22.979	17.273	12.637	18.764	4.447	5.01	6.911	7.468	11.362	-1.41891	down	5.90E-10
HOGA1	4.35	4.49	2.699	2.135	5.657	0.21	0.29	0.727	0.323	0.434	-3.28117	down	3.23E-38
ANKRD2	0.61	0.412	1.112	0.388	0.283	0.183	0.222	0.2	0.075	0.213	-1.64578	down	1.38E-08
TLX1	0.975	1.072	0.882	0.947	0.046	0	0	0.069	0	0.04	-5.10632	down	2.46E-30
PDZD7	1.084	0.786	1.142	1.255	0.335	0.217	0.24	0.446	0.133	0.348	-1.72978	down	2.38E-12

CYP17A1	1.837	2.736	1.617	3.953	6.331	0.239	0.025	4.15	0.323	0.7	-1.59842	down	5.70E-11
GSTO2	5.481	5.978	4.51	2.385	10.692	1.311	1.382	2.222	2.431	2.866	-1.50762	down	9.94E-11
GPAM	50.126	44.294	35.149	35.867	21.312	39.44	137.985	178.206	64.14	160.356	1.63525	up	1.40E-16
LOC10175	0.605	0.138	0.27	0.482	0.762	4.56	0.706	0.715	1.704	1.243	1.981546	up	4.28E-22
FAM196A	0.873	1.004	4.073	1.217	0.36	1.055	0.468	0.572	0.832	0.832	-1.00077	down	0.000247
MKI67	0.168	0.176	0.41	0.273	0.327	1.889	1.708	1.752	3.157	1.326	2.855673	up	3.25E-41
COL3A1	4.995	6.107	6.011	9.004	10.436	11.384	23.038	13.537	17.14	14.299	1.119006	up	2.25E-09
ABCA12	5.11	4.197	11.634	1.146	27.632	0.245	0.063	0.218	1.861	0.335	-4.18856	down	4.01E-65
ATIC	4205.008	4235.959	2567.995	3001.741	2519.42	475.647	423.327	568.808	874.71	929.657	-2.33679	down	2.53E-25
LOC10704	2.429	8.065	4.108	4.732	2.095	0.037	2.373	0.702	1.974	3.336	-1.34681	down	4.55E-09
TRPM8	0.23	0.244	0.404	0.094	0.069	1.031	0.62	0.142	0.173	0.294	1.114628	up	3.15E-08
UGT1A1	39.561	37.223	44.172	46.488	71.827	13.279	36.749	20.206	19.776	26.474	-1.03848	down	9.61E-06
LOC12111	5.572	5.416	23.484	11.677	68.075	0.651	2.966	1.921	1.835	3.683	-3.36838	down	8.54E-46
AHR2	5.188	4.649	7.267	7.84	9.885	1.203	0.521	0.778	1.539	2.338	-2.44796	down	2.39E-26
AHR1B	4.608	3.848	6.309	2.694	3.261	1.479	0.452	0.599	0.42	1.474	-2.22632	down	1.33E-21
SLC19A1	67.212	62.132	29.781	38.601	23.339	11.338	16.286	24.189	14.066	19.156	-1.37829	down	7.04E-10
LOC76858	0.209	0.219	1.432	0.791	2.062	0.337	0.086	10.446	0.399	4.918	1.778945	up	7.32E-18
COQ10B	31.709	32.659	22.38	37.282	58.093	12.956	9.487	6.341	47.728	9.949	-1.07475	down	9.65E-06
AOX1	0.818	0.819	1.758	1.008	6.053	0.58	0.136	0.147	0.654	0.233	-2.57548	down	2.05E-26
RAPH1	8.514	7.701	11.651	7.638	9.274	2.287	4.518	3.378	5.159	4.795	-1.15274	down	1.34E-06
LOC42411	16.941	16.13	19.558	15.841	26.377	6.862	7.393	10.009	8.658	14.466	-1.001	down	3.38E-05
IDH1	256.182	246.123	290.413	244.149	481.266	121.886	111.481	149.658	154.347	195.754	-1.05016	down	1.33E-05
PPP1R1C	0.281	0.444	0.205	0.749	0.192	0.304	1.754	1.095	0.334	0.571	1.114886	up	1.76E-06
OSBPL6	2.165	2.21	1.961	1.498	0.993	0.508	0.633	1.016	0.692	1.468	-1.03104	down	1.20E-05
PDE11A	19.234	16.791	0.348	4.895	0.061	5.129	36.192	38.257	38.238	39.102	1.924656	up	2.16E-20
CDCA7	0.208	0.484	1.145	0.514	0.696	1.574	3.963	1.83	2.048	1.178	1.795965	up	2.48E-19
RAPGEF4	21.935	20.538	17.359	11.14	36.575	8.74	7.422	7.912	11.355	6.065	-1.37389	down	4.97E-09
G6PC2	2.067	1.947	0.812	2.106	1.925	0.734	0.637	0.677	1.183	0.486	-1.25155	down	1.61E-07
SPC25	0.417	0.448	0.332	0.441	0.949	3.024	2.633	2.464	2.869	1.455	2.264008	up	3.12E-24
DPP4	4.234	3.722	3.601	5.071	9.228	10.931	14.524	7.209	18.456	15.304	1.361035	up	9.40E-13
ITGB6	0.971	0.877	0.5	0.447	0.438	3.304	2.566	3.74	0.781	7.292	2.449596	up	2.67E-31
LOC10704	0.24	0.24	0.524	0.572	1.416	0.407	0.364	0.128	0.133	0.275	-1.19175	down	1.10E-05
LOC42419	0.762	0.715	1.71	0.465	1.364	0.024	0.06	0.076	0.186	0.035	-3.7013	down	2.03E-34
TUB4A	3.194	3.174	6.562	4.328	5.309	1.736	1.729	2.112	1.163	2.073	-1.35601	down	3.16E-08
IHH	4.952	4.974	10.832	8.392	14.985	1.768	3.358	3.913	2.391	3.419	-1.57124	down	3.11E-11
CYP27A1	192.862	196.24	98.741	133.035	298.843	60.571	120.034	83.52	88.831	96.453	-1.03316	down	8.30E-06
PLCD4	0.512	0.711	0.995	0.719	1.412	0.333	0.257	0.293	0.25	0.203	-1.69903	down	4.19E-11
IGFBP2	19.814	18.98	11.072	14.401	21.131	0.176	2.549	3.157	7.595	3.272	-2.34978	down	7.23E-25

TMEM169	13.324	12.606	5.022	4.267	2.767	2.43	4.48	2.464	3.363	3.929	-1.18832	down	1.02E-07
PECR	46.492	42.24	62.908	57.024	61.257	15.27	36.676	16.537	39.528	19.577	-1.08101	down	6.03E-06
ARHGEF1	0.476	0.488	0.145	0.235	0.179	0.737	0.302	2.149	0.28	0.639	1.428196	up	4.24E-12
EAF2	6.465	6.932	2.603	3.063	2.765	5.587	6.358	17.998	7.589	9.145	1.096356	up	7.67E-09
SEMA5B	1.118	1.356	3.459	4.95	3.75	1.26	1.34	1.174	1.193	1.395	-1.20103	down	3.92E-07
SLC12A8	0.247	0.246	0.306	0.554	0.298	1.764	1.665	1.504	2.058	0.594	2.196397	up	3.69E-25
SCTR	4.74	4.324	0.287	2.085	0.549	1.017	10.841	8.118	12.48	6.593	1.703639	up	1.65E-16
NXPH2	4.48	3.663	2.488	1.492	3.923	3.788	9.946	6.256	6.431	9.691	1.170015	up	8.79E-10
ASPM	0.611	0.639	0.406	1.148	1.133	2.486	2.557	3.067	2.952	1.839	1.711042	up	7.42E-18
CRIP2	9.622	9.271	13.613	4.971	13.144	5.462	4.187	6.398	4.052	5.063	-1.00835	down	6.74E-05
REG4	0.831	0.502	1.374	0.473	0.853	0.079	0.328	0.1	0.487	0.347	-1.58496	down	5.45E-06
LOC10085	2.765	2.605	3.779	1.831	1.208	0.332	0.488	1.514	1.614	1.008	-1.29735	down	2.62E-07
LOC10705	0.051	0.033	0.067	0.083	0.104	1.53	0.07	0.158	0.097	0.153	2.553067	up	1.20E-21
FASLG	0.394	0.358	0.569	0.236	0.405	0.236	0.899	1.001	0.809	1.13	1.052572	up	2.61E-08
SUCO	6.512	6.015	8.369	5.144	6.028	7.374	27.677	27.087	14.462	20.087	1.592037	up	1.84E-16
FMO3	553.076	547.561	600.864	963.532	1308.063	291.415	220.106	287.027	271.617	382.302	-1.45176	down	2.47E-10
NUF2	0.068	0.122	0.256	0.306	0.226	1.059	1.009	0.925	1.146	0.505	2.241657	up	2.93E-22
RGS8	6.23	6.334	1.454	1.004	4.903	0.405	0.261	0.401	1.104	0.483	-2.90599	down	1.83E-36
RGSL1	1.999	2.292	0.7	0.614	1.794	0.095	0.734	0.686	0.901	0.457	-1.36324	down	8.31E-09
GLUL	216.185	224.988	358.723	324.283	678.3	78.473	160.395	177.021	287.962	142.784	-1.09017	down	8.64E-06
SOAT1	7.702	6.988	20.825	8.325	17.535	6.588	5.689	5.37	4.093	7.184	-1.08525	down	1.63E-05
ABL2	2.044	1.783	3.941	1.973	2.067	2.509	5.819	7.943	7.943	9.002	1.49172	up	2.19E-15
TOR3A	22.63	20.597	13.745	7.774	1.6	27.33	99.054	88.205	84.312	83.008	2.525058	up	1.05E-33
KIAA0040	27.146	26.189	17.705	19.883	28.757	6.182	6.133	7.591	7.736	11.34	-1.61818	down	8.70E-13
RABGAP1I	9.058	8.331	14.313	9.315	13.418	3.817	3.319	4.578	5.008	6.166	-1.24976	down	1.67E-07
SERPINC1	259.983	261.638	354.37	334.689	510.496	105.014	177.229	156.665	184.258	201.798	-1.06099	down	9.15E-06
PLPPR5	8.421	7.441	3.569	4.29	3.863	2.345	17.582	13.955	17.054	14.419	1.244315	up	1.41E-10
ZNF644	6.697	6.357	12.756	6.691	7.442	4.088	3.82	3.677	4.262	4.086	-1.0026	down	6.23E-05
CLCA1	0.1	0.033	0.03	0.164	0.026	91.023	2.58	0.468	1.656	0.847	8.075606	up	#######
SSX2IP	2.355	1.78	1.613	1.374	0.639	2.123	5.317	6.856	5.446	8.871	1.881679	up	1.68E-20
VTG2	4938.704	5884.15	43.669	1565.017	4.114	2321.824	11886.27	11182.5	10342.81	11543.32	1.926648	up	2.65E-20
LOC12111	3.51	7.943	0	1.355	0	0.864	17.34	13.062	2.127	2.403	1.482392	up	3.08E-12
VTG3	625.379	748.039	2.05	89.471	0.449	93.195	1903.229	1798.952	1512.386	1663.628	2.25016	up	2.04E-26
SPATA1	21.159	19.818	12.843	23.628	2.939	12.632	49.046	44.053	34.765	44.51	1.202487	up	7.63E-10
VTG1	2389.735	3147.829	3.494	185.475	1.276	400.174	5307.664	6336.57	7066.791	5409.385	2.097937	up	1.13E-23
PTGFR	0.136	0.159	0.301	0.239	0.279	1.705	0.369	0.393	0.055	0.71	1.532447	up	1.22E-13
ELOVL1	56.467	57.037	41.339	42.21	37.258	23.822	19.02	22.9	17.59	30.924	-1.03612	down	8.30E-06
KIF2C	0.474	0.276	0.561	0.347	0.381	1.356	0.698	0.969	1.624	0.556	1.349334	up	1.09E-11

RBP	330.724	399.221	17.878	55.352	2.696	67.659	970.74	1039.307	858.801	773.354	2.202737 up	1.06E-25
RAD54L	0.394	0.445	0.877	0.581	0.684	1.683	1.564	1.124	0.842	1.315	1.129531 up	4.52E-09
CYP4A22	50.13	52.443	87.296	66.467	105.941	9.518	34.585	26.966	31.732	38.672	-1.35654 down	6.58E-09
CYP4B7	400.359	358.574	324.755	325.616	466.218	16.401	55.709	45.039	182.466	87.448	-2.27664 down	1.71E-23
ORC1	0.188	0.205	0.519	0.258	0.245	1.312	0.49	0.519	0.562	0.374	1.199866 up	1.38E-09
PODN	2.283	1.903	5.682	1.607	1.414	0.461	1.092	1.339	0.885	1.146	-1.38763 down	1.34E-08
DHCR24	22.675	25.84	0.604	0.787	51.673	53.4	105.707	108.54	67.645	95.02	2.082727 up	7.61E-25
DAB1	0.954	0.815	3.121	1.119	2.971	0.636	0.687	0.986	0.991	0.445	-1.26063 down	8.93E-07
KANK4	4.823	5.238	3.25	8.253	10.794	27.16	11.846	14.819	7.479	6.991	1.07754 up	6.52E-09
ANGPTL3	47.918	48.945	26.193	51.599	48.217	39.743	241.244	250.081	195.533	204.778	2.063128 up	4.06E-24
DNAJC6	2.165	1.793	2.369	0.473	3.857	0.352	0.405	1.702	0.982	0.63	-1.38725 down	1.92E-08
NEGR1	0.19	0.195	0.044	0.194	0.14	0.248	0.241	0.652	1.546	0.875	2.215533 up	1.69E-26
CRYZ	91.59	83.779	102.778	67.333	70.775	21.886	44.54	48.473	50.932	41.415	-1.00611 down	3.08E-05
NEU2	1.117	0.817	0.314	0.576	0.788	3.309	1.216	0.667	3.238	1.466	1.452781 up	2.02E-12
KLHL24	23.395	22.179	23.13	14.804	19.244	7.258	8.833	6.678	10.056	9.339	-1.28498 down	3.50E-08
EHHADH	274.411	257.616	291.099	309.829	356.991	87.038	76.719	76.263	92.767	133.005	-1.67749 down	1.61E-13
DNAJB11	20.71	21.364	20.588	17.848	19.015	97.03	41.69	67.917	53.359	38.627	1.585146 up	1.81E-16
SLCO2A1	16.89	18.443	24.963	11.819	17.125	6.009	12.791	6.488	10.659	7.658	-1.03311 down	2.15E-05
TF	3907.862	4254.025	3132.064	7230.778	5040.879	23427.33	7781.489	8600.474	5966.948	6411.189	1.147019 up	1.05E-09
MAB21L4	5.913	6.043	7.571	9.273	15.714	4.114	4.878	4.022	3.971	4.931	-1.02211 down	2.31E-05
OTOS	0.896	1.515	0.892	1.842	1.381	0.185	0.691	0.291	0.784	0.203	-1.59694 down	1.09E-07
SGPP2	0.885	0.962	0.263	1.936	3.467	0.084	0.2	0.023	0.444	0.285	-2.85238 down	1.57E-29
MOGAT1	1.422	1.726	2.128	2.632	8.621	0.891	0.704	0.775	0.768	0.759	-2.08315 down	5.21E-18
PCOLCE2	2.885	2.765	3.479	2.576	4.171	1.661	0.281	0.602	0.544	0.365	-2.19929 down	3.08E-20
PLOD2	13.752	12.993	15.679	16.286	8.696	18.937	40.115	29.525	27.375	32.739	1.14131 up	1.28E-09
LOC7707(	1.785	2.335	3.299	2.082	7.479	1.263	2.407	1.542	0.41	0.677	-1.42992 down	2.58E-09
LOC12111	17.118	17.826	22.987	18.719	32.738	2.247	4.333	9.84	1.154	1.853	-2.49301 down	3.19E-27
BDH1B	4.741	5.446	4.386	2.933	11.844	1.882	2.407	1.371	1.489	1.236	-1.80686 down	6.90E-15
HRASLS	2.403	2.567	1.734	5.152	3.432	26.019	1.389	1.743	2.846	2.037	1.154317 up	9.26E-10
RTP2	5.707	6.898	5.74	12.564	6.633	3.161	0.895	1.641	2.12	3.586	-1.71865 down	8.21E-14
PDCD1	0.224	0.244	0.436	0.245	0.332	1.632	0.511	0.582	0.901	0.652	1.527188 up	4.98E-12
C2orf72	45.018	46.835	41.902	41.539	44.64	19.839	18.958	15.136	29.845	17.249	-1.12229 down	1.98E-06
ALPI	2.276	2.978	1.982	7.011	6.817	0.342	0.678	1.693	0.467	0.943	-2.35161 down	1.79E-23
CLCN2	5.259	5.023	6.373	7.091	8.974	2.317	2.214	2.132	1.733	2.645	-1.56687 down	1.28E-11
HRG	171.057	164.513	402.462	210.89	358.187	40.577	152.224	104.177	57.275	53.039	-1.68223 down	4.17E-13
ECT2	0.326	0.373	0.202	0.082	0.205	0.567	1.136	1.082	1.253	0.631	1.970064 up	5.57E-20
SERPINI1	0.052	0.102	0.316	0.227	0.372	1.517	0.337	1.306	0.971	0.203	2.014369 up	8.13E-20
LOC10705	0.51	0.516	1.065	0.257	2.652	0.244	0.225	0.009	0.073	0.342	-2.47858 down	2.18E-24

SMC4	0.936	0.823	1.281	0.925	1.01	4.269	2.275	2.392	5.16	2.181	1.70906	up	1.90E-18
IFT80	2.329	2.327	3.494	2.548	2.656	9.599	3.548	3.169	12.019	3.048	1.232401	up	1.77E-11
MLF1	1.58	1.77	10.575	6.262	8.683	3.178	1.428	1.564	1.479	1.389	-1.67495	down	6.63E-12
PTX3	0.048	0	0.399	0.059	0.025	0.015	0.662	0.019	3.141	2.266	3.510395	up	1.84E-40
C3orf33	4.902	5.566	11.013	5.524	7.193	2.688	3.166	0.902	2.638	2.723	-1.4965	down	7.17E-10
SUCNR1	10.112	8.721	20.68	8.036	5.365	1.733	3.972	1.826	4.563	4.667	-1.65825	down	2.43E-12
HPS3	51.125	49.911	41.8	53.537	35.407	361.01	42.173	55.499	55.571	43.049	1.265685	up	1.39E-11
CP	208.596	200.646	155.517	210.35	129.634	1475.051	174.479	214.502	216.815	167.289	1.313145	up	3.45E-12
TUBA3E	0.192	0.163	0.031	0	0.099	0.416	0.73	0.734	0.275	1.059	2.715759	up	7.92E-24
SLC28A2	1.9	1.83	2.645	2.501	4.872	1.76	1.254	0.844	0.958	1.09	-1.21827	down	7.19E-07
DUOX1	0.118	0.177	0.05	0.051	0	0.266	0.235	0.578	4.167	0.15	3.751552	up	3.42E-54
KIAA0101	2.021	2.952	1.287	0.996	2.421	3.878	10.729	10.092	4.304	9.502	1.991856	up	4.40E-20
PATL2	0.431	0.311	0.936	0.477	0.321	0.773	2.018	1.242	1.206	0.861	1.29907	up	6.64E-11
CYP1A2	186.343	193.424	80.067	111.37	352.381	4.235	94.355	80.529	38.365	64.01	-1.71412	down	1.61E-14
CYP1A1	6.762	6.585	8.984	8.907	42.226	1.806	10.284	4.237	2.469	4.612	-1.64983	down	7.24E-13
CHRNA3	0.746	0.418	1.464	0.81	0.164	0.512	0.164	0.187	0.178	0.217	-1.51394	down	3.62E-07
TRPM1	0.314	0.362	0.075	0.487	0.137	4.675	2.633	2.566	1.029	0.791	3.083645	up	3.55E-44
CCNB1	1.182	1.152	2.823	1.149	1.659	5.359	3.271	4.836	3.771	2.399	1.301217	up	4.20E-12
LIPC	10.231	9.369	17.981	17.582	21.798	0.965	1.013	1.955	3.913	0.747	-3.16215	down	5.46E-41
SCG3	0.277	0.258	0.134	0.235	0.216	1.118	0.423	0.767	0.561	0.372	1.528738	up	3.32E-13
KIAA1024	0.16	0.137	0.052	0.089	0.035	0.6	0.473	0.629	1.369	0.31	2.824499	up	1.58E-37
MFGE8	138.896	146.087	39.039	75.693	26.068	84.886	346.704	275.943	217.76	184.666	1.382306	up	4.07E-12
PGPEP1L	6.832	6.269	12.13	11.861	8.377	0.922	0.757	2.641	0.831	2.889	-2.49888	down	1.50E-27
ADAMTS1	1.168	1.212	0.396	1.154	0.274	6.267	2.616	1.699	1.826	0.94	1.665614	up	1.67E-16
ALDH1A3	0.407	0.44	0.452	0.311	0.185	0.258	0.622	0.716	3.357	1.172	1.76789	up	7.79E-18
PCSK6	10.296	9.946	18.096	13.867	8.212	5.231	40.489	27.654	24.91	29.974	1.085959	up	3.76E-09
CORO2B	0.557	0.608	0.694	4.147	1.16	0.442	0.382	0.602	0.395	0.207	-1.81856	down	2.38E-14
PAQR5	1.348	1.26	1.339	1.931	1.86	0.692	0.554	0.461	0.857	0.836	-1.18524	down	1.29E-06
KIF23	0.095	0.124	0.14	0.258	0.15	1.76	0.729	0.924	1.394	0.701	2.836165	up	1.32E-36
TMED3	10.988	11.981	10.281	14.372	11.035	41.135	27.101	34.133	20.376	20.564	1.288681	up	1.45E-11
CETP	25.686	27.129	125.438	13.017	74.071	0.258	0.694	1.859	1.798	0.467	-5.70662	down	#######
CX3CL1	8.187	8.904	5.292	11.119	18.702	5.792	3.005	3.464	4.157	2.885	-1.4351	down	7.93E-10
EXOC3L1	1.275	1.501	1.576	3.319	4.536	0.796	1.195	1.059	0.856	1.042	-1.30192	down	3.27E-08
LCAT	92.519	82.067	354.367	283.575	528.767	35.978	21.447	27.958	45.168	48.775	-2.90294	down	1.76E-35
LOC41566	48.739	55.791	52.034	72.387	96.728	7.244	37.508	39.317	18.295	58.324	-1.01917	down	1.10E-05
LOC10174	652.428	670.99	572.533	1307.915	921.024	2.672	132.169	209.586	172.772	311.974	-2.3146	down	3.77E-25
LOC41566	31.199	32.997	32.033	30.862	30.096	4.557	15.633	23.787	16.509	12.196	-1.11276	down	2.49E-06
LOC10085	673.037	787.092	345.96	334.899	411.752	23.65	253.587	270.01	230.453	208.474	-1.37213	down	8.99E-10

LOC10174	10.563	11.234	12.576	16.408	21.977	3.549	10.538	8.143	4.681	4.543	-1.20973	down	1.82E-07
PDPR	9.716	9.018	13.894	7.72	13.274	4.745	5.936	5.048	4.822	4.342	-1.10693	down	5.00E-06
MT4	711.399	809.728	1038.719	734.886	291.299	790.275	114.82	98.884	71.138	299.386	-1.38347	down	2.95E-09
MT3	310.257	378.472	491.141	215.847	100.131	292.4	32.119	44.096	24.081	91.904	-1.62609	down	3.68E-12
GALR1L	2.159	2.201	1.509	3.049	1.385	0.945	0.637	0.995	1.471	0.822	-1.08029	down	3.59E-06
SLC6A2	2.224	2.327	2.448	1.587	1.529	2.166	8.235	6.751	2.235	3.633	1.185992	up	3.81E-10
TOX3	1.191	0.994	0.419	0.881	0.163	0.129	0.602	0.132	0.284	0.251	-1.38057	down	3.76E-09
CDCA9	20.937	20.591	13.664	15.924	17.513	3.863	9.591	5.34	10.571	7.004	-1.28495	down	2.12E-08
RGS9BP	1.13	1.118	2.26	1.351	2.091	0.642	0.595	0.736	0.606	0.819	-1.22505	down	9.10E-07
CES1L2	38.947	39.648	81.674	126.021	111.344	44.47	27.438	1.266	26.709	6.71	-1.89928	down	9.60E-17
CES1L1	111.626	109.5	127.726	130.396	178.969	101.671	53.302	3.67	18.261	39.72	-1.60335	down	1.94E-12
TERB1	3.686	3.169	6.137	3.53	1.611	0.453	2.285	0.464	0.668	0.854	-1.93941	down	8.49E-17
BEAN1	0.894	0.509	0.588	0.831	0.599	0.674	1.958	1.123	2.373	1.477	1.151371	up	1.31E-07
DYNLRB2	2.244	2.109	0.585	1.207	1.131	0.556	0.042	0.764	0.817	0.406	-1.49118	down	2.99E-08
CMC2	1.814	1.737	1.986	2.023	1.73	5.253	3.928	3.69	3.243	2.988	1.039575	up	5.97E-08
CENPN	0.316	0.447	0.405	0.375	0.612	2.268	1.639	1.614	1.722	1.395	2.000501	up	1.09E-20
LOC77659	3.882	4.966	5.974	6.112	5.108	33.608	6.881	7.881	4.909	6.489	1.198377	up	1.08E-10
GINS2	0.197	0.297	0.676	0.237	0.436	0.816	1.589	0.662	1.026	0.439	1.295774	up	1.52E-10
SLC7A5	13.905	12.708	34.749	16.312	13.231	5.969	7.827	10.748	6.152	9.131	-1.19051	down	1.25E-06
CA5A	214.2	216.023	174.242	126.055	187.64	42.432	56.017	62.632	39.507	44.837	-1.90344	down	3.64E-17
ZFPM1	15.468	15.391	15.633	9.886	14.325	1.085	9.267	5.699	4.415	4.581	-1.49695	down	4.78E-11
CIDEC	25.144	27.625	46.895	23.439	53.649	11.107	8.515	11.44	12.977	20.231	-1.45944	down	7.74E-10
MVD	13.917	17.885	4.657	2.51	30.493	14.725	43.952	30.992	20.145	32.632	1.036066	up	3.27E-08
CDT1	0.437	0.767	1.142	0.632	1.276	1.386	6.956	1.611	2.686	1.265	1.707432	up	6.61E-17
SPIRE2	9.4	10.136	19.47	2.665	10.706	8.987	2.055	0.75	0.556	1.837	-1.8842	down	2.88E-15
CHTF8	0.194	0.153	0.062	0.128	0.28	0.698	1.638	0.728	0.853	0.352	2.378377	up	2.27E-17
NQO1	98.194	109.419	133.846	137.243	232.833	55.18	86.604	65.383	60.084	69.725	-1.07828	down	5.69E-06
PMFBP1	0.335	0.328	0.419	0.384	0.377	1.378	2.957	4.351	2.87	1.764	2.850099	up	6.12E-42
LOC12111	0.211	0.11	0.134	0.277	0.477	2.951	7.847	13.235	6.236	4.921	4.85753	up	1.63E-74
LOC12110	0.174	0.228	15.743	0.613	18.161	0.262	89.295	89.932	67.971	44.079	3.061423	up	3.43E-50
NT5DC2	16.51	16.917	14.552	32.542	49.533	8.805	9.071	6.387	8.418	8.526	-1.65803	down	3.21E-13
SMIM4	37.41	36.755	18.075	56.563	44.851	14.588	20.317	12.156	15.68	18.722	-1.24921	down	2.96E-08
ITIH3	533.19	482.107	639.242	820.104	691.524	311.056	383.684	365.654	279.084	195.137	-1.04486	down	1.01E-05
MUSTN1	1.16	0.901	1.501	1.907	1.043	0.763	0.745	0.736	0.899	0.066	-1.01984	down	0.001174
LOC12110	30.207	27.185	44.322	20.077	39.055	13.857	15.642	14.462	19.764	14.923	-1.03215	down	3.16E-05
LOC69326	47.834	42.313	73.317	31.811	59.283	23.196	23.445	21.02	27.792	22.609	-1.10842	down	6.45E-06
CACNA2D	0.995	1.185	1.176	1.043	1.456	0.395	0.259	0.377	0.382	0.463	-1.6394	down	8.36E-12
MANF	17.274	19.109	30.432	24.052	29.633	115.491	64.816	70.081	45.496	28.357	1.427996	up	2.82E-14

LOC1211C	2.474	2.67	6.941	1.457	0.379	0.674	0.759	9.137	7.925	11.055	1.085621	up	6.17E-10
LOC1211C	0.236	0.288	1.114	0.723	0.129	0.224	0.065	0.356	0.18	0.412	-1.00637	down	0.000741
GMPPB	15.331	18.192	17.541	15.616	16.166	38.744	40.735	34.567	28.951	24.748	1.017722	up	3.05E-08
MST1R	0.552	0.61	1.135	0.754	0.866	1.011	3.948	2.765	2.568	4.878	1.952035	up	2.53E-22
USP4	34.851	31.939	33.217	25.706	30.698	9.329	12.208	11.523	21.015	12.002	-1.24306	down	1.25E-07
ALAS1	50.567	46.966	63.98	31.99	55.316	24.485	26.663	19.982	30.456	19.84	-1.03499	down	2.41E-05
SEMA3F	1.137	1.099	1.993	1.035	2.229	0.661	0.629	0.806	0.718	0.649	-1.1124	down	2.61E-05
OASL	7.621	9.268	20.363	15.717	5.168	17.207	30.322	28.916	13.464	60.368	1.37002	up	6.04E-13
OGN	0.744	0.5	1.068	0.904	0.452	0.785	1.701	1.586	1.838	1.743	1.060009	up	2.25E-08
ATP2B2	0.413	0.45	0.052	0.076	0.057	0.023	0.387	1.272	0.408	0.365	1.224153	up	3.27E-09
SUSD3	2.994	2.254	1.889	3.366	0.725	2.273	1.422	0.441	0.566	0.715	-1.05085	down	1.84E-05
DNASE1L3	5.72	5.065	11.301	8.801	1.546	9.159	22.055	15.808	12.715	18.475	1.269797	up	2.61E-11
ABHD6	87.008	72.637	40.937	70.693	36.2	11.572	20.032	23.647	20.601	43.875	-1.36068	down	8.54E-10
IP6K2	70.166	60.534	91.078	45.614	15.293	21.757	34.478	21.473	20.521	27.199	-1.1723	down	6.30E-07
LOC77093	2.901	2.674	1.32	3.582	3.39	1.097	1.395	1.082	1.567	1.124	-1.14564	down	1.01E-06
PRRT3	2.035	2.402	2.542	2.585	2.979	0.891	1.947	0.931	1.16	1.195	-1.03373	down	1.69E-05
P4HTM	4.254	4.248	4.653	5.244	4.76	1.927	2.485	2.621	1.836	2.632	-1.0095	down	3.17E-05
GPR27	0.309	0.392	0.087	0.463	0.149	3.928	0.112	0.653	0.381	0.279	1.931124	up	6.82E-18
PROK2	0.259	0.207	0.046	0.118	0	6.925	0.768	0.516	0.219	0.104	3.748901	up	4.18E-44
BHLHE40	14.579	15.981	27.992	29.928	54.212	6.159	8.378	11.624	9.42	12.963	-1.55544	down	2.33E-11
GHRL	0.524	0.374	1.213	0.782	0.831	0.206	0.44	0.26	0.545	0.258	-1.12142	down	0.000693
HBEGF	30.34	31.798	43.511	22.292	20.809	7.296	5.455	11.592	8.778	14.27	-1.6501	down	1.62E-12
TENM2	2.348	2.093	5.216	7.734	11.746	0.109	0.056	0.099	0.045	0.075	-6.22719	down	#######
HMMR	0.161	0.243	0.444	0.214	0.458	1.159	0.739	1.016	1.49	0.774	1.764978	up	1.77E-17
PTTG2	0.163	0.266	0.486	0.201	0.251	2.216	1.003	1.746	2.56	1.324	2.690049	up	2.37E-27
AFAP1L1	0.936	1.69	2.557	2.542	3.614	1.369	1.431	0.929	0.771	1.067	-1.02566	down	3.58E-05
DUSP1	33.482	31.109	90.424	20.028	34.609	13.467	20.015	13.388	15.062	17.478	-1.40055	down	9.03E-09
STC2	18.675	18.883	15.306	31.688	29.823	0.926	11.799	3.66	17.893	17.8	-1.13495	down	7.47E-07
MXD3	0.861	1.164	1.964	0.786	1.454	0.331	0.432	0.313	0.365	0.64	-1.57942	down	1.06E-09
LOC10175	0.028	0.186	0.497	0.21	0.437	1.317	1.049	0.952	1.894	1.411	2.281788	up	1.37E-22
LOC41626	28.651	28.968	31.396	19.497	34.431	11.296	11.658	11.096	14.937	19.875	-1.05361	down	1.09E-05
GRIA1	0.327	0.317	0.163	1.681	1.982	0.831	0.343	0.059	0.234	0.444	-1.22379	down	3.12E-07
SPARC	8.177	10.57	14.218	12.368	22.153	10.785	49.487	21.456	34.81	28.043	1.099161	up	2.95E-09
TRPC7	0.315	0.344	1.872	1.381	0.405	1.268	0.162	0.01	0	0.095	-1.48877	down	3.53E-08
LOC10174	4.412	4.148	7.51	4.91	7.225	4.408	2.142	0.813	0.745	1.178	-1.6023	down	1.56E-11
LECT2	0.954	1.249	2.462	8.815	0.51	0.047	0.353	1.013	0.837	0.242	-2.48664	down	8.94E-23
SLC22A5	40.76	42.726	34.423	30.492	37.282	15.505	15.297	12.524	31.175	16.767	-1.02462	down	1.90E-05
FGF1	1.145	1.332	1.418	1.499	1.285	0.84	1.918	1.44	8.979	2.589	1.238491	up	1.34E-10

EGR1	81.128	64.473	88.761	3.013	5.115	10.844	10.959	6.148	5.482	13.147	-2.38002	down	3.02E-25
BHLHA15	0.159	0.415	0.505	0.326	0.163	5.5	0.652	9.038	1.21	0.789	3.450315	up	2.85E-37
BAIAP2L1	23.712	23.194	33.089	21.851	33.955	14.967	10.597	11.588	14.843	12.608	-1.07176	down	1.19E-05
NPTX2	0.437	0.313	0.673	0.648	1.722	0.096	0.174	0.165	0.107	0.127	-2.49442	down	3.53E-20
GAA2	26.976	27.62	16.32	22.585	20.657	8.11	13.558	9.236	8.149	10.967	-1.19037	down	1.35E-07
CYP3A5	1487.008	1384.543	900.646	1710.507	882.877	352.011	534.782	436.05	433.578	1300.997	-1.05798	down	2.18E-06
RNF216	2.352	2.423	3.154	2.45	2.902	4.206	8.822	7.754	7.968	4.041	1.303613	up	4.12E-12
LOC10174	1.163	1.458	0.216	0.653	0.756	4.486	8.064	8.755	7.798	2.033	2.872941	up	1.17E-38
CACNA1H	0.06	0.042	0.158	0.103	0.147	0.037	0.965	1.408	0.825	1.255	3.125677	up	4.69E-44
SOX8	0.807	0.688	2.121	0.341	1.305	0.121	0.276	0.515	0.21	0.751	-1.48778	down	9.32E-08
MSLN	0.351	0.266	0.629	0.171	0.53	0.177	0.575	0.96	0.618	1.569	1	up	1.45E-07
FAHD1	23.39	22.484	16.26	14.56	17.856	7.891	10.287	8.437	8.369	10.078	-1.06908	down	5.02E-06
PLK1	0.239	0.073	0.279	0.157	0.425	1.555	1.405	1.328	1.592	0.733	2.490056	up	1.32E-28
RMI2	16.163	14.381	11.234	5.409	5.134	3.491	2.828	2.805	2.624	3.281	-1.7993	down	2.44E-15
RHBDF1	1.817	1.82	3.071	2.347	1.169	11.037	4.286	3.673	3.505	1.919	1.255693	up	9.84E-12
HBAD	93.174	97.936	309.947	23.94	123.835	9.458	28.474	18.472	7.097	36.869	-2.69246	down	2.06E-30
HBA1	365.186	383.036	1151.922	127.271	387.325	42.809	105.932	69.806	36.804	174.436	-2.49016	down	1.41E-26
PDIA2	0.22	0.352	0.575	0.382	0.314	12.036	0.428	0.697	1.144	0.166	2.969626	up	2.13E-42
LOC41665	7.203	7.536	6.535	1.798	2.021	0.047	0.265	0.356	0.268	0.295	-4.34382	down	9.83E-69
C16orf96	1.74	1.911	1.353	1.788	1.298	0.641	0.809	0.534	1.245	0.618	-1.07142	down	1.79E-05
ZKSCAN7	3.465	2.733	3.456	1.473	1.73	1.196	1.442	1.542	0.772	0.849	-1.1475	down	2.39E-06
NME4	747.074	821.07	1052.988	918.346	1253.88	124.114	351.496	330.435	544.087	523.613	-1.35511	down	5.86E-09
DECR2	121.269	119.623	149.746	120.838	144.37	39.916	57.149	52.625	60.434	77.557	-1.18887	down	4.07E-07
MCRIP2	62.663	56.778	49.221	43.999	43.835	23.501	21.334	26.005	16.959	26.534	-1.16566	down	5.32E-07
LOC12110	1.23	1.319	0.49	1.095	0.035	0.952	0.105	0.187	0.033	0	-1.70303	down	3.78E-09
LOC12110	1.268	1.333	0.721	1.389	0.977	0.206	0.186	0.294	0.058	0.164	-2.6405	down	3.85E-25
SDF2L1	19.198	16.741	25.485	18.423	18.859	82.33	41.491	65.627	30.691	34.356	1.366383	up	2.90E-13
CDC45	0.14	0.165	0.367	0.161	0.287	0.684	1.164	1.265	2.32	0.556	2.413594	up	1.50E-27
CLDN5	8.478	13.541	14.928	11.196	19.093	2.175	5.102	4.175	5.102	8.651	-1.41535	down	1.24E-09
COMT	25.212	23.734	31.14	30.333	38.819	17.529	21.456	13.201	13.57	8.82	-1.00078	down	3.17E-05
RIMBP2	2.897	2.804	1.685	3.33	2.154	3.116	7.506	6.576	5.531	5.343	1.124816	up	4.44E-09
AACS	7.247	6.549	6.818	7.214	4.705	10.979	31.045	37.079	9.683	17.163	1.703241	up	7.71E-18
RAD9B	1.605	1.591	1.683	1.202	0.539	0.167	0.114	0.248	0.349	0.316	-2.46609	down	2.24E-22
PPTC7	26.451	23.969	28.714	22.315	24.565	7.62	7.749	8.127	12.688	8.204	-1.50524	down	8.41E-11
UNG	10.68	12.674	7.614	8.913	9.779	4.203	3.956	2.261	4.461	4.043	-1.39163	down	2.12E-09
ACACB	1.373	1.365	0.944	2.486	1.391	0.02	0.072	0.044	0.094	0.118	-4.42141	down	4.90E-68
FAM222A	1.655	1.592	0.582	0.878	1.87	0.098	1.693	0.307	0.355	0.53	-1.13935	down	4.10E-07
CRYBB3	0.418	0.41	1.289	0.129	0.429	0	0	0.041	0.05	0.236	-3.01298	down	9.36E-13

LOC10175	2.334	2.719	2.561	1.871	1.901	1.04	1.433	0.755	0.869	1.184	-1.10765	down	9.45E-06
IGLL1	9.491	6.984	64.494	48.14	2.771	23.356	4.013	2.992	2.776	3.403	-1.85153	down	9.01E-15
VPS29L	2.106	2.448	1.502	1.318	6.329	1.032	0.711	1.104	2.069	0.796	-1.26169	down	9.76E-07
CHCHD10	310.225	289.108	416.78	254.481	521.347	153.864	178.818	165.575	172.418	186.645	-1.06361	down	1.19E-05
MMP11	1.993	2.144	0.992	1.228	1.358	0.036	0.492	0.224	0.64	0.312	-2.17545	down	1.19E-18
DERL3	50.473	48.736	47.267	47.022	39.884	165.766	66.549	113.064	67.351	56.493	1.007565	up	2.80E-08
SLC2A11	1.59	1.464	1.87	1.186	2.764	1.28	0.478	0.945	0.327	0.885	-1.17954	down	2.57E-06
SLC2A11L	3.606	3.592	3.219	2.567	6.134	1.166	0.708	0.896	0.679	0.332	-2.33656	down	5.03E-22
LOC76955	17.466	12.831	48.828	14.684	24.095	5.87	3.401	6.789	4.265	3.762	-2.29105	down	5.16E-22
LOC10175	10.07	6.966	32.213	15.319	9.528	9.842	3.453	6.964	3.662	5.678	-1.3237	down	9.44E-08
GSTT1L	3.5	3.63	9.075	10.313	21.286	3.369	5.067	2.298	2.972	2.567	-1.55436	down	4.11E-11
TBX6	2.077	2.257	1.313	2.702	4.297	1.51	1.4	1.153	0.254	0.31	-1.44954	down	4.09E-10
LOC41695	1.175	1.185	0.618	1.032	0.658	0.633	3.972	3.453	2.386	6.025	1.81777	up	5.75E-19
PISD	20.194	19.222	12.543	12.178	6.629	8.404	58.22	53.228	27.001	70.537	1.619089	up	4.85E-16
ACADS	35.448	36.42	50.216	40.071	74.657	26.302	21.848	22.73	24.491	22.915	-1.00143	down	4.62E-05
LOC10085	4.583	5.225	3.024	4.197	7.868	0.77	1.48	1.175	0.937	1.362	-2.11991	down	2.00E-17
PRODH	0.862	0.852	4.639	2.415	3.652	17.073	3.4	2.829	5.656	5.65	1.478068	up	1.18E-15
SLC35E4	0.209	0.239	0.312	0.204	0.174	0.153	5.497	2.912	3.036	3.622	3.735545	up	2.83E-56
TCN2	106.731	126.53	35.771	38.128	31.915	93.94	305.896	341.834	267.144	280.556	1.926974	up	2.96E-21
HORMAD1	1.423	1.088	1.587	0.976	1.965	0.225	0.291	0.398	0.852	0.465	-1.65547	down	3.10E-12
LOC41701	111.681	109.563	124.638	98.657	150.498	15.066	27.996	24.543	45.341	39.46	-1.96502	down	6.88E-18
LOC10705	3.791	2.89	5.611	4.597	6.688	2.328	1.775	1.718	2.697	2.322	-1.12066	down	5.87E-06
LOC12110	0.534	0.73	2.048	1.755	7.974	0.451	0.299	0	1.249	1.009	-2.11433	down	5.97E-15
LOC10704	0.14	0.252	0.335	0.202	0.216	0.022	0.216	0.027	1.17	1.014	1.093501	up	2.60E-05
MHCY2	0.315	0.289	0.251	0.078	0.907	1.503	3.582	0.541	2.376	0.1	2.135547	up	6.11E-23
MHCY14	1.109	0.968	2.202	1.215	0.577	0.027	0.061	0.819	0.042	0.094	-2.53548	down	2.23E-22
LOC12110	1.131	0.841	0.951	0.317	0.172	0.127	0.132	0.481	0.049	0.174	-1.81965	down	3.76E-12
LOC12110	0.174	0.13	0.257	0.245	0.307	2.525	2.658	0.291	8.467	0	3.640859	up	5.67E-50
LENG9L7	4.913	5.016	7.793	9.996	2.767	0.135	0	4.7	0	0.072	-2.63395	down	2.13E-28
MHCY7	1.2	0.974	2.181	1.171	0.861	0.054	0.103	1.034	0.107	0.034	-2.25727	down	6.67E-19
ZNFY1	1.434	1.495	1.681	1.217	0.89	0.166	0.178	0.086	0.27	0.157	-2.96313	down	4.74E-30
YLEC17	0.083	0.109	0.066	0.341	0	0.462	2.473	1.748	0.87	0.375	3.296141	up	1.07E-19
LOC12110	1.892	1.345	2.562	4.688	0.933	3.504	7.684	10.554	3.381	4.418	1.370769	up	2.66E-12
LOC12110	0.996	0.879	4.17	0.918	0.279	0.411	0.512	0.412	0.273	0.232	-1.97376	down	2.51E-14
LOC12110	0.866	0.54	1.248	0.39	0.508	0.281	0.064	0.274	0.334	0.093	-1.7589	down	1.05E-09
LOC12110	1.782	1.403	1.563	0.448	0.336	0	0.075	0.34	0.416	0	-2.72753	down	1.69E-19
LOC12110	10.943	11.057	14.532	7.957	1.516	0.579	0.592	8.132	0.772	1.4	-2.00282	down	1.08E-17
MHCY8	2.228	1.938	2.656	1.732	1.521	0.693	0.495	1.259	1.321	1.154	-1.03271	down	4.40E-05

MHCY32	0.15	0.147	0.268	0.446	8.325	3.326	14.466	0.657	6.177	0.779	1.443747	up	6.02E-14
MOGL	13.688	10.911	1.441	3.719	2.789	2.751	4.65	2.068	1.141	4.151	-1.14051	down	1.62E-07
LOC1211C	2.086	2.181	22.64	26.21	25.371	0.703	19.144	0.664	0.081	0.885	-1.86944	down	4.68E-17
BTN3A3L1	0.297	0.36	0.624	0.731	0.914	0.302	9.274	0.43	0.303	0.153	1.836383	up	9.38E-17
KIFC1	0.701	0.582	4.305	3.332	4.287	6.397	6.962	1.282	3.834	14.898	1.337049	up	1.21E-12
IL4I1	0.662	0.6	1.835	0.561	0.701	32.674	2.851	2.199	1.364	4.574	3.32282	up	2.15E-52
TRIM7.1	0.057	0.037	2.341	0.117	0.059	0.009	1.13	0.022	0.014	0.052	-1.08636	down	0.00028
TRIM27.1	0.821	0.682	1.079	0.278	0.455	0.129	0.281	0.183	0.137	0.401	-1.54722	down	3.45E-09
BF2	180.458	117.958	399.975	278.384	216.187	409.014	1140.776	353.517	365.972	764.352	1.346494	up	8.45E-13
LOC1211C	1.894	1.973	0.277	0.921	0	0.024	0.358	0.121	0.184	0.07	-2.73412	down	2.92E-20
LOC10704	0.733	0.625	0.711	0.838	1.08	0.335	0.36	0.025	0.365	0.317	-1.50449	down	1.61E-07
FCN2	1.003	1.114	0.965	0.611	1.238	0.491	0.356	0.428	0.489	0.294	-1.2586	down	3.45E-07
MAN1B1	82.815	78.378	81.445	67.277	71.142	27.441	32.552	36.207	40.721	38.327	-1.12059	down	1.98E-06
EXFABP	4.471	7.057	6.987	22.369	8.956	4031.649	22.516	25.866	19.829	22.089	6.369736	up	#######
LCN15	0.332	0.528	0	1.092	0.049	31.068	0.439	0.889	0.453	3.006	4.159982	up	2.59E-60
PTGDS	154.498	170.177	127.819	223.199	34.349	747.95	141.192	288.594	134.16	499.102	1.350803	up	3.40E-12
BSPRY	5.053	5.265	2.832	3.691	4.582	2.414	1.443	1.899	1.309	2.962	-1.09489	down	5.22E-06
STPG3	2.738	3.512	3.672	2.191	2.479	0.245	1.814	0.59	0.378	0.815	-1.92387	down	3.99E-15
NSMF	0.468	0.31	1.023	0.218	1.076	4.673	3.246	4.108	5.509	5.792	2.912032	up	1.11E-44
GSL	1.128	1.641	1.741	1.002	1.773	3.49	2.27	2.065	5.355	2.06	1.064345	up	1.21E-08
BRINP1	0.307	0.211	2.795	0.579	0.614	0.256	0.346	0.12	0.117	0.402	-1.85614	down	9.91E-12
ORM1	821.733	916.944	1578.27	3646.15	3222.935	18890.05	1475.61	1730.331	1038.155	834.64	1.234565	up	2.24E-11
LOC10705	75.951	77.659	59.169	58.121	65.695	19.841	18.458	18.767	42.104	26.903	-1.41672	down	7.18E-10
SLC25A25	43.558	44.396	31.78	31.397	45.136	10.901	7.399	8.751	28.501	12.658	-1.52469	down	3.83E-11
ST6GALNA	1.965	2.037	2.057	1.959	2.018	0.902	1.089	0.896	0.611	1.138	-1.1134	down	7.21E-06
FPGS	41.104	41.489	85.376	36.875	85.68	10.616	14.771	8.41	14.107	12.9	-2.25633	down	1.86E-22
DNM1	9.543	8.703	6.504	6.356	9.262	1.613	5.343	4.376	3.858	4.333	-1.04785	down	6.76E-06
LOC1211C	0.464	0.938	0.302	1.56	1.82	0.104	1.517	8.823	1.609	1.259	1.387815	up	4.06E-11
ASS1	5.658	7.386	12.713	12.465	14.161	36.245	25.061	14.242	11.729	19.771	1.031017	up	1.41E-08
NTNG2	0.19	0.177	0.662	0.494	1.06	0.006	0.299	0.117	0.067	0.018	-2.33762	down	2.57E-18
ADAMTSL	2.087	1.977	3.885	1.935	2.054	13.674	5.391	3.914	2.769	3.364	1.285696	up	2.68E-12
FAM69B	0.345	0.319	0.485	0.751	0.563	0.101	2.878	2.439	3.561	0.624	1.960894	up	1.08E-19
AGPAT2	9.837	11.148	19.814	12.831	6.98	14.738	126.841	137.552	101.845	88.457	2.953188	up	1.07E-44
EGFL7	3.296	4.035	7.742	4.319	4.197	3.95	11.1	18.695	12.068	11.389	1.277772	up	4.44E-12
LOC41711	1.094	1.081	2.958	1.086	1.088	3.005	2.353	3.948	2.206	4.791	1.157242	up	1.76E-10
HSPA5	203.11	183.635	232.322	172.83	247.172	1365.078	481.353	1097.783	311.835	279.139	1.766491	up	6.23E-20
MVB12B	4.325	3.783	5.199	4.809	4.784	7.999	13.368	12.077	8.593	11.318	1.220096	up	7.69E-11
HS3ST3B1	76.526	69.615	73.481	95.337	88.844	45.946	33.47	27.712	51.616	35.883	-1.05292	down	8.95E-06

HS3ST3A1	5.418	5.768	2.626	4.097	4.397	2.626	1.185	0.26	2.015	0.427	-1.77525	down	6.96E-15
FN3K	3.913	3.747	7.322	10.982	14.227	1.812	3.411	2.45	4.784	3.328	-1.34804	down	1.00E-08
TEN1	4.687	5.166	5.237	10.568	7.423	1.024	2.914	2.136	3.719	1.371	-1.56672	down	1.05E-11
ACOX1	304.321	272.828	426.332	464.053	441.56	87.703	101.683	99.12	141.198	109.517	-1.82393	down	1.45E-15
FBF1	14.52	14.705	15.185	12.617	14.558	4.202	4.608	3.061	9.446	4.809	-1.454	down	4.01E-10
MRPL38	52.316	48.259	56.053	35.782	44.87	16.934	19.978	19.145	26.452	20.672	-1.20137	down	3.90E-07
TRIM65	5.692	5.816	6.734	4.437	5.341	1.588	2.732	1.993	4.814	2.553	-1.03412	down	2.41E-05
UNC13D	4.85	4.296	6.665	3.729	2.569	1.278	2.01	1.861	1.886	1.434	-1.38384	down	4.61E-09
SMIM5	0.681	0.722	0.237	1.602	0.909	1.895	2.373	1.662	2.225	2.273	1.327887	up	4.12E-11
CCDC57	1.045	0.92	0.555	0.711	0.769	0.192	0.252	0.373	0.652	0.444	-1.0622	down	3.03E-05
FASN	110.775	119.381	100.058	62.645	146.049	115.643	220.869	388.518	327.299	412.559	1.442671	up	3.35E-14
PCTP	2.597	2.63	1.359	1.574	1.48	0.411	0.565	0.488	0.85	1.029	-1.52648	down	1.64E-10
KPNA2	1.883	1.577	2.2	1.866	1.482	9.533	4.829	6.744	7.586	4.966	1.901084	up	1.46E-21
CACNG4	0	0	0.353	0.364	0	1.471	0.166	0.346	0.653	0.182	1.967158	up	1.64E-12
RGS9	1.299	1.369	1.497	0.838	0.902	0.415	0.578	0.461	0.599	0.457	-1.2326	down	2.73E-07
ABCA8	66.088	65.042	30.608	67.383	91.882	12.307	48.61	25.308	36.953	19.948	-1.16527	down	2.63E-07
CDC42EP4	2.502	2.086	2.77	1.721	2.228	1.177	1.126	1.098	0.573	1.194	-1.12878	down	3.52E-06
CCDC40	0.266	0.269	0.549	0.487	0.286	1.228	0.97	0.727	0.565	0.339	1.041997	up	1.04E-07
CBX2	0.516	0.535	0.24	0.106	0.309	1.079	1.879	1.914	1.416	1.09	2.109367	up	1.41E-20
RBFOX3	0.214	0.159	0.327	0.123	0.086	0.346	1.83	1.564	0.854	1.535	2.746562	up	1.73E-34
ENPP7	0.952	1.027	0.061	0.529	0.026	0.319	0.873	2.31	1.767	1.094	1.292329	up	8.82E-09
TIMP2	102.314	120.649	120.318	83.634	148.091	85.035	464.424	418.167	387.519	403.232	1.612587	up	8.88E-17
SOCS3	3.048	2.755	5.606	4.452	1.599	30.413	13.23	39.656	7.153	5.593	2.459319	up	2.05E-33
TK1	1.116	0.548	2.311	0.917	3.497	3.663	11.472	6.316	10.06	5.502	2.140797	up	1.92E-24
P4HB	266.978	272.602	344.816	341.594	418.659	899.482	601.215	694.082	674.245	502.014	1.035411	up	9.40E-09
GRIN2C	1.068	1.271	0.191	0.296	0.839	0.332	0.012	0.098	0.038	0.196	-2.43005	down	6.09E-24
HN1	39.239	38.608	28.761	35.62	42.532	13.314	11.213	12.536	26.901	13.922	-1.24616	down	8.84E-08
SLC16A5	100.899	98.569	67.509	91.142	104.637	14.656	11.377	13.93	57.072	21.459	-1.96539	down	6.38E-18
GATSL2	2.567	2.397	5.105	1.018	4.584	1.006	0.862	0.815	0.529	1.283	-1.80056	down	9.98E-14
SPNS2	1.863	2.73	2.567	3.154	4.708	0.727	1.685	1.498	1.883	1.668	-1.00915	down	4.28E-05
MIS12	0.314	0.78	0.4	0.774	1.29	4.003	5.807	2.597	4.371	1.476	2.357443	up	1.72E-23
MMP28	0.074	0.039	0.71	0.659	0.183	1.057	1.862	0.626	0.666	1.947	1.883785	up	1.65E-18
LOC12110	1.481	1.211	2.801	0.355	3.169	13.6	1.522	2.986	1.589	3.516	1.363725	up	1.93E-12
SERPINF1	74.377	64.558	89.8	202.538	74.576	134.519	24.592	24.752	16.786	10.565	-1.25998	down	4.86E-08
SRR	5.107	4.768	4.455	3.419	3.831	1.652	1.946	1.232	1.505	0.701	-1.61618	down	1.33E-11
TMEM199	14.771	17.083	13.655	32.582	31.142	118.11	23.122	44.574	17.401	22.994	1.050162	up	1.23E-08
SEBOX	6.22	6.517	4.5	18.195	17.428	70.497	11.693	26.939	8.443	10.819	1.280215	up	1.45E-11
SARM1	4.158	4.11	1.827	8.341	3.139	37.355	3.161	5.849	3.469	2.61	1.28122	up	3.27E-11

SLC13A2	0.047	0.133	0.025	0.064	0.144	0.859	1.955	4.83	1.204	0.141	4.427388	up	1.59E-62
DHRS13	17.405	23.36	14.841	18.293	13.772	7.838	10.095	9.065	9.154	6.89	-1.02627	down	1.21E-05
PIPOX	25.067	27.286	25.785	23.869	35.325	5.535	14.045	13.036	15.931	10.58	-1.21571	down	2.18E-07
MED13	24.129	20.91	40.145	20.433	24.737	7.646	11.353	8.628	11.093	14.276	-1.2984	down	4.36E-08
CA4	14.605	12.925	8.114	0.151	2.646	0	3.68	0.055	0.067	0.102	-3.29796	down	3.03E-46
ACACA	39.426	40.517	32.023	29.127	40.439	52.389	70.609	88.637	73.225	98.434	1.078207	up	5.90E-09
EVI2A	0.625	0.324	0.788	0.835	0.294	2.144	1.258	1.484	0.621	1.013	1.184425	up	9.46E-09
CLUH	25.099	24.566	25.932	21.027	30.204	8.435	13	11.094	11.129	13.12	-1.1594	down	7.47E-07
FAM64A	0.423	0.332	0.789	0.635	0.596	0.98	1.154	0.999	1.163	1.344	1.021889	up	7.72E-08
FBXO39	0.27	0.238	0.364	0.239	0.256	0.721	0.865	0.73	1.318	0.884	1.721	up	2.42E-16
TEKT1	0.191	0.104	0.304	0.209	0.261	1.005	0.392	0.447	0.637	0.575	1.511009	up	5.48E-10
ASIP	0.562	0.735	1.598	0.198	0.165	0	0.248	0.627	0	0.073	-1.77565	down	1.12E-05
ACSS2	10.983	11.935	6.329	9.149	7.517	71.357	12.324	10.956	6.904	6.212	1.230663	up	7.70E-11
FAM83D	0.311	0.18	0.391	0.246	0.283	0.43	0.652	0.588	1.107	0.39	1.163572	up	7.44E-09
RPN2	10.605	11.005	15.321	11.706	19.106	43.082	26.12	32.041	22.118	21.487	1.096339	up	1.30E-09
RBPJL	0.057	0.025	0.045	0.046	0.039	18.97	0.716	0.603	0.269	0.341	6.58994	up	#######
MATN4	0.029	0.009	0.011	0	0.015	2.724	0.044	0.056	0.027	0.013	5.377808	up	1.62E-55
PI3	9.954	13.416	15.077	52.646	13.145	7324.136	165.204	85.62	61.737	100.505	6.21379	up	#######
TTPAL	2.052	2.341	4.009	2.487	3.081	4.11	6.805	7.577	4.517	5.735	1.040663	up	8.62E-09
GDAP1L1	0.38	0.327	1.069	0.882	0.257	0.074	0.095	0.072	0.062	0.151	-2.6694	down	8.24E-24
EPB42	0.861	1.048	2.935	0.467	0.938	0.149	0.266	0.269	0.181	0.498	-2.19271	down	5.04E-17
GATA5	9.765	8.934	4.151	9.352	21.945	6.193	2.509	1.361	1.786	1.428	-2.02754	down	8.85E-19
TNFRSF6B	0.199	0.104	0.175	0.049	0.02	3.619	0.246	0.078	0.114	0.108	2.917307	up	7.69E-31
SDCBP2	1.478	1.624	2.398	1.742	2.292	0.276	1.175	0.413	1.25	1.211	-1.13947	down	9.34E-06
TPX2	0.395	0.488	0.943	0.663	0.899	2.039	1.772	1.968	2.708	1.326	1.532868	up	2.24E-15
LOC77197	0.556	0.685	0.352	0.43	0.017	2.157	0.925	0.792	0.78	0.932	1.451006	up	1.06E-11
LOC10085	0.582	0.533	0.463	0.064	0.199	2.88	0.797	0.393	0.222	0.771	1.457014	up	2.40E-10
WFDC2	5.532	6.109	0.358	1.952	0	9.037	21.744	22.056	19.761	10.839	2.579888	up	2.20E-33
WFDC8	75.251	79.538	7.492	29.801	0.154	151.065	275.625	228.368	181.828	179.676	2.402717	up	8.40E-30
SPINT4	2.528	2.408	0.318	0.474	0	1.153	8.843	5.469	5.499	8.062	2.340232	up	4.68E-25
LOC77199	0.826	0.42	0.122	0.151	0.157	0.095	1.383	2.268	0.554	0.719	1.579517	up	4.04E-11
UBE2U	0.241	0.158	0.346	0.516	0.843	4.305	4.119	4.748	6.079	3.624	3.439456	up	1.13E-44
C20orf85	1.036	0.414	0.939	0.544	0.296	0.214	0.266	0.427	0.329	0.208	-1.15826	down	0.000142
PCK1	3.422	4.317	65.761	5.388	29.476	0.231	0.133	0.808	24.749	3.303	-1.89048	down	2.82E-13
RBM38	1.867	1.777	4.031	1.699	2.14	1.032	0.619	0.446	1.575	1.661	-1.10964	down	1.81E-05
UTS2	1.114	0.865	5.154	1.43	4.218	0.345	1.574	0.815	1.195	2.329	-1.02964	down	0.000513
TNFRSF25	4.731	4.345	11.095	4.798	5.462	2.808	3.814	2.541	2.422	3.396	-1.02216	down	5.99E-05
ESPN	0.409	0.47	0.315	0.23	0.162	0.414	4.525	1.491	3.254	4.865	3.19341	up	3.26E-46

ACOT7	0.806	0.538	3.143	1.19	1.926	0.988	0.794	0.513	0.769	0.58	-1.06002	down	0.000139
MIB2	37.939	37.536	44.728	17.633	58.931	11.781	12.553	16.422	30.155	15.354	-1.18959	down	1.03E-06
LOC41940	8.82	8.759	5.918	5.218	47.946	1.448	1.056	1.032	3.085	1.235	-3.2858	down	5.28E-44
LOC77106	34.474	35.519	33.965	35.915	49.009	2.67	7.327	8.126	6.883	7.3	-2.54743	down	3.94E-29
SLC2A5	6.868	7.38	16.248	16.837	31.737	3.031	11.197	12.768	5.291	7.239	-1.00024	down	4.72E-05
CENPS	0.55	0.3	0.566	0.603	0.518	0.709	1.46	1.145	1.377	0.684	1.081642	up	1.65E-07
SLC25A34	9.25	9.784	18.042	14.779	13.308	4.33	6.094	5.357	6.147	7.841	-1.13011	down	3.09E-06
RNF186	11.188	11.918	0.265	5.554	0.043	0.438	22.974	20.675	113.24	73.249	2.992493	up	6.04E-43
CAMK2N1	11.466	14.312	4.295	4.522	3.703	2.131	21.618	21.112	10.056	23.632	1.036227	up	1.29E-07
CELA2A	0.203	0.266	0.485	0.467	0.334	0.503	1.253	2.664	1.473	1.249	2.021762	up	4.90E-17
MTHFR	13.337	12.16	30.133	12.285	4.395	13.484	49.782	48.088	10.883	37.254	1.141153	up	3.42E-10
DRAXIN	0.724	0.636	0.465	0.337	0.311	0.448	5.152	4.798	2.555	1.339	2.528464	up	1.17E-31
ECE1	39.205	38.167	53.402	47.779	35.696	13.492	14.101	14.53	17.473	19.808	-1.43195	down	5.79E-10
LOC11253	13.755	12.357	22.05	16.679	10.052	4.93	5.115	5.357	5.733	7.015	-1.41154	down	1.63E-09
DPYSL2	1.602	1.773	1.878	1.072	1.27	3.176	9.307	3.328	3.68	4.364	1.650523	up	7.14E-17
GNRH1	0.102	0.665	0.971	0	0.104	1.699	0.313	0.555	0.775	0.643	1.111205	up	7.42E-05
DMTN	0.926	0.799	1.2	0.544	0.68	0.258	0.293	0.183	0.34	0.497	-1.39823	down	3.07E-08
LOC10174	25.547	25.293	7.821	17.495	25.19	10.333	4.842	9.437	12.398	5.699	-1.24658	down	9.32E-08
ADAM32L	1.354	1.2	1.01	1.38	1.989	0.276	0.247	0.067	0.294	0.139	-2.75468	down	1.29E-26
IDO2	10.394	12.035	5.352	19.063	27.269	0.073	2.943	0.578	0.659	0.579	-3.93764	down	1.64E-61
ANK1	1.223	1.303	2.635	0.715	1.221	0.183	0.378	0.246	0.304	0.767	-1.91519	down	3.59E-16
NCAPH	0.605	0.485	0.927	0.671	0.544	2.312	1.709	1.723	1.831	1.039	1.412863	up	9.54E-13
ADD2	1.232	1.137	1.521	0.989	0.687	0.65	0.285	0.484	0.346	0.423	-1.34503	down	6.16E-08
SLC20A1	16.074	16.555	17.413	10.516	25.991	3.222	5.556	6.115	8.57	6.41	-1.53452	down	4.72E-11
CASP14	1.235	1.087	1.424	1.278	0.897	0.119	0.131	0.116	0.325	0.077	-2.93852	down	1.25E-26
RETSAT	181.098	181.873	305.999	306.703	245.919	84.674	65.292	68.247	62.392	72.919	-1.78887	down	5.40E-15
FABP3	13.543	18.173	2.598	3.493	0.203	49.203	43.786	170.723	80.983	179.87	3.786495	up	1.73E-62
LOC12110	3.245	3.671	0.699	2.366	1.904	1.48	1.379	0.305	0.675	0.397	-1.48727	down	2.30E-10
NR0B2	61.603	62.574	21.179	38.695	58.573	21.038	22.348	18.611	23.886	25.599	-1.12188	down	8.12E-07
TRNP1	1.298	0.955	1.033	1.155	1.055	0.368	0.278	0.338	0.309	0.489	-1.62215	down	8.87E-08
TCEA3	36.177	36.872	33.198	29.543	14.657	5.162	16.496	13.059	16.103	17.566	-1.13742	down	7.94E-07
LOC10174	0.118	0.173	0.798	1.21	1.15	0.036	0.03	0.046	0.112	0.107	-3.36173	down	6.01E-23
RHCE	8.15	6.385	11.823	9.087	7.332	3.23	2.801	4.78	5.273	3.545	-1.12365	down	5.40E-06
STMN1	5.07	4.523	8.573	3.977	4.092	13.31	46.355	47.791	31.148	15.304	2.552275	up	2.42E-35
PAQR7	24.346	28.129	11.665	9.039	4.872	12.366	104.453	89.408	44.795	55.852	1.975088	up	5.23E-22
DNALI1	1.001	1.403	2.504	1.968	2.937	1.195	0.551	0.837	1.193	0.97	-1.0472	down	0.00016
IFI27L2	1.02	1.144	1.74	27.689	12.533	26	50.497	59.921	74.373	67.726	2.657927	up	8.50E-36
FAM167B	18.468	19.179	15.698	20.949	9.892	5.243	5.502	9.306	4.268	3.509	-1.59687	down	2.53E-12

MYCL	25.236	25.063	17.182	6.684	21.801	0.943	1.546	3.304	4.445	3.191	-2.83671	down	1.52E-34
MFSD2A	110.116	114.057	68.368	54.029	85.642	18.879	40.589	47.533	47.373	48.009	-1.09463	down	2.37E-06
GALE	203.78	179.069	256.203	94.255	87.192	12.265	18.481	20.465	197.224	34.232	-1.53738	down	1.48E-10
HMGCL	1321.124	1321.949	1041.953	914.18	1355.956	46.769	85.798	132.337	1042.758	463.949	-1.74908	down	3.11E-14
GRHL3	0.226	0.247	0.18	0.171	0.184	0.093	1.212	0.758	0.774	0.794	1.843718	up	6.36E-17
IL22RA1	1.048	0.851	0.331	0.475	0.408	3.188	0.984	4.358	5.412	3.269	2.465057	up	6.33E-30
SPA17	5.129	5.559	3.796	3.066	5.694	2.117	1.864	2.426	1.779	1.265	-1.29787	down	1.87E-07
TMEM45L	0.867	1.134	1.972	3.459	2.645	1.977	0.204	0.599	0.779	0.605	-1.27401	down	7.69E-07
NCAPD3	0.182	0.129	0.23	0.318	0.234	0.442	0.968	0.753	1.072	0.378	1.720314	up	2.72E-16
THY1	0.058	0.304	0.046	0.238	0.219	0.275	1.432	0.302	0.48	0.105	1.578869	up	7.19E-10
USP2	2.717	2.8	1.374	3.055	4.658	0.417	0.903	1.451	1.325	1.404	-1.40804	down	1.22E-09
ZBTB32	25.252	27.628	28.602	8.546	14.028	1.443	7.137	4.115	13.083	22.804	-1.09879	down	3.35E-06
ZPR1	128.277	139.38	105.824	94.012	102.903	59.549	50.264	49.348	37.516	70.928	-1.09184	down	2.50E-06
APOA5	226.944	249.026	159.85	201.021	232.501	105.286	81.697	94.581	64.608	119.436	-1.19953	down	1.56E-07
APOC3	964.043	1052.284	2974.691	3131.941	4903.002	1087.703	784.241	686.596	1583.591	1597.886	-1.18226	down	1.06E-06
APOA1	3019.254	3252.166	8200.317	9716.31	8069.09	2375.727	1014.059	1330.2	2668.164	2867.352	-1.65322	down	9.76E-13
TAGLN	2.18	2.11	3.114	3.729	3.373	3.456	10.862	5.406	4.812	7.695	1.151526	up	2.62E-09
FXYD2	7.766	4.487	6.425	4.393	4.04	0.375	1.451	1.338	0.289	0.821	-2.6638	down	4.74E-23
SCN4B	1.577	2.38	1.504	1.283	2.197	0.3	1.669	0.78	0.819	0.72	-1.05926	down	1.12E-05
LOC10174	1.523	1.158	0.721	0.072	0.888	0.295	0.193	0.05	0.213	0.043	-2.45038	down	5.79E-24
HYOU1	31.471	30.812	41.361	31.712	27.66	158.935	87.477	131.883	80.548	63.613	1.680266	up	2.89E-18
BGLAP	2.805	2.936	1.638	3.227	1.825	0.521	0.961	1.459	1.694	0.846	-1.1807	down	2.26E-05
LOC1211C	0	0	0.072	0.374	3.318	6.9	0.117	2.024	0.108	10.409	2.375874	up	3.99E-29
LOC10174	0.02	0.013	0.032	0.083	1.055	1.035	0.455	0.566	0.365	0.783	1.409503	up	8.66E-11
S100A12	2.745	1.976	3.443	9.983	3.384	89.483	4.726	7.07	1.898	2.483	2.294675	up	4.37E-28
S100A6	1.272	2.011	4.726	3.571	0.98	12.137	7.297	3.06	2.324	4.504	1.222819	up	1.87E-09
CKS1B	1.373	1.796	3.682	5.166	4.156	8.275	15.816	9.642	12.265	7.057	1.713591	up	6.21E-17
FDPS	41.825	42.11	7.962	5.635	47.361	88.661	127.04	174.615	59.94	139.955	2.026205	up	1.76E-23
SYT11	0.614	0.468	2.816	2.184	2.532	0.641	1.102	0.594	0.498	0.414	-1.40531	down	1.18E-08
GOLPH3L	1.159	1.435	1.649	0.385	0.582	0.412	0.253	0.5	0.682	0.602	-1.08753	down	9.21E-05
CIART	0.214	0.482	2.613	0.762	2.785	0.486	0.88	0.408	0.386	0.839	-1.19153	down	2.00E-05
LOC1211C	0.115	0.075	0.214	0	0.118	1.377	0.315	0.599	1.39	0.069	2.832938	up	1.76E-22
PCP4L1	2.075	1.357	5.083	3.804	18.36	2.47	2.46	1.868	2.435	2.021	-1.44641	down	4.05E-07
NR1I3	11.506	12.651	21.074	12.57	28.884	5.108	9.748	8.038	7.387	8.68	-1.15365	down	2.05E-06
APOA2	1836.682	2018.685	3427.281	2772.508	4123.305	1004.56	1108.841	1299.042	1176.808	1148.324	-1.30519	down	3.57E-08
TMOD4	0.723	1.269	1.885	0.999	1.756	0.468	0.304	0.231	0.251	0.565	-1.86343	down	1.08E-11
LOC1211C	1.273	1.24	1.603	1.655	1.703	0.77	2.374	0.069	0.169	0.321	-1.0122	down	5.42E-05
LOC1211C	1.707	2.146	2.676	0.723	3.534	0.264	0.685	0.666	0.483	0.748	-1.92029	down	3.40E-14

TULP1	0.396	0.339	3.696	0.217	0.174	0.018	0.058	0.125	0.171	0.623	-2.27113	down	8.40E-18
ELF3	1.802	1.886	5.136	1.871	1.286	17.979	13.704	11.369	1.248	8.955	2.151702	up	3.94E-27
CSRP1	1.668	1.898	3.368	2.702	4.32	7.856	6.012	3.49	12.771	9.772	1.515203	up	2.88E-15
UBE2T	0.274	0.426	0.464	0.084	0.633	1.485	1.338	1.283	1.242	0.496	1.63286	up	5.51E-13
SHISA4	1.226	0.705	0.384	0.366	0.267	0.161	0.153	0.174	0.071	0.168	-2.01227	down	4.25E-10
LOC10705	17.207	16.935	13.494	18.533	16.182	1.084	10.181	6.554	2.504	2.897	-1.8262	down	2.67E-16
SLC26A9	0.066	0.086	0.01	0.003	0	0.004	7.243	3.711	5	2.464	6.760143	up	#######
C1orf186	0.024	0.101	0.122	0.087	0.049	0.095	2.162	0.323	1.476	0.321	3.497461	up	2.39E-42
FAM72A	0.454	0.348	0.628	0.421	1.013	3.065	2.088	2.339	3.329	1.98	2.1582	up	1.61E-24
IKBKE	2.56	2.477	4.719	5.727	8.738	2.084	3.006	2.073	2.718	2.004	-1.02681	down	2.90E-05
CD55	2.146	2.534	5.146	3.223	3.775	24.999	4.157	1.887	2.282	3.257	1.120382	up	5.14E-10
LAMB3	0.296	0.278	0.164	0.519	0.172	0.801	0.364	1.111	1.056	1.345	1.70708	up	3.17E-16
G0S2	17.283	17.63	33.545	71.809	20.964	106.642	19.689	173.475	23.205	105.707	1.410872	up	2.28E-13
HSD11B1E	103.736	96.97	127.297	125.527	179.039	40.433	66.301	38.383	47.79	51.109	-1.37423	down	2.58E-09
GUCA1A	0.963	0.641	1.561	0.964	0.575	0.141	0.306	0.465	0.434	0.522	-1.33007	down	5.05E-07
SLC16A1	80.737	81.864	85.098	65.917	80.697	17.772	25.101	26.923	19.29	19.462	-1.86096	down	2.57E-16
SYCP1	5.077	4.565	9.032	3.579	2.613	0.647	3.151	1.812	2.691	1.798	-1.29954	down	6.67E-08
TSHB	2.692	2.656	5.24	1.229	0.966	0.132	1.273	1.334	0.448	0.812	-1.67528	down	1.22E-10
PACSIN1	3.057	2.979	2.103	5.131	1.149	0.435	6.804	9.167	8.523	12.011	1.356908	up	8.51E-12
TSPO2	1.038	0.781	1.984	0.782	0.498	0.107	0.124	0.297	0.148	0.063	-2.77372	down	5.42E-24
PGC	2.585	2.642	4.652	1.925	4.342	0.633	2.131	1.429	1.541	1.558	-1.14625	down	6.13E-06
CHIA-M31	0.124	0.809	0.066	1.151	1.037	1.416	1.842	1.174	5.953	4.844	2.254762	up	8.82E-25
CHIA	323.094	320.506	459.022	400.362	363.803	573.153	887.116	1118.009	1473.966	1289.322	1.516703	up	1.77E-15
LOC76878	0.737	0.599	0.428	1.424	0.491	8.755	5.138	2.845	15.615	10.232	3.53117	up	3.66E-54
FMOD	0.578	0.697	0.133	0.575	0.156	1.931	0.36	0.641	0.929	0.482	1.020047	up	8.77E-07
LOC10705	1.478	1.284	2.353	0.381	0.126	0.457	0.022	0.187	0	0.157	-2.76466	down	2.13E-28
LOC10704	1.069	1.001	1.257	0.426	0.349	0.381	0.075	0.202	0.085	0.256	-2.03233	down	2.81E-16
VWA5A1	3.4	3.36	1.917	2.377	3.428	1.132	0.811	0.976	1.051	0.771	-1.60997	down	2.89E-12
DBF4B	2.769	2.891	0.507	1.102	0.954	0.83	0.778	0.55	0.672	1.213	-1.02333	down	1.26E-05
C1QL1	0.528	0.653	1.124	0.237	0.858	0.113	0.296	0.375	0.238	0.148	-1.53499	down	3.71E-09
PLCD3	2.246	2.13	1.433	2.418	1.519	0.383	1.021	0.947	0.933	1.175	-1.12721	down	1.09E-06
ACE	0	0.011	0.035	0.244	0.162	2.467	0	0.02	0	0.024	2.460866	up	3.57E-26
SLC4A1	2.92	2.766	7.813	0.831	2.847	0.158	0.482	0.366	0.282	0.781	-3.05041	down	1.51E-36
COL1A1	0.472	0.573	0.814	0.776	1.076	0.872	3.883	1.28	2.449	1.297	1.396969	up	9.34E-13
SLC35B1	36.478	35.208	36.24	31.353	29.908	114.652	57.665	82.354	68.799	52.976	1.153801	up	3.96E-10
C27H17OI	11.499	8.589	25.485	14.803	13.014	6.615	1.412	2.621	6.648	5.568	-1.68229	down	4.01E-12
MEOX1	0	0	0.507	0.22	1.774	0	0.169	0.032	0	0.967	-1.09518	down	0.000367
PHOSPHC	25.754	25.461	25.759	24.48	19.312	8.929	13.909	13.761	11.743	11.375	-1.01594	down	1.74E-05

FBXO47	0.743	0.505	0.093	0.287	0.03	1.154	0.539	0.637	0.959	0.751	1.282352	up	3.64E-09
PLXDC1	2.596	2.764	2.147	4.258	1.85	7.3	3.981	6.956	8.472	5.832	1.256754	up	5.61E-11
CACNB1	3.613	3.616	3.693	3.325	3.512	0.727	2.694	1.476	1.695	2.154	-1.02144	down	1.64E-05
CSF3	0.957	1.356	2.006	1.048	0.786	6.298	2.425	6.795	1.612	1.356	1.586289	up	1.97E-15
RARA	10.973	12.41	15.375	11.101	11.083	10.352	44.894	42.879	24.294	38.088	1.397054	up	3.08E-13
TOP2A	0.342	0.255	0.321	0.388	0.281	2.757	3.367	2.557	5.29	1.509	3.281959	up	9.34E-50
KRT10	0.362	0.403	0.663	0.461	0.465	1.39	1.619	1.456	0.916	1.114	1.462264	up	1.55E-12
P3H4	4.043	4.802	2.653	3.765	2.923	0.902	1.552	1.53	1.274	1.805	-1.36385	down	3.22E-09
G6PC	546.432	558.921	287.335	471.538	539.08	91.668	125.183	104.485	311.113	185.952	-1.55413	down	6.01E-12
VAT1	115.805	120.68	33.306	87.503	40.757	31.815	21.569	25.247	28.996	38.797	-1.44277	down	5.48E-11
RND2	2.62	1.917	1.709	1.848	1.823	1.188	0.424	0.789	1.24	0.896	-1.1273	down	7.72E-06
BRCA1	0.071	0.057	0.165	0.166	0.28	0.541	0.802	0.635	1.254	0.37	2.277425	up	1.17E-26
SLC1A6	0.116	0.21	0.171	0.125	0.211	3.345	0.303	0.098	0.349	0.234	2.370677	up	2.26E-27
MPND	7.891	7.569	12.217	7.376	6.539	2.248	3.053	4.012	3.898	3.071	-1.35276	down	1.45E-08
STAP2	3.545	3.857	4.369	2.185	2.45	0.76	0.63	1.41	2.275	2.065	-1.19966	down	2.21E-06
NMRK2	0.481	0.525	1.118	0.461	0.535	1.39	1.236	1.252	1.377	1.269	1.063006	up	3.31E-07
ACSBG2	14.586	15.643	11.837	8.17	13.21	137.351	274.363	158.679	75.246	138.787	3.627932	up	1.71E-60
MBD3	2.78	2.639	2.807	2.022	2.09	4.819	7.064	6.186	3.046	4.066	1.028929	up	3.21E-08
MEX3D	0.596	0.738	0.777	0.636	0.69	0.879	3.01	2.711	1.028	1.433	1.397219	up	3.57E-12
LRG1	0.422	0.461	0.359	0.44	0.984	1.499	1.071	0.879	1.182	1.35	1.164212	up	2.92E-08
CREB3L3	159.827	156.871	111.394	114.31	126.707	43.594	48.79	38.231	72.37	62.691	-1.33256	down	5.53E-09
MIDN	1.534	1.929	5.572	2.288	1.636	2.507	8.217	5.972	4.601	5.925	1.070527	up	2.53E-09
GAMT	161.068	171.338	127.697	148.859	189.083	6.97	84.744	64.614	78.13	60.483	-1.43603	down	2.29E-10
PCSK4	2.348	2.734	1.991	1.566	1.895	0.321	0.737	0.904	1.949	0.579	-1.22934	down	5.36E-07
ADAMTSL	0.983	1.337	0.423	0.162	2.564	0.037	0.101	0.169	0.112	0.231	-3.06303	down	1.80E-28
LOC10085	1.85	1.341	1.179	1.31	0.877	0.529	0.731	0.822	0.787	0.386	-1.00926	down	0.000149
USHBP1	1.913	1.846	2.234	1.218	1.441	0.77	0.819	0.535	0.502	0.764	-1.35046	down	4.07E-08
LOC4201C	3.702	4.864	6.514	4.686	2.361	5.656	13.685	9.972	5.097	15.769	1.181094	up	6.56E-10
COMP	1.53	1.326	1.717	1.658	0.99	0.541	0.654	0.489	0.891	0.674	-1.15098	down	2.44E-06
LOC10705	6.294	8.386	4.573	4.719	3.256	1.479	1.747	1.864	1.227	5.318	-1.22626	down	1.80E-07
LOC1211C	0.088	0.022	0.123	0	0.193	1.599	2.007	0.043	0	3.094	3.9687	up	3.69E-56
LOC1211C	0.686	0.658	0.66	1.216	0.474	4.916	3.296	2.928	2.201	2.579	2.106086	up	7.92E-24
LPAR2	8.601	8.413	3.964	5.818	5.397	1.661	1.563	2.396	2.453	0.65	-1.88325	down	5.71E-16
ACP5	15.183	16.497	42.327	27.342	39.833	5.877	22.343	12.223	6.197	17.495	-1.13831	down	2.03E-06
CALR	241.781	241.895	342.532	279.632	306.725	1577.87	686.648	921.201	634.798	481.299	1.606625	up	3.99E-17
LOC10704	4.908	3.093	3.336	1.377	2.029	0.222	1.169	1.308	0.856	1.786	-1.46399	down	1.71E-08
LOC10174	8.533	8.596	14.905	13.45	11.949	3.866	5.209	5.381	7.327	5.708	-1.06278	down	1.17E-05
LOC76984	32.556	33.04	8.417	20.533	10.931	8.37	6.264	7.626	11.487	4.593	-1.45989	down	7.02E-11

LOC10175	1.051	0.647	0.043	0.034	0.021	0.401	0	0.112	0.059	0.025	-1.58096	down	4.33E-10
LOC10704	0.009	0.006	0.5	1.416	0.047	0.011	0.038	0.018	0.009	0.012	-4.41431	down	3.30E-54
SLC27A2	142.476	149.833	167.679	140.63	245.202	56.414	97.311	93.623	74.237	88.609	-1.04404	down	1.22E-05
LOC10705	0.091	0.126	0.77	0.477	0.619	1.986	1.391	1.252	0.586	0.689	1.500792	up	2.13E-14
LOC10704	0.147	0.116	8.733	11.362	4.421	12.666	3.272	11.825	10.74	18.529	1.202489	up	5.95E-11
LOC1211C	0.265	0.368	1.277	1.237	1.682	0.215	0.323	0.336	0.284	1.062	-1.11941	down	2.48E-05
LOC10704	0.168	0.184	0.805	0.438	2.796	0.243	0.635	0.548	0.054	0.254	-1.33793	down	4.59E-06
LOC10085	1.032	1.377	7.206	2.828	7.798	0.188	1.852	0.869	0.135	0.384	-2.5601	down	7.25E-26
LOC10705	0.463	0.382	2.116	0.629	1.365	0.411	1.241	0.424	0.154	0.164	-1.04791	down	0.000204
LOC1211C	0.207	0.406	0.823	0.085	1.31	0.149	0.248	0.108	0	0	-2.47529	down	8.47E-13
CHIR-A2	0.295	0.709	0.51	0.769	0.91	0.03	1.113	0.038	0.094	0.134	-1.17739	down	8.51E-05
CHIR-AB1	1.081	1.29	0.253	0.444	0.163	0.335	0.621	0.223	0	0	-1.45054	down	1.58E-07
LOC11253	0.318	0.173	1.027	0.842	0.98	0.361	0.345	0.276	0.084	0.399	-1.18619	down	2.60E-05
LOC10704	0.688	0.922	0.109	1.526	0.318	0.319	0.212	0.054	0	0	-2.59633	down	4.14E-17
LOC11252	0.324	0.593	0.438	0.16	0.632	2.264	2.062	0.96	0	0	1.297862	up	4.79E-09
LOC1211C	0.064	0.063	0.305	1.494	1.179	0.099	0.229	0.05	0	0.029	-2.9162	down	1.18E-18
LOC1211C	0.464	0.203	4.252	0.509	0.636	0.455	0.875	0.181	0.074	0.42	-1.59426	down	1.15E-07
LOC1211C	0.033	0.109	1.94	0.247	1.2	0.269	0.24	0.13	0	0	-2.45617	down	2.45E-13
LOC11253	0.417	0.26	6.651	2.071	3.179	0.553	2.487	0.248	0.151	0.161	-1.8034	down	5.60E-13
LOC11253	1.699	2.017	4.442	3.064	3.784	0.205	1.082	0.129	0.086	0.082	-3.23983	down	6.94E-40
LOC1211C	0.429	0.345	1.971	0.678	1.017	0	0.678	0.052	0	0	-2.59637	down	6.62E-18
LOC43106	0.041	0.134	0.697	0.337	0.38	0.517	1.392	0.438	0.613	0.421	1.08693	up	4.49E-08
LOC11253	0.196	0.23	1.448	0.884	1.346	0.484	1.045	0.214	0	0.018	-1.2183	down	8.79E-06
LOC10085	0.362	0.568	0.979	1.169	0.817	0.224	0.942	0.339	0.207	0.153	-1.06044	down	8.65E-05
LOC10085	0.066	0.022	0	0.055	0	1.316	0.444	0.467	0.032	0	3.935205	up	1.59E-24
LOC10705	1.089	0.786	1.705	1.358	0.231	0.209	0.656	0.029	0	0.034	-2.47133	down	2.54E-17
CHIR-B3	3.536	3.698	0.456	0.184	0.537	1.187	0.998	0.583	0.024	0.045	-1.56623	down	7.09E-11
LOC1211C	2.682	1.949	2.524	0.401	3.255	0.117	3.062	0.402	0.233	0.171	-1.43871	down	3.11E-09
LOC11253	0.05	0.105	0.97	0.55	1.303	0.216	0.072	0.094	0.105	0.325	-1.86836	down	3.22E-12
LOC1211C	0.169	0.331	0.896	0.786	1.156	0.174	0.116	0.088	0	0.051	-2.94538	down	7.14E-14
LOC11253	0.851	0.875	1.513	0.887	1.093	0.48	0.625	0.119	0.029	0	-2.05402	down	6.89E-16
LOC1211C	0.106	0.312	1.056	1.046	0.817	0.164	0.164	0.248	0.152	0.384	-1.58108	down	4.55E-06
LOC11253	2.15	1.872	1.712	1.823	2.73	1.132	1.549	0.634	0.081	0.015	-1.59114	down	2.57E-11
LOC10704	0.203	0.08	1.388	0.333	0.083	0.05	0.292	0.127	0.193	0.037	-1.57124	down	2.61E-05
LOC1211C	0.068	0.103	0.323	0.129	0.763	0.237	0.162	0.404	1.009	1.302	1.164961	up	5.13E-08
LOC10085	0.216	0.336	2.345	3.752	1.72	0.1	0.444	0.232	0.077	0	-3.28687	down	1.07E-32
LOC10704	1.145	0.624	1.317	2.51	0.523	0.138	0.065	0.298	0.243	0.23	-2.64509	down	8.96E-21
LOC10705	0	0	0	0	0	2.011	0	0.512	0.824	1.375	9.884781	up	3.14E-53

FKBP11	17.495	22.133	21.819	27.326	17.85	86.428	65.308	60.533	41.158	37.091	1.446066	up	7.32E-14
METTL7A	142.141	140.895	176.986	159.581	210.518	54.351	83.887	76.37	72.725	56.03	-1.27357	down	5.19E-08
RACGAP1	0.029	0.075	0.275	0.201	0.207	1.433	0.694	0.987	1.645	0.949	2.850676	up	1.86E-34
LOC10704	0.392	0.257	0.769	0.198	0.434	0.663	1.255	0.741	0.834	0.805	1.066204	up	4.50E-08
LOC10704	0.692	0.763	1.247	0.958	0.748	1.804	3.743	1.308	2.119	1.449	1.240631	up	2.43E-09
APOF	37.366	50.766	36.826	63.533	23.793	40.006	214.461	133.536	155.849	175.323	1.760323	up	2.18E-18
TIMELESS	0.35	0.414	0.481	0.312	0.19	0.301	1.24	0.744	0.603	0.81	1.079692	up	7.96E-08
ZBTB39	1.483	1.009	3.411	2.154	2.754	1.094	0.908	0.959	0.757	1.382	-1.08318	down	2.72E-05
TAC3	0.091	0.208	1.231	0.99	1.378	0.056	0.164	0.053	0	0.021	-3.70637	down	1.39E-30
INHBE	4.017	4.779	3.518	12.377	2.036	9.539	5.523	9.243	24.348	15.366	1.260045	up	9.65E-11
KRT7	0.635	0.432	0.738	1.138	0.496	2.186	1.175	0.773	1.599	1.309	1.032924	up	8.23E-08
POU6F1	0.897	0.795	2.112	0.587	0.44	0.211	0.759	0.397	0.318	0.294	-1.2854	down	3.64E-07
LOC10175	1.523	1.574	0.648	0.555	0.88	0.273	0.56	0.23	0.363	0.078	-1.78075	down	4.86E-14
LOC1211C	0.594	1.033	0.032	0	0	0.05	0	0.042	0	0	-4.10053	down	4.96E-32
ADPRHL	4.281	4.138	2.677	2.177	3.602	1.325	0.808	1.387	1.534	0.427	-1.62149	down	5.06E-12
LOC10705	10.202	5.451	99.121	66.896	4.471	34.422	3.047	2.834	2.904	4.106	-1.97597	down	2.04E-16
LOC11253	3.567	3.559	6.025	0.829	1.517	4.349	8.479	10.094	16.252	6.94	1.572907	up	1.95E-16
VKORC1	7.719	7.772	7.827	8.078	7.185	5.338	33.713	10.447	24.154	12.543	1.15961	up	1.89E-09
LOC1211C	0.377	0.123	12.832	10.917	8.412	5.35	0.97	0.501	1.764	1.399	-1.70938	down	1.07E-11
LOC10705	2.671	2.885	17.939	1.035	2.537	3.073	2.124	3.441	1.658	1.436	-1.20574	down	1.81E-05
LOC11253	1.12	1.111	0.863	0.579	1.614	0.727	0.446	0.522	0.482	0.376	-1.0488	down	8.13E-05
OTX5	3.385	3.379	6.24	1.429	2.499	0.384	0.17	0.388	0.573	0.103	-3.38344	down	1.49E-42
LOC10705	5.249	4.591	10.795	2.095	3.419	0.743	0.185	0.726	1.058	0.217	-3.15609	down	3.03E-35
NKPD1	0.904	0.861	2.013	1.825	1.469	1.217	1.714	2.63	2.662	6.603	1.067406	up	7.72E-09
LOC1211C	3.558	4.239	2.535	2.678	1.308	0.951	1.078	1.666	1.393	1.694	-1.07749	down	1.31E-05
LOC42622	85.864	97.813	72.44	292.66	10.808	146.281	70.341	311.879	409.384	377.166	1.232682	up	2.53E-10
LOC1211C	3.144	2.495	2.678	1.228	1.151	0.654	0.569	0.985	0.897	0.629	-1.51702	down	3.30E-10
ALPK2	1.282	0.914	3.265	0.609	0.412	0.199	0.037	0.043	0.104	0.066	-3.83679	down	2.36E-49
SMAD7	1.084	1.035	3.634	4.505	3.217	1.136	1.268	1.512	1.116	1.288	-1.09168	down	8.55E-06
ENHO	11.656	11.328	6.264	7.549	2.636	13.095	24.696	12.427	27.835	31.904	1.479347	up	9.51E-14
ARID3C	2.61	2.312	1.969	1.971	1.86	1.786	3.735	4.649	5.252	6.822	1.052494	up	2.42E-08
LOC10085	2.842	2.447	3.327	2.85	2.704	10.49	2.383	5.446	5.918	8.2	1.194514	up	1.70E-10
RUSC2	0.621	0.736	0.94	1.289	0.973	0.974	9.848	1.791	9.446	8.726	2.754091	up	1.17E-38
NPR2	0.607	0.703	0.907	1.714	2.219	0.85	0.405	0.523	0.426	0.318	-1.28433	down	2.36E-07
AVDL	0.933	0.862	0.83	1.443	1.353	0.102	0.226	0.343	0.157	0.099	-2.54149	down	3.83E-15
AVD	0.679	0.578	0.703	2.345	0.488	293.2	1.675	2.227	2.462	13.269	6.026839	up	#######
CA9	1.104	1.293	0.961	2.131	3.296	0.588	0.528	0.24	0.318	0.557	-1.97494	down	5.04E-15
NPR3	0.084	0.139	0.074	0.152	0.188	0.154	0.273	0.181	0.488	1.157	1.8144	up	2.33E-18

CENPK	0.269	0.07	0.236	0.111	0.111	0.8	1.023	0.399	1.129	0.268	2.175909	up	2.39E-17
BHMT2	524.771	561.713	292.981	788.682	2438.933	74.395	287.13	458.344	306.575	219.151	-1.7756	down	7.36E-15
DMGDH	52.099	51.451	39.368	61.156	111.39	6.442	30.11	29.175	33.476	22.642	-1.37239	down	2.29E-09
S100Z	2.605	2.791	0.713	2.97	4.582	0.232	1.238	1.296	0.559	1.134	-1.61418	down	2.69E-12
HMGCR	31.138	34.359	11.575	7.257	41.515	44.955	70.463	90.009	33.633	87.469	1.375539	up	1.06E-12
ANKRD31	0.761	0.768	0.969	0.828	0.809	1.433	2.082	1.644	1.881	1.624	1.066235	up	1.00E-08
GLDC	69.204	66.091	99.52	67.77	72.787	41.352	369.046	279.53	231.58	275.2	1.672665	up	7.91E-18
PLIN2	135.306	116.516	114.391	25.832	53.914	21.939	28.969	70.65	13.353	77.455	-1.07034	down	5.91E-06
ALDH1A1	66.11	65.019	78.501	84.962	205.63	24.822	53.975	45.208	53.631	37.801	-1.21528	down	2.76E-07
ANXA1	0.209	0.273	0.221	0.647	0.405	2.367	0.667	0.289	0.398	0.105	1.122146	up	1.55E-07
FBP2	3.111	2.678	4.789	5.166	1.373	0.569	0.837	0.831	1.434	0.906	-1.9018	down	1.70E-15
CKS2	0.305	0	0	0.063	0.078	1.133	0.157	0.179	0.291	0.138	2.077076	up	1.65E-07
GADD45G	9.213	10.267	11.004	14.629	6.816	22.378	33.137	39.116	27.773	168.112	2.483891	up	4.22E-32
SHB	3.992	3.684	5.439	2.551	2.615	15.039	33.848	3.373	2.917	27.31	2.173515	up	1.92E-26
MFSD7	1.309	1.008	2.038	1.828	1.356	1.592	5.345	4.031	3.104	3.216	1.196787	up	2.81E-10
LPL	1.707	1.894	5.175	0.471	5.173	0.144	0.207	0.141	0.081	0.058	-4.5034	down	6.03E-65
PSD3	7.349	6.967	7.679	4.194	8.275	1.994	2.011	1.571	0.975	0.859	-2.21678	down	2.37E-22
LMNB1	0.293	0.575	0.616	0.584	0.451	2.085	1.268	0.653	1.157	0.832	1.249251	up	1.38E-09
RHOBTB3	2.66	2.558	3.444	3.373	4.449	2.194	0.767	0.976	0.922	0.9	-1.51636	down	1.28E-10
RFESD	1.142	1.128	2.147	1.617	0.724	1.038	0.449	0.417	0.579	0.659	-1.10369	down	4.03E-05
LOC76841	3.926	3.775	0.9	4.596	3.422	0.442	0.428	0.557	0.397	0.484	-2.84543	down	1.83E-26
TMEM246	5.054	5.373	3.954	4.953	8.482	1.967	1.601	1.81	4.524	3.802	-1.02105	down	2.24E-05
ALDOB	11839.59	10520.93	7994.081	8633.508	7428.55	1220.652	1785.283	2428.288	3279.549	4214.338	-1.84413	down	2.09E-16
KIAA1958	30.304	27.952	9.523	15.084	9.131	3.591	43.107	36.751	60.889	51.368	1.089035	up	1.53E-08
SMC2	0.127	0.124	0.223	0.357	0.39	2.14	2.111	1.427	3.228	1.064	3.024358	up	4.85E-42
MUSK	0.035	0.03	0.009	0.036	0.065	0.021	0.427	0.124	1.145	0.326	3.508147	up	4.84E-49
LPAR1	0.395	0.296	0.131	0.359	0.11	0.759	1.531	0.424	1.531	1.519	2.154256	up	9.86E-23
SPINK4	35.548	33.616	33.127	67.803	24.315	3.652	17.708	13.992	18.505	17.969	-1.43645	down	1.97E-10
SEMA6A	1.654	1.45	1.538	1.04	2.022	0.222	0.892	0.648	0.621	0.542	-1.39564	down	3.83E-09
CDKN2A	0.782	0.648	0.831	0.964	1.152	4.907	1.635	3.012	5.073	5.777	2.219544	up	1.41E-25
CDKN2B	0.209	0.561	0.183	0.567	0.773	1.644	0.494	1.387	2.852	6.204	2.453369	up	6.46E-28
CCDC112	2.162	1.871	1.46	1.92	1.839	0.676	0.673	1.039	0.833	0.731	-1.22614	down	7.63E-07
LOC10705	14.178	14.894	14.025	12.341	21.783	19.137	47.612	29.766	32.528	32.399	1.063902	up	1.05E-08
LOC10705	0.092	0.216	1.78	1.099	1.317	0.227	1.017	0.329	0.017	0.149	-1.37041	down	2.46E-07
LOC12110	1.098	1.293	3.497	0.782	3.759	0.227	2.933	0.4	0.279	0.132	-1.3919	down	8.71E-07
LOC10705	0.256	0.28	1.429	1.569	1.434	0.176	0.293	0.445	0.435	0.258	-1.62526	down	7.33E-09
LOC12110	0.014	0.009	1.624	1.345	1.382	0	0	0.465	0	0	-3.21987	down	7.07E-30
CDIPT	3.413	4.386	3.397	4.007	3.005	6.83	11.149	8.562	9.823	9.866	1.344017	up	2.15E-11

LOC1211(	0.373	0.929	0.476	0.859	2.072	0.925	0.307	0.35	0.142	0.338	-1.18941	down	0.000702
LOC1211(	0.334	0.109	1.13	0.617	0.515	0	0.172	0.13	0.159	0	-2.53989	down	7.31E-08
PKMYT1	0.051	0.117	0.041	0.126	0.235	0.363	1.571	0.358	0.705	0.369	2.551543	up	4.10E-20
LOC1211(	0.534	1.017	0.426	4.591	1.247	0.03	0	0.455	0.046	0	-3.86686	down	1.37E-32
LOC1211(	0.814	0.875	0.538	0.68	1.176	0.128	0.028	0.527	0.053	0.037	-2.39355	down	1.39E-19
LOC1211(	1.881	1.885	2.629	1.665	1.527	0.546	1.257	0.657	0.953	1.083	-1.09159	down	9.01E-06
LOC11253	2.155	2.053	0.757	0.13	4.036	0.118	0.293	0	0	0.057	-4.27165	down	7.79E-45
LOC10705	0.452	0.371	1.943	1.664	2.801	0.687	0.785	0.215	0.713	0.234	-1.4552	down	2.00E-09
LOC11253	3.871	3.576	1.459	0.728	4.37	2.542	0.486	1.407	1.381	0.721	-1.09855	down	1.88E-05
LOC1211(	1.528	1.676	3.059	0.441	6.025	4.475	4.33	6.738	6.382	5.38	1.100744	up	1.68E-09
LOC1211(	8.155	9.755	2.529	2.504	0	0.923	2.092	1.68	2.127	3.529	-1.1479	down	2.17E-07
HSD17B1(	88.222	99.833	145.586	124.656	140.919	43.79	50.86	61.527	48.228	69.522	-1.12927	down	2.11E-06
PFKFB1	84.999	89.914	105.215	93.816	111.586	23.472	33.067	42.552	26.882	45.462	-1.50187	down	5.93E-11
LOC11253	3.581	3.767	1.183	5.585	8.088	5.303	10.937	9.686	9.897	9.937	1.0431	up	4.59E-08
LOC1211(	0.997	0.47	0.794	1.803	0	0	0	0.125	0	0	-4.96809	down	4.31E-32
MHCY13	3.428	2.686	3.329	0.991	1.896	0.326	1.084	2.734	0.898	1.09	-1.00715	down	0.000169
LOC11253	2.993	2.829	7.512	3.722	2.373	0.712	1.029	1.012	1.433	1.081	-1.88216	down	2.67E-15

qvalue	function
1.96E-15	LOC121113333; C-type lectin domain family 2 member B-like isoform X4
1.40E-16	LOC776463; C-type lectin domain family 2 member B-like
0.000336	LOC121109244; C-type lectin domain family 2 member D-like isoform X3
2.08E-09	LOC112532872; C-type lectin domain family 2 member D-like isoform X3
2.08E-05	LOC121109235; thymidine phosphorylase isoform X4
1.81E-07	CD69L; C-type lectin domain family 2 member B
2.33E-15	SHANK3; LOW QUALITY PROTEIN: SH3 and multiple ankyrin repeat domains protein 3 isoform X1
1.06E-14	LOC121109250; BCL-6 corepressor-like protein 1 isoform X1
1.58E-06	ARF5; ADP-ribosylation factor 5
5.92E-66	ELAPOR2; endosome/lysosome-associated apoptosis and autophagy regulator family member 2 isoform X1
7.24E-09	NAPEPLD; N-acyl-phosphatidylethanolamine-hydrolyzing phospholipase D isoform X3
1.01E-13	GTSE1; G2 and S phase-expressed protein 1
7.64E-20	ALG12; dol-P-Man:Man(7)GlcNAc(2)-PP-Dol alpha-1,6-mannosyltransferase isoform X1
1.05E-31	CRELD2; protein disulfide isomerase CRELD2 precursor
0.00042	LOC417741; secreted frizzled-related protein 5
1.63E-06	IQUB; IQ and ubiquitin-like domain-containing protein isoform X4
4.89E-20	CPED1; cadherin-like and PC-esterase domain-containing protein 1 isoform X4
1.48E-10	AMIGO2; amphoterin-induced protein 2 precursor
7.55E-12	SRGAP1; SLIT-ROBO Rho GTPase-activating protein 1 isoform X2
1.24E-08	IFNG; interferon gamma precursor
3.93E-09	LYZ; lysozyme C precursor
0.000234	LOC121109191; uncharacterized protein LOC121109191 isoform X1
2.54E-06	PPFIA2; liprin-alpha-2 isoform X14
3.18E-08	MGP; matrix Gla protein precursor
1.15E-07	HIST1H2B7; histone H2B 7
0.000185	HIST1H2B5; histone H2B 5
0.000209	LOC121106438; histone H3
0.002033	HIST1H2B7L4; histone H2B 1/2/3/4/6
0.000608	APOLD1; apolipoprotein L domain-containing protein 1
2.60E-09	GBE; eye-globin isoform X1
4.63E-10	LOC417973; uncharacterized protein LOC417973 isoform X3
2.17E-21	MEI1; meiosis inhibitor protein 1 isoform X12
6.50E-09	CHADL; chondroadherin-like protein precursor
1.17E-08	ADSL; adenylosuccinate lyase
2.02E-27	NPTXR; neuronal pentraxin receptor
1.29E-10	DNAL4; dynein light chain 4, axonemal

1.42E-27 FAM20CL; extracellular serine/threonine protein kinase FAM20C isoform X1
2.15E-16 SUN2; SUN domain-containing protein 2
2.38E-28 CARD10; caspase recruitment domain-containing protein 10 isoform X7
1.53E-10 MCM5; DNA replication licensing factor MCM5
6.82E-06 C12orf73; uncharacterized protein C12orf73 homolog
4.11E-29 HSP90B1; endoplasmin precursor
1.30E-16 ASCL1; achaete-scute homolog 1
6.94E-05 PAH; phenylalanine-4-hydroxylase
4.55E-05 IGF1; insulin-like growth factor I preproprotein
4.55E-07 TRIM24; transcription intermediary factor 1-alpha
4.76E-16 CGTL; cystine/glutamate transporter isoform X4
7.21E-05 LOC418114; pseudouridine-metabolizing bifunctional protein C1861.05 isoform X3
2.06E-05 LOC121106531; uncharacterized protein LOC121106531 isoform X1
1.28E-20 SLCO1B1; solute carrier organic anion transporter family member 1C1 isoform X1
7.15E-07 SOX5; transcription factor SOX-5 isoform X14
1.41E-24 ENDOUL; poly(U)-specific endoribonuclease-A isoform X1
8.51E-06 MPPED1; metallophosphoesterase domain-containing protein 1
1.23E-10 SULT4A1; sulfotransferase 4A1 isoform X2
2.23E-20 PNPLA3; patatin-like phospholipase domain-containing protein 2 isoform X3
2.03E-08 KIAA0930; uncharacterized protein KIAA0930 homolog isoform X3
1.60E-09 LOC426193; tetratricopeptide repeat protein 38
1.11E-07 MANSC1; MANSC domain-containing protein 1 precursor
1.77E-11 LOC101751545; cyclin-dependent kinase inhibitor 1 isoform X3
9.01E-29 FOXM1; forkhead box protein M1
2.39E-07 A2ML3; alpha-2-macroglobulin-like isoform X6
2.97E-12 CASR; extracellular calcium-sensing receptor
4.64E-11 CNP1; C-type natriuretic peptide 1 precursor
9.83E-23 GNB3; guanine nucleotide-binding protein G(I)/G(S)/G(T) subunit beta-3
5.81E-46 CDCA3; cell division cycle-associated protein 3 isoform 2
7.08E-07 LRRC23; leucine-rich repeat-containing protein 23 isoform X1
4.83E-08 EPHA1; ephrin type-A receptor 1 precursor
3.74E-07 GSTK1; glutathione S-transferase kappa 1 isoform X1
1.63E-11 KEL; kell blood group glycoprotein isoform X4
8.12E-06 STYK1; tyrosine-protein kinase STYK1
8.04E-05 LOC100859872; uncharacterized protein LOC100859872 isoform X1
1.44E-06 HAO2; hydroxyacid oxidase 2
4.82E-09 MAB21L3; protein mab-21-like 3 isoform X1
4.17E-16 SLC22A15; solute carrier family 22 member 15 isoform X3

3.50E-08 ZBTB20; zinc finger and BTB domain-containing protein 20 isoform X2
4.09E-19 CLDND1; claudin domain-containing protein 1 isoform X2
3.08E-20 APOV1; apovitellenin-1 precursor
9.45E-15 SLC19A2; thiamine transporter 1
1.62E-14 DPT; dermatopontin
1.93E-08 NIT2; omega-amidase NIT2 isoform X2
4.38E-08 NFKBIZ; NF-kappa-B inhibitor zeta isoform X3
2.37E-34 KIAA1524; protein CIP2A homolog
1.15E-16 LOC418414; uncharacterized protein C3orf85 homolog
2.33E-92 CD200L; uncharacterized protein LOC418424 precursor
4.16E-83 LOC121110262; uncharacterized protein LOC121110262 isoform X1
4.26E-08 CREG1; protein CREG1 precursor
9.52E-23 ROBO1; roundabout homolog 1 isoform X7
2.27E-08 ADAMTS1; LOW QUALITY PROTEIN: A disintegrin and metalloproteinase with thrombospondin motifs 1 isoform X1
2.46E-08 MAP3K7CL; MAP3K7 C-terminal-like protein isoform X4
2.06E-05 EVA1C; protein eva-1 homolog C isoform X5
8.67E-05 CBR3; carbonyl reductase [NADPH] 1
6.25E-10 PCP4; calmodulin regulator protein PCP4
1.31E-12 MX1; interferon-induced GTP-binding protein Mx isoform X3
1.29E-14 SIK1; serine/threonine-protein kinase SIK2
1.08E-10 MAOB; amine oxidase [flavin-containing] B
4.97E-31 MID1IP1; mid1-interacting protein 1
1.56E-23 OTC; ornithine transcarbamylase, mitochondrial isoform X1
0.000189 DMD; dystrophin
1.82E-15 NR0B1; nuclear receptor subfamily 0 group B member 1 isoform X2
2.87E-55 SLC51AL; organic solute transporter subunit alpha
9.43E-16 MAP3K15; mitogen-activated protein kinase kinase kinase 15
1.49E-14 PDHA2; pyruvate dehydrogenase E1 component subunit alpha, somatic form, mitochondrial precursor
7.73E-39 ADGRG2; adhesion G-protein coupled receptor G2 isoform X1
5.95E-10 LOC121106447; uncharacterized protein LOC121106447
3.90E-38 LOC107052718; LOW QUALITY PROTEIN: uncharacterized protein LOC107052718
4.69E-05 RGN; regucalcin isoform X3
7.73E-11 MRPL30; 39S ribosomal protein L30, mitochondrial
4.94E-20 LYGL; lysozyme g isoform X1
6.42E-28 LYG2; lysozyme g isoform X1
4.18E-09 IL1R2; interleukin-1 receptor type 2 isoform X1
1.54E-23 IL1RL1; interleukin-1 receptor-like 1 isoform LV precursor
1.16E-09 SEPT14; septin-10 isoform X3

1.12E-06 LOC771012; coagulation factor X
3.07E-13 LOC121107687; SPARC
5.43E-12 HS6ST3; heparan-sulfate 6-O-sulfotransferase 3
7.13E-07 DCT; L-dopachrome tautomerase precursor
4.43E-39 ACOD1; cis-aconitate decarboxylase
7.05E-05 PCDH20; protocadherin-20
1.33E-05 CPB2; carboxypeptidase B2
7.98E-29 CKAP2; cytoskeleton-associated protein 2
1.53E-05 N4BP2L1; NEDD4-binding protein 2-like 1 isoform X1
2.26E-05 FLT1; vascular endothelial growth factor receptor 1 precursor
5.50E-60 GJB6; gap junction beta-6 protein isoform X1
2.33E-66 GJB2; gap junction beta-2 protein
3.25E-08 ZC3H12C; probable ribonuclease ZC3H12C isoform X3
5.88E-06 SLC35F2; solute carrier family 35 member F2 isoform X1
1.35E-06 ENDOD1; endonuclease domain-containing 1 protein
3.98E-05 TAF1D; TATA box-binding protein-associated factor RNA polymerase I subunit D
8.63E-05 FZD4; frizzled-4 precursor
1.29E-07 AQP11; aquaporin-11
9.87E-25 CAPN5; calpain-5
3.37E-19 TSKU; tsukushin precursor
5.97E-29 WNT11; protein Wnt-11 isoform X4
2.42E-08 ART7B; uncharacterized protein LOC428124 precursor
2.90E-26 LOC121109083; glutamine-rich protein 2-like isoform X1
1.58E-05 PDE2A; LOW QUALITY PROTEIN: cGMP-dependent 3',5'-cyclic phosphodiesterase isoform X1
2.53E-45 ADAM15; disintegrin and metalloproteinase domain-containing protein 9 isoform X3
2.25E-25 LOC107052463; olfactory receptor 51G2-like isoform X1
1.08E-39 HBE; hemoglobin subunit epsilon
1.43E-26 HBBA; hemoglobin subunit beta
1.83E-47 HBE1; hemoglobin subunit epsilon 1
2.86E-07 TAF10; transcription initiation factor TFIID subunit 10
6.10E-15 TPP1; tripeptidyl-peptidase 1
0.000345 LOC107055991; autophagy-related protein 9B
2.86E-13 AOC1; amiloride-sensitive amine oxidase [copper-containing] isoform X1
7.01E-05 GIMAP1-GIMAP5; GTPase IMAP family member 1
2.44E-10 ELP6; elongator complex protein 6
1.97E-08 SCAP; sterol regulatory element-binding protein cleavage-activating protein isoform X2
5.22E-54 ACKR2; atypical chemokine receptor 2
1.48E-60 CYP8B1; cytochrome P450, family 8, subfamily B

1.30E-20 CHDSD; D-threo-3-hydroxyaspartate dehydratase isoform X3
1.36E-09 CATH3; cathelicidin-3 precursor
8.65E-24 CATH2; cathelicidin-2 precursor
3.52E-14 CATH1; cathelicidin-1 precursor
1.50E-10 GIMAP6; GTPase IMAP family member 5
5.50E-07 DLEC1; deleted in lung and esophageal cancer protein 1 isoform X3
7.00E-09 ACAA1; 3-ketoacyl-CoA thiolase, peroxisomal
4.48E-11 SLC22A13; solute carrier family 22 member 13
0.000209 CRYGN; gamma-crystallin N isoform X2
1.35E-13 MAP3K8; mitogen-activated protein kinase kinase kinase 8 isoform X1
0.000118 PTER; phosphotriesterase-related protein
3.14E-24 ACBD7; acyl-CoA-binding domain-containing protein 7 isoform X2
8.10E-19 OLAH; S-acyl fatty acid synthase thioesterase, medium chain isoform X1
1.10E-41 ABCB1LA; phosphatidylcholine translocator ABCB4 isoform X5
5.70E-16 CYP51A1; leucine-rich repeat and death domain-containing protein 1 isoform X2
1.43E-09 CDK6; cyclin-dependent kinase 6
0.000121 LOC420562; probable acyl-CoA dehydrogenase 6 isoform X2
1.05E-07 COL1A2; collagen alpha-2(I) chain precursor
1.85E-16 ICA1; islet cell autoantigen 1 isoform X1
7.81E-17 VWDE; von Willebrand factor D and EGF domain-containing protein
2.98E-06 HOXA2; homeobox protein Hox-A2
1.59E-07 FKBP14; peptidyl-prolyl cis-trans isomerase FKBP14
1.97E-13 SATB1; DNA-binding protein SATB1 isoform X1
9.97E-05 LOC101750511; BCL-6 corepressor-like
0.000121 MTURN; maturin, neural progenitor differentiation regulator homolog
1.82E-08 ACAD11; acyl-CoA dehydrogenase family member 11
2.56E-17 TGM4; protein-glutamine gamma-glutamyltransferase 4
5.25E-31 KIF15; kinesin-like protein KIF15
4.00E-07 TRANK1; TPR and ankyrin repeat-containing protein 1 isoform X2
3.87E-32 ANLN; anillin isoform X2
4.40E-09 AMPH; amphiphysin isoform X1
0.000154 _
9.83E-15 PDIA4; protein disulfide-isomerase A4 isoform X1
7.16E-08 LOC121109603; uncharacterized protein LOC121109603
3.96E-10 KCNG2; potassium voltage-gated channel subfamily G member 2 isoform X1
1.87E-20 DCDC2; doublecortin domain-containing protein 2 isoform X4
2.57E-14 ID4; DNA-binding protein inhibitor ID-4
1.84E-15 ELOVL2; elongation of very long chain fatty acids protein 2

8.94E-16 SYCP2L; synaptonemal complex protein 2-like isoform X3
4.51E-23 GCNT2; N-acetyllactosaminide beta-1,6-N-acetylglucosaminyl-transferase isoform X2
1.14E-12 F13A1; coagulation factor XIII A chain
7.11E-12 PPP1R3G; protein phosphatase 1 regulatory subunit 3G
5.17E-07 ECI2; enoyl-CoA delta isomerase 2 isoform X3
2.48E-10 SERPINB1; leukocyte elastase inhibitor isoform X1
9.90E-07 LOC420903; MARVEL domain-containing protein 3 isoform X2
1.56E-12 ANKRD33B; ankyrin repeat domain-containing protein 33B isoform X2
3.77E-32 CMBL; carboxymethylenebutenolidase homolog
4.47E-10 SRD5A1; 3-oxo-5-alpha-steroid 4-dehydrogenase 1 isoform X3
1.36E-07 SEC61B; protein transport protein Sec61 subunit beta
3.54E-28 IFI6; interferon alpha inducible protein 6
6.18E-07 GLIPR2; Golgi-associated plant pathogenesis-related protein 1
0.000218 CIDEA; cell death activator CIDE-A
4.61E-06 TGIF1; homeobox protein AKR
0.000157 LPIN2; phosphatidate phosphatase LPIN2 isoform X1
8.52E-10 NDC80; kinetochore protein NDC80 homolog isoform X1
5.82E-39 ABHD3; phospholipase ABHD3 isoform X1
5.69E-10 LAMA3; LOW QUALITY PROTEIN: laminin subunit alpha-3 isoform X1
2.17E-67 DSC1; desmocollin-2 isoform X4
1.73E-12 MCM4; DNA replication licensing factor MCM4
3.61E-05 C8orf22; pancreatic progenitor cell differentiation and proliferation factor-like protein
1.62E-05 FAM110B; protein FAM110B isoform X1
9.27E-26 CYP7A1; cholesterol 7-alpha-monooxygenase
1.57E-05 MTFR1; mitochondrial fission regulator 1 isoform X2
2.23E-15 XKR9; XK-related protein 9 isoform X2
3.44E-06 MSC; musculin
1.64E-33 TRPA1; transient receptor potential cation channel subfamily A member 1
1.64E-14 GDAP1; ganglioside-induced differentiation-associated protein 1 isoform X1
2.21E-20 FABP4; fatty acid-binding protein, adipocyte
4.14E-16 CA13; carbonic anhydrase 13
5.26E-37 ATP6V0D2; V-type proton ATPase subunit d 2
0.000176 TMEM55A; type 2 phosphatidylinositol 4,5-bisphosphate 4-phosphatase
1.84E-09 PTDSS1; phosphatidylserine synthase 1 isoform X1
7.61E-09 NCALD; neurocalcin-delta
7.80E-17 RIMS2; regulating synaptic membrane exocytosis protein 2 isoform X50
0.000151 LOC121109903; basic proline-rich protein-like
1.09E-11 SAMD12; sterile alpha motif domain-containing protein 12 isoform X3

3.54E-05 ENPP2; ectonucleotide pyrophosphatase/phosphodiesterase family member 2 isoform X14
1.10E-20 SQLE; squalene monooxygenase
3.02E-05 MYC; myc proto-oncogene protein
1.77E-22 LOC428383; leucine-rich repeat protein lrrA-like
2.18E-07 RHPN1; rhophilin-1 isoform X1
0.000218 LOC121113091; uncharacterized protein LOC121113091
2.30E-11 NRBP2; nuclear receptor-binding protein 2 isoform X2
0.000234 FANCL; E3 ubiquitin-protein ligase FANCL isoform X1
1.17E-11 KIAA1841; uncharacterized protein KIAA1841 homolog isoform X6
1.92E-08 BUB1; mitotic checkpoint serine/threonine-protein kinase BUB1
3.44E-08 CAPN13; calpain-13 isoform X2
1.21E-07 UGP2; UTP--glucose-1-phosphate uridylyltransferase isoform X1
2.68E-07 BIRC5; survivin isoform 3
4.80E-11 RRBP1; ribosome-binding protein 1 isoform X2
1.16E-10 GINS1; DNA replication complex GINS protein PSF1
1.63E-05 CAPN2; calpain-2 catalytic subunit
4.87E-14 TLR5; toll-like receptor 5 precursor
3.66E-05 MAD2L1BP; MAD2L1-binding protein
4.77E-06 RMDN2; regulator of microtubule dynamics protein 2 isoform X3
6.14E-09 PLD5; inactive phospholipase D5 isoform X5
1.93E-16 GREM2; gremlin-2
1.62E-05 SLC35F3; putative thiamine transporter SLC35F3 isoform X2
3.38E-09 GNPAT; dihydroxyacetone phosphate acyltransferase isoform X1
9.96E-08 SULT; sulfotransferase
5.41E-13 DACT2; dapper homolog 2
0.000179 AGPAT4; 1-acyl-sn-glycerol-3-phosphate acyltransferase delta isoform X2
1.58E-15 LOC421583; solute carrier family 22 member 2
1.21E-14 FNDC1; fibronectin type III domain-containing protein 1
1.97E-08 PERPB; p53 apoptosis effector related to PMP-22
9.31E-08 SGK1; serine/threonine-protein kinase Sgk1
8.28E-14 LOC107056139; pantetheinase-like precursor
1.59E-32 CENPW; centromere protein W
7.56E-31 HEY2; hairy/enhancer-of-split related with YRPW motif protein 2
5.24E-20 GJA1; gap junction alpha-1 protein
2.35E-06 MCM9; DNA helicase MCM9 isoform X1
1.10E-21 DCBLD1; discoidin, CUB and LCCL domain-containing protein 1 isoform X2
4.41E-53 RFX6; DNA-binding protein RFX6 isoform X1
2.30E-08 LOC421740; sulfotransferase family 3A, member 1-like

3.15E-18	LOC121110238; uncharacterized protein LOC121110238 isoform X1
1.81E-05	SLC22A16; solute carrier family 22 member 16 isoform X2
2.36E-07	LOC421792; thiosulfate sulfurtransferase/rhodanese-like domain-containing protein 3
2.62E-08	MMS22L; protein MMS22-like isoform X1
0.000146	SMIM8; small integral membrane protein 8 isoform X1
1.37E-17	ME1; NADP-dependent malic enzyme
7.36E-09	RWDD2A; RWD domain-containing protein 2A
0.003409	IRAK1BP1; interleukin-1 receptor-associated kinase 1-binding protein 1
6.38E-09	FAM110C; protein FAM110C
9.98E-08	CMPK2; UMP-CMP kinase 2, mitochondrial
3.63E-10	MBOAT2; lysophospholipid acyltransferase 2 isoform X1
7.17E-36	RRM2; ribonucleoside-diphosphate reductase subunit M2
1.44E-10	ATP6V1C2; V-type proton ATPase subunit C 2 isoform X2
1.89E-19	PDIA6; protein disulfide-isomerase A6
7.89E-06	VSNL1; visinin-like protein 1 isoform X2
6.51E-21	RHOB; rho-related GTP-binding protein RhoB
1.01E-08	APOB; apolipoprotein B precursor
5.93E-09	TP53I3; quinone oxidoreductase PIG3 isoform X8
0.000222	SLC5A6; sodium-dependent multivitamin transporter
9.33E-06	MPV17; translation initiation factor IF-2 isoform X4
5.31E-05	DTNB; dystrobrevin beta isoform X11
3.91E-24	KIF3C; kinesin-like protein KIF3C
6.30E-05	HADHB; trifunctional enzyme subunit beta, mitochondrial
1.92E-09	LOC121110416; protein aveugle-like isoform X1
9.82E-13	GVINP1; interferon-induced very large GTPase 1
2.04E-09	LOC770271; uncharacterized protein LOC770271
1.04E-16	LOC770996; L-gulonolactone oxidase
1.02E-38	PBK; lymphokine-activated killer T-cell-originated protein kinase isoform X1
3.18E-11	CLU; clusterin precursor
2.25E-10	LOC426385; serine/threonine-protein kinase 35-like isoform X1
3.52E-07	MSRA; mitochondrial peptide methionine sulfoxide reductase isoform X14
4.82E-68	SOX7; transcription factor SOX-7
3.80E-07	XKR6; XK-related protein 6
1.71E-18	FDFT1; squalene synthase
0.000151	AvBD13; gallinacin-13 precursor
4.27E-11	AvBD7; gallinacin-7 preproprotein
3.08E-17	DEFB4A; gallinacin-2 precursor
1.23E-12	AvBD1; gallinacin-1 alpha precursor

1.12E-24	MCM3; DNA replication licensing factor MCM3 isoform X1
5.48E-15	CRISP2; serotriflin isoform X1
2.15E-26	RHAG; ammonium transporter Rh type A
1.15E-15	CYP2AC1; cytochrome P450 2AC1
3.30E-07	SUPT3H; transcription initiation protein SPT3 homolog isoform X2
7.03E-11	RCAN2; calcipressin-2 isoform X2
0.00014	CYP39A1; 24-hydroxycholesterol 7-alpha-hydroxylase isoform X5
1.95E-09	ADGRF5; adhesion G protein-coupled receptor F5
1.16E-16	ARR3; arrestin-C
6.70E-36	KIF4B; chromosome-associated kinesin KIF4
0.000103	RAB33A; RAB33A, member RAS oncogene family
3.99E-29	CCNB3; G2/mitotic-specific cyclin-B3 isoform X2
3.30E-31	CENPI; centromere protein I
1.52E-09	GCNA; acidic repeat-containing protein isoform X1
8.94E-16	SLC6A14; sodium- and chloride-dependent neutral and basic amino acid transporter B(0+) isoform X2
5.84E-09	GPC3; glypican-3
3.32E-11	CLCN5; H(+)/Cl(-) exchange transporter 5 isoform X3
4.05E-23	ZNF185; zinc finger protein 185 isoform X5
3.51E-11	NSDHL; sterol-4-alpha-carboxylate 3-dehydrogenase, decarboxylating
1.69E-08	SLC16A2; monocarboxylate transporter 8
2.06E-05	COL4A5; collagen alpha-5(IV) chain isoform X1
2.55E-11	LOC121110638; uncharacterized protein LOC121110638
3.72E-14	SH2D1A; SH2 domain-containing protein 1A isoform X1
1.98E-05	IDS; iduronate 2-sulfatase isoform X4
6.61E-10	TRIM2; tripartite motif-containing protein 2 isoform X2
1.54E-15	MSMO1; methylsterol monooxygenase 1
6.64E-11	LOC107052719; uncharacterized protein LOC107052719 isoform X1
2.20E-07	DDX60; probable ATP-dependent RNA helicase DDX60
6.89E-13	TBC1D9; TBC1 domain family member 9 isoform X3
4.12E-13	DCLK2; serine/threonine-protein kinase DCLK2 isoform X3
1.31E-10	SH2D4A; SH2 domain-containing protein 4A
0.000612	RBPMS; RNA-binding protein with multiple splicing
3.98E-20	MMRN1; multimerin-1
1.52E-09	ARHGEF38; rho guanine nucleotide exchange factor 38
1.06E-06	LRP2BP; LRP2-binding protein isoform X1
2.39E-16	ACSL1; long-chain-fatty-acid--CoA ligase 1
2.62E-08	HMGB2; high mobility group protein B2
0.000151	VEGFC; vascular endothelial growth factor C

0.000702 EREG; proepiregulin precursor
0.000158 AREG; amphiregulin isoform X1
0.000327 SPARCL1; SPARC-like protein 1
2.36E-09 PLACL2; PLAC8 like 2
1.82E-11 GPAT3; glycerol-3-phosphate acyltransferase 3 isoform X1
8.87E-08 DUSP4; dual specificity protein phosphatase 4
6.85E-35 LOC430303; prolow-density lipoprotein receptor-related protein 1 isoform X3
9.10E-07 CL2; ribonuclease CL2 precursor
5.92E-09 SHROOM3; protein Shroom3 isoform X7
6.04E-09 DCK; deoxycytidine kinase
4.87E-08 LOC100858497; uncharacterized protein LOC100858497
3.64E-07 ADAMTS3; A disintegrin and metalloproteinase with thrombospondin motifs 3 isoform X3
9.42E-13 SULT1B; sulfotransferase family, cytosolic, 1B isoform X1
2.39E-18 CCNA2; cyclin-A2
4.64E-07 MAD2L1; mitotic spindle assembly checkpoint protein MAD2A
0.001781 FABP2; fatty acid-binding protein, intestinal
7.74E-08 PITX2; pituitary homeobox 2
1.19E-17 ENPEP; glutamyl aminopeptidase
0.000196 ADH6; alcohol dehydrogenase 6
3.88E-06 LOC100857280; alcohol dehydrogenase 1-like
0.00062 EMCN; endomucin precursor
2.80E-06 SLC39A8; metal cation symporter ZIP8 isoform X2
2.19E-37 CENPE; centromere-associated protein E isoform X3
2.64E-44 CCDC110; coiled-coil domain-containing protein 110
1.70E-05 CYP4V2; cytochrome P450 family 4 subfamily V member 2
2.61E-14 PDGFRL; platelet-derived growth factor receptor-like protein isoform X5
2.56E-05 UGDH; UDP-glucose 6-dehydrogenase
1.06E-09 FAM114A1; protein NOXP20 isoform X1
3.19E-09 TLR1A; toll-like receptor1 isoform X1
1.39E-22 RBPJ; recombining binding protein suppressor of hairless isoform X1
0.000379 SEL1L3; protein sel-1 homolog 3
1.34E-07 SOD3; extracellular superoxide dismutase [Cu-Zn]
5.04E-15 PPARGC1A; peroxisome proliferator-activated receptor gamma coactivator 1-alpha isoform X1
5.04E-18 NCAPG; condensin complex subunit 3 isoform X1
1.44E-07 BST1; ADP-ribosyl cyclase 2 isoform X3
1.19E-09 HAUS3; HAUS augmin-like complex subunit 3
5.85E-18 SLBP; histone RNA hairpin-binding protein
2.78E-50 SPON2; spondin-2 isoform X2

2.33E-38	LOC107049163; T-cell surface glycoprotein CD8 alpha chain-like isoform X1
1.05E-29	LOC121106480; uncharacterized protein LOC121106480 isoform X2
2.37E-34	SMYD1; histone-lysine N-methyltransferase SMYD1
1.42E-12	FABP1; fatty acid-binding protein, liver
6.60E-05	ADRA1D; alpha-1D adrenergic receptor
2.85E-06	CYP26B1; cytochrome P450 26B1 isoform X1
5.68E-20	NAT8; putative N-acetyltransferase 8B
1.25E-06	NAT8B; N-acetyltransferase 8B (GCN5-related, putative, gene/pseudogene)
1.72E-20	MRGPRH; proto-oncogene Mas isoform X2
1.69E-11	LOC107056412; membrane-spanning 4-domains subfamily A member 12-like isoform X3
1.06E-08	TMEM109; transmembrane protein 109
3.15E-07	VWCE; von Willebrand factor C and EGF domain-containing protein isoform X4
1.15E-13	DAGLA; diacylglycerol lipase-alpha isoform X2
4.86E-17	TCIRG1; V-type proton ATPase 116 kDa subunit a3
0.000151	ALDH3B1; aldehyde dehydrogenase family 3 member B1
7.18E-12	SPTB; spectrin beta chain, erythrocytic isoform X1
1.63E-05	NUDT8; nucleoside diphosphate-linked moiety X motif 8
1.65E-40	BUB1B; mitotic checkpoint serine/threonine-protein kinase BUB1 beta
1.45E-05	C15orf52; coiled-coil domain-containing protein 9B isoform X6
2.22E-16	KNSTRN; small kinetochore-associated protein
9.51E-06	CHST14; carbohydrate sulfotransferase 14
6.84E-05	ANO9; anoctamin-9 isoform X1
6.24E-11	LOC770612; interferon-induced transmembrane protein 3
3.76E-22	DHCR7; 7-dehydrocholesterol reductase
1.81E-22	E2F8; transcription factor E2F8 isoform X2
8.62E-10	RCN1; reticulocalbin-1 isoform X1
2.48E-15	PRRG4; transmembrane gamma-carboxyglutamic acid protein 4 isoform X1
2.15E-17	MICAL2; F-actin-monooxygenase MICAL2 isoform X20
0.0003	AMPD3; AMP deaminase 3 isoform X2
0.000221	ST5; DENN domain-containing protein 2B isoform X4
2.46E-06	CALCA; calcitonin isoform 1 preproprotein
2.68E-09	NUCB2; nucleobindin-2 isoform X1
#######	SAA; serum amyloid A isoform X2
1.21E-10	TMEM86A; lysoplasmalogenase-like protein TMEM86A
8.34E-07	KCNQ1; potassium voltage-gated channel subfamily KQT member 1
1.69E-83	TRPM5; transient receptor potential cation channel subfamily M member 5 isoform X1
2.98E-15	DUSP8; dual specificity protein phosphatase 8 isoform X2
4.87E-08	PNPLA2; patatin-like phospholipase domain-containing protein 2

2.71E-23 CHKA; choline kinase alpha isoform X2
0.000712 TESMIN; tesmin isoform X2
2.04E-12 CPT1A; carnitine O-palmitoyltransferase 1, liver isoform isoform X2
1.79E-06 FADS1; acyl-CoA (8-3)-desaturase
2.98E-11 SMTNL1; smoothelin-like protein 1
0.000138 RTN4RL2; reticulon-4 receptor-like 2 isoform X2
6.14E-13 PGR2/3; P2X purinoceptor 3 precursor
4.07E-17 INCENP; inner centromere protein
2.12E-22 SMIM38; small integral membrane protein 38
1.57E-75 FGF19; fibroblast growth factor 19 precursor
1.17E-09 EHF; ETS homologous factor isoform X1
2.06E-36 ACCS; 1-aminocyclopropane-1-carboxylate synthase-like protein 1 isoform X3
7.13E-11 MDK; midkine isoform X1
3.78E-19 CHAC1; glutathione-specific gamma-glutamylcyclotransferase 1
4.01E-33 NUSAP1; nucleolar and spindle-associated protein 1
3.04E-08 LTK; leukocyte tyrosine kinase receptor isoform X1
7.10E-81 SPTBN5; spectrin beta chain, non-erythrocytic 5
1.40E-07 EHD4; EH domain-containing protein 4
2.32E-06 PCNX1; pecanex-like protein 1 isoform X3
0.000138 PLEKHD1; pleckstrin homology domain-containing family D member 1 isoform X4
8.26E-08 GALNT16; polypeptide N-acetylgalactosaminyltransferase 16 isoform X2
4.93E-15 RDH11; retinol dehydrogenase 12
0.000347 LGALSL2; galectin-related protein A isoform X3
1.35E-08 LOC112532477; fibrinogen-like protein 1-like protein
2.18E-07 SLC25A21; mitochondrial 2-oxodicarboxylate carrier isoform X8
5.48E-06 TTC6; tetratricopeptide repeat protein 6 isoform X3
2.96E-21 ACSS1B; acetyl-coenzyme A synthetase 2-like, mitochondrial isoform X2
4.09E-06 TMEM63C; calcium permeable stress-gated cation channel 1 isoform X1
1.39E-79 SPIA3; alpha-1-antiproteinase isoform X1
4.44E-18 LOC107049127; serpin A3-4-like isoform X1
2.31E-24 BDKRB1; B1 bradykinin receptor
4.50E-69 LOC121110856; uncharacterized protein LOC121110856
1.29E-27 AMN; protein amnionless isoform X1
2.27E-06 BRF1; transcription factor IIIB 90 kDa subunit isoform X2
0.000215 MTHFD1; C-1-tetrahydrofolate synthase, cytoplasmic
0.000111 SYNE2; nesprin-2 isoform X1
6.59E-06 DHRS7; dehydrogenase/reductase SDR family member 7
1.87E-33 DLGAP5; disks large-associated protein 5

1.30E-07	GCH1; GTP cyclohydrolase 1
0.000221	VCPKMT; protein-lysine methyltransferase METTL21D isoform X3
7.73E-08	SOS2; son of sevenless homolog 2 isoform X2
1.62E-06	LOC107051813; prostaglandin D2 receptor-like
0.00041	STYX; serine/threonine/tyrosine-interacting protein isoform X1
5.52E-11	LOC112532663; uncharacterized protein LOC112532663 isoform X1
0.000138	LOC121106456; protein MANBAL-like
1.43E-17	GRID1; glutamate receptor ionotropic, delta-1 isoform X3
7.36E-20	SNCG; gamma-synuclein
7.87E-08	DYDC2; DPY30 domain-containing protein 1
0.000537	SFTPA1; surfactant protein A1 precursor
6.11E-10	RET; proto-oncogene tyrosine-protein kinase receptor Ret precursor
3.43E-07	DNAJC12; dnaJ homolog subfamily C member 12
4.67E-05	RHOBTB1; rho-related BTB domain-containing protein 1 isoform X1
1.50E-10	CDK1; cyclin-dependent kinase 1 isoform X1
1.40E-08	RNASE6; angiogenin precursor
1.73E-05	PAOX; peroxisomal N(1)-acetyl-spermine/spermidine oxidase isoform X2
2.00E-09	PAPSS2; bifunctional 3'-phosphoadenosine 5'-phosphosulfate synthase 2 isoform X3
9.60E-22	PRKG1; cGMP-dependent protein kinase 1 isoform X3
#######	HKDC1; hexokinase HKDC1 isoform X3
9.53E-07	NPFFR1; neuropeptide FF receptor 1
2.15E-26	CYP2C18; cytochrome P450 family 2 subfamily C member 18
1.14E-32	SCD; stearoyl-CoA desaturase
6.10E-15	CYP2C23b; cytochrome P450 2H2 precursor
2.18E-10	CYP2C23a; cytochrome P450 2H1 precursor
4.24E-11	MSMB; beta-microseminoprotein
4.60E-71	LOC112532694; bone morphogenetic protein 7-like
8.10E-19	ANKRD22; ankyrin repeat domain-containing protein 22 isoform X1
3.74E-07	PANK1; pantothenate kinase 1 isoform X3
1.18E-07	KIF11; kinesin-like protein KIF11 isoform X2
8.47E-06	CPN1; carboxypeptidase N catalytic chain precursor
0.000372	LOXL4; lysyl oxidase homolog 4 precursor
6.81E-58	CRTAC1; cartilage acidic protein 1
1.01E-08	PI4K2A; LOW QUALITY PROTEIN: phosphatidylinositol 4-kinase type 2-alpha
5.39E-36	HOGA1; 4-hydroxy-2-oxoglutarate aldolase, mitochondrial
1.92E-07	ANKRD2; ankyrin repeat domain-containing protein 2 isoform X2
2.53E-28	TLX1; T-cell leukemia homeobox protein 1
5.56E-11	PDZD7; PDZ domain-containing protein 7 isoform X4

1.11E-09	CYP17A1; steroid 17-alpha-hydroxylase/17,20 lyase precursor
1.88E-09	GSTO2; glutathione S-transferase omega-1
4.84E-15	GPAM; glycerol-3-phosphate acyltransferase 1, mitochondrial isoform X1
2.32E-20	LOC101750892; deleted in malignant brain tumors 1 protein-like isoform X2
0.001502	FAM196A; inhibitory synaptic factor 2A isoform X4
6.40E-39	MKI67; proliferation marker protein Ki-67 isoform X4
3.57E-08	COL3A1; collagen alpha-1(III) chain precursor
2.43E-62	ABCA12; ATP-binding cassette sub-family A member 12 isoform X2
1.80E-23	ATIC; bifunctional purine biosynthesis protein ATIC
6.88E-08	LOC107048987; putative methyltransferase DDB_G0268948
4.17E-07	TRPM8; transient receptor potential cation channel subfamily M member 8
8.22E-05	UGT1A1; UDP-glucuronosyltransferase 1A1 isoform X4
2.25E-43	LOC121111295; UDP-glucuronosyltransferase 1A1-like
1.80E-24	AHR2; aryl hydrocarbon receptor 2 isoform X4
6.91E-20	AHR1B; aryl hydrocarbon receptor 1 beta isoform X2
1.20E-08	SLC19A1; reduced folate transporter isoform X2
2.88E-16	LOC768589; baculoviral IAP repeat-containing protein 5.1 isoform X1
8.25E-05	COQ10B; coenzyme Q-binding protein COQ10 homolog B, mitochondrial
1.55E-24	AOX1; aldehyde oxidase isoform X4
1.35E-05	RAPH1; ras-associated and pleckstrin homology domains-containing protein 1 isoform X4
0.000256	LOC424111; uncharacterized protein LOC424111
0.00011	IDH1; isocitrate dehydrogenase [NADP] cytoplasmic isoform X2
1.73E-05	PPP1R1C; protein phosphatase 1 regulatory subunit 1C isoform X2
0.000101	OSBPL6; oxysterol-binding protein-related protein 6 isoform X1
1.03E-18	PDE11A; dual 3',5'-cyclic-AMP and -GMP phosphodiesterase 11A isoform X3
1.11E-17	CDCA7; cell division cycle-associated protein 7 isoform X2
7.48E-08	RAPGEF4; rap guanine nucleotide exchange factor 4 isoform X5
1.92E-06	G6PC2; glucose-6-phosphatase 2 isoform X5
2.01E-22	SPC25; kinetochore protein Spc25
2.31E-11	DPP4; dipeptidyl peptidase 4
2.93E-29	ITGB6; integrin beta-6 isoform X1
9.35E-05	LOC107049146; gap junction gamma-1 protein-like
2.60E-32	LOC424199; uncharacterized protein LOC424199 isoform X1
4.19E-07	TUB4A; tubulin alpha-5 chain isoform X2
6.26E-10	IHH; indian hedgehog protein precursor
7.21E-05	CYP27A1; sterol 26-hydroxylase, mitochondrial
8.30E-10	PLCD4; 1-phosphatidylinositol 4,5-bisphosphate phosphodiesterase delta-4 isoform X7
4.91E-23	IGFBP2; insulin-like growth factor-binding protein 2 isoform X1

1.26E-06	TMEM169; transmembrane protein 169
5.38E-05	PECR; peroxisomal trans-2-enoyl-CoA reductase isoform X1
9.50E-11	ARHGEF1; uncharacterized protein ARHGEF1 isoform X3
1.12E-07	EAF2; ELL-associated factor 2
4.35E-06	SEMA5B; semaphorin-5B isoform X5
2.59E-23	SLC12A8; solute carrier family 12 member 8 isoform X1
5.64E-15	SCTR; secretin receptor precursor
1.47E-08	NXPH2; neurexophilin-2 isoform X1
2.90E-16	ASPM; abnormal spindle-like microcephaly-associated protein isoform X3
0.000475	CRIP2; cysteine-rich protein 2 isoform X2
4.90E-05	REG4; regenerating islet-derived protein 4 precursor
2.98E-06	LOC100857694; flavin-containing monooxygenase 5-like
6.26E-20	LOC107053928; tumor necrosis factor ligand superfamily member 18-like
3.51E-07	FASLG; tumor necrosis factor ligand superfamily member 6 isoform X1
6.20E-15	SUCO; SUN domain-containing ossification factor isoform X5
4.43E-09	FMO3; flavin containing monooxygenase 3
1.61E-20	NUF2; kinetochore protein Nuf2 isoform X1
2.69E-34	RGS8; regulator of G-protein signaling 8 isoform X2
1.21E-07	RGSL1; regulator of G-protein signaling protein-like isoform X1
7.47E-05	GLUL; glutamine synthetase
0.000133	SOAT1; sterol O-acyltransferase 1 isoform X4
6.74E-14	ABL2; tyrosine-protein kinase ABL2 isoform X2
1.30E-31	TOR3A; torsin-3A
2.16E-11	KIAA0040; uncharacterized protein KIAA0040 homolog
1.98E-06	RABGAP1L; rab GTPase-activating protein 1-like isoform X8
7.88E-05	SERPINC1; antithrombin-III precursor
2.62E-09	PLPPR5; phospholipid phosphatase-related protein type 5 isoform X5
0.000443	ZNF644; zinc finger protein 644 isoform X3
#######	CLCA1; calcium-activated chloride channel regulator 1-like
8.10E-19	SSX2IP; afadin- and alpha-actinin-binding protein isoform X3
1.27E-18	VTG2; vitellogenin-2 precursor
7.06E-11	LOC121111362; uncharacterized protein LOC121111362 isoform X1
1.54E-24	VTG3; vitellogenin-3
1.29E-08	SPATA1; spermatogenesis-associated protein 1 isoform X3
6.95E-22	VTG1; vitellogenin-1 precursor
3.25E-12	PTGFR; prostaglandin F2-alpha receptor isoform X1
7.21E-05	ELOVL1; elongation of very long chain fatty acids protein 1
2.32E-10	KIF2C; kinesin-like protein KIF2C isoform X10

7.67E-24 RBP; riboflavin-binding protein precursor
6.86E-08 RAD54L; DNA repair and recombination protein RAD54-like
9.73E-08 CYP4A22; cytochrome P450 4B1
1.04E-21 CYP4B7; cytochrome P450 4B7
2.25E-08 ORC1; origin recognition complex subunit 1
1.88E-07 PODN; podocan isoform X3
5.14E-23 DHCR24; delta(24)-sterol reductase
9.30E-06 DAB1; disabled homolog 1
9.65E-08 KANK4; KN motif and ankyrin repeat domain-containing protein 4 isoform X1
2.58E-22 ANGPTL3; angiopoietin-related protein 3 precursor
2.64E-07 DNAJC6; putative tyrosine-protein phosphatase auxilin
1.31E-24 NEGR1; neuronal growth regulator 1 isoform X2
0.000235 CRYZ; quinone oxidoreductase isoform X1
4.76E-11 NEU2; sialidase-2 isoform X3
4.61E-07 KLHL24; kelch-like protein 24 isoform X1
4.25E-12 EHHADH; peroxisomal bifunctional enzyme isoform X2
6.11E-15 DNAJB11; dnaJ homolog subfamily B member 11
0.000171 SLCO2A1; solute carrier organic anion transporter family member 2A1 isoform X1
1.75E-08 TF; ovotransferrin precursor
0.000182 MAB21L4; protein mab-21-like 4
1.34E-06 OTOS; otospiralin precursor
1.48E-27 SGPP2; sphingosine-1-phosphate phosphatase 2
2.10E-16 MOGAT1; 2-acylglycerol O-acyltransferase 1
1.46E-18 PCOLCE2; procollagen C-endopeptidase enhancer 2 isoform X1
2.10E-08 PLOD2; procollagen-lysine,2-oxoglutarate 5-dioxygenase 2 isoform X2
4.06E-08 LOC770705; uncharacterized protein LOC770705
2.58E-25 LOC121113436; uncharacterized protein LOC121113436
2.03E-13 BDH1B; D-beta-hydroxybutyrate dehydrogenase, mitochondrial isoform X1
1.55E-08 HRASLS; phospholipase A and acyltransferase 1
2.22E-12 RTP2; receptor-transporting protein 2-like
1.11E-10 PDCD1; programmed cell death protein 1
1.93E-05 C2orf72; uncharacterized protein C2orf72 homolog
1.07E-21 ALPI; intestinal-type alkaline phosphatase
2.71E-10 CLCN2; chloride channel protein 2 isoform X2
1.07E-11 HRG; histidine-rich glycoprotein isoform X1
2.59E-18 ECT2; protein ECT2 isoform X3
3.75E-18 SERPINI1; neuroserpin precursor
1.42E-22 LOC107054133; SLIT and NTRK-like protein 3

7.99E-17	SMC4; structural maintenance of chromosomes protein 4
3.66E-10	IFT80; intraflagellar transport protein 80 homolog
1.45E-10	MLF1; myeloid leukemia factor 1 isoform X1
3.37E-38	PTX3; pentraxin-related protein PTX3 precursor
1.22E-08	C3orf33; protein C3orf33 homolog
5.67E-11	SUCNR1; succinate receptor 1 isoform X3
2.92E-10	HPS3; Hermansky-Pudlak syndrome 3 protein
7.82E-11	CP; ceruloplasmin isoform X2
4.91E-22	TUBA3E; tubulin alpha-3 chain
7.63E-06	SLC28A2; sodium/nucleoside cotransporter 1
1.27E-51	DUOX1; dual oxidase 2
2.06E-18	KIAA0101; PCNA-associated factor
1.29E-09	PATL2; protein PAT1 homolog 2 isoform X1
4.63E-13	CYP1A2; cytochrome P450 1A5 isoform X1
1.80E-11	CYP1A1; cytochrome P450 1A4
4.03E-06	CHRNA3; neuronal acetylcholine receptor subunit alpha-3 precursor
8.65E-42	TRPM1; transient receptor potential cation channel subfamily M member 1 isoform X5
9.41E-11	CCNB1; G2/mitotic-specific cyclin-B2
1.04E-38	LIPC; hepatic triacylglycerol lipase isoform X1
8.56E-12	SCG3; secretogranin-3 isoform 2 precursor
2.55E-35	KIAA1024; major intrinsically disordered Notch2-binding receptor 1 isoform X1
9.13E-11	MFGE8; lactadherin isoform 1 precursor
1.25E-25	PGPEP1L; pyroglutamyl-peptidase 1-like protein isoform X1
5.68E-15	ADAMTS17; A disintegrin and metalloproteinase with thrombospondin motifs 17 isoform X3
3.03E-16	ALDH1A3; aldehyde dehydrogenase family 1 member A3
5.77E-08	PCSK6; proprotein convertase subtilisin/kexin type 6 isoform X1
6.77E-13	CORO2B; coronin-2B isoform X3
1.30E-05	PAQR5; membrane progestin receptor gamma
2.02E-34	KIF23; kinesin-like protein KIF23 isoform X18
3.04E-10	TMED3; transmembrane emp24 domain-containing protein 3 isoform 1 precursor
#######	CETP; cholesteryl ester transfer protein precursor
1.33E-08	CX3CL1; fractalkine precursor
4.32E-07	EXOC3L1; exocyst complex component 3-like protein
2.44E-33	LCAT; phosphatidylcholine-sterol acyltransferase precursor
9.30E-05	LOC415662; C-factor-like isoform X3
2.63E-23	LOC101747680; C-factor-like
2.39E-05	LOC415664; uncharacterized oxidoreductase-like isoform X1
1.51E-08	LOC100857820; C-factor

2.15E-06 LOC101748539; uncharacterized oxidoreductase C663.09c-like
4.53E-05 PDPR; pyruvate dehydrogenase phosphatase regulatory subunit, mitochondrial
4.61E-08 MT4; metallothionein
8.32E-11 MT3; metallothionein-3
3.35E-05 GALR1L; galanin receptor type 1-like isoform X2
6.67E-09 SLC6A2; sodium-dependent noradrenaline transporter
5.77E-08 TOX3; TOX high mobility group box family member 3 isoform 1
2.89E-07 CDCA9; borealin-2
9.46E-06 RGS9BP; regulator of G-protein signaling 9-binding protein
3.32E-15 CES1L2; fatty acyl-CoA hydrolase precursor, medium chain
4.60E-11 CES1L1; fatty acyl-CoA hydrolase precursor, medium chain isoform X1
2.97E-15 TERB1; telomere repeats-binding bouquet formation protein 1 isoform X3
1.59E-06 BEAN1; protein BEAN1
3.98E-07 DYNLRB2; dynein light chain roadblock-type 2
7.65E-07 CMC2; COX assembly mitochondrial protein 2 homolog isoform X2
5.35E-19 CENPN; centromere protein N
2.04E-09 LOC776594; uncharacterized protein LOC776594
2.80E-09 GINS2; DNA replication complex GINS protein PSF2 isoform X1
1.26E-05 SLC7A5; large neutral amino acids transporter small subunit 1
1.34E-15 CA5A; carbonic anhydrase 5A, mitochondrial isoform X2
9.43E-10 ZFPM1; zinc finger protein ZFPM1 isoform X5
1.30E-08 CIDEC; cell death activator CIDE-3 isoform X1
4.32E-07 MVD; diphosphomevalonate decarboxylase
2.33E-15 CDT1; DNA replication factor Cdt1 isoform X1
8.75E-14 SPIRE2; protein spire homolog 2 isoform X1
8.54E-16 CHTF8; chromosome transmission fidelity protein 8 homolog
5.10E-05 NQO1; NAD(P)H dehydrogenase [quinone] 1 isoform 1
1.25E-39 PMFBP1; polyamine-modulated factor 1-binding protein 1 isoform X6
1.48E-71 LOC121111704; uncharacterized protein LOC121111704
1.15E-47 LOC121106611; uncharacterized protein LOC121106611 isoform X2
8.30E-12 NT5DC2; 5'-nucleotidase domain-containing protein 2 isoform X1
3.94E-07 SMIM4; small integral membrane protein 4
8.62E-05 ITIH3; inter-alpha-trypsin inhibitor heavy chain H3 isoform X3
0.005946 MUSTN1; musculoskeletal embryonic nuclear protein 1
0.00024 LOC121106612; store-operated calcium entry regulator STIMATE-like isoform X1
5.71E-05 LOC693265; CMD-4
1.81E-10 CACNA2D2; voltage-dependent calcium channel subunit alpha-2/delta-2 isoform X3
7.99E-13 MANF; mesencephalic astrocyte-derived neurotrophic factor isoform X2

1.06E-08	LOC121106623; guanylate-binding protein 1-like
0.003944	LOC121106627; guanylate-binding protein 1-like
4.05E-07	GMPPB; mannose-1-phosphate guanyltransferase beta
1.40E-20	MST1R; macrophage-stimulating protein receptor isoform X1
1.53E-06	USP4; ubiquitin carboxyl-terminal hydrolase 4
0.000189	ALAS1; 5-aminolevulinate synthase, nonspecific, mitochondrial precursor
0.000203	SEMA3F; semaphorin-3F precursor
1.53E-11	OASL; 59 kDa 2'-5'-oligoadenylate synthase-like protein
3.05E-07	OGN; mimecan isoform X1
5.07E-08	ATP2B2; plasma membrane calcium-transporting ATPase 2 isoform X7
0.000148	SUSD3; sushi domain-containing protein 3
5.30E-10	DNASE1L3; deoxyribonuclease gamma precursor
1.43E-08	ABHD6; monoacylglycerol lipase ABHD6 isoform X2
6.73E-06	IP6K2; inositol hexakisphosphate kinase 2 isoform X1
1.04E-05	LOC770936; SRSF protein kinase 3 isoform X1
0.000138	PRRT3; proline-rich transmembrane protein 3 isoform X1
0.000241	P4HTM; transmembrane prolyl 4-hydroxylase
2.69E-16	GPR27; probable G-protein coupled receptor 27
1.00E-41	PROK2; prokineticin-2
4.76E-10	BHLHE40; class E basic helix-loop-helix protein 40
0.003724	GHRL; appetite-regulating hormone preproprotein
3.87E-11	HBEGF; proheparin-binding EGF-like growth factor precursor
#######	TENM2; teneurin-2 isoform 2
6.73E-16	HMMR; hyaluronan mediated motility receptor isoform X1
1.93E-25	PTTG2; securin isoform 1
0.00027	AFAP1L1; actin filament-associated protein 1-like 1 isoform X5
1.30E-07	DUSP1; dual specificity protein phosphatase 1
7.90E-06	STC2; stanniocalcin-2
1.76E-08	MXD3; lateral signaling target protein 2 homolog isoform X3
7.87E-21	LOC101751218; max dimerization protein 3 isoform X3
9.28E-05	LOC416263; LON peptidase N-terminal domain and RING finger protein 1 isoform X3
3.51E-06	GRIA1; glutamate receptor 1 precursor
4.61E-08	_
4.64E-07	TRPC7; short transient receptor potential channel 7 isoform X1
3.27E-10	LOC101749540; small integral membrane protein 32
5.22E-21	LECT2; myeloid protein 1 precursor
0.000153	SLC22A5; solute carrier family 22 member 5
2.50E-09	FGF1; fibroblast growth factor 1 isoform X1

2.13E-23 EGR1; early growth response protein 1
4.49E-35 BHLHA15; class A basic helix-loop-helix protein 15
9.97E-05 BAIAP2L1; brain-specific angiogenesis inhibitor 1-associated protein 2-like protein 1
1.67E-18 NPTX2; neuronal pentraxin-2 isoform X1
1.63E-06 GAA2; lysosomal alpha-glucosidase isoform X2
2.10E-05 CYP3A5; cytochrome P450 family 3 subfamily A member 5
9.25E-11 RNF216; E3 ubiquitin-protein ligase RNF216
1.99E-36 LOC101747873; urotensin-2 receptor
1.10E-41 CACNA1H; voltage-dependent T-type calcium channel subunit alpha-1H isoform X2
1.17E-06 SOX8; transcription factor SOX-8
1.75E-06 MSLN; mesothelin
4.55E-05 FAHD1; acylpyruvase FAHD1, mitochondrial
1.21E-26 PLK1; serine/threonine-protein kinase PLK1
7.47E-14 RMI2; recQ-mediated genome instability protein 2
2.12E-10 RHBDF1; inactive rhomboid protein 1 isoform X2
2.15E-28 HBAD; hemoglobin subunit alpha-D
1.10E-24 HBA1; hemoglobin subunit alpha-A
4.65E-40 PDIA2; protein disulfide-isomerase A2
6.43E-66 LOC416655; protein PERCC1 isoform X1
0.000145 C16orf96; uncharacterized protein C16orf96 homolog isoform X3
2.29E-05 ZKSCAN7; zinc finger protein 436 isoform X3
8.72E-08 NME4; nucleoside diphosphate kinase, mitochondrial
4.52E-06 DECR2; peroxisomal 2,4-dienoyl-CoA reductase [(3E)-enoyl-CoA-producing] isoform X1
5.75E-06 MCRIP2; MAPK regulated corepressor interacting protein 2 isoform X1
5.80E-08 LOC121106812; cyclin-F-like isoform X1
2.68E-23 LOC121106758; uncharacterized protein LOC121106758
7.52E-12 SDF2L1; stromal cell-derived factor 2-like protein 1
1.25E-25 CDC45; cell division control protein 45 homolog isoform X2
2.03E-08 CLDN5; claudin-5
0.000241 COMT; catechol O-methyltransferase
6.75E-08 RIMBP2; RIMS-binding protein 2 isoform X14
3.01E-16 AACS; acetoacetyl-CoA synthetase
1.26E-20 RAD9B; cell cycle checkpoint control protein RAD9B isoform X2
1.61E-09 PPTC7; protein phosphatase PTC7 homolog
3.38E-08 UNG; uracil-DNA glycosylase
3.08E-65 ACACB; acetyl-CoA carboxylase 2 isoform X5
4.53E-06 FAM222A; protein FAM222A isoform X3
2.30E-11 CRYBB3; beta-crystallin B3

8.10E-05 LOC101750445; WW domain-binding protein 11
2.62E-13 IGLL1; Ig lambda chain V-1 region isoform X34
1.01E-05 VPS29L; vacuolar protein sorting 29 homolog
9.97E-05 CHCHD10; coiled-coil-helix-coiled-coil-helix domain-containing protein 10, mitochondrial
5.08E-17 MMP11; stromelysin-3
3.74E-07 DERL3; derlin-3 isoform X2
2.45E-05 SLC2A11; solute carrier family 2, facilitated glucose transporter member 11
2.72E-20 SLC2A11L1; solute carrier family 2, facilitated glucose transporter member 11-like
2.78E-20 LOC769554; solute carrier family 2, facilitated glucose transporter member 11
1.18E-06 LOC101751878; solute carrier family 2, facilitated glucose transporter member 11
8.17E-10 GSTT1L; glutathione S-transferase theta-1 isoform X1
7.14E-09 TBX6; T-box-containing protein TBX6L isoform X1
2.53E-17 LOC416959; melanotransferrin isoform X2
1.56E-14 PISD; phosphatidylserine decarboxylase proenzyme, mitochondrial isoform X1
0.000341 ACADS; short-chain specific acyl-CoA dehydrogenase, mitochondrial isoform X1
7.56E-16 LOC100858984; dynein light chain 2, cytoplasmic
3.70E-14 PRODH; proline dehydrogenase 1, mitochondrial isoform X1
1.19E-53 SLC35E4; solute carrier family 35 member E4
1.51E-19 TCN2; transcobalamin-2
7.11E-11 HORMAD2; HORMA domain-containing protein 2 isoform X9
2.71E-16 LOC417013; acyl-CoA dehydrogenase family member 11 isoform X2
5.26E-05 LOC107050670; rho GTPase-activating protein 33-like
1.77E-13 LOC121106953; uncharacterized protein LOC121106953 isoform X1
0.000202 LOC107049794; uncharacterized protein LOC107049794 isoform X3
3.58E-21 MHCY2; LOW QUALITY PROTEIN: class I histocompatibility antigen, F10 alpha chain
1.26E-20 MHCY14; major histocompatibility complex-Y, class I heavy chain, 14 precursor
8.50E-11 LOC121106942; C-type lectin domain family 2 member I-like isoform X5
1.83E-47 LOC121106923; LOW QUALITY PROTEIN: class I histocompatibility antigen, F10 alpha chain-like
1.91E-26 LENG9L7; leukocyte receptor cluster member 9-like, MHC-Y region, 7
2.92E-17 MHCY7; major histocompatibility complex-Y, class I heavy chain, 7 precursor
4.79E-28 ZNFY1; zinc finger, MHC-Y region, 1
4.88E-18 YLEC17; c-type lectin-like protein, MHC-Y region, 17
6.17E-11 LOC121106436; class I histocompatibility antigen, F10 alpha chain-like isoform X1
7.12E-13 LOC121106918; class I histocompatibility antigen, F10 alpha chain-like isoform X2
1.74E-08 LOC121106502; C-type lectin domain family 2 member I-like isoform X5
7.55E-18 LOC121106943; C-type lectin domain family 2 member E-like isoform X1
4.14E-16 LOC121106928; class I histocompatibility antigen, F10 alpha chain-like isoform X1
0.000326 MHCY8; major histocompatibility complex Y, class I heavy chain 8 isoform X2

1.65E-12	MHCY32; major histocompatibility complex Y, class I heavy chain 32
1.92E-06	MOGL; erythroid membrane-associated protein-like
1.69E-15	LOC121106920; uncharacterized protein LOC121106920 isoform X18
3.26E-15	BTN3A3L1; erythroid membrane-associated protein-like isoform X4
2.92E-11	KIFC1; kinesin-like protein KIFC1 isoform X2
7.31E-50	IL4I1; L-amino-acid oxidase precursor
0.001677	TRIM7.1; tripartite motif-containing protein 7 isoform X5
5.34E-08	TRIM27.1; tripartite motif-containing 27 isoform X1
2.10E-11	BF2; Major histocompatibility complex class I antigen BF2 precursor
1.39E-18	LOC121106935; collagen alpha-1(I) chain-like
1.92E-06	LOC107049645; uncharacterized protein LOC107049645 isoform X2
3.87E-06	FCN2; ficolin-2 isoform X12
1.93E-05	MAN1B1; endoplasmic reticulum mannosyl-oligosaccharide 1,2-alpha-mannosidase isoform X2
#######	EXFABP; extracellular fatty acid-binding protein precursor
1.18E-57	LCN15; lipocalin-15
7.73E-11	PTGDS; prostaglandin-H2 D-isomerase precursor
4.71E-05	BSPRY; B box and SPRY domain-containing protein
1.20E-13	STPG3; protein STPG3 isoform X1
2.80E-42	NSMF; NMDA receptor synaptonuclear signaling and neuronal migration factor isoform X2
1.70E-07	GSL; glutamine synthetase
2.13E-10	BRINP1; BMP/retinoic acid-inducible neural-specific protein 1 precursor
4.59E-10	ORM1; orosomucoid 1 (ovoglycoprotein) precursor
1.22E-08	LOC107052201; alpha-1-acid glycoprotein
7.69E-10	SLC25A25; calcium-binding mitochondrial carrier protein SCaMC-2 isoform X4
6.33E-05	ST6GALNAC6; alpha-N-acetylgalactosaminide alpha-2,6-sialyltransferase 6
1.06E-20	FPGS; folylpolyglutamate synthase, mitochondrial isoform X9
5.96E-05	DNM1; dynamin-1 isoform X8
8.09E-10	LOC121107102; uncharacterized protein LOC121107102
1.97E-07	ASS1; argininosuccinate synthase
1.07E-16	NTNG2; netrin-G2 isoform X1
6.20E-11	ADAMTSL2; ADAMTS-like protein 2 isoform X7
4.91E-18	FAM69B; divergent protein kinase domain 1B isoform X1
2.74E-42	AGPAT2; 1-acyl-sn-glycerol-3-phosphate acyltransferase beta isoform X2
9.92E-11	EGFL7; epidermal growth factor-like protein 7 isoform X2
3.23E-09	LOC417113; carboxyl-terminal PDZ ligand of neuronal nitric oxide synthase protein
2.89E-18	HSPA5; endoplasmic reticulum chaperone BiP isoform X1
1.48E-09	MVB12B; multivesicular body subunit 12B isoform X2
7.73E-05	HS3ST3B1L; heparan sulfate glucosamine 3-O-sulfotransferase 3B1

2.04E-13 HS3ST3A1; heparan sulfate glucosamine 3-O-sulfotransferase 3A1
1.43E-07 FN3K; fructosamine-3-kinase isoform X2
2.25E-10 TEN1; CST complex subunit TEN1 isoform X2
4.53E-14 ACOX1; peroxisomal acyl-coenzyme A oxidase 1 isoform X3
7.01E-09 FBF1; fas-binding factor 1 homolog isoform X12
4.34E-06 MRPL38; 39S ribosomal protein L38, mitochondrial
0.000189 TRIM65; tripartite motif-containing protein 65
6.97E-08 UNC13D; protein unc-13 homolog D isoform X7
8.17E-10 SMIM5; small integral membrane protein 5
0.000231 CCDC57; coiled-coil domain-containing protein 57 isoform X9
9.42E-13 FASN; fatty acid synthase
3.01E-09 PCTP; phosphatidylcholine transfer protein isoform X2
7.49E-20 KPNA2; importin subunit alpha-1
3.91E-11 CACNG4; voltage-dependent calcium channel gamma-4 subunit
3.10E-06 RGS9; regulator of G-protein signaling 9 isoform X1
2.99E-06 ABCA8; ATP-binding cassette sub-family A member 10
3.30E-05 CDC42EP4; cdc42 effector protein 4 isoform X1
1.29E-06 CCDC40; coiled-coil domain-containing protein 40
6.86E-19 CBX2; chromobox protein homolog 2
2.25E-32 RBFOX3; RNA binding protein fox-1 homolog 3 isoform X8
1.27E-07 ENPP7; ectonucleotide pyrophosphatase/phosphodiesterase family member 7
3.09E-15 TIMP2; metalloproteinase inhibitor 2 precursor
2.52E-31 SOCS3; suppressor of cytokine signaling 3
1.26E-22 TK1; thymidine kinase, cytosolic
1.35E-07 P4HB; protein disulfide-isomerase precursor
3.80E-22 GRIN2C; glutamate receptor ionotropic, NMDA 2C isoform X3
1.11E-06 HN1; jupiter microtubule associated homolog 1 isoform 3
2.53E-16 SLC16A5; monocarboxylate transporter 6
2.68E-12 GATSL2; cytosolic arginine sensor for mTORC1 subunit 2 isoform X7
0.000318 SPNS2; spinster homolog 2
1.04E-21 MIS12; protein MIS12 homolog
7.00E-17 MMP28; matrix metalloproteinase-28 isoform X1
4.58E-11 LOC121106433; C-C motif chemokine 3-like
6.27E-07 SERPINF1; pigment epithelium-derived factor isoform X1
2.80E-10 SRR; serine racemase isoform X7
1.73E-07 TMEM199; transmembrane protein 199
3.04E-10 SEBOX; homeobox protein SEBOX isoform X1
6.58E-10 SARM1; NAD(+) hydrolase SARM1

8.38E-60	SLC13A2; solute carrier family 13 member 2
0.000101	DHRS13; dehydrogenase/reductase SDR family member 13 isoform X1
2.54E-06	PIPOX; peroxisomal sarcosine oxidase isoform X1
5.65E-07	MED13; mediator of RNA polymerase II transcription subunit 13 isoform X3
8.25E-44	CA4; carbonic anhydrase 4
8.77E-08	ACACA; acetyl-CoA carboxylase isoform X1
1.36E-07	EVI2A; protein EVI2A precursor
7.90E-06	CLUH; clustered mitochondria protein homolog isoform X2
9.80E-07	FAM64A; uncharacterized protein FAM64A isoform X5
8.05E-15	FBXO39; F-box only protein 39 isoform X2
9.47E-09	TEKT1; tektin-1 isoform X2
9.45E-05	ASIP; agouti-signaling protein precursor
1.48E-09	ACSS2; acetyl-coenzyme A synthetase, cytoplasmic isoform X8
1.09E-07	FAM83D; protein FAM83D-B
2.13E-08	RPN2; dolichyl-diphosphooligosaccharide--protein glycosyltransferase subunit 2 precursor
#######	RBPJL; recombining binding protein suppressor of hairless-like protein isoform X3
6.32E-53	MATN4; matrilin-4
#######	PI3; elafin
1.25E-07	TTPAL; alpha-tocopherol transfer protein-like isoform X1
5.09E-22	GDAP1L1; ganglioside-induced differentiation-associated protein 1-like 1
1.81E-15	EPB42; protein 4.2
3.82E-17	GATA5; transcription factor GATA-5 isoform X1
8.17E-29	TNFRSF6B; tumor necrosis factor receptor superfamily member 6B isoform X1
8.02E-05	SDCBP2; syndecan binding protein 2
6.89E-14	TPX2; targeting protein for Xklp2 isoform X4
2.26E-10	LOC771972; uncharacterized protein LOC771972 isoform X1
4.34E-09	LOC100859272; WAP four-disulfide core domain protein 2 isoform X1
2.68E-31	WFDC2; WAP four-disulfide core domain protein 3 isoform X4
8.22E-28	WFDC8; balbiani ring protein 3 isoform X2
3.23E-23	SPINT4; kunitz-type protease inhibitor 4
8.08E-10	LOC771994; protein qua-1
2.80E-42	UBE2U; ubiquitin-conjugating enzyme E2 C
0.000917	C20orf85; uncharacterized protein C20orf85 homolog isoform X1
7.33E-12	PCK1; phosphoenolpyruvate carboxykinase, cytosolic [GTP]
0.000146	RBM38; RNA-binding protein 38
0.002855	UTS2; urotensin-2 precursor
0.000428	TNFRSF25; tumor necrosis factor receptor superfamily member 25
8.73E-44	ESPN; espin isoform X6

0.000905 ACOT7; cytosolic acyl coenzyme A thioester hydrolase isoform X1
1.06E-05 MIB2; E3 ubiquitin-protein ligase MIB2
1.22E-41 LOC419409; Golgi integral membrane protein 4 isoform X1
3.66E-27 LOC771069; C-factor isoform X2
0.000348 SLC2A5; solute carrier family 2, facilitated glucose transporter member 5
1.96E-06 CENPS; centromere protein S
2.92E-05 SLC25A34; solute carrier family 25 member 34
1.37E-40 RNF186; E3 ubiquitin-protein ligase RNF186
1.57E-06 CAMK2N1; calcium/calmodulin-dependent protein kinase II inhibitor 1
1.77E-15 CELA2A; chymotrypsin-like elastase family member 2A precursor
6.03E-09 MTHFR; methylenetetrahydrofolate reductase
1.31E-29 DRAXIN; draxin isoform X1
9.98E-09 ECE1; endothelin-converting enzyme 1 isoform X4
2.62E-08 LOC112530142; uncharacterized protein LOC112530142
2.50E-15 DPYSL2; dihydropyrimidinase-related protein 2 isoform X2
0.000519 GNRH1; progonadoliberin-1 precursor
4.08E-07 DMTN; dematin isoform X2
1.17E-06 LOC101749127; uncharacterized protein LOC101749127 isoform X1
1.02E-24 ADAM32L2; disintegrin and metalloproteinase domain-containing protein 32
8.15E-59 IDO2; indoleamine 2,3-dioxygenase 2 isoform X2
1.17E-14 ANK1; ankyrin-1 isoform X13
2.33E-11 NCAPH; condensin complex subunit 2 isoform X2
7.87E-07 ADD2; beta-adducin
9.32E-10 SLC20A1; sodium-dependent phosphate transporter 1
9.87E-25 CASP14; caspase-14
1.61E-13 RETSAT; all-trans-retinol 13,14-reductase
8.84E-60 FABP3; fatty acid-binding protein, heart
4.18E-09 LOC121107469; collagen alpha-1(I) chain-like
8.52E-06 NR0B2; nuclear receptor subfamily 0 group B member 2
1.11E-06 TRNP1; TMF-regulated nuclear protein 1 isoform X1
8.35E-06 TCEA3; transcription elongation factor A protein 3
3.54E-21 LOC101749201; peptide methionine sulfoxide reductase MsrA isoform X3
4.86E-05 RHCE; blood group Rh(CE) polypeptide
3.32E-33 STMN1; stathmin isoform X1
2.80E-20 PAQR7; membrane progestin receptor alpha isoform X1
0.001019 DNALI1; axonemal dynein light intermediate polypeptide 1
1.22E-33 IFI27L2; interferon alpha-inducible protein 27, mitochondrial
5.86E-11 FAM167B; protein FAM167B isoform X2

2.00E-32 MYCL; protein L-Myc
2.28E-05 MFSD2A; sodium-dependent lysophosphatidylcholine symporter 1
2.74E-09 GALE; UDP-glucose 4-epimerase isoform X1
8.77E-13 HMGCL; hydroxymethylglutaryl-CoA lyase, mitochondrial isoform 4
2.26E-15 GRHL3; grainyhead-like protein 3 homolog isoform X4
6.31E-28 IL22RA1; interleukin-22 receptor subunit alpha-1 precursor
2.20E-06 SPA17; sperm surface protein Sp17
8.11E-06 TMEM45L; transmembrane protein 45B isoform X2
9.01E-15 NCAPD3; condensin-2 complex subunit D3 isoform X3
1.22E-08 THY1; thy-1 membrane glycoprotein isoform X1
2.01E-08 USP2; ubiquitin carboxyl-terminal hydrolase 2 isoform X3
3.15E-05 ZBTB32; zinc finger and BTB domain-containing protein 16
2.39E-05 ZPR1; zinc finger protein ZPR1 isoform X2
1.86E-06 APOA5; apolipoprotein A-V
1.09E-05 APOC3; apolipoprotein C-III precursor
2.39E-11 APOA1; apolipoprotein A-I isoform X1
4.13E-08 TAGLN; transgelin
2.81E-21 FXYD2; sodium/potassium-transporting ATPase subunit gamma
9.47E-05 SCN4B; sodium channel subunit beta-4 isoform X3
3.64E-22 LOC101748511; uncharacterized protein LOC101748511 isoform X4
1.19E-16 HYOU1; hypoxia up-regulated protein 1 isoform X1
0.000179 BGLAP; osteocalcin preproprotein
3.68E-27 LOC121107551; sperm-associated antigen 4 protein-like isoform X2
1.65E-09 LOC101747704; sperm-associated antigen 4 protein-like isoform X3
3.76E-26 S100A12; protein MRP-126
3.00E-08 S100A6; protein S100-A6
2.21E-15 CKS1B; cyclin-dependent kinases regulatory subunit 1
1.06E-21 FDPS; farnesyl pyrophosphate synthase
1.67E-07 SYT11; synaptotagmin-11 isoform X1
0.000626 GOLPH3L; Golgi phosphoprotein 3-like
0.000159 CIART; circadian-associated transcriptional repressor isoform X1
1.00E-20 LOC121107589; uncharacterized protein LOC121107589 isoform X4
4.49E-06 PCP4L1; Purkinje cell protein 4 like 1
2.00E-05 NR1I3; nuclear receptor subfamily 1 group I member 3
4.67E-07 APOA2; apolipoprotein A-II isoform X1
2.30E-10 TMOD4; tropomodulin-4
0.00039 LOC121107543; acyl-coenzyme A thioesterase THEM4-like isoform X2
9.52E-13 LOC121106470; deaminated glutathione amidase-like isoform X7

3.25E-16	TULP1; tubby-related protein 1 isoform X1
3.18E-25	ELF3; ETS-related transcription factor Elf-3
8.75E-14	CSRP1; cysteine and glycine-rich protein 1
1.40E-11	UBE2T; ubiquitin-conjugating enzyme E2 T isoform X2
7.39E-09	SHISA4; protein shisa-4
8.86E-15	LOC107050775; epidermal growth factor receptor kinase substrate 8-like protein 3 isoform X1
#######	SLC26A9; solute carrier family 26 member 9
5.14E-40	C1orf186; regulator of hemoglobinization and erythroid cell expansion protein
1.06E-22	FAM72A; protein FAM72A isoform X1
0.000222	IKBKE; inhibitor of nuclear factor kappa-B kinase subunit epsilon isoform X1
8.90E-09	CD55; complement decay-accelerating factor isoform X1
1.04E-14	LAMB3; laminin subunit beta-3
5.97E-12	G0S2; G0/G1 switch protein 2
4.07E-08	HSD11B1B; corticosteroid 11-beta-dehydrogenase isozyme 1 isoform X1
5.48E-06	GUCA1A; guanylyl cyclase-activating protein 1
8.56E-15	SLC16A1; monocarboxylate transporter 1
8.51E-07	SYCP1; synaptonemal complex protein 1 isoform X1
2.29E-09	TSHB; thyrotropin subunit beta isoform X1
1.84E-10	PACSIN1; protein kinase C and casein kinase substrate in neurons protein 1
3.42E-22	TSPO2; translocator protein 2
5.46E-05	PGC; gastricsin isoform X1
5.94E-23	CHIA-M31; acidic mammalian chitinase isoform X1
5.47E-14	CHIA; acidic mammalian chitinase precursor
1.33E-51	LOC768786; acidic mammalian chitinase isoform X1
9.14E-06	FMOD; fibromodulin precursor
1.91E-26	LOC107057545; uncharacterized protein LOC107057545 isoform X3
9.30E-15	LOC107049117; maestro heat-like repeat-containing protein family member 7 isoform X1
6.64E-11	VWA5A1; von Willebrand factor A domain-containing protein 5A isoform X8
0.000105	DBF4B; protein DBF4 homolog B isoform X3
5.72E-08	C1QL1; C1q-related factor isoform X1
1.11E-05	PLCD3; 1-phosphatidylinositol 4,5-bisphosphate phosphodiesterase delta-3 isoform X5
2.65E-24	ACE; angiotensin-converting enzyme precursor
2.29E-34	SLC4A1; band 3 anion transport protein isoform X3
2.30E-11	COL1A1; collagen alpha-1(I) chain isoform X1
6.93E-09	SLC35B1; solute carrier family 35 member B1
9.05E-11	C27H17ORF105; uncharacterized protein C17orf105 homolog
0.002125	MEOX1; homeobox protein MOX-1
0.000141	PHOSPHO1; phosphoethanolamine/phosphocholine phosphatase

5.63E-08 FBXO47; F-box only protein 47
1.10E-09 PLXDC1; plexin domain-containing protein 1 isoform X2
0.000134 CACNB1; LOW QUALITY PROTEIN: voltage-dependent L-type calcium channel subunit beta-1 isoform X1
6.09E-14 CSF3; myelomonocytic growth factor precursor
7.97E-12 RARA; retinoic acid receptor alpha
2.94E-47 TOP2A; DNA topoisomerase 2-alpha
3.73E-11 KRT10; keratin, type I cytoskeletal 13
5.01E-08 P3H4; endoplasmic reticulum protein SC65
1.32E-10 G6PC; glucose-6-phosphatase
1.07E-09 VAT1; synaptic vesicle membrane protein VAT-1 homolog
6.76E-05 RND2; rho-related GTP-binding protein RhoN
9.31E-25 BRCA1; breast cancer type 1 susceptibility protein isoform X5
1.86E-25 SLC1A6; excitatory amino acid transporter 4 isoform X2
2.02E-07 MPND; MPN domain-containing protein isoform X3
2.13E-05 STAP2; signal-transducing adaptor protein 2 isoform X2
3.72E-06 NMRK2; nicotinamide riboside kinase 2
7.98E-58 ACSBG2; long-chain-fatty-acid--CoA ligase ACSBG2 isoform X4
4.25E-07 MBD3; methyl-CpG-binding domain protein 3 isoform X2
8.09E-11 MEX3D; RNA-binding protein MEX3D
3.90E-07 LRG1; leucine-rich alpha-2-glycoprotein isoform X1
8.26E-08 CREB3L3; cyclic AMP-responsive element-binding protein 3-like protein 3 isoform X2
4.00E-08 MIDN; midnolin-A isoform X1
4.15E-09 GAMT; guanidinoacetate N-methyltransferase isoform X1
5.79E-06 PCSK4; proprotein convertase subtilisin/kexin type 4 isoform X3
1.63E-26 ADAMTSL5; ADAMTS-like protein 5 isoform X3
0.000959 LOC100859819; cocaine- and amphetamine-regulated transcript protein isoform X5
5.30E-07 USHBP1; Usher syndrome type-1C protein-binding protein 1 isoform X4
1.12E-08 LOC420107; uncharacterized protein LOC420107 isoform X1
2.34E-05 COMP; cartilage oligomeric matrix protein
2.12E-06 LOC107055358; mucin-7-like isoform X3
1.51E-53 LOC121107782; zinc finger protein 707-like isoform X1
4.91E-22 LOC121107774; zinc finger protein 34-like isoform X1
1.82E-14 LPAR2; lysophosphatidic acid receptor 2
1.98E-05 ACP5; tartrate-resistant acid phosphatase type 5 isoform X1
1.47E-15 CALR; calreticulin
2.37E-07 LOC107049412; microtubule-associated serine/threonine-protein kinase 1 isoform X1
9.82E-05 LOC101747860; mucin-19-like isoform X9
1.36E-09 LOC769841; sulfotransferase 2B1 isoform X2

7.52E-09 LOC101751912; butyrophilin subfamily 3 member A2-like isoform X8
1.26E-51 LOC107049467; uncharacterized protein LOC107049467 isoform X2
0.000102 SLC27A2; bile acyl-CoA synthetase
6.10E-13 LOC107050008; platelet glycoprotein VI isoform X1
1.16E-09 LOC107049793; V-set and transmembrane domain-containing protein 1-like isoform X2
0.000193 LOC121107868; V-set and transmembrane domain-containing protein 1-like isoform X4
4.18E-05 LOC107049945; platelet glycoprotein VI-like isoform X1
5.29E-24 LOC100859870; leukocyte immunoglobulin-like receptor subfamily A member 2
0.001261 LOC107050226; platelet glycoprotein VI-like isoform X3
2.10E-11 LOC121107808; leukocyte immunoglobulin-like receptor subfamily B member 1
0.000585 CHIR-A2; immunoglobulin-like receptor CHIR-A2 precursor
1.88E-06 CHIR-AB1; immunoglobulin-like receptor CHIR-AB1 precursor
0.000202 LOC112531171; immunoglobulin superfamily member 1-like isoform X1
1.52E-15 LOC107049866; osteoclast-associated immunoglobulin-like receptor
7.22E-08 LOC112529946; T-cell-interacting, activating receptor on myeloid cells protein 1-like
5.07E-17 LOC121107862; platelet glycoprotein VI-like
1.40E-06 LOC121107894; platelet glycoprotein VI-like isoform X1
6.40E-12 LOC121107814; LOW QUALITY PROTEIN: leukocyte immunoglobulin-like receptor subfamily A member 6
1.42E-11 LOC112531135; platelet glycoprotein VI-like
1.23E-37 LOC112531119; platelet glycoprotein VI-like isoform X5
2.62E-16 LOC121107865; LOW QUALITY PROTEIN: leukocyte immunoglobulin-like receptor subfamily A member 2
5.81E-07 LOC431060; immunoglobulin superfamily member 1-like isoform X3
7.59E-05 LOC112531163; T-cell-interacting, activating receptor on myeloid cells protein 1-like isoform X1
0.000593 LOC100857964; platelet glycoprotein VI-like
1.06E-22 LOC100857597; osteoclast-associated immunoglobulin-like receptor isoform X1
9.43E-16 LOC107050473; LOW QUALITY PROTEIN: platelet glycoprotein VI-like isoform X1
1.37E-09 CHIR-B3; immunoglobulin-like receptor CHIR-B3 isoform X1
4.84E-08 LOC121107818; platelet glycoprotein VI-like
7.35E-11 LOC112531212; leukocyte immunoglobulin-like receptor subfamily A member 2 isoform X4
1.95E-12 LOC121107822; leukocyte immunoglobulin-like receptor subfamily B member 3
2.19E-14 LOC112531339; leukocyte immunoglobulin-like receptor subfamily A member 2 isoform X3
4.16E-05 LOC121106510; V-set and transmembrane domain-containing protein 1-like isoform X1
5.23E-10 LOC112531100; leukocyte immunoglobulin-like receptor subfamily A member 2
0.000202 LOC107049195; killer cell immunoglobulin-like receptor 3DL3 isoform X1
6.61E-07 LOC121107882; T-cell-interacting, activating receptor on myeloid cells protein 1-like isoform X3
1.25E-30 LOC100859740; V-set and transmembrane domain-containing protein 1
4.41E-19 LOC107049819; uncharacterized protein LOC107049819
1.12E-50 LOC107056248; olfactory receptor 14J1-like

1.99E-12	FKBP11; peptidyl-prolyl cis-trans isomerase FKBP11
6.67E-07	METTL7A; methyltransferase-like protein 7A
2.40E-32	RACGAP1; rac GTPase-activating protein 1 isoform X3
5.82E-07	LOC107049666; integrin beta-7 isoform X2
3.84E-08	LOC107049862; uncharacterized protein LOC107049862
9.16E-17	APOF; apolipoprotein F
1.01E-06	TIMELESS; LOW QUALITY PROTEIN: protein timeless homolog
0.00021	ZBTB39; zinc finger and BTB domain-containing protein 39
1.46E-28	TAC3; tachykinin-3 isoform X2
1.84E-09	INHBE; inhibin beta E chain
1.04E-06	KRT7; keratin, type II cytoskeletal cochleal isoform X1
4.06E-06	POU6F1; LOW QUALITY PROTEIN: POU domain, class 6, transcription factor 1
1.34E-12	LOC101751319; uncharacterized protein LOC101751319
5.67E-30	LOC121106503; maestro heat-like repeat-containing protein family member 2A isoform X2
1.12E-10	ADPRHL; protein ADP-ribosylarginine hydrolase isoform X5
6.85E-15	LOC107051274; Ig gamma chain C region isoform X1
6.55E-15	LOC112531456; uncharacterized protein LOC112531456 isoform X1
3.03E-08	VKORC1; vitamin K epoxide reductase complex, subunit 1 precursor
2.29E-10	LOC121108153; cold shock protein CS66-like
0.000146	LOC107055390; mRNA decay activator protein ZFP36-like isoform X1
0.000562	LOC112530471; uncharacterized protein LOC112530471 isoform X1
3.30E-40	OTX5; cone-rod homeobox protein isoform X1
4.10E-33	LOC107050328; uncharacterized protein LOC107050328
1.13E-07	NKPD1; NTPase KAP family P-loop domain-containing protein 1
0.000109	LOC121108182; unconventional myosin-Vb-like isoform X2
4.54E-09	LOC426220; avidin isoform X1
5.84E-09	LOC121108212; uncharacterized protein LOC121108212
7.29E-47	ALPK2; alpha-protein kinase 2
7.41E-05	SMAD7; mothers against decapentaplegic homolog 7 isoform X1
2.55E-12	ENHO; adropin
3.28E-07	ARID3C; AT-rich interactive domain-containing protein 3C isoform X2
3.11E-09	LOC100857191; C-C motif chemokine 26
1.99E-36	RUSC2; iporin isoform X3
2.71E-06	NPR2; atrial natriuretic peptide receptor 2
1.15E-13	AVDL; avidin isoform X1
#######	AVD; avidin precursor
1.51E-13	CA9; carbonic anhydrase 9 isoform X2
9.76E-17	NPR3; atrial natriuretic peptide receptor 3 isoform X4

8.94E-16 CENPK; centromere protein K
2.15E-13 BHMT2; betaine--homocysteine S-methyltransferase 1
3.63E-08 DMGDH; dimethylglycine dehydrogenase, mitochondrial isoform X2
6.22E-11 S100Z; protein S100-Z
2.59E-11 HMGCR; 3-hydroxy-3-methylglutaryl-Coenzyme A reductase
1.43E-07 ANKRD31; ankyrin repeat domain-containing protein 31 isoform X8
3.07E-16 GLDC; glycine dehydrogenase (decarboxylating), mitochondrial precursor
5.28E-05 PLIN2; perilipin-2
3.13E-06 ALDH1A1; retinal dehydrogenase 1
1.86E-06 ANXA1; annexin A1 isoform X1
5.26E-14 FBP2; fructose-1,6-bisphosphatase isozyme 2
1.96E-06 CKS2; cyclin-dependent kinases regulatory subunit 2
4.90E-30 GADD45G; growth arrest and DNA damage-inducible protein GADD45 gamma
1.46E-24 SHB; SH2 domain-containing adapter protein B isoform X1
5.00E-09 MFSD7; solute carrier family 49 member A3
3.52E-62 LPL; lipoprotein lipase precursor
1.32E-20 PSD3; PH and SEC7 domain-containing protein 3 isoform X4
2.25E-08 LMNB1; lamin-B1
2.40E-09 RHOBTB3; rho-related BTB domain-containing protein 3 isoform X3
0.000301 RFESD; Rieske domain-containing protein
1.41E-24 LOC768418; zinc finger FYVE domain-containing protein 16-like
0.000177 TMEM246; post-GPI attachment to proteins factor 4
6.99E-15 ALDOB; fructose-bisphosphate aldolase B
2.12E-07 KIAA1958; uncharacterized protein KIAA1958 homolog isoform X1
1.02E-39 SMC2; structural maintenance of chromosomes protein 2
1.47E-46 MUSK; muscle, skeletal receptor tyrosine protein kinase isoform X4
5.72E-21 LPAR1; lysophosphatidic acid receptor 1 isoform X1
3.59E-09 SPINK4; serine protease inhibitor Kazal-type 4
5.87E-08 SEMA6A; semaphorin-6A isoform X3
1.02E-23 CDKN2A; ARF tumor suppressor
5.50E-26 CDKN2B; cyclin-dependent kinase 4 inhibitor B
8.04E-06 CCDC112; coiled-coil domain-containing protein 112 isoform X3
1.49E-07 LOC107050717; calumenin isoform X1
2.80E-06 LOC107050135; leukocyte immunoglobulin-like receptor subfamily B member 5
9.09E-06 LOC121108673; osteoclast-associated immunoglobulin-like receptor
1.08E-07 LOC107050652; putative killer cell immunoglobulin-like receptor-like protein KIR3DX1
6.96E-28 LOC121108719; uncharacterized protein LOC121108719 isoform X2
4.43E-10 CDIPT; CDP-diacylglycerol--inositol 3-phosphatidyltransferase isoform X1

0.003768 LOC121108733; protein spinster homolog 1-like isoform X2
9.29E-07 LOC121108734; E3 ubiquitin-protein ligase Hakai-like
1.93E-18 PKMYT1; membrane-associated tyrosine- and threonine-specific cdc2-inhibitory kinase isoform X2
1.60E-30 LOC121108741; vasodilator-stimulated phosphoprotein-like isoform X1
6.26E-18 LOC121108762; uncharacterized protein LOC121108762 isoform X1
7.77E-05 LOC121108855; uncharacterized protein LOC121108855 isoform X2
2.02E-42 LOC112530182; soluble scavenger receptor cysteine-rich domain-containing protein SSC5D-like
3.19E-08 LOC107050463; clumping factor A-like
0.000151 LOC112531297; uncharacterized protein LOC112531297 isoform X1
2.70E-08 LOC121108875; uncharacterized protein LOC121108875 isoform X6
2.53E-06 LOC121108901; putative protein CRIPAK
2.05E-05 HSD17B10; 3-hydroxyacyl-CoA dehydrogenase type-2 isoform X2
1.16E-09 PFKFB1; 6-phosphofructo-2-kinase/fructose-2, 6-bisphosphatase
5.93E-07 LOC112530174; mucin-22-like isoform X1
4.96E-30 LOC121108923; mental retardation GTPase activating protein homolog 4-like
0.001071 MHCY13; major histocompatibility complex-Y, class I heavy chain, 13 precursor
8.17E-14 LOC112530071; NF-kappa-B inhibitor delta-like isoform X1
