# Supplementary material for: Comparative analysis of liver transcriptome reveals adaptive responses to hypoxia environmental condition in Tibetan chicken
Source: Anim Biosci. 2023 Aug 23;37(1):28–38. doi: 10.5713/ab.23.0126 (PMC10766467; doi:10.5713/ab.23.0126)
Supplement: Supplementary file 6 [file ab-23-0126-Supplementary-Table-6.pdf]

Table S6

Geneid	HT1	HT2	HT3	HT4	HT5	LT1	LT2	LT3	LT4	LT5	logFC	type	pvalue
LOC12111	3.334	4.314	4.976	0.771	0.913	0.096	0.274	0.226	0.988	0.626	-2.69195	down	2.23E-29
LOC1211C	2.719	2.124	1.201	0.265	0.41	0.15	0.297	0.539	0.701	0.411	-1.67687	down	1.99E-14
LOC77646	21.883	20.546	22.844	2.642	1.25	0.756	0.507	0.815	5.924	0.306	-3.05671	down	3.62E-41
LOC11253	5.471	4.844	26.895	5.635	2.36	2.773	2.898	3.549	5.22	1.163	-1.53436	down	1.44E-11
LOC12111	0.514	0.62	0.011	0.033	0.014	0	0.023	1.397	1.625	1.948	2.061928	up	8.82E-16
CD69L	18.257	17.274	8	0.32	0.614	4.332	0.301	0.53	0.164	0.694	-2.88356	down	2.80E-38
SHANK3	1.733	1.677	3.229	3.177	3.394	0.625	0.796	1.069	0.705	0.863	-1.70156	down	1.24E-15
LOC1211C	1.419	1.132	2.508	2.285	2.131	0.342	0.539	0.696	0.488	0.637	-1.80819	down	1.85E-16
ARF5	0.644	0.692	1.245	0.68	0.898	2.761	1.061	1.322	0.613	3.536	1.15895	up	4.82E-07
NCAPH2	0.6	0.714	0.434	0.717	1.027	0.898	0.652	3.372	1.231	0.937	1.020685	up	2.37E-05
STRIP2	0.663	0.55	1.717	1.626	1.342	0.353	1.826	0.049	0.211	0.504	-1.00171	down	4.89E-05
PFKFB3	2.176	2.406	3.906	3.272	3.183	7.16	3.504	8.189	4.834	9.25	1.139976	up	4.41E-08
MCM10	0.111	0.091	0.251	0.099	0.2	0.161	0.364	1.185	0.579	0.439	1.85212	up	1.52E-14
ELAPOR2	2.989	3.226	1.334	0.728	1.061	34.144	15.397	26.309	13.417	26.939	3.636717	up	6.91E-57
CACNA2D	1.303	1.019	0.961	0.974	0.624	0.734	0.39	0.454	0.443	0.397	-1.01186	down	1.79E-06
FGL2	8.704	8.533	12.528	14.921	11.429	11.07	19.217	35.299	39.282	27.206	1.234814	up	6.72E-09
ORC5	0.592	0.753	0.547	0.66	0.955	2.015	0.875	1.89	1.149	1.46	1.074062	up	9.04E-07
GTSE1	0.172	0.113	0.199	0.437	0.096	0.53	0.174	2.903	0.803	0.973	2.398355	up	1.58E-20
ALG12	12.218	11.172	12.612	15.737	13.32	47.073	26.388	46.523	26.668	35.887	1.488313	up	1.48E-12
CRELD2	16.486	14.861	20.903	18.59	14.191	74.923	28.225	59.368	32.142	48.603	1.516389	up	3.63E-13
ARL8BL	0.035	0.228	1.109	0	0.25	0.034	0.179	0.045	0.036	0.085	-2.08304	down	2.09E-07
IQUB	0.993	0.449	2.583	0.444	0.465	0.255	0.474	0.477	0.165	0.37	-1.50017	down	1.22E-09
CPED1	3.776	3.847	5.473	5.571	5.853	1.521	1.165	1.083	1.431	0.724	-2.04839	down	3.66E-21
AMIGO2	1.022	1.06	1.593	1.993	2.793	0.79	0.548	0.895	0.558	0.525	-1.35006	down	7.66E-10
SRGAP1	6.908	6.068	5.45	1.341	3.581	0.74	0.775	1.088	1.184	0.714	-2.37369	down	5.37E-28
CTSEAL	1213.242	1230.546	222.805	875.565	99.982	1553.457	1492.751	1425.564	1720.938	1527.825	1.083913	up	9.79E-07
PPFIA2	1.5	1.318	2.114	2.201	1.631	0.427	0.888	1.033	1.108	0.514	-1.14146	down	4.38E-08
SOCS2	39.881	35.397	32.19	30.561	21.379	10.112	5.772	17.991	28.211	10.665	-1.13163	down	1.52E-08
IKBIP	2.156	1.726	2.719	2.608	2.517	5.472	2.39	8.184	5.082	6.307	1.225954	up	9.77E-09
GAS2L3	0.237	0.183	0.572	0.315	0.079	0.54	0.298	1.312	0.623	1.124	1.488091	up	2.75E-11
OC3	0.122	0.08	0.13	0.201	0.292	0.079	0.625	0	1.043	0.299	1.305144	up	2.57E-05
LOC1211C	0.85	0.86	0.923	1.207	0.238	2.021	0.528	2.828	2.459	1.454	1.186825	up	1.72E-05
LOC10085	0.424	0.324	0.282	0.349	0.799	1.438	2.356	0.554	1.523	1.908	1.8342	up	1.31E-11
HIST1H2B	0.184	0.241	0.366	0	0.378	1.069	0.079	0.601	0.472	0.15	1.017102	up	0.011138
EMP1	0.074	0.109	0.309	0.304	0.38	0.483	0.284	1.062	0.379	0.695	1.300018	up	2.60E-07
GUCY2C	0.047	0.025	0.142	0.054	0.319	0.191	0.121	1.032	0.183	0.331	1.653959	up	4.76E-11

GBE	0.033	0.079	2.065	1.056	0.533	2.201	0.245	4.395	2.399	4.155	1.829214	up	1.88E-18
LOC41797	0	0.266	23.459	10.244	2.214	12.585	0.08	40.275	14.903	54.853	1.761564	up	1.38E-17
CYP2D6	199.941	202.347	258.873	189.449	422.847	114.734	232.584	64.096	100.812	101.402	-1.05331	down	1.96E-06
MEI1	1.876	1.816	1.172	1.415	2.148	0.386	0.677	0.731	0.837	0.663	-1.35385	down	1.07E-10
CHADL	1.099	1.125	1.179	1.815	0.233	0.439	0.326	0.624	0.355	0.381	-1.35699	down	4.06E-10
ADSL	312.305	295.773	586.536	605.692	277.771	88.568	158.617	53.487	69.888	81.794	-2.19971	down	6.91E-24
NPTXR	2.066	1.895	0.571	0.682	0.203	14.788	7.929	13.096	11.466	17.441	3.577426	up	3.68E-54
DNAL4	2.011	1.833	1.659	2.273	2.822	4.834	3.237	4.761	4.55	5.375	1.102155	up	2.58E-07
FAM20CL	433.81	459.966	1.954	25.555	0.424	1459.967	951.748	967.731	775.842	1041.741	2.495297	up	2.79E-29
SUN2	18.332	17.316	9.806	6.471	7.075	27.906	38.772	35.555	35.705	50.139	1.672453	up	4.24E-15
SH3BP1	0.915	0.849	1.403	1.122	0.842	1.653	1.33	2.604	2.031	3.518	1.117162	up	2.64E-07
CARD10	1.29	1.45	2.944	2.441	0.998	0.176	0.869	0.759	0.233	0.371	-1.91947	down	7.45E-18
CSF2RB	0.238	0.393	0.341	0.579	0.196	0.295	0.26	1.592	1.075	1.371	1.392004	up	5.73E-09
MCM5	0.54	0.535	0.614	0.596	0.998	1.688	0.896	6.186	2.168	2.611	2.043436	up	4.58E-19
GLT8D2	0	0.084	0.034	0.088	0.066	1.227	0.018	0.028	0.066	0.053	2.334374	up	2.37E-12
HSP90B1	157.01	148.585	216.797	181.815	205.894	560.891	234.207	615.893	238.884	445.115	1.20284	up	6.13E-09
ASCL1	5.587	5.175	8.032	2.459	3.31	1.5	0.84	0.765	0.751	0.787	-2.4021	down	1.58E-25
PAH	238.822	233.114	374.235	380.978	642.348	156.645	281.989	141.831	137.822	145.756	-1.11347	down	2.85E-07
IGF1	1.396	2.968	5.042	4.753	9.936	1.255	1.619	0.195	0.205	2.65	-2.02317	down	4.41E-17
PMCH	0	0	0.082	0.042	0	0.099	0.088	1.005	0.579	0.713	4.270123	up	3.06E-18
DRAM1	5.441	3.752	9.407	5.573	6.327	5.902	5.29	19.336	14.281	17.398	1.028147	up	1.03E-06
SVOPL	0.209	0.232	0.035	0.036	0	1.709	0.643	0.983	0.72	1.003	3.291756	up	3.80E-41
CGTL	10.907	8.938	16.05	5.069	31.518	132.906	43.24	116.85	101.128	73.814	2.690539	up	4.26E-36
RAB19	0.297	0.389	1.064	0.366	0.671	2.301	0.989	1.397	0.792	1.117	1.241386	up	3.21E-08
TUBA8B	0.086	0.131	0.433	0.211	0.176	0.138	0.098	1.046	0.499	0.912	1.372535	up	1.46E-06
USP41	2.549	2.52	5.669	4.382	2.167	2.284	1.639	27.99	16.048	28.03	2.135819	up	2.63E-22
AKR1E2	1.799	2.14	3.315	2.908	6.322	4.146	7.353	7.864	9.944	10.893	1.285871	up	5.73E-10
LOC42513	2.991	3.243	1.902	1.998	5.493	11.453	12.748	16.276	15.558	19.706	2.276667	up	6.01E-26
SLCO1B1	1.066	0.722	0.939	0.309	0.386	13.71	6.095	5.634	6.548	9.588	3.600872	up	2.40E-55
LDHB	371.616	323.912	870.705	415.305	520.845	107.071	86.434	100.672	68.484	83.071	-2.48904	down	1.55E-29
ETFRF1	17.209	16.714	14.727	23.218	24.144	10.78	9.396	8.805	8.872	8.814	-1.04073	down	6.86E-07
SSPN	0.694	0.719	3.161	1.225	0.776	5.453	2.264	3.22	2.952	4.734	1.501313	up	6.72E-14
BHLHE41	1.708	1.624	7.576	3.005	1.425	12.728	5.38	6.588	6.598	11.229	1.470832	up	1.53E-13
ENDOUL	1.525	1.284	0.447	0.403	1.314	1.697	0.898	10.395	4.585	1.978	1.974121	up	1.86E-17
SULT4A1	3.1	3.017	2.947	1.635	1.33	10.319	7.838	5.217	7.024	8.928	1.708551	up	4.37E-16
PNPLA3	156.704	150.92	24.41	32.198	22.297	384.097	321.499	198.602	419.803	323.653	2.09175	up	6.05E-22
PARVG	0.833	1.199	1.977	1.356	0.976	2.727	1.95	5.31	4.393	5.75	1.665786	up	2.16E-14
KIAA0930	11.283	11.171	9.327	8.652	9.075	20.902	17.353	27.447	24.238	28.76	1.261501	up	2.41E-09

MANSC1	1.901	1.486	1.474	2.536	0.862	6.286	10.416	4.997	6.837	2.402	1.904701	up	2.07E-18
FOXM1	0.088	0.109	0.094	0.081	0.172	0.333	0.243	3.222	0.635	0.74	3.237517	up	2.18E-36
A2ML1	0.423	0.313	0.55	0.147	0.267	1.217	1.471	1.688	0.86	1.342	1.948973	up	1.76E-20
CD86	0.335	0.326	0.555	0.601	0.084	0.655	0.508	1.294	1.843	2.531	1.842604	up	2.08E-16
GPR162	0.25	0.278	0.697	0.226	0.295	1.053	2.222	2.564	0.693	0.593	2.025723	up	9.50E-20
CDCA3	0.108	0.236	0.287	0.119	0.371	1.818	0.678	13.106	2.777	3.365	4.27167	up	7.03E-55
USP5	10.567	11.536	7.291	8.294	10.055	39.707	16.672	42.804	31.809	22.593	1.685572	up	3.21E-15
LRRC23	6.919	5.962	3.119	4.935	3.728	1.289	3.033	2.217	2.812	1.345	-1.2049	down	9.81E-09
EPHA1	0.234	0.347	0.187	0.234	0.209	0.464	0.522	1.983	0.659	0.625	1.808033	up	9.00E-14
GSTK1	25.813	24.89	36.138	24.733	60.593	12.184	13.001	11.761	10.736	11.456	-1.54157	down	6.60E-13
KEL	0.295	0.55	1.339	0.171	0.418	0.059	0.141	0.011	0.16	0.106	-2.52694	down	3.27E-22
LOC10085	0.216	0.094	1.373	0	0.074	0	0	0	0	0	-8.46107	down	8.98E-14
HSD3B1	3.125	2.257	3.378	3.893	2.655	0.68	2.601	0.718	0.85	1.068	-1.3706	down	4.02E-10
HAO2	41.002	37.026	43.088	67.589	79.114	12.365	49.18	10.989	15.762	19.155	-1.31754	down	1.10E-09
MAB21L3	0.579	0.43	0.396	1.773	0.466	1.594	1.212	1.475	2.719	1.745	1.261782	up	1.26E-08
SLC22A15	1.153	1.048	0.659	2.636	0.798	3.309	2.642	2.574	3.918	3.3	1.321974	up	1.19E-09
CLDND1	82.942	76.685	23.146	32.862	17.209	193.63	145.197	146.126	170.73	241.457	1.945946	up	3.01E-19
APOV1	9688.972	12281.13	57.327	2791.273	5.816	16919.54	19474.43	11907.62	13783.81	21155.54	1.745527	up	2.10E-15
SLC19A2	8.997	8.025	8.924	11.297	8.364	1.354	5.375	0.833	1.761	2.876	-1.90206	down	2.34E-18
COL8A1	0.469	0.473	0.378	0.761	0.475	0.477	1.277	0.86	0.895	1.649	1.011502	up	3.71E-06
KIAA1524	0.124	0.106	0.175	0.078	0.029	0.794	0.578	1.731	0.743	0.507	3.07543	up	2.60E-34
HJURP	0.122	0.071	0.151	0.1	0.222	0.433	0.358	2.068	1.222	0.93	2.902153	up	9.13E-30
LOC41841	17.461	16.654	13.158	18.336	30.201	5.053	11.458	8.52	6.726	4.814	-1.38935	down	5.97E-11
CD200L	0	0	0.023	0.313	0.211	1.418	0.15	4.896	1.592	1.509	4.115779	up	7.98E-44
CREG1	48.616	45.349	90.265	84.383	124.389	28.808	53.224	25.583	30.711	28.644	-1.23492	down	1.31E-08
MAEL	1.904	1.824	2.163	2.202	3.364	0.356	1.391	1.247	0.853	0.991	-1.24289	down	3.28E-09
LIPI	11.979	12.518	3.549	14.683	11.611	18.667	14.808	32.921	29.295	15.396	1.031536	up	3.71E-06
SAMSN1	1.965	1.504	2.99	2.408	1.748	2.531	2.113	6.394	4.338	9.197	1.210584	up	2.10E-08
ADAMTS1	0.289	0.327	0.705	0.609	0.702	1.901	0.386	0.993	0.849	1.29	1.04046	up	8.81E-07
MAP3K7C	6.165	6.29	15.608	12.845	8.111	27.568	30.911	19.713	25.651	37.821	1.530964	up	6.75E-14
EVA1C	7.313	8.262	15.975	6.286	11.069	3.098	3.349	4.053	4.541	6.295	-1.1965	down	3.41E-08
CBR3	25.821	26.229	39.048	34.234	40.938	16.549	14.113	16.16	15.001	11.126	-1.18851	down	2.00E-08
CHAF1B	0.644	0.486	0.679	0.766	0.907	1.242	0.545	4.063	1.517	1.877	1.407311	up	2.10E-10
KCNJ15	0.115	0.131	0.336	0.186	0.268	0.675	1.732	0.334	0.242	0.098	1.567768	up	1.65E-14
PCP4	19.784	17.63	42.577	28.453	30.131	5.615	5.463	13.923	8.223	9.205	-1.70743	down	1.96E-15
MX1	1.326	1.437	2.837	1.887	0.579	3.057	1.268	37.546	25.773	38.009	3.710511	up	6.96E-58
SIK1	4.647	4.614	6.964	4.199	7.148	41.055	7.752	33.604	14.961	11.99	1.98764	up	3.08E-21
VTCN1L	0.067	0.107	0.305	0.46	0.462	0.135	0.239	0.842	0.337	1.397	1.071562	up	2.34E-06

OTC	0.045	0.074	0	0	0	4.613	0.581	2.577	0.745	2.504	6.474295	up	2.32E-78
NR0B1	37.5	36.706	148.366	39.949	35.715	12.902	8.634	9.376	8.159	17.634	-2.3948	down	9.12E-27
SLC51AL	1.037	0.838	1.068	1.955	3.483	0.071	0.125	0.685	0.363	0.229	-2.50434	down	5.99E-28
MAP3K15	1.168	1.278	2.428	1.267	1.369	9.457	3.835	5.799	5.404	9.43	2.174716	up	7.83E-25
PDHA2	12.365	12.837	21.312	13.524	16.276	93.447	37.306	54.09	51.665	88.14	2.088784	up	2.81E-23
ADGRG2	0.147	0.183	0.172	0.12	0.105	0.932	1.322	1.697	2.17	0.828	3.247928	up	9.83E-43
GRPR	0.273	0.29	0.054	0.028	0.035	0.562	0.904	1.427	1.121	1.089	2.898583	up	5.56E-22
PIGA	0.77	0.601	1.027	0.763	0.944	1.717	1.039	2.364	1.828	1.935	1.11272	up	1.85E-07
LOC12110	1.293	1.071	29.826	154.672	238.651	82.124	38.093	12.137	49.06	0.153	-1.22868	down	2.35E-08
LOC10705	27.588	27.701	135.478	188.365	451.459	39.483	138.553	10.527	133.046	0.093	-1.3684	down	1.06E-09
LOC41866	1.345	0.923	1.167	1.339	1.202	1.716	1.028	2.443	3.703	3.641	1.067618	up	1.02E-06
CRLF2	0.753	0.548	2.546	0.64	0.487	12.336	0.751	1.027	0.92	32.366	3.251111	up	1.17E-49
LYGL	0.491	0.321	1.074	0.941	0.252	0.515	0.384	13.243	11.869	6.586	3.402086	up	1.57E-42
LYG2	5.389	7.857	3.234	13.497	12.139	8.982	29.575	7.415	35.73	2.537	1.000034	up	1.48E-06
NPAS2	0.296	0.405	0.548	0.165	0.519	1.013	0.648	2.208	0.679	1.525	1.649028	up	1.01E-13
IL1R2	1.412	1.015	4.233	3.323	1.28	1.231	5.105	2.923	6.183	9.557	1.149927	up	2.15E-08
IL1RL1	1.863	2.123	4.115	1.225	2.715	0.977	2.689	1.883	33.431	5.827	1.895331	up	2.53E-19
IL18R1	0.65	0.527	1.464	0.586	0.686	0.647	0.508	3.047	2.374	3.045	1.296819	up	1.67E-09
LOC77101	171.429	145.53	349.521	449.305	214.164	106.911	166.335	92.858	105.086	143.202	-1.11413	down	1.37E-07
IRS2	4.169	3.889	3.629	2.03	2.478	13.11	2.287	8.31	6.806	5.847	1.166557	up	1.89E-08
RAB20	33.363	32.669	54.294	22.269	35.116	9.953	12.278	21.286	28.671	16.384	-1.00457	down	1.62E-06
LOC12110	1.523	2.514	0.799	7.567	0.711	6.939	9.048	11.168	6.711	4.275	1.539874	up	1.85E-11
GGACT	45.645	52.728	34.891	22.313	18.248	157.062	51.043	165.077	110.762	85.267	1.711299	up	1.26E-15
DOCK9	5.304	5.281	7.746	4.802	5.196	2.426	1.747	3.919	2.107	3.234	-1.07622	down	2.02E-07
HS6ST3	1.605	1.497	1.513	1.551	0.979	0.206	0.53	0.491	0.096	0.707	-1.81291	down	6.52E-17
ACOD1	1.389	1.414	1.721	1.603	0.649	1.902	4.363	11.583	16.237	7.62	2.620823	up	1.10E-31
BORA	0.021	0.051	0.141	0.093	0.189	0.158	0.127	1.655	0.436	0.498	2.525568	up	2.14E-24
LOC11253	1.608	1.246	0.952	2.225	0.38	0.195	0.09	1.078	0.076	0.389	-1.80747	down	8.95E-18
DIAPH3	0.042	0.008	0.019	0.064	0.031	0.081	0.071	1.061	0.196	0.215	3.268891	up	8.65E-31
EPSTI1	2.942	3	5.379	3.795	2.701	3.501	3.032	28.314	15.342	22.458	2.027343	up	2.40E-20
SLC25A30	6.842	5.348	7.314	6.052	4.795	17.397	5.614	11.05	10.217	17.448	1.024013	up	8.05E-07
HTR2A	0.082	0.108	0.159	0.116	0.206	0.274	0.05	0.246	1.075	0.135	1.400829	up	4.19E-08
PHF11	2.079	2.185	3.607	1.956	2.179	1.846	2.388	6.835	5.397	7.902	1.020928	up	1.78E-06
NEK3	1.31	1.418	1.223	1.54	1.438	2.908	2.237	3.244	3.683	2.714	1.09296	up	7.28E-07
CKAP2	0.174	0.142	0.531	0.19	0.312	1.024	0.334	6.055	1.679	1.481	2.965767	up	7.52E-35
POSTN	0.715	0.878	4.066	3.799	2.149	5.282	3.147	8.223	5.586	9.353	1.444125	up	4.82E-12
SLC7A1	1.007	0.953	0.666	0.957	0.926	3.043	1.225	1.544	1.507	1.838	1.021256	up	1.28E-06
SKA3	0.118	0.139	0.414	0.253	0.243	0.961	0.343	2.81	1.091	0.947	2.393255	up	1.56E-20

ZC3H12C	1.766	1.946	1.532	1.719	1.351	0.941	0.524	0.662	0.72	0.874	-1.15878	down	2.74E-08
ACAT1	280.209	264.862	449.211	387.065	396.999	193.051	173.378	142.133	153.7	169.538	-1.09622	down	2.67E-07
MMP1	0	0	0.037	0	0	0.022	0	2.969	1.69	0.93	7.06301	up	1.91E-60
MMP7	0.724	0.148	0.721	0.521	0.093	0.263	0.735	0.118	2.739	0.666	1.032885	up	8.51E-05
BIRC3	36.843	35.835	41.515	30.761	35.499	32.717	40.858	109.246	111.226	105.226	1.14573	up	7.87E-08
ENDOD1	0.612	0.607	0.903	0.924	1.226	0.325	0.346	0.361	0.415	0.323	-1.26878	down	2.31E-08
TAF1D	25.24	23.33	26.332	31.761	22.81	9.695	13.497	11.565	12.216	13.12	-1.10732	down	8.44E-08
THRSPB	67.486	71.497	246.224	149.037	160.855	984.824	476.047	473.672	510.538	679.473	2.168352	up	1.09E-25
THRSP	672.36	706.424	1527.01	988.249	1502.24	4001.762	1725.972	3116.236	2228.042	2440.525	1.32426	up	5.08E-11
CAPN5	2.419	2.309	4.736	2.929	5.462	0.783	0.85	0.739	1.109	0.697	-2.09412	down	2.12E-21
ART7B	0.219	0.315	1.671	1.042	2.874	1.482	3.1	2.912	5.202	1.645	1.227631	up	1.65E-08
IL18BP	0.253	0.332	0.323	0.75	0.573	0.54	0.087	1.986	0.832	1.492	1.144175	up	0.000104
LOC1211(	0.205	0.323	1.583	0.317	0.099	1.108	3.725	0.094	2.36	1.97	1.871046	up	7.86E-20
SLCO2B1	22.211	22.886	18.128	38.38	42.452	9.751	25.447	9.82	11.454	12.285	-1.06701	down	4.42E-07
KCNE3	0.557	0.84	1.841	1.408	1.451	0.166	0.293	0.783	0.571	0.42	-1.44707	down	2.07E-08
P2RY6	0.615	0.827	0.98	1.685	0.807	1.555	1.839	5.4	3.26	5.421	1.829268	up	7.44E-15
ART1	1.709	2.083	2.144	0.936	3.43	1.905	3.295	5.748	6.932	4.425	1.114066	up	2.39E-07
LOC1070£	0.246	0.161	0.033	0.303	0	0.159	0.14	0.482	1.639	0.469	1.951955	up	5.25E-09
HBE	0	0.145	18.385	0	0.152	0	0	0.097	2.885	0	-2.64527	down	4.88E-21
HBBA	251.682	258.768	745.477	87.668	273.636	38.918	35.923	35.107	92.746	16.722	-2.88176	down	2.70E-37
HBE1	46.694	46.334	122.225	11.314	34.039	0.069	2.91	3.337	7.352	1.915	-4.06339	down	6.39E-64
RRP8	6.953	5.844	7.671	5.397	5.629	3.577	2.269	2.302	2.799	2.843	-1.19116	down	3.58E-08
TAF10	33.266	32.073	29.405	36.015	40.872	13.667	11.466	13.86	10.994	12.93	-1.44772	down	5.54E-12
TPP1	197.54	210.865	179.416	267.845	237.865	66.906	54.816	66.982	48.186	65.322	-1.85534	down	9.12E-19
AOC1	48.381	46.087	56.519	27.358	115.457	11.223	33.585	6.281	13.241	9.379	-1.99486	down	2.54E-19
LOC11252	0.509	0.817	1.179	0.399	0.546	0.09	0.277	0.332	0.522	0.34	-1.1416	down	6.65E-06
ELP6	3.722	3.527	2.005	2.384	1.855	4.532	7.06	2.753	6.317	8.639	1.11845	up	1.89E-07
SCAP	12.353	12.424	5.509	6.9	7	17.34	26.33	14.147	18.582	28.898	1.252708	up	2.75E-09
VIPR1	9.132	8.976	16.062	12.995	18.055	4.454	9.61	6.06	6.25	5.547	-1.03069	down	1.76E-06
ACKR2	1.96	1.81	4.688	2.679	3.108	7.389	18.858	5.448	3.736	6.779	1.566794	up	2.74E-15
CYP8B1	3.027	2.459	7.577	4.087	5.467	13.402	35.637	9.103	6.245	12.08	1.757205	up	1.38E-18
CHDSD	1.816	1.503	2.053	1.127	7.993	0.919	0.931	1.454	1.082	1.85	-1.21591	down	2.48E-08
CATH3	0.349	0.513	1.804	1.719	0.09	0	0.074	0.114	0.268	0.214	-2.73054	down	1.40E-12
CATH2	0.158	0.155	1.513	4.098	0	0	0	0	0.325	0	-4.16725	down	8.69E-24
CATH1	0.102	0.201	1.342	3.694	0.21	0.049	0.131	0.067	0.21	0.209	-3.04914	down	2.16E-21
DLEC1	3.841	3.28	5.44	4.057	2.006	0.892	1.365	1.027	0.826	0.996	-1.86587	down	6.85E-18
ACAA1	153.55	145.844	192.261	192.372	200.895	46.633	63.838	67.781	31.13	56.286	-1.73591	down	2.14E-16
MAP3K8	0.832	0.711	0.888	1.681	0.814	2.265	2.29	4.742	6.495	3.273	1.951353	up	9.70E-19

MPP7	1.181	1.023	1.276	1.77	0.598	3.548	2.573	3.294	2.438	2.006	1.244095	up	5.00E-09
LOC10175	0.006	0.036	0.053	0.005	0.011	0.021	0.057	0.497	0.079	1.037	3.869939	up	5.92E-43
MASTL	0.513	0.53	0.294	0.111	0.212	0.218	0.33	1.551	0.784	0.463	1.00907	up	3.89E-05
OTUD1	16.248	16.067	19.749	7.161	12.621	8.767	6.242	7.137	6.253	6.161	-1.0557	down	8.41E-07
ST8SIA6	0	0.022	0	0.055	0	0	1.278	0.13	0.341	0	4.418881	up	5.39E-21
RSU1	2.554	2.116	3.427	2.316	2.365	7.287	3.891	4.947	3.756	5.979	1.016775	up	6.19E-07
FAM188A	3.97	3.07	4.932	3.24	3.909	3.527	3.508	12.562	8.005	10.654	1.000339	up	3.25E-06
ACBD7	1.497	1.217	3.527	1.728	6.917	0.294	0.484	0.423	0.207	1.158	-2.53404	down	1.36E-26
OLAH	0.248	0.282	1.006	0.256	1.495	0.054	0.061	0.084	0.057	0.222	-2.76887	down	1.82E-27
ABCB1LA	27.561	25.71	20.052	17.421	19.97	3.959	8.583	6.179	3.121	9.857	-1.80417	down	1.14E-17
CYP51A1	48.663	52.847	8.028	7.111	62.297	88.917	68.794	59.533	77.017	89.524	1.100753	up	1.39E-07
CDK6	8.803	8.955	11.844	8.675	7.442	32.325	16.922	16.833	18.209	24.744	1.253807	up	7.01E-10
SAMD9L	2.251	2.167	5.343	4.254	2.407	2.37	2.965	22.984	13.666	18.619	1.883463	up	3.94E-18
PDK4	72.111	69.423	92.979	153.728	64.769	9.396	44.949	23.508	13.869	13.568	-2.10512	down	7.96E-23
ASNS	4.736	4.231	3.433	3.862	2.961	16.362	7.035	16.658	12.735	8.181	1.665033	up	7.89E-15
LOC39515	2.725	2.891	0.059	0	0	8.274	0	0.048	0	12.8	1.895125	up	6.01E-16
VWDE	3.3	3.238	1.375	2.446	0.372	1.143	0.049	0.017	0.026	1.406	-2.02057	down	3.01E-21
IGF2BP3	0.18	0.133	0.703	0.327	0.257	0.333	0.253	1.519	0.873	1.692	1.542393	up	2.82E-12
SNX10	2.157	1.47	3.788	2.079	2.308	4.138	1.911	9.162	5.645	8.028	1.290878	up	9.28E-10
HOXA2	1.397	1.516	1.744	0.809	1.141	0.953	0.244	0.29	0.293	0.415	-1.58758	down	3.53E-10
WIPF3	1.121	1.131	1.22	0.407	0.626	1.811	1.118	3	1.999	1.829	1.114049	up	1.23E-07
FKBP14	2.634	2.28	3.848	1.517	2.599	4.64	3.632	6.8	6.766	5.071	1.062888	up	3.38E-07
SATB1	0.905	0.762	1.234	1.188	0.641	3.777	2.194	3.077	3.051	4.325	1.794808	up	3.03E-17
SGO1	0.108	0.05	0.135	0.038	0.095	0.119	0.302	1.988	0.568	0.427	2.983589	up	1.79E-26
LRRC3B	2.343	2.84	6.435	2.755	3.355	1.186	2.57	1.039	1.72	1.406	-1.16177	down	5.19E-07
EOMES	1.415	1.14	1.455	0.616	1.143	2.003	1.571	5.736	1.675	2.246	1.196824	up	4.17E-08
LOC10175	0.982	0.675	1.017	0.363	0.454	0.048	0.294	0.128	0.252	0.241	-1.85263	down	7.00E-09
ACAD11	94.158	91.985	133.79	127.816	125.149	33.112	67.993	33.215	31.761	27.708	-1.56377	down	3.54E-13
CCR5	1.249	1.284	2.616	1.662	1.038	1.497	2.451	4.272	4.591	3.538	1.058144	up	6.48E-07
CCR2	6.706	6.107	9.783	10.106	6.604	9.373	11.26	22.841	19.812	19.315	1.071314	up	4.56E-07
KIF15	0.065	0.038	0.103	0.331	0.214	0.55	0.264	2.789	1.254	0.911	2.932863	up	1.92E-33
TRANK1	16.125	15.35	13.587	10.786	8.698	31.638	22.274	77.708	63.325	72.309	2.049727	up	4.33E-21
LOC42073	0.229	0.15	0.438	0.113	0.094	0.178	0.157	1.078	0.752	0.6	1.428643	up	1.15E-05
ANLN	0.042	0.049	0.153	0.144	0.232	0.405	0.335	1.899	0.771	0.622	2.691355	up	3.94E-28
AMPH	0.024	0.056	1.094	0.01	2.771	0.047	0.135	0.063	0.15	0	-3.30743	down	8.15E-32
LOC12110	0.547	0.596	2.177	0.599	0.468	0.397	0.35	0.119	0.327	0.745	-1.17659	down	7.52E-05
INHBA	4.865	4.491	2.44	9.618	6.196	11.483	24.098	7.146	8.825	22.262	1.41854	up	1.44E-11
EZH2	0.874	0.961	1.905	1.387	1.771	1.87	1.66	4.854	2.89	4.231	1.167903	up	4.67E-08

IGFBP1	40.494	39.916	40.039	34.635	17.005	13.132	12.893	19.16	4.839	8.351	-1.55965	down	7.74E-14
TRIP13	0.219	0.387	0.174	0.162	0.562	0.381	0.28	1.772	0.471	0.608	1.220753	up	5.03E-06
SALL3	0.028	0.029	0.303	0.176	0.231	0.844	0.751	0.664	1.033	0.996	2.475313	up	5.98E-30
DCDC2	1.066	0.95	2.24	1.084	1.124	1.753	2.577	4.481	5.002	3.856	1.450014	up	4.03E-12
NRSN1	2.815	2.74	1.361	1.638	0.533	3.066	4.05	9.738	7.51	1.713	1.520385	up	1.32E-11
ID4	2.554	3.021	38.134	8.279	11.246	1.626	0.657	0.914	1.077	1.116	-3.55112	down	1.28E-47
ELOVL2	182.236	179.616	5.847	23.01	45.086	410.411	287.903	318.808	357.125	320.689	1.959497	up	3.30E-19
SYCP2L	87.486	81.32	3.059	10.072	17.064	169.328	121.436	133.015	153.047	136.438	1.841634	up	3.48E-17
TXNDC5	2.63	2.896	5.819	4.605	3.285	8.024	5.69	12.273	6.724	7.36	1.05863	up	3.47E-07
PPP1R3G	6.647	6.35	8.518	6.672	18.704	4.015	2.137	3.242	3.888	2.793	-1.5442	down	5.76E-13
ECI2	248.06	223.024	457.551	325.633	209.669	51.489	70.964	76.521	56.723	61.225	-2.20764	down	1.39E-24
SERPINB1	0.182	0.238	0.512	0.287	0.359	0.745	0.263	1.608	0.99	1.796	1.772167	up	3.12E-14
LOC4209C	1.399	1.359	0.971	1.558	1.669	4.459	3.792	2.299	4.322	2.916	1.353943	up	1.82E-10
CTNND2	3.216	2.687	0.587	0.945	0.31	5.104	2.4	3.248	4.025	3.135	1.209061	up	3.11E-08
CMBL	109.934	119.002	147.194	84.218	175.479	18.043	28.211	46.073	24.749	13.136	-2.28773	down	1.97E-26
C5orf49	0.039	0	0	0.193	0.322	0.607	1.373	0.102	0.121	0	1.98182	up	3.87E-10
SRD5A1	3.159	2.519	9.173	6.425	4.217	1.483	1.881	1.848	1.255	1.609	-1.65778	down	3.66E-14
LOC1211C	0.586	0.332	0.218	0.257	0.241	0.983	0.867	0.204	1.161	0.638	1.235037	up	3.40E-06
MOCOS	33.131	32.776	10.736	22.622	11.783	49.566	64.661	37.227	83.763	48.819	1.354851	up	2.39E-10
SEC61B	63.379	70.841	78.921	65.259	55.47	157.547	89.304	173.815	123.97	175.099	1.108168	up	1.17E-07
IFI6	18.124	21.104	45.414	28.49	5.721	53.617	12.925	2324.65	1339.473	2801.815	5.780321	up	#######
GLIPR2	0.355	0.398	0.878	0.698	0.456	1.693	0.812	1.407	1.508	1.658	1.344095	up	6.82E-10
FAM65B	0.393	0.451	1.813	0.794	0.463	2.112	0.771	1.74	1.551	1.783	1.022645	up	2.62E-07
LDLRAD4	4	4.112	6.699	4.179	11.312	2.34	2.491	3.312	3.724	3.103	-1.0171	down	1.69E-06
CIDEA	199.64	214.587	173.713	209.271	309.342	96.034	141.676	119.674	91.874	67.985	-1.09715	down	1.54E-07
TGIF1	12.25	10.563	11.097	3.061	3.781	3.472	3.591	5.129	3.043	4.259	-1.06365	down	3.31E-07
MYOM1	1.006	0.83	1.409	0.757	2.832	0.87	0.551	0.419	0.972	0.406	-1.08538	down	1.18E-06
LPIN2	29.822	30.149	58.891	30.487	110.317	18.669	28.362	21.996	27.166	16.919	-1.19887	down	3.78E-08
NDC80	0.325	0.206	0.606	0.396	0.4	0.449	0.333	3.162	1.132	1.653	1.796895	up	3.53E-15
COLEC12	0.88	0.745	0.979	1.147	1.169	1.308	1.283	4.063	2.008	2.308	1.156025	up	2.66E-07
ABHD3	12.343	10.311	30.543	4.203	14.329	2.362	0.941	2.173	1.442	1.011	-3.17654	down	6.59E-44
LAMA3	0.63	0.857	1.484	1.121	2.179	0.264	0.469	0.665	0.418	0.455	-1.46334	down	1.04E-11
DSC1	0.063	0.069	0.006	0.006	0	2.726	0.12	0.979	0.539	1.289	5.246908	up	3.29E-76
MCM4	0.945	0.866	1.037	0.742	1.931	2.065	1.759	8.013	3.297	2.8	1.698792	up	7.99E-15
C8orf22	2183.872	2049.309	595.971	1564.483	746.212	372.55	586.288	816.627	417.479	662.544	-1.32215	down	2.03E-11
CYP7A1	5.926	9.393	3.053	30.473	24.168	74.876	69.66	43.681	71.586	101.342	2.306274	up	2.83E-26
MTFR1	103.881	100.861	62.933	67.583	61.468	36.845	41.77	39.128	31.02	38.654	-1.08187	down	1.14E-07
XKR9	0.272	0.235	0.097	0.395	0.345	2.963	0.772	1.082	1.147	2.033	2.56847	up	6.79E-30

MSC	0.485	0.571	1.7	0.459	0.424	0.047	0.269	0.254	0.348	0.238	-1.65015	down	1.02E-09
TRPA1	1.316	1.061	0.084	2.772	4.407	0.489	0.201	0.476	0.362	0.365	-2.3453	down	2.19E-25
TERF1	0.611	0.5	0.6	0.619	1.538	0.602	0.922	4.031	1.703	1.258	1.137571	up	4.14E-07
PKIA	1.626	1.396	0.728	0.167	0.522	0.098	0	12.213	0.417	7.148	2.16146	up	1.01E-14
FABP5	9.668	10.196	10.394	16.943	14.285	4.017	2.657	4.262	4.943	5.886	-1.49803	down	7.90E-13
FABP4	0.671	0.603	1.803	3.858	0.861	0.244	0	0.11	0.688	1.097	-1.86335	down	1.08E-10
CA13	2.575	2.243	4.942	1.926	2.63	0.546	0.371	1.228	0.534	0.733	-2.06733	down	2.35E-20
ATP6V0D2	0.331	0.185	0.978	0.233	0	3.157	3.774	2.53	5.986	8.076	3.763868	up	2.01E-54
PTDSS1	16.268	15.45	18.831	8.89	8.963	53.26	41.726	19.38	28.714	31.363	1.350581	up	1.67E-11
CPQ	22.699	22.404	10.172	11.779	18.934	49.185	31.756	53.217	59.634	59.345	1.557656	up	3.66E-13
LAPTM4B	0.205	0.355	1.226	0.301	0.873	0.085	0.088	0.421	0.195	0.36	-1.36139	down	4.49E-08
NCALD	15.006	14.897	22.578	15.2	31.005	6.759	13.303	10.256	7.786	6.671	-1.14006	down	1.66E-07
DCSTAMP	4.222	3.077	3.015	0.466	0.332	0.85	0.975	0.707	1.137	1.343	-1.14787	down	6.26E-08
ANGPT1	0.75	0.772	1.704	1.278	1.416	1.709	1.225	3.847	2.66	2.896	1.058689	up	7.23E-07
SAMD12	1.124	1.049	1.62	2.375	2.304	2.771	3.989	5.379	3.248	3.084	1.124027	up	8.11E-08
SQLE	22.329	24.556	0.467	0.583	24.186	49.622	25.713	18.349	27.13	33.355	1.095968	up	1.57E-07
FAM84B	4.677	4.439	9.128	4.27	6.928	2.488	2.997	2.234	3.433	1.936	-1.16932	down	8.85E-08
TMEM71	0.268	0.202	0.374	0.292	0.265	0.439	0.313	1.024	0.735	1.113	1.367975	up	6.97E-10
NDRG1	187.047	174.209	179.567	230.082	172.537	124.898	126.319	64.366	67.363	84.378	-1.0135	down	1.67E-06
LY6E	213.996	237.845	542.189	956.757	125.634	450.885	289.767	2131.955	1135.436	1649.264	1.446014	up	6.16E-11
RHPN1	0.708	0.818	1.538	0.937	1.039	0.178	0.534	0.324	0.472	0.135	-1.61414	down	3.04E-12
TSTA3	4.979	5.299	4.769	6.335	5.727	14.309	7.271	13.755	9.697	11.277	1.054456	up	6.91E-07
LOC12111	3.227	3.889	5.916	2.652	5.582	1.151	0.471	1.442	0.436	0.903	-2.27069	down	9.30E-22
BUB1	4.253	4.887	8.572	9.029	2.457	16.877	16.629	8.61	12.362	30.737	1.545075	up	1.02E-13
LOC42123	1.271	1.004	1.146	1.936	0.635	0.623	0.394	0.397	0.748	0.481	-1.17934	down	1.72E-08
LOC10174	0.314	0.441	0.501	0.258	1.015	1.435	1.458	1.584	1.382	0.698	1.372719	up	3.59E-08
CAPN13	0.264	0.107	0.381	1.645	0.336	0.232	0.161	0.115	0.168	0.124	-1.76606	down	4.76E-13
EHBP1	19.949	18.589	22.644	19.693	24.768	7.389	13.694	10.526	11.406	9.613	-1.00523	down	1.72E-06
WDPCP	0.147	0.144	0.135	0.085	0.177	9.137	0.26	2.229	1.428	1.57	4.399832	up	1.95E-77
UGP2	111.949	111.93	146.823	110.221	109.185	1281.37	238.139	896.02	636.241	517.972	2.596759	up	2.70E-33
SLC1A4	0.663	0.798	0.625	0.501	0.447	0.722	0.944	3.125	1.73	2.356	1.547288	up	2.07E-12
SLX4IP	0.403	0.306	0.459	0.278	0.532	0.746	0.547	0.79	0.938	1.384	1.153094	up	9.02E-08
BIRC5	1.724	1.45	1.863	1.889	2.235	2.147	2.209	14.531	6.401	4.667	1.708673	up	1.36E-13
RRBP1	20.908	22.272	26.808	27.12	8.192	46.032	47.201	44.661	56.755	51.672	1.225995	up	4.03E-09
GINS1	0.504	0.431	0.617	0.7	1.273	1.613	0.695	5.717	2.146	2.376	1.830177	up	7.01E-14
TLR5	1.701	1.462	1.014	2.244	0.513	0.252	0.36	0.397	0.582	0.169	-1.97506	down	3.76E-21
ATF3	1.389	1.264	2.887	1.079	1.605	1.577	0.686	6.678	4.677	6.862	1.315779	up	7.32E-10
DTL	0.013	0.017	0.01	0.032	0.122	0.178	0.202	1.615	0.661	0.28	3.885466	up	2.26E-36

LPGAT1	63.84	62.964	73.877	58.585	71.183	19.719	51.271	25.607	23.347	25.992	-1.17906 down	3.33E-08
LOC10705	0.246	0.209	0.176	0.445	0.329	1.048	0.546	0.932	0.757	1.066	1.626646 up	1.91E-10
LOC42141	15.524	14.791	15.424	24.637	22.95	40.15	40.547	40.372	26.714	46.629	1.058726 up	3.37E-07
CMTR1	6.775	6.425	9.906	6.185	5.38	6.694	5.775	23.612	15.992	23.057	1.115548 up	1.75E-07
NFKBIE	1.106	0.82	1.526	1.434	1.085	1.19	1.218	4.462	3.227	5.587	1.392501 up	7.21E-10
EIF2AK2	6.762	6.172	8.032	8.465	7.513	9.888	7.731	32.047	17.25	28.138	1.363288 up	2.93E-10
GREM2	0.261	0.43	1.984	0.788	0.728	0	0.009	0.422	0	0.307	-2.49758 down	5.48E-25
ACTN2	0.321	0.202	0.02	0.061	0.025	1.184	0.222	0.226	0.38	0.546	2.015279 up	3.22E-15
SLC35F3	0.254	0.206	0.501	0.338	1.305	0.433	0.32	0.19	0.211	0.04	-1.12167 down	1.87E-05
CCSAP	1.888	1.887	5.386	1.806	2.662	0.773	1.532	2.04	1.365	0.948	-1.03297 down	3.02E-06
C1orf131	2.91	3.293	3.071	1.971	2.484	7.38	5.743	3.956	8.717	5.011	1.16574 up	1.28E-08
GNPAT	11.337	11.076	5.99	4.546	8.803	35.819	19.576	16.256	20.095	14.764	1.350967 up	6.49E-11
SULT	1527.22	1357.682	2707.046	1942.924	485.372	1036.263	1050.345	207.815	513.027	1064.838	-1.05046 down	1.65E-06
DACT2	0.361	0.382	0.406	0.794	2.125	0.092	0.19	0.194	0.174	0.139	-2.35888 down	6.55E-22
MPC1L	355.379	296.186	600.226	259.974	295.607	105.244	123.391	212.053	79.446	138.897	-1.45547 down	5.80E-12
TBXT	0.389	0.238	0.47	0.402	0.245	0.523	0.214	0.492	0.773	1.499	1.003296 up	1.36E-05
LOC42158	1.671	1.488	1.635	0.86	0.951	0.702	0.997	0.105	0.041	0.165	-1.71387 down	6.18E-12
FNDC1	2.801	2.213	0.846	1.502	0.292	0.282	0.135	0.479	0.402	0.44	-2.13558 down	1.14E-23
EZR	1.536	1.423	2.385	1.348	1.966	3.568	1.602	7.041	3.406	3.908	1.172753 up	3.97E-08
LOC12111	0.286	0.402	0.159	5.69	0.72	0.055	0.086	0.355	0	0.538	-2.80517 down	1.05E-33
MTFR2	0.078	0.076	0.418	0.064	0.16	0.151	0.233	1.955	0.798	0.493	2.182081 up	1.36E-16
SLC2A12	0.173	0.198	0.189	0.284	0.344	0.721	1.623	0.592	0.653	0.388	1.738899 up	1.46E-14
LOC10705	32.969	32.892	24.991	42.267	11.618	52.368	46.958	77.608	124.193	71.77	1.365313 up	3.08E-10
TMEM200	0.75	0.771	2.214	0.551	2.284	1.953	2.358	4.594	4.185	3.539	1.339072 up	4.96E-11
CENPW	0.078	0.051	0.112	0.103	0.193	0.198	0.255	2.767	0.853	0.847	3.183759 up	8.98E-32
HEY2	0.109	0.091	0.21	0.076	0.096	1.352	0.53	1.216	0.811	1.459	3.194295 up	4.65E-35
GJA1	19.079	19.274	11.943	15.972	6.838	78.817	48.849	56.569	60.992	74.048	2.126662 up	7.92E-23
DCBLD1	3.256	3.62	2.803	2.341	1.677	11.849	8.825	9.62	8.012	11.587	1.864596 up	2.16E-18
LOC42174	143.135	116.205	363.465	245.924	407.447	68.272	90.7	49.419	75.937	54.101	-1.91489 down	2.57E-18
SLC16A10	16.435	16.164	23.918	18.022	32.807	6.9	14.482	7.818	8.895	6.583	-1.26454 down	4.90E-09
DDO	1.907	2.007	1.419	2.226	1.731	7.212	1.959	5.505	4.791	3.266	1.290579 up	2.30E-09
LOC42179	2.157	1.85	1.565	2.206	3.151	3.621	3.562	13.837	11.851	9.84	1.965955 up	7.75E-18
MMS22L	0.133	0.196	0.238	0.24	0.642	0.444	0.346	1.198	0.566	0.62	1.128546 up	4.85E-07
NT5E	0.293	0.453	1.344	0.723	0.527	1.177	1.602	2.149	1.989	1.226	1.28444 up	1.02E-09
ME1	8.539	9.374	9.18	7.501	16.873	207.856	33.552	127.613	115.935	100.068	3.506648 up	8.15E-55
RWDD2A	3.585	3.795	3.749	2.782	5.311	44.115	7.801	24.953	22.984	21.77	2.66127 up	1.18E-34
TTK	0.133	0.043	0.098	0.164	0.234	0.221	0.17	1.798	0.565	0.575	2.300026 up	6.58E-20
SH3BGRL2	33.767	31.02	42.679	24.48	19.65	20.752	14.994	12.276	12.387	9.94	-1.10757 down	2.49E-07

GFRAL	1.187	1.504	0.189	0.175	0.026	0.109	0.213	0.163	0.551	0.245	-1.26285	down	1.05E-08
LRRC1	1.144	0.964	0.817	0.821	0.446	2.241	1.932	1.786	1.551	0.968	1.015216	up	2.67E-06
CMPK2	8.775	8.064	19.72	12.554	3.339	10.007	7.094	97.503	55.549	87.368	2.295511	up	1.04E-25
RSAD2	1.418	0.976	3.298	2.576	1.341	2.204	1.211	90.115	36.632	80.65	4.454711	up	6.73E-75
ID2	27.698	20.921	48.734	28.432	43.509	14.23	14.281	17.114	13.781	17.985	-1.12924	down	1.48E-07
MBOAT2	54.307	53.068	2.92	4.222	2.508	83.244	88.154	42.678	58.649	56.066	1.490314	up	4.48E-12
LOC42193	2.428	2.592	1.221	1.706	4.134	4.98	3.575	5.216	4.522	5.904	1.001789	up	4.30E-06
RRM2	0.136	0.204	0.619	0.895	0.879	2.109	1.312	26.308	6.003	5.247	3.903866	up	1.60E-57
VSNL1	18.663	15.883	23.938	16.742	23.218	3.723	10.473	8.775	4.914	11.137	-1.3349	down	2.72E-10
TTC32	9.556	8.712	8.732	6.738	2.95	24.879	14.453	6.821	19.874	15.331	1.148868	up	3.01E-08
RHOB	166.363	172.302	92.283	194.081	174.943	26.817	30.025	58.39	17.679	22.828	-2.36078	down	4.09E-29
APOB	1455.796	1551.823	478.735	607.197	484.529	2491.767	2234.636	2242.712	2614.042	2296.036	1.375621	up	1.40E-10
PRKACA	28.773	31.426	34.977	27.061	24.916	66.563	34.728	85.254	69.113	62.248	1.111257	up	1.18E-07
CGREF1	0.196	0.154	0.062	0.644	0.845	1.67	0.536	8.139	0.965	3.013	2.910217	up	2.76E-28
SLC5A6	43.773	41.637	47.298	34.546	44.871	21.483	21.287	22.517	18.692	19.935	-1.02949	down	9.15E-07
DTNB	12.246	11.435	2.323	2.942	2.779	2.476	5.472	1.263	1.285	1.277	-1.42975	down	1.25E-11
KIF3C	1.136	1.361	1.274	1.236	1.068	10.099	2.501	8.333	7.369	6.442	2.514828	up	1.54E-30
RAB10	39.592	36.129	37.608	29.044	35.162	83.692	47.068	90.601	73.015	68.209	1.030195	up	7.27E-07
HADHB	362.649	336.843	399.756	407.366	334.114	125.369	147.076	185.564	88.079	141.84	-1.41994	down	8.12E-12
LOC12111	6.675	7.37	25.456	52.844	35.568	8.737	10.825	5.994	4.066	6.013	-1.84365	down	2.86E-17
GVINP1	13.324	13.946	18.782	19.544	16.333	5.104	5.407	6.848	5.569	6.137	-1.49493	down	8.31E-13
LOC77027	0.949	1.031	0.455	0.826	1.47	0.514	0.192	0.63	0.33	0.421	-1.17879	down	3.00E-08
LOC77099	0.509	0.541	1.469	0.349	0.762	1.15	0.978	2.382	0.653	2.41	1.059862	up	1.11E-06
C3H8ORF8	4.545	4.435	4.744	7.777	3.666	11.116	5.506	35.604	25.908	31.637	2.124671	up	7.98E-22
NUGGC	0.893	0.71	1.748	0.503	0.446	1.322	1.406	1.5	3.443	3.684	1.399878	up	3.39E-11
PBK	0.135	0.159	0.086	0.133	0.138	1.278	0.805	7.812	1.769	1.983	4.379273	up	7.97E-54
LOC12111	1.558	1.463	1.781	1.265	1.111	2.316	1.715	6.416	3.178	6.888	1.514232	up	3.36E-12
CLU	11.367	12.74	10.805	12.473	30.681	11.626	11.027	89.366	31.072	121.308	1.759885	up	6.92E-16
ZNF395	2.428	2.581	6.638	7.327	10.156	2.299	4.183	2.088	2.546	2.62	-1.08427	down	7.53E-07
LOC42638	0.052	0.183	0.718	0.776	0.394	0.542	1.028	1.26	0.936	0.56	1.025202	up	2.78E-06
MSRA	11.61	11.261	12.555	13.88	16.305	6.79	5.54	4.197	4.191	4.835	-1.36027	down	1.70E-10
SOX7	3.242	3.065	2.072	1.882	2.475	49.543	22.474	38.501	46.228	41.024	3.95631	up	2.10E-65
XKR6	0.687	0.589	1.533	0.802	0.901	0.141	0.589	0.327	0.296	0.549	-1.24406	down	1.13E-08
TDH	348.897	331.718	393.93	562	483.821	131.256	329.793	104.618	146.428	157.942	-1.28516	down	1.68E-09
BLK	0.486	0.712	1.101	0.58	0.771	3.704	1.395	7.322	3.683	3.393	2.415679	up	5.57E-27
AvBD13	65.643	71.449	112.278	364.193	337.728	20.096	48.279	2.499	22.799	15.767	-3.11969	down	4.09E-43
AvBD7	0.154	0.656	0.922	7.927	0	0.224	0.132	0.202	0.871	0.316	-2.46527	down	1.29E-18
DEFB4A	0.275	0.763	1.804	6.43	0	0.266	0.059	0.269	0.704	0	-2.83182	down	3.16E-22

AvBD1	0.883	0.525	1.279	6.927	0.165	0.078	0.069	0.315	0.741	0.263	-2.73363	down	1.12E-20
MCM3	0.205	0.191	0.262	0.324	0.466	0.92	0.45	4.861	1.86	1.674	2.749324	up	3.40E-32
CRISP2	0.086	0.075	2.056	1.533	0.56	0.083	0.368	0	0.206	0.094	-2.5129	down	3.33E-17
RHAG	1.114	1.21	2.461	0.895	1.691	0.294	0.325	0.033	0.416	0.124	-2.62342	down	3.31E-23
CYP2AC1	632.367	539.155	1236.022	975.454	1033.102	65.746	385.485	80.486	100.39	129.591	-2.53547	down	6.19E-30
SUPT3H	5.372	5.091	5.659	4.89	14.773	2.088	2.007	3.257	2.162	2.748	-1.54477	down	3.78E-13
RCAN2	2.931	2.809	2.735	5.073	3.064	2.129	0.993	1.788	1.382	1.035	-1.18038	down	1.11E-08
ADGRF5	2.642	2.89	2.883	2.995	1.282	0.579	1.251	1.4	0.775	0.791	-1.40308	down	1.73E-11
ARR3	1.942	1.912	0.361	1.283	1.343	0.088	0.14	0.617	0.41	0.134	-2.29603	down	3.57E-23
KIF4B	0.161	0.146	0.335	0.14	0.257	0.815	0.665	5.201	1.533	1.39	3.202265	up	5.30E-41
LOC42214	0.586	0.777	0.778	0.914	0.602	0.509	0.783	1.551	1.773	3.442	1.138685	up	6.15E-07
RAB33A	0.709	0.497	1.612	0.333	0.364	0.343	0.432	0.463	0.052	0.331	-1.11425	down	0.000352
ERCC6L	0.197	0.281	0.278	0.213	0.138	0.407	0.321	1.588	0.697	0.6	1.702036	up	1.10E-12
CCNB3	0.287	0.137	0.458	0.193	0.295	1.037	0.536	5.767	2.037	1.987	3.047602	up	7.45E-33
CENPI	0	0.033	0.081	0.052	0.144	0.383	0.185	1.885	0.72	0.574	3.574236	up	4.71E-37
GCNA	0.329	0.225	0.656	0.406	0.169	0.638	0.332	2.307	0.921	0.71	1.456645	up	9.52E-10
SLC6A14	0.466	0.499	0.399	2.202	1.155	0.124	0.398	0.419	9.879	0.829	1.302134	up	6.95E-09
FAM162BI	1.129	1.318	2.071	3.171	3.112	2.698	3.393	4.834	8.385	4.37	1.132141	up	1.15E-07
GPC3	0.465	0.397	0.123	0.324	0.362	1.665	1.517	0.663	1.085	1.049	1.836088	up	1.47E-15
SLITRK4	0.064	0.08	0.058	0.063	0.023	0.268	0.233	0.231	0	1.025	2.588241	up	7.64E-26
NSDHL	29.648	29.047	3.061	3.52	36.354	55.012	36.23	30.292	40.787	43.279	1.016478	up	1.08E-06
SLC16A2	78.76	83.669	98.773	104.677	105.799	28.272	46.328	39.607	43.898	34.113	-1.29504	down	5.91E-10
IRS4	24.867	22.638	34.105	10.963	15.878	8.814	8.7	10.372	10.474	6.713	-1.26661	down	2.76E-09
COL4A5	0.178	0.12	1.061	0.118	0.092	0.053	0.114	0.032	0.03	0.04	-2.52219	down	5.78E-21
LOC12111	1.993	1.467	3.592	1.296	2.075	3.109	4.137	10.518	4.996	7.74	1.548583	up	6.22E-13
SH2D1A	1.818	1.263	2.994	1.244	1.728	2.854	3.405	9.115	4.576	7.277	1.588994	up	6.47E-14
GPR50	0.46	1.164	1.026	2.369	0.598	2.583	1.86	1.682	2.485	2.758	1.016458	up	1.06E-05
1-Mar	1.312	1.57	1.533	2.01	1.198	1.57	1.492	4.864	3.949	4.107	1.067522	up	1.40E-06
MSMO1	201.642	197.348	11.709	32.025	267.106	355.686	254.401	224.31	327.846	280.567	1.023331	up	9.92E-07
DDX60	2.713	2.607	4.405	23.13	14.791	4.227	18.07	93.212	47.419	98.544	2.456106	up	7.08E-28
MGARP	12.176	11.256	20.313	18.498	21.268	3.265	8.778	9.24	5.839	10.249	-1.15994	down	2.86E-08
TTC29	2.221	1.929	0.238	0.327	0.153	0.078	0.175	0.512	0.057	0.57	-1.80248	down	4.84E-18
TMEM154	0.468	0.242	0.279	0.224	0.26	0.51	0.416	2.088	0.68	0.718	1.579421	up	5.53E-10
PLK4	0.218	0.142	0.217	0.224	0.212	0.622	0.288	1.706	0.86	0.694	2.036039	up	3.88E-17
CXCL13L3	0	0	0.136	0.07	0.088	0.498	0.073	0.336	0.439	1.471	3.238501	up	4.65E-12
CCNG2	14.072	13.32	17.076	18.961	23.953	30.806	30.812	41.151	39.822	34.099	1.015771	up	9.02E-07
LOC42251	4.013	4.053	11.026	7.756	3.707	6.349	5.453	48.913	28.468	43.391	2.117136	up	1.65E-22
MMRN1	2.569	2.953	0.155	0.021	0.035	0	0.094	0	0	0.028	-5.49765	down	1.85E-76

ACSL1	167.114	151.745	183.9	262.414	249.984	35.568	76.48	75.387	36.508	61.158	-1.83214 down	2.99E-18
WWC2	28.127	23.253	60.659	14.844	11.517	17.726	15.11	10.83	9.079	16.179	-1.00571 down	8.09E-06
NEIL3	0.115	0.054	0.124	0.195	0.211	0.294	0.098	1.114	0.53	0.342	1.759132 up	1.44E-12
HMGB2	3.299	3.25	3.424	2.955	5.749	4.703	4.307	15.93	10.191	8.67	1.22948 up	2.17E-08
VEGFC	7.835	8.058	18.325	5.016	10.031	2.805	2.163	5.997	4.673	1.877	-1.49171 down	4.30E-12
EREG	1.265	1.313	1.633	0.43	0.762	0	0.783	0.057	0.626	0.571	-1.40511 down	3.40E-07
AREG	1.735	1.123	8.198	0.737	0.521	3.078	0.45	0.484	0.22	1.148	-1.19387 down	3.55E-06
PLACL2	2.75	3.512	6.256	7.048	3.026	6.023	3.523	53.017	35.192	25.913	2.452327 up	6.36E-27
GPAT3	7.264	7.386	8.397	9.107	17.669	2.696	3.544	5.401	3.931	3.652	-1.37367 down	5.66E-11
DUSP4	0.599	1.122	0.629	0.47	1.118	3.189	0.884	1.671	1.201	1.937	1.172403 up	2.16E-07
LOC4303	0.074	0.101	0.177	0.084	0.065	0.249	0.937	12.027	0.176	0.178	4.745352 up	8.12E-77
SHROOM	0.496	0.407	1.226	0.995	0.284	0.17	0.18	0.183	0.207	0.093	-2.02602 down	5.86E-19
NAAA	2.407	2.572	5.92	3.516	4.807	6.345	4.203	10.374	5.569	12.203	1.009163 up	1.20E-06
DCK	0.47	0.517	0.913	0.896	0.888	1.693	1.301	3.879	1.909	2.244	1.580262 up	2.60E-12
ADAMTS3	1.836	1.893	2.519	1.536	1.694	3.301	4.057	6.441	5.063	5.115	1.338537 up	1.61E-10
CENPC	0.253	0.413	0.793	0.45	0.663	0.544	0.839	2.181	1.296	1.687	1.346243 up	2.00E-09
SULT1B	30.586	33.569	49.609	13.958	87.131	8.271	25.026	25.132	9.357	5.509	-1.5515 down	9.42E-13
CCNA2	1.02	1.09	1.575	1.403	1.133	2.742	2.792	10.196	3.374	3.813	1.880355 up	5.25E-17
MAD2L1	0.749	0.791	1.49	1.444	1.361	1.255	2.288	8.162	2.216	2.708	1.510095 up	4.75E-11
FABP2	0	0	0	1.883	0.102	0	0	0	0	0	-8.63662 down	6.97E-13
ARSJ	0.539	0.362	0.419	0.299	0.429	0.444	0.392	1.426	1.106	1.014	1.095501 up	3.57E-06
PITX2	0.311	0.445	0.449	0.399	0.359	0.846	0.514	1.801	1.016	0.762	1.328949 up	4.95E-08
ENPEP	8.84	8.815	9.553	7.139	5.576	18.62	16.755	25.686	21.752	17.723	1.332311 up	2.21E-10
CASP6	17.864	14.593	13.318	12.265	6.664	31.879	18.475	26.05	29.114	37.196	1.141139 up	6.54E-08
C4orf17	15.074	24.217	14.516	24.06	31.974	50.485	69.23	36.576	30.116	34.497	1.00797 up	6.16E-07
DAPP1	1.269	1.467	2.62	2.365	2.88	2.829	3.63	6.071	5.525	5.491	1.150908 up	4.97E-08
CENPE	0.039	0.025	0.116	0.1	0.115	0.335	0.158	2.161	0.729	0.708	3.356144 up	1.63E-42
CCDC110	0.033	0	0	0.26	0.017	0.323	0.271	0.544	1.83	0.3	3.37719 up	3.13E-29
TLR3	3.55	3.085	3.735	4.778	5.225	6.14	6.231	19.013	13.597	19.331	1.658188 up	1.67E-14
LRRC66	0.271	0.239	1.178	0.277	0.231	0.209	0.232	0.208	0.163	0.253	-1.04055 down	5.39E-05
UCHL1	4.533	3.235	1.078	0.242	16.085	13.226	15.992	27.375	24.761	3.9	1.759688 up	1.83E-16
FAM114A	3.049	2.595	2.04	3.165	2.268	6.886	4.351	9.912	7.902	6.331	1.431232 up	3.84E-11
TLR1A	0.625	0.963	1.381	1.748	1.429	1.357	1.438	4.134	3.071	4.584	1.245988 up	1.17E-08
TBC1D1	6.054	5.95	6.763	5.365	6.448	11.183	11.984	15.293	18.565	16.297	1.26152 up	1.49E-09
RBPJ	16.543	15.854	18.495	3.857	20.887	1.759	1.539	4.091	5.172	2.109	-2.36581 down	3.49E-28
SEL1L3	23.422	22.452	35.091	17.068	33.257	10.192	7.688	17.329	17.354	8.716	-1.09923 down	1.40E-07
PPARGC1	6.235	5.475	3.024	3.071	5.326	1.065	1.19	1.112	0.862	0.755	-2.21332 down	8.00E-25
NCAPG	0.103	0.105	0.31	0.132	0.412	0.721	0.284	2.737	0.799	1.124	2.409789 up	4.44E-24

BST1	21.71	22.562	2.829	13.861	10.918	29.366	19.049	55.51	28.111	27.425	1.149486	up	3.32E-07
EVC2	0.867	0.646	0.628	0.455	0.383	1.661	1.347	1.376	1.63	1.546	1.342093	up	3.06E-10
HAUS3	0.566	0.536	0.789	0.756	0.582	1.576	1.529	3.117	1.66	2.39	1.668028	up	3.72E-14
TACC3	3.278	3.328	3.72	3.324	3.238	3.77	4.066	15.289	8.388	8.946	1.260213	up	7.65E-09
SLBP	0.132	0.245	0.946	0.56	0.226	0.745	0.658	9.738	3.745	8.165	3.447095	up	6.72E-46
SPON2	0.232	0.346	0.632	3.372	0.765	1.298	35.19	4.905	3.14	1.353	3.100061	up	1.37E-43
ATOH8	15.211	13.715	16.493	12.129	13.617	6.504	5.501	5.224	6.598	6.563	-1.22744	down	5.54E-09
CD8A	1.189	1.415	3.43	1.121	2.242	2.586	2.164	6.848	3.079	5.228	1.082454	up	2.36E-07
LOC10704	0	0.035	0.028	0.101	0.109	0.307	0.06	0.046	1.464	0.029	2.781171	up	6.39E-19
SMYD1	1.567	1.467	0.174	1.386	0.571	6.955	15.643	8.655	17.137	14.456	3.6037	up	7.75E-53
CYP26B1	1.016	1.078	0.778	0.472	1.063	0.38	0.941	0.363	0.187	0.212	-1.07931	down	3.63E-06
NAT8	10.089	11.661	7.855	21.419	23.949	5.319	7.278	8.346	4.14	9.259	-1.12628	down	5.87E-08
WDR54	0.918	0.656	1.137	0.754	1.187	3.094	1.132	3.019	1.656	2.516	1.294343	up	2.12E-09
LOC10705	3.023	3.262	3.794	3.719	4.613	9.145	4.478	7.695	11.37	11.55	1.264489	up	1.93E-09
LOXL3	0.558	0.823	1.196	2.502	2.637	0.462	0.529	0.901	0.999	0.797	-1.06399	down	4.76E-07
LOC10175	0.239	0.06	0.263	0.151	0.057	0.213	0.298	0.647	0.565	1.156	1.895803	up	5.49E-13
LOC10704	0.311	0.203	1.238	0.284	1.136	0.167	0.413	0.406	0.213	0.17	-1.20928	down	6.33E-05
LOC10705	0.718	0.515	1.035	0.787	0.738	1.325	1.461	3.439	2.245	1.986	1.461709	up	5.57E-10
TMEM109	8.362	9.296	10.342	13.653	11.82	5.267	5.436	4.253	3.985	3.873	-1.22871	down	1.02E-08
TKFC	32.712	36.02	61.152	39.186	89.191	124.966	49.174	179.497	88.474	82.943	1.023622	up	5.57E-07
DAGLA	1.444	1.387	0.965	0.936	0.73	2.985	2.24	2.854	3.273	3.63	1.45489	up	8.40E-12
TCIRG1	11.835	12.294	15.393	16.677	12.762	34.84	25.986	23.555	46.599	40.265	1.312147	up	2.69E-10
ALDH3B2	0.249	0.296	0.459	0.307	0.352	0.422	0.546	0.997	1.103	1.592	1.483758	up	2.60E-10
GPX2	0.167	0.073	0.619	0.046	0.57	3.279	0.19	4.712	1.196	0.545	2.745761	up	5.14E-23
SPTB	1.182	1.253	2.213	0.42	1.11	0.262	0.33	0.284	0.446	0.233	-1.98676	down	1.12E-18
CDHR5	0.01	0.051	0.185	0.255	0.04	0.169	1.807	0.621	0.329	2.293	3.258182	up	9.76E-40
BUB1B	0.043	0.084	0.136	0.237	0.11	0.248	0.447	3.333	1.161	1.161	3.369234	up	2.10E-39
C15orf52	0.882	0.954	1.441	0.955	1.406	0.462	0.5	0.682	0.637	0.442	-1.04862	down	2.04E-06
KNSTRN	0.253	0.165	0.46	0.831	0.89	1.118	0.956	6.459	1.74	2.42	2.2858	up	5.98E-20
KNL1	0.041	0.033	0.066	0.098	0.113	0.266	0.18	1.686	0.606	0.565	3.21601	up	9.31E-39
B4GALNT4	0.042	0.094	0.181	0.159	0.182	0.335	0.216	1.586	0.943	1.924	2.917442	up	4.61E-33
LOC77061	77.416	82.614	158.739	190.458	73.271	191.412	117.28	957.832	660.941	747.006	2.198919	up	1.31E-23
LOC10705	39.658	45.591	59.649	71.652	51.127	52.271	44.924	214.281	154.567	200.993	1.317255	up	1.36E-09
DHCR7	20.697	21.731	2.016	2.903	33.831	54.228	55.868	35.736	36.913	42.61	1.472982	up	1.35E-12
E2F8	0.192	0.203	0.289	0.412	0.164	0.734	0.42	3.18	1.413	1.458	2.510862	up	1.20E-25
KIF18A	0.137	0.09	0.084	0.139	0.076	0.123	0.171	1.435	0.509	0.605	2.423165	up	6.11E-20
RCN1	5.229	5.745	5.444	5.877	7.058	13.873	7.153	20.248	12.557	11.363	1.151096	up	6.17E-08
PRRG4	1.373	1.063	4.372	1.151	2.014	6.877	7.371	4.867	5.82	5.258	1.59763	up	1.34E-15

DEPDC7	7.603	6.189	6.332	6.463	7.495	1.375	2.333	6.306	2.577	3.716	-1.06329	down	1.34E-07
WEE1	0.803	0.823	1.632	0.729	0.434	0.911	0.758	3.892	1.94	1.702	1.056884	up	1.54E-06
PDE3B	12.752	11.286	18.888	14.52	16.245	4.695	8.524	7.714	7.928	6.352	-1.06527	down	4.73E-07
SAA	18.068	17.014	34.513	38.923	20.811	81.06	254.929	446.752	585.721	734.297	4.023113	up	4.98E-67
TMEM86A	132.445	119.628	82.819	229.35	159.732	23.451	79.908	28.176	19.811	26.596	-2.0245	down	2.62E-21
KCNQ1	1.006	0.859	0.601	0.536	0.536	4.13	1.599	2.566	2.168	1.553	1.762514	up	5.13E-16
TRPM5	0.198	0.373	0.151	0.965	0.144	32.984	18.456	25.121	97.277	57.817	6.979299	up	#######
SYT8	0.099	0.016	0.632	0.041	2.063	0.12	0.424	0.291	0.051	0.122	-1.49536	down	8.55E-07
CTSD	96.128	125.637	250.72	233.262	767.082	105.986	104.823	151.509	114.28	78.341	-1.40818	down	4.65E-11
DUSP8	0.615	0.733	1.304	0.308	0.427	4.803	1.228	4	2.431	4.779	2.346054	up	3.66E-28
PNPLA2	44.613	45.171	100.424	37.369	99.724	19.173	30.229	29.293	34.415	20.515	-1.2924	down	2.49E-09
LRRC56	0.37	0.331	0.069	0.091	0.125	0.646	0.343	1.064	0.582	0.769	1.782392	up	6.98E-15
RNH1	14.679	15.225	17.425	14.465	15.323	48.087	24.109	54.742	28.264	32.942	1.286661	up	7.35E-10
CHKA	20.577	15.45	22.142	9.008	3.506	80.579	27.012	144.932	57.462	74.096	2.441892	up	3.48E-29
TESMIN	0.48	0.358	1.521	0.412	0.316	0.225	0.296	0.367	0.283	0.291	-1.07567	down	2.19E-06
CPT1A	107.933	94.737	63.556	70.728	127.52	12.419	30.583	32.514	14.104	20.449	-2.07714	down	8.77E-23
LOC42885	0.182	0.155	0.015	0.045	0	0.477	2.071	1.69	0.093	0.865	3.693522	up	1.36E-38
PGR2/3	0.309	0.121	0.147	1.826	0.127	0	0.053	0	0.063	0	-4.38891	down	6.91E-17
INCENP	0.18	0.151	0.358	0.32	0.224	0.605	0.369	2.209	0.975	0.816	2.007845	up	3.74E-19
SMIM38	1.844	1.327	0.147	1.819	0.474	0	0	0.12	0	0	-5.48954	down	8.90E-26
FGF19	0.264	0.104	0.196	0.174	0.036	4.264	1.625	0.345	0.596	4.782	3.89847	up	9.82E-53
HSD17B12	62.841	63.515	54.042	59.362	45.414	142.151	113.697	102.739	152.917	174.281	1.265899	up	1.42E-09
C11orf96	12.258	12.187	14.482	13.531	8.476	36.581	20.737	23.755	38.793	26.241	1.261637	up	1.47E-09
ACCS	6.002	4.903	2.753	3.512	1.412	11.417	4.586	9.551	17.767	9.896	1.517729	up	2.84E-12
TSPAN18	1.105	1.835	2.335	1.816	3.187	0.864	1.234	1.166	1.177	0.556	-1.03968	down	1.57E-05
MDK	0.867	1.267	3.207	3.519	3.744	1.259	0.501	0.766	0.837	1.313	-1.42957	down	4.37E-10
CHAC1	25.729	26.957	18.29	109.452	33.208	6.414	7.308	4.187	3.833	3.09	-3.10463	down	6.19E-45
NUSAP1	0.097	0.255	0.245	0.373	0.183	0.707	0.319	5.593	1.414	1.512	3.043865	up	1.91E-33
EHD4	0.987	0.714	1.411	1.028	0.875	1.366	2.268	2.162	3.371	2.718	1.243989	up	2.47E-09
PAPLN	5.324	4.912	26.664	11.121	42.818	5.262	12.975	3.812	3.47	16.885	-1.09902	down	1.48E-06
GALNT16	0.883	0.742	1.649	1.57	0.711	0.435	0.273	0.288	0.344	0.33	-1.73092	down	1.39E-14
RDH11	4.659	5.179	3.221	1.487	5.496	10.546	9.115	9.415	11.673	6.899	1.249181	up	1.92E-09
LGALSL2	0.62	0.766	1.617	0.324	1.405	0.109	0.133	0.092	0.116	0.104	-3.08305	down	1.49E-30
SCG5	0.186	0.191	1.482	1.18	1.433	1.75	1.022	2.743	2.645	3.576	1.390951	up	5.84E-11
LOC11253	0.164	0.063	0.098	0.112	0.028	1.497	0.28	0.179	0.225	0.268	2.384403	up	8.55E-20
G2E3	2.075	1.851	2.57	2.119	2.513	3.713	3.835	5.8	3.614	5.806	1.032482	up	7.07E-07
TTC6	0.807	0.437	2.69	0.156	0.257	0.072	0.075	0.103	0.067	0.046	-3.5639	down	3.06E-45
CLEC14A	2.212	2.435	1.824	0.706	2.085	0.277	0.333	0.408	1.628	0.341	-1.63099	down	1.63E-12

ACSS1B	42.926	41.52	46.602	47.98	76.447	8.772	8.047	12.34	25.364	3.038	-2.14992	down	6.14E-24
RPS6KL1	1.015	1.189	1.327	1.382	1.898	2.714	2.899	4.492	3.202	4.46	1.382609	up	1.30E-10
FLVCR2	0.555	0.363	0.771	0.585	1.293	3.896	0.375	5.677	8.968	0.745	2.4609	up	3.42E-29
DIO2	49.66	52.581	203.045	10.549	71.573	16.609	31.911	8.034	15.972	31.822	-1.8924	down	1.05E-16
GALC	0.376	0.449	0.833	0.893	1.745	0.15	0.307	0.47	0.352	0.401	-1.35192	down	1.48E-09
TTC8	0.979	1.556	2.172	1.494	2.765	4.402	2.031	5.914	4.358	4.661	1.252314	up	3.51E-09
BDKRB1	0.133	0.237	0.303	0.219	0.098	0.129	0.749	8.41	0.352	17.495	4.76958	up	8.69E-74
LOC12111	0.625	0.368	4.008	0.488	0.803	0.575	0.32	0.939	0.353	0.614	-1.16615	down	9.64E-06
AMN	2.966	4.119	6.067	4.949	11.505	1.105	0.526	0.834	0.374	0.112	-3.32442	down	9.76E-44
MTHFD1	168.316	161.333	234.28	152.415	343.279	94.16	150.758	80.269	78.513	84.182	-1.11894	down	2.81E-07
ESR2	5.119	5.07	5.73	3.284	4.446	2.568	2.932	2.155	2.32	1.259	-1.07357	down	5.88E-07
DHRS7	868.149	797.527	1003.922	724.93	1188.741	283.399	440.031	282.936	258.392	308.199	-1.54289	down	5.26E-13
ARMH4	0.126	0.165	0.078	0.075	0.006	0.222	1.201	0.187	5	0.202	3.905199	up	6.64E-56
DLGAP5	0.257	0.168	0.303	0.322	0.349	1.236	0.589	5.096	2.218	1.541	2.927972	up	4.43E-35
WDHD1	0.336	0.555	0.267	0.74	0.653	1.026	0.988	2.007	1.314	1.546	1.429778	up	2.78E-10
GCH1	87.373	94.623	99.629	67.794	186.64	50.207	44.554	48.082	39.401	31.555	-1.32612	down	4.96E-10
SOS2	9.302	9.379	11.862	14.758	15.676	5.778	5.354	7.972	5.054	5.466	-1.04137	down	4.51E-07
LOC11253	1.73	2.019	1.973	1.191	1.345	0.86	0.958	0.366	0.576	1.032	-1.12181	down	8.41E-06
LOC12110	0.982	0.989	1.19	1.01	0.727	0.445	0.355	0.612	0.181	0.68	-1.1059	down	3.58E-07
GRID1	0.591	0.53	1.061	0.147	0.01	0.178	0.064	0.115	0.117	0.067	-2.102	down	3.17E-20
SNCG	0.037	0.016	0.07	0.041	1.68	0.024	0.043	0.033	0.026	0.041	-3.42626	down	1.24E-25
DNAJC12	47.04	41.616	64.729	49.829	55.959	14.665	18.708	29.367	24.901	14.621	-1.3416	down	1.24E-10
LOC10174	0.43	0.384	1.682	0.08	0.121	0.152	0.067	0.383	0.14	0.368	-1.27698	down	1.92E-06
CDK1	1.008	0.716	8.736	0.805	1.597	3.067	1.919	15.684	6.558	6.641	1.396503	up	7.50E-12
RNASE6	2.549	3.117	6.954	5.151	1.073	1.214	3.748	15.446	12.688	19.615	1.483753	up	1.47E-11
PAPSS2	103.418	92.74	139.544	99.126	136.152	33.473	53.912	61.278	62.281	46.545	-1.14891	down	3.95E-08
ASAH2	0.194	0.184	2.521	0.231	0.199	0.051	0.18	0.023	0.108	0.144	-2.70586	down	7.83E-20
DNA2	0.034	0.022	0.071	0.157	0.161	0.347	0.316	2.006	0.679	0.559	3.119909	up	5.62E-32
HKDC1	0.042	0.172	0.528	0.207	0.076	39.419	0.728	15.26	55.847	49.843	7.289186	up	#######
CYP2C18	1704.444	1566.283	682.717	855.24	1306.675	462.707	250.504	59.89	390.887	296.202	-2.06628	down	3.52E-22
SCD	143.146	170.585	16.981	51.148	16.442	1466.008	776.956	1597.615	983.375	993.037	3.868322	up	1.37E-61
CYP2C23b	588.344	527.607	295.271	352.718	570.35	125.584	185.273	54.356	159.591	132.218	-1.82896	down	7.71E-18
CYP2C23a	1174.55	1044.819	1141.807	758.226	1003.909	376.82	430.244	172.944	231.894	326.531	-1.73561	down	7.68E-16
MSMB	5.047	4.939	8.814	1.882	4.879	18.481	12.064	9.916	9.397	21.087	1.472576	up	5.34E-13
C10orf128	2.225	2.306	4.657	3.415	2.953	4.386	5.046	9.028	5.373	8.905	1.073252	up	3.18E-07
ANKRD22	0.151	0.049	0.06	0.248	0.039	1.022	0.161	2.561	0.348	0.586	3.084693	up	3.87E-21
ACTA2	7.16	6.919	10.318	9.822	12.616	19.974	20.902	17.823	27.431	22.881	1.218727	up	2.64E-09
IFIT5	26.904	24.599	64.378	48.686	21.755	22.134	17.22	449.399	240.364	388.146	2.584067	up	1.64E-31

PANK1	87.26	75.486	129.212	87.423	116.202	40.763	45.939	55.766	35.681	31.272	-1.2427	down	4.42E-09
KIF11	0.544	0.55	1.01	0.769	0.515	1.113	0.867	3.749	1.587	1.83	1.431368	up	3.08E-11
CPN1	48.282	50.567	48.407	36.398	76.073	18.958	19.938	29.987	22.17	14.471	-1.29938	down	4.69E-10
LOXL4	0.866	0.908	1.01	0.969	0.905	0.231	0.357	0.351	0.337	0.33	-1.5333	down	5.45E-11
CRTAC1	6.912	8.008	0.079	0.244	0.017	0.192	1.282	0.086	0.034	0.04	-3.21934	down	5.76E-41
PI4K2A	22.469	22.979	17.273	12.637	18.764	8.199	10.699	10.347	9.084	7.242	-1.04633	down	4.55E-07
HOGA1	4.35	4.49	2.699	2.135	5.657	1.477	1.182	0.148	0.84	0.785	-2.12363	down	8.14E-20
ANKRD2	0.61	0.412	1.112	0.388	0.283	0.191	0.286	0.128	0.161	0.29	-1.40515	down	2.17E-07
ARHGAP1	0.13	0.061	0.133	0.129	0.133	0.278	0.214	1.573	0.58	0.621	2.468502	up	1.11E-22
PROM1L	4.272	4.011	6.877	4.973	4.388	6.575	4.708	15.206	18.097	13.489	1.243729	up	4.56E-09
TLX1	0.975	1.072	0.882	0.947	0.046	0	0	0	0	0	-9.61728	down	2.06E-36
STN1	0.283	0.516	0.439	0.869	1.469	0.577	0.713	4.299	0.932	2.473	1.329402	up	1.08E-08
SFR1	0.095	0.173	0.252	0.113	0.152	0.235	0.506	1.201	0.531	0.717	2.015891	up	1.21E-16
GPAM	50.126	44.294	35.149	35.867	21.312	204.289	133.023	88.406	96.715	203.872	1.959454	up	1.99E-20
RGS10	1.07	1.013	2.254	1.218	0.63	1.403	1.18	5.24	4.973	6.288	1.62473	up	5.29E-14
LOC10175	0.605	0.138	0.27	0.482	0.762	0.559	1.424	0.694	2.406	0.195	1.223758	up	1.59E-08
CPXM2	1.089	1.906	1.418	1.687	3.264	0.128	0.251	0.5	0.717	1.095	-1.79707	down	4.38E-17
FAM196A	0.873	1.004	4.073	1.217	0.36	0.105	0.642	0.191	0.48	0.372	-2.06905	down	8.66E-20
MKI67	0.168	0.176	0.41	0.273	0.327	1.005	0.879	6.126	2.364	2.301	3.221937	up	1.79E-44
ALDH18A1	4.614	5.091	9.894	5.147	5.39	26.732	11.79	27.272	14.304	13.409	1.633424	up	2.57E-15
FRZB	56.751	58.945	50.548	40.422	64.142	16.858	35.065	20.456	29.566	20.843	-1.14107	down	5.89E-08
BARD1	0	0.029	0.062	0.055	0.091	0.172	0.104	1.274	0.591	0.326	3.3526	up	5.63E-31
ABCA12	5.11	4.197	11.634	1.146	27.632	0.113	0.887	0.648	0.258	0.205	-4.55453	down	2.09E-79
ATIC	4205.008	4235.959	2567.995	3001.741	2519.42	528.741	871.653	499.368	566.56	377.477	-2.5392	down	8.31E-32
LOC10704	2.429	8.065	4.108	4.732	2.095	0.958	0.282	0.548	1.231	0.662	-2.53977	down	1.99E-28
UGT1A1	39.561	37.223	44.172	46.488	71.827	24.713	33.166	21.847	18.61	16.829	-1.05491	down	7.94E-07
LOC12111	5.572	5.416	23.484	11.677	68.075	1.257	4.033	2.28	1.699	1.574	-3.39643	down	1.75E-48
LOC12111	3.342	3.479	3.634	2.096	4.724	21.468	4.113	44.068	4.644	3.32	2.167288	up	9.11E-23
TRAF3IP1	0.813	0.71	1.363	1.003	1.271	2.363	1.448	3.807	1.877	2.592	1.227213	up	1.61E-08
AHR1B	4.608	3.848	6.309	2.694	3.261	1.633	1.536	1.419	0.894	1.363	-1.5972	down	2.58E-13
STAT1	19.896	18.228	25.649	25.151	15.937	26.032	24.54	101.086	84.281	112.561	1.732632	up	1.18E-15
COQ10B	31.709	32.659	22.38	37.282	58.093	8.021	13.044	19.284	14.27	6.618	-1.57236	down	3.43E-14
AOX1	0.818	0.819	1.758	1.008	6.053	0.371	1.252	1.216	1.561	0.463	-1.10362	down	5.84E-07
ICOS	1.272	0.542	1.615	0.572	0.357	0.999	0.974	1.63	2.56	3.415	1.135157	up	9.58E-08
CTLA4	0.131	0.315	0.278	0.036	0	1.397	0.187	0.857	0.852	1.645	2.691855	up	1.16E-18
LOC42411	16.941	16.13	19.558	15.841	26.377	7.379	9.626	7.243	6.558	6.34	-1.35228	down	2.24E-10
IDH1	256.182	246.123	290.413	244.149	481.266	123.379	172.331	129.857	118.137	97.782	-1.2428	down	5.44E-09
PIKFYVE	8.55	7.751	16.078	7.097	11.397	3.971	4.774	4.697	6.174	4.729	-1.06312	down	8.21E-07

PDE11A	19.234	16.791	0.348	4.895	0.061	42.799	40.116	0.113	38.233	22.5	1.798322	up	5.46E-17
CDCA7	0.208	0.484	1.145	0.514	0.696	1.625	1.255	7.46	2.341	2.94	2.356122	up	4.37E-26
RAPGEF4	21.935	20.538	17.359	11.14	36.575	4.99	12.29	10.219	11.653	6.107	-1.2486	down	3.02E-09
SPC25	0.417	0.448	0.332	0.441	0.949	1.327	1.094	9.299	2.72	2.728	2.728004	up	1.64E-28
IFIH1	5.446	4.954	7.47	6.273	6.549	6.679	6.468	33.193	21.698	28.888	1.65886	up	1.70E-14
DPP4	4.234	3.722	3.601	5.071	9.228	16.259	14.302	12.348	14.979	20.046	1.591567	up	2.50E-14
SLC4A10	0.187	0.138	0.278	0.215	0.281	1.465	0.766	1.578	0.69	1.074	2.337008	up	2.26E-25
ITGB6	0.971	0.877	0.5	0.447	0.438	5.151	0.877	1.356	3.667	1.276	1.929232	up	2.60E-19
LOC42419	0.762	0.715	1.71	0.465	1.364	0.019	0.133	0	0.2	0.048	-3.63198	down	9.62E-35
IHH	4.952	4.974	10.832	8.392	14.985	7.362	2.405	4.106	4.595	2.403	-1.08024	down	9.03E-07
VIL1	0.57	0.444	0.687	0.481	0.776	0.373	0.513	1.087	5.09	0.995	1.444258	up	1.52E-10
IGFBP2	19.814	18.98	11.072	14.401	21.131	2.387	1.93	1.238	0.551	1.266	-3.53318	down	8.97E-54
ARHGEF1	0.476	0.488	0.145	0.235	0.179	2.344	0.8	2.861	1.84	2.856	2.808703	up	2.44E-33
EAF2	6.465	6.932	2.603	3.063	2.765	15.629	6.389	16.196	13.713	14.647	1.608557	up	1.30E-13
SEMA5B	1.118	1.356	3.459	4.95	3.75	1.106	1.588	1.212	1.247	1.492	-1.13829	down	8.15E-08
MYLK	24.439	24.724	27.659	15.53	18.65	5.682	7.096	9.657	14.769	12.594	-1.15635	down	1.87E-08
SLC12A8	0.247	0.246	0.306	0.554	0.298	2.841	1.625	2.562	0.429	1.723	2.471577	up	9.55E-28
PARP9	7.544	7.86	11.286	9.896	8.931	9.141	9.723	31.187	18.758	26.105	1.060133	up	7.53E-07
SCTR	4.74	4.324	0.287	2.085	0.549	5.121	7.871	3.07	3.918	4.292	1.017758	up	3.39E-06
DPP10	0.176	0.291	0.436	0.244	0.029	0.153	1.72	2.921	0.171	0.121	2.10794	up	1.58E-19
MCM6	0.647	0.586	0.684	0.873	1.889	1.781	1.326	6.675	2.256	2.827	1.666592	up	3.50E-14
KIF5C	0.354	0.314	0.664	0.557	0.308	0.923	0.763	1.175	0.763	1.844	1.313517	up	9.77E-10
NMI	9.6	9.662	15.516	11.147	9.809	16.617	12.33	42.511	28.653	43.043	1.360859	up	1.51E-10
UPP2	40.223	37.093	61.261	47.83	61.272	5.867	27.944	3.535	11.75	10.789	-2.04811	down	1.74E-20
NTNG1	1.586	1.549	4.667	1.87	2.384	0.712	0.883	0.66	0.766	0.883	-1.62548	down	8.97E-13
ATP6V1G3	0.273	0.208	0.417	0.168	0.514	1.013	1.477	2.793	0.933	1.246	2.236046	up	2.33E-20
ASPM	0.611	0.639	0.406	1.148	1.133	0.913	1.13	2.939	1.716	1.429	1.044682	up	2.74E-06
HTATIP2	4.119	4.442	3.439	3.446	5.307	5.197	6.397	15.356	10.243	5.777	1.04983	up	1.53E-06
REG4	0.831	0.502	1.374	0.473	0.853	0.124	0.709	0.334	0.197	0.418	-1.1761	down	0.000271
PHGDH	584.736	604.741	549.887	746.551	656.436	237.112	474.513	238.139	239.985	197.048	-1.18008	down	2.09E-08
LOC10085	2.765	2.605	3.779	1.831	1.208	1.718	1.798	1.428	0.233	0.675	-1.05782	down	3.87E-06
LOC10705	0.051	0.033	0.067	0.083	0.104	0.049	0.029	5.748	3.455	5.785	5.457423	up	4.95E-79
FASLG	0.394	0.358	0.569	0.236	0.405	0.518	0.691	1.492	1.117	1.251	1.367127	up	3.86E-10
SUCO	6.512	6.015	8.369	5.144	6.028	31.539	11.904	23.772	21.302	27.42	1.853972	up	1.02E-18
FMO3	553.076	547.561	600.864	963.532	1308.063	255.106	677.339	257.859	241.41	251.845	-1.23875	down	7.89E-09
NUF2	0.068	0.122	0.256	0.306	0.226	0.262	0.26	2.853	1.164	0.955	2.483906	up	3.81E-22
CENPL	0.274	0.055	0.235	0.121	0.173	0.163	0.361	2.095	0.866	0.552	2.227637	up	1.69E-16
RGS8	6.23	6.334	1.454	1.004	4.903	0.382	0.631	1.055	0.783	1.158	-2.31183	down	8.21E-28

GLUL	216.185	224.988	358.723	324.283	678.3	184.058	236.944	157.46	136.126	96.509	-1.15203 down	1.17E-07
SOAT1	7.702	6.988	20.825	8.325	17.535	3.941	4.321	5.28	6.603	5.11	-1.28091 down	3.75E-09
ABL2	2.044	1.783	3.941	1.973	2.067	9.222	4.961	5.831	4.736	12.143	1.643167 up	1.15E-15
TOR3A	22.63	20.597	13.745	7.774	1.6	80.999	69.46	76.931	59.746	87.624	2.497796 up	2.03E-30
SERPINC1	259.983	261.638	354.37	334.689	510.496	165.38	191.467	162.796	173.345	166.088	-1.00253 down	2.16E-06
NCF2	0.484	0.736	0.834	1.156	1.75	1.574	0.988	2.653	1.876	3.514	1.095559 up	6.85E-07
PLPPR5	8.421	7.441	3.569	4.29	3.863	16.102	11.31	13.194	12.059	11.177	1.210526 up	1.20E-08
MTF2	8.227	7.696	8.515	8.218	4.423	19.759	7.661	15.188	17.611	16.647	1.051643 up	5.44E-07
CLCA1	0.1	0.033	0.03	0.164	0.026	0.895	3.499	10.75	24.316	10.765	7.132446 up	#######
SSX2IP	2.355	1.78	1.613	1.374	0.639	6.274	3.356	5.673	5.783	6.316	1.819301 up	4.53E-17
VTG2	4938.704	5884.15	43.669	1565.017	4.114	11764.71	9198.474	8303.704	10016.63	9485.489	1.971482 up	4.27E-19
LOC12111	3.51	7.943	0	1.355	0	75.781	12.21	32.906	33.385	13.798	3.713511 up	3.69E-57
VTG3	625.379	748.039	2.05	89.471	0.449	2248.1	1331.339	1043.224	1386.54	1479.229	2.353377 up	1.75E-26
SPATA1	21.159	19.818	12.843	23.628	2.939	45.134	34.228	39.319	33.832	45.597	1.301214 up	1.50E-09
THAP10	0.586	0.516	0.304	0.223	0.166	0.805	0.696	0.909	1.254	1.229	1.444196 up	1.87E-10
VTG1	2389.735	3147.829	3.494	185.475	1.276	6936.695	4641.689	5493.699	5527.128	5666.104	2.302976 up	3.89E-25
KIF2C	0.474	0.276	0.561	0.347	0.381	0.794	0.416	4.148	0.959	1.288	1.896501 up	5.50E-16
MMACHC	5.189	4.085	8.857	3.444	4.665	2.996	2.474	2.278	2.164	3.082	-1.01364 down	4.76E-06
RBP	330.724	399.221	17.878	55.352	2.696	871.222	773.958	596.107	813.063	1046.719	2.347372 up	3.16E-26
CYP4A22	50.13	52.443	87.296	66.467	105.941	22.721	42.975	25.39	20.393	28.634	-1.37047 down	2.19E-10
CYP4B7	400.359	358.574	324.755	325.616	466.218	55.539	113.522	98.105	71.428	50.003	-2.27093 down	3.78E-26
STIL	0.048	0.016	0.095	0.072	0.139	0.254	0.129	1.496	0.563	0.65	3.045909 up	2.63E-32
PODN	2.283	1.903	5.682	1.607	1.414	0.366	0.757	0.461	0.442	0.54	-2.3263 down	2.98E-25
DHCR24	22.675	25.84	0.604	0.787	51.673	38.625	61.319	23.086	37.321	44.774	1.013865 up	4.96E-07
DAB1	0.954	0.815	3.121	1.119	2.971	0.212	0.452	0.413	0.567	0.512	-2.05582 down	9.22E-20
TACSTD2	1.413	1.345	2.08	0.809	0.924	0	0.329	0.28	0.615	0.21	-2.19214 down	1.70E-15
ANGPTL3	47.918	48.945	26.193	51.599	48.217	269.748	304.769	238.462	239.045	308.813	2.61018 up	5.91E-33
PGM1	39.663	40.911	36.779	56.112	46.143	144.432	65.339	166.757	87.819	84.931	1.322586 up	4.56E-10
DNAJC6	2.165	1.793	2.369	0.473	3.857	3.321	3.259	10.769	1.344	5.541	1.184852 up	1.75E-08
GADD45A	1.483	1.698	4.369	2.803	4.493	0.646	1.33	1.646	1.065	1.758	-1.20319 down	1.32E-06
DEPDC1	0.154	0.236	0.266	0.19	0.344	0.212	0.176	1.462	0.765	0.631	1.443873 up	6.11E-09
NEU2	1.117	0.817	0.314	0.576	0.788	1.506	3.013	0.858	2.674	1.218	1.358398 up	1.27E-09
KLHL24	23.395	22.179	23.13	14.804	19.244	7.391	9.498	7.657	6.731	5.81	-1.47006 down	4.17E-12
EHHADH	274.411	257.616	291.099	309.829	356.991	41.899	104.486	52.69	44.826	41.111	-2.38615 down	6.12E-28
DNAJB11	20.71	21.364	20.588	17.848	19.015	53.129	30.368	47.853	31.35	43.746	1.052596 up	4.00E-07
AMOTL2	3.166	3.356	4.378	5.043	6.719	1.058	2.202	2.453	3.21	2.258	-1.0189 down	1.10E-06
TM4SF19	0.466	0.638	1.373	0.534	0.726	0.684	0.616	1.403	0.826	3.952	1.000385 up	3.10E-06
PER2	31.449	25.95	43.329	19.482	12.35	10.298	11.607	9.341	17.234	12.76	-1.11404 down	1.53E-07

CLSTN2	0.41	0.553	1.235	1.126	0.979	0.26	0.428	0.213	0.179	0.18	-1.76788	down	2.29E-15
PXYLP1	2.951	2.654	2.915	4.503	1.882	7.548	5.664	7.938	6.598	7.357	1.235602	up	5.88E-09
SGPP2	0.885	0.962	0.263	1.936	3.467	0.741	0.115	0.392	0.2	0.184	-2.19929	down	5.99E-21
MOGAT1	1.422	1.726	2.128	2.632	8.621	0.431	1.068	1.767	0.896	1.443	-1.55936	down	9.01E-13
GK5	17.146	17.654	25.66	10.469	12.64	3.29	9.91	5.421	13.117	5.477	-1.16698	down	5.42E-08
PCOLCE2	2.885	2.765	3.479	2.576	4.171	0.977	0.953	1.597	1.133	0.621	-1.58705	down	1.71E-13
PLOD2	13.752	12.993	15.679	16.286	8.696	57.876	35.403	51.905	46.696	42.685	1.798963	up	1.81E-17
LOC12111	17.118	17.826	22.987	18.719	32.738	4.763	7.469	4.818	21.024	10.279	-1.17769	down	2.10E-08
BDH1B	4.741	5.446	4.386	2.933	11.844	1.863	2.132	0.947	1.33	0.992	-2.01378	down	1.26E-19
HRASLS	2.403	2.567	1.734	5.152	3.432	9.18	6.606	8.165	4.872	13.248	1.460126	up	1.54E-11
RTP2	5.707	6.898	5.74	12.564	6.633	2.24	6.928	2.079	1.91	2.456	-1.26549	down	5.09E-09
PDCD1	0.224	0.244	0.436	0.245	0.332	0.554	0.467	1.332	1.582	1.973	1.992456	up	1.53E-15
C2orf72	45.018	46.835	41.902	41.539	44.64	18.314	29.59	19.748	18.355	19.066	-1.06564	down	3.68E-07
CLCN2	5.259	5.023	6.373	7.091	8.974	2.587	4.328	2.657	3.037	3.543	-1.01823	down	1.88E-06
AHSG	647.406	586.191	341.982	385.887	516.601	3584.804	1226.355	3155.554	2948.166	2892.059	2.478104	up	6.50E-30
FETUB	129.897	121.696	142.33	117.69	145.302	312.127	336.231	432.966	234.873	187.893	1.195106	up	6.30E-09
ECT2	0.326	0.373	0.202	0.082	0.205	0.535	0.488	3.337	1.087	1.03	2.441845	up	1.67E-24
LOC10705	0.51	0.516	1.065	0.257	2.652	0.25	0.159	0.594	0.467	0.538	-1.31402	down	2.45E-09
SMC4	0.936	0.823	1.281	0.925	1.01	2.986	3.127	11.515	2.94	5.477	2.387066	up	3.30E-27
IFT80	2.329	2.327	3.494	2.548	2.656	16.023	8.297	12.273	3.817	10.785	1.938332	up	3.13E-20
MLF1	1.58	1.77	10.575	6.262	8.683	2.79	1.537	2.522	1.845	2.165	-1.41027	down	2.30E-10
PTX3	0.048	0	0.399	0.059	0.025	0.092	0.51	2.681	0.098	0.547	2.875325	up	3.29E-23
ARHGEF26	23.545	19.621	29.884	21.264	16.511	7.316	11.872	7.522	19.253	8.36	-1.02858	down	1.06E-06
SUCNR1	10.112	8.721	20.68	8.036	5.365	3.945	3.016	2.92	1.886	3.178	-1.82364	down	1.58E-16
AADAC	72.642	74.888	113.183	132.934	105.013	43.354	71.467	34.777	50.392	48.44	-1.0052	down	2.24E-06
DUOX1	0.118	0.177	0.05	0.051	0	3.97	1.798	0.479	0.938	1.976	4.514618	up	1.71E-70
KIAA0101	2.021	2.952	1.287	0.996	2.421	4.696	4.775	7.126	5.804	6.279	1.56692	up	1.34E-11
PATL2	0.431	0.311	0.936	0.477	0.321	0.72	0.851	5.692	2.624	2.907	2.367037	up	9.50E-25
TERB2	139.647	144.533	246.722	179.339	133.526	226.065	273.892	447.205	412.411	399.09	1.059558	up	3.05E-07
CD276	1.304	1.262	1.756	1.389	1.793	4.056	2.95	4.655	3.355	4.192	1.35539	up	2.32E-10
CYP1A2	186.343	193.424	80.067	111.37	352.381	47.473	253.701	15.727	82.093	23.882	-1.127	down	4.17E-07
STOML1	1.689	2.009	3.074	2.163	1.329	1.295	2.286	12.289	5.669	8.77	1.561689	up	1.96E-12
PMLL	1.652	1.926	2.885	2.058	1.656	1.883	1.707	10.04	6.847	8.65	1.516584	up	3.90E-12
LOC10705	0.901	0.786	0.867	0.617	1.042	1.164	0.674	4.71	3.583	3.471	1.689718	up	1.78E-12
CHRNA3	0.746	0.418	1.464	0.81	0.164	0.465	0.364	0.209	0.164	0.131	-1.43072	down	1.96E-07
HYKK	5.999	6.293	7.576	4.861	6.118	3.383	2.571	2.612	2.138	2.875	-1.18346	down	4.38E-08
IDH3A	5.608	4.443	16.134	12.166	9.347	35.713	8.9	24.741	13.805	14.456	1.033097	up	2.04E-07
TRPM1	0.314	0.362	0.075	0.487	0.137	1.451	3.361	3.059	1.598	4.392	3.328811	up	1.86E-46

CCNB1	1.182	1.152	2.823	1.149	1.659	1.845	1.895	12.567	3.661	4.259	1.604262	up	4.35E-13
LIPC	10.231	9.369	17.981	17.582	21.798	0.864	1.206	2.21	1.08	3.05	-3.19319	down	2.44E-46
SCG3	0.277	0.258	0.134	0.235	0.216	0.512	0.66	1.097	0.681	0.494	1.616253	up	6.56E-12
SEMA6D	5.433	4.993	4.704	5.885	5.547	2.672	2.202	2.869	2.153	1.773	-1.18634	down	9.12E-09
DUT	1.237	1.425	1.067	1.419	2.435	2.346	1.739	6.21	2.842	3.088	1.096871	up	6.95E-07
FBN1	1.163	1.455	1.829	1.864	2.128	2.917	4.199	3.629	3.305	4.336	1.123001	up	4.84E-08
TM6SF1	18.945	17.685	25.32	26.036	25.066	52.969	41.051	54.168	36.351	47.637	1.0382	up	4.43E-07
LOC41547	0.36	0.319	0.574	0.463	0.388	0.788	0.496	1.131	0.65	1.34	1.06422	up	8.87E-07
PEX11A	28.78	25.19	13.222	20.54	14.935	6.635	17.476	9.738	5.771	11.509	-1.00569	down	8.64E-07
TICRR	0.543	0.639	0.509	0.504	0.623	0.6	0.846	2.428	1.048	1.191	1.115831	up	6.41E-07
MFGE8	138.896	146.087	39.039	75.693	26.068	314.031	195.808	210.483	264.593	238.075	1.522211	up	1.83E-12
ARRDC4	4.512	4.463	4.245	5.502	10.039	14.702	6.659	14.803	15.595	15.58	1.227185	up	4.85E-09
PGPEP1L	6.832	6.269	12.13	11.861	8.377	1.865	2.891	1.346	2.342	2.106	-2.10712	down	3.90E-22
LRRK1	0.549	0.449	0.521	0.529	0.331	0.762	0.685	1.096	1.191	1.429	1.116222	up	3.02E-07
PCSK6	10.296	9.946	18.096	13.867	8.212	31.96	18.994	21.468	27.297	24.05	1.034562	up	2.98E-07
RASL12	2.324	2.354	2.276	1.811	2.211	0.984	1.294	0.742	1.219	1.183	-1.01678	down	4.24E-06
CORO2B	0.557	0.608	0.694	4.147	1.16	0.872	0.586	0.485	0.704	0.9	-1.01354	down	1.40E-06
PAQR5	1.348	1.26	1.339	1.931	1.86	0.373	0.592	1.073	0.685	0.599	-1.21867	down	1.12E-08
KIF23	0.095	0.124	0.14	0.258	0.15	0.63	0.375	6.196	3.809	1.195	3.983319	up	8.17E-58
TMED3	10.988	11.981	10.281	14.372	11.035	28.906	17.234	31.255	17.346	24.161	1.019335	up	1.63E-06
PRC1	1.274	1.246	2.64	0.912	1.787	3.887	2.036	9.912	2.825	3.641	1.504097	up	1.66E-12
ADGRG5	0.471	0.483	1.2	0.387	0.629	0.532	0.644	1.865	1.948	1.425	1.015592	up	3.94E-06
CETP	25.686	27.129	125.438	13.017	74.071	2.095	0.458	4.278	0.591	0.958	-4.98392	down	1.58E-90
LCAT	92.519	82.067	354.367	283.575	528.767	26.533	69.44	38.128	41.104	28.522	-2.71889	down	4.84E-34
LOC41566	48.739	55.791	52.034	72.387	96.728	21.717	54.896	19.98	20.127	19.971	-1.2525	down	5.66E-09
LOC10174	652.428	670.99	572.533	1307.915	921.024	169.097	200.749	170.304	165.711	220.825	-2.1542	down	2.34E-24
LOC11253	29.432	32.367	17.144	21.458	6.47	16.635	6.554	4.338	4.347	4.308	-1.56239	down	9.89E-13
SETD6	20.448	20.309	9.756	6.154	7.196	4.578	4.009	5.274	8.16	6.844	-1.14552	down	1.37E-08
MT4	711.399	809.728	1038.719	734.886	291.299	29.124	282.326	311.005	120.404	43.863	-2.18845	down	9.78E-25
MT3	310.257	378.472	491.141	215.847	100.131	6.013	84.865	126.248	47.135	8.124	-2.45722	down	4.83E-30
SLC6A2	2.224	2.327	2.448	1.587	1.529	6.855	4.049	4.837	4.194	5.144	1.309558	up	2.37E-10
GPT2	193.86	180.774	302.476	147.878	604.144	62.765	184.412	54.181	86.554	72.107	-1.63536	down	1.36E-13
LOC10085	3.081	2.797	4.203	2.885	2.036	2.66	2.203	18.466	12.837	20.487	1.916644	up	3.67E-18
RGS9BP	1.13	1.118	2.26	1.351	2.091	0.506	0.542	0.768	0.613	0.718	-1.33559	down	1.58E-09
PDCD2L	9.731	8.954	15.511	6.181	15.106	12.39	18.416	32.671	17.868	30.129	1.006524	up	8.81E-07
CES1L2	38.947	39.648	81.674	126.021	111.344	14.516	16.189	1.232	21.673	1.244	-2.85766	down	2.24E-37
CES1L1	111.626	109.5	127.726	130.396	178.969	16.118	18.967	5.638	56.824	2.884	-2.7123	down	4.98E-35
TERB1	3.686	3.169	6.137	3.53	1.611	1.431	1.314	1.054	1.25	0.735	-1.64763	down	1.32E-13

BEAN1	0.894	0.509	0.588	0.831	0.599	1.054	0.764	1.574	3.311	1.081	1.184913	up	1.89E-06
CMC2	1.814	1.737	1.986	2.023	1.73	4.203	3.458	4.992	3.954	4.529	1.185517	up	6.23E-08
CENPN	0.316	0.447	0.405	0.375	0.612	1.765	1.018	4.717	2.122	1.835	2.407756	up	1.74E-24
LOC12111	0.38	0.345	0.482	0.407	0.357	1.771	1.452	0.505	0.766	2.717	1.868616	up	3.45E-18
GINS2	0.197	0.297	0.676	0.237	0.436	0.66	0.414	2.277	1.213	0.992	1.58938	up	7.16E-12
SLC7A5	13.905	12.708	34.749	16.312	13.231	5.619	10.961	8.003	5.724	5.971	-1.32515	down	1.49E-09
CA5A	214.2	216.023	174.242	126.055	187.64	34.656	78.483	38.658	40.801	31.204	-2.0365	down	2.79E-21
CIDEC	25.144	27.625	46.895	23.439	53.649	15.399	19.028	18.231	13.011	15.298	-1.12627	down	2.10E-07
MVD	13.917	17.885	4.657	2.51	30.493	27.585	27.121	23.292	37.123	27.48	1.037635	up	4.93E-07
CDT1	0.437	0.767	1.142	0.632	1.276	2.727	1.494	10.715	3.571	3.075	2.341576	up	4.76E-25
FANCA	0.209	0.233	0.256	0.093	0.17	0.463	0.268	1.409	0.491	0.391	1.647794	up	5.87E-12
SPIRE2	9.4	10.136	19.47	2.665	10.706	4.208	4.788	0.596	3.844	1.378	-1.82162	down	1.25E-15
CHTF8	0.194	0.153	0.062	0.128	0.28	0.64	0.233	2.032	1.476	1.081	2.733539	up	7.51E-20
PMFBP1	0.335	0.328	0.419	0.384	0.377	1.338	1.391	1.75	0.705	0.976	1.738136	up	1.48E-16
LOC12111	0.211	0.11	0.134	0.277	0.477	3.064	2.631	3.086	0.692	1.759	3.210417	up	1.39E-32
NT5DC2	16.51	16.917	14.552	32.542	49.533	7.226	6.546	15.59	11.997	2.69	-1.56182	down	3.80E-14
SMIM4	37.41	36.755	18.075	56.563	44.851	10.636	12.737	19.041	12.837	5.335	-1.67634	down	5.64E-16
MUSTN1	1.16	0.901	1.501	1.907	1.043	0.421	0.186	0.189	0.446	0.237	-2.13472	down	4.16E-12
PRKCD	1.569	1.476	2.385	1.534	3.023	2.751	2.97	4.527	4.671	5.611	1.03924	up	5.44E-07
GBP4L	2.605	3.432	8.426	5.351	3.121	6.025	4.187	14.482	9.482	17.484	1.171322	up	2.45E-08
LOC1211C	2.474	2.67	6.941	1.457	0.379	0.465	0.631	2.436	0.947	1.057	-1.32956	down	1.33E-09
LOC1211C	0.236	0.288	1.114	0.723	0.129	0	0.107	0.205	0.355	0	-1.89251	down	6.10E-13
GBP	3.028	3.094	19.819	18.221	3.153	2.872	3.384	6.393	7.851	0	-1.20647	down	1.46E-08
LOC1211C	0.681	0.603	2.711	2.173	0.37	0	0.411	0.733	0.986	0	-1.61572	down	2.23E-10
GMPPB	15.331	18.192	17.541	15.616	16.166	36.277	23.724	46.976	33.199	36.723	1.094376	up	2.11E-07
MST1R	0.552	0.61	1.135	0.754	0.866	9.933	1.81	5.369	3.069	6.686	2.776442	up	3.91E-37
SLC38A3	80.991	85.356	144.322	107.206	236.443	31.804	83.851	53.068	60.815	53.081	-1.21112	down	1.82E-08
OASL	7.621	9.268	20.363	15.717	5.168	9.555	5.612	191.286	93.233	183.482	3.054887	up	8.12E-42
ATP2B2	0.413	0.45	0.052	0.076	0.057	1.664	0.131	0.522	0.219	0.239	1.400579	up	5.23E-10
CECR5L	4.502	3.983	10.851	4.028	3.394	15.107	7.833	9.356	10.191	11.688	1.017521	up	1.87E-07
DNASE1L3	5.72	5.065	11.301	8.801	1.546	8.579	15.246	35.783	26.251	39.567	1.951137	up	2.38E-19
ABHD6	87.008	72.637	40.937	70.693	36.2	16.073	27.449	19.588	11.009	29.879	-1.56387	down	3.01E-14
IP6K2	70.166	60.534	91.078	45.614	15.293	11.922	22.396	21.623	14.824	38.22	-1.37503	down	4.99E-11
MCM2	1.357	1.178	1.039	1.587	1.152	2.156	1.553	6.316	3.542	2.667	1.361923	up	9.18E-10
CFAP100	0.785	0.514	1.225	0.417	0.571	0.127	0.321	0.15	0.101	0.294	-1.81723	down	2.47E-12
NTN4L	0.253	0.231	0.253	0.13	0.252	0.238	0.191	1.263	0.97	1.227	1.792611	up	1.25E-14
PRRT3	2.035	2.402	2.542	2.585	2.979	1.234	0.899	0.55	0.972	0.909	-1.45751	down	3.40E-11
FAM3D	0.547	0.975	0.363	0.225	0.125	1.06	1.273	1.152	2.215	0.646	1.503485	up	2.14E-09

C3orf67	0.125	0.057	0.03	0	0.038	0.241	0.011	1.694	0.473	0.928	3.716453	up	9.84E-37
GPR27	0.309	0.392	0.087	0.463	0.149	1.25	0.994	2.018	0.951	1.323	2.218941	up	6.76E-19
PROK2	0.259	0.207	0.046	0.118	0	1.17	2.137	1.765	0.678	1.881	3.587989	up	1.64E-36
BHLHE40	14.579	15.981	27.992	29.928	54.212	9.245	12.417	13.634	8.009	24.714	-1.06884	down	3.71E-07
HBEGF	30.34	31.798	43.511	22.292	20.809	8.677	18.263	14.757	15.637	12.784	-1.08498	down	3.65E-07
KCNMB1	2.165	2.251	3.823	3.079	2.85	2.714	6.685	15.767	12.07	5.764	1.601359	up	1.77E-13
TENM2	2.348	2.093	5.216	7.734	11.746	0.021	0.023	0.187	0.048	0.052	-6.43849	down	#######
HMMR	0.161	0.243	0.444	0.214	0.458	0.636	0.54	3.563	1.157	1.46	2.271093	up	3.36E-22
PTTG2	0.163	0.266	0.486	0.201	0.251	0.63	0.695	5.578	1.377	2.362	2.956095	up	1.56E-27
LOC41614	9.844	10.681	17.565	12.045	8.298	13.023	13.698	59.487	45.133	45.849	1.600359	up	9.89E-14
DUSP1	33.482	31.109	90.424	20.028	34.609	16.531	14.364	14.818	15.066	25.222	-1.28552	down	5.76E-09
STC2	18.675	18.883	15.306	31.688	29.823	5.35	10.373	4.773	4.546	5.554	-1.90218	down	8.53E-19
MXD3	0.861	1.164	1.964	0.786	1.454	0.13	0.523	0.55	0.51	0.594	-1.43102	down	1.29E-09
LOC10175	0.028	0.186	0.497	0.21	0.437	0.439	0.388	1.964	0.844	1.16	1.816249	up	2.60E-12
GRIA1	0.327	0.317	0.163	1.681	1.982	0.033	0.623	0.258	0.427	0.022	-1.70982	down	2.45E-14
GPX3	867.991	945.104	1312.301	2096.424	2210.356	549.777	947.244	419.793	513.745	443.788	-1.37055	down	1.40E-10
MRNIP	1.218	1.165	2.289	1.566	2.92	2.057	2.535	7.059	4.23	3.168	1.056202	up	1.54E-06
LECT2	0.954	1.249	2.462	8.815	0.51	0.185	0.13	0.499	1.018	0.468	-2.60207	down	2.25E-27
SLC22A5	40.76	42.726	34.423	30.492	37.282	12.276	20.927	22.914	16.267	10.658	-1.16088	down	1.91E-08
IRF1	16.405	14.38	24.922	15.38	15.174	27.281	20.153	50.969	41.44	58.06	1.197966	up	1.02E-08
LEAP2	96.682	98.368	85.891	140.103	52.303	308.857	283.177	167.624	191.299	376.046	1.487192	up	1.71E-12
YIPF5	30.659	28.106	20.453	18.425	20.253	60.445	39.32	58.174	45.845	51.47	1.114386	up	1.15E-07
GFRA3	290.416	277.802	351.34	274.504	381.394	69.358	77.391	145.165	114.288	70.337	-1.72509	down	1.56E-16
EGR1	81.128	64.473	88.761	3.013	5.115	8.636	3.556	12.506	13.944	32.995	-1.75908	down	5.93E-17
BHLHA15	0.159	0.415	0.505	0.326	0.163	1.459	1.22	14.09	1.058	2.789	3.712524	up	1.07E-35
NPTX2	0.437	0.313	0.673	0.648	1.722	0.082	0.217	0.018	0.246	0.15	-2.40318	down	3.98E-20
PDGFA	2.159	2.052	4.094	1.986	2.271	0.597	1.054	1.612	1.16	1.724	-1.03051	down	1.27E-05
CYP3A5	1487.008	1384.543	900.646	1710.507	882.877	288.604	870.173	254.674	975.217	505.691	-1.13705	down	2.70E-08
RNF216	2.352	2.423	3.154	2.45	2.902	3.659	5.087	7.187	6.151	4.682	1.010764	up	1.21E-06
LOC10174	1.163	1.458	0.216	0.653	0.756	1.426	4.072	3.619	4.234	1.204	1.776136	up	7.73E-15
PLD6	1.54	1.389	5.818	1.317	1.304	0.887	0.996	0.707	1.324	0.97	-1.218	down	3.69E-07
PEMT	160.853	158.596	118.057	108.361	86.333	345.778	237.903	293.344	295.387	265.337	1.185353	up	1.49E-08
CACNA1H	0.06	0.042	0.158	0.103	0.147	0.873	0.494	0.344	0.342	1.354	2.727973	up	1.10E-32
SOX8	0.807	0.688	2.121	0.341	1.305	0.213	0.251	0.447	0.226	0.4	-1.77218	down	5.75E-12
MEIOB	1.591	1.449	0.294	0.209	0.039	0.431	0.261	0.233	0.235	0.447	-1.15393	down	2.07E-07
PLK1	0.239	0.073	0.279	0.157	0.425	0.431	0.326	5.587	1.272	1.73	2.988781	up	1.89E-33
IL21R	0.252	0.279	0.617	0.485	0.398	0.431	0.728	1.075	1.33	1.868	1.417073	up	1.54E-10
SOCS1	5.662	4.121	7.918	8.084	8.578	8.464	9.189	18.673	17.098	18.607	1.067652	up	5.10E-07

CIITA	0.57	0.492	0.821	0.551	0.448	0.886	0.856	1.207	1.292	1.932	1.097569 up	1.60E-07
CARHSP1	2.219	2.095	4.488	3.529	2.904	13.879	3.897	11.919	5.341	12.145	1.630496 up	8.98E-15
HBZ	0	0.128	0.052	0.107	0	1.64	0.056	1.532	0.134	0	3.527423 up	1.32E-15
HBAD	93.174	97.936	309.947	23.94	123.835	9.736	13.998	12.304	39.873	7.574	-2.95818 down	1.12E-38
HBA1	365.186	383.036	1151.922	127.271	387.325	48.276	53.186	49.282	138.56	30.386	-2.9171 down	4.17E-38
LOC41665	7.203	7.536	6.535	1.798	2.021	0.11	0.169	0.149	0.421	0.099	-4.71895 down	9.51E-81
ZKSCAN7	3.465	2.733	3.456	1.473	1.73	0.836	0.636	1.213	2.06	1.21	-1.10973 down	1.39E-07
NME4	747.074	821.07	1052.988	918.346	1253.88	373.024	348.068	394.162	400.395	404.58	-1.31976 down	3.26E-10
DECR2	121.269	119.623	149.746	120.838	144.37	71.526	50.075	76.132	65.081	53.438	-1.05227 down	4.28E-07
LOC12110	0.412	0.651	0.383	0.479	0.07	0.864	0.498	1.703	0.352	0.842	1.092207 up	9.54E-05
CCNF	1.144	1.081	1.308	0.662	0.751	1.57	1.131	3.722	2.152	1.646	1.04645 up	1.71E-06
ACSM4	1.16	1.074	2.749	1.418	3.226	2.11	4.725	3.788	5.805	8.723	1.384995 up	1.74E-11
LOC12110	1.268	1.333	0.721	1.389	0.977	0.745	0.4	0.434	0.201	0.358	-1.40956 down	5.24E-10
ERI2	1.085	1.065	2.432	2.194	1.598	3	4.036	5.705	2.768	2.591	1.111538 up	1.12E-07
CDC45	0.14	0.165	0.367	0.161	0.287	0.948	0.621	3.761	1.405	1.349	2.846036 up	7.58E-32
CLDN5	8.478	13.541	14.928	11.196	19.093	2.59	5.809	7.106	6.256	3.93	-1.3878 down	4.95E-11
COMT	25.212	23.734	31.14	30.333	38.819	8.97	14.959	14.344	13.372	12.046	-1.22839 down	4.55E-09
RIMBP2	2.897	2.804	1.685	3.33	2.154	6.808	6.158	5.228	6.031	6.191	1.240495 up	4.55E-09
AACS	7.247	6.549	6.818	7.214	4.705	44.4	24.813	14.433	13.041	34.894	2.015809 up	9.72E-22
ATP2A2	15.283	13.796	24.41	14.236	13.583	88.832	21.497	64.198	38.079	52.124	1.702991 up	2.09E-16
P2RX7	4.314	3.019	8.787	4.016	2.9	5.162	4.113	18.812	8.806	21.622	1.344726 up	1.72E-10
HPD	1659.923	1628.15	1574.406	2282.459	1931.67	431.392	1311.21	354.792	656.525	407.425	-1.52161 down	1.05E-12
LRRC43	0.127	0.138	1.869	0.417	0.413	0.287	0.126	0.414	0.087	0.259	-1.33364 down	1.26E-06
RAD9B	1.605	1.591	1.683	1.202	0.539	0.108	0.217	0.81	0.473	0.377	-1.73515 down	6.76E-15
PPTC7	26.451	23.969	28.714	22.315	24.565	8.724	10.225	12.468	9.412	9.905	-1.31247 down	3.10E-10
ACACB	1.373	1.365	0.944	2.486	1.391	0.018	0.112	0.085	0.067	0.061	-4.44199 down	1.20E-70
FAM222A	1.655	1.592	0.582	0.878	1.87	0.354	1.042	0.468	0.106	0.18	-1.61084 down	4.16E-13
CHCHD10	310.225	289.108	416.78	254.481	521.347	156.701	137.845	128.883	105.657	128.293	-1.44672 down	1.50E-11
MMP11	1.993	2.144	0.992	1.228	1.358	0.408	0.966	0.4	0.334	0.329	-1.66054 down	7.93E-13
SLC2A11	1.59	1.464	1.87	1.186	2.764	0.912	0.178	0.591	0.627	1.06	-1.39636 down	2.79E-10
SLC2A11L	3.606	3.592	3.219	2.567	6.134	0.779	0.549	0.99	0.636	0.996	-2.27356 down	1.37E-23
LOC76955	17.466	12.831	48.828	14.684	24.095	4.916	6.61	5.521	4.588	4.901	-2.15138 down	5.25E-22
LOC10175	10.07	6.966	32.213	15.319	9.528	4.126	5.991	2.751	5.848	5.631	-1.60545 down	4.16E-13
TBX6	2.077	2.257	1.313	2.702	4.297	0.36	1.286	1.083	1.017	1.052	-1.39724 down	3.72E-11
LOC41695	1.175	1.185	0.618	1.032	0.658	2.683	1.942	5.048	4.921	2.731	1.890851 up	9.22E-18
PISD	20.194	19.222	12.543	12.178	6.629	66.952	28.23	66.532	67.577	57.542	2.019006 up	1.05E-20
LOC10085	4.583	5.225	3.024	4.197	7.868	2.472	2.684	1.197	2.351	2.088	-1.20563 down	1.74E-07
CIT	0.019	0.009	0.022	0.035	0.034	0.108	0.112	1.036	0.321	0.29	3.916168 up	4.87E-45

SLC35E4	0.209	0.239	0.312	0.204	0.174	3.462	1.137	2.147	1.124	2.305	3.15484	up	6.30E-40
TCN2	106.731	126.53	35.771	38.128	31.915	297.539	270.06	188.889	189.072	340.505	1.923272	up	3.66E-19
HORMAD:	1.423	1.088	1.587	0.976	1.965	0.672	0.552	0.692	0.405	0.505	-1.31508	down	1.17E-09
LOC41701	111.681	109.563	124.638	98.657	150.498	33.571	41.953	47.147	34.017	27.285	-1.69346	down	1.21E-15
LOC1211C	0.534	0.73	2.048	1.755	7.974	0.752	0.829	1.014	1.493	2.024	-1.09271	down	7.49E-06
MHCY14	1.109	0.968	2.202	1.215	0.577	1.146	0.101	0.27	0.44	0.266	-1.44737	down	1.06E-09
LOC1211C	1.131	0.841	0.951	0.317	0.172	0.398	0.176	0.151	0.33	0.042	-1.63261	down	2.45E-11
MHCY11	0.567	1.215	1.511	0.354	3.776	0.549	0.401	0.128	0.722	0.144	-1.93024	down	5.78E-15
MHCY9	1.902	1.998	3.085	1.291	0.376	5.915	2.669	6.565	5.496	3.311	1.468749	up	4.60E-12
LENG9L7	4.913	5.016	7.793	9.996	2.767	2.782	0.051	0	0	0.016	-3.41728	down	1.70E-45
MHCY15	1.035	0.816	1.204	0.981	0.6	1.542	0	0.017	0.109	0.065	-1.41701	down	6.30E-09
MHCY7	1.2	0.974	2.181	1.171	0.861	1.344	0.214	0.572	0.731	0.307	-1.01042	down	1.14E-05
LOC10085	2.662	2.712	4.583	2.073	3.45	0.503	0.119	0.083	0.624	0.052	-3.48187	down	1.98E-46
LOC1211C	0.996	0.879	4.17	0.918	0.279	0.468	0.193	0.079	0.015	0.049	-3.16317	down	7.92E-33
LOC1211C	0.866	0.54	1.248	0.39	0.508	0.32	0.317	0.269	0.233	0.152	-1.4566	down	2.41E-08
LOC1211C	1.782	1.403	1.563	0.448	0.336	0.422	0	0.047	0	0.03	-3.45761	down	2.28E-27
LOC1211C	1.26	0.714	1.528	4.729	15.029	8.032	0.048	0.074	2.489	0.031	-1.12338	down	1.59E-06
LOC1211C	10.943	11.057	14.532	7.957	1.516	6.797	1.599	0.235	1.791	0.706	-2.04711	down	6.81E-20
MHCY8	2.228	1.938	2.656	1.732	1.521	0.98	0.36	0.928	1.099	0.837	-1.25995	down	5.24E-09
MHCY6	4.16	4.452	5.831	2.379	5.454	4.177	0.737	1.352	1.593	1.129	-1.30894	down	4.65E-09
MHCY32	0.15	0.147	0.268	0.446	8.325	0.816	0.32	0.073	0.077	0.031	-2.82085	down	1.19E-27
LOC1211C	2.086	2.181	22.64	26.21	25.371	1.415	2.957	0.871	2.576	0.336	-3.26592	down	9.74E-46
BTN3A3L1	0.297	0.36	0.624	0.731	0.914	0.697	2.66	0.166	2.999	0.26	1.211381	up	2.11E-08
KIFC1	0.701	0.582	4.305	3.332	4.287	10.089	4.282	16.177	5.282	9.345	1.773835	up	3.09E-17
IL4I1	0.662	0.6	1.835	0.561	0.701	1.591	1.677	6.272	4.707	2.374	1.929718	up	3.91E-18
TRIM7.1	0.057	0.037	2.341	0.117	0.059	0.041	0.061	4.307	0.073	1.774	1.259031	up	4.30E-09
TRIM27.1	0.821	0.682	1.079	0.278	0.455	0.164	0.379	0.221	0.254	0.085	-1.58323	down	2.15E-10
BLB1	8.097	12.531	19.195	28.102	17.274	23.041	25.06	53.654	41.916	54.07	1.214655	up	1.41E-08
DMA	2.389	2.955	4.634	5.282	2.944	4.5	4.675	11.572	10.871	14.278	1.333874	up	1.30E-09
DMB2	1.748	1.707	3.574	4.117	2.439	3.032	3.7	7.907	6.826	9.431	1.185108	up	4.82E-08
BF1	102.667	115.593	124.619	110.625	113.802	93.927	113.195	272.666	336.34	334.889	1.020704	up	1.84E-06
TAP1	6.663	6.357	9.565	7.392	6.255	7.958	8.206	19.679	17.411	23.386	1.080728	up	3.73E-07
CYP21A1	0.111	0.145	0.044	1.045	0.596	0.107	0.142	2.492	0.823	0.565	1.087027	up	0.000274
LOC1211C	1.894	1.973	0.277	0.921	0	0.037	0.099	0.404	0.04	0.316	-2.49239	down	2.10E-19
LOC10704	0.733	0.625	0.711	0.838	1.08	0	0.163	0.25	0.262	0.469	-1.79673	down	3.53E-11
MAN1B1	82.815	78.378	81.445	67.277	71.142	34.703	36.051	40.178	29.826	31.182	-1.14808	down	3.27E-08
EXFABP	4.471	7.057	6.987	22.369	8.956	23.977	33.946	17.01	37.555	24.728	1.460981	up	8.83E-12
STPG3	2.738	3.512	3.672	2.191	2.479	1.744	1.2	1.6	1.22	0.943	-1.12086	down	7.26E-07

NSMF	0.468	0.31	1.023	0.218	1.076	4.256	5.141	1.546	5.782	4.783	2.794869	up	3.34E-39
BRINP1	0.307	0.211	2.795	0.579	0.614	0.43	0.314	0.46	0.126	0.038	-1.71612	down	3.21E-11
LOC10705	75.951	77.659	59.169	58.121	65.695	25.735	32.412	43.406	24.459	20.792	-1.19709	down	4.95E-09
SLC25A25	43.558	44.396	31.78	31.397	45.136	12.399	15.104	26.79	10.112	9.021	-1.41839	down	4.33E-12
AK1	0.206	0.404	0.273	0.141	0.67	1.063	0.205	1.076	1.056	0.561	1.222999	up	4.16E-06
FPGS	41.104	41.489	85.376	36.875	85.68	10.45	14.543	12.961	11.086	10.676	-2.28237	down	2.63E-25
DNM1	9.543	8.703	6.504	6.356	9.262	3.459	6.557	2.947	3.85	2.741	-1.04556	down	8.76E-07
PHYHD1	7.328	7.702	5.413	8.719	10.513	1.302	6.359	3.562	2.917	4.196	-1.11334	down	9.79E-08
LOC41719	0.381	0.311	0.657	0.73	0.326	0.246	0.352	4.187	1.237	3.478	1.979653	up	4.51E-15
ASS1	5.658	7.386	12.713	12.465	14.161	12.502	11.3	39.519	33.708	27.675	1.251257	up	4.37E-09
LAMC3	0.196	0.069	0.073	0.098	0.439	1.377	1.107	1.043	0.129	0.401	2.206615	up	8.98E-23
FAM69B	0.345	0.319	0.485	0.751	0.563	2.142	0.936	0.742	0.895	1.51	1.33589	up	3.77E-09
AGPAT2	9.837	11.148	19.814	12.831	6.98	105.724	67.556	80.148	101.116	93.412	2.885626	up	4.54E-40
LOC41711	1.094	1.081	2.958	1.086	1.088	5.538	1.762	5.506	5.4	4.072	1.607606	up	1.01E-14
HSPA5	203.11	183.635	232.322	172.83	247.172	452.942	178.894	883.871	263.315	456.191	1.105117	up	1.86E-07
MVB12B	4.325	3.783	5.199	4.809	4.784	18.241	5.278	15.955	12.843	11.939	1.488281	up	1.21E-12
PIK3R5	0.987	1.022	1.995	1.309	1.236	1.482	1.872	4.031	3.261	3.26	1.085779	up	2.42E-07
HS3ST3B1	76.526	69.615	73.481	95.337	88.844	32.22	61.914	34.826	31.994	40.677	-1.00192	down	1.77E-06
HS3ST3A1	5.418	5.768	2.626	4.097	4.397	0.36	1.005	1.163	0.238	0.855	-2.6213	down	1.29E-33
FN3K	3.913	3.747	7.322	10.982	14.227	3.071	3.678	4.114	2.981	3.408	-1.21987	down	8.03E-09
MXRA7	1.115	1.55	1.289	1.022	0.65	4.931	1.758	11.005	1.935	2.832	1.99628	up	2.37E-19
ST6GALNA	1.081	0.874	0.426	0.423	0.529	0.347	0.697	3.184	1.615	1.118	1.061347	up	5.02E-06
TEN1	4.687	5.166	5.237	10.568	7.423	1.259	2.557	2.161	1.212	1.321	-1.95814	down	1.05E-19
ACOX1	304.321	272.828	426.332	464.053	441.56	107.899	124.232	112.866	109.443	98.296	-1.78822	down	3.90E-17
MRPL38	52.316	48.259	56.053	35.782	44.87	25.423	23.121	24.516	22.1	19.065	-1.05468	down	5.53E-07
SMIM5	0.681	0.722	0.237	1.602	0.909	4.875	3.892	2.076	1.855	1.3	1.752468	up	3.34E-16
MYO15L	0.333	0.301	0.036	0.032	0.085	0.403	0.219	0.71	0.604	1.02	1.902512	up	2.55E-15
FASN	110.775	119.381	100.058	62.645	146.049	1022.308	312.285	561.44	607.844	419.522	2.439525	up	3.71E-30
TRIM25	15.807	17.413	23.624	22.527	19.432	20.69	27.283	70.606	45.863	51.357	1.127022	up	1.32E-07
KPNA2	1.883	1.577	2.2	1.866	1.482	2.982	3.292	19.884	5.633	7.153	2.111507	up	3.20E-21
CACNG1	0.295	0.193	0.268	2.11	0.519	2.69	1.51	0.605	1.943	0.413	1.079883	up	1.30E-05
SDK2	0.273	0.481	0.456	0.484	0.421	0.809	0.647	1.14	1.089	1.522	1.297773	up	2.95E-09
RNF213	3.711	3.683	12.269	5.923	4.87	5.488	6.062	27.944	16.456	25.214	1.413966	up	1.19E-11
CBX2	0.516	0.535	0.24	0.106	0.309	3.979	1.691	4.356	3.046	3.572	3.282524	up	4.95E-41
RBFOX3	0.214	0.159	0.327	0.123	0.086	3.115	0.972	1.413	1.377	1.632	3.219571	up	1.08E-42
ENPP7	0.952	1.027	0.061	0.529	0.026	2.492	0.638	1.345	1.056	3.052	1.723811	up	2.52E-12
LOC12110	0.102	0.089	0.135	0.056	0	1.117	0.667	0.355	0.696	0.583	3.144856	up	2.33E-22
TIMP2	102.314	120.649	120.318	83.634	148.091	606.094	337.179	370.695	287.896	521.016	1.884365	up	1.79E-19

SOCS3	3.048	2.755	5.606	4.452	1.599	21.174	11.203	30.478	28.842	30.489	2.806599	up	5.28E-37
TK1	1.116	0.548	2.311	0.917	3.497	10.323	3.719	25.151	8.016	6.892	2.688358	up	1.19E-31
LOC10085	2.368	2.302	3.841	2.2	3.591	3.814	3.478	11.618	8.376	11.906	1.454022	up	1.22E-11
ARL16	1.728	1.557	2.905	1.483	3.254	2.882	2.46	6.971	5.376	7.316	1.193948	up	1.80E-08
KRABZFP	0.342	0.28	0.204	0.421	0.176	1.241	0.219	1.451	0.614	5.591	2.675196	up	7.00E-18
GRIN2C	1.068	1.271	0.191	0.296	0.839	0.133	0.454	0.41	0.147	0.575	-1.09002	down	1.64E-07
OTOP3	0.434	0.426	0.49	0.694	0.793	0.934	2.06	0.661	0.804	1.607	1.095028	up	2.52E-07
SLC16A5	100.899	98.569	67.509	91.142	104.637	31.655	37.75	50.327	27.208	20.289	-1.4684	down	1.10E-12
CCL4	0.67	0.776	1.231	0.127	1.059	0.599	0.528	31.103	10.416	18.167	3.974839	up	1.33E-56
GATSL2	2.567	2.397	5.105	1.018	4.584	0.887	1.52	1.162	0.595	0.401	-1.77829	down	7.22E-15
MIS12	0.314	0.78	0.4	0.774	1.29	2.736	1.448	14.104	2.705	3.44	2.777962	up	5.96E-27
TMEM120	19.329	20.747	33.045	32.708	21.598	10.615	9.548	13.712	14.127	11.464	-1.09947	down	1.27E-07
MMP28	0.074	0.039	0.71	0.659	0.183	0.503	0.216	0.621	1.889	0.607	1.201634	up	2.07E-07
LOC1211C	1.481	1.211	2.801	0.355	3.169	0.657	1.002	7.415	5.76	12.515	1.600233	up	1.68E-12
SERPINF1	74.377	64.558	89.8	202.538	74.576	7.07	13.871	11.037	15.619	10.732	-3.11631	down	1.85E-45
SRR	5.107	4.768	4.455	3.419	3.831	1.584	1.863	1.012	1.972	1.431	-1.45614	down	5.26E-11
SEBOX	6.22	6.517	4.5	18.195	17.428	22.559	10.666	43.077	39.929	15.462	1.316849	up	1.71E-09
SLC13A2	0.047	0.133	0.025	0.064	0.144	0.121	0.786	0.387	2.191	0.229	3.15334	up	5.46E-30
SPAG5	0.029	0.064	0.156	0.161	0.151	0.303	0.167	1.94	0.682	0.815	2.789032	up	3.78E-28
CORO6	0.518	0.397	0.179	0.407	0.901	3.726	2.691	5.614	1.985	0.607	2.603424	up	3.83E-28
GSG2	0.257	0.236	0.256	0.359	0.092	0.473	0.165	1.311	0.501	0.6	1.342139	up	1.42E-07
ALDH3A2	214.585	215.271	241.807	224.643	354.676	110.95	159.323	113.229	122.61	95.125	-1.05705	down	6.10E-07
DOC2B	0.011	0	0.009	0	0	5.135	0.048	0.148	0.035	0.945	7.980939	up	6.65E-96
PRR11	0.194	0.07	0.137	0.159	0.177	0.208	0.221	1.855	0.397	0.545	2.12249	up	1.15E-13
VMP1	18.126	15.486	13.41	13.021	13.419	38.078	24.9	38.85	33.018	33.234	1.194023	up	1.35E-08
MED13	24.129	20.91	40.145	20.433	24.737	8.981	12.41	11.425	9.476	9.916	-1.32001	down	7.27E-10
ACACA	39.426	40.517	32.023	29.127	40.439	204.683	100.664	168.898	123.51	122.501	1.988256	up	7.11E-21
C17orf78	0.122	0.175	0.135	0.12	0.374	1.153	0.332	0.444	0.249	0.338	1.437143	up	6.54E-08
TADA2A	2.004	1.77	1.433	1.767	3.035	7.252	3.173	4.337	4.141	3.684	1.173794	up	2.01E-08
DUSP14	0.586	0.629	0.73	0.309	0.787	3.301	1.424	3.394	1.818	1.646	1.927644	up	3.06E-18
EVI2A	0.625	0.324	0.788	0.835	0.294	0.731	1.157	1.599	2.137	3.343	1.643876	up	2.81E-12
CLUH	25.099	24.566	25.932	21.027	30.204	11.361	14.744	13.609	10.571	11.592	-1.03534	down	7.82E-07
LOC10085	4.819	5.096	6.406	4.686	5.925	9.954	6.563	16.748	12.632	14.317	1.16063	up	3.76E-08
FAM64A	0.423	0.332	0.789	0.635	0.596	0.76	0.537	2.209	1.611	1.403	1.230893	up	4.28E-08
FBXO39	0.27	0.238	0.364	0.239	0.256	0.493	0.488	1.602	1.226	0.91	1.783728	up	1.88E-14
TEKT1	0.191	0.104	0.304	0.209	0.261	0.277	0.245	1.288	0.587	0.468	1.418057	up	1.21E-06
LOC10705	6.771	7.469	7.903	5.264	7.808	30.044	9.572	23.149	23.091	18.419	1.565996	up	7.27E-14
ASIP	0.562	0.735	1.598	0.198	0.165	0	0.206	0.315	0.412	0.066	-1.70044	down	3.86E-06

TOX2	0.328	0.221	0.563	0.379	0.442	0.556	0.473	0.924	0.736	1.786	1.20893	up	4.63E-08
ACSS2	10.983	11.935	6.329	9.149	7.517	15.335	13.365	25.926	20.69	18.633	1.032895	up	2.10E-06
FAM83D	0.311	0.18	0.391	0.246	0.283	0.44	0.48	1.563	0.835	0.763	1.528868	up	7.90E-11
RPN2	10.605	11.005	15.321	11.706	19.106	34.314	18.285	32.193	22.628	30.274	1.023268	up	4.93E-07
RBPJL	0.057	0.025	0.045	0.046	0.039	0.091	1.335	0.025	0.406	0.231	3.269805	up	1.77E-25
PI3	9.954	13.416	15.077	52.646	13.145	12.03	199.648	23.949	228.012	48.41	2.296345	up	3.08E-26
TTPAL	2.052	2.341	4.009	2.487	3.081	4.518	5.454	6.243	6.919	6.878	1.102932	up	6.93E-08
GDAP1L1	0.38	0.327	1.069	0.882	0.257	0.224	0.063	0.218	0.199	0.083	-1.8824	down	4.94E-15
RPESL	1.563	1.728	2.696	1.498	1.586	7.819	2.273	11.834	9.695	5.781	2.04318	up	4.76E-21
EPB42	0.861	1.048	2.935	0.467	0.938	0.067	0.265	0.315	0.76	0.183	-1.97122	down	7.23E-16
ZNFX1	6.703	7.556	14.281	12.287	5.833	8.08	7.363	59.948	37.073	46.89	1.771868	up	3.39E-16
GATA5	9.765	8.934	4.151	9.352	21.945	1.102	4.814	4.076	1.751	2.477	-1.92859	down	1.88E-19
COL20A1	0	0	0.005	0	0	0.677	0	0	1.122	0.005	7.499049	up	6.18E-72
HELZ2	6.097	6.136	11.043	5.603	7.422	7.129	6.152	36.346	20.917	30.801	1.481066	up	3.41E-12
SLC52A3	1.959	1.815	1.435	1.011	1.116	3.088	1.759	3.082	3.125	3.742	1.011646	up	3.01E-06
TPX2	0.395	0.488	0.943	0.663	0.899	1.478	1.172	5.702	2.2	2.013	1.889351	up	9.67E-18
IRF9	6.254	7.146	5.257	3.23	5.353	13.017	8.013	15.146	12.698	18.043	1.296489	up	1.11E-09
BPIFB3	0.011	0.011	0.103	0.074	0.017	0.044	0.014	1.323	0.617	0.419	3.454081	up	7.26E-39
LOC77197	0.556	0.685	0.352	0.43	0.017	1.854	0.825	2.244	1.477	1.311	1.915752	up	8.20E-16
LOC10085	0.582	0.533	0.463	0.064	0.199	1.314	0.331	1.469	2.028	0.602	1.638908	up	6.42E-10
WFDC2	5.532	6.109	0.358	1.952	0	17.215	13.318	20.348	15.096	13.446	2.508762	up	7.12E-29
WFDC8	75.251	79.538	7.492	29.801	0.154	239.842	170.729	318.786	263.366	207.997	2.642918	up	7.14E-32
SPINT4	2.528	2.408	0.318	0.474	0	9.706	3.978	6.546	5.869	6.311	2.499299	up	5.43E-26
LOC77199	0.826	0.42	0.122	0.151	0.157	1.392	1.071	0.719	1.82	1.401	1.930554	up	3.58E-14
UBE2U	0.241	0.158	0.346	0.516	0.843	1.543	0.99	15.199	4.557	4.502	3.667387	up	5.76E-44
MMP9	0.016	0.307	0.258	0.786	0.2	0.518	0.498	1.545	1.33	3.115	2.157019	up	9.98E-19
PCK1	3.422	4.317	65.761	5.388	29.476	0.07	0.995	2.535	0.413	1.035	-4.42267	down	1.25E-73
AURKB	0.977	1.215	1.312	1.369	1.239	1.576	1.309	5.928	2.397	1.926	1.103179	up	1.48E-06
UTS2	1.114	0.865	5.154	1.43	4.218	1.617	0.773	0.182	1.356	1.081	-1.35053	down	7.16E-07
ESPN	0.409	0.47	0.315	0.23	0.162	4.903	0.472	0.976	3.257	7.735	3.446764	up	1.26E-49
AJAP1	0	0	0.02	0.011	0.007	0	0	0	0.013	1.966	5.527932	up	2.03E-61
KBP	3.424	3.238	8.425	4.669	3.62	1.533	2.441	2.133	2.624	2.248	-1.08993	down	5.47E-07
MMP23A	1.292	1.102	1.579	1.297	5.424	0.553	0.717	1.299	0.904	0.925	-1.28092	down	2.01E-09
MIB2	37.939	37.536	44.728	17.633	58.931	10.595	9.406	20.524	22.333	10.328	-1.42679	down	8.75E-12
LOC41940	8.82	8.759	5.918	5.218	47.946	0.868	0.924	1.313	1.85	0.912	-3.70666	down	2.50E-58
FNDC10	3.834	3.489	2.521	2.027	6.311	1.893	1.168	1.925	1.665	1.348	-1.18411	down	1.69E-08
CCNL2	66.827	55.969	92.787	54.873	42.426	26.918	29.887	32.586	31.812	34.254	-1.00908	down	1.59E-06
LOC77106	34.474	35.519	33.965	35.915	49.009	4.045	8.09	6.712	4.654	4.02	-2.77866	down	1.06E-36

CENPS	0.55	0.3	0.566	0.603	0.518	0.555	0.372	3.472	1.34	0.937	1.3941 up	1.99E-08
RNF186	11.188	11.918	0.265	5.554	0.043	27.689	35.136	17.275	33.193	18.043	2.180536 up	5.94E-23
CAMK2N1	11.466	14.312	4.295	4.522	3.703	51.975	4.816	6.727	22.135	17.623	1.431046 up	1.33E-11
CELA2A	0.203	0.266	0.485	0.467	0.334	1.062	0.521	1.221	1.792	2.16	1.941661 up	1.37E-13
DRAXIN	0.724	0.636	0.465	0.337	0.311	1.182	0.886	1.454	3.347	3.614	2.081491 up	3.85E-20
EPHB2	3.285	3.747	3.113	2.948	5.027	14.264	6.291	11.573	6.458	7.997	1.361977 up	6.51E-11
P3H1	8.357	8.893	9.095	6.283	8.827	15.743	11.518	35.628	17.912	17.502	1.245589 up	5.42E-09
ECE1	39.205	38.167	53.402	47.779	35.696	12.789	18.422	15.674	15.314	16.204	-1.45025 down	5.62E-12
LOC11253	13.755	12.357	22.05	16.679	10.052	4.619	5.908	4.762	4.814	6.005	-1.52016 down	1.06E-12
DPYSL2	1.602	1.773	1.878	1.072	1.27	4.568	3.054	3.452	4.645	2.946	1.296651 up	4.14E-10
CDCA2	0.336	0.279	0.349	0.316	0.395	0.326	0.344	1.427	0.77	0.668	1.075288 up	2.55E-06
EGR3	0.164	0.197	0.283	0.202	0	0.318	0.21	0.786	0.505	1.7	2.049983 up	2.72E-13
LOC10705	1.314	1.271	1.046	0.63	0.759	0.477	0.397	0.786	0.309	0.425	-1.06669 down	6.46E-06
DMTN	0.926	0.799	1.2	0.544	0.68	0.217	0.339	0.239	0.313	0.092	-1.78547 down	5.24E-14
LOC10174	25.547	25.293	7.821	17.495	25.19	5.827	17.652	7.786	7.047	10.585	-1.05139 down	5.01E-07
ADAM32L	1.354	1.2	1.01	1.38	1.989	0.232	0.644	0.157	0.105	0.112	-2.46683 down	3.79E-23
IDO2	10.394	12.035	5.352	19.063	27.269	1.37	6.145	0.308	0.449	0.262	-3.11768 down	6.70E-42
ANK1	1.223	1.303	2.635	0.715	1.221	0.294	0.404	0.265	0.473	0.141	-2.16648 down	4.69E-22
NCAPH	0.605	0.485	0.927	0.671	0.544	1.435	0.853	4.187	1.691	1.434	1.569128 up	4.46E-12
ADD2	1.232	1.137	1.521	0.989	0.687	0.49	0.334	0.673	0.549	0.328	-1.22758 down	1.93E-08
SLC20A1	16.074	16.555	17.413	10.516	25.991	6.85	5.711	5.39	5.988	5.42	-1.55955 down	3.32E-13
CASP14	1.235	1.087	1.424	1.278	0.897	0.206	0.364	0.25	0.175	0.279	-2.21204 down	1.48E-18
RETSAT	181.098	181.873	305.999	306.703	245.919	68.665	63.549	44.938	35.779	62.133	-2.1509 down	2.72E-23
FABP3	13.543	18.173	2.598	3.493	0.203	210.982	35.213	48.032	84.067	89.894	3.622463 up	1.42E-54
SERINC2	1.761	1.695	1.951	0.682	1.747	5.94	1.677	4.389	3.281	3.592	1.268055 up	2.32E-09
GBP1	8.111	10.391	15.138	12.653	11.533	15.183	12.148	50.99	36.803	40.156	1.425004 up	2.78E-11
THEMIS2	3.527	3.471	6.422	5.837	6.674	7.684	7.029	16.847	14.102	11.336	1.136082 up	6.85E-08
LOC10174	0.118	0.173	0.798	1.21	1.15	0.171	0.277	0.192	0.091	0.289	-1.75264 down	6.92E-10
MTFR1L	20.669	19.3	29.918	13.246	19.449	9.207	8.747	11.57	10.643	9.747	-1.03919 down	1.07E-06
TRNAU1A	12.629	11.351	6.146	7.127	5.097	3.189	4.978	3.781	4.63	3.949	-1.04466 down	2.60E-07
CTGFL	0.289	0.532	1.05	0.453	0.696	0.254	0.347	0.307	0.139	0.443	-1.01679 down	2.10E-05
STMN1	5.07	4.523	8.573	3.977	4.092	22.732	11.165	47.605	22.122	22.778	2.268237 up	1.21E-25
PAQR7	24.346	28.129	11.665	9.039	4.872	91.983	55.716	39.179	43.962	61.288	1.904044 up	4.14E-19
DNALI1	1.001	1.403	2.504	1.968	2.937	0.796	0.946	1.027	0.88	1.052	-1.06093 down	8.51E-06
CSF3R	1.081	1.746	1.602	2.991	2.741	2.425	2.373	4.379	6.738	10.072	1.354316 up	5.30E-10
CLSPN	0.579	0.379	0.551	0.47	0.327	0.609	0.525	2.603	0.884	0.982	1.278969 up	1.93E-08
IFI27L2	1.02	1.144	1.74	27.689	12.533	18.024	30.741	4.377	341.82	306.093	3.989674 up	2.44E-64
TMEM35B	35.636	43.246	43.684	26.653	25.922	15.295	19.446	18.648	15.222	17.697	-1.02091 down	1.23E-06

NFYC	25.285	23.993	35.842	24.007	33.118	15.021	14.43	15.084	11.13	15.046	-1.00832	down	2.19E-06
MYCL	25.236	25.063	17.182	6.684	21.801	4.01	4.315	4.245	3.638	2.597	-2.3511	down	2.77E-27
GALE	203.78	179.069	256.203	94.255	87.192	46.704	41.939	74.94	28.56	19.335	-1.95597	down	4.76E-20
HMGCL	1321.124	1321.949	1041.953	914.18	1355.956	244.009	396.528	363.658	216.612	127.708	-2.14277	down	1.25E-23
GRHL3	0.226	0.247	0.18	0.171	0.184	0.557	0.266	0.308	0.813	1.096	1.587808	up	1.54E-11
IL22RA1	1.048	0.851	0.331	0.475	0.408	3.08	4.894	4.343	6.97	7.064	3.079439	up	1.49E-39
LOC10705	2.002	1.958	3.387	2.308	2.827	1.292	1.013	1.622	0.932	1.205	-1.0409	down	1.31E-06
SPA17	5.129	5.559	3.796	3.066	5.694	1.706	3.098	3.045	1.754	1.525	-1.06233	down	1.67E-06
CHEK1	1.146	0.734	1.54	0.373	0.613	1.85	1.244	3.525	1.274	1.699	1.121478	up	4.40E-07
C20orf173	0.903	1.008	0.777	0.633	1.249	0.361	0.309	0.566	0.395	0.532	-1.07741	down	1.09E-06
TMEM45L	0.867	1.134	1.972	3.459	2.645	0.384	1.1	0.162	0.305	0.121	-2.27921	down	3.04E-20
NCAPD3	0.182	0.129	0.23	0.318	0.234	0.772	0.383	3.105	0.935	0.758	2.43995	up	5.28E-25
SCN3B	0.701	0.619	1.075	0.612	0.851	2.52	0.842	2.284	2.94	2.49	1.520294	up	4.17E-13
CRTAM	1.386	1.671	2.218	1.614	1.727	2.014	1.656	7.125	3.911	4.64	1.166481	up	9.39E-08
ZBTB32	25.252	27.628	28.602	8.546	14.028	3.333	3.274	9.15	18.079	5.46	-1.40479	down	8.49E-12
ZPR1	128.277	139.38	105.824	94.012	102.903	49.826	52.167	31.942	37.928	39.158	-1.43456	down	1.00E-11
APOA5	226.944	249.026	159.85	201.021	232.501	66.403	87.128	41.457	60.955	54.093	-1.7862	down	3.77E-17
FXYD2	7.766	4.487	6.425	4.393	4.04	0.684	0.775	1.976	0.621	1.072	-2.40127	down	1.69E-21
SCN4B	1.577	2.38	1.504	1.283	2.197	0.408	0.72	0.714	0.689	0.575	-1.52386	down	6.67E-12
JAML	0.919	0.989	1.647	1.024	0.944	0.878	1.901	3.064	2.171	3.709	1.085127	up	4.14E-07
MPZL2	0.597	0.53	0.725	0.468	0.643	0.275	0.45	2.174	1.517	1.803	1.068351	up	4.13E-06
LOC10174	1.523	1.158	0.721	0.072	0.888	0.159	0.05	0.123	0.706	0.231	-1.77728	down	5.00E-16
HYOU1	31.471	30.812	41.361	31.712	27.66	87.819	42.949	89.033	43.353	67.118	1.018619	up	7.51E-07
PLET1	3.093	2.981	3.518	1.77	3.432	1.096	1.297	1.479	2.351	0.926	-1.04868	down	2.32E-06
IL18	2.926	2.277	5.517	3.361	4.144	3.693	3.541	11.105	8.199	10.461	1.021367	up	1.10E-06
DIXDC1	0.381	0.339	0.549	0.403	0.317	2.26	0.405	1.924	0.741	0.983	1.663803	up	1.38E-14
LOC10174	0.095	0.04	0.054	0.05	0.119	0.171	0.168	1.853	0.46	0.327	3.039206	up	4.64E-32
LOC10174	0.02	0.013	0.032	0.083	1.055	0	0	0.053	0.124	0.132	-1.94378	down	2.68E-08
LOC10175	0.587	0.616	1.155	0.397	0.488	0.134	0.45	0.293	0.31	0.219	-1.20283	down	3.57E-07
CD244	1.468	1.276	3.088	2.971	1.845	4.158	3.05	5.522	3.775	5.249	1.030352	up	1.17E-06
CD48	8.957	8.742	18.85	16.092	16.147	16.997	16.473	36.521	35.385	34.265	1.021441	up	9.24E-07
SLAMF1	0.106	0.374	0.607	0.313	0.413	1.128	0.688	0.996	1.239	1.265	1.549345	up	3.83E-11
CKS1B	1.373	1.796	3.682	5.166	4.156	8.466	7.777	30.482	10.377	11.876	2.092208	up	4.06E-20
ECM1	0.854	0.838	2.168	1.294	0.987	1.344	0.593	3.695	2.765	4.081	1.022245	up	4.89E-06
CIART	0.214	0.482	2.613	0.762	2.785	0.322	0.528	0.28	0.927	0.622	-1.35403	down	9.30E-08
CA14	0.214	0.361	1.585	0.528	2.013	0.178	0.602	0.32	0.597	0.325	-1.21516	down	9.45E-06
PCP4L1	2.075	1.357	5.083	3.804	18.36	1.236	3.408	0.625	5.239	0.783	-1.44167	down	6.15E-08
APOA2	1836.682	2018.685	3427.281	2772.508	4123.305	775.539	1249.588	578.471	1077.364	786.495	-1.66617	down	1.24E-14

TMOD4	0.723	1.269	1.885	0.999	1.756	0.223	0.421	0.816	0.405	0.43	-1.5289	down	6.85E-10
LOC1211(	1.273	1.24	1.603	1.655	1.703	3.212	2.858	3.984	2.582	2.761	1.0422	up	1.92E-06
LOC1211(	1.377	2.274	3.499	15.089	1.684	12.446	13.444	14.903	13.387	16.303	1.558678	up	4.75E-12
THEM4	1.379	1.504	1.63	1.751	3.047	0.121	0.214	0.819	0.086	0.41	-2.49288	down	8.34E-22
LOC1211(	1.707	2.146	2.676	0.723	3.534	0.413	0.547	0.975	0.492	0.96	-1.66962	down	8.45E-13
PFDN2	11.692	13.549	20.304	0.767	14.309	0.361	0.212	0.812	0.447	0.559	-4.66124	down	1.05E-67
TULP1	0.396	0.339	3.696	0.217	0.174	0	0.048	0.111	0.406	0.054	-2.95151	down	9.75E-31
FKBP5	111.755	99.425	223.982	47.184	74.273	20.244	25.394	29.745	85.614	26.154	-1.57246	down	2.63E-13
ELF3	1.802	1.886	5.136	1.871	1.286	4.592	5.509	10.579	14.691	16.195	2.10521	up	5.04E-23
CSRP1	1.668	1.898	3.368	2.702	4.32	11.4	10.923	14.018	18.598	24.531	2.509099	up	7.36E-31
TMEM9	0.347	0.293	0.357	0.268	0.272	1.125	0.261	0.719	0.607	0.517	1.068517	up	1.34E-05
LOC3951(	0.944	0.881	0.892	0.982	0.827	2.888	1.494	2.515	1.717	1.811	1.202838	up	2.92E-08
UBE2T	0.274	0.426	0.464	0.084	0.633	0.962	0.702	4.385	1.546	0.925	2.17637	up	2.33E-17
SHISA4	1.226	0.705	0.384	0.366	0.267	0.396	0.254	0.049	0.114	0.304	-1.39611	down	2.17E-06
LOC10705	17.207	16.935	13.494	18.533	16.182	4.148	4.947	5.302	3.725	8.604	-1.62336	down	6.63E-15
KCNA3	0.153	0.305	0.746	0.993	0.514	0.388	0.901	1.455	1.771	1.397	1.123382	up	2.29E-07
NUAK2	0.135	0.399	0.658	0.145	0.237	0.787	0.995	1.822	0.736	0.986	1.755395	up	1.32E-14
LOC1211(	2.336	2.667	13.532	2.99	5.007	1.407	1.56	2.197	3.712	1.338	-1.37675	down	8.01E-10
SLC45A3	1.655	1.516	4.072	1.441	3.063	0.328	0.464	0.576	0.651	0.802	-2.05607	down	7.71E-21
SLC26A9	0.066	0.086	0.01	0.003	0	5.769	0.746	1.536	3.938	2.697	6.433254	up	#######
FAM72A	0.454	0.348	0.628	0.421	1.013	1.069	0.758	6.594	1.72	2.984	2.194247	up	6.09E-21
CAMK1G	0.091	0.094	0.055	0.136	0.055	0.895	0.365	0.684	0.449	1.087	2.998759	up	5.45E-35
LAMB3	0.296	0.278	0.164	0.519	0.172	3.236	1.386	2.565	1.506	3.002	3.028392	up	4.25E-39
G0S2	17.283	17.63	33.545	71.809	20.964	15.36	92.174	76.113	141.252	241.758	1.813315	up	6.31E-17
GUCA1A	0.963	0.641	1.561	0.964	0.575	0.339	0.269	0.206	0.18	0.186	-1.99053	down	2.71E-15
MOV10	20.693	22.727	15.294	15.974	9.551	30.56	28.779	53.44	47.349	53.133	1.340008	up	5.17E-10
SLC16A1	80.737	81.864	85.098	65.917	80.697	20.656	25.583	25.283	19.839	18.994	-1.83714	down	4.70E-18
SYCP1	5.077	4.565	9.032	3.579	2.613	1.99	1.699	3.961	2.348	1.908	-1.06217	down	5.26E-07
TSHB	2.692	2.656	5.24	1.229	0.966	0.828	0.365	1.395	0.745	0.489	-1.7405	down	4.42E-13
PACSIN1	3.057	2.979	2.103	5.131	1.149	12.017	7.289	11.927	9.634	12.637	1.89131	up	5.76E-18
ITPR3	1.748	2.077	1.917	1.958	2.4	3.587	3.756	4.719	4.271	6.386	1.169147	up	2.61E-08
TSPO2	1.038	0.781	1.984	0.782	0.498	0.034	0.177	0.136	0.923	0.085	-1.90349	down	6.04E-15
CHIA-M3:	0.124	0.809	0.066	1.151	1.037	0.918	1.408	1.022	6.911	1.415	1.871384	up	1.37E-15
LOC7687{	0.737	0.599	0.428	1.424	0.491	0.733	2.382	0.755	4.393	3.633	1.691738	up	1.62E-13
FMOD	0.578	0.697	0.133	0.575	0.156	0.354	0.858	1.947	1.186	0.834	1.273761	up	3.92E-07
GTSF1	4.407	2.561	2.058	0.688	1.688	4.282	9.52	2.146	5.226	3.091	1.089255	up	7.13E-08
DBF4B	2.769	2.891	0.507	1.102	0.954	0.666	0.999	1.145	0.335	0.506	-1.17028	down	3.14E-08
KIF18B	0.025	0.074	0.121	0.114	0.155	0.611	0.302	1.581	0.466	0.66	2.875398	up	9.90E-28

C1QL1	0.528	0.653	1.124	0.237	0.858	0.232	0.32	0.314	0.305	0.432	-1.08239	down	4.76E-06
GH	0.916	0.632	0.729	1.296	0.784	0.099	0.435	3.124	3.549	2.83	1.202985	up	3.65E-06
MRC2	0.907	1.665	5.262	2.221	1.584	3.472	0.919	8.315	5.78	9.171	1.248319	up	1.43E-09
SLC4A1	2.92	2.766	7.813	0.831	2.847	0.287	0.597	0.376	1.19	0.249	-2.66773	down	5.17E-31
C27H17OI	11.499	8.589	25.485	14.803	13.014	5.174	7.346	5.25	5.689	6.283	-1.30294	down	4.34E-09
RAB18L	13.855	12.891	28.425	17.439	22.415	6.191	10.424	8.554	9.15	5.943	-1.23879	down	1.08E-08
PNPO	30.385	31.339	44.875	62.093	37.864	14.459	21.787	16.543	16.241	23.233	-1.16267	down	2.37E-08
SRCIN1	0.09	0.07	0.331	0.849	0.114	1.071	0.428	0.879	0.492	0.389	1.161661	up	1.51E-07
CISD3	17.526	17.821	15.362	17.194	13.187	6.578	7.544	9.671	7.038	9.666	-1.00162	down	1.36E-06
PPP1R1B	2.268	2.589	3.554	0.991	4.04	1.449	0.886	1.513	1.238	1.46	-1.0375	down	4.30E-06
CSF3	0.957	1.356	2.006	1.048	0.786	2.1	1.907	3.999	6.642	3.234	1.538378	up	6.82E-12
CDC6	0.076	0.136	0.196	0.047	0.097	0.312	0.145	1.186	0.408	0.294	2.076912	up	5.13E-13
RARA	10.973	12.41	15.375	11.101	11.083	35.648	15.065	19.315	22.246	30.831	1.014321	up	6.48E-07
TOP2A	0.342	0.255	0.321	0.388	0.281	1.239	1.227	13.115	3.943	3.311	3.84265	up	1.22E-57
CCR7	0.262	0.268	0.371	0.206	0.549	0.947	0.602	0.787	1.073	1.609	1.596497	up	8.83E-13
P3H4	4.043	4.802	2.653	3.765	2.923	0.907	1.853	1.156	1.46	2.015	-1.29841	down	7.33E-10
BRCA1	0.071	0.057	0.165	0.166	0.28	0.634	0.322	3.393	0.957	0.839	3.047212	up	7.21E-38
ANGPTL4	12.453	12.116	22.343	19.188	6.518	8.129	15.035	30.132	23.392	80.053	1.10993	up	1.93E-07
LOC10085	0.669	0.945	2.285	1.243	0.617	3.351	2.964	2.165	3.699	2.608	1.359675	up	2.46E-11
CELF5	1.402	1.574	1.764	1.949	0.551	3.62	2.528	2.215	3.924	2.321	1.012195	up	2.23E-06
MPND	7.891	7.569	12.217	7.376	6.539	3.562	4.834	2.423	2.999	2.622	-1.33883	down	9.99E-10
STAP2	3.545	3.857	4.369	2.185	2.45	0.726	2.475	0.757	0.944	0.948	-1.48692	down	1.90E-10
NMRK2	0.481	0.525	1.118	0.461	0.535	1.437	1.336	2.724	2.262	1.476	1.564037	up	8.32E-11
ACSBG2	14.586	15.643	11.837	8.17	13.21	159.778	52.108	148.794	112.82	179.065	3.362418	up	1.95E-50
MEX3D	0.596	0.738	0.777	0.636	0.69	2.407	0.585	2.219	1.652	1.949	1.357041	up	7.51E-10
LRG1	0.422	0.461	0.359	0.44	0.984	0.3	1.492	1.251	1.387	1.866	1.238201	up	1.96E-07
CREB3L3	159.827	156.871	111.394	114.31	126.707	40.845	69.249	62.133	48.896	39.86	-1.35827	down	5.35E-11
MIDN	1.534	1.929	5.572	2.288	1.636	5.378	3.841	6.457	6.247	6.112	1.112975	up	3.38E-08
GAMT	161.068	171.338	127.697	148.859	189.083	51.007	60.622	50.592	58.227	58.584	-1.51602	down	3.89E-13
PCSK4	2.348	2.734	1.991	1.566	1.895	0.34	2.696	0.637	0.579	0.836	-1.04915	down	3.49E-06
ADAMTSL	0.983	1.337	0.423	0.162	2.564	0	0.101	0.051	0.121	0.032	-4.14226	down	6.41E-43
LOC10085	1.85	1.341	1.179	1.31	0.877	0.276	0.146	0.186	0.292	0.559	-2.16422	down	1.15E-17
USHBP1	1.913	1.846	2.234	1.218	1.441	0.432	0.626	0.687	0.719	0.43	-1.57831	down	2.61E-12
LOC42010	3.702	4.864	6.514	4.686	2.361	6.65	3.571	27.298	14.701	29.421	1.883248	up	1.06E-17
LOC10705	57.522	65.076	78.023	71.484	31.665	67.635	42.415	368.421	242.865	352.847	1.822171	up	8.94E-17
LOC12110	0.088	0.022	0.123	0	0.193	1.902	0.019	0	0.294	0.081	2.416501	up	1.05E-22
LOC12110	0.686	0.658	0.66	1.216	0.474	2.109	2.062	2.844	3.011	2.071	1.710038	up	1.65E-14
ACP5	15.183	16.497	42.327	27.342	39.833	5.887	6.267	11.069	6.974	13.249	-1.70015	down	2.03E-15

LOC10704	4.908	3.093	3.336	1.377	2.029	1.391	1.125	1.954	1.289	0.979	-1.12906	down	1.28E-06
LIG1	0.732	0.415	0.865	0.612	1.003	0.733	0.563	3.538	1.74	1.237	1.105666	up	1.03E-06
LOC76984	32.556	33.04	8.417	20.533	10.931	7.425	7.748	7.835	8.163	6.824	-1.47293	down	4.06E-13
LOC10175	1.051	0.647	0.043	0.034	0.021	0.026	0	0.045	0.007	0	-4.43954	down	5.03E-48
LOC11253	0	0	0.022	0.005	0.023	0	2.327	0.08	0.04	0.132	5.554031	up	1.06E-75
LOC10175	0.026	0.009	0.166	0.053	0	0.176	1.033	0.577	0.467	0.159	3.222193	up	1.11E-30
LOC10704	0.009	0.006	0.5	1.416	0.047	0.027	0	0.03	0	0.026	-4.49404	down	2.15E-56
LOC42702	0.23	0.276	0.823	0.912	0.236	0.371	0.392	1.45	1.139	1.816	1.059498	up	5.08E-05
SLC27A2	142.476	149.833	167.679	140.63	245.202	74.969	110.176	52.232	60.576	75.529	-1.1793	down	4.10E-08
LOC10085	1.032	1.377	7.206	2.828	7.798	0.588	1.332	1.031	1.474	0.579	-2.01504	down	3.95E-18
LOC10705	0.086	0.169	0.513	0.141	0.265	0.125	0.294	0.505	0.353	1.23	1.091273	up	0.000182
LOC10085	1.988	1.661	4.137	2.924	1.51	0.243	2.155	0.785	0.531	1.639	-1.19007	down	1.60E-07
CHIR-B6	0.241	0.032	0.077	0.357	0.099	0.187	0.701	1.009	0.149	1.185	1.996438	up	1.75E-09
LOC11252	0.324	0.593	0.438	0.16	0.632	1.159	0.995	2.959	0.199	0.159	1.347444	up	1.63E-07
LOC12110	0.064	0.063	0.305	1.494	1.179	0	0.136	0.458	0.327	0.443	-1.18379	down	5.63E-06
LOC10704	0.052	0	0.416	0.408	0.536	0.784	0.914	0.136	1.366	0.491	1.383125	up	8.86E-09
LOC12110	0.464	0.203	4.252	0.509	0.636	0.187	0.397	0.657	0.754	0.411	-1.33183	down	1.45E-06
LOC11253	0.417	0.26	6.651	2.071	3.179	0.557	1.424	0.596	0.387	0.941	-1.68624	down	5.64E-12
LOC11253	1.699	2.017	4.442	3.064	3.784	0.248	0.681	0.255	1.465	0.111	-2.44067	down	4.78E-26
LOC12110	0.429	0.345	1.971	0.678	1.017	0	0.677	0.172	0.068	0.486	-1.65854	down	7.53E-09
LOC10085	0.066	0.022	0	0.055	0	0.161	0.369	1.736	0.205	0.435	4.297846	up	5.25E-25
LOC10705	1.089	0.786	1.705	1.358	0.231	0.255	2.31	3.238	0.732	6.543	1.338341	up	7.77E-09
LOC11253	0.05	0.105	0.97	0.55	1.303	0.077	0.171	0.261	0.133	0.327	-1.61477	down	5.31E-11
LOC12110	0.657	0.706	0.224	0	0.145	0.045	0	1.042	0.193	4.259	1.67433	up	3.15E-09
LOC11253	0.038	0.149	0.303	0.219	0.586	0.147	0.422	0.844	0.273	2.052	1.525683	up	2.39E-08
LOC12110	2.839	1.928	2.347	2.692	1.346	0.951	1.212	1.141	1.792	0.179	-1.07934	down	5.26E-05
LOC10705	0	0	0	0	0	0.803	0	0	1.205	1.3	9.371994	up	4.16E-39
FKBP11	17.495	22.133	21.819	27.326	17.85	53.348	36.519	46.684	32.268	53.437	1.059668	up	4.44E-07
METTL7A	142.141	140.895	176.986	159.581	210.518	81.362	84.878	68.539	64.753	87.129	-1.10224	down	1.85E-07
TMPRSS12	1.529	1.699	1.16	1.257	2.294	0.564	0.933	0.856	0.473	0.615	-1.20494	down	1.59E-07
RACGAP1	0.029	0.075	0.275	0.201	0.207	0.613	0.27	3.944	1.563	1.023	3.227458	up	4.82E-37
ESPL1	0.205	0.159	0.262	0.182	0.222	0.235	0.314	1.532	0.488	0.566	1.601134	up	3.91E-12
LOC10704	0.392	0.257	0.769	0.198	0.434	0.891	0.67	0.886	0.727	1.011	1.027812	up	2.30E-06
LOC10704	0.692	0.763	1.247	0.958	0.748	1.305	0.716	9.372	2.952	2.83	1.960898	up	3.48E-16
LOC10085	40.202	42.39	33.643	30.324	42.627	71.89	47.656	137.771	65.496	61.431	1.022198	up	1.76E-06
TUBA1A	29.193	32.818	32.372	27.198	37.85	59.625	58.06	131.295	90.146	68.813	1.355394	up	1.60E-10
CNPY2	21.558	28.126	25.31	18.054	20.555	65.441	37.909	72.477	56.826	66.209	1.395439	up	3.28E-11
STAT2	5.504	5.392	6.718	5.234	3.53	8.069	6.997	17.212	14.459	16.101	1.252143	up	4.63E-09

APOF	37.366	50.766	36.826	63.533	23.793	100.585	136.168	77.884	78.845	183.641	1.442862	up	9.27E-12
TIMELESS	0.35	0.414	0.481	0.312	0.19	0.575	0.524	1.233	0.699	0.883	1.161483	up	4.07E-07
TAC3	0.091	0.208	1.231	0.99	1.378	0.11	0.466	0	0.07	0.074	-2.42853	down	7.27E-17
INHBE	4.017	4.779	3.518	12.377	2.036	13.692	35.12	11.971	12.613	34.148	2.008354	up	1.73E-20
LOC10704	0.24	0.196	0.191	0.123	0.185	0.174	0.307	1.369	0.584	0.318	1.552367	up	2.42E-07
LOC12110	0.594	1.033	0.032	0	0	0	0.046	0	0.014	0.022	-4.2575	down	2.71E-33
LOC11253	3.567	3.559	6.025	0.829	1.517	9.42	9.941	7.718	5.137	3.005	1.184186	up	3.77E-09
LOC12110	0.377	0.123	12.832	10.917	8.412	1.535	1.064	0.69	6.852	1.975	-1.43028	down	2.47E-10
LOC10705	2.671	2.885	17.939	1.035	2.537	1.733	1.657	2.995	2.792	3.586	-1.08427	down	5.68E-06
OTX5	3.385	3.379	6.24	1.429	2.499	0.521	0.486	0.622	0.265	0.398	-2.88236	down	1.66E-35
LOC10705	5.249	4.591	10.795	2.095	3.419	0.639	0.794	0.744	0.4	0.564	-3.05544	down	3.25E-36
SPIN1L	1.35	1.495	1.534	1.765	1.963	6.931	2.091	9.4	2.805	5.563	1.723834	up	9.98E-16
ALPK2	1.282	0.914	3.265	0.609	0.412	0.088	0.047	0.167	0.112	0.114	-3.60534	down	1.66E-48
SMAD7	1.084	1.035	3.634	4.505	3.217	1.524	0.946	1.395	1.055	1.617	-1.04302	down	1.09E-06
IFNW1	0.065	0	0.052	0	0.067	0	0	3.138	1.066	1.699	4.966212	up	5.10E-29
ENHO	11.656	11.328	6.264	7.549	2.636	38.459	21.654	28.036	31.097	35.812	1.975196	up	7.68E-20
ARID3C	2.61	2.312	1.969	1.971	1.86	7.631	8.78	4.192	4.958	6.598	1.5842	up	3.61E-14
CCL19	1.967	1.859	6.144	5.12	2.874	2.434	3.678	22.412	12.893	20.682	1.789176	up	1.84E-16
LOC10085	2.842	2.447	3.327	2.85	2.704	2.833	3.159	8.815	7.33	16.334	1.44061	up	3.44E-11
FANCG	0.22	0.24	0.281	0.374	0.589	0.242	0.515	1.382	0.603	0.721	1.020952	up	2.92E-05
AVDL	0.933	0.862	0.83	1.443	1.353	0.159	0.188	0.43	0.113	0.135	-2.39724	down	3.31E-15
AVD	0.679	0.578	0.703	2.345	0.488	1.513	1.103	17.213	18.815	16.443	3.521338	up	7.78E-44
CA9	1.104	1.293	0.961	2.131	3.296	0.174	0.768	1.006	0.816	0.294	-1.52092	down	3.08E-11
NPR3	0.084	0.139	0.074	0.152	0.188	0.814	0.403	0.275	0.372	1.029	2.174412	up	4.97E-23
CAPSL	11.653	11.464	1.396	10.224	0.591	0.506	7.677	0.52	0.899	0.514	-1.80366	down	3.75E-17
CCDC152	405.002	313.768	508.739	388.95	334.177	163.683	179.324	164.271	164.58	148.907	-1.2489	down	3.31E-09
SELENOP1	995.256	877.47	1165.874	1023.356	991.338	450.692	527.105	488.576	475.635	374.039	-1.12556	down	8.00E-08
NIM1K	237.897	193.624	200.797	136.927	290.768	89.321	100.359	105.526	131.896	52.534	-1.14406	down	4.76E-08
HMGCS1	1102.285	1000.148	825.11	665.005	1477.042	396.477	486.196	579.016	702.918	246.692	-1.07206	down	2.14E-07
DHX29	10.579	9.476	11.964	6.815	6.901	3.508	4.567	4.485	5.769	3.934	-1.03848	down	7.05E-07
CENPK	0.269	0.07	0.236	0.111	0.111	0.182	0.322	2.214	0.911	0.572	2.390775	up	4.51E-17
SGTB	3.827	3.292	3.296	3.897	3.128	7.595	6.528	9.443	8.984	9.625	1.273745	up	2.08E-09
BHMT2	524.771	561.713	292.981	788.682	2438.933	387.772	523.333	393.371	292.318	352.45	-1.24094	down	4.79E-09
DMGDH	52.099	51.451	39.368	61.156	111.39	42.722	55.152	20.31	13.838	21.11	-1.04268	down	1.72E-06
VLDLR	2.221	2.274	2.291	1.759	2.92	1.95	2.782	10.026	5.773	7.633	1.296243	up	2.80E-09
CD274	1.592	1.399	2.48	1.973	1.891	2.473	1.756	5.775	4.28	5.568	1.088153	up	3.23E-07
LOC11253	0.126	0.107	0.281	0.169	0.14	1.273	0.346	0.343	0.237	0.301	1.597108	up	9.05E-14
ALDH1A1	66.11	65.019	78.501	84.962	205.63	48.669	55.206	44.652	39.828	44.044	-1.10595	down	2.23E-07

FBP2	3.111	2.678	4.789	5.166	1.373	1.517	3.086	0.791	0.878	0.324	-1.37509	down	1.76E-09
ZNF367	0.557	0.425	0.429	0.595	0.964	1.142	1.127	4.028	1.601	1.033	1.58674	up	1.60E-12
GADD45G	9.213	10.267	11.004	14.629	6.816	35.534	21.074	59.043	92.264	131.587	2.70869	up	8.09E-34
TPPP2	1.417	1.78	1.445	2.702	3.261	1.263	0.581	1.111	0.989	1.252	-1.02856	down	1.58E-05
ADAMTS1	0.097	0.121	0.135	0.093	0.091	0.735	0.103	3.848	0.058	1.043	3.417697	up	1.56E-41
ISOC1	11.484	10.732	10.19	9.035	14.043	5.052	7.357	5.648	4.687	4.825	-1.00889	down	1.97E-06
PPIP5K2	14.486	14.036	15.709	20.013	19.902	5.93	10.251	9.432	6.174	8.079	-1.07764	down	2.00E-07
ST8SIA4	1.766	1.733	1.758	2.449	2.116	1.425	2.735	7.113	4.341	5.507	1.104197	up	1.64E-06
PCGF3	7.783	6.775	5.021	7.649	2.945	14.492	9.696	11.819	14.893	14.063	1.106232	up	2.19E-07
MFSD7	1.309	1.008	2.038	1.828	1.356	3.253	2.187	3.026	3.695	3.088	1.015786	up	1.13E-06
CHRNA6	0.017	0.044	0.641	0.041	0.586	0.358	1.118	0.132	1.153	0.014	1.059326	up	3.84E-07
LPL	1.707	1.894	5.175	0.471	5.173	0.041	0.063	0.222	0.065	1.119	-3.25118	down	1.13E-42
PSD3	7.349	6.967	7.679	4.194	8.275	0.871	0.92	1.34	1.671	0.743	-2.63474	down	9.55E-34
LMNB1	0.293	0.575	0.616	0.584	0.451	0.992	0.875	2.705	1.287	1.608	1.565783	up	4.18E-11
RHOBTB3	2.66	2.558	3.444	3.373	4.449	1.96	1.452	1.345	1.559	1.249	-1.12314	down	1.70E-07
ALDOB	11839.59	10520.93	7994.081	8633.508	7428.55	3593.159	3558.533	4356.686	3875.725	2281.676	-1.39369	down	1.27E-11
KIAA1958	30.304	27.952	9.523	15.084	9.131	37.834	50.683	53.443	54.268	48.349	1.410628	up	6.57E-11
SMC2	0.127	0.124	0.223	0.357	0.39	0.876	0.518	6.738	2.21	1.85	3.314495	up	3.23E-43
LPAR1	0.395	0.296	0.131	0.359	0.11	1.291	0.084	0.473	1.258	0.828	1.603764	up	5.50E-12
CDKN2A	0.782	0.648	0.831	0.964	1.152	1.919	2.072	5.279	2.889	4.968	1.967032	up	1.01E-17
CDKN2B	0.209	0.561	0.183	0.567	0.773	7.231	1.965	6.037	2.961	4.191	3.284404	up	8.38E-43
LOC10705	14.178	14.894	14.025	12.341	21.783	44.593	27.976	55.557	30.154	38.867	1.35215	up	1.23E-10
CHIR-IG1-	1.543	1.607	2.131	4.347	4.334	5.306	3.873	17.48	3.65	16.146	1.733967	up	6.42E-15
LOC10705	0.256	0.28	1.429	1.569	1.434	0.579	0.195	0.223	0.468	0.466	-1.36104	down	1.39E-07
LOC12110	0.014	0.009	1.624	1.345	1.382	0	0	0	0.014	0.284	-3.85321	down	4.61E-41
CDIPT	3.413	4.386	3.397	4.007	3.005	11.214	8.382	15.126	9.879	10.716	1.602885	up	5.43E-13
PRODH2	0.182	0.159	0.266	0.1	0.125	0.675	1.01	0.198	0.28	0.397	1.615659	up	3.51E-09
PKMYT1	0.051	0.117	0.041	0.126	0.235	0.469	0.283	1.364	0.941	0.729	2.720945	up	2.42E-20
LOC12110	0.534	1.017	0.426	4.591	1.247	1.647	0	0.127	0.15	0.715	-1.56445	down	4.30E-10
LOC12110	0.814	0.875	0.538	0.68	1.176	0.2	0.024	0.054	0	0.304	-2.79996	down	4.39E-27
LOC12110	1.881	1.885	2.629	1.665	1.527	1.134	0.73	1.065	0.607	1.119	-1.0415	down	1.99E-06
LOC11253	2.155	2.053	0.757	0.13	4.036	0.123	0.812	0.083	1.788	0.155	-1.62304	down	2.17E-11
LOC11253	0.16	0.992	0	0	0	0.077	0.613	1.146	0.164	0.587	1.163677	up	0.012824
LOC12110	1.528	1.676	3.059	0.441	6.025	7.88	8.963	10.845	7.034	7.331	1.723695	up	2.97E-16
LOC12110	0.892	1.389	1.082	0.558	0.741	1.768	1.668	2.107	2.7	2.326	1.179954	up	6.24E-07
LOC10705	0.496	0.46	0.513	0.25	0.032	0.685	1.238	1.853	0.645	1.397	1.72947	up	1.76E-15
LOC12110	0.374	0.77	0.852	0.703	2.033	1.347	1.416	6.427	3.347	1.925	1.61072	up	4.55E-11
HSD17B10	88.222	99.833	145.586	124.656	140.919	73.04	45.149	40.292	49.685	74.998	-1.08142	down	3.54E-07

LOC10704	3.842	3.525	4.839	2.729	2.295	10.879	2.997	3.628	11.718	13.525	1.310651	up	3.31E-10
FLNA	0.545	0.436	0.579	0.299	0.373	0.411	0.983	1.503	0.994	0.694	1.036929	up	0.000297
LOC10704	0.19	0.331	0.285	0.26	0.249	0.551	0.351	1.653	0.638	0.673	1.552168	up	9.74E-11
LOC1211C	0.997	0.47	0.794	1.803	0	0	0.034	0.365	0.082	0	-3.06565	down	5.02E-22
LOC1125З	2.993	2.829	7.512	3.722	2.373	1.278	1.196	0.758	0.946	1.024	-1.90006	down	2.01E-17
ZFPL1	0.205	0.737	0.489	0.253	0.105	0.694	1.05	1.07	0.21	1.005	1.169031	up	0.001035

qvalue	function
1.83E-27	LOC121113333; C-type lectin domain family 2 member B-like isoform X4
5.10E-13	LOC121106515; C-type lectin domain family 2 member D-like
6.16E-39	LOC776463; C-type lectin domain family 2 member B-like
2.77E-10	LOC112532872; C-type lectin domain family 2 member D-like isoform X3
2.60E-14	LOC121113330; endogenous retrovirus group K member 8 Gag polyprotein-like
3.94E-36	CD69L; C-type lectin domain family 2 member B
3.61E-14	SHANK3; LOW QUALITY PROTEIN: SH3 and multiple ankyrin repeat domains protein 3 isoform X1
5.70E-15	LOC121109250; BCL-6 corepressor-like protein 1 isoform X1
5.19E-06	ARF5; ADP-ribosylation factor 5
0.000181	NCAPH2; condensin-2 complex subunit H2 isoform X3
0.000347	STRIP2; striatin-interacting protein 2 isoform X2
5.54E-07	PFKFB3; 6-phosphofructo-2-kinase/fructose-2, 6-bisphosphatase 3 isoform X1
3.93E-13	MCM10; protein MCM10 homolog
2.89E-54	ELAPOR2; endosome/lysosome-associated apoptosis and autophagy regulator family member 2 isoform X1
1.72E-05	CACNA2D1; voltage-dependent calcium channel subunit alpha-2/delta-1 isoform X6
9.46E-08	FGL2; fibroleukin precursor
9.18E-06	ORC5; origin recognition complex subunit 5 isoform X1
6.94E-19	GTSE1; G2 and S phase-expressed protein 1
3.17E-11	ALG12; dol-P-Man:Man(7)GlcNAc(2)-PP-Dol alpha-1,6-mannosyltransferase isoform X1
8.41E-12	CRELD2; protein disulfide isomerase CRELD2 precursor
2.40E-06	ARL8BL; ADP-ribosylation factor-like 8B-like
1.90E-08	IQUB; IQ and ubiquitin-like domain-containing protein isoform X4
1.69E-19	CPED1; cadherin-like and PC-esterase domain-containing protein 1 isoform X4
1.22E-08	AMIGO2; amphoterin-induced protein 2 precursor
4.03E-26	SRGAP1; SLIT-ROBO Rho GTPase-activating protein 1 isoform X2
9.90E-06	CTSEAL; cathepsin E-A-like precursor
5.52E-07	PPFIA2; liprin-alpha-2 isoform X14
2.05E-07	SOCS2; suppressor of cytokine signaling 2
1.35E-07	IKBIP; inhibitor of nuclear factor kappa-B kinase-interacting protein isoform X1
5.19E-10	GAS2L3; GAS2-like protein 3 isoform X1
0.000195	OC3; uncharacterized protein LOC417943 precursor
0.000135	LOC121106438; histone H3
2.54E-10	LOC100857439; histone H3
0.038396	HIST1H2B7L4; histone H2B 1/2/3/4/6
2.93E-06	EMP1; epithelial membrane protein 1
8.77E-10	GUCY2C; heat-stable enterotoxin receptor isoform X3

6.88E-17 GBE; eye-globin isoform X1
4.68E-16 LOC417973; uncharacterized protein LOC417973 isoform X3
1.87E-05 CYP2D6; cytochrome P450 2D49
1.90E-09 MEI1; meiosis inhibitor protein 1 isoform X12
6.72E-09 CHADL; chondroadherin-like protein precursor
3.88E-22 ADSL; adenylosuccinate lyase
1.23E-51 NPTXR; neuronal pentraxin receptor
2.91E-06 DNAL4; dynein light chain 4, axonemal
2.28E-27 FAM20CL; extracellular serine/threonine protein kinase FAM20C isoform X1
1.17E-13 SUN2; SUN domain-containing protein 2
2.97E-06 SH3BP1; SH3 domain-binding protein 1
2.60E-16 CARD10; caspase recruitment domain-containing protein 10 isoform X7
8.13E-08 CSF2RB; cytokine receptor common subunit beta isoform X1
1.76E-17 MCM5; DNA replication licensing factor MCM5
5.01E-11 GLT8D2; glycosyltransferase 8 domain-containing protein 2 precursor
8.65E-08 HSP90B1; endoplasmin precursor
9.83E-24 ASCL1; achaete-scute homolog 1
3.20E-06 PAH; phenylalanine-4-hydroxylase
1.43E-15 IGF1; insulin-like growth factor I preproprotein
1.10E-16 PMCH; pro-MCH precursor
1.04E-05 DRAM1; DNA damage-regulated autophagy modulator protein 1 isoform X1
6.40E-39 SVOPL; putative transporter SVOPL isoform X3
5.23E-34 CGTL; cystine/glutamate transporter isoform X4
4.12E-07 RAB19; ras-related protein Rab-19
1.44E-05 TUBA8B; tubulin alpha-2 chain
1.33E-20 USP41; ubl carboxyl-terminal hydrolase 18 isoform X1
9.30E-09 AKR1E2; aldo-keto reductase family 1, member B1-like
3.85E-24 LOC425137; aldo-keto reductase family 1, member B1-like isoform X3
8.90E-53 SLCO1B1; solute carrier organic anion transporter family member 1C1 isoform X1
1.27E-27 LDHB; L-lactate dehydrogenase B chain isoform X1
7.17E-06 ETFRF1; electron transfer flavoprotein regulatory factor 1
1.66E-12 SSPN; sarcospan isoform X1
3.66E-12 BHLHE41; class E basic helix-loop-helix protein 41
6.28E-16 ENDOUL; poly(U)-specific endoribonuclease-A isoform X1
1.32E-14 SULT4A1; sulfotransferase 4A1 isoform X2
2.95E-20 PNPLA3; patatin-like phospholipase domain-containing protein 2 isoform X3
5.52E-13 PARVG; gamma-parvin
3.58E-08 KIAA0930; uncharacterized protein KIAA0930 homolog isoform X3

7.54E-17 MANSC1; MANSC domain-containing protein 1 precursor
2.74E-34 FOXM1; forkhead box protein M1
7.68E-19 A2ML1; alpha-2-macroglobulin-like protein 1 isoform X11
6.41E-15 CD86; T-lymphocyte activation antigen CD86 isoform X2
3.86E-18 GPR162; probable G-protein coupled receptor 162
2.55E-52 CDCA3; cell division cycle-associated protein 3 isoform 2
8.92E-14 USP5; ubiquitin carboxyl-terminal hydrolase 5 isoform X1
1.36E-07 LRRC23; leucine-rich repeat-containing protein 23 isoform X1
2.19E-12 EPHA1; ephrin type-A receptor 1 precursor
1.47E-11 GSTK1; glutathione S-transferase kappa 1 isoform X1
1.64E-20 KEL; kell blood group glycoprotein isoform X4
2.19E-12 LOC100859872; uncharacterized protein LOC100859872 isoform X1
6.66E-09 HSD3B1; hydroxy-delta-5-steroid dehydrogenase, 3 beta- and steroid delta-isomerase 1
1.72E-08 HAO2; hydroxyacid oxidase 2
1.71E-07 MAB21L3; protein mab-21-like 3 isoform X1
1.85E-08 SLC22A15; solute carrier family 22 member 15 isoform X3
1.18E-17 CLDND1; claudin domain-containing protein 1 isoform X2
5.95E-14 APOV1; apovitellenin-1 precursor
8.49E-17 SLC19A2; thiamine transporter 1
3.38E-05 COL8A1; collagen alpha-1(VIII) chain precursor
2.93E-32 KIAA1524; protein CIP2A homolog
7.57E-28 HJURP; Holliday junction recognition protein isoform X1
1.08E-09 LOC418414; uncharacterized protein C3orf85 homolog
1.69E-41 CD200L; uncharacterized protein LOC418424 precursor
1.78E-07 CREG1; protein CREG1 precursor
4.80E-08 MAEL; protein maelstrom homolog isoform X3
3.38E-05 LIPI; lipase member I isoform X2
2.77E-07 SAMSN1; SAM domain-containing protein SAMSN-1
8.98E-06 ADAMTS1; LOW QUALITY PROTEIN: A disintegrin and metalloproteinase with thrombospondin motifs 1 isoform X1
1.66E-12 MAP3K7CL; MAP3K7 C-terminal-like protein isoform X4
4.36E-07 EVA1C; protein eva-1 homolog C isoform X5
2.65E-07 CBR3; carbonyl reductase [NADPH] 1
3.62E-09 CHAF1B; chromatin assembly factor 1 subunit B isoform X1
4.26E-13 KCNJ15; ATP-sensitive inward rectifier potassium channel 15 isoform X2
5.57E-14 PCP4; calmodulin regulator protein PCP4
3.34E-55 MX1; interferon-induced GTP-binding protein Mx isoform X3
1.43E-19 SIK1; serine/threonine-protein kinase SIK2
2.20E-05 VTCN1L; V-set domain-containing T-cell activation inhibitor 1 isoform X1

2.91E-75 OTC; ornithine transcarbamylase, mitochondrial isoform X1
6.29E-25 NR0B1; nuclear receptor subfamily 0 group B member 1 isoform X2
4.47E-26 SLC51AL; organic solute transporter subunit alpha
4.60E-23 MAP3K15; mitogen-activated protein kinase kinase kinase 15
1.53E-21 PDHA2; pyruvate dehydrogenase E1 component subunit alpha, somatic form, mitochondrial precursor
1.89E-40 ADGRG2; adhesion G-protein coupled receptor G2 isoform X1
2.72E-20 GRPR; gastrin-releasing peptide receptor
2.15E-06 PIGA; phosphatidylinositol N-acetylglucosaminyltransferase subunit A
3.08E-07 LOC121106447; uncharacterized protein LOC121106447
1.66E-08 LOC107052718; LOW QUALITY PROTEIN: uncharacterized protein LOC107052718
1.03E-05 LOC418667; colony stimulating factor 2 receptor, alpha, low-affinity (granulocyte-macrophage) isoform X1
3.49E-47 CRLF2; cytokine receptor-like factor 2 isoform X2
2.92E-40 LYGL; lysozyme g isoform X1
1.45E-05 LYG2; lysozyme g isoform X1
2.45E-12 NPAS2; neuronal PAS domain-containing protein 2 isoform X6
2.83E-07 IL1R2; interleukin-1 receptor type 2 isoform X1
9.94E-18 IL1RL1; interleukin-1 receptor-like 1 isoform LV precursor
2.54E-08 IL18R1; interleukin-18 receptor 1 isoform X2
1.62E-06 LOC771012; coagulation factor X
2.52E-07 IRS2; insulin receptor substrate 2 isoform X1
1.57E-05 RAB20; ras-related protein Rab-20
3.52E-10 LOC121107687; SPARC
3.65E-14 GGACT; gamma-glutamylaminecyclotransferase isoform X2
2.32E-06 DOCK9; dedicator of cytokinesis protein 9 isoform X17
2.07E-15 HS6ST3; heparan-sulfate 6-O-sulfotransferase 3
1.02E-29 ACOD1; cis-aconitate decarboxylase
1.22E-22 BORA; protein aurora borealis isoform X6
3.10E-16 LOC112532134; uncharacterized protein LOC112532134 isoform X1
7.68E-29 DIAPH3; protein diaphanous homolog 3 isoform X3
1.04E-18 EPSTI1; epithelial-stromal interaction protein 1
8.28E-06 SLC25A30; kidney mitochondrial carrier protein 1 isoform X2
5.28E-07 HTR2A; 5-hydroxytryptamine receptor 2A
1.71E-05 PHF11; PHD finger protein 11 isoform X1
7.56E-06 NEK3; serine/threonine-protein kinase Nek3 isoform X2
8.78E-33 CKAP2; cytoskeleton-associated protein 2
9.80E-11 POSTN; periostin isoform X14
1.27E-05 SLC7A1; high affinity cationic amino acid transporter 1
6.88E-19 SKA3; spindle and kinetochore-associated protein 3 isoform X1

3.55E-07 ZC3H12C; probable ribonuclease ZC3H12C isoform X3
3.00E-06 ACAT1; acetyl-CoA acetyltransferase, mitochondrial
9.75E-58 MMP1; interstitial collagenase
0.000574 MMP7; matrilysin precursor
9.60E-07 BIRC3; inhibitor of apoptosis protein
3.03E-07 ENDOD1; endonuclease domain-containing 1 protein
1.02E-06 TAF1D; TATA box-binding protein-associated factor RNA polymerase I subunit D
6.87E-24 THRSPB; thyroid hormone responsive spot 14 beta
9.31E-10 THRSP; thyroid hormone-inducible hepatic protein
1.00E-19 CAPN5; calpain-5
2.22E-07 ART7B; uncharacterized protein LOC428124 precursor
0.000684 IL18BP; interleukin-18-binding protein isoform X2
3.23E-18 LOC121109083; glutamine-rich protein 2-like isoform X1
4.80E-06 SLCO2B1; solute carrier organic anion transporter family member 2B1 isoform X2
2.74E-07 KCNE3; potassium voltage-gated channel subfamily E member 3 isoform X2
2.00E-13 P2RY6; P2Y purinoceptor 3
2.71E-06 ART1; ecto-ADP-ribosyltransferase 5
7.50E-08 LOC107052463; olfactory receptor 51G2-like isoform X1
2.23E-19 HBE; hemoglobin subunit epsilon
3.65E-35 HBBA; hemoglobin subunit beta
3.60E-61 HBE1; hemoglobin subunit epsilon 1
4.56E-07 RRP8; ribosomal RNA-processing protein 8
1.12E-10 TAF10; transcription initiation factor TFIID subunit 10
3.43E-17 TPP1; tripeptidyl-peptidase 1
9.97E-18 AOC1; amiloride-sensitive amine oxidase [copper-containing] isoform X1
5.76E-05 LOC112529948; GTPase IMAP family member 9-like
2.19E-06 ELP6; elongator complex protein 6
4.06E-08 SCAP; sterol regulatory element-binding protein cleavage-activating protein isoform X2
1.70E-05 VIPR1; vasoactive intestinal polypeptide receptor 1 isoform X2
7.66E-14 ACKR2; atypical chemokine receptor 2
5.08E-17 CYP8B1; cytochrome P450, family 8, subfamily B
3.23E-07 CHDSD; D-threo-3-hydroxyaspartate dehydratase isoform X3
3.02E-11 CATH3; cathelicidin-3 precursor
4.86E-22 CATH2; cathelicidin-2 precursor
1.01E-19 CATH1; cathelicidin-1 precursor
2.41E-16 DLEC1; deleted in lung and esophageal cancer protein 1 isoform X3
6.57E-15 ACAA1; 3-ketoacyl-CoA thiolase, peroxisomal
3.64E-17 MAP3K8; mitogen-activated protein kinase kinase kinase 8 isoform X1

7.15E-08 MPP7; MAGUK p55 subfamily member 7 isoform X7
1.18E-40 LOC101750112; highly reducing polyketide synthase PKS6-like isoform X1
0.000283 MASTL; serine/threonine-protein kinase greatwall isoform X1
8.62E-06 OTUD1; OTU domain-containing protein 1
2.45E-19 ST8SIA6; alpha-2,8-sialyltransferase 8F
6.53E-06 RSU1; ras suppressor protein 1
2.99E-05 FAM188A; ubiquitin carboxyl-terminal hydrolase MINDY-3 isoform X3
9.28E-25 ACBD7; acyl-CoA-binding domain-containing protein 7 isoform X2
1.31E-25 OLAH; S-acyl fatty acid synthase thioesterase, medium chain isoform X1
3.89E-16 ABCB1LA; phosphatidylcholine translocator ABCB4 isoform X5
1.65E-06 CYP51A1; leucine-rich repeat and death domain-containing protein 1 isoform X2
1.13E-08 CDK6; cyclin-dependent kinase 6
1.40E-16 SAMD9L; sterile alpha motif domain-containing protein 9-like
4.17E-21 PDK4; pyruvate dehydrogenase kinase, isozyme 4
2.11E-13 ASNS; asparagine synthetase [glutamine-hydrolyzing]
1.80E-14 LOC395159; Schwann cell-specific EGF-like repeat autocrine factor precursor
1.41E-19 VWDE; von Willebrand factor D and EGF domain-containing protein
5.89E-11 IGF2BP3; insulin-like growth factor 2 mRNA-binding protein 3
1.47E-08 SNX10; sorting nexin-10 isoform X2
5.88E-09 HOXA2; homeobox protein Hox-A2
1.47E-06 WIPF3; WAS/WASL-interacting protein family member 3 isoform X2
3.74E-06 FKBP14; peptidyl-prolyl cis-trans isomerase FKBP14
1.00E-15 SATB1; DNA-binding protein SATB1 isoform X1
1.21E-24 SGO1; shugoshin 1
5.56E-06 LRRC3B; leucine-rich repeat-containing protein 3B
5.27E-07 EOMES; eomesodermin homolog isoform X1
9.83E-08 LOC101750511; BCL-6 corepressor-like
8.21E-12 ACAD11; acyl-CoA dehydrogenase family member 11
6.80E-06 CCR5; C-C chemokine receptor type 5 isoform X2
4.93E-06 CCR2; C-C chemokine receptor type 2
2.03E-31 KIF15; kinesin-like protein KIF15
1.99E-19 TRANK1; TPR and ankyrin repeat-containing protein 1 isoform X2
9.40E-05 _
2.97E-26 ANLN; anillin isoform X2
7.70E-30 AMPH; amphiphysin isoform X1
0.000513 LOC121109702; uncharacterized protein LOC121109702
2.77E-10 INHBA; inhibin beta A chain isoform X1
5.85E-07 EZH2; histone-lysine N-methyltransferase EZH2 isoform X5

1.90E-12	IGFBP1; insulin-like growth factor-binding protein 1 precursor
4.45E-05	TRIP13; pachytene checkpoint protein 2 homolog
5.06E-28	SALL3; sal-like protein 3 isoform X1
8.31E-11	DCDC2; doublecortin domain-containing protein 2 isoform X4
2.57E-10	NRSN1; neurensin-1
3.42E-45	ID4; DNA-binding protein inhibitor ID-4
1.28E-17	ELOVL2; elongation of very long chain fatty acids protein 2
1.14E-15	SYCP2L; synaptonemal complex protein 2-like isoform X3
3.83E-06	TXNDC5; thioredoxin domain-containing protein 5 isoform X2
1.29E-11	PPP1R3G; protein phosphatase 1 regulatory subunit 3G
8.07E-23	ECI2; enoyl-CoA delta isomerase 2 isoform X3
7.91E-13	SERPINB1; leukocyte elastase inhibitor isoform X1
3.15E-09	LOC420903; MARVEL domain-containing protein 3 isoform X2
4.01E-07	CTNND2; catenin delta-2 isoform X5
1.32E-24	CMBL; carboxymethylenebutenolidase homolog
6.43E-09	C5orf49; uncharacterized protein C5orf49 homolog isoform X3
9.18E-13	SRD5A1; 3-oxo-5-alpha-steroid 4-dehydrogenase 1 isoform X3
3.12E-05	LOC121109782; SUN domain-containing protein 3-like isoform X1
4.08E-09	MOCOS; molybdenum cofactor sulfurase isoform X1
1.40E-06	SEC61B; protein transport protein Sec61 subunit beta
#######	IFI6; interferon alpha inducible protein 6
1.10E-08	GLIPR2; Golgi-associated plant pathogenesis-related protein 1
2.95E-06	FAM65B; rho family-interacting cell polarization regulator 2
1.63E-05	LDLRAD4; low-density lipoprotein receptor class A domain-containing protein 4 isoform X8
1.81E-06	CIDEA; cell death activator CIDE-A
3.68E-06	TGIF1; homeobox protein AKR
1.18E-05	MYOM1; myomesin-1
4.80E-07	LPIN2; phosphatidate phosphatase LPIN2 isoform X1
9.73E-14	NDC80; kinetochore protein NDC80 homolog isoform X1
2.99E-06	COLEC12; collectin-12
1.44E-41	ABHD3; phospholipase ABHD3 isoform X1
2.04E-10	LAMA3; LOW QUALITY PROTEIN: laminin subunit alpha-3 isoform X1
3.16E-73	DSC1; desmocollin-2 isoform X4
2.13E-13	MCM4; DNA replication licensing factor MCM4
3.87E-10	C8orf22; pancreatic progenitor cell differentiation and proliferation factor-like protein
1.89E-24	CYP7A1; cholesterol 7-alpha-monooxygenase
1.37E-06	MTFR1; mitochondrial fission regulator 1 isoform X2
5.66E-28	XKR9; XK-related protein 9 isoform X2

1.60E-08 MSC; musculin
1.34E-23 TRPA1; transient receptor potential cation channel subfamily A member 1
4.51E-06 TERF1; telomeric repeat-binding factor 1
2.65E-13 PKIA; cAMP-dependent protein kinase inhibitor alpha
1.75E-11 FABP5; fatty acid binding protein 5
1.92E-09 FABP4; fatty acid-binding protein, adipocyte
1.02E-18 CA13; carbonic anhydrase 13
6.86E-52 ATP6V0D2; V-type proton ATPase subunit d 2
3.20E-10 PTDSS1; phosphatidylserine synthase 1 isoform X1
8.45E-12 CPQ; carboxypeptidase Q isoform X1
5.64E-07 LAPTM4B; lysosomal-associated transmembrane protein 4B isoform X1
1.94E-06 NCALD; neurocalcin-delta
7.71E-07 DCSTAMP; LOW QUALITY PROTEIN: dendritic cell-specific transmembrane protein
7.53E-06 ANGPT1; angiopoietin-1 precursor
9.87E-07 SAMD12; sterile alpha motif domain-containing protein 12 isoform X3
1.85E-06 SQLE; squalene monooxygenase
1.07E-06 FAM84B; protein LRATD2
1.12E-08 TMEM71; transmembrane protein 71 isoform X3
1.62E-05 NDRG1; protein NDRG1 isoform X1
1.11E-09 LY6E; lymphocyte antigen 6E precursor
6.34E-11 RHPN1; rhophilin-1 isoform X1
7.22E-06 TSTA3; GDP-L-fucose synthase isoform X2
4.46E-20 LOC121113091; uncharacterized protein LOC121113091
2.47E-12 BUB1; mitotic checkpoint serine/threonine-protein kinase BUB1
2.31E-07 LOC421232; GDNF-inducible zinc finger protein 1
4.56E-07 LOC101747556; uncharacterized protein LOC101747556
1.08E-11 CAPN13; calpain-13 isoform X2
1.66E-05 EHBP1; EH domain-binding protein 1 isoform X21
2.28E-74 WDPCP; WD repeat-containing and planar cell polarity effector protein fritz homolog isoform X5
2.82E-31 UGP2; UTP--glucose-1-phosphate uridylyltransferase isoform X1
4.37E-11 SLC1A4; neutral amino acid transporter A
1.09E-06 SLX4IP; protein SLX4IP
3.25E-12 BIRC5; survivin isoform 3
5.85E-08 RRBP1; ribosome-binding protein 1 isoform X2
1.72E-12 GINS1; DNA replication complex GINS protein PSF1
1.73E-19 TLR5; toll-like receptor 5 precursor
1.17E-08 ATF3; cyclic AMP-dependent transcription factor ATF-3
2.82E-34 DTL; denticleless protein homolog

4.26E-07	LPGAT1; acyl-CoA:lysophosphatidylglycerol acyltransferase 1 isoform X2
3.29E-09	LOC107052935; translation initiation factor IF-2-like
3.74E-06	LOC421419; uncharacterized protein LOC421419 isoform 2
2.04E-06	CMTR1; cap-specific mRNA (nucleoside-2'-O-)-methyltransferase 1
1.16E-08	NFKBIE; NF-kappa-B inhibitor epsilon
4.94E-09	EIF2AK2; interferon-induced, double-stranded RNA-activated protein kinase isoform X2
3.23E-23	GREM2; gremlin-2
8.92E-14	ACTN2; alpha-actinin-2
0.000146	SLC35F3; putative thiamine transporter SLC35F3 isoform X2
2.80E-05	CCSAP; centriole, cilia and spindle-associated protein
1.74E-07	C1orf131; uncharacterized protein C1orf131 homolog
1.17E-09	GNPAT; dihydroxyacetone phosphate acyltransferase isoform X1
1.60E-05	SULT; sulfotransferase
3.18E-20	DACT2; dapper homolog 2
1.17E-10	MPC1L; mitochondrial pyruvate carrier 1
0.00011	TBXT; brachyury protein
1.24E-10	LOC421583; solute carrier family 22 member 2
6.30E-22	FNDC1; fibronectin type III domain-containing protein 1
5.02E-07	EZR; ezrin
1.15E-31	LOC121110362; uncharacterized protein LOC121110362 isoform X1
4.25E-15	MTFR2; mitochondrial fission regulator 2 isoform X1
3.79E-13	SLC2A12; solute carrier family 2, facilitated glucose transporter member 12
5.17E-09	LOC107056139; pantetheinase-like precursor
9.11E-10	TMEM200A; transmembrane protein 200A
8.38E-30	CENPW; centromere protein W
5.54E-33	HEY2; hairy/enhancer-of-split related with YRPW motif protein 2
4.16E-21	GJA1; gap junction alpha-1 protein
7.85E-17	DCBLD1; discoidin, CUB and LCCL domain-containing protein 1 isoform X2
9.28E-17	LOC421740; sulfotransferase family 3A, member 1-like
7.03E-08	SLC16A10; monocarboxylate transporter 10 isoform X2
3.43E-08	DDO; D-aspartate oxidase
2.69E-16	LOC421792; thiosulfate sulfurtransferase/rhodanese-like domain-containing protein 3
5.23E-06	MMS22L; protein MMS22-like isoform X1
1.60E-08	NT5E; 5'-nucleotidase
2.90E-52	ME1; NADP-dependent malic enzyme
1.34E-32	RWDD2A; RWD domain-containing protein 2A
2.75E-18	TTK; dual specificity protein kinase TTK
2.81E-06	SH3BGRL2; SH3 domain-binding glutamic acid-rich-like protein 2 isoform X1

1.44E-07 GFRAL; GDNF family receptor alpha-like isoform X3
2.49E-05 LRRC1; leucine-rich repeat-containing protein 1 isoform X1
6.59E-24 CMPK2; UMP-CMP kinase 2, mitochondrial
5.79E-72 RSAD2; radical S-adenosyl methionine domain-containing protein 2
1.74E-06 ID2; DNA-binding protein inhibitor ID-2
9.16E-11 MBOAT2; lysophospholipid acyltransferase 2 isoform X1
3.86E-05 LOC421935; cystin-1
7.08E-55 RRM2; ribonucleoside-diphosphate reductase subunit M2
4.60E-09 VSNL1; visinin-like protein 1 isoform X2
3.88E-07 TTC32; tetratricopeptide repeat protein 32 isoform X2
3.28E-27 RHOB; rho-related GTP-binding protein RhoB
2.46E-09 APOB; apolipoprotein B precursor
1.41E-06 PRKACA; cGMP-dependent protein kinase 1-like isoform X2
2.13E-26 CGREF1; cell growth regulator with EF hand domain protein 1
9.29E-06 SLC5A6; sodium-dependent multivitamin transporter
2.43E-10 DTNB; dystrobrevin beta isoform X11
1.34E-28 KIF3C; kinesin-like protein KIF3C
7.56E-06 RAB10; ras-related protein Rab-10
1.61E-10 HADHB; trifunctional enzyme subunit beta, mitochondrial
9.47E-16 LOC121110416; protein aveugle-like isoform X1
1.84E-11 GVINP1; interferon-induced very large GTPase 1
3.87E-07 LOC770271; uncharacterized protein LOC770271
1.12E-05 LOC770996; L-gulonolactone oxidase
3.84E-20 C3H8ORF80; nuclear GTPase SLIP-GC
6.33E-10 NUGGC; nuclear GTPase SLIP-GC
2.60E-51 PBK; lymphokine-activated killer T-cell-originated protein kinase isoform X1
6.97E-11 LOC121110417; uncharacterized protein LOC121110417 isoform X1
2.06E-14 CLU; clusterin precursor
7.78E-06 ZNF395; zinc finger protein 395 isoform X2
2.58E-05 LOC426385; serine/threonine-protein kinase 35-like isoform X1
2.95E-09 MSRA; mitochondrial peptide methionine sulfoxide reductase isoform X14
1.27E-62 SOX7; transcription factor SOX-7
1.54E-07 XKR6; XK-related protein 6
2.55E-08 TDH; L-threonine 3-dehydrogenase, mitochondrial
3.90E-25 BLK; tyrosine-protein kinase Blk isoform X1
8.25E-41 AvBD13; gallinacin-13 precursor
4.78E-17 AvBD7; gallinacin-7 preproprotein
1.60E-20 DEFB4A; gallinacin-2 precursor

4.95E-19 AvBD1; gallinacin-1 alpha precursor
3.30E-30 MCM3; DNA replication licensing factor MCM3 isoform X1
1.10E-15 CRISP2; serotriflin isoform X1
1.79E-21 RHAG; ammonium transporter Rh type A
5.22E-28 CYP2AC1; cytochrome P450 2AC1
8.72E-12 SUPT3H; transcription initiation protein SPT3 homolog isoform X2
1.52E-07 RCAN2; calcipressin-2 isoform X2
3.31E-10 ADGRF5; adhesion G protein-coupled receptor F5
1.92E-21 ARR3; arrestin-C
8.66E-39 KIF4B; chromosome-associated kinesin KIF4
6.49E-06 LOC422147; putative P2Y purinoceptor 10
0.002016 RAB33A; RAB33A, member RAS oncogene family
2.38E-11 ERCC6L; DNA excision repair protein ERCC-6-like
7.56E-31 CCNB3; G2/mitotic-specific cyclin-B3 isoform X2
6.26E-35 CENPI; centromere protein I
1.50E-08 GCNA; acidic repeat-containing protein isoform X1
9.76E-08 SLC6A14; sodium- and chloride-dependent neutral and basic amino acid transporter B(0+) isoform X2
1.37E-06 FAM162BL; protein FAM162B isoform X2
4.25E-14 GPC3; glypican-3
4.87E-24 SLITRK4; SLIT and NTRK-like protein 4 isoform X2
1.09E-05 NSDHL; sterol-4-alpha-carboxylate 3-dehydrogenase, decarboxylating
9.58E-09 SLC16A2; monocarboxylate transporter 8
4.08E-08 IRS4; insulin receptor substrate 4
2.61E-19 COL4A5; collagen alpha-5(IV) chain isoform X1
1.39E-11 LOC121110638; uncharacterized protein LOC121110638
1.60E-12 SH2D1A; SH2 domain-containing protein 1A isoform X1
8.74E-05 GPR50; melatonin receptor type 1C
1.38E-05 MARCH1; E3 ubiquitin-protein ligase MARCHF1 isoform X1
1.00E-05 MSMO1; methylsterol monooxygenase 1
5.23E-26 DDX60; probable ATP-dependent RNA helicase DDX60
3.70E-07 MGARP; protein MGARP isoform X6
1.71E-16 TTC29; tetratricopeptide repeat protein 29 isoform X3
9.00E-09 TMEM154; transmembrane protein 154
1.27E-15 PLK4; serine/threonine-protein kinase PLK4 isoform X1
9.49E-11 CXCL13L3; C-X-C motif chemokine ligand 13-like 3 precursor
9.17E-06 CCNG2; cyclin-G2
8.46E-21 LOC422513; probable E3 ubiquitin-protein ligase HERC3 isoform X1
1.89E-73 MMRN1; multimerin-1

1.08E-16 ACSL1; long-chain-fatty-acid--CoA ligase 1
6.88E-05 WWC2; protein WWC2 isoform X1
3.09E-11 NEIL3; endonuclease 8-like 3 isoform X4
2.85E-07 HMGB2; high mobility group protein B2
8.83E-11 VEGFC; vascular endothelial growth factor C
3.76E-06 EREG; proepiregulin precursor
3.25E-05 AREG; amphiregulin isoform X1
4.42E-25 PLACL2; PLAC8 like 2
1.03E-09 GPAT3; glycerol-3-phosphate acyltransferase 3 isoform X1
2.47E-06 DUSP4; dual specificity protein phosphatase 4
8.85E-74 LOC430303; prolow-density lipoprotein receptor-related protein 1 isoform X3
2.25E-17 SHROOM3; protein Shroom3 isoform X7
1.20E-05 NAAA; N-acylethanolamine-hydrolyzing acid amidase isoform X1
5.45E-11 DCK; deoxycytidine kinase
2.81E-09 ADAMTS3; A disintegrin and metalloproteinase with thrombospondin motifs 3 isoform X3
3.01E-08 CENPC; centromere protein C
2.06E-11 SULT1B; sulfotransferase family, cytosolic, 1B isoform X1
1.68E-15 CCNA2; cyclin-A2
8.76E-10 MAD2L1; mitotic spindle assembly checkpoint protein MAD2A
1.55E-11 FABP2; fatty acid-binding protein, intestinal
3.27E-05 ARSJ; arylsulfatase J
6.17E-07 PITX2; pituitary homeobox 2
3.78E-09 ENPEP; glutamyl aminopeptidase
8.02E-07 CASP6; caspase-6
6.50E-06 C4orf17; uncharacterized protein C4orf17 homolog isoform X1
6.19E-07 DAPP1; dual adapter for phosphotyrosine and 3-phosphotyrosine and 3-phosphoinositide isoform X1
2.99E-40 CENPE; centromere-associated protein E isoform X3
2.54E-27 CCDC110; coiled-coil domain-containing protein 110
4.29E-13 TLR3; toll-like receptor 3 isoform X1
0.000378 LRRC66; leucine-rich repeat-containing protein 66 isoform X2
5.66E-15 UCHL1; ubiquitin carboxyl-terminal hydrolase isozyme L1
7.12E-10 FAM114A1; protein NOXP20 isoform X1
1.60E-07 TLR1A; toll-like receptor1 isoform X1
2.27E-08 TBC1D1; TBC1 domain family member 1
2.68E-26 RBPJ; recombining binding protein suppressor of hairless isoform X1
1.65E-06 SEL1L3; protein sel-1 homolog 3
4.68E-23 PPARGC1A; peroxisome proliferator-activated receptor gamma coactivator 1-alpha isoform X1
2.51E-22 NCAPG; condensin complex subunit 3 isoform X1

3.68E-06 BST1; ADP-ribosyl cyclase 2 isoform X3
5.15E-09 EVC2; limbin isoform X2
9.33E-13 HAUS3; HAUS augmin-like complex subunit 3
1.07E-07 TACC3; transforming acidic coiled-coil-containing protein 3 isoform X2
1.66E-43 SLBP; histone RNA hairpin-binding protein
2.83E-41 SPON2; spondin-2 isoform X2
7.89E-08 ATOH8; protein atonal homolog 8 isoform X1
2.68E-06 CD8A; T-cell surface glycoprotein CD8 alpha chain isoform X1
2.45E-17 LOC107049163; T-cell surface glycoprotein CD8 alpha chain-like isoform X1
2.43E-50 SMYD1; histone-lysine N-methyltransferase SMYD1
3.32E-05 CYP26B1; cytochrome P450 26B1 isoform X1
7.26E-07 NAT8; putative N-acetyltransferase 8B
3.17E-08 WDR54; WD repeat-containing protein 54
2.90E-08 LOC107056280; basic proline-rich protein-like isoform X3
5.13E-06 LOXL3; lysyl oxidase homolog 3 isoform X6
1.24E-11 LOC101752158; uncharacterized protein LOC101752158 isoform X1
0.000438 LOC107049267; glycine N-acyltransferase-like protein 3
9.05E-09 LOC107056412; membrane-spanning 4-domains subfamily A member 12-like isoform X3
1.41E-07 TMEM109; transmembrane protein 109
5.93E-06 TKFC; triokinase/FMN cyclase
1.66E-10 DAGLA; diacylglycerol lipase-alpha isoform X2
4.55E-09 TCIRG1; V-type proton ATPase 116 kDa subunit a3
4.40E-09 ALDH3B2; LOW QUALITY PROTEIN: aldehyde dehydrogenase family 3 member B1
2.72E-21 GPX2; glutathione peroxidase 2
4.15E-17 SPTB; spectrin beta chain, erythrocytic isoform X1
1.50E-37 CDHR5; cadherin-related family member 5 precursor
3.18E-37 BUB1B; mitotic checkpoint serine/threonine-protein kinase BUB1 beta
1.94E-05 C15orf52; coiled-coil domain-containing protein 9B isoform X6
2.51E-18 KNSTRN; small kinetochore-associated protein
1.35E-36 KNL1; kinetochore scaffold 1 isoform X3
4.74E-31 B4GALNT4; N-acetyl-beta-glucosaminyl-glycoprotein 4-beta-N-acetylgalactosaminyltransferase 1 isoform X1
7.22E-22 LOC770612; interferon-induced transmembrane protein 3
2.09E-08 LOC107053353; interferon induced transmembrane protein 2
2.91E-11 DHCR7; 7-dehydrocholesterol reductase
7.55E-24 E2F8; transcription factor E2F8 isoform X2
2.56E-18 KIF18A; kinesin-like protein KIF18A
7.61E-07 RCN1; reticulocalbin-1 isoform X1
3.89E-14 PRRG4; transmembrane gamma-carboxyglutamic acid protein 4 isoform X1

1.59E-06	DEPDC7; DEP domain-containing protein 7
1.51E-05	WEE1; wee1-like protein kinase
5.11E-06	PDE3B; cGMP-inhibited 3',5'-cyclic phosphodiesterase B
3.13E-64	SAA; serum amyloid A isoform X2
1.23E-19	TMEM86A; lysoplasmalogenase-like protein TMEM86A
1.54E-14	KCNQ1; potassium voltage-gated channel subfamily KQT member 1
#######	TRPM5; transient receptor potential cation channel subfamily M member 5 isoform X1
8.75E-06	SYT8; synaptotagmin-8
8.59E-10	CTSD; cathepsin D precursor
2.79E-26	DUSP8; dual specificity protein phosphatase 8 isoform X2
3.68E-08	PNPLA2; patatin-like phospholipase domain-containing protein 2
1.88E-13	LRRC56; leucine-rich repeat-containing protein 56 isoform X3
1.18E-08	RNH1; ribonuclease inhibitor isoform X2
2.80E-27	CHKA; choline kinase alpha isoform X2
2.07E-05	TESMIN; tesmin isoform X2
4.58E-21	CPT1A; carnitine O-palmitoyltransferase 1, liver isoform isoform X2
1.93E-36	LOC428851; acyl-CoA (8-3)-desaturase-like
2.19E-15	PGR2/3; P2X purinoceptor 3 precursor
1.45E-17	INCENP; inner centromere protein
5.65E-24	SMIM38; small integral membrane protein 38
3.03E-50	FGF19; fibroblast growth factor 19 precursor
2.17E-08	HSD17B12; very-long-chain 3-oxoacyl-CoA reductase
2.26E-08	C11orf96; uncharacterized protein C11orf96 homolog
5.93E-11	ACCS; 1-aminocyclopropane-1-carboxylate synthase-like protein 1 isoform X3
0.000125	TSPAN18; tetraspanin-18
7.20E-09	MDK; midkine isoform X1
1.40E-42	CHAC1; glutathione-specific gamma-glutamylcyclotransferase 1
2.03E-31	NUSAP1; nucleolar and spindle-associated protein 1
3.66E-08	EHD4; EH domain-containing protein 4
1.45E-05	PAPLN; papilin isoform X2
3.62E-13	GALNT16; polypeptide N-acetylgalactosaminyltransferase 16 isoform X2
2.90E-08	RDH11; retinol dehydrogenase 12
1.30E-28	LGALSL2; galectin-related protein A isoform X3
1.06E-09	SCG5; neuroendocrine protein 7B2 isoform X2
3.50E-18	LOC112531814; uncharacterized protein LOC112531814
7.37E-06	G2E3; G2/M phase-specific E3 ubiquitin-protein ligase isoform X5
7.14E-43	TTC6; tetratricopeptide repeat protein 6 isoform X3
3.50E-11	CLEC14A; C-type lectin domain family 14 member A

3.46E-22	ACSS1B; acetyl-coenzyme A synthetase 2-like, mitochondrial isoform X2
2.29E-09	RPS6KL1; LOW QUALITY PROTEIN: ribosomal protein S6 kinase-like 1 isoform X1
2.77E-27	FLVCR2; feline leukemia virus subgroup C receptor-related protein 2 isoform X2
3.30E-15	DIO2; type II iodothyronine deiodinase isoform a
2.27E-08	GALC; galactocerebrosidase isoform X2
5.13E-08	TTC8; tetratricopeptide repeat protein 8 isoform X2
7.10E-71	BDKRB1; B1 bradykinin receptor
8.04E-05	LOC121113247; uncharacterized protein LOC121113247
2.05E-41	AMN; protein amnionless isoform X1
3.15E-06	MTHFD1; C-1-tetrahydrofolate synthase, cytoplasmic
6.22E-06	ESR2; estrogen receptor beta
1.19E-11	DHRS7; dehydrogenase/reductase SDR family member 7
2.52E-53	ARMH4; armadillo-like helical domain-containing protein 4 isoform X12
5.32E-33	DLGAP5; disks large-associated protein 5
4.69E-09	WDHD1; WD repeat and HMG-box DNA-binding protein 1
8.12E-09	GCH1; GTP cyclohydrolase 1
4.88E-06	SOS2; son of sevenless homolog 2 isoform X2
7.12E-05	LOC112532661; uncharacterized protein LOC112532661
3.95E-06	LOC121106456; protein MANBAL-like
1.35E-18	GRID1; glutamate receptor ionotropic, delta-1 isoform X3
7.72E-24	SNCG; gamma-synuclein
2.19E-09	DNAJC12; dnaJ homolog subfamily C member 12
1.83E-05	LOC101748577; E3 SUMO-protein ligase EGR2
1.49E-10	CDK1; cyclin-dependent kinase 1 isoform X1
2.83E-10	RNASE6; angiogenin precursor
5.00E-07	PAPSS2; bifunctional 3'-phosphoadenosine 5'-phosphosulfate synthase 2 isoform X3
3.23E-18	ASAH2; LOW QUALITY PROTEIN: putative neutral ceramidase C isoform X1
5.40E-30	DNA2; DNA replication ATP-dependent helicase/nuclease DNA2
#######	HKDC1; hexokinase HKDC1 isoform X3
1.76E-20	CYP2C18; cytochrome P450 family 2 subfamily C member 18
7.47E-59	SCD; stearoyl-CoA desaturase
2.69E-16	CYP2C23b; cytochrome P450 2H2 precursor
2.28E-14	CYP2C23a; cytochrome P450 2H1 precursor
1.21E-11	MSMB; beta-microseminoprotein
3.54E-06	C10orf128; transmembrane protein 273 isoform X5
1.78E-19	ANKRD22; ankyrin repeat domain-containing protein 22 isoform X1
3.90E-08	ACTA2; actin, aortic smooth muscle isoform X1
1.50E-29	IFIT5; interferon-induced protein with tetratricopeptide repeats 5

6.39E-08 PANK1; pantothenate kinase 1 isoform X3
5.78E-10 KIF11; kinesin-like protein KIF11 isoform X2
7.70E-09 CPN1; carboxypeptidase N catalytic chain precursor
9.94E-10 LOXL4; lysyl oxidase homolog 4 precursor
9.23E-39 CRTAC1; cartilage acidic protein 1
4.92E-06 PI4K2A; LOW QUALITY PROTEIN: phosphatidylinositol 4-kinase type 2-alpha
3.34E-18 HOGA1; 4-hydroxy-2-oxoglutarate aldolase, mitochondrial
2.48E-06 ANKRD2; ankyrin repeat domain-containing protein 2 isoform X2
5.73E-21 ARHGAP19; rho GTPase-activating protein 19 isoform X1
6.57E-08 PROM1L; prominin-1-A
2.62E-34 TLX1; T-cell leukemia homeobox protein 1
1.48E-07 STN1; CST complex subunit STN1 isoform X4
3.79E-15 SFR1; swi5-dependent recombination DNA repair protein 1 homolog
8.66E-19 GPAM; glycerol-3-phosphate acyltransferase 1, mitochondrial isoform X1
1.31E-12 RGS10; regulator of G-protein signaling 10 isoform X2
2.15E-07 LOC101750892; deleted in malignant brain tumors 1 protein-like isoform X2
1.42E-15 CPXM2; inactive carboxypeptidase-like protein X2
3.54E-18 FAM196A; inhibitory synaptic factor 2A isoform X4
4.01E-42 MKI67; proliferation marker protein Ki-67 isoform X4
7.25E-14 ALDH18A1; delta-1-pyrroline-5-carboxylate synthase isoform X7
7.29E-07 FRZB; secreted frizzled-related protein 3 precursor
5.05E-29 BARD1; BRCA1-associated RING domain protein 1 isoform X1
3.10E-76 ABCA12; ATP-binding cassette sub-family A member 12 isoform X2
7.80E-30 ATIC; bifunctional purine biosynthesis protein ATIC
1.54E-26 LOC107048987; putative methyltransferase DDB_G0268948
8.16E-06 UGT1A1; UDP-glucuronosyltransferase 1A1 isoform X4
4.92E-46 LOC121111295; UDP-glucuronosyltransferase 1A1-like
4.72E-21 LOC121111296; UDP-glucuronosyltransferase 1A9-like
2.16E-07 TRAF3IP1; TRAF3-interacting protein 1 isoform X1
6.08E-12 AHR1B; aryl hydrocarbon receptor 1 beta isoform X2
3.44E-14 STAT1; signal transducer and activator of transcription 1-alpha/beta
8.66E-13 COQ10B; coenzyme Q-binding protein COQ10 homolog B, mitochondrial
6.19E-06 AOX1; aldehyde oxidase isoform X4
1.16E-06 ICOS; inducible T-cell costimulator isoform X1
4.28E-17 CTLA4; cytotoxic T-lymphocyte protein 4 precursor
3.82E-09 LOC424111; uncharacterized protein LOC424111
7.76E-08 IDH1; isocitrate dehydrogenase [NADP] cytoplasmic isoform X2
8.42E-06 PIKFYVE; 1-phosphatidylinositol 3-phosphate 5-kinase isoform X6

1.75E-15 PDE11A; dual 3',5'-cyclic-AMP and -GMP phosphodiesterase 11A isoform X3
2.85E-24 CDCA7; cell division cycle-associated protein 7 isoform X2
4.43E-08 RAPGEF4; rap guanine nucleotide exchange factor 4 isoform X5
1.28E-26 SPC25; kinetochore protein Spc25
4.36E-13 IFIH1; interferon-induced helicase C domain-containing protein 1
6.37E-13 DPP4; dipeptidyl peptidase 4
1.38E-23 SLC4A10; sodium-driven chloride bicarbonate exchanger isoform X6
1.02E-17 ITGB6; integrin beta-6 isoform X1
1.11E-32 LOC424199; uncharacterized protein LOC424199 isoform X1
9.18E-06 IHH; indian hedgehog protein precursor
2.66E-09 VIL1; villin-1
2.87E-51 IGFBP2; insulin-like growth factor-binding protein 2 isoform X1
2.57E-31 ARHGEF1; uncharacterized protein ARHGEF1 isoform X3
3.13E-12 EAF2; ELL-associated factor 2
9.91E-07 SEMA5B; semaphorin-5B isoform X5
2.50E-07 MYLK; myosin light chain kinase, smooth muscle isoform X3
6.99E-26 SLC12A8; solute carrier family 12 member 8 isoform X1
7.78E-06 PARP9; protein mono-ADP-ribosyltransferase PARP9 isoform X2
3.12E-05 SCTR; secretin receptor precursor
6.35E-18 DPP10; inactive dipeptidyl peptidase 10 isoform X2
8.83E-13 MCM6; DNA replication licensing factor MCM6
1.54E-08 KIF5C; kinesin heavy chain isoform 5C
2.65E-09 NMI; N-myc-interactor isoform X1
7.60E-19 UPP2; uridine phosphorylase 2 isoform X2
1.98E-11 NTNG1; netrin-G1 isoform X5
1.01E-18 ATP6V1G3; V-type proton ATPase subunit G 3
2.56E-05 ASPM; abnormal spindle-like microcephaly-associated protein isoform X3
1.50E-05 HTATIP2; oxidoreductase HTATIP2 isoform X1
0.001595 REG4; regenerating islet-derived protein 4 precursor
2.76E-07 PHGDH; D-3-phosphoglycerate dehydrogenase
3.50E-05 LOC100857694; flavin-containing monooxygenase 5-like
6.74E-76 LOC107053928; tumor necrosis factor ligand superfamily member 18-like
6.42E-09 FASLG; tumor necrosis factor ligand superfamily member 6 isoform X1
3.82E-17 SUCO; SUN domain-containing ossification factor isoform X5
1.10E-07 FMO3; flavin containing monooxygenase 3
1.90E-20 NUF2; kinetochore protein Nuf2 isoform X1
5.25E-15 CENPL; centromere protein L
6.05E-26 RGS8; regulator of G-protein signaling 8 isoform X2

1.40E-06	GLUL; glutamine synthetase
5.47E-08	SOAT1; sterol O-acyltransferase 1 isoform X4
3.38E-14	ABL2; tyrosine-protein kinase ABL2 isoform X2
1.76E-28	TOR3A; torsin-3A
2.04E-05	SERPINC1; antithrombin-III precursor
7.17E-06	NCF2; neutrophil cytosol factor 2
1.64E-07	PLPPR5; phospholipid phosphatase-related protein type 5 isoform X5
5.81E-06	MTF2; metal-response element-binding transcription factor 2 isoform X2
#######	CLCA1; calcium-activated chloride channel regulator 1-like
1.46E-15	SSX2IP; afadin- and alpha-actinin-binding protein isoform X3
1.65E-17	VTG2; vitellogenin-2 precursor
1.59E-54	LOC121111362; uncharacterized protein LOC121111362 isoform X1
1.18E-24	VTG3; vitellogenin-3
2.28E-08	SPATA1; spermatogenesis-associated protein 1 isoform X3
3.24E-09	THAP10; sterile alpha motif domain-containing protein 13 isoform X4
2.33E-23	VTG1; vitellogenin-1 precursor
1.65E-14	KIF2C; kinesin-like protein KIF2C isoform X10
4.24E-05	MMACHC; cyanocobalamin reductase / alkylcobalamin dealkylase
2.08E-24	RBP; riboflavin-binding protein precursor
3.75E-09	CYP4A22; cytochrome P450 4B1
2.47E-24	CYP4B7; cytochrome P450 4B7
2.58E-30	STIL; SCL-interrupting locus protein isoform X2
1.80E-23	PODN; podocan isoform X3
5.33E-06	DHCR24; delta(24)-sterol reductase
3.75E-18	DAB1; disabled homolog 1
4.88E-14	TACSTD2; tumor-associated calcium signal transducer 2 precursor
6.04E-31	ANGPTL3; angiopoietin-related protein 3 precursor
7.50E-09	PGM1; phosphoglucomutase-1 isoform X1
2.35E-07	DNAJC6; putative tyrosine-protein phosphatase auxilin
1.30E-05	GADD45A; growth arrest and DNA damage-inducible protein GADD45 alpha
8.63E-08	DEPDC1; DEP domain-containing protein 1A isoform 1
1.97E-08	NEU2; sialidase-2 isoform X3
8.57E-11	KLHL24; kelch-like protein 24 isoform X1
4.55E-26	EHHADH; peroxisomal bifunctional enzyme isoform X2
4.38E-06	DNAJB11; dnaJ homolog subfamily B member 11
1.11E-05	AMOTL2; angiomotin-like protein 2 isoform X2
2.86E-05	TM4SF19; transmembrane 4 L6 family member 19
1.80E-06	PER2; period circadian protein homolog 2 isoform X3

6.47E-14 CLSTN2; calsyntenin-2 isoform X2
8.33E-08 PXYLP1; 2-phosphoxylose phosphatase 1 isoform X1
2.70E-19 SGPP2; sphingosine-1-phosphate phosphatase 2
1.98E-11 MOGAT1; 2-acylglycerol O-acyltransferase 1
6.73E-07 GK5; putative glycerol kinase 5
4.06E-12 PCOLCE2; procollagen C-endopeptidase enhancer 2 isoform X1
6.10E-16 PLOD2; procollagen-lysine,2-oxoglutarate 5-dioxygenase 2 isoform X2
2.77E-07 LOC121113436; uncharacterized protein LOC121113436
5.07E-18 BDH1B; D-beta-hydroxybutyrate dehydrogenase, mitochondrial isoform X1
2.97E-10 HRASLS; phospholipase A and acyltransferase 1
7.29E-08 RTP2; receptor-transporting protein 2-like
4.40E-14 PDCD1; programmed cell death protein 1
4.05E-06 C2orf72; uncharacterized protein C2orf72 homolog
1.80E-05 CLCN2; chloride channel protein 2 isoform X2
5.44E-28 AHSG; alpha-2-HS-glycoprotein
8.88E-08 FETUB; fetuin-B precursor
9.67E-23 ECT2; protein ECT2 isoform X3
3.63E-08 LOC107054133; SLIT and NTRK-like protein 3
2.33E-25 SMC4; structural maintenance of chromosomes protein 4
1.34E-18 IFT80; intraflagellar transport protein 80 homolog
3.93E-09 MLF1; myeloid leukemia factor 1 isoform X1
1.78E-21 PTX3; pentraxin-related protein PTX3 precursor
1.06E-05 ARHGEF26; rho guanine nucleotide exchange factor 26
4.90E-15 SUCNR1; succinate receptor 1 isoform X3
2.11E-05 AADAC; arylacetamide deacetylase isoform X2
1.16E-67 DUOX1; dual oxidase 2
2.60E-10 KIAA0101; PCNA-associated factor
5.54E-23 PATL2; protein PAT1 homolog 2 isoform X1
3.41E-06 TERB2; telomere repeats-binding bouquet formation protein 2 isoform X1
3.95E-09 CD276; CD276 antigen isoform X1
4.54E-06 CYP1A2; cytochrome P450 1A5 isoform X1
4.15E-11 STOML1; stomatin-like protein 1 isoform X4
8.06E-11 PMLL; protein PML isoform X1
3.78E-11 LOC107052152; microtubule-associated protein 1S-like
2.26E-06 CHRNA3; neuronal acetylcholine receptor subunit alpha-3 precursor
5.52E-07 HYKK; hydroxylysine kinase
2.35E-06 IDH3A; isocitrate dehydrogenase [NAD] subunit alpha, mitochondrial isoform X1
4.83E-44 TRPM1; transient receptor potential cation channel subfamily M member 1 isoform X5

9.90E-12 CCNB1; G2/mitotic-specific cyclin-B2
6.15E-44 LIPC; hepatic triacylglycerol lipase isoform X1
1.31E-10 SCG3; secretogranin-3 isoform 2 precursor
1.26E-07 SEMA6D; semaphorin-6D isoform X8
7.26E-06 DUT; deoxyuridine 5'-triphosphate nucleotidohydrolase, mitochondrial
6.04E-07 FBN1; fibrillin-1 isoform X2
4.80E-06 TM6SF1; transmembrane 6 superfamily member 1 isoform X2
9.03E-06 LOC415472; uncharacterized protein LOC415472
8.83E-06 PEX11A; peroxisomal membrane protein 11A isoform X2
6.73E-06 TICRR; treslin
3.89E-11 MFGE8; lactadherin isoform 1 precursor
6.97E-08 ARRDC4; arrestin domain-containing protein 4
1.93E-20 PGPEP1L; pyroglutamyl-peptidase 1-like protein isoform X1
3.38E-06 LRRK1; leucine-rich repeat serine/threonine-protein kinase 1 isoform X2
3.34E-06 PCSK6; proprotein convertase subtilisin/kexin type 6 isoform X1
3.81E-05 RASL12; ras-like protein family member 12 isoform X1
1.38E-05 CORO2B; coronin-2B isoform X3
1.53E-07 PAQR5; membrane progestin receptor gamma
3.82E-55 KIF23; kinesin-like protein KIF23 isoform X18
1.58E-05 TMED3; transmembrane emp24 domain-containing protein 3 isoform 1 precursor
3.55E-11 PRC1; protein regulator of cytokinesis 1
3.56E-05 ADGRG5; adhesion G-protein coupled receptor G5 isoform X3
2.87E-87 CETP; cholesteryl ester transfer protein precursor
5.42E-32 LCAT; phosphatidylcholine-sterol acyltransferase precursor
8.03E-08 LOC415662; C-factor-like isoform X3
1.33E-22 LOC101747680; C-factor-like
2.16E-11 LOC112533303; C-factor-like
1.86E-07 SETD6; N-lysine methyltransferase SETD6 isoform X1
5.69E-23 MT4; metallothionein
4.13E-28 MT3; metallothionein-3
4.04E-09 SLC6A2; sodium-dependent noradrenaline transporter
3.26E-12 GPT2; alanine aminotransferase 2
1.31E-16 LOC100858381; uncharacterized protein LOC100858381 isoform X1
2.41E-08 RGS9BP; regulator of G-protein signaling 9-binding protein
8.98E-06 PDCD2L; programmed cell death protein 2-like
3.05E-35 CES1L2; fatty acyl-CoA hydrolase precursor, medium chain
5.89E-33 CES1L1; fatty acyl-CoA hydrolase precursor, medium chain isoform X1
3.18E-12 TERB1; telomere repeats-binding bouquet formation protein 1 isoform X3

1.81E-05 BEAN1; protein BEAN1
7.68E-07 CMC2; COX assembly mitochondrial protein 2 homolog isoform X2
1.00E-22 CENPN; centromere protein N
1.24E-16 LOC121111713; uncharacterized protein LOC121111713
1.42E-10 GINS2; DNA replication complex GINS protein PSF2 isoform X1
2.27E-08 SLC7A5; large neutral amino acids transporter small subunit 1
1.31E-19 CA5A; carbonic anhydrase 5A, mitochondrial isoform X2
2.41E-06 CIDEC; cell death activator CIDE-3 isoform X1
5.30E-06 MVD; diphosphomevalonate decarboxylase
2.84E-23 CDT1; DNA replication factor Cdt1 isoform X1
1.18E-10 FANCA; Fanconi anemia group A protein
3.64E-14 SPIRE2; protein spire homolog 2 isoform X1
3.12E-18 CHTF8; chromosome transmission fidelity protein 8 homolog
4.62E-15 PMFBP1; polyamine-modulated factor 1-binding protein 1 isoform X6
1.38E-30 LOC121111704; uncharacterized protein LOC121111704
9.50E-13 NT5DC2; 5'-nucleotidase domain-containing protein 2 isoform X1
1.69E-14 SMIM4; small integral membrane protein 4
8.57E-11 MUSTN1; musculoskeletal embryonic nuclear protein 1
5.81E-06 PRKCD; protein kinase C delta type isoform X2
3.20E-07 GBP4L; guanylate-binding protein 1 isoform X1
2.04E-08 LOC121106623; guanylate-binding protein 1-like
1.37E-11 LOC121106627; guanylate-binding protein 1-like
1.97E-07 GBP; guanylate binding protein
3.82E-09 LOC121106680; guanylate-binding protein 1-like
2.41E-06 GMPPB; mannose-1-phosphate guanyltransferase beta
5.24E-35 MST1R; macrophage-stimulating protein receptor isoform X1
2.43E-07 SLC38A3; sodium-coupled neutral amino acid transporter 3
1.43E-39 OASL; 59 kDa 2'-5'-oligoadenylate synthase-like protein
8.53E-09 ATP2B2; plasma membrane calcium-transporting ATPase 2 isoform X7
2.17E-06 CECR5L; haloacid dehalogenase-like hydrolase domain-containing 5
9.40E-18 DNASE1L3; deoxyribonuclease gamma precursor
7.65E-13 ABHD6; monoacylglycerol lipase ABHD6 isoform X2
9.16E-10 IP6K2; inositol hexakisphosphate kinase 2 isoform X1
1.45E-08 MCM2; DNA replication licensing factor MCM2 isoform X1
5.21E-11 CFAP100; cilia- and flagella-associated protein 100 isoform X1
3.26E-13 NTN4L; netrin-4
6.33E-10 PRRT3; proline-rich transmembrane protein 3 isoform X1
3.20E-08 FAM3D; protein FAM3D

1.28E-34	C3orf67; protein CFAP20DC isoform X1
2.58E-17	GPR27; probable G-protein coupled receptor 27
2.09E-34	PROK2; prokineticin-2
4.07E-06	BHLHE40; class E basic helix-loop-helix protein 40
4.02E-06	HBEGF; proheparin-binding EGF-like growth factor precursor
4.19E-12	KCNMB1; calcium-activated potassium channel subunit beta-1
#######	TENM2; teneurin-2 isoform 2
1.68E-20	HMMR; hyaluronan mediated motility receptor isoform X1
1.13E-25	PTTG2; securin isoform 1
2.40E-12	LOC416147; E3 ubiquitin/ISG15 ligase TRIM25-like
8.17E-08	DUSP1; dual specificity protein phosphatase 1
3.23E-17	STC2; stanniocalcin-2
2.00E-08	MXD3; lateral signaling target protein 2 homolog isoform X3
5.45E-11	LOC101751218; max dimerization protein 3 isoform X3
6.25E-13	GRIA1; glutamate receptor 1 precursor
2.45E-09	GPX3; glutathione peroxidase 3 precursor
1.50E-05	MRNIP; MRN complex-interacting protein isoform X1
1.61E-25	LECT2; myeloid protein 1 precursor
2.54E-07	SLC22A5; solute carrier family 22 member 5
1.41E-07	IRF1; interferon regulatory factor 1 isoform X1
3.64E-11	LEAP2; liver-expressed antimicrobial peptide 2 precursor
1.37E-06	YIPF5; protein YIPF5
4.86E-15	GFRA3; GDNF family receptor alpha-3
1.89E-15	EGR1; early growth response protein 1
1.31E-33	BHLHA15; class A basic helix-loop-helix protein 15
1.69E-18	NPTX2; neuronal pentraxin-2 isoform X1
0.000103	PDGFA; platelet-derived growth factor subunit A preproprotein
3.51E-07	CYP3A5; cytochrome P450 family 3 subfamily A member 5
1.21E-05	RNF216; E3 ubiquitin-protein ligase RNF216
2.07E-13	LOC101747873; urotensin-2 receptor
4.05E-06	PLD6; mitochondrial cardiolipin hydrolase
2.01E-07	PEMT; phosphatidylethanolamine N-methyltransferase isoform X2
1.10E-30	CACNA1H; voltage-dependent T-type calcium channel subunit alpha-1H isoform X2
1.16E-10	SOX8; transcription factor SOX-8
2.38E-06	MEIOB; meiosis-specific with OB domain-containing protein isoform X1
2.03E-31	PLK1; serine/threonine-protein kinase PLK1
2.68E-09	IL21R; interleukin-21 receptor precursor
5.47E-06	SOCS1; suppressor of cytokine signaling 1

1.87E-06	CIITA; MHC class II transactivator isoform X5
2.39E-13	CARHSP1; calcium-regulated heat-stable protein 1
3.82E-14	HBZ; hemoglobin subunit pi
1.61E-36	HBAD; hemoglobin subunit alpha-D
5.82E-36	HBA1; hemoglobin subunit alpha-A
1.55E-77	LOC416655; protein PERCC1 isoform X1
1.65E-06	ZKSCAN7; zinc finger protein 436 isoform X3
5.46E-09	NME4; nucleoside diphosphate kinase, mitochondrial
4.65E-06	DECR2; peroxisomal 2,4-dienoyl-CoA reductase [(3E)-enoyl-CoA-producing] isoform X1
0.000636	LOC121106810; cyclin-F-like isoform X1
1.65E-05	CCNF; cyclin-F isoform X1
3.32E-10	ACSM4; acyl-coenzyme A synthetase ACSM4, mitochondrial
8.55E-09	LOC121106758; uncharacterized protein LOC121106758
1.34E-06	ERI2; ERI1 exoribonuclease 2 isoform X3
7.20E-30	CDC45; cell division control protein 45 homolog isoform X2
9.11E-10	CLDN5; claudin-5
6.57E-08	COMT; catechol O-methyltransferase
6.56E-08	RIMBP2; RIMS-binding protein 2 isoform X14
4.63E-20	AACS; acetoacetyl-CoA synthetase
6.41E-15	ATP2A2; sarcoplasmic/endoplasmic reticulum calcium ATPase 2 isoform 2
2.99E-09	P2RX7; P2X purinoceptor 7
2.29E-11	HPD; 4-hydroxyphenylpyruvate dioxygenase
1.26E-05	LRRC43; leucine-rich repeat-containing protein 43
1.83E-13	RAD9B; cell cycle checkpoint control protein RAD9B isoform X2
5.21E-09	PPTC7; protein phosphatase PTC7 homolog
8.52E-68	ACACB; acetyl-CoA carboxylase 2 isoform X5
9.49E-12	FAM222A; protein FAM222A isoform X3
2.88E-10	CHCHD10; coiled-coil-helix-coiled-coil-helix domain-containing protein 10, mitochondrial
1.76E-11	MMP11; stromelysin-3
4.71E-09	SLC2A11; solute carrier family 2, facilitated glucose transporter member 11
7.50E-22	SLC2A11L1; solute carrier family 2, facilitated glucose transporter member 11-like
2.58E-20	LOC769554; solute carrier family 2, facilitated glucose transporter member 11
9.49E-12	LOC101751878; solute carrier family 2, facilitated glucose transporter member 11
6.91E-10	TBX6; T-box-containing protein TBX6L isoform X1
3.18E-16	LOC416959; melanotransferrin isoform X2
4.66E-19	PISD; phosphatidylserine decarboxylase proenzyme, mitochondrial isoform X1
2.03E-06	LOC100858984; dynein light chain 2, cytoplasmic
1.12E-42	CIT; citron Rho-interacting kinase isoform X14

9.80E-38 SLC35E4; solute carrier family 35 member E4
1.42E-17 TCN2; transcobalamin-2
1.83E-08 HORMAD2; HORMA domain-containing protein 2 isoform X9
3.54E-14 LOC417013; acyl-CoA dehydrogenase family member 11 isoform X2
6.41E-05 LOC121106953; uncharacterized protein LOC121106953 isoform X1
1.66E-08 MHCY14; major histocompatibility complex-Y, class I heavy chain, 14 precursor
4.64E-10 LOC121106942; C-type lectin domain family 2 member I-like isoform X5
1.58E-13 MHCY11; major histocompatibility complex-Y, class I heavy chain 11 precursor
9.39E-11 MHCY9; major histocompatibility complex Y, class I heavy chain 9 isoform X3
4.10E-43 LENG9L7; leukocyte receptor cluster member 9-like, MHC-Y region, 7
8.88E-08 MHCY15; major histocompatibility complex-Y, class I heavy chain, 15 precursor
9.36E-05 MHCY7; major histocompatibility complex-Y, class I heavy chain, 7 precursor
5.05E-44 LOC100859628; class I histocompatibility antigen, F10 alpha chain-like isoform X6
7.99E-31 LOC121106918; class I histocompatibility antigen, F10 alpha chain-like isoform X2
3.15E-07 LOC121106502; C-type lectin domain family 2 member I-like isoform X5
1.62E-25 LOC121106943; C-type lectin domain family 2 member E-like isoform X1
1.55E-05 LOC121106922; class I histocompatibility antigen, F10 alpha chain-like isoform X4
2.84E-18 LOC121106928; class I histocompatibility antigen, F10 alpha chain-like isoform X1
7.49E-08 MHCY8; major histocompatibility complex Y, class I heavy chain 8 isoform X2
6.68E-08 MHCY6; LOW QUALITY PROTEIN: major histocompatibility complex Y, class I heavy chain 6
8.67E-26 MHCY32; major histocompatibility complex Y, class I heavy chain 32
2.37E-43 LOC121106920; uncharacterized protein LOC121106920 isoform X18
2.78E-07 BTN3A3L1; erythroid membrane-associated protein-like isoform X4
1.02E-15 KIFC1; kinesin-like protein KIFC1 isoform X2
1.40E-16 IL4I1; L-amino-acid oxidase precursor
6.23E-08 TRIM7.1; tripartite motif-containing protein 7 isoform X5
3.69E-09 TRIM27.1; tripartite motif-containing 27 isoform X1
1.90E-07 BLB1; Major histocompatibility complex class II beta chain BLB1, (similar to HLA class II, D beta chain) precursor
2.01E-08 DMA; B locus M alpha chain 1 precursor
6.03E-07 DMB2; major histocompatibility complex, class II, DM beta 2 isoform X1
1.76E-05 BF1; MHC BF1 class I isoform X1
4.09E-06 TAP1; antigen peptide transporter 1
0.001614 CYP21A1; steroid 21-hydroxylase precursor
8.32E-18 LOC121106935; collagen alpha-1(I) chain-like
6.58E-10 LOC107049645; uncharacterized protein LOC107049645 isoform X2
4.19E-07 MAN1B1; endoplasmic reticulum mannosyl-oligosaccharide 1,2-alpha-mannosidase isoform X2
1.74E-10 EXFABP; extracellular fatty acid-binding protein precursor
7.55E-06 STPG3; protein STPG3 isoform X1

5.00E-37 NSMF; NMDA receptor synaptonuclear signaling and neuronal migration factor isoform X2
6.01E-10 BRINP1; BMP/retinoic acid-inducible neural-specific protein 1 precursor
7.10E-08 LOC107052201; alpha-1-acid glycoprotein
8.89E-11 SLC25A25; calcium-binding mitochondrial carrier protein SCaMC-2 isoform X4
3.74E-05 AK1; adenylate kinase isoenzyme 1 isoform X2
1.59E-23 FPGS; folylpolyglutamate synthase, mitochondrial isoform X9
8.95E-06 DNM1; dynamin-1 isoform X8
1.18E-06 PHYHD1; phytanoyl-CoA dioxygenase domain-containing protein 1
1.24E-13 LOC417192; torsin family 1, member B-like precursor
6.32E-08 ASS1; argininosuccinate synthase
4.67E-21 LAMC3; laminin subunit gamma-3 isoform X3
5.50E-08 FAM69B; divergent protein kinase domain 1B isoform X1
7.13E-38 AGPAT2; 1-acyl-sn-glycerol-3-phosphate acyltransferase beta isoform X2
2.65E-13 LOC417113; carboxyl-terminal PDZ ligand of neuronal nitric oxide synthase protein
2.16E-06 HSPA5; endoplasmic reticulum chaperone BiP isoform X1
2.62E-11 MVB12B; multivesicular body subunit 12B isoform X2
2.74E-06 PIK3R5; phosphoinositide 3-kinase regulatory subunit 5 isoform X1
1.70E-05 HS3ST3B1L; heparan sulfate glucosamine 3-O-sulfotransferase 3B1
1.39E-31 HS3ST3A1; heparan sulfate glucosamine 3-O-sulfotransferase 3A1
1.12E-07 FN3K; fructosamine-3-kinase isoform X2
9.36E-18 MXRA7; matrix-remodeling-associated protein 7 isoform X3
4.45E-05 ST6GALNAC1; alpha-N-acetylgalactosaminide alpha-2,6-sialyltransferase 1 isoform X1
4.27E-18 TEN1; CST complex subunit TEN1 isoform X2
1.27E-15 ACOX1; peroxisomal acyl-coenzyme A oxidase 1 isoform X3
5.89E-06 MRPL38; 39S ribosomal protein L38, mitochondrial
1.02E-14 SMIM5; small integral membrane protein 5
7.21E-14 MYO15L; myosin XVB isoform X2
3.19E-28 FASN; fatty acid synthase
1.57E-06 TRIM25; E3 ubiquitin/ISG15 ligase TRIM25
1.48E-19 KPNA2; importin subunit alpha-1
0.000105 CACNG1; voltage-dependent calcium channel gamma-1 subunit
4.34E-08 SDK2; protein sidekick-2 isoform X2
2.33E-10 RNF213; E3 ubiquitin-protein ligase RNF213 isoform X1
8.17E-39 CBX2; chromobox protein homolog 2
2.05E-40 RBFOX3; RNA binding protein fox-1 homolog 3 isoform X8
5.30E-11 ENPP7; ectonucleotide pyrophosphatase/phosphodiesterase family member 7
1.18E-20 LOC121107135; uncharacterized protein LOC121107135
7.14E-18 TIMP2; metalloproteinase inhibitor 2 precursor

6.91E-35 SOCS3; suppressor of cytokine signaling 3
1.10E-29 TK1; thymidine kinase, cytosolic
2.39E-10 LOC100857706; uncharacterized protein LOC100857706 isoform X1
2.40E-07 ARL16; ADP-ribosylation factor-like protein 16 isoform X1
2.46E-16 KRABZFP; uncharacterized protein LOC769812 precursor
1.92E-06 GRIN2C; glutamate receptor ionotropic, NMDA 2C isoform X3
2.85E-06 OTOP3; proton channel OTOP3
2.38E-11 SLC16A5; monocarboxylate transporter 6
5.43E-54 CCL4; chemokine C-C motif ligand 4 precursor
1.95E-13 GATSL2; cytosolic arginine sensor for mTORC1 subunit 2 isoform X7
4.16E-25 MIS12; protein MIS12 homolog
1.51E-06 TMEM120A; ion channel TACAN
2.38E-06 MMP28; matrix metalloproteinase-28 isoform X1
3.59E-11 LOC121106433; C-C motif chemokine 3-like
4.39E-43 SERPINF1; pigment epithelium-derived factor isoform X1
9.64E-10 SRR; serine racemase isoform X7
2.59E-08 SEBOX; homeobox protein SEBOX isoform X1
4.65E-28 SLC13A2; solute carrier family 13 member 2
2.87E-26 SPAG5; sperm-associated antigen 5 isoform X2
2.90E-26 CORO6; coronin-6 isoform X2
1.68E-06 GSG2; serine/threonine-protein kinase haspin
6.44E-06 ALDH3A2; aldehyde dehydrogenase family 3 member A2 isoform X1
1.36E-92 DOC2B; double C2-like domain-containing protein beta isoform X1
2.77E-12 PRR11; proline-rich protein 11 isoform X1
1.83E-07 VMP1; vacuole membrane protein 1 isoform X2
1.17E-08 MED13; mediator of RNA polymerase II transcription subunit 13 isoform X3
3.18E-19 ACACA; acetyl-CoA carboxylase isoform X1
8.02E-07 C17orf78; uncharacterized protein C17orf78
2.66E-07 TADA2A; transcriptional adapter 2-alpha isoform X1
1.10E-16 DUSP14; dual specificity protein phosphatase 14
5.88E-11 EVI2A; protein EVI2A precursor
8.06E-06 CLUH; clustered mitochondria protein homolog isoform X2
4.77E-07 LOC100857331; tapasin-related protein isoform X2
5.40E-07 FAM64A; uncharacterized protein FAM64A isoform X5
4.83E-13 FBXO39; F-box only protein 39 isoform X2
1.20E-05 TEKT1; tektin-1 isoform X2
1.78E-12 LOC107057310; protein NDRG3 isoform X2
3.50E-05 ASIP; agouti-signaling protein precursor

5.80E-07 TOX2; TOX high mobility group box family member 2 isoform X1
2.00E-05 ACSS2; acetyl-coenzyme A synthetase, cytoplasmic isoform X8
1.42E-09 FAM83D; protein FAM83D-B
5.30E-06 RPN2; dolichyl-diphosphooligosaccharide--protein glycosyltransferase subunit 2 precursor
1.09E-23 RBPJL; recombining binding protein suppressor of hairless-like protein isoform X3
2.04E-24 PI3; elafin
8.48E-07 TTPAL; alpha-tocopherol transfer protein-like isoform X1
1.35E-13 GDAP1L1; ganglioside-induced differentiation-associated protein 1-like 1
2.18E-19 RPESL; somatomedin-B and thrombospondin type-1 domain-containing protein isoform X1
2.15E-14 EPB42; protein 4.2
1.03E-14 ZNFX1; LOW QUALITY PROTEIN: NFX1-type zinc finger-containing protein 1 isoform X1
7.47E-18 GATA5; transcription factor GATA-5 isoform X1
4.59E-69 COL20A1; collagen alpha-1(XX) chain precursor
7.08E-11 HELZ2; helicase with zinc finger domain 2
2.79E-05 SLC52A3; solute carrier family 52, riboflavin transporter, member 3
3.33E-16 TPX2; targeting protein for Xklp2 isoform X4
1.73E-08 IRF9; interferon regulatory factor 9
1.06E-36 BPIFB3; BPI fold containing family B member 3 isoform X3
2.43E-14 LOC771972; uncharacterized protein LOC771972 isoform X1
1.04E-08 LOC100859272; WAP four-disulfide core domain protein 2 isoform X1
5.62E-27 WFDC2; WAP four-disulfide core domain protein 3 isoform X4
6.82E-30 WFDC8; balbiani ring protein 3 isoform X2
3.51E-24 SPINT4; kunitz-type protease inhibitor 4
9.01E-13 LOC771994; protein qua-1
1.27E-41 UBE2U; ubiquitin-conjugating enzyme E2 C
3.74E-17 MMP9; matrix metalloproteinase-9 precursor
9.70E-71 PCK1; phosphoenolpyruvate carboxykinase, cytosolic [GTP]
1.45E-05 AURKB; aurora kinase A isoform X2
7.46E-06 UTS2; urotensin-2 precursor
3.67E-47 ESPN; espin isoform X6
1.07E-58 AJAP1; adherens junction-associated protein 1
5.83E-06 KBP; probable glutamate receptor isoform X1
3.01E-08 MMP23A; matrix metalloproteinase-23 isoform X2
1.72E-10 MIB2; E3 ubiquitin-protein ligase MIB2
1.24E-55 LOC419409; Golgi integral membrane protein 4 isoform X1
2.27E-07 FNDC10; fibronectin type III domain-containing protein 10
1.55E-05 CCNL2; cyclin-L1 isoform X1
1.36E-34 LOC771069; C-factor isoform X2

2.64E-07 CENPS; centromere protein S
3.13E-21 RNF186; E3 ubiquitin-protein ligase RNF186
2.57E-10 CAMK2N1; calcium/calmodulin-dependent protein kinase II inhibitor 1
3.28E-12 CELA2A; chymotrypsin-like elastase family member 2A precursor
1.64E-18 DRAXIN; draxin isoform X1
1.17E-09 EPHB2; ephrin type-B receptor 2 precursor
7.73E-08 P3H1; prolyl 3-hydroxylase 1 precursor
1.14E-10 ECE1; endothelin-converting enzyme 1 isoform X4
2.31E-11 LOC112530142; uncharacterized protein LOC112530142
6.84E-09 DPYSL2; dihydropyrimidinase-related protein 2 isoform X2
2.39E-05 CDCA2; cell division cycle-associated protein 2
6.36E-12 EGR3; LOW QUALITY PROTEIN: early growth response protein 3
5.61E-05 LOC107050638; vinexin isoform X1
1.30E-12 DMTN; dematin isoform X2
5.38E-06 LOC101749127; uncharacterized protein LOC101749127 isoform X1
2.03E-21 ADAM32L2; disintegrin and metalloproteinase domain-containing protein 32
1.19E-39 IDO2; indoleamine 2,3-dioxygenase 2 isoform X2
2.32E-20 ANK1; ankyrin-1 isoform X13
9.12E-11 NCAPH; condensin complex subunit 2 isoform X2
2.57E-07 ADD2; beta-adducin
7.72E-12 SLC20A1; sodium-dependent phosphate transporter 1
5.45E-17 CASP14; caspase-14
1.48E-21 RETSAT; all-trans-retinol 13,14-reductase
4.93E-52 FABP3; fatty acid-binding protein, heart
3.46E-08 SERINC2; serine incorporator 2 isoform X1
5.23E-10 GBP1; guanylate-binding protein 1
8.39E-07 THEMIS2; protein THEMIS2
1.11E-08 LOC101749201; peptide methionine sulfoxide reductase MsrA isoform X3
1.07E-05 MTFR1L; mitochondrial fission regulator 1-like isoform X2
2.94E-06 TRNAU1AP; tRNA selenocysteine 1-associated protein 1 isoform X4
0.000161 CTGFL; CCN family member 2 isoform X2
7.58E-24 STMN1; stathmin isoform X1
1.60E-17 PAQR7; membrane progestin receptor alpha isoform X1
7.19E-05 DNALI1; axonemal dynein light intermediate polypeptide 1
8.64E-09 CSF3R; granulocyte colony-stimulating factor receptor precursor
2.57E-07 CLSPN; claspin isoform X3
1.42E-61 IFI27L2; interferon alpha-inducible protein 27, mitochondrial
1.22E-05 TMEM35B; transmembrane protein 35B

2.07E-05 NFYC; nuclear transcription factor Y subunit gamma isoform X8
1.97E-25 MYCL; protein L-Myc
2.01E-18 GALE; UDP-glucose 4-epimerase isoform X1
6.93E-22 HMGCL; hydroxymethylglutaryl-CoA lyase, mitochondrial isoform 4
2.97E-10 GRHL3; grainyhead-like protein 3 homolog isoform X4
2.27E-37 IL22RA1; interleukin-22 receptor subunit alpha-1 precursor
1.30E-05 LOC107057415; uncharacterized protein LOC107057415 isoform X2
1.62E-05 SPA17; sperm surface protein Sp17
4.78E-06 CHEK1; serine/threonine-protein kinase Chk1 isoform X1
1.09E-05 C20orf173; CMP-N-acetylneuraminate-beta-galactosamide- alpha-2,3-sialyltransferase 4 isoform X3
1.31E-18 TMEM45L; transmembrane protein 45B isoform X2
3.12E-23 NCAPD3; condensin-2 complex subunit D3 isoform X3
9.50E-12 SCN3B; sodium channel subunit beta-3 isoform X1
1.14E-06 CRTAM; cytotoxic and regulatory T-cell molecule isoform X1
1.68E-10 ZBTB32; zinc finger and BTB domain-containing protein 16
1.97E-10 ZPR1; zinc finger protein ZPR1 isoform X2
1.23E-15 APOA5; apolipoprotein A-V
8.01E-20 FXYD2; sodium/potassium-transporting ATPase subunit gamma
1.33E-10 SCN4B; sodium channel subunit beta-4 isoform X3
4.51E-06 JAML; junctional adhesion molecule-like isoform X1
3.71E-05 MPZL2; myelin protein zero-like protein 2 precursor
1.51E-14 LOC101748511; uncharacterized protein LOC101748511 isoform X4
7.77E-06 HYOU1; hypoxia up-regulated protein 1 isoform X1
2.18E-05 PLET1; uncharacterized protein PLET1
1.11E-05 IL18; interleukin-18
3.58E-13 DIXDC1; dixin isoform X6
4.49E-30 LOC101749531; LOW QUALITY PROTEIN: ras GTPase-activating-like protein IQGAP3
3.48E-07 LOC101747704; sperm-associated antigen 4 protein-like isoform X3
3.93E-06 LOC101750908; SLAM family member 8 isoform X3
1.17E-05 CD244; natural killer cell receptor 2B4 isoform X2
9.37E-06 CD48; CD48 antigen isoform X1
7.11E-10 SLAMF1; signaling lymphocytic activation molecule
1.72E-18 CKS1B; cyclin-dependent kinases regulatory subunit 1
4.34E-05 ECM1; extracellular matrix protein 1
1.13E-06 CIART; circadian-associated transcriptional repressor isoform X1
7.90E-05 CA14; carbonic anhydrase 14 isoform X3
7.59E-07 PCP4L1; Purkinje cell protein 4 like 1
3.26E-13 APOA2; apolipoprotein A-II isoform X1

1.10E-08	TMOD4; tropomodulin-4
1.84E-05	LOC121107543; acyl-coenzyme A thioesterase THEM4-like isoform X2
9.69E-11	LOC121107545; prefoldin subunit 2-like
4.01E-20	THEM4; acyl-coenzyme A thioesterase THEM4 isoform X2
1.86E-11	LOC121106470; deaminated glutathione amidase-like isoform X7
6.88E-65	PFDN2; prefoldin subunit 2 isoform X1
8.61E-29	TULP1; tubby-related protein 1 isoform X1
6.16E-12	FKBP5; peptidyl-prolyl cis-trans isomerase FKBP5 isoform X1
2.67E-21	ELF3; ETS-related transcription factor Elf-3
6.57E-29	CSRP1; cysteine and glycine-rich protein 1
0.000108	TMEM9; proton-transporting V-type ATPase complex assembly regulator TMEM9 isoform X2
3.77E-07	LOC395100; cryptochrome 4 isoform X1
7.76E-16	UBE2T; ubiquitin-conjugating enzyme E2 T isoform X2
2.05E-05	SHISA4; protein shisa-4
1.80E-13	LOC107050775; epidermal growth factor receptor kinase substrate 8-like protein 3 isoform X1
2.60E-06	KCNA3; potassium voltage-gated channel subfamily A member 3 isoform X1
3.44E-13	NUAK2; NUAK family SNF1-like kinase 2
1.27E-08	LOC121107619; Krueppel-like factor 15 isoform X1
3.44E-19	SLC45A3; solute carrier family 45 member 3
#######	SLC26A9; solute carrier family 26 member 9
2.73E-19	FAM72A; protein FAM72A isoform X1
6.40E-33	CAMK1G; calcium/calmodulin-dependent protein kinase type 1G
6.26E-37	LAMB3; laminin subunit beta-3
2.01E-15	G0S2; G0/G1 switch protein 2
7.62E-14	GUCA1A; guanylyl cyclase-activating protein 1
8.44E-09	MOV10; putative helicase MOV-10 isoform X1
1.67E-16	SLC16A1; monocarboxylate transporter 1
5.62E-06	SYCP1; synaptonemal complex protein 1 isoform X1
1.00E-11	TSHB; thyrotropin subunit beta isoform X1
2.03E-16	PACSIN1; protein kinase C and casein kinase substrate in neurons protein 1
3.40E-07	ITPR3; inositol 1,4,5-trisphosphate receptor type 3 isoform X1
1.64E-13	TSPO2; translocator protein 2
3.97E-14	CHIA-M31; acidic mammalian chitinase isoform X1
3.87E-12	LOC768786; acidic mammalian chitinase isoform X1
4.29E-06	FMOD; fibromodulin precursor
8.71E-07	GTSF1; gametocyte-specific factor 1 isoform X1
4.04E-07	DBF4B; protein DBF4 homolog B isoform X3
7.22E-26	KIF18B; kinesin-like protein KIF18B

4.24E-05 C1QL1; C1q-related factor isoform X1
3.33E-05 GH; somatotropin precursor
2.19E-08 MRC2; C-type mannose receptor 2
4.67E-29 SLC4A1; band 3 anion transport protein isoform X3
6.28E-08 C27H17ORF105; uncharacterized protein C17orf105 homolog
1.48E-07 RAB18L; ras-related protein Rab-18-B-like
3.10E-07 PNPO; pyridoxine-5'-phosphate oxidase
1.78E-06 SRCIN1; SRC kinase signaling inhibitor 1 isoform X6
1.34E-05 CISD3; CDGSH iron-sulfur domain-containing protein 3, mitochondrial
3.86E-05 PPP1R1B; protein phosphatase 1 regulatory subunit 1B isoform X1
1.36E-10 CSF3; myelomonocytic growth factor precursor
1.16E-11 CDC6; cell division control protein 6 homolog
6.80E-06 RARA; retinoic acid receptor alpha
5.56E-55 TOP2A; DNA topoisomerase 2-alpha
1.95E-11 CCR7; C-C chemokine receptor type 7 precursor
1.17E-08 P3H4; endoplasmic reticulum protein SC65
9.98E-36 BRCA1; breast cancer type 1 susceptibility protein isoform X5
2.23E-06 ANGPTL4; angiopoietin-related protein 4
4.66E-10 LOC100857637; transducin-like enhancer protein 1 isoform X3
2.11E-05 CELF5; CUGBP Elav-like family member 5 isoform X3
1.57E-08 MPND; MPN domain-containing protein isoform X3
3.27E-09 STAP2; signal-transducing adaptor protein 2 isoform X2
1.49E-09 NMRK2; nicotinamide riboside kinase 2
5.90E-48 ACSBG2; long-chain-fatty-acid--CoA ligase ACSBG2 isoform X4
1.20E-08 MEX3D; RNA-binding protein MEX3D
2.27E-06 LRG1; leucine-rich alpha-2-glycoprotein isoform X1
9.78E-10 CREB3L3; cyclic AMP-responsive element-binding protein 3-like protein 3 isoform X2
4.32E-07 MIDN; midnolin-A isoform X1
8.95E-12 GAMT; guanidinoacetate N-methyltransferase isoform X1
3.20E-05 PCSK4; proprotein convertase subtilisin/kexin type 4 isoform X3
1.26E-40 ADAMTSL5; ADAMTS-like protein 5 isoform X3
3.92E-16 LOC100859819; cocaine- and amphetamine-regulated transcript protein isoform X5
5.47E-11 USHBP1; Usher syndrome type-1C protein-binding protein 1 isoform X4
3.63E-16 LOC420107; uncharacterized protein LOC420107 isoform X1
2.82E-15 LOC107055361; liprin-alpha-2
5.42E-21 LOC121107782; zinc finger protein 707-like isoform X1
4.26E-13 LOC121107774; zinc finger protein 34-like isoform X1
5.78E-14 ACP5; tartrate-resistant acid phosphatase type 5 isoform X1

1.27E-05 LOC107049412; microtubule-associated serine/threonine-protein kinase 1 isoform X1
1.04E-05 LIG1; DNA ligase 1
9.30E-12 LOC769841; sulfotransferase 2B1 isoform X2
1.39E-45 LOC101751912; butyrophilin subfamily 3 member A2-like isoform X8
9.65E-73 LOC112531052; cingulin-like isoform X38
9.76E-29 LOC101751153; E3 ubiquitin-protein ligase TRIM39-like isoform X7
8.36E-54 LOC107049467; uncharacterized protein LOC107049467 isoform X2
0.000359 LOC427029; uncharacterized protein LOC427029 isoform X3
5.18E-07 SLC27A2; bile acyl-CoA synthetase
1.40E-16 LOC100859870; leukocyte immunoglobulin-like receptor subfamily A member 2
0.001129 LOC107050189; platelet glycoprotein VI-like
1.88E-06 LOC100857615; platelet glycoprotein VI isoform X1
2.64E-08 CHIR-B6; immunoglobulin-like receptor CHIR-B6 precursor
1.91E-06 LOC112529946; T-cell-interacting, activating receptor on myeloid cells protein 1-like
4.94E-05 LOC121107862; platelet glycoprotein VI-like
1.23E-07 LOC107049967; uncharacterized protein LOC107049967 isoform X1
1.43E-05 LOC121107894; platelet glycoprotein VI-like isoform X1
1.14E-10 LOC112531135; platelet glycoprotein VI-like
3.10E-24 LOC112531119; platelet glycoprotein VI-like isoform X5
1.05E-07 LOC121107865; LOW QUALITY PROTEIN: leukocyte immunoglobulin-like receptor subfamily A member 2
3.12E-23 LOC100857597; osteoclast-associated immunoglobulin-like receptor isoform X1
1.08E-07 LOC107050473; LOW QUALITY PROTEIN: platelet glycoprotein VI-like isoform X1
9.72E-10 LOC112531212; leukocyte immunoglobulin-like receptor subfamily A member 2 isoform X4
4.63E-08 LOC121107883; T-cell-interacting, activating receptor on myeloid cells protein 1-like
3.12E-07 LOC112531328; LOW QUALITY PROTEIN: platelet glycoprotein VI-like
0.000371 LOC121107930; CLK4-associating serine/arginine rich protein-like
6.19E-37 LOC107056248; olfactory receptor 14J1-like
4.82E-06 FKBP11; peptidyl-prolyl cis-trans isomerase FKBP11
2.15E-06 METTL7A; methyltransferase-like protein 7A
1.87E-06 TMPRSS12; transmembrane protease serine 12 isoform X1
6.35E-35 RACGAP1; rac GTPase-activating protein 1 isoform X3
8.09E-11 ESPL1; LOW QUALITY PROTEIN: separin isoform X1
2.17E-05 LOC107049666; integrin beta-7 isoform X2
1.06E-14 LOC107049862; uncharacterized protein LOC107049862
1.70E-05 LOC100859737; tubulin alpha-1B chain
2.79E-09 TUBA1A; tubulin alpha-1 chain
6.13E-10 CNPY2; protein canopy homolog 2
6.66E-08 STAT2; signal transducer and activator of transcription 2 isoform X2

1.82E-10 APOF; apolipoprotein F
4.44E-06 TIMELESS; LOW QUALITY PROTEIN: protein timeless homolog
2.30E-15 TAC3; tachykinin-3 isoform X2
7.60E-19 INHBE; inhibin beta E chain
2.74E-06 LOC107049257; pH-response regulator protein palI/RIM9-like
2.82E-31 LOC121106503; maestro heat-like repeat-containing protein family member 2A isoform X2
5.50E-08 LOC112531456; uncharacterized protein LOC112531456 isoform X1
4.20E-09 LOC121108153; cold shock protein CS66-like
4.97E-05 LOC107055390; mRNA decay activator protein ZFP36-like isoform X1
2.01E-33 OTX5; cone-rod homeobox protein isoform X1
4.02E-34 LOC107050328; uncharacterized protein LOC107050328
2.94E-14 SPIN1L; spindlin-W isoform X3
4.76E-46 ALPK2; alpha-protein kinase 2
1.09E-05 SMAD7; mothers against decapentaplegic homolog 7 isoform X1
4.05E-27 IFNW1; interferon type B precursor
3.18E-18 ENHO; adropin
9.07E-13 ARID3C; AT-rich interactive domain-containing protein 3C isoform X2
5.69E-15 CCL19; C-C motif chemokine 19 precursor
6.41E-10 LOC100857191; C-C motif chemokine 26
0.000218 FANCG; Fanconi anemia group G protein isoform X1
9.17E-14 AVDL; avidin isoform X1
1.67E-41 AVD; avidin precursor
5.78E-10 CA9; carbonic anhydrase 9 isoform X2
2.65E-21 NPR3; atrial natriuretic peptide receptor 3 isoform X4
1.23E-15 CAPSL; calcyphosin-like protein isoform X2
4.85E-08 CCDC152; coiled-coil domain-containing protein 152
9.74E-07 SELENOP1; selenoprotein P precursor
5.95E-07 NIM1K; serine/threonine-protein kinase NIM1 isoform X2
2.45E-06 HMGCS1; hydroxymethylglutaryl-CoA synthase, cytoplasmic isoform X2
7.36E-06 DHX29; ATP-dependent RNA helicase DHX29
1.46E-15 CENPK; centromere protein K
3.11E-08 SGTB; small glutamine-rich tetratricopeptide repeat-containing protein beta
6.88E-08 BHMT2; betaine--homocysteine S-methyltransferase 1
1.65E-05 DMGDH; dimethylglycine dehydrogenase, mitochondrial isoform X2
4.13E-08 VLDLR; very low-density lipoprotein receptor isoform X1
3.59E-06 CD274; programmed cell death 1 ligand 1 isoform X2
2.20E-12 LOC112532140; uncharacterized protein LOC112532140
2.55E-06 ALDH1A1; retinal dehydrogenase 1

2.66E-08 FBP2; fructose-1,6-bisphosphatase isozyme 2
3.42E-11 ZNF367; zinc finger protein 367
8.99E-32 GADD45G; growth arrest and DNA damage-inducible protein GADD45 gamma
0.000125 TPPP2; tubulin polymerization-promoting protein family member 2
2.70E-39 ADAMTS19; A disintegrin and metalloproteinase with thrombospondin motifs 19 isoform X2
1.88E-05 ISOC1; isochorismatase domain-containing protein 1
2.30E-06 PPIP5K2; inositol hexakisphosphate and diphosphoinositol-pentakisphosphate kinase 2 isoform X1
1.59E-05 ST8SIA4; CMP-N-acetylneuraminate-poly-alpha-2, 8-sialyltransferase
2.49E-06 PCGF3; polycomb group RING finger protein 3
1.13E-05 MFSD7; solute carrier family 49 member A3
4.20E-06 CHRNA6; neuronal acetylcholine receptor subunit alpha-6 isoform X2
2.12E-40 LPL; lipoprotein lipase precursor
1.05E-31 PSD3; PH and SEC7 domain-containing protein 3 isoform X4
7.75E-10 LMNB1; lamin-B1
1.98E-06 RHOBTB3; rho-related BTB domain-containing protein 3 isoform X3
2.47E-10 ALDOB; fructose-bisphosphate aldolase B
1.18E-09 KIAA1958; uncharacterized protein KIAA1958 homolog isoform X1
6.60E-41 SMC2; structural maintenance of chromosomes protein 2
1.12E-10 LPAR1; lysophosphatidic acid receptor 1 isoform X1
3.46E-16 CDKN2A; ARF tumor suppressor
1.63E-40 CDKN2B; cyclin-dependent kinase 4 inhibitor B
2.17E-09 LOC107050717; calumenin isoform X1
1.74E-13 CHIR-IG1-5; immunoglobulin-like receptor CHIR-AB1-like precursor
1.65E-06 LOC107050652; putative killer cell immunoglobulin-like receptor-like protein KIR3DX1
7.69E-39 LOC121108719; uncharacterized protein LOC121108719 isoform X2
1.22E-11 CDIPT; CDP-diacylglycerol--inositol 3-phosphatidyltransferase isoform X1
5.13E-08 PRODH2; hydroxyproline dehydrogenase isoform X1
1.05E-18 PKMYT1; membrane-associated tyrosine- and threonine-specific cdc2-inhibitory kinase isoform X2
7.08E-09 LOC121108741; vasodilator-stimulated phosphoprotein-like isoform X1
3.09E-25 LOC121108762; uncharacterized protein LOC121108762 isoform X1
1.90E-05 LOC121108855; uncharacterized protein LOC121108855 isoform X2
4.13E-10 LOC112530182; soluble scavenger receptor cysteine-rich domain-containing protein SSC5D-like
0.043145 LOC112530947; uncharacterized protein LOC112530947
9.07E-15 LOC121108875; uncharacterized protein LOC121108875 isoform X6
6.56E-06 LOC121108907; guanine nucleotide-binding protein G(s) subunit alpha isoforms XLas-like
5.04E-14 LOC107051395; mucin-2-like isoform X1
8.40E-10 LOC121108911; DNA replication licensing factor MCM7-like
3.90E-06 HSD17B10; 3-hydroxyacyl-CoA dehydrogenase type-2 isoform X2

5.52E-09	LOC107049485; guanine nucleotide-binding protein-like 3-like protein isoform X2
0.001726	FLNA; filamin-A
1.74E-09	LOC107049632; uncharacterized protein LOC107049632 isoform X14
2.48E-20	LOC121108923; mental retardation GTPase activating protein homolog 4-like
6.75E-16	LOC112530071; NF-kappa-B inhibitor delta-like isoform X1
0.005161	ZFPL1; LOW QUALITY PROTEIN: zinc finger protein-like 1 isoform X1
