# Supplementary material for: Comparative analysis of liver transcriptome reveals adaptive responses to hypoxia environmental condition in Tibetan chicken
Source: Anim Biosci. 2023 Aug 23;37(1):28–38. doi: 10.5713/ab.23.0126 (PMC10766467; doi:10.5713/ab.23.0126)
Supplement: Supplementary file 7 [file ab-23-0126-Supplementary-Table-7.pdf]

Table S7

##total input seqs number with gos: 280

namespac	descriptio	number	percent	id	genes
biological_	reproduct	45	0.1607	GO:00000	A2ML3;ABCB1LA;ACOD1;ACOX1;ADAM15;ADGRG2;AHR1B;ASCL1;BUB1;CBX2;CDK6;CDT1;CYP
biological_	immune sy	47	0.1678	GO:00023	A2ML3;ACAA1;ACKR2;ACOD1;ADD2;ADGRF5;AGPAT2;ANK1;AVD;CDK6;CDKN2B;CDT1;CREG1;
molecular_	catalytic a	112	0.4	GO:00038	ABCA12;ABCB1LA;ABHD3;ABHD6;ACAA1;ACACA;ACACB;ACAD11;ACOD1;ACOX1;ACSBG2;ACS
molecular_	structural	6	0.0214	GO:00051	ADD2;ANK1;CARD10;EPB42;IFT80;LAMB3
molecular_	transporte	26	0.0928	GO:00052	ABCA12;ABCB1LA;APOF;ATP6V0D2;CACNA1H;CETP;CHRNA3;FABP2;FABP4;GJA1;GRIN2C;HBB
molecular_	binding	183	0.6535	GO:00054	A2ML3;ABCA12;ABCB1LA;ACACA;ACACB;ACAD11;ACKR2;ACOX1;ADAM15;ADAMTSL5;ADD2;A
cellular_cc	intracellul	233	0.8321	GO:00056	A2ML3;ABCA12;ABCB1LA;ABHD6;ACAA1;ACACA;ACACB;ACAD11;ACKR2;ACOD1;ACOX1;ACSB
biological_	behavior	28	0.1	GO:00076	AHR1B;ALDH1A3;ARR3;ASCL1;ASIP;CASR;CHRNA3;DAB1;DCDC2;DNM1;DPP4;ESPN;FABP4;GHF
biological_	metabolic	156	0.5571	GO:00081	A2ML3;ABCB1LA;ABHD3;ABHD6;ACAA1;ACACA;ACACB;ACAD11;ACOD1;ACOX1;ACSBG2;ACSL
biological_	cellular pr	246	0.8785	GO:00099	A2ML3;ABCA12;ABCB1LA;ABHD3;ABHD6;ACAA1;ACACA;ACACB;ACAD11;ACKR2;ACOD1;ACO
molecular_	antioxidar	2	0.0071	GO:00162	GSTK1;GSTO2
biological_	reproduct	44	0.1571	GO:00224	A2ML3;ABCB1LA;ACOD1;ACOX1;ADAM15;ADGRG2;AHR1B;ASCL1;BUB1;CBX2;CDK6;CDT1;CYP
biological_	biological	10	0.0357	GO:00226	ADD2;AMIGO2;ANGPTL3;DAB1;EPHA1;ITGB6;LOC121107687;PDIA6;SPON2;TENM2
biological_	signaling	86	0.3071	GO:00230	ACKR2;ACSL1;ADGRF5;ADGRG2;AHR1B;ANGPTL3;ARHGEF38;ARR3;ASCL1;ASIP;ATP6V0D2;BHL
biological_	multicellul	145	0.5178	GO:00325	A2ML3;ABCB1LA;ACACA;ACACB;ACKR2;ACOD1;ACOX1;ADAM15;ADD2;ADGRF5;ADGRG2;AHR
biological_	developm	128	0.4571	GO:00325	A2ML3;ABCB1LA;ACACA;ACACB;ACKR2;ACOD1;ACOX1;ADAM15;ADD2;ADGRF5;ADGRG2;AHR
cellular_cc	protein-cc	69	0.2464	GO:00329	ABHD6;ADD2;AHR1B;ASCL1;ATP6V0D2;BUB1;CACNA1H;CARD10;CBX2;CDC45;CDCA9;CDK6;CD
molecular_	cargo rece	1	0.0035	GO:00380	ACKR2
biological_	growth	10	0.0357	GO:00400	ALPI;ASCL1;CDT1;DHCR7;GJA1;LIPC;MUSTN1;STMN1;TENM2;WNT11
biological_	locomotio	29	0.1035	GO:00400	ACKR2;ADD2;ASCL1;DAB1;DCDC2;DPP4;ENPEP;FDPS;FGF19;GJA1;GRIN2C;IFT80;MAP3K15;MCN
biological_	pigmentat	5	0.0178	GO:00434	ASIP;DCT;GSTO2;LOC121107687;SHROOM3
biological_	interspecie	27	0.0964	GO:00444	ABCB1LA;ACOD1;AVD;CA5A;CDK6;CDT1;CYP3A5;DPP4;DUOX1;ECI2;FGF19;GJA1;GPAM;IL22RA
biological_	rhythmic p	7	0.025	GO:00485	A2ML3;LIPC;PANK1;PPARGC1A;PROK2;SLC4A1;SRD5A1
biological_	response t	164	0.5857	GO:00508	A2ML3;ABCB1LA;ACAA1;ACACA;ACACB;ACKR2;ACOD1;ACSBG2;ACSL1;ADGRF5;ADGRG2;AGP
biological_	localizatio	105	0.375	GO:00511	A2ML3;ABCA12;ABCB1LA;ACAA1;ACACA;ACACB;ACKR2;ACOX1;ACSL1;ADD2;AGPAT2;ANGPTL
biological_	intraspecie	1	0.0035	GO:00517	GRID1
biological_	multi-orga	50	0.1785	GO:00517	A2ML3;ABCB1LA;ACOD1;ACOX1;ADAM15;ADGRG2;AHR1B;AVD;BUB1;CA5A;CDK6;CDT1;CYP3
molecular_	molecular	17	0.0607	GO:00600	ACKR2;ADGRF5;ADGRG2;CASR;CHRNA3;EPHA1;GPR27;GRIN2C;IL1RL1;IL22RA1;LOC112531135
biological_	biological	201	0.7178	GO:00650	A2ML3;ABCA12;ABCB1LA;ABHD6;ACACA;ACACB;ACKR2;ACOD1;ACOX1;ACSBG2;ACSL1;ADAM
biological_	detoxificat	2	0.0071	GO:00987	GSTK1;GSTO2
molecular_	molecular	24	0.0857	GO:00987	A2ML3;ANGPTL3;ARHGEF38;CDKN2B;FGF19;GHRL;GPC3;GRIN2C;LOC107050717;MDK;PCOLCE
biological_	biominera	2	0.0071	GO:01101	GPC3;WNT11
cellular_cc	cellular an	271	0.9678	GO:01101	A2ML3;ABCA12;ABCB1LA;ABHD3;ABHD6;ACAA1;ACACA;ACACB;ACAD11;ACKR2;ACOD1;ACO
molecular_	molecular	2	0.0071	GO:01401	HBBA;HBE1
molecular_	transcripti	16	0.0571	GO:01401	AHR1B;ASCL1;BHLHA15;CREB3L3;CREG1;ELF3;KIAA1958;LOC112530071;MED13;MYCL;PPARGC

4B7;DHCR24;FDPS;GHRL;GJA1;HORMAD2;HYOU1;KCNQ1;LOC100858984;MAP3K15;MCM4;MCM5;MCM9;MEI1;MKI67;PANK1;PROK2;PTTG2;RBPJ;SC
DACT2;DPP4;EPB42;FASN;GADD45G;GJA1;GPAM;GPC3;HBA1;HSP90B1;IL1RL1;LOC112530071;LOC121106502;LOC121106942;LOC121106943;LOC76
L1;ADAM15;AGPAT2;ALDH1A3;ALG12;ALPI;ALPK2;ATIC;ATP6V0D2;BUB1;CA5A;CASR;CDC45;CDK6;CDKN2B;CES1L2;CHKA;CYP2C23a;CYP2C23b;CYP

HR1B;ALDH1A3;ALPI;ANGPTL3;ANK1;APOF;ARHGEF38;ARR3;ASCL1;ASIP;ATIC;AVD;BHLHA15;CACNA1H;CARD10;CASR;CBX2;CDC45;CDK6;CDKN2B;
G2;ACSL1;ADD2;ADGRF5;ADGRG2;AGPAT2;AHR1B;ALDH1A3;ALG12;ALPI;ALPK2;ANGPTL3;ANK1;ARR3;ASCL1;ATIC;ATP6V0D2;BHLHA15;BUB1;CA5A;

1;ADAM15;ADGRF5;AGPAT2;ALDH1A3;ALG12;ALPI;ALPK2;ANGPTL3;ANK1;APOF;ASCL1;ASIP;ATIC;BHLHA15;BUB1;CACNA1H;CASR;CDC45;CDK6;CD
1;ACSBG2;ACSL1;ADAM15;ADD2;ADGRF5;ADGRG2;AGPAT2;AHR1B;ALDH1A3;ALG12;ALPI;ALPK2;AMIGO2;ANGPTL3;ANK1;APOF;ARHGEF38;ARR3;A

4B7;DHCR24;FDPS;GHRL;GJA1;HORMAD2;KCNQ1;LOC100858984;MAP3K15;MCM4;MCM5;MCM9;MEI1;MKI67;PANK1;PROK2;PTTG2;RBPJ;SCD;SMC4

HA15;CACNA1H;CARD10;CASR;CDK6;CDKN2B;CHRNA3;CREB3L3;DAB1;DACT2;DHCR24;DLGAP5;DNAJB11;DNM1;DPYSL2;DUOX1;ENPEP;EPHA1;FAS
1B;ALDH1A3;ALPI;ANGPTL3;ANK1;ARR3;ASCL1;ASIP;ATIC;ATP6V0D2;BUB1;C8orf22;CACNA1H;CASR;CBX2;CDK6;CDKN2B;CDT1;CES1L2;CETP;CHRNA
1B;ALDH1A3;ALPI;ANGPTL3;ANK1;ARR3;ASCL1;ATIC;ATP6V0D2;BHLHA15;C8orf22;CACNA1H;CASR;CBX2;CDK6;CDKN2B;CDT1;CES1L2;CHRNA3;CRE
KN2B;CENPN;CETP;CHRNA3;CREG1;DNAJB11;DNM1;DPYSL2;ESPN;GJA1;GRIN2C;HADHB;HBA1;HBBA;HBE1;HSP90B1;HSPA5;HYOU1;IFT80;ITGB6;KC

T2;AHR1B;ALDH1A3;ANGPTL3;ARHGEF38;ARR3;ASCL1;ASIP;ATIC;ATP6V0D2;AVD;BHLHA15;CA5A;CACNA1H;CARD10;CASR;CDC45;CDK6;CDKN2B;
3;ANK1;APOF;ARR3;ASCL1;ASIP;ATP6V0D2;BHLHA15;CA5A;CACNA1H;CASR;CDT1;CES1L2;CETP;CHKA;CHRNA3;CREG1;DAB1;DCDC2;DHCR24;DLGA

5;DPP4;DUOX1;ECI2;FDPS;FGF19;GHRL;GJA1;GPAM;GRID1;IL22RA1;LOC100858984;LOC121107687;LOC768786;MAP3K15;MCM9;MDK;MEI1;PANK1;

15;ADD2;ADGRF5;ADGRG2;AGPAT2;AHR1B;ALDH1A3;AMIGO2;ANGPTL3;ANK1;ARHGEF38;ARR3;ASCL1;ASIP;ATP6V0D2;BHLHA15;BUB1;CACNA1H;

1;ACSBG2;ACSL1;ADAM15;ADAMTSL5;ADD2;ADGRF5;ADGRG2;AGPAT2;AHR1B;ALDH1A3;ALG12;ALPI;ALPK2;AMIGO2;ANGPTL3;ANK1;APOF;ARR3;

3A5;CYP4B7;CYP7A1;CYP8B1;DCT;DHCR24;DHCR7;DLGAP5;DNM1;DPP4;DPYSL2;DUOX1;DUSP4;ECI2;EHHADH;ELOVL2;ENPEP;EPHA1;FAM20CL;FASN

CDT1;CETP;CHKA;CHRNA3;COQ10B;CREB3L3;CREG1;CSRP1;CYP3A5;CYP4B7;CYP8B1;DAB1;DACT2;DCDC2;DCT;DHCR24;DHCR7;DNAJB11;DNAJC12;
CACNA1H;CARD10;CASR;CBX2;CCNB3;CDC45;CDCA7;CDCA9;CDK6;CDKN2B;CDT1;CENPN;CES1L2;CHKA;COQ10B;CPED1;CREB3L3;CREG1;CRELD2;C

KN2B;CDT1;CES1L2;CETP;CHKA;COQ10B;CREB3L3;CYP2C23a;CYP2C23b;CYP3A5;CYP4B7;CYP7A1;CYP8B1;DCT;DHCR24;DHCR7;DLGAP5;DNAJB11;DN
SCL1;ASIP;ATIC;ATP6V0D2;BHLHA15;BUB1;C8orf22;CACNA1H;CARD10;CASR;CDC45;CDCA9;CDK6;CDKN2B;CDT1;CENPN;CES1L2;CETP;CHKA;CHRNA

LG;FGF19;G0S2;GHRL;GJA1;GPR27;GRIN2C;GSTO2;HSP90B1;HSPA5;HYOU1;IFT80;IL1RL1;IL22RA1;IQUB;KCNQ1;LOC112530071;LOC112531135;LOC1
A3;CREG1;CRTAC1;CSRP1;CYP4B7;DAB1;DACT2;DCDC2;DCT;DHCR24;DHCR7;DNAJB11;DNM1;DPP4;DPYSL2;DUOX1;DUSP4;ELF3;ENPEP;EPB42;EPHA1
G1;CRTAC1;CSRP1;CYP3A5;CYP4B7;DAB1;DACT2;DCDC2;DCT;DHCR24;DHCR7;DNAJB11;DPYSL2;DUOX1;DUSP4;ELF3;ENPEP;EPB42;EPHA1;FABP4;FAS
NQ1;KLHL24;LAMB3;LOC100858984;LOC121111295;MAP3K15;MCM3;MCM4;MCM5;MCM9;MED13;MIB2;MIS12;NPTXR;PANK1;PCP4;PDHA2;PDIA6;P

CDT1;CES1L2;CHKA;CHRNA3;CPT1A;CREB3L3;CREG1;CYP2C23a;CYP2C23b;CYP3A5;CYP4B7;CYP7A1;DAB1;DACT2;DCDC2;DHCR24;DLGAP5;DNAJB11
P5;DNM1;DPP4;DPYSL2;ECI2;EHHADH;ENPEP;FABP2;FABP4;FASLG;FDPS;FGF19;GHRL;GJA1;GPAM;GRIN2C;GSTK1;HBA1;HBBA;HBE1;HMGCL;HSP90B1

CARD10;CASR;CBX2;CDC45;CDCA7;CDK6;CDKN2B;CDT1;CES1L2;CETP;CHKA;CHRNA3;CREB3L3;CREG1;CRTAC1;CYP3A5;CYP4B7;CYP7A1;DAB1;DACT2

ASCL1;ASIP;ATIC;ATP6V0D2;AVD;BHLHA15;BUB1;CA5A;CACNA1H;CARD10;CASR;CBX2;CCNB3;CDC45;CDCA3;CDCA7;CDCA9;CDK6;CDKN2B;CDT1;CE

N;FBP2;FDFT1;FDPS;FGF19;FPGS;GALE;GLDC;GPAM;GPAT3;GSTK1;GSTO2;HADHB;HKDC1;HMGCL;HOGA1;HS6ST3;HSPA5;LIPC;LOC121111295;LOC76

DNM1;DPP4;DPYSL2;DUOX1;ECI2;EHHADH;ELF3;ENPEP;EPB42;EPHA1;ESPN;FABP2;FABP4;FAM110C;FAM20CL;FASLG;FASN;FBP2;FGF19;GADD45G;G
SRP1;CYP2C23a;CYP2C23b;CYP3A5;CYP4B7;CYP7A1;CYP8B1;DAB1;DACT2;DCDC2;DCT;DHCR24;DHCR7;DLEC1;DLGAP5;DNAJB11;DNAJC12;DNM1;D

NM1;DPP4;DPYSL2;DUOX1;DUSP4;ECI2;EHHADH;ELF3;ELOVL2;ENPEP;EPB42;EPHA1;FABP2;FABP4;FAM20CL;FASN;FBP2;FDFT1;FDPS;FGF19;FPGS;GAD
A3;COQ10B;CREB3L3;CREG1;CRTAC1;CSRP1;CYP2C23a;CYP2C23b;CYP3A5;CYP4B7;CYP7A1;CYP8B1;DAB1;DACT2;DCDC2;DCT;DHCR24;DHCR7;DLGA

21106502;LOC121106942;LOC121106943;LPL;MAP3K15;MAP3K7CL;MDK;MED13;NPTX2;NSMF;PCP4;PDGFRL;PDIA6;PODN;PPARGC1A;PRODH;PROK
;FABP2;FABP4;FASN;FDPS;FGF19;GADD45G;GHRL;GJA1;GPAM;GPC3;GRIN2C;HADHB;HBA1;HBBA;HBE1;HMGCL;HS6ST3;HSP90B1;HSPA5;HYOU1;IFT
N;FGF19;GADD45G;GHRL;GJA1;GPC3;GRIN2C;GSTK1;HADHB;HBA1;HMGCL;HS6ST3;HSP90B1;HSPA5;IFT80;KCNQ1;LAMB3;LIPC;LOC100858984;LOC
PPARGC1A;RAD9B;RAPGEF4;RBPJ;RRM2;SDF2L1;SLC51AL;SMC4;SOX8;SPC25;SPTB;STMN1;SUN2;TCIRG1;TEN1;TOP2A;TRPM1;UBE2U;VPS29L

;DNM1;DPP4;DPYSL2;DUOX1;ECI2;ELF3;EPHA1;FABP4;FASLG;FASN;FGF19;G0S2;GADD45G;GHRL;GJA1;GLDC;GPAM;GPR27;GRIN2C;GSTK1;GSTO2;HB
L;HSPA5;HYOU1;IFT80;ITGB6;KCNQ1;LIPC;LOC100858984;LOC121106502;LOC121106942;LOC121106943;LOC121107687;LRG1;MDK;MED13;MEI1;MI

2;DCDC2;DCT;DHCR24;DHCR7;DLEC1;DLGAP5;DNAJB11;DNM1;DPP4;DPYSL2;DUOX1;DUSP4;ELF3;ENPEP;EPB42;EPHA1;ESPN;FABP4;FAM110C;FASL

ENPN;CES1L2;CETP;CHKA;CHRNA3;COQ10B;CPED1;CREB3L3;CREG1;CRELD2;CRTAC1;CSRP1;CYP2C23a;CYP2C23b;CYP3A5;CYP4B7;CYP7A1;CYP8B1;

8786;LPL;MAN1B1;MAP3K15;MAP3K7CL;ME1;MIB2;MICAL2;MSMO1;OLAH;PANK1;PDGFRL;PDHA2;PDIA2;PDIA6;PNPLA3;PPTC7;PRODH;PTDSS1;RAD

ALE;GHRL;GJA1;GLDC;GRIN2C;GSTK1;GSTO2;HADHB;HBA1;HBBA;HBE1;HKDC1;HMGCL;HOGA1;HORMAD2;HS6ST3;HSP90B1;HSPA5;HYOU1;IL1RL1;IL
PP4;DPYSL2;DUSP4;ECI2;EHHADH;ELF3;ELOVL2;ENPEP;EPB42;ESPN;FABP2;FABP4;FAM110C;FAM20CL;FASLG;FASN;FBP2;FDFT1;FDPS;FPGS;G0S2;GAD

D45G;GALE;GHRL;GLDC;GPAM;GPAT3;GPC3;GRIN2C;GSTK1;GSTO2;HADHB;HBA1;HKDC1;HMGCL;HOGA1;HS6ST3;HSP90B1;HSPA5;ITGB6;KCNQ1;KIA
P5;DNAJB11;DNAJC12;DNM1;DPP4;DPYSL2;DUOX1;DUSP4;ECI2;EHHADH;ELF3;ELOVL2;ENPEP;EPB42;EPHA1;ESPN;FABP2;FABP4;FAM20CL;FASLG;FA

2;RAPGEF4;RBPJ;RCAN2;RDH11;RGS8;RND2;SAA;SGK1;SNCG;SOCS3;SOX8;SPTB;STC2;SUCO;TCIRG1;TENM2;TLR5;TPP1;TRPM1;TSKU;VSNL1;WNT11
30;ITGB6;KCNQ1;LAMB3;LIPC;LOC107054133;LOC121107687;LOC768786;LPL;MAP3K15;MCM4;MCM9;MDK;MED13;MEI1;MICAL2;MKI67;MLF1;MUST
107054133;LOC121107687;MAP3K15;MCM4;MDK;MED13;MEI1;MIB2;MICAL2;MKI67;MLF1;MUSTN1;MYCL;NSMF;OTX5;PANK1;PCP4;PDCD1;PPARGC

A1;HBBA;HBE1;HMGCL;HSP90B1;HSPA5;HYOU1;IFT80;IL1RL1;IL22RA1;IQUB;ITGB6;KCNQ1;KIAA0101;LIPC;LOC112530071;LOC112531135;LOC121106
S12;PDIA2;PDIA6;PGR2/3;RAPGEF4;RBP;RBPJ;RHAG;SAA;SGK1;SHROOM3;SLC16A1;SLC16A5;SLC25A25;SLC26A9;SLC4A1;SLC51AL;SNCG;SOX8;SPIRE2

;FASN;FDFT1;FDPS;FGF19;G0S2;GADD45G;GHRL;GJA1;GLDC;GPAM;GPAT3;GPC3;GPR27;GRIN2C;GSTO2;HBA1;HBBA;HBE1;HKDC1;HORMAD2;HS6ST

AB1;DACT2;DCDC2;DCT;DHCR24;DHCR7;DLEC1;DLGAP5;DNAJB11;DNAJC12;DNM1;DPP4;DPYSL2;DUOX1;DUSP4;ECI2;EHHADH;ELF3;ELOVL2;ENPEP

)9B;RBPJ;RDH11;RETSAT;RND2;RRM2;SCD;SDF2L1;SGK1;SGPP2;SMC4;SQLE;SRD5A1;SRR;SULT1B;TCIRG1;TK1;TOP2A;TOR3A;TPP1;UBE2U;UGP2;UNG;U

.22RA1;ITGB6;KCNQ1;KIAA0101;KIAA1958;LAMB3;LIPC;LOC100858984;LOC107050717;LOC112530071;LOC112531135;LOC121106502;LOC12110694
)D45G;GALE;GHRL;GJA1;GLDC;GPAM;GPAT3;GPC3;GSTK1;GSTO2;HADHB;HBA1;HBBA;HBE1;HKDC1;HMGCL;HN1;HOGA1;HORMAD2;HSP90B1;HSPA

AA0101;KLHL24;LIPC;LOC107050717;LOC121111295;LOC768786;LPL;MAN1B1;MAP3K15;MAP3K7CL;MCM3;MCM4;MCM5;MCM9;ME1;MED13;MIB2;N
SN;FBP2;FDFT1;FDPS;FGF19;FPGS;G0S2;GADD45G;GALE;GHRL;GJA1;GLDC;GPAM;GPAT3;GPC3;GPR27;GRIN2C;GSTK1;GSTO2;HADHB;HBA1;HBBA;HBI

N1;MYCL;NPTX2;NSMF;OTX5;PANK1;PCP4;PDCD1;PDIA6;PNPLA3;PPARGC1A;PROK2;PSD3;PTTG2;RAPGEF4;RBPJ;RCAN2;RCN1;RDH11;RGS8;RGS9BP
1A;PROK2;PSD3;PTTG2;RAPGEF4;RBPJ;RCN1;RGS8;RHAG;RND2;SCD;SGK1;SHROOM3;SMC4;SMTNL1;SMYD1;SNCG;SOCS3;SOX7;SOX8;SPIRE2;SPON2

5502;LOC121106942;LOC121106943;LOC121107687;LOC121111295;LOC768786;LPL;LRG1;MAN1B1;MAP3K15;MAP3K7CL;MCM9;MDK;ME1;MED13;M

3;HSP90B1;HSPA5;HYOU1;IFT80;IL1RL1;IL22RA1;IQUB;KANK4;KCNQ1;KIAA0101;KIAA1958;LIPC;LOC100858984;LOC107050717;LOC107054133;LOC1

P;EPB42;EPHA1;ESPN;FABP2;FABP4;FAM110C;FAM20CL;FASLG;FASN;FBP2;FDFT1;FDPS;FGF19;FPGS;G0S2;GADD45G;GALE;GHRL;GJA1;GLDC;GPAM;GI

2;LOC121106943;LOC121107687;LOC121111295;LOC768786;LPL;MAN1B1;MAP3K15;MCM3;MCM4;MDK;ME1;MED13;MIB2;MICAL2;MKI67;MLF1;MY
5;HYOU1;IFT80;IQUB;ITGB6;KANK4;KCNQ1;KIAA0101;KIAA1958;KLHL24;LIPC;LOC100858984;LOC107050717;LOC112530071;LOC121106502;LOC121

MICAL2;MLF1;MSMO1;OLAH;PANK1;PDGFRL;PDHA2;PDIA2;PDIA6;PGR2/3;PNPLA3;PPARGC1A;PPTC7;PRODH;PTDSS1;PTTG2;RAD9B;RBPJ;RCN1;RDH
E1;HKDC1;HMGCL;HOGA1;HORMAD2;HS6ST3;HSP90B1;HSPA5;HYOU1;IFT80;IL1RL1;IL22RA1;IQUB;ITGB6;KCNQ1;KIAA0101;KLHL24;LAMB3;LIPC;LOC

;RHAG;RND2;SAA;SCD;SGK1;SHROOM3;SMC4;SMTNL1;SMYD1;SNCG;SOCS3;SOX7;SOX8;SPINT4;SPIRE2;SPON2;SPTB;SRD5A1;SRR;STC2;STMN1;SUN

IUSTN1;NSMF;PANK1;PCOLCE2;PCP4;PDCD1;PDGFRL;PDIA2;PDIA6;PGR2/3;PNPLA3;PODN;PPARGC1A;PRODH;PROK2;PSD3;RAD9B;RAPGEF4;RBP;RB

12530071;LOC112531135;LOC121106502;LOC121106942;LOC121106943;LOC121107687;LOC121111295;LOC768786;LPL;MAP3K15;MAP3K7CL;MCM

PAT3;GPC3;GPR27;GRID1;GRIN2C;GSTK1;GSTO2;HADHB;HBA1;HBAD;HBBA;HBE1;HKDC1;HMGCL;HN1;HOGA1;HORMAD2;HSP90B1;HSPA5;HYOU1;I

CL;NPTXR;NSMF;PCOLCE2;PCP4;PDIA6;PGR2/3;PNPLA3;PODN;PPARGC1A;PRODH;PROK2;PSD3;PTTG2;RAD9B;RAPGEF4;RBP;RBPJ;RCN1;RGS8;RHAG
106942;LOC121106943;LOC121107687;LOC121111295;LOC768786;LRG1;MAN1B1;MAP3K15;MAP3K7CL;MCM3;MCM4;MCM5;MCM9;MDK;ME1;ME

11;RETSAT;RMI2;RRM2;SAA;SCD;SDF2L1;SGK1;SGPP2;SLC16A1;SMC4;SMTNL1;SOCS3;SPTB;SQLE;SRD5A1;SRR;STC2;SULT1B;TCIRG1;TCN2;TEN1;TK1;
100858984;LOC107050717;LOC107054133;LOC112530071;LOC112531135;LOC121106502;LOC121106942;LOC121106943;LOC121107687;LOC1211

PJ;RCAN2;RDH11;RGS8;RGS9BP;RHAG;RND2;SAA;SCD;SDF2L1;SGK1;SHROOM3;SLC16A1;SLC25A25;SLC4A1;SMC4;SMTNL1;SNCG;SOCS3;SOX8;SPIN

4;MCM5;MDK;ME1;MED13;MIB2;MICAL2;MKI67;MLF1;MUSTN1;MYCL;NPTXR;NSMF;OTX5;PANK1;PCOLCE2;PCP4;PDCD1;PDGFRL;PDIA2;PDIA6;PGR

T80;IL1RL1;IL22RA1;IQUB;ITGB6;KANK4;KCNQ1;KIAA0101;KIAA1958;KLHL24;LAMB3;LIPC;LOC100858984;LOC107050717;LOC107054133;LOC11253

[illegible]13;MIB2;MICAL2;MIS12;MKI67;MLF1;MSMO1;MUSTN1;MYCL;NPTX2;NPTXR;NSMF;OLAH;OTX5;PANK1;PCP4;PDHA2;PDIA2;PDIA6;
