# Supplementary material for: Comparative analysis of liver transcriptome reveals adaptive responses to hypoxia environmental condition in Tibetan chicken
Source: Anim Biosci. 2023 Aug 23;37(1):28–38. doi: 10.5713/ab.23.0126 (PMC10766467; doi:10.5713/ab.23.0126)
Supplement: Supplementary file 8 [file ab-23-0126-Supplementary-Table-8.pdf]

Table S8

layer1	layer2	layer3	ID	Input.num	Backgrour	pvalue	FDR	Input	Hyperlink
5. Organis	5.2 Endoc	PPAR sign	ko03320	14	69	2.16E-07	3.97E-05	ACAA1|AC	http://www.genome.jp/kegg-bin/show_pathway?ko0
1. Metabo	1.3 Lipid n	Fatty acid	ko00071	9	36	5.26E-06	0.000484	ACAA1|AC	http://www.genome.jp/kegg-bin/show_pathway?ko0
1. Metabo	1.0 Global	Fatty acid	ko01212	11	59	1.03E-05	0.00063	ACAA1|AC	http://www.genome.jp/kegg-bin/show_pathway?ko0
1. Metabo	1.3 Lipid n	Fatty acid	ko00061	6	20	6.72E-05	0.00309	ACACA|AC	http://www.genome.jp/kegg-bin/show_pathway?ko0
1. Metabo	1.0 Global	Biosynthes	ko01130	18	204	0.000691	0.025433	ACAA1|AC	http://www.genome.jp/kegg-bin/show_pathway?ko0
1. Metabo	1.3 Lipid n	Steroid bio	ko00100	5	20	0.000709	0.021743	DHCR24|D	http://www.genome.jp/kegg-bin/show_pathway?ko0
1. Metabo	1.0 Global	Biosynthes	ko01110	29	406	0.000714	0.018761	ACAA1|AC	http://www.genome.jp/kegg-bin/show_pathway?ko0
1. Metabo	1.9 Metab	Sesquiterp	ko00909	2	2	0.001405	0.032325	FDFT1|SQI	http://www.genome.jp/kegg-bin/show_pathway?ko0
1. Metabo	1.1 Carbol	Pyruvate r	ko00620	6	36	0.002027	0.041444	ACACA|AC	http://www.genome.jp/kegg-bin/show_pathway?ko0
1. Metabo	1.5 Amino	Valine, leu	ko00280	7	49	0.002206	0.040581	AACS|ACA	http://www.genome.jp/kegg-bin/show_pathway?ko0
1. Metabo	1.1 Carbol	Propanoat	ko00640	5	31	0.005531	0.092511	ACACA|AC	http://www.genome.jp/kegg-bin/show_pathway?ko0
1. Metabo	1.3 Lipid n	Glycerolip	ko00561	7	61	0.00762	0.116846	AGPAT2|A	http://www.genome.jp/kegg-bin/show_pathway?ko0
4. Cellular	4.2 Cell gr	Cell cycle	ko04110	10	118	0.013303	0.188285	BUB1|CCN	http://www.genome.jp/kegg-bin/show_pathway?ko0
5. Organis	5.2 Endoc	Glucagon	ko04922	7	78	0.02689	0.353412	ACACA|AC	http://www.genome.jp/kegg-bin/show_pathway?ko0
1. Metabo	1.3 Lipid n	Biosynthes	ko01040	4	31	0.02765	0.339179	ACAA1|AC	http://www.genome.jp/kegg-bin/show_pathway?ko0
4. Cellular	4.1 Transp	Peroxisom	ko04146	7	79	0.028588	0.328762	ACAA1|AC	http://www.genome.jp/kegg-bin/show_pathway?ko0
5. Organis	5.2 Endoc	Adipocyto	ko04920	6	66	0.036995	0.400421	ACACB|AC	http://www.genome.jp/kegg-bin/show_pathway?ko0
1. Metabo	1.0 Global	Carbon m	ko01200	8	106	0.045835	0.468532	ACOX1|AC	http://www.genome.jp/kegg-bin/show_pathway?ko0
1. Metabo	1.0 Global	Metabolic	ko01100	64	1385	0.046764	0.45287	AACS|ACA	http://www.genome.jp/kegg-bin/show_pathway?ko0
5. Organis	5.5 Excret	Collecting	ko04966	3	23	0.053308	0.490438	ATP6V0D2	http://www.genome.jp/kegg-bin/show_pathway?ko0
1. Metabo	1.1 Carbol	Butanoate	ko00650	3	23	0.053308	0.467083	AACS|EHH	http://www.genome.jp/kegg-bin/show_pathway?ko0
5. Organis	5.4 Digest	Cholester	ko04979	5	55	0.054692	0.457425	ANGPTL3|	http://www.genome.jp/kegg-bin/show_pathway?ko0
1. Metabo	1.1 Carbol	Glycolysis	ko00010	5	56	0.058287	0.466295	ACSS1B|A	http://www.genome.jp/kegg-bin/show_pathway?ko0
1. Metabo	1.11 Xeno	Drug meta	ko00983	5	56	0.058287	0.446866	CES1L2|GS	http://www.genome.jp/kegg-bin/show_pathway?ko0
3. Environ	3.2 Signal	AMPK sign	ko04152	8	112	0.059748	0.439747	ACACA|AC	http://www.genome.jp/kegg-bin/show_pathway?ko0
1. Metabo	1.8 Metab	Retinol me	ko00830	4	40	0.061877	0.437897	CYP3A5|L(	http://www.genome.jp/kegg-bin/show_pathway?ko0
1. Metabo	1.11 Xeno	Toluene d	ko00623	1	2	0.073635	0.501808	CMBL	http://www.genome.jp/kegg-bin/show_pathway?ko0
1. Metabo	1.11 Xeno	Fluoroben	ko00364	1	2	0.073635	0.483887	CMBL	http://www.genome.jp/kegg-bin/show_pathway?ko0
1. Metabo	1.1 Carbol	C5-Branch	ko00660	1	2	0.073635	0.467201	ACOD1	http://www.genome.jp/kegg-bin/show_pathway?ko0
1. Metabo	1.11 Xeno	Chlorocyc	ko00361	1	2	0.073635	0.451628	CMBL	http://www.genome.jp/kegg-bin/show_pathway?ko0
1. Metabo	1.6 Metab	beta-Alan	ko00410	3	28	0.0861	0.511047	ACOX1|AL	http://www.genome.jp/kegg-bin/show_pathway?ko0
1. Metabo	1.1 Carbol	Amino sug	ko00520	4	46	0.093054	0.53506	GALE|HKD	http://www.genome.jp/kegg-bin/show_pathway?ko0
1. Metabo	1.3 Lipid n	Primary bi	ko00120	2	15	0.106964	0.596406	CYP7A1|C	http://www.genome.jp/kegg-bin/show_pathway?ko0
1. Metabo	1.1 Carbol	Ascorbate	ko00053	2	15	0.106964	0.578865	ALDH1A3|	http://www.genome.jp/kegg-bin/show_pathway?ko0
1. Metabo	1.1 Carbol	Glyoxylate	ko00630	3	31	0.108985	0.572948	ACSS1B|G	http://www.genome.jp/kegg-bin/show_pathway?ko0
2. Genetic	2.4 Replica	DNA repli	ko03030	3	32	0.117084	0.598428	MCM3|MC	http://www.genome.jp/kegg-bin/show_pathway?ko0

1. Metabo	1.11 Xeno	Drug meta	ko00982	3	32	0.117084	0.582255	GSTK1|GS	http://www.genome.jp/kegg-bin/show_pathway?ko0
1. Metabo	1.11 Xeno	Metabolis	ko00980	3	34	0.133921	0.64846	GSTK1|GS	http://www.genome.jp/kegg-bin/show_pathway?ko0
1. Metabo	1.9 Metab	Geraniol c	ko00281	1	4	0.141856	0.669271	HMGCL	http://www.genome.jp/kegg-bin/show_pathway?ko0
1. Metabo	1.11 Xeno	Caprolacta	ko00930	1	4	0.141856	0.652539	EHHADH	http://www.genome.jp/kegg-bin/show_pathway?ko0
1. Metabo	1.5 Amino	Phenylalar	ko00400	1	4	0.141856	0.636624	IL4I1	http://www.genome.jp/kegg-bin/show_pathway?ko0
1. Metabo	1.9 Metab	Limonene	ko00903	1	4	0.141856	0.621466	ALDH1A3	http://www.genome.jp/kegg-bin/show_pathway?ko0
1. Metabo	1.1 Carbol	Galactose	ko00052	3	36	0.151534	0.648425	GALE|HKD	http://www.genome.jp/kegg-bin/show_pathway?ko0
1. Metabo	1.3 Lipid n	Glyceroph	ko00564	6	98	0.162391	0.679089	AGPAT2|C	http://www.genome.jp/kegg-bin/show_pathway?ko0
5. Organis	5.2 Endoc	Insulin sig	ko04910	7	120	0.164776	0.673753	ACACA|A(	http://www.genome.jp/kegg-bin/show_pathway?ko0
5. Organis	5.4 Digest	Mineral ab	ko04978	3	38	0.169831	0.679323	MT3|MT4|	http://www.genome.jp/kegg-bin/show_pathway?ko0
1. Metabo	1.9 Metab	Insect hor	ko00981	1	5	0.174062	0.681436	ALDH1A3	http://www.genome.jp/kegg-bin/show_pathway?ko0
1. Metabo	1.11 Xeno	Aminober	ko00627	1	5	0.174062	0.667239	EHHADH	http://www.genome.jp/kegg-bin/show_pathway?ko0
1. Metabo	1.3 Lipid n	Steroid hc	ko00140	3	39	0.179207	0.672941	CYP3A5|C	http://www.genome.jp/kegg-bin/show_pathway?ko0
1. Metabo	1.1 Carbol	Pentose ai	ko00040	2	21	0.185289	0.681863	LOC12111	http://www.genome.jp/kegg-bin/show_pathway?ko0
1. Metabo	1.11 Xeno	Chloroalka	ko00625	1	6	0.205062	0.739831	ALDH1A3	http://www.genome.jp/kegg-bin/show_pathway?ko0
1. Metabo	1.5 Amino	Tryptopha	ko00380	3	42	0.208114	0.736403	ALDH1A3|	http://www.genome.jp/kegg-bin/show_pathway?ko0
1. Metabo	1.6 Metab	Glutathior	ko00480	3	45	0.237961	0.826129	GSTK1|GS	http://www.genome.jp/kegg-bin/show_pathway?ko0
1. Metabo	1.5 Amino	Arginine a	ko00330	3	46	0.248071	0.845279	ALDH1A3|	http://www.genome.jp/kegg-bin/show_pathway?ko0
4. Cellular	4.2 Cell gr	Cellular se	ko04218	8	160	0.253045	0.846551	CCNB3|CD	http://www.genome.jp/kegg-bin/show_pathway?ko0
1. Metabo	1.2 Energy	Methane r	ko00680	2	26	0.254999	0.837853	ACSS1B|FE	http://www.genome.jp/kegg-bin/show_pathway?ko0
1. Metabo	1.3 Lipid n	Fatty acid	ko00062	2	27	0.26908	0.86861	ELOVL2|H.	http://www.genome.jp/kegg-bin/show_pathway?ko0
1. Metabo	1.8 Metab	Folate bio:	ko00790	2	27	0.26908	0.853634	ALPI|FPGS	http://www.genome.jp/kegg-bin/show_pathway?ko0
5. Organis	5.2 Endoc	Thyroid hc	ko04918	3	50	0.289055	0.901459	CREB3L3|H	http://www.genome.jp/kegg-bin/show_pathway?ko0
4. Cellular	4.4 Cellula	Quorum s	ko02024	1	9	0.291263	0.893205	ACSL1	http://www.genome.jp/kegg-bin/show_pathway?ko0
1. Metabo	1.3 Lipid n	alpha-Lin	ko00592	2	29	0.297206	0.896489	ACAA1|A(	http://www.genome.jp/kegg-bin/show_pathway?ko0
1. Metabo	1.10 Biosy	Isoquinoli	ko00950	1	10	0.31787	0.943356	IL4I1	http://www.genome.jp/kegg-bin/show_pathway?ko0
1. Metabo	1.3 Lipid n	Synthesis	ko00072	1	10	0.31787	0.928382	HMGCL	http://www.genome.jp/kegg-bin/show_pathway?ko0
1. Metabo	1.5 Amino	Lysine deg	ko00310	3	53	0.320113	0.920326	ALDH1A3|	http://www.genome.jp/kegg-bin/show_pathway?ko0
1. Metabo	1.1 Carbol	Starch anc	ko00500	2	31	0.325159	0.92045	HKDC1|U(	http://www.genome.jp/kegg-bin/show_pathway?ko0
1. Metabo	1.5 Amino	Tyrosine n	ko00350	2	33	0.352813	0.9836	DCT|IL4I1	http://www.genome.jp/kegg-bin/show_pathway?ko0
5. Organis	5.1 Immur	Toll and Ir	ko04624	2	35	0.380061	0.999979	ANK1|DU(	http://www.genome.jp/kegg-bin/show_pathway?ko0
3. Environ	3.2 Signal	Two-comp	ko02020	1	13	0.391857	0.999979	ALPI	http://www.genome.jp/kegg-bin/show_pathway?ko0
1. Metabo	1.8 Metab	Thiamine	ko00730	1	13	0.391857	0.999979	ALPI	http://www.genome.jp/kegg-bin/show_pathway?ko0
5. Organis	5.2 Endoc	Melanoge	ko04916	4	86	0.404493	0.999979	ASIP|CREB	http://www.genome.jp/kegg-bin/show_pathway?ko0
1. Metabo	1.1 Carbol	Fructose a	ko00051	2	37	0.406812	0.999979	FBP2|HKD	http://www.genome.jp/kegg-bin/show_pathway?ko0
5. Organis	5.4 Digest	Fat digesti	ko04975	2	37	0.406812	0.999979	AGPAT2|F.	http://www.genome.jp/kegg-bin/show_pathway?ko0
1. Metabo	1.5 Amino	Phenylalar	ko00360	1	14	0.414694	0.999979	IL4I1	http://www.genome.jp/kegg-bin/show_pathway?ko0
1. Metabo	1.7 Glycan	Various ty	ko00513	2	40	0.445849	0.999979	ALG12|MA	http://www.genome.jp/kegg-bin/show_pathway?ko0

1. Metabo	1.8 Metab	One carbo	ko00670	1	16	0.457831	0.999979	ATIC	http://www.genome.jp/kegg-bin/show_pathway?ko0
5. Organis	5.2 Endoc	Renin-ang	ko04614	1	16	0.457831	0.999979	ENPEP	http://www.genome.jp/kegg-bin/show_pathway?ko0
3. Environ	3.2 Signal	FoxO sign	ko04068	5	120	0.470524	0.999979	CCNB3|CD	http://www.genome.jp/kegg-bin/show_pathway?ko0
4. Cellular	4.2 Cell gr	p53 signal	ko04115	3	68	0.472261	0.999979	CDK6|GAD	http://www.genome.jp/kegg-bin/show_pathway?ko0
1. Metabo	1.2 Energy	Nitrogen r	ko00910	1	17	0.478194	0.999979	CA5A	http://www.genome.jp/kegg-bin/show_pathway?ko0
1. Metabo	1.8 Metab	Pantothen	ko00770	1	17	0.478194	0.999979	PANK1	http://www.genome.jp/kegg-bin/show_pathway?ko0
2. Genetic	2.3 Folding	Protein pr	ko04141	6	152	0.508347	0.999979	DNAJB11|I	http://www.genome.jp/kegg-bin/show_pathway?ko0
5. Organis	5.6 Nervou	Synaptic v	ko04721	3	73	0.519712	0.999979	ATP6V0D2	http://www.genome.jp/kegg-bin/show_pathway?ko0
1. Metabo	1.7 Glycan	N-Glycan	ko00510	2	48	0.542368	0.999979	ALG12|MA	http://www.genome.jp/kegg-bin/show_pathway?ko0
5. Organis	5.8 Develo	Dorso-ver	ko04320	1	21	0.552292	0.999979	SPIRE2	http://www.genome.jp/kegg-bin/show_pathway?ko0
1. Metabo	1.9 Metab	Terpenoid	ko00900	1	21	0.552292	0.999979	FDPS	http://www.genome.jp/kegg-bin/show_pathway?ko0
5. Organis	5.2 Endoc	Regulation	ko04923	2	49	0.553586	0.999979	FABP4|PN	http://www.genome.jp/kegg-bin/show_pathway?ko0
5. Organis	5.2 Endoc	GnRH secr	ko04929	2	49	0.553586	0.999979	ARR3|CAC	http://www.genome.jp/kegg-bin/show_pathway?ko0
5. Organis	5.2 Endoc	Cortisol sy	ko04927	2	49	0.553586	0.999979	CACNA1H	http://www.genome.jp/kegg-bin/show_pathway?ko0
1. Metabo	1.5 Amino	Histidine r	ko00340	1	22	0.569113	0.999979	ALDH1A3	http://www.genome.jp/kegg-bin/show_pathway?ko0
2. Genetic	2.3 Folding	Protein ex	ko03060	1	22	0.569113	0.999979	HSPA5	http://www.genome.jp/kegg-bin/show_pathway?ko0
1. Metabo	1.4 Nucleo	Pyrimidine	ko00240	2	51	0.575433	0.999979	RRM2|TK1	http://www.genome.jp/kegg-bin/show_pathway?ko0
5. Organis	5.4 Digest	Bile secret	ko04976	2	51	0.575433	0.999979	CYP7A1|SI	http://www.genome.jp/kegg-bin/show_pathway?ko0
1. Metabo	1.7 Glycan	Glycosami	ko00534	1	23	0.585303	0.999979	HS6ST3	http://www.genome.jp/kegg-bin/show_pathway?ko0
5. Organis	5.2 Endoc	Parathyroi	ko04928	3	83	0.607125	0.999979	ARR3|CAS	http://www.genome.jp/kegg-bin/show_pathway?ko0
5. Organis	5.4 Digest	Vitamin di	ko04977	1	26	0.630319	0.999979	TCN2	http://www.genome.jp/kegg-bin/show_pathway?ko0
1. Metabo	1.1 Carbol	Citrate cyc	ko00020	1	26	0.630319	0.999979	PDHA2	http://www.genome.jp/kegg-bin/show_pathway?ko0
1. Metabo	1.1 Carbol	Pentose p	ko00030	1	28	0.657586	0.999979	FBP2	http://www.genome.jp/kegg-bin/show_pathway?ko0
5. Organis	5.6 Nervou	Cholinergi	ko04725	3	90	0.661667	0.999979	CHRNA3|(	http://www.genome.jp/kegg-bin/show_pathway?ko0
5. Organis	5.5 Excreto	Aldosteron	ko04960	1	29	0.670456	0.999979	SGK1	http://www.genome.jp/kegg-bin/show_pathway?ko0
2. Genetic	2.4 Replica	Base excis	ko03410	1	31	0.694767	0.999979	UNG	http://www.genome.jp/kegg-bin/show_pathway?ko0
4. Cellular	4.2 Cell gr	Ferroptosi	ko04216	1	32	0.706242	0.999979	ACSL1	http://www.genome.jp/kegg-bin/show_pathway?ko0
5. Organis	5.2 Endoc	Insulin sec	ko04911	2	65	0.706391	0.999979	CREB3L3|F	http://www.genome.jp/kegg-bin/show_pathway?ko0
4. Cellular	4.1 Transp	Phagosom	ko04145	5	159	0.716781	0.999979	ATP6V0D2	http://www.genome.jp/kegg-bin/show_pathway?ko0
1. Metabo	1.5 Amino	Alanine, as	ko00250	1	33	0.717287	0.999979	IL4I1	http://www.genome.jp/kegg-bin/show_pathway?ko0
1. Metabo	1.3 Lipid n	Linoleic ac	ko00591	1	33	0.717287	0.999979	CYP3A5	http://www.genome.jp/kegg-bin/show_pathway?ko0
4. Cellular	4.1 Transp	Endocytos	ko04144	8	252	0.733687	0.999979	ARR3|DNM	http://www.genome.jp/kegg-bin/show_pathway?ko0
5. Organis	5.4 Digest	Carbohydr	ko04973	1	35	0.738148	0.999979	HKDC1	http://www.genome.jp/kegg-bin/show_pathway?ko0
5. Organis	5.1 Immur	Antigen p	ko04612	3	103	0.74791	0.999979	LOC1211C	http://www.genome.jp/kegg-bin/show_pathway?ko0
5. Organis	5.2 Endoc	Aldosteron	ko04925	2	71	0.751304	0.999979	CACNA1H	http://www.genome.jp/kegg-bin/show_pathway?ko0
2. Genetic	2.1 Transc	Basal trans	ko03022	1	37	0.757472	0.999979	TAF10	http://www.genome.jp/kegg-bin/show_pathway?ko0
5. Organis	5.2 Endoc	Progester	ko04914	2	73	0.764904	0.999979	BUB1|CCN	http://www.genome.jp/kegg-bin/show_pathway?ko0
3. Environ	3.1 Memb	ABC trans	ko02010	1	38	0.766594	0.999979	ABCA12	http://www.genome.jp/kegg-bin/show_pathway?ko0

5. Organis	5.5 Excreto	Endocrine	ko04961	1	39	0.775373	0.999979	DNM1	http://www.genome.jp/kegg-bin/show_pathway?ko0
1. Metabo	1.5 Amino	Glycine, se	ko00260	1	39	0.775373	0.999979	GLDC	http://www.genome.jp/kegg-bin/show_pathway?ko0
5. Organis	5.2 Endocı	Thyroid hc	ko04919	3	111	0.791645	0.999979	MED13|RC	http://www.genome.jp/kegg-bin/show_pathway?ko0
1. Metabo	1.5 Amino	Cysteine a	ko00270	1	43	0.807314	0.999979	IL4I1	http://www.genome.jp/kegg-bin/show_pathway?ko0
5. Organis	5.7 Sensor	Taste trans	ko04742	1	43	0.807314	0.999979	TRPM5	http://www.genome.jp/kegg-bin/show_pathway?ko0
3. Environı	3.3 Signali	ECM-rece	ko04512	2	80	0.807529	0.999979	ITGB6|LAN	http://www.genome.jp/kegg-bin/show_pathway?ko0
5. Organis	5.3 Circula	Adrenergi	ko04261	3	115	0.811037	0.999979	CREB3L3|k	http://www.genome.jp/kegg-bin/show_pathway?ko0
3. Environı	3.2 Signal	TNF signa	ko04668	2	82	0.818377	0.999979	CREB3L3|S	http://www.genome.jp/kegg-bin/show_pathway?ko0
3. Environı	3.2 Signal	MAPK sigr	ko04010	7	247	0.823844	0.999979	ARR3|CAC	http://www.genome.jp/kegg-bin/show_pathway?ko0
5. Organis	5.9 Aging	Longevity	ko04211	2	84	0.828674	0.999979	CREB3L3|F	http://www.genome.jp/kegg-bin/show_pathway?ko0
3. Environı	3.2 Signal	Hedgehog	ko04340	1	47	0.834719	0.999979	ARR3	http://www.genome.jp/kegg-bin/show_pathway?ko0
5. Organis	5.8 Develo	Axon rege	ko04361	2	86	0.838443	0.999979	DUSP4|PS	http://www.genome.jp/kegg-bin/show_pathway?ko0
5. Organis	5.4 Digest	Protein dig	ko04974	2	86	0.838443	0.999979	DPP4|KCN	http://www.genome.jp/kegg-bin/show_pathway?ko0
2. Genetic	2.4 Replica	Fanconi ar	ko03460	1	49	0.846926	0.999979	RMI2	http://www.genome.jp/kegg-bin/show_pathway?ko0
1. Metabo	1.3 Lipid n	Sphingolip	ko00600	1	50	0.852688	0.999979	SGPP2	http://www.genome.jp/kegg-bin/show_pathway?ko0
5. Organis	5.6 Nervou	Long-term	ko04720	1	51	0.858233	0.999979	GRIN2C	http://www.genome.jp/kegg-bin/show_pathway?ko0
5. Organis	5.2 Endocı	Growth hc	ko04935	3	127	0.86027	0.999979	CREB3L3|(	http://www.genome.jp/kegg-bin/show_pathway?ko0
4. Cellular	4.2 Cell gr	Oocyte me	ko04114	2	93	0.868785	0.999979	BUB1|PTT(	http://www.genome.jp/kegg-bin/show_pathway?ko0
3. Environı	3.2 Signal	PI3K-Akt s	ko04151	8	297	0.87307	0.999979	CDK6|CRE	http://www.genome.jp/kegg-bin/show_pathway?ko0
5. Organis	5.4 Digest	Gastric aci	ko04971	1	55	0.878406	0.999979	KCNQ1	http://www.genome.jp/kegg-bin/show_pathway?ko0
4. Cellular	4.1 Transp	Lysosome	ko04142	3	133	0.880385	0.999979	ATP6V0D2	http://www.genome.jp/kegg-bin/show_pathway?ko0
3. Environı	3.2 Signal	Phospholi	ko04072	3	133	0.880385	0.999979	AGPAT2|D	http://www.genome.jp/kegg-bin/show_pathway?ko0
3. Environı	3.2 Signal	Notch sigr	ko04330	1	57	0.887391	0.999979	RBPJ	http://www.genome.jp/kegg-bin/show_pathway?ko0
3. Environı	3.2 Signal	HIF-1 sigr	ko04066	2	99	0.89053	0.999979	HKDC1|PD	http://www.genome.jp/kegg-bin/show_pathway?ko0
3. Environı	3.3 Signali	Cell adhes	ko04514	4	173	0.893422	0.999979	LOC12110	http://www.genome.jp/kegg-bin/show_pathway?ko0
5. Organis	5.6 Nervou	Dopamine	ko04728	2	100	0.893812	0.999979	ARR3|CRE	http://www.genome.jp/kegg-bin/show_pathway?ko0
5. Organis	5.10 Enviro	Circadian	ko04713	2	100	0.893812	0.999979	CACNA1H	http://www.genome.jp/kegg-bin/show_pathway?ko0
5. Organis	5.2 Endocı	Estrogen s	ko04915	2	101	0.897002	0.999979	CREB3L3|H	http://www.genome.jp/kegg-bin/show_pathway?ko0
5. Organis	5.2 Endocı	Relaxin sig	ko04926	2	104	0.906045	0.999979	ARR3|CRE	http://www.genome.jp/kegg-bin/show_pathway?ko0
3. Environı	3.2 Signal	cAMP sigr	ko04024	5	217	0.913892	0.999979	ACOX1|CF	http://www.genome.jp/kegg-bin/show_pathway?ko0
5. Organis	5.2 Endocı	Prolactin s	ko04917	1	66	0.920293	0.999979	SOCS3	http://www.genome.jp/kegg-bin/show_pathway?ko0
5. Organis	5.1 Immur	Natural kil	ko04650	1	67	0.923297	0.999979	FASLG	http://www.genome.jp/kegg-bin/show_pathway?ko0
5. Organis	5.1 Immur	IL-17 sign	ko04657	1	69	0.928969	0.999979	HSP90B1	http://www.genome.jp/kegg-bin/show_pathway?ko0
5. Organis	5.4 Digest	Salivary se	ko04970	1	71	0.934222	0.999979	LOC10175	http://www.genome.jp/kegg-bin/show_pathway?ko0
5. Organis	5.1 Immur	Toll-like re	ko04620	1	72	0.936701	0.999979	TLR5	http://www.genome.jp/kegg-bin/show_pathway?ko0
1. Metabo	1.2 Energy	Oxidative	ko00190	2	120	0.942956	0.999979	ATP6V0D2	http://www.genome.jp/kegg-bin/show_pathway?ko0
4. Cellular	4.3 Cellula	Gap juncti	ko04540	1	76	0.94572	0.999979	GJA1	http://www.genome.jp/kegg-bin/show_pathway?ko0
5. Organis	5.1 Immur	Th1 and T	ko04658	1	77	0.947767	0.999979	RBPJ	http://www.genome.jp/kegg-bin/show_pathway?ko0

5. Organis	5.10 Envir	Thermoge	ko04714	5	241	0.950815	0.999979	ACSL1|CP	http://www.genome.jp/kegg-bin/show_pathway?ko0
2. Genetic	2.3 Folding	Ubiquitin	ko04120	2	125	0.951326	0.999979	SOCS3|UB	http://www.genome.jp/kegg-bin/show_pathway?ko0
3. Environ	3.2 Signal	NF-kappa	ko04064	1	80	0.953456	0.999979	CARD10	http://www.genome.jp/kegg-bin/show_pathway?ko0
5. Organis	5.1 Immur	NOD-like	ko04621	2	127	0.954335	0.999979	CASR|LOC	http://www.genome.jp/kegg-bin/show_pathway?ko0
4. Cellular	4.2 Cell gr	Apoptosis	ko04210	2	128	0.955771	0.999979	FASLG|GA	http://www.genome.jp/kegg-bin/show_pathway?ko0
3. Environ	3.2 Signal	Jak-STAT	ko04630	2	132	0.961098	0.999979	IL22RA1|S	http://www.genome.jp/kegg-bin/show_pathway?ko0
5. Organis	5.4 Digest	Pancreatic	ko04972	1	85	0.961596	0.999979	KCNQ1	http://www.genome.jp/kegg-bin/show_pathway?ko0
3. Environ	3.2 Signal	TGF-beta	ko04350	1	87	0.96444	0.999979	CDKN2B	http://www.genome.jp/kegg-bin/show_pathway?ko0
5. Organis	5.6 Nervou	Glutamate	ko04724	1	88	0.965781	0.999979	GRIN2C	http://www.genome.jp/kegg-bin/show_pathway?ko0
1. Metabo	1.4 Nucleo	Purine me	ko00230	2	141	0.970925	0.999979	ATIC|RRM	http://www.genome.jp/kegg-bin/show_pathway?ko0
5. Organis	5.1 Immur	Th17 cell	ko04659	1	93	0.971769	0.999979	AHR1B	http://www.genome.jp/kegg-bin/show_pathway?ko0
3. Environ	3.2 Signal	mTOR sig	ko04150	2	144	0.973633	0.999979	SGK1|WN	http://www.genome.jp/kegg-bin/show_pathway?ko0
5. Organis	5.8 Develo	Osteoclast	ko04380	1	97	0.975797	0.999979	SOCS3	http://www.genome.jp/kegg-bin/show_pathway?ko0
3. Environ	3.2 Signal	Sphingolip	ko04071	1	106	0.982884	0.999979	SGPP2	http://www.genome.jp/kegg-bin/show_pathway?ko0
5. Organis	5.8 Develo	Axon guid	ko04360	2	160	0.984431	0.999979	DPYSL2|EF	http://www.genome.jp/kegg-bin/show_pathway?ko0
3. Environ	3.2 Signal	Calcium si	ko04020	2	161	0.98494	0.999979	CACNA1H	http://www.genome.jp/kegg-bin/show_pathway?ko0
5. Organis	5.6 Nervou	Neurotrop	ko04722	1	110	0.985328	0.999979	FASLG	http://www.genome.jp/kegg-bin/show_pathway?ko0
5. Organis	5.1 Immur	Leukocyte	ko04670	1	112	0.986416	0.999979	RAPGEF4	http://www.genome.jp/kegg-bin/show_pathway?ko0
5. Organis	5.6 Nervou	Retrograd	ko04723	1	120	0.99002	0.999979	ABHD6	http://www.genome.jp/kegg-bin/show_pathway?ko0
4. Cellular	4.2 Cell gr	Necroptos	ko04217	1	124	0.991446	0.999979	FASLG	http://www.genome.jp/kegg-bin/show_pathway?ko0
5. Organis	5.1 Immur	T cell rece	ko04660	1	125	0.991769	0.999979	PDCD1	http://www.genome.jp/kegg-bin/show_pathway?ko0
3. Environ	3.3 Signali	Neuroacti	ko04080	5	313	0.992263	0.999979	CHRNA3|C	http://www.genome.jp/kegg-bin/show_pathway?ko0
4. Cellular	4.5 Cell m	Regulation	ko04810	2	183	0.992801	0.999979	FGF19|ITG	http://www.genome.jp/kegg-bin/show_pathway?ko0
4. Cellular	4.3 Cellula	Signaling	ko04550	1	129	0.992946	0.999979	WNT11	http://www.genome.jp/kegg-bin/show_pathway?ko0
4. Cellular	4.3 Cellula	Focal adhe	ko04510	2	186	0.993497	0.999979	ITGB6|LAM	http://www.genome.jp/kegg-bin/show_pathway?ko0
3. Environ	3.2 Signal	Rap1 sign	ko04015	2	188	0.993923	0.999979	FGF19|RAI	http://www.genome.jp/kegg-bin/show_pathway?ko0
5. Organis	5.1 Immur	Chemokin	ko04062	1	133	0.993954	0.999979	ARR3	http://www.genome.jp/kegg-bin/show_pathway?ko0
3. Environ	3.2 Signal	Apelin sig	ko04371	1	139	0.995204	0.999979	PPARGC1	http://www.genome.jp/kegg-bin/show_pathway?ko0
3. Environ	3.3 Signali	Cytokine-	ko04060	2	195	0.995212	0.999979	FASLG|IL2	http://www.genome.jp/kegg-bin/show_pathway?ko0
3. Environ	3.2 Signal	cGMP-PK	ko04022	1	140	0.995385	0.999979	CREB3L3	http://www.genome.jp/kegg-bin/show_pathway?ko0
3. Environ	3.2 Signal	Ras signal	ko04014	2	199	0.995824	0.999979	FASLG|FGI	http://www.genome.jp/kegg-bin/show_pathway?ko0
3. Environ	3.2 Signal	Wnt signa	ko04310	1	151	0.996982	0.999979	WNT11	http://www.genome.jp/kegg-bin/show_pathway?ko0
3. Environ	3.2 Signal	Hippo sig	ko04390	1	157	0.997607	0.999979	WNT11	http://www.genome.jp/kegg-bin/show_pathway?ko0
5. Organis	5.7 Sensor	Olfactory	ko04740	1	279	0.999979	0.999979	ARR3	http://www.genome.jp/kegg-bin/show_pathway?ko0

3320
0071
1212
0061
1130
0100
1110
0909
0620
0280
0640
0561
4110
4922
1040
4146
4920
1200
1100
4966
0650
4979
0010
0983
4152
0830
0623
0364
0660
0361
0410
0520
0120
0053
0630
3030

[illegible]0982
[illegible]0980
[illegible]0281
[illegible]0930
[illegible]0400
[illegible]0903
[illegible]0052
[illegible]0564
[illegible]4910
[illegible]4978
[illegible]0981
[illegible]0627
[illegible]0140
[illegible]0040
[illegible]0625
[illegible]0380
[illegible]0480
[illegible]0330
[illegible]4218
[illegible]0680
[illegible]0062
[illegible]0790
[illegible]4918
[illegible]2024
[illegible]0592
[illegible]0950
[illegible]0072
[illegible]0310
[illegible]0500
[illegible]0350
[illegible]4624
[illegible]2020
[illegible]0730
[illegible]4916
[illegible]0051
[illegible]4975
[illegible]0360
[illegible]0513

0670
4614
4068
4115
0910
0770
4141
4721
0510
4320
0900
4923
4929
4927
0340
3060
0240
4976
0534
4928
4977
0020
0030
4725
4960
3410
4216
4911
4145
0250
0591
4144
4973
4612
4925
3022
4914
2010

4961
0260
4919
0270
4742
4512
4261
4668
4010
4211
4340
4361
4974
3460
0600
4720
4935
4114
4151
4971
4142
4072
4330
4066
4514
4728
4713
4915
4926
4024
4917
4650
4657
4970
4620
0190
4540
4658

04714
04120
04064
04621
04210
04630
04972
04350
04724
00230
04659
04150
04380
04071
04360
04020
04722
04670
04723
04217
04660
04080
04810
04550
04510
04015
04062
04371
04060
04022
04014
04310
04390
04740
