# Supplementary material for: Comparative analysis of liver transcriptome reveals adaptive responses to hypoxia environmental condition in Tibetan chicken
Source: Anim Biosci. 2023 Aug 23;37(1):28–38. doi: 10.5713/ab.23.0126 (PMC10766467; doi:10.5713/ab.23.0126)
Supplement: Supplementary file 9 [file ab-23-0126-Supplementary-Table-9.pdf]

Table S9

layer1	layer2	layer3	ID	Input.num	Backgrour	pvalue	FDR	Input
1. Metabo	1.3 Lipid n	Fatty acid	ko00071	7	36	2.54E-05	0.003205	ACAA1|AC
5. Organis	5.2 Endoc	PPAR sign	ko03320	9	69	5.12E-05	0.003227	ACAA1|AC
1. Metabo	1.0 Global	Fatty acid	ko01212	6	59	0.003439	0.144424	ACAA1|AC
4. Cellular	4.1 Transp	Peroxisom	ko04146	7	79	0.003543	0.111597	ACAA1|AC
1. Metabo	1.1 Carbol	Propanoat	ko00640	4	31	0.007077	0.178351	ACACB|AC
1. Metabo	1.3 Lipid n	Fatty acid	ko00061	3	20	0.012873	0.270326	ACACB|AC
1. Metabo	1.5 Amino	Valine, leu	ko00280	4	49	0.033654	0.605771	ACAA1|EH
1. Metabo	1.11 Xeno	Drug meta	ko00982	3	32	0.045018	0.709034	GSTK1|GS
5. Organis	5.2 Endoc	Glucagon	ko04922	5	78	0.045485	0.636786	ACACB|CF
1. Metabo	1.11 Xeno	Fluoroben	ko00364	1	2	0.049395	0.622373	CMBL
1. Metabo	1.11 Xeno	Chlorocyc	ko00361	1	2	0.049395	0.565793	CMBL
1. Metabo	1.11 Xeno	Toluene d	ko00623	1	2	0.049395	0.518644	CMBL
1. Metabo	1.11 Xeno	Metabolis	ko00980	3	34	0.052389	0.50777	GSTK1|GS
1. Metabo	1.8 Metab	Retinol me	ko00830	3	40	0.077679	0.699111	CYP3A5|L(
5. Organis	5.2 Endoc	Adipocyto	ko04920	4	66	0.082885	0.696231	ACACB|AC
1. Metabo	1.9 Metab	Geraniol c	ko00281	1	4	0.096358	0.758818	HMGCL
1. Metabo	1.11 Xeno	Caprolacta	ko00930	1	4	0.096358	0.714181	EHHADH
1. Metabo	1.1 Carbol	Butanoate	ko00650	2	23	0.111875	0.783124	EHHADH|I
1. Metabo	1.11 Xeno	Aminober	ko00627	1	5	0.118965	0.788926	EHHADH
1. Metabo	1.2 Energy	Methane r	ko00680	2	26	0.137014	0.863186	ACSS1B|FE
1. Metabo	1.8 Metab	Folate bio	ko00790	2	27	0.14565	0.873898	ALPI|FPGS
3. Environ	3.2 Signal	AMPK sigr	ko04152	5	112	0.149128	0.854094	ACACB|CF
1. Metabo	1.6 Metab	beta-Alan	ko00410	2	28	0.154394	0.845813	ACOX1|EH
5. Organis	5.4 Digest	Cholester	ko04979	3	55	0.158306	0.831105	CETP|LIPC
1. Metabo	1.3 Lipid n	alpha-Linc	ko00592	2	29	0.163237	0.822717	ACAA1|AC
1. Metabo	1.11 Xeno	Drug meta	ko00983	3	56	0.164377	0.796598	CES1L2|GS
1. Metabo	1.1 Carbol	Glyoxylate	ko00630	2	31	0.181177	0.845493	ACSS1B|H
1. Metabo	1.3 Lipid n	Biosynthes	ko01040	2	31	0.181177	0.815297	ACAA1|AC
1. Metabo	1.3 Lipid n	Glycerolip	ko00561	3	61	0.195695	0.850261	GPAT3|LIP
4. Cellular	4.4 Cellula	Quorum s	ko02024	1	9	0.203896	0.856364	ACSL1
1. Metabo	1.3 Lipid n	Synthesis	ko00072	1	10	0.223822	0.909729	HMGCL
1. Metabo	1.1 Carbol	Pyruvate r	ko00620	2	36	0.227074	0.894104	ACACB|AC
5. Organis	5.4 Digest	Mineral ab	ko04978	2	38	0.245679	0.938048	MT3|MT4
1. Metabo	1.0 Global	Biosynthes	ko01130	7	204	0.249848	0.925907	ACAA1|AC
1. Metabo	1.3 Lipid n	Steroid hc	ko00140	2	39	0.255005	0.918019	CYP3A5|L(
1. Metabo	1.0 Global	Carbon m	ko01200	4	106	0.273739	0.958086	ACOX1|AC
1. Metabo	1.8 Metab	Thiamine	ko00730	1	13	0.280667	0.955784	ALPI
3. Environ	3.2 Signal	Two-comp	ko02020	1	13	0.280667	0.930632	ALPI
1. Metabo	1.6 Metab	Glutathior	ko00480	2	45	0.310902	0.999512	GSTK1|GS
1. Metabo	1.1 Carbol	Ascorbate	ko00053	1	15	0.316239	0.996152	LOC12111
1. Metabo	1.8 Metab	One carbo	ko00670	1	16	0.333362	0.999512	ATIC
5. Organis	5.2 Endoc	Parathyroi	ko04928	3	83	0.344113	0.999512	ARR3|CAS
1. Metabo	1.2 Energy	Nitrogen r	ko00910	1	17	0.350058	0.999512	CA5A
1. Metabo	1.8 Metab	Pantother	ko00770	1	17	0.350058	0.999512	PANK1
5. Organis	5.2 Endoc	Melanoge	ko04916	3	86	0.364694	0.999512	ASIP|CREB
1. Metabo	1.1 Carbol	Glycolysis	ko00010	2	56	0.410418	0.999512	ACSS1B|FE
5. Organis	5.8 Develc	Dorso-ver	ko04320	1	21	0.412777	0.999512	SPIRE2
1. Metabo	1.1 Carbol	Pentose ai	ko00040	1	21	0.412777	0.999512	LOC12111
1. Metabo	1.0 Global	Biosynthes	ko01110	11	406	0.436704	0.999512	ACAA1|AC
5. Organis	5.5 Excret	Collecting	ko04966	1	23	0.441837	0.999512	SLC4A1
1. Metabo	1.7 Glycan	Glycosami	ko00534	1	23	0.441837	0.999512	HS6ST3
4. Cellular	4.1 Transp	Endocytos	ko04144	7	252	0.443241	0.999512	ARR3|DNM
5. Organis	5.1 Immur	Antigen p	ko04612	3	103	0.478003	0.999512	LOC12110
5. Organis	5.2 Endoc	Insulin sec	ko04911	2	65	0.486397	0.999512	CREB3L3|F
1. Metabo	1.3 Lipid n	Fatty acid	ko00062	1	27	0.495727	0.999512	HADHB
1. Metabo	1.1 Carbol	Pentose p	ko00030	1	28	0.508369	0.999512	FBP2

2. Genetic	2.4 Replica	Base excis	ko03410	1	31	0.544431	0.999512	UNG
4. Cellular	4.2 Cell gr	Ferroptosi	ko04216	1	32	0.555856	0.999512	ACSL1
1. Metabo	1.3 Lipid n	Linoleic ac	ko00591	1	33	0.566996	0.999512	CYP3A5
1. Metabo	1.5 Amino	Tyrosine n	ko00350	1	33	0.566996	0.999512	DCT
4. Cellular	4.2 Cell gr	Cellular se	ko04218	4	160	0.570685	0.999512	LOC1211C
5. Organis	5.2 Endoc	Insulin sig	ko04910	3	120	0.58109	0.999512	ACACB|FB
5. Organis	5.1 Immur	Toll and Ir	ko04624	1	35	0.588446	0.999512	ANK1
1. Metabo	1.1 Carbol	Galactose	ko00052	1	36	0.598771	0.999512	GALE
5. Organis	5.4 Digest	Fat digesti	ko04975	1	37	0.608838	0.999512	FABP2
1. Metabo	1.1 Carbol	Fructose a	ko00051	1	37	0.608838	0.999512	FBP2
2. Genetic	2.1 Transc	Basal trans	ko03022	1	37	0.608838	0.999512	TAF10
3. Environ	3.1 Memb	ABC trans	ko02010	1	38	0.618653	0.999512	ABCA12
5. Organis	5.9 Aging	Longevity	ko04211	2	84	0.625218	0.999512	CREB3L3|F
5. Organis	5.5 Excret	Endocrine	ko04961	1	39	0.628223	0.999512	DNM1
3. Environ	3.2 Signal	cAMP sigr	ko04024	5	217	0.636075	0.999512	ACOX1|CF
1. Metabo	1.7 Glycan	Various ty	ko00513	1	40	0.637553	0.999512	MAN1B1
1. Metabo	1.5 Amino	Tryptopha	ko00380	1	42	0.65552	0.999512	EHHADH
5. Organis	5.6 Nervo	Cholinergi	ko04725	2	90	0.662585	0.999512	CHRNA3|C
1. Metabo	1.5 Amino	Arginine a	ko00330	1	46	0.688834	0.999512	HOGA1
1. Metabo	1.1 Carbol	Amino suc	ko00520	1	46	0.688834	0.999512	GALE
3. Environ	3.2 Signal	Hedgehog	ko04340	1	47	0.696648	0.999512	ARR3
1. Metabo	1.7 Glycan	N-Glycan	ko00510	1	48	0.704267	0.999512	MAN1B1
5. Organis	5.2 Endoc	Cortisol sy	ko04927	1	49	0.711695	0.999512	CREB3L3
5. Organis	5.2 Endoc	Regulation	ko04923	1	49	0.711695	0.999512	FABP4
5. Organis	5.2 Endoc	GnRH sec	ko04929	1	49	0.711695	0.999512	ARR3
2. Genetic	2.4 Replica	Fanconi ar	ko03460	1	49	0.711695	0.999512	RMI2
5. Organis	5.6 Nervo	Dopamine	ko04728	2	100	0.718152	0.999512	ARR3|CRE
5. Organis	5.2 Endoc	Thyroid hc	ko04918	1	50	0.718937	0.999512	CREB3L3
1. Metabo	1.3 Lipid n	Sphingolip	ko00600	1	50	0.718937	0.999512	SGPP2
5. Organis	5.4 Digest	Bile secret	ko04976	1	51	0.725997	0.999512	SLC51AL
5. Organis	5.6 Nervo	Long-tern	ko04720	1	51	0.725997	0.999512	GRIN2C
5. Organis	5.2 Endoc	Relaxin sic	ko04926	2	104	0.738134	0.999512	ARR3|CRE
1. Metabo	1.5 Amino	Lysine deç	ko00310	1	53	0.739593	0.999512	EHHADH
4. Cellular	4.1 Transp	Phagosom	ko04145	3	159	0.764213	0.999512	LOC1211C
3. Environ	3.2 Signal	Notch sigr	ko04330	1	57	0.764801	0.999512	RBPJ
5. Organis	5.2 Endoc	Thyroid hc	ko04919	2	111	0.770201	0.999512	MED13|RC
5. Organis	5.3 Circula	Adrenergi	ko04261	2	115	0.786952	0.999512	CREB3L3|F
3. Environ	3.3 Signali	Cell adhes	ko04514	3	173	0.81181	0.999512	LOC1211C
5. Organis	5.2 Endoc	Growth hc	ko04935	2	127	0.830928	0.999512	CREB3L3|C
5. Organis	5.2 Endoc	Aldosteroı	ko04925	1	71	0.835354	0.999512	CREB3L3
5. Organis	5.1 Immur	Toll-like re	ko04620	1	72	0.839498	0.999512	TLR5
5. Organis	5.6 Nervo	Synaptic v	ko04721	1	73	0.843538	0.999512	DNM1
3. Environ	3.2 Signal	Phospholi	ko04072	2	133	0.849706	0.999512	DNM1|RA
5. Organis	5.10 Envir	Thermoge	ko04714	4	241	0.857433	0.999512	ACSL1|CP
5. Organis	5.1 Immur	Th1 and T	ko04658	1	77	0.85871	0.999512	RBPJ
3. Environ	3.2 Signal	NF-kappa	ko04064	1	80	0.869118	0.999512	CARD10
3. Environ	3.2 Signal	TNF signa	ko04668	1	82	0.875629	0.999512	CREB3L3
5. Organis	5.8 Develc	Axon rege	ko04361	1	86	0.887699	0.999512	PSD3
5. Organis	5.6 Nervo	Glutamate	ko04724	1	88	0.893288	0.999512	GRIN2C
5. Organis	5.1 Immur	Th17 cell c	ko04659	1	93	0.906079	0.999512	AHR1B
1. Metabo	1.3 Lipid n	Glyceroph	ko00564	1	98	0.917342	0.999512	GPAT3
5. Organis	5.10 Envir	Circadian	ko04713	1	100	0.92146	0.999512	GRIN2C
5. Organis	5.2 Endoc	Estrogen s	ko04915	1	101	0.923442	0.999512	CREB3L3
3. Environ	3.2 Signal	Sphingolip	ko04071	1	106	0.932629	0.999512	SGPP2
1. Metabo	1.0 Global	Metabolic	ko01100	27	1385	0.936713	0.999512	ACAA1|AC
5. Organis	5.1 Immur	Leukocyte	ko04670	1	112	0.942214	0.999512	RAPGEF4
5. Organis	5.6 Nervo	Retrograd	ko04723	1	120	0.952914	0.999512	ABHD6
3. Environ	3.3 Signali	Neuroacti	ko04080	4	313	0.956442	0.999512	CHRNA3|C

5. Organis	5.1 Immur	NOD-like	ko04621	1	127	0.960642	0.999512	CASR
4. Cellular	4.1 Transp	Lysosome	ko04142	1	133	0.966252	0.999512	TPP1
5. Organis	5.1 Immur	Chemokin	ko04062	1	133	0.966252	0.999512	ARR3
3. Environ	3.2 Signal	Apelin sig	ko04371	1	139	0.971065	0.999512	PPARGC1A
3. Environ	3.2 Signal	cGMP-PK(	ko04022	1	140	0.971797	0.999512	CREB3L3
1. Metabo	1.4 Nucleo	Purine me	ko00230	1	141	0.972512	0.999512	ATIC
2. Genetic	2.3 Folding	Protein pr	ko04141	1	152	0.979273	0.999512	MAN1B1
3. Environ	3.2 Signal	Calcium si	ko04020	1	161	0.983552	0.999512	GRIN2C
3. Environ	3.2 Signal	Rap1 signa	ko04015	1	188	0.99179	0.999512	RAPGEF4
3. Environ	3.2 Signal	MAPK sign	ko04010	1	247	0.998212	0.999512	ARR3
5. Organis	5.7 Sensor	Olfactory	ko04740	1	279	0.999221	0.999512	ARR3
3. Environ	3.2 Signal	PI3K-Akt s	ko04151	1	297	0.999512	0.999512	CREB3L3

Hyperlink
http://www.genome.jp/kegg-bin/show_pathway?ko00071/K00232%09yellow/K01897%09yellow/
http://www.genome.jp/kegg-bin/show_pathway?ko03320/K00232%09yellow/K01059%09yellow/
http://www.genome.jp/kegg-bin/show_pathway?ko01212/K00232%09yellow/K01897%09yellow/
http://www.genome.jp/kegg-bin/show_pathway?ko04146/K00232%09yellow/K01640%09yellow/
http://www.genome.jp/kegg-bin/show_pathway?ko00640/K00232%09yellow/K01895%09yellow/
http://www.genome.jp/kegg-bin/show_pathway?ko00061/K01071%09yellow/K01897%09yellow/
http://www.genome.jp/kegg-bin/show_pathway?ko00280/K01640%09yellow/K07509%09yellow/
http://www.genome.jp/kegg-bin/show_pathway?ko00982/K00699%09yellow/K00799%09yellow/
http://www.genome.jp/kegg-bin/show_pathway?ko04922/K01946%09yellow/K03841%09yellow/
http://www.genome.jp/kegg-bin/show_pathway?ko00364/K01061%09yellow
http://www.genome.jp/kegg-bin/show_pathway?ko00361/K01061%09yellow
http://www.genome.jp/kegg-bin/show_pathway?ko00623/K01061%09yellow
http://www.genome.jp/kegg-bin/show_pathway?ko00980/K00699%09yellow/K00799%09yellow/
http://www.genome.jp/kegg-bin/show_pathway?ko00830/K00699%09yellow/K07424%09yellow/
http://www.genome.jp/kegg-bin/show_pathway?ko04920/K01897%09yellow/K01946%09yellow/
http://www.genome.jp/kegg-bin/show_pathway?ko00281/K01640%09yellow
http://www.genome.jp/kegg-bin/show_pathway?ko00930/K07514%09yellow
http://www.genome.jp/kegg-bin/show_pathway?ko00650/K01640%09yellow/K07514%09yellow
http://www.genome.jp/kegg-bin/show_pathway?ko00627/K07514%09yellow
http://www.genome.jp/kegg-bin/show_pathway?ko00680/K01895%09yellow/K03841%09yellow
http://www.genome.jp/kegg-bin/show_pathway?ko00790/K01077%09yellow/K01930%09yellow
http://www.genome.jp/kegg-bin/show_pathway?ko04152/K01946%09yellow/K03841%09yellow/
http://www.genome.jp/kegg-bin/show_pathway?ko00410/K00232%09yellow/K07514%09yellow
http://www.genome.jp/kegg-bin/show_pathway?ko04979/K01059%09yellow/K16835%09yellow/
http://www.genome.jp/kegg-bin/show_pathway?ko00592/K00232%09yellow/K07513%09yellow
http://www.genome.jp/kegg-bin/show_pathway?ko00983/K00699%09yellow/K00799%09yellow/
http://www.genome.jp/kegg-bin/show_pathway?ko00630/K01895%09yellow/K18123%09yellow
http://www.genome.jp/kegg-bin/show_pathway?ko01040/K00232%09yellow/K07513%09yellow
http://www.genome.jp/kegg-bin/show_pathway?ko00561/K01059%09yellow/K13506%09yellow/
http://www.genome.jp/kegg-bin/show_pathway?ko02024/K01897%09yellow
http://www.genome.jp/kegg-bin/show_pathway?ko00072/K01640%09yellow
http://www.genome.jp/kegg-bin/show_pathway?ko00620/K01895%09yellow/K01946%09yellow
http://www.genome.jp/kegg-bin/show_pathway?ko04978/K14739%09yellow
http://www.genome.jp/kegg-bin/show_pathway?ko01130/K00602%09yellow/K01895%09yellow/
http://www.genome.jp/kegg-bin/show_pathway?ko00140/K00699%09yellow/K07424%09yellow
http://www.genome.jp/kegg-bin/show_pathway?ko01200/K00232%09yellow/K01895%09yellow/
http://www.genome.jp/kegg-bin/show_pathway?ko00730/K01077%09yellow
http://www.genome.jp/kegg-bin/show_pathway?ko02020/K01077%09yellow
http://www.genome.jp/kegg-bin/show_pathway?ko00480/K00799%09yellow/K13299%09yellow
http://www.genome.jp/kegg-bin/show_pathway?ko00053/K00699%09yellow
http://www.genome.jp/kegg-bin/show_pathway?ko00670/K00602%09yellow
http://www.genome.jp/kegg-bin/show_pathway?ko04928/K04439%09yellow/K04612%09yellow/
http://www.genome.jp/kegg-bin/show_pathway?ko00910/K01672%09yellow
http://www.genome.jp/kegg-bin/show_pathway?ko00770/K09680%09yellow
http://www.genome.jp/kegg-bin/show_pathway?ko04916/K01827%09yellow/K08725%09yellow/
http://www.genome.jp/kegg-bin/show_pathway?ko00010/K01895%09yellow/K03841%09yellow
http://www.genome.jp/kegg-bin/show_pathway?ko04320/K02098%09yellow
http://www.genome.jp/kegg-bin/show_pathway?ko00040/K00699%09yellow
http://www.genome.jp/kegg-bin/show_pathway?ko01110/K00232%09yellow/K00602%09yellow/
http://www.genome.jp/kegg-bin/show_pathway?ko04966/K06573%09yellow
http://www.genome.jp/kegg-bin/show_pathway?ko00534/K08103%09yellow
http://www.genome.jp/kegg-bin/show_pathway?ko04144/K01528%09yellow/K04439%09yellow/
http://www.genome.jp/kegg-bin/show_pathway?ko04612/K06751%09yellow
http://www.genome.jp/kegg-bin/show_pathway?ko04911/K04351%09yellow/K09048%09yellow
http://www.genome.jp/kegg-bin/show_pathway?ko00062/K07509%09yellow
http://www.genome.jp/kegg-bin/show_pathway?ko00030/K03841%09yellow

http://www.genome.jp/kegg-bin/show_pathway?ko03410/K03648%09yellow
http://www.genome.jp/kegg-bin/show_pathway?ko04216/K01897%09yellow
http://www.genome.jp/kegg-bin/show_pathway?ko00591/K07424%09yellow
http://www.genome.jp/kegg-bin/show_pathway?ko00350/K01827%09yellow
http://www.genome.jp/kegg-bin/show_pathway?ko04218/K06751%09yellow/K10995%09yellow
http://www.genome.jp/kegg-bin/show_pathway?ko04910/K01946%09yellow/K03841%09yellow/
http://www.genome.jp/kegg-bin/show_pathway?ko04624/K10380%09yellow
http://www.genome.jp/kegg-bin/show_pathway?ko00052/K01784%09yellow
http://www.genome.jp/kegg-bin/show_pathway?ko04975/K08751%09yellow
http://www.genome.jp/kegg-bin/show_pathway?ko00051/K03841%09yellow
http://www.genome.jp/kegg-bin/show_pathway?ko03022/K03134%09yellow
http://www.genome.jp/kegg-bin/show_pathway?ko02010/K05646%09yellow
http://www.genome.jp/kegg-bin/show_pathway?ko04211/K07202%09yellow/K09048%09yellow
http://www.genome.jp/kegg-bin/show_pathway?ko04961/K01528%09yellow
http://www.genome.jp/kegg-bin/show_pathway?ko04024/K00232%09yellow/K04351%09yellow/
http://www.genome.jp/kegg-bin/show_pathway?ko00513/K23741%09yellow
http://www.genome.jp/kegg-bin/show_pathway?ko00380/K07514%09yellow
http://www.genome.jp/kegg-bin/show_pathway?ko04725/K04805%09yellow/K09048%09yellow
http://www.genome.jp/kegg-bin/show_pathway?ko00330/K18123%09yellow
http://www.genome.jp/kegg-bin/show_pathway?ko00520/K01784%09yellow
http://www.genome.jp/kegg-bin/show_pathway?ko04340/K04439%09yellow
http://www.genome.jp/kegg-bin/show_pathway?ko00510/K23741%09yellow
http://www.genome.jp/kegg-bin/show_pathway?ko04927/K09048%09yellow
http://www.genome.jp/kegg-bin/show_pathway?ko04923/K08753%09yellow
http://www.genome.jp/kegg-bin/show_pathway?ko04929/K04439%09yellow
http://www.genome.jp/kegg-bin/show_pathway?ko03460/K15365%09yellow
http://www.genome.jp/kegg-bin/show_pathway?ko04728/K04439%09yellow/K09048%09yellow
http://www.genome.jp/kegg-bin/show_pathway?ko04918/K09048%09yellow
http://www.genome.jp/kegg-bin/show_pathway?ko00600/K04717%09yellow
http://www.genome.jp/kegg-bin/show_pathway?ko04976/K14360%09yellow
http://www.genome.jp/kegg-bin/show_pathway?ko04720/K05211%09yellow
http://www.genome.jp/kegg-bin/show_pathway?ko04926/K04439%09yellow/K09048%09yellow
http://www.genome.jp/kegg-bin/show_pathway?ko00310/K07514%09yellow
http://www.genome.jp/kegg-bin/show_pathway?ko04145/K06751%09yellow
http://www.genome.jp/kegg-bin/show_pathway?ko04330/K06053%09yellow
http://www.genome.jp/kegg-bin/show_pathway?ko04919/K15164%09yellow/K17903%09yellow
http://www.genome.jp/kegg-bin/show_pathway?ko04261/K04351%09yellow/K09048%09yellow
http://www.genome.jp/kegg-bin/show_pathway?ko04514/K06751%09yellow
http://www.genome.jp/kegg-bin/show_pathway?ko04935/K05254%09yellow/K09048%09yellow
http://www.genome.jp/kegg-bin/show_pathway?ko04925/K09048%09yellow
http://www.genome.jp/kegg-bin/show_pathway?ko04620/K10168%09yellow
http://www.genome.jp/kegg-bin/show_pathway?ko04721/K01528%09yellow
http://www.genome.jp/kegg-bin/show_pathway?ko04072/K01528%09yellow/K04351%09yellow
http://www.genome.jp/kegg-bin/show_pathway?ko04714/K01897%09yellow/K07202%09yellow/
http://www.genome.jp/kegg-bin/show_pathway?ko04658/K06053%09yellow
http://www.genome.jp/kegg-bin/show_pathway?ko04064/K20912%09yellow
http://www.genome.jp/kegg-bin/show_pathway?ko04668/K09048%09yellow
http://www.genome.jp/kegg-bin/show_pathway?ko04361/K12494%09yellow
http://www.genome.jp/kegg-bin/show_pathway?ko04724/K05211%09yellow
http://www.genome.jp/kegg-bin/show_pathway?ko04659/K09093%09yellow
http://www.genome.jp/kegg-bin/show_pathway?ko00564/K13506%09yellow
http://www.genome.jp/kegg-bin/show_pathway?ko04713/K05211%09yellow
http://www.genome.jp/kegg-bin/show_pathway?ko04915/K09048%09yellow
http://www.genome.jp/kegg-bin/show_pathway?ko04071/K04717%09yellow
http://www.genome.jp/kegg-bin/show_pathway?ko01100/K00232%09yellow/K00602%09yellow/
http://www.genome.jp/kegg-bin/show_pathway?ko04670/K04351%09yellow
http://www.genome.jp/kegg-bin/show_pathway?ko04723/K13700%09yellow
http://www.genome.jp/kegg-bin/show_pathway?ko04080/K04805%09yellow/K05206%09yellow/

http://www.genome.jp/kegg-bin/show_pathway?ko04621/K04612%09yellow
http://www.genome.jp/kegg-bin/show_pathway?ko04142/K01279%09yellow
http://www.genome.jp/kegg-bin/show_pathway?ko04062/K04439%09yellow
http://www.genome.jp/kegg-bin/show_pathway?ko04371/K07202%09yellow
http://www.genome.jp/kegg-bin/show_pathway?ko04022/K09048%09yellow
http://www.genome.jp/kegg-bin/show_pathway?ko00230/K00602%09yellow
http://www.genome.jp/kegg-bin/show_pathway?ko04141/K23741%09yellow
http://www.genome.jp/kegg-bin/show_pathway?ko04020/K05211%09yellow
http://www.genome.jp/kegg-bin/show_pathway?ko04015/K04351%09yellow
http://www.genome.jp/kegg-bin/show_pathway?ko04010/K04439%09yellow
http://www.genome.jp/kegg-bin/show_pathway?ko04740/K04439%09yellow
http://www.genome.jp/kegg-bin/show_pathway?ko04151/K09048%09yellow

/K07509%09yellow/K07513%09yellow/K07514%09yellow/K08765%09yellow/K13239%09yellow
/K01897%09yellow/K07513%09yellow/K07514%09yellow/K08751%09yellow/K08753%09yellow/K08[illegible]

/K01897%09yellow/K07513%09yellow/K07514%09yellow/K13239%09yellow/K13299%09yellow

/K01946%09yellow/K03841%09yellow/K07509%09yellow/K07513%09yellow/K07514%09yellow

/K00699%09yellow/K01640%09yellow/K01895%09yellow/K01946%09yellow/K03841%09yellow/K07

/K00699%09yellow/K00799%09yellow/K01061%09yellow/K01071%09yellow/K01077%09yellow/K01[illegible]

640%09yellow/K01672%09yellow/K01784%09yellow/K01827%09yellow/K01895%09yellow/K01897%

%09yellow/K01930%09yellow/K01946%09yellow/K03841%09yellow/K04717%09yellow/K07424%09y

ellow/K07509%09yellow/K07513%09yellow/K07514%09yellow/K09680%09yellow/K13299%09yello

w/K13506%09yellow/K18123%09yellow/K22283%09yellow/K23741%09yellow
