# Supplementary material for: Comparative analysis of liver transcriptome reveals adaptive responses to hypoxia environmental condition in Tibetan chicken
Source: Anim Biosci. 2023 Aug 23;37(1):28–38. doi: 10.5713/ab.23.0126 (PMC10766467; doi:10.5713/ab.23.0126)
Supplement: Supplementary file 10 [file ab-23-0126-Supplementary-Table-10.pdf]

Table S10

layer1	layer2	layer3	ID	Input.num	Backgrour	pvalue	FDR	Input
1. Metabo	1.3 Lipid n	Steroid bi	ko00100	5	20	0.000111	0.014077	DHCR24|D
1. Metabo	1.9 Metab	Sesquiterp	ko00909	2	2	0.000631	0.040076	FDFT1|SQI
4. Cellular	4.2 Cell gr	Cell cycle	ko04110	10	118	0.000776	0.032858	BUB1|CCN
1. Metabo	1.1 Carbol	Pyruvate r	ko00620	4	36	0.012284	0.390003	ACACA|AL
1. Metabo	1.3 Lipid n	Fatty acid	ko00061	3	20	0.013093	0.332563	ACACA|A(
1. Metabo	1.0 Global	Biosynthes	ko01130	11	204	0.014203	0.300639	ACACA|AL
1. Metabo	1.0 Global	Biosynthes	ko01110	18	406	0.014376	0.260828	ACACA|A(
1. Metabo	1.0 Global	Fatty acid	ko01212	5	59	0.016138	0.256193	ACACA|A(
5. Organis	5.2 Endoc	PPAR sign	ko03320	5	69	0.029636	0.418191	ACSBG2|C
2. Genetic	2.4 Replica	DNA repli	ko03030	3	32	0.045723	0.580684	MCM3|M(
1. Metabo	1.1 Carbol	C5-Brancl	ko00660	1	2	0.049696	0.573765	ACOD1
1. Metabo	1.3 Lipid n	Primary bi	ko00120	2	15	0.053409	0.565249	CYP7A1|C
1. Metabo	1.3 Lipid n	Glycerolip	ko00561	4	61	0.067178	0.65628	AGPAT2|A
1. Metabo	1.9 Metab	Limonene	ko00903	1	4	0.096931	0.879299	ALDH1A3
1. Metabo	1.5 Amino	Phenylalar	ko00400	1	4	0.096931	0.820679	IL4I1
1. Metabo	1.3 Lipid n	Glyceroph	ko00564	5	98	0.100909	0.800968	AGPAT2|C
1. Metabo	1.1 Carbol	Amino su	ko00520	3	46	0.10879	0.812723	HKDC1|LC
5. Organis	5.5 Excret	Collecting	ko04966	2	23	0.113035	0.797525	ATP6V0D2
1. Metabo	1.9 Metab	Insect hor	ko00981	1	5	0.119663	0.799851	ALDH1A3
1. Metabo	1.5 Amino	Valine, leu	ko00280	3	49	0.12519	0.794956	AACS|ALD
1. Metabo	1.11 Xeno	Chloroalka	ko00625	1	6	0.141825	0.857702	ALDH1A3
1. Metabo	1.1 Carbol	Glycolysis	ko00010	3	56	0.166507	0.961197	ALDH1A3|
1. Metabo	1.3 Lipid n	Biosynthes	ko01040	2	31	0.182913	0.999692	ELOVL2|S(
1. Metabo	1.1 Carbol	Starch anc	ko00500	2	31	0.182913	0.967914	HKDC1|U(
3. Environ	3.2 Signal	FoxO sign	ko04068	5	120	0.185032	0.939965	CCNB3|CD
1. Metabo	1.10 Biosy	Isoquinoli	ko00950	1	10	0.225049	0.999692	IL4I1
1. Metabo	1.1 Carbol	Galactose	ko00052	2	36	0.229142	0.999692	HKDC1|U(
1. Metabo	1.3 Lipid n	Fatty acid	ko00071	2	36	0.229142	0.999692	ACSBG2|A
4. Cellular	4.2 Cell gr	p53 signal	ko04115	3	68	0.244476	0.999692	CDK6|GAD
1. Metabo	1.0 Global	Carbon m	ko01200	4	106	0.27741	0.999692	GLDC|HKD
1. Metabo	1.5 Amino	Tryptopha	ko00380	2	42	0.285422	0.999692	ALDH1A3|
1. Metabo	1.5 Amino	Phenylalar	ko00360	1	14	0.300227	0.999692	IL4I1
1. Metabo	1.1 Carbol	Ascorbate	ko00053	1	15	0.317857	0.999692	ALDH1A3
1. Metabo	1.5 Amino	Arginine a	ko00330	2	46	0.322784	0.999692	ALDH1A3|
5. Organis	5.2 Endoc	Renin-ang	ko04614	1	16	0.335044	0.999692	ENPEP
2. Genetic	2.3 Foldin	Protein pr	ko04141	5	152	0.336286	0.999692	DNAJB11|
5. Organis	5.2 Endoc	Insulin sig	ko04910	4	120	0.357204	0.999692	ACACA|FA
5. Organis	5.2 Endoc	Thyroid h	ko04918	2	50	0.35964	0.999692	HSP90B1|I
1. Metabo	1.4 Nucle	Pyrimidine	ko00240	2	51	0.368744	0.999692	RRM2|TK1
1. Metabo	1.0 Global	Metabolic	ko01100	37	1385	0.374083	0.999692	AACS|ACA
1. Metabo	1.5 Amino	Lysine deg	ko00310	2	53	0.386796	0.999692	ALDH1A3|
5. Organis	5.4 Digest	Cholester	ko04979	2	55	0.404619	0.999692	ANGPTL3|
1. Metabo	1.11 Xeno	Drug meta	ko00983	2	56	0.413438	0.999692	RRM2|TK1
1. Metabo	1.1 Carbol	Pentose a	ko00040	1	21	0.414717	0.999692	UGP2
1. Metabo	1.9 Metab	Terpenoid	ko00900	1	21	0.414717	0.999692	FDPS
1. Metabo	1.5 Amino	Histidine r	ko00340	1	22	0.429472	0.999692	ALDH1A3
2. Genetic	2.3 Foldin	Protein ex	ko03060	1	22	0.429472	0.999692	HSPA5
1. Metabo	1.1 Carbol	Butanoate	ko00650	1	23	0.443855	0.999692	AACS
5. Organis	5.4 Digest	Vitamin di	ko04977	1	26	0.484873	0.999692	TCN2
1. Metabo	1.1 Carbol	Citrate cyc	ko00020	1	26	0.484873	0.999692	PDHA2
5. Organis	5.2 Endoc	Adipocyto	ko04920	2	66	0.497712	0.999692	ACSBG2|S
1. Metabo	1.3 Lipid n	Fatty acid	ko00062	1	27	0.497864	0.999692	ELOVL2
1. Metabo	1.6 Metab	beta-Alan	ko00410	1	28	0.510529	0.999692	ALDH1A3
5. Organis	5.5 Excret	Aldostero	ko04960	1	29	0.522875	0.999692	SGK1
3. Environ	3.2 Signal	AMPK sigr	ko04152	3	112	0.53829	0.999692	ACACA|FA
1. Metabo	1.1 Carbol	Propanoat	ko00640	1	31	0.546644	0.999692	ACACA

1. Metabo	1.1 Carbol	Glyoxylate	ko00630	1	31	0.546644	0.999692	GLDC
5. Organis	5.6 Nervo	Synaptic v	ko04721	2	73	0.551937	0.999692	ATP6V0D2
5. Organis	5.2 Endoc	Progester	ko04914	2	73	0.551937	0.999692	BUB1|CCN
1. Metabo	1.5 Amino	Tyrosine n	ko00350	1	33	0.569233	0.999692	IL4I1
1. Metabo	1.5 Amino	Alanine, a	ko00250	1	33	0.569233	0.999692	IL4I1
4. Cellular	4.2 Cell gr	Cellular se	ko04218	4	160	0.575517	0.999692	CCNB3|CD
5. Organis	5.2 Endoc	Glucagon	ko04922	2	78	0.588076	0.999692	ACACA|PD
5. Organis	5.4 Digest	Carbohydr	ko04973	1	35	0.5907	0.999692	HKDC1
5. Organis	5.1 Immur	Toll and Ir	ko04624	1	35	0.5907	0.999692	DUOX1
3. Environ	3.2 Signal	MAPK sigr	ko04010	6	247	0.592647	0.999692	CACNA1H
3. Environ	3.3 Signali	ECM-rece	ko04512	2	80	0.601915	0.999692	ITGB6|LAN
1. Metabo	1.1 Carbol	Fructose a	ko00051	1	37	0.6111	0.999692	HKDC1
5. Organis	5.4 Digest	Fat digesti	ko04975	1	37	0.6111	0.999692	AGPAT2
5. Organis	5.4 Digest	Mineral ak	ko04978	1	38	0.620917	0.999692	SLC26A9
3. Environ	3.2 Signal	PI3K-Akt s	ko04151	7	297	0.623392	0.999692	CDK6|FAS
1. Metabo	1.3 Lipid n	Steroid hc	ko00140	1	39	0.630487	0.999692	CYP7A1
1. Metabo	1.5 Amino	Glycine, se	ko00260	1	39	0.630487	0.999692	GLDC
1. Metabo	1.7 Glycan	Various ty	ko00513	1	40	0.639817	0.999692	ALG12
1. Metabo	1.8 Metab	Retinol me	ko00830	1	40	0.639817	0.999692	RDH11
5. Organis	5.4 Digest	Protein dig	ko04974	2	86	0.641322	0.999692	DPP4|KCN
1. Metabo	1.5 Amino	Cysteine a	ko00270	1	43	0.666419	0.999692	IL4I1
5. Organis	5.7 Sensor	Taste tran	ko04742	1	43	0.666419	0.999692	TRPM5
1. Metabo	1.6 Metab	Glutathior	ko00480	1	45	0.683057	0.999692	RRM2
4. Cellular	4.2 Cell gr	Oocyte me	ko04114	2	93	0.683348	0.999692	BUB1|PTT(
1. Metabo	1.7 Glycan	N-Glycan	ko00510	1	48	0.706475	0.999692	ALG12
5. Organis	5.2 Endoc	GnRH secr	ko04929	1	49	0.713892	0.999692	CACNA1H
5. Organis	5.2 Endoc	Cortisol sy	ko04927	1	49	0.713892	0.999692	CACNA1H
5. Organis	5.2 Endoc	Regulatior	ko04923	1	49	0.713892	0.999692	PNPLA3
3. Environ	3.2 Signal	HIF-1 sigr	ko04066	2	99	0.716094	0.999692	HKDC1|PD
5. Organis	5.4 Digest	Bile secret	ko04976	1	51	0.728169	0.999692	CYP7A1
5. Organis	5.4 Digest	Gastric aci	ko04971	1	55	0.754628	0.999692	KCNQ1
1. Metabo	1.2 Energy	Oxidative	ko00190	2	120	0.809054	0.999692	ATP6V0D2
5. Organis	5.2 Endoc	Prolactin s	ko04917	1	66	0.814885	0.999692	SOCS3
5. Organis	5.1 Immur	Natural kil	ko04650	1	67	0.81957	0.999692	FASLG
2. Genetic	2.3 Folding	Ubiquitin i	ko04120	2	125	0.826767	0.999692	SOCS3|UB
5. Organis	5.1 Immur	IL-17 sign	ko04657	1	69	0.828587	0.999692	HSP90B1
4. Cellular	4.2 Cell gr	Apoptosis	ko04210	2	128	0.836674	0.999692	FASLG|GA
5. Organis	5.4 Digest	Salivary se	ko04970	1	71	0.837155	0.999692	LOC10175
5. Organis	5.2 Endoc	Aldosteror	ko04925	1	71	0.837155	0.999692	CACNA1H
3. Environ	3.2 Signal	Jak-STAT	ko04630	2	132	0.849088	0.999692	IL22RA1|S
4. Cellular	4.1 Transp	Lysosome	ko04142	2	133	0.852055	0.999692	ATP6V0D2
4. Cellular	4.3 Cellula	Gap juncti	ko04540	1	76	0.856754	0.999692	GJA1
3. Environ	3.2 Signal	TNF signa	ko04668	1	82	0.877194	0.999692	SOCS3
5. Organis	5.4 Digest	Pancreatic	ko04972	1	85	0.886296	0.999692	KCNQ1
5. Organis	5.8 Develo	Axon rege	ko04361	1	86	0.889178	0.999692	DUSP4
3. Environ	3.2 Signal	TGF-beta	ko04350	1	87	0.891987	0.999692	CDKN2B
5. Organis	5.6 Nervo	Cholinergi	ko04725	1	90	0.899996	0.999692	KCNQ1
4. Cellular	4.1 Transp	Phagosom	ko04145	2	159	0.912733	0.999692	ATP6V0D2
5. Organis	5.8 Develo	Axon guid	ko04360	2	160	0.914522	0.999692	DPYSL2|EF
5. Organis	5.8 Develo	Osteoclast	ko04380	1	97	0.916456	0.999692	SOCS3
5. Organis	5.10 Enviro	Circadian	ko04713	1	100	0.922656	0.999692	CACNA1H
5. Organis	5.2 Endoc	Estrogen s	ko04915	1	101	0.924619	0.999692	HSP90B1
5. Organis	5.6 Nervo	Neurotrop	ko04722	1	110	0.940195	0.999692	FASLG
5. Organis	5.2 Endoc	Thyroid hc	ko04919	1	111	0.941714	0.999692	SLCO1B1
4. Cellular	4.5 Cell m	Regulatior	ko04810	2	183	0.94729	0.999692	FGF19|ITG
5. Organis	5.3 Circula	Adrenergi	ko04261	1	115	0.947415	0.999692	KCNQ1
4. Cellular	4.3 Cellula	Focal adhe	ko04510	2	186	0.950557	0.999692	ITGB6|LAN
4. Cellular	4.2 Cell gr	Necroptos	ko04217	1	124	0.958293	0.999692	FASLG

3. Environ	3.3 Signali	Cytokine-(	ko04060	2	195	0.959241	0.999692	FASLG|IL2
5. Organis	5.1 Immur	T cell rece	ko04660	1	125	0.959353	0.999692	PDCD1
5. Organis	5.2 Endoc	Growth hc	ko04935	1	127	0.961395	0.999692	SOCS3
3. Environ	3.2 Signal	Ras signal	ko04014	2	199	0.962614	0.999692	FASLG|FGI
3. Environ	3.2 Signal	Phospholi	ko04072	1	133	0.966926	0.999692	AGPAT2
1. Metabo	1.4 Nuclec	Purine me	ko00230	1	141	0.973092	0.999692	RRM2
3. Environ	3.2 Signal	mTOR sigı	ko04150	1	144	0.975096	0.999692	SGK1
3. Environ	3.2 Signal	Calcium si	ko04020	1	161	0.983945	0.999692	CACNA1H
3. Environ	3.3 Signali	Cell adhes	ko04514	1	173	0.988228	0.999692	PDCD1
3. Environ	3.2 Signal	Rap1 signa	ko04015	1	188	0.992016	0.999692	FGF19
5. Organis	5.10 Enviro	Thermoge	ko04714	1	241	0.997984	0.999692	PNPLA3
4. Cellular	4.1 Transp	Endocytos	ko04144	1	252	0.998486	0.999692	RBP
3. Environ	3.3 Signali	Neuroacti	ko04080	1	313	0.999692	0.999692	LOC10174

Hyperlink
http://www.genome.jp/kegg-bin/show_pathway?ko00100/K00213%09red/K00511%09red/K0080
http://www.genome.jp/kegg-bin/show_pathway?ko00909/K00511%09red/K00801%09red
http://www.genome.jp/kegg-bin/show_pathway?ko04110/K02091%09red/K02178%09red/K0220
http://www.genome.jp/kegg-bin/show_pathway?ko00620/K00029%09red/K00128%09red/K0016
http://www.genome.jp/kegg-bin/show_pathway?ko00061/K00665%09red/K11262%09red/K1501
http://www.genome.jp/kegg-bin/show_pathway?ko01130/K00128%09red/K00161%09red/K0028
http://www.genome.jp/kegg-bin/show_pathway?ko01110/K00128%09red/K00161%09red/K0021
http://www.genome.jp/kegg-bin/show_pathway?ko01212/K00507%09red/K00665%09red/K1020
http://www.genome.jp/kegg-bin/show_pathway?ko03320/K00029%09red/K00489%09red/K0050
http://www.genome.jp/kegg-bin/show_pathway?ko03030/K02209%09red/K02212%09red/K0254
http://www.genome.jp/kegg-bin/show_pathway?ko00660/K17724%09red
http://www.genome.jp/kegg-bin/show_pathway?ko00120/K00489%09red/K07431%09red
http://www.genome.jp/kegg-bin/show_pathway?ko00561/K00128%09red/K00629%09red/K1350
http://www.genome.jp/kegg-bin/show_pathway?ko00903/K00128%09red
http://www.genome.jp/kegg-bin/show_pathway?ko00400/K03334%09red
http://www.genome.jp/kegg-bin/show_pathway?ko00564/K00629%09red/K01613%09red/K0872
http://www.genome.jp/kegg-bin/show_pathway?ko00520/K00844%09red/K00963%09red/K0118
http://www.genome.jp/kegg-bin/show_pathway?ko04966/K02146%09red/K02154%09red
http://www.genome.jp/kegg-bin/show_pathway?ko00981/K00128%09red
http://www.genome.jp/kegg-bin/show_pathway?ko00280/K00128%09red/K01907%09red/K0333
http://www.genome.jp/kegg-bin/show_pathway?ko00625/K00128%09red
http://www.genome.jp/kegg-bin/show_pathway?ko00010/K00128%09red/K00161%09red/K0084
http://www.genome.jp/kegg-bin/show_pathway?ko01040/K00507%09red/K10205%09red
http://www.genome.jp/kegg-bin/show_pathway?ko00500/K00844%09red/K00963%09red
http://www.genome.jp/kegg-bin/show_pathway?ko04068/K04389%09red/K04402%09red/K0468
http://www.genome.jp/kegg-bin/show_pathway?ko00950/K03334%09red
http://www.genome.jp/kegg-bin/show_pathway?ko00052/K00844%09red/K00963%09red
http://www.genome.jp/kegg-bin/show_pathway?ko00071/K00128%09red/K15013%09red
http://www.genome.jp/kegg-bin/show_pathway?ko04115/K02091%09red/K04402%09red/K1080
http://www.genome.jp/kegg-bin/show_pathway?ko01200/K00029%09red/K00161%09red/K0028
http://www.genome.jp/kegg-bin/show_pathway?ko00380/K00128%09red/K03334%09red
http://www.genome.jp/kegg-bin/show_pathway?ko00360/K03334%09red
http://www.genome.jp/kegg-bin/show_pathway?ko00053/K00128%09red
http://www.genome.jp/kegg-bin/show_pathway?ko00330/K00128%09red/K00318%09red
http://www.genome.jp/kegg-bin/show_pathway?ko04614/K11141%09red
http://www.genome.jp/kegg-bin/show_pathway?ko04141/K09486%09red/K09487%09red/K0949
http://www.genome.jp/kegg-bin/show_pathway?ko04910/K00665%09red/K00844%09red/K0469
http://www.genome.jp/kegg-bin/show_pathway?ko04918/K09487%09red/K09490%09red
http://www.genome.jp/kegg-bin/show_pathway?ko00240/K00857%09red/K10808%09red
http://www.genome.jp/kegg-bin/show_pathway?ko01100/K00029%09red/K00128%09red/K0016
http://www.genome.jp/kegg-bin/show_pathway?ko00310/K00128%09red/K11426%09red
http://www.genome.jp/kegg-bin/show_pathway?ko04979/K00489%09red/K22288%09red
http://www.genome.jp/kegg-bin/show_pathway?ko00983/K00857%09red/K10808%09red
http://www.genome.jp/kegg-bin/show_pathway?ko00040/K00963%09red
http://www.genome.jp/kegg-bin/show_pathway?ko00900/K00787%09red
http://www.genome.jp/kegg-bin/show_pathway?ko00340/K00128%09red
http://www.genome.jp/kegg-bin/show_pathway?ko03060/K09490%09red
http://www.genome.jp/kegg-bin/show_pathway?ko00650/K01907%09red
http://www.genome.jp/kegg-bin/show_pathway?ko04977/K14619%09red
http://www.genome.jp/kegg-bin/show_pathway?ko00020/K00161%09red
http://www.genome.jp/kegg-bin/show_pathway?ko04920/K04696%09red/K15013%09red
http://www.genome.jp/kegg-bin/show_pathway?ko00062/K10205%09red
http://www.genome.jp/kegg-bin/show_pathway?ko00410/K00128%09red
http://www.genome.jp/kegg-bin/show_pathway?ko04960/K13302%09red
http://www.genome.jp/kegg-bin/show_pathway?ko04152/K00507%09red/K00665%09red/K1126
http://www.genome.jp/kegg-bin/show_pathway?ko00640/K11262%09red

http://www.genome.jp/kegg-bin/show_pathway?ko00630/K00281%09red
http://www.genome.jp/kegg-bin/show_pathway?ko04721/K02146%09red/K02154%09red
http://www.genome.jp/kegg-bin/show_pathway?ko04914/K02178%09red/K21771%09red
http://www.genome.jp/kegg-bin/show_pathway?ko00350/K03334%09red
http://www.genome.jp/kegg-bin/show_pathway?ko00250/K03334%09red
http://www.genome.jp/kegg-bin/show_pathway?ko04218/K02091%09red/K04402%09red/K046[illegible]
http://www.genome.jp/kegg-bin/show_pathway?ko04922/K00161%09red/K11262%09red
http://www.genome.jp/kegg-bin/show_pathway?ko04973/K00844%09red
http://www.genome.jp/kegg-bin/show_pathway?ko04624/K13411%09red
http://www.genome.jp/kegg-bin/show_pathway?ko04010/K04381%09red/K04389%09red/K044[illegible]
http://www.genome.jp/kegg-bin/show_pathway?ko04512/K06244%09red/K06589%09red
http://www.genome.jp/kegg-bin/show_pathway?ko00051/K00844%09red
http://www.genome.jp/kegg-bin/show_pathway?ko04975/K13509%09red
http://www.genome.jp/kegg-bin/show_pathway?ko04978/K14706%09red
http://www.genome.jp/kegg-bin/show_pathway?ko04151/K02091%09red/K04389%09red/K0624
http://www.genome.jp/kegg-bin/show_pathway?ko00140/K00489%09red
http://www.genome.jp/kegg-bin/show_pathway?ko00260/K00281%09red
http://www.genome.jp/kegg-bin/show_pathway?ko00513/K03847%09red
http://www.genome.jp/kegg-bin/show_pathway?ko00830/K11153%09red
http://www.genome.jp/kegg-bin/show_pathway?ko04974/K01278%09red/K04926%09red
http://www.genome.jp/kegg-bin/show_pathway?ko00270/K03334%09red
http://www.genome.jp/kegg-bin/show_pathway?ko04742/K04980%09red
http://www.genome.jp/kegg-bin/show_pathway?ko00480/K10808%09red
http://www.genome.jp/kegg-bin/show_pathway?ko04114/K02178%09red/K06635%09red
http://www.genome.jp/kegg-bin/show_pathway?ko00510/K03847%09red
http://www.genome.jp/kegg-bin/show_pathway?ko04929/K04855%09red
http://www.genome.jp/kegg-bin/show_pathway?ko04927/K04855%09red
http://www.genome.jp/kegg-bin/show_pathway?ko04923/K16816%09red
http://www.genome.jp/kegg-bin/show_pathway?ko04066/K00161%09red/K00844%09red
http://www.genome.jp/kegg-bin/show_pathway?ko04976/K00489%09red
http://www.genome.jp/kegg-bin/show_pathway?ko04971/K04926%09red
http://www.genome.jp/kegg-bin/show_pathway?ko00190/K02146%09red/K02154%09red
http://www.genome.jp/kegg-bin/show_pathway?ko04917/K04696%09red
http://www.genome.jp/kegg-bin/show_pathway?ko04650/K04389%09red
http://www.genome.jp/kegg-bin/show_pathway?ko04120/K04696%09red/K06688%09red
http://www.genome.jp/kegg-bin/show_pathway?ko04657/K09487%09red
http://www.genome.jp/kegg-bin/show_pathway?ko04210/K04389%09red/K04402%09red
http://www.genome.jp/kegg-bin/show_pathway?ko04970/K13912%09red
http://www.genome.jp/kegg-bin/show_pathway?ko04925/K04855%09red
http://www.genome.jp/kegg-bin/show_pathway?ko04630/K04696%09red/K05138%09red
http://www.genome.jp/kegg-bin/show_pathway?ko04142/K02146%09red/K02154%09red
http://www.genome.jp/kegg-bin/show_pathway?ko04540/K07372%09red
http://www.genome.jp/kegg-bin/show_pathway?ko04668/K04696%09red
http://www.genome.jp/kegg-bin/show_pathway?ko04972/K04926%09red
http://www.genome.jp/kegg-bin/show_pathway?ko04361/K04459%09red
http://www.genome.jp/kegg-bin/show_pathway?ko04350/K04685%09red
http://www.genome.jp/kegg-bin/show_pathway?ko04725/K04926%09red
http://www.genome.jp/kegg-bin/show_pathway?ko04145/K02146%09red/K02154%09red
http://www.genome.jp/kegg-bin/show_pathway?ko04360/K05102%09red/K07528%09red
http://www.genome.jp/kegg-bin/show_pathway?ko04380/K04696%09red
http://www.genome.jp/kegg-bin/show_pathway?ko04713/K04855%09red
http://www.genome.jp/kegg-bin/show_pathway?ko04915/K09487%09red
http://www.genome.jp/kegg-bin/show_pathway?ko04722/K04389%09red
http://www.genome.jp/kegg-bin/show_pathway?ko04919/K08747%09red
http://www.genome.jp/kegg-bin/show_pathway?ko04810/K06589%09red/K22603%09red
http://www.genome.jp/kegg-bin/show_pathway?ko04261/K04926%09red
http://www.genome.jp/kegg-bin/show_pathway?ko04510/K06244%09red/K06589%09red
http://www.genome.jp/kegg-bin/show_pathway?ko04217/K04389%09red

http://www.genome.jp/kegg-bin/show_pathway?ko04060/K04389%09red/K05138%09red
http://www.genome.jp/kegg-bin/show_pathway?ko04660/K06744%09red
http://www.genome.jp/kegg-bin/show_pathway?ko04935/K04696%09red
http://www.genome.jp/kegg-bin/show_pathway?ko04014/K04389%09red/K22603%09red
http://www.genome.jp/kegg-bin/show_pathway?ko04072/K13509%09red
http://www.genome.jp/kegg-bin/show_pathway?ko00230/K10808%09red
http://www.genome.jp/kegg-bin/show_pathway?ko04150/K13302%09red
http://www.genome.jp/kegg-bin/show_pathway?ko04020/K04855%09red
http://www.genome.jp/kegg-bin/show_pathway?ko04514/K06744%09red
http://www.genome.jp/kegg-bin/show_pathway?ko04015/K22603%09red
http://www.genome.jp/kegg-bin/show_pathway?ko04714/K16816%09red
http://www.genome.jp/kegg-bin/show_pathway?ko04144/K13649%09red
http://www.genome.jp/kegg-bin/show_pathway?ko04080/K04241%09red

9%09red/K02212%09red/K02541%09red/K04402%09red/K04685%09red/K06628%09red/K06635%0

1%09red/K00318%09red/K00511%09red/K00787%09red/K00801%09red/K00844%09red/K00963%0
3%09red/K00281%09red/K00318%09red/K00511%09red/K00629%09red/K00787%09red/K00801%0

1%09red/K00213%09red/K00281%09red/K00318%09red/K00489%09red/K00507%09red/K00511%0

)9red/K00844%09red/K00963%09red/K01613%09red/K03334%09red/K07750%09red/K09828%09re(

)9red/K00629%09red/K00665%09red/K00787%09red/K00801%09red/K00844%09red/K00857%09re(

d/K00963%09red/K01183%09red/K01613%09red/K01907%09red/K02146%09red/K02154%09red/K0

)3334%09red/K03847%09red/K07431%09red/K07750%09red/K08729%09red/K09828%09red/K102(

)5%09red/K10808%09red/K11153%09red/K11262%09red/K11426%09red/K13509%09red/K14156%0

9red/K15013%09red/K16816%09red/K17724%09red
